# Supplementary material for: Reading mixtures of uniform sequence-defined macromolecules to increase data storage capacity
Source: Commun Chem. 2020 Dec 9;3:184. doi: 10.1038/s42004-020-00431-9 (PMC9814948; doi:10.1038/s42004-020-00431-9)
Supplement: Supplementary file 1 — Supplementary Information [file 42004_2020_431_MOESM1_ESM.pdf]

Supplementary Information

Reading the mixture of uniform sequence defined macromolecules to increase data storage capacity

Maximiliane Frölich,[1] Dennis Hofheinz,[2] Michael A. R. Meier[1,3*]

[1] Laboratory of Applied Chemistry, Institute of Organic Chemistry (IOC), Karlsruhe Institute of Technology (KIT), Straße am Forum 7, 76131 Karlsruhe, Germany.

[2] Department of Computer Science, ETH Zürich, Universitätsstrasse 6, 8092 Zürich, Switzerland.

[3] Laboratory of Applied Chemistry, Institute of Biological and Chemical Systems – Functional Molecular Systems (IBCS-FMS), Karlsruhe Institute of Technology (KIT), Hermann-von-Helmholtz-Platz 1, 76344 Eggenstein-Leopoldshafen, Germany.

[*] Laboratory of Applied Chemistry, Institute of Organic Chemistry (IOC), Karlsruhe Institute of Technology (KIT), Straße am Forum 7, 76131 Karlsruhe, Germany.

Email: m.a.r.meier@kit.edu; Web: www.meier-michael.com

Table of contents

1 Supplementary Methods 3
1.1 Materials 3
1.2 Instrumentation 4
1.3 Experimental procedures 6
1.3.1 Synthesis of the monomer M1 6
1.3.1.1 Esterification 6
1.3.1.2 *N*-Formylation 6
1.3.1.3 Dehydration 7
1.3.2 TAG synthesis 8
1.3.2.1 TAG1 synthesis 8
1.3.2.2 TAG2 synthesis 10
1.3.3 Oligomer synthesis with TAG3 12
1.3.3.1 Synthesis of tetramer T1 12
1.3.3.2 Synthesis of hexamer H1 24
1.3.3.3 Synthesis of tetramer T2 33
1.3.3.4 Synthesis of tetramer T3 45
1.3.3.5 Synthesis of tetramer T4 57
1.3.4 Oligomer synthesis with TAG1 69
1.3.4.1 Synthesis of tetramer T5 69
1.3.4.2 Synthesis of hexamer H2 81
1.3.4.3 Synthesis of tetramer T6 90
1.3.4.4 Synthesis of tetramer T7 102
1.3.4.5 Synthesis of tetramer T8 114
1.3.5 Oligomer synthesis with TAG2 126
1.3.5.1 Synthesis of tetramer T9 126
1.3.5.2 Synthesis of hexamer H3 138
1.3.5.3 Synthesis of tetramer T10 147
1.3.5.4 Synthesis of tetramer T11 159
1.3.5.5 Synthesis of tetramer T12 171
1.3.6 Equations 183
1.3.7 Tetramer mixture 184

1.3.8 MS and MS/MS Data's 185

1.4 Supplementary References 197

1 Supplementary Methods

1.1 Materials

The following chemicals were used as received from the following suppliers unless otherwise noted: propionaldehyde **14l** (97% Merck), isobutyraldehyde **14c** (98%, Sigma-Aldrich), 3-methylbutyraldehyde **14g** (≥ 98%, VWR), cyclohexancarboxaldehyde **14j** (98%), heptanal **14b** (≥ 95%,Sigma-Aldrich), octanal **14i** (99%, Sigma-Aldrich), dodecanal **14h** (≥ 95%, VWR), 2-phenylpropanal **14e** (98%, Fisher Scientific), 4-chlorobutyric acid **TAG3** (99% Sigma-Aldrich), succinic anhydride **11** (>99.0%, Sigma-Aldrich), 4-(dimethylamino)-pyridin **12** (DMAP) (99% Alfa Aesar), sodium sulfate **9** (Merck), 2,2,3,3,4,4-heptafluro-1-butanol (98%, Alfa Aesar) **13**, 1*H*,1*H*,2*H*,2*H*-perfluoro-1-octanol (97%, Alfa Aesar) **10**, acetaldehyde (99.5%, Fluka) **14f**, 2-ethylbutanal (98%, TCI) **14a**, nonanal (97%, Alfa Aesar), **14k**, tridecanal (96%, Alfa Aesar) **14d**, 11-aminoundecanoic acid **1** (97%, Sigma-Aldrich), benzyl alcohol **2** (99%, Sigma-Aldrich), thionyl chloride **3** (99%, Sigma-Aldrich), trimethyl orthoformate **5** (99%, Sigma-Aldrich), phosphoryl trichloride **8** (99%, Sigma-Aldrich), diisopropylamine **7** (> 99.5%, Sigma-Aldrich), palladium on activated charcoal **16** (10% palladium basis, Sigma-Aldrich), hydrogen (99,999%, Air Liquide), TLC silica gel F_{254} (Sigma-Aldrich), silica gel 60 (0.040 - 0.063, Sigma-Aldrich and Rocc), cerium(IV)-sulfate (99%, Sigma-Aldrich), phosphomolybdic acid hydrate (99%, Sigma-Aldrich), sodium carbonate (98%, Sigma-Aldrich), sodium hydrogen carbonate (> 95%, Sigma-Aldrich), sodium sulfate (> 99%, anhydrous, Sigma-Aldrich), magnesium sulfate (≥ 99%, Carl Roth), DMSO-d6 (≥ 99.8%, Euriso-top), MeOH-d4 (≥ 99.8%, Euriso-top), CDCl3 (≥ 99.8%, Euriso-top), dichloromethane (DCM, HPLC grade ≥ 99.9%, Sigma-Aldrich), methanol (HPLC grade 99.8%, Acros Organics), tetrahydrofuran (THF, 99.5%, extra dry over molecular sieves, Acros Organics), ethanol (analytical reagent grade, Fisher Scientific), diethyl ether (analytical reagent grade, Fisher Scientific), cyclohexane (technical grade), ethyl acetate (technical grade). All solvents were used without further purification, unless otherwise noted. Water, when used in the synthesis, was de-ionised.

1.2 Instrumentation

NMR

^{1}H, ^{19}F and ^{13}C spectra were recorded at the Karlsruhe Institute of Technology (KIT, Germany) on a Bruker Avance 400 NMR instrument at 400 MHz for ^{1}H NMR, at 376 MHz for ^{19}F and 101 MHz for ^{13}C NMR. ^{1}H spectra were recorded on a Bruker Avance 300 NMR instrument at 300 MHz for ^{1}H NMR or on a Bruker AVANCE DRX at 500 MHz for ^{1}H NMR and 126 MHz for ^{13}C NMR. $CDCl_3$or CD_3OD were used as solvents. Chemical shifts are presented in parts per million (*δ*) relative to the resonance signal at 7.26 ppm (^{1}H, $CDCl_3$) and 77.16 ppm (^{13}C, $CDCl_3$) or 3.31 ppm (^{1}H, CD_3OD) and 49.00 ppm (^{13}C, CD_3OD), respectively. The spin multiplicity and corresponding signal patterns were abbreviated as follows: s = singlet, d = doublet, t = triplet, q = quartet, quint. = quintet, sext. = sextet, m = multiplet and br = broad signal. Coupling constants (*J*) are reported in Hertz (Hz). All measurements were recorded in a standard fashion at 25 °C unless otherwise stated. Full assignment of structures was aided by 2D NMR analysis (COSY, HSQC and HMBC). If isomers of a substance were observed, all species which could be assigned clearly were labelled with additional appendices (a, b, c. etc.). Hereby, the main isomer was labelled with the appendix “a”, the second isomer with appendix “b” and so on.

Size Exclusion Chromatography (SEC) measurements were performed on a SHIMADZU Size Exclusion Chromatography (SEC) system equipped with a SHIMADZU isocratic pump (LCYCLO20AD), a SHIMADZU refractive index detector (24°C) (RID-20A), a SHIMADZU autosampler (SIL-20A) and a VARIAN column oven (510, 50°C). For separation, a three-column setup was used with one SDV 3 µm, 8×50 mm precolumn and two SDV 3 µm, 1000 Å, 3×300 mm columns supplied by PSS, Germany. Tetrahydrofuran (THF) stabilized with 250 ppm butylated hydroxytoluene (BHT, ≥99.9%) supplied by SIGMA-ALDRICH was used at a flow rate of 1.0 mL min^{-1}. Calibration was carried out by injection of eight narrow polymethylmethacrylate (PMMA) standards ranging from 102 to 58300 kDa.

Orbitrap Electrospray-Ionisation Mass Spectrometry (ESI-MS) mass spectra were recorded on a Q Exactive (Orbitrap) mass spectrometer (Thermo Fisher Scientific, San Jose, CA, USA) equipped with an atmospheric pressure ionisation source operating in the nebuliser assisted electrospray mode. The instrument was calibrated in the m/z-range 150-2000 using a standard containing caffeine, Met-Arg-Phe-Ala acetate (MRFA) and a mixture of fluorinated phosphazenes (Ultramark 1621, all from SIGMA-ALDRICH). A constant spray voltage of 3.5 kV, a dimensionless sheath gas of 6, and a sweep gas flow rate of 2 were applied. The capillary voltage and the S-lens RF level were set to 68.0 V and 320 °C, respectively. For the interpretation of the spectra, molecular peaks $[M]^+$, peaks of pseudo molecules $[M+H]^+$ and $[M+Na]^+$ characteristic fragment peaks are indicated with their mass to charge ratio (*m/z*) and their intensity in percent, relative to the most intense peak (100%).

Electron ionisation (EI) mass spectra were recorded on a Finnigan instrument, model MAT 90 (70 eV). 3-nitrobenzyl alcohol (3-NBA) was used as matrix. For the interpretation of the spectra, molecular peaks $[M]^+$, peaks of pseudo molecules $[M+H]^+$ and characteristic fragment peaks are indicated with their mass to charge ratio (*m/z*) and their intensity in percent, relative to the most intense peak (100%).

Fast atom bombardment (FAB) mass spectra were recorded on a Finnigan MAT 95 instrument. The protonated molecule ion is expressed by the term: $[M+H]^+$ and $[M+Na]^+$

Infrared spectra (IR) were recorded on a Bruker Alpha-p instrument in a frequency range from 3998 to 374 cm^{-1} applying KBr and Attenuated Total Reflection (ATR) technology. IR (Type of measurement) ν / cm^{-1} = wave number (signal intensity, molecular oscillation assignment). The signal shape and intensity is reported relative to the signal of highest intensity and was abbreviated in the following pattern: br = brought, vs = very strong, s = strong, m = medium, w = weak, vw = very weak.

All **thin layer chromatography** experiments were performed on silica gel coated aluminium foil (silica gel 60 F_{254}, SIGMA-ALDRICH). Compounds were visualized by staining with Seebach-solution (mixture of phosphomolybdic acid hydrate, cerium(IV)-sulfate, sulfuric acid and water).

1.3 Experimental procedures

1.3.1 Synthesis of the monomer M1

1.3.1.1 Esterification

Monomer **M1** was synthesised according to the reported procedure from Meier *et al.*[1]

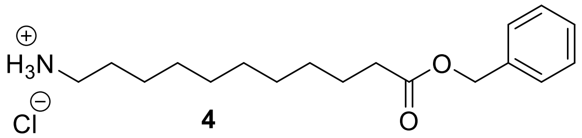

In a 500 mL three necked flask 15.0 g 11-aminoundecanoic acid **1** (74.5 mmol, 1.00 eq.) were suspended in 75 mL THF and 96.7 g (895 mmol, 12.0 eq.) benzyl alcohol **2** were added. The suspension was cooled in an ice bath and subsequently 16.5 mL thionyl chloride **3** (27.1 g, 231 mmol, 3.10 eq.) were added dropwise at 0 °C. After addition of the thionyl chloride **3**, the solution was warmed to room temperature and stirred overnight. The yellow solution was then poured into 500 mL diethylether and stored in the freezer for one hour. The product was the filtered off and dried under high vacuum. The 11-(benzyloxy)-11-oxoundecan-1-aminium chloride **4** was obtained as a white solid in a yield of 72.4% (17.6 g, 53.9 mmol).

^{1}H-NMR (300 MHz, CDO_3D): δ / ppm = 7.53 – 6.77 (m, 5 H, CH_{Ar}), 5.08 (s, 2 H, CH_2), 3.03 – 2.71 (m, 2 H, CH_2), 2.39 – 2.08 (m, 2 H, CH_2), 1.76 – 1.45 (m, 4 H, CH_2), 1.42 – 1.02 (m, 12 H, CH_2). ^{1}H-NMR was in accordance to the literature.[1]

1.3.1.2 *N*-Formylation

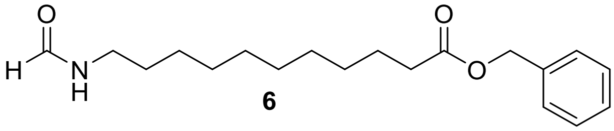

In a 250 mL round bottom flask 17.6 g 11-(benzyloxy)-11-oxoundecan-1-aminium chloride **4** (53.9 mmol, 1.00 eq.) were dissolved in 58.9 mL trimethyl orthoformate **5** (57.2 g, 539 mmol, 10.0 eq.) and heated to 100 °C for 12 hours. Trimethyl orthoformate **5** was removed under reduced pressure and the product was used without further purification. The product **6** was obtained in quantitative yield (17.2 g, 53.9 mmol).

^{1}H-NMR (300 MHz, $CDCl_3$): δ / ppm = 8.01 (s, 1 H, CH), 7.38 – 7.08 (m, 5 H, CH_{Ar}), 5.03 (s, 2 H, CH_2), 4.52 (s, 1 H, NH), 3.31 – 3.18 (m, 2 H, CH_2), 2.26 (t, J = 7.6 Hz, 2 H, CH_2), 1.67 – 1.30 (m, 4 H, CH_2), 1.18 (s, 12 H, CH_2). ^{1}H-NMR was in accordance to the literature.[1]

1.3.1.3 Dehydration

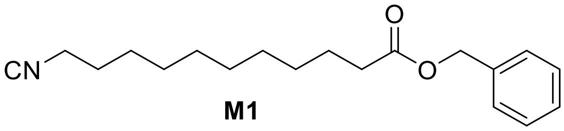

M1

In a 500 mL three necked flask, 17.2 g of benzyl 11-formamidoundecanoate **6** (53.8 mmol, 1.00 eq.) were dissolved in 200 mL DCM, 24.7 mL diisopropylamine **7** (17.7 g, 167 mmol, 3.10 eq.) were added and the reaction mixture was cooled to 0 °C. Subsequently, 6.54 mL phosphorus oxychloride **8** (10.7 g, 69.9 mmol, 1.30 eq.) were added dropwise and the reaction mixture was then stirred at room temperature for two hours. The reaction was quenched by addition of sodium carbonate solution (20 %, 75 mL) at 0 °C. After stirring this mixture for 30 min, 50 mL water and 50 mL DCM were added. The aqueous phase was separated, and the organic layer was washed with water (3 × 80 mL) and brine (80 mL). The combined organic layers were dried over sodium sulfate **9** and the solvent was evaporated under reduced pressure. The crude product was then purified by column chromatography (hexane / ethyl acetate 19:1 → 8:1). The product monomer **M1** was obtained as slightly yellow oil in a yield of 57.1% (9.30 g, 30.8 mmol).

^{1}H-NMR (300 MHz, $CDCl_3$): δ / ppm = 7.47 – 7.03 (m, 5 H, CH_{Ar}), 5.00 (s, 2 H, CH_2), 3.24 (s, 2 H, CH_2), 2.33 – 2.13 (m, 2 H, CH_2), 1.62 – 1.47 (m, 4 H, CH_2), 1.36 – 1.31 (m, 12 H, CH_2). ^{1}H-NMR was in accordance to the literature.[1]

1.3.2 TAG synthesis

1.3.2.1 TAG1 synthesis

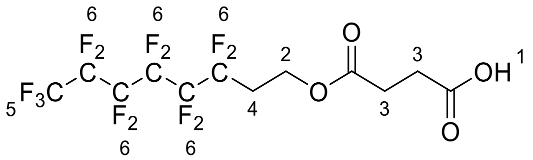

TAG1

In a 500 mL round bottom flask, 606 µL 1*H*,1*H*,2*H*,2*H*-Perfluoro-1-octanol **10** (1.00 g, 2.75 mmol), 316 mg succinic anhydride **11** (3.16 mmol, 1.15 eq.) and 26.8 mg DMAP **12** (219 µmol, 0.08 eq.) were dissolved in 6.00 mL DCM. After 2 days stirring at room temperature, the solution was washed with 10 % $NaHSO_4$ (ca. 15 mL) and 10 mL DCM. The aqueous phase was separated and washed with DCM (2 × 60 mL). The organic layer was washed with water (3 × 80 mL). The combined organic layers were dried over sodium sulfate **9** and the solvent was evaporated under reduced pressure. The product **TAG1** was obtained as a colourless solid in a yield of 83.7% (1.06 g, 2.30 mmol).

R_f = 0.48 in cyclohexane / ethyl acetate (2:1).

IR (ATR): ν / cm^{-1} = 2971.4 (w), 1731.5 (vs), 1697.1 (vs), 1435.2 (w), 1405.6 (m), 1364.0 (s), 1173.0 (vs), 1137.5 (vs), 1080.8 (vs), 1022.1 (s), 947.0 (s), 871.5 (w), 834.0 (s), 781.1 (s), 734.2 (vs), 706.2 (vs), 646.5 (vs), 563.7 (s), 532.2 (s), 506.3 (m), 463.1 (m).

^{1}H NMR (400 MHz, $CDCl_3$): δ / ppm = 11.25 (s, 1 H, OH^1), 4.41 (t, *J* = 6.5 Hz, 2 H, CH_2^2), 2.76 – 2.59 (m, 4 H, CH_2^3), 2.56 – 2.38 (m, 2 H, CH_2^4).

^{13}C NMR (101 MHz, $CDCl_3$): δ / ppm = 178.06, 171.82, 56.81, 30.60, 28.83, 28.79.

^{19}F NMR (376 MHz, $CDCl_3$): δ / ppm =-84.82 – -85.49 (m, 3 F, CF_3^5), -117.80 – -118.24 (m, 2 F, CF_2^6), 125.96 – -126.62 (m, 2 F, CF_2^6), -126.68 – -127.49 (m, 2 F, CF_2^6), -127.68 – -128.41 (m, 2 F, CF_2^6), -130.13 – -130.91 (m, 2 F, CF_2^6). Total integral of CF_2 region normalized with respect to the CF_3^5 group = 10.

ESI-MS [*m/z*]: $[M + H]^+$ calculated for $^{12}C_{12}{}^{1}H_9{}^{16}O_4{}^{19}F_{13}$, 465.0366; found, 465.0354, Δ = 1.2 mmu.

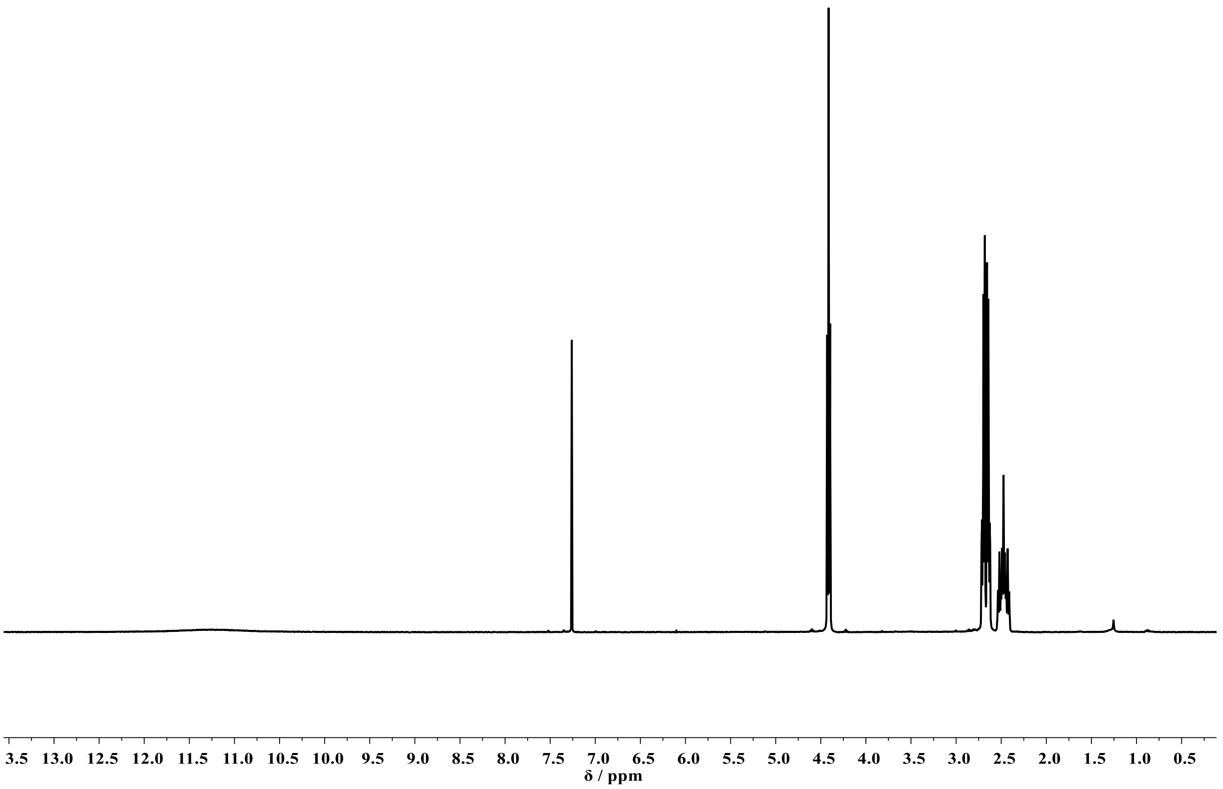

Supplementary Figure 1: ^{1}H-NMR of compound TAG1 measured in $CDCl_3$.

1.3.2.2 TAG2 synthesis

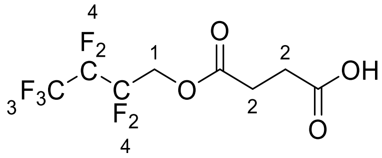

TAG2

In a 500 mL round bottom flask, 625 mL 2,2,3,3,4,4-heptafluro-1-butanol **13** (1.00 g, 4.99 mmol, 1.00 eq.), 575 mg succinc anhydride **11** (5.75 mmol, 1.15 eq.) and 48.9 mg DMAP **12** (400 µmol, 0.08 eq.) were dissolved in 6.00 mL DCM. After 2 days stirring at room temperature the solution was washed with 10 % $NaHSO_4$ (ca. 15 mL) and 10 mL DCM. The aqueous phase was separated and washed with DCM (2 × 60 mL). The organic layer was washed with water (3 × 80 mL). The combined organic layers were dried over sodium sulfate **9** and the solvent was evaporated under reduced pressure. The product **TAG2** was obtained as a colourless solid in a yield of 85.0% (1.28 g, 424 mmol).

IR (ATR): ν / cm^{-1} = 2934.2 (w), 1749.3 (s), 1690.9 (s), 1454.2 (w), 1420.8 (m), 1400.3 (m), 1367.9 (m), 1350.8 (m), 1302.6 (m), 1280.5 (m), 1255.6 (s), 1223.6 (vs), 1175.1 (vs), 1143.5 (vs), 1120.4 (vs), 1030.1 (m), 1018.2 (m), 991.7 (s), 974.6 (m), 950.2 (s), 913.0 (vs), 846.3 (m), 780.6 (w), 733.2 (vs), 689.4 (m), 654.5 (w), 631.6 (w), 588.6 (w), 539.9 (s), 437.3 (w).

^{1}H NMR (400 MHz, $CDCl_3$): δ / ppm = 4.76 – 4.39 (m, 2 H, CH_2^{1}), 2.73 (s, 4 H, CH_2^{2}).

^{13}C NMR (101 MHz, $CDCl_3$): δ / ppm = 177.97, 170.71, 59.67, 28.73, 28.47.

^{19}F NMR (376 MHz, $CDCl_3$): δ / ppm = -85.27 (t, J = 9.7 Hz, 3 F, CF_3^{3}), -123.85 – -126.58 (m, 2 F, CF_2^{4}), -131.47 – -134.72 (m, 2 F, CF_2^{4}). Total integral of CF_2 region normalized with respect to the CF_3^{3} group = 4.

ESI-MS [*m/z*]: $[M + Na]^+$ calculated for $^{12}C_8{}^{1}H_7{}^{16}O_4{}^{19}F_7$, 323.0125; found, 323.0117, Δ = 0.8 mmu.

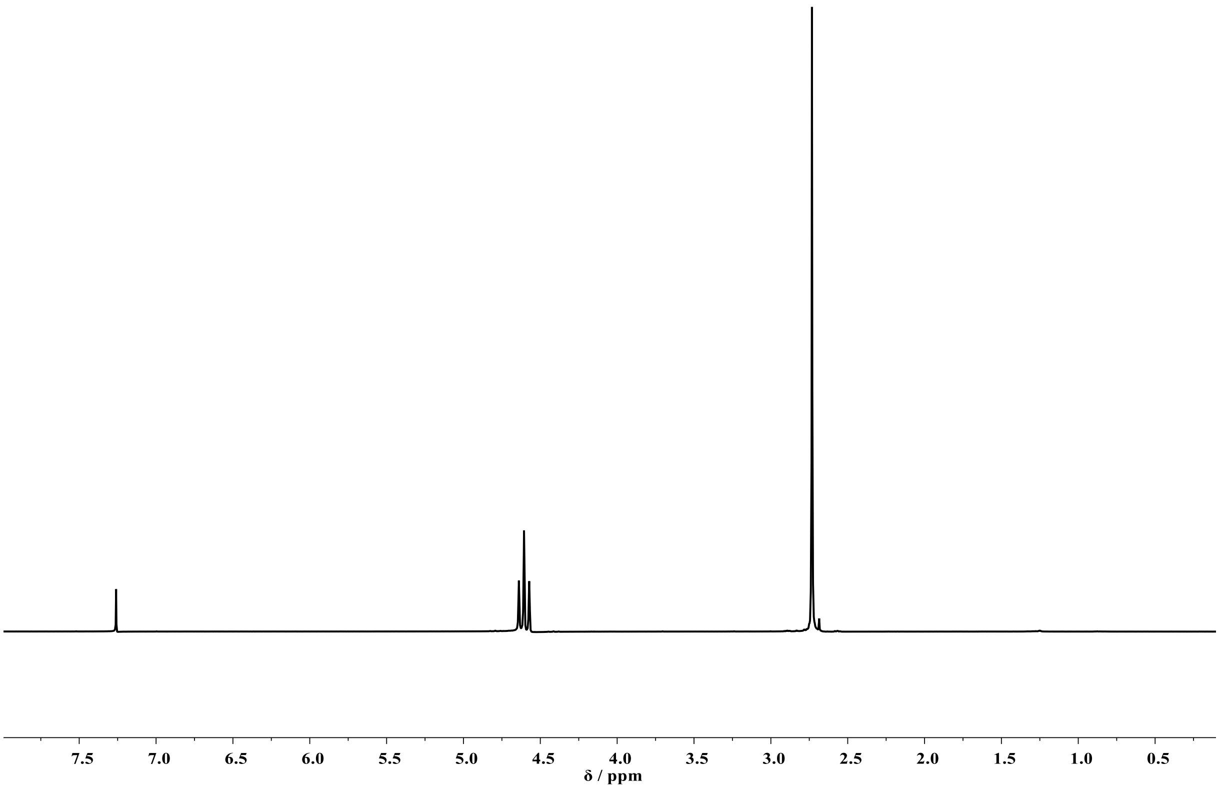

Supplementary Figure 2: ^{1}H-NMR of compound TAG2 measured in $CDCl_3$.

1.3.3 Oligomer synthesis with TAG3

1.3.3.1 Synthesis of tetramer T1

Passerini reaction

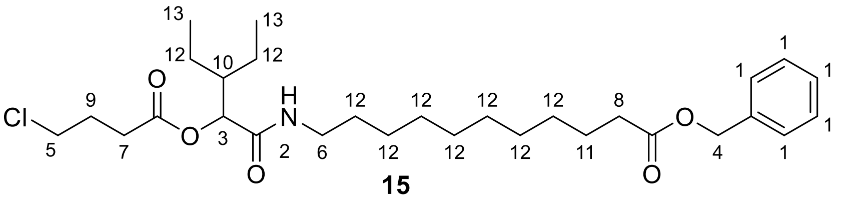

15

In a 50 mL round bottom flask, 205 µL 4-chlorobutyric acid **TAG3** (254 mg, 2.07 mmol, 1.00 eq.) was stirred in 2.00 mL DCM, subsequently 328 µL 2-ethybutanal **14a** (311 mg, 3.11 mmol, 1.50 eq.) and 936 mg of the monomer **M1** (3.11 mmol, 1.50 eq.) were added. The resulting reaction mixture was stirred at room temperature for 3 days. Afterwards, the crude mixture was dried under reduced pressure. The residue was adsorbed onto celite® and purified *via* column chromatography on silica gel eluting with a graduent solvent mixture of ethyl acetate and cyclohexane (8:1 → 6:1) to yield product **15** as a yellow, highly viscous oil. (992 mg, 1.88 mmol, 90.8%).

R_f: 0.52 in cyclohexane / ethyl acetate (2:1).

IR (ATR): ν / cm^{-1} = 3326.0 (vw), 2926.4 (m), 2854.3 (w), 1735.5 (vs), 1654.2 (s), 1531.1 (m), 1456.1 (m), 1379.6 (w), 1142.9 (s), 1004.2 (m), 785.6 (vw), 735.0 (m), 697.1 (m), 650.8 (w).

^{1}H NMR (400 MHz, $CDCl_3$): δ / ppm = 7.40 – 7.28 (m, 5 H, CH_{Ar}^{1}), 6.03 (t, *J* = 5.9 Hz, 1 H, NH^{2}), 5.28 (d, *J* = 3.8 Hz, 1 H, CH^{3}), 5.10 (s, 2 H, CH_2^{4}), 3.68 – 3.50 (m, 2 H, CH_2^{5}), 3.36 – 3.15 (m, 2 H, CH_2^{6}), 2.60 (td, *J* = 7.1, 2.5 Hz, 2 H, CH_2^{7}), 2.33 (t, *J* = 7.5 Hz, 2 H, CH_2^{8}), 2.18 – 2.07 (m, 2 H, CH_2^{9}), 1.88 – 1.77 (m, 1 H, CH^{10}), 1.69 – 1.33 (m, 2 H, CH_2^{11}), 1.53 – 1.13 (m, 18 H, CH_2^{12}), 0.95 – 0.86 (m, 6 H, CH_3^{13}).

^{13}C NMR (101 MHz, $CDCl_3$): δ / ppm = 173.74, 171.58, 169.57, 136.21, 128.60, 128.22, 75.47, 66.12, 63.43, 44.02, 43.60, 39.32, 34.38, 31.17, 29.57, 29.49, 29.39, 29.26, 29.15, 26.91, 25.00, 22.33, 21.97, 11.67, 11.65.

ESI-MS [*m*/z]: $[M+H]^+$ calculated for $^{12}C_{29}{}^{1}H_{46}{}^{16}O_5{}^{14}N^{35}Cl$: 524.3137; found: 524.3126; Δ = 1.1 mmu.

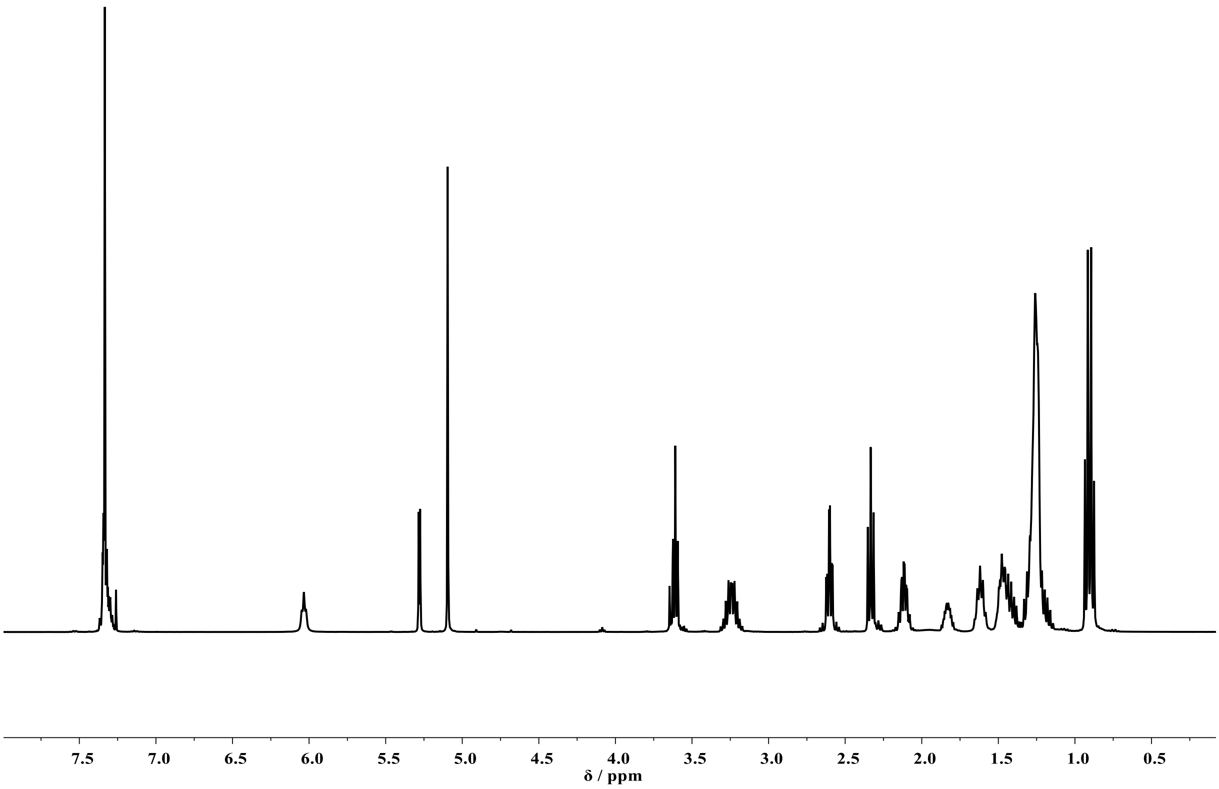

Supplementary Figure 3: ^{1}H-NMR of compound 15 measured in $CDCl_3$.

Deprotection

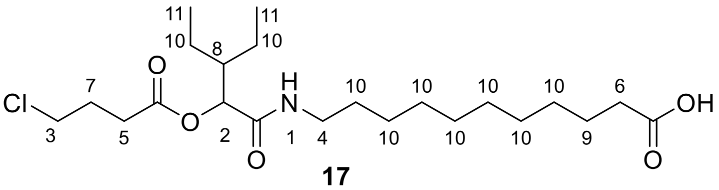

17

In a 50 mL round bottom flask, 444 mg of the passerini product **15** (850 µmol, 1.00 eq.) were dissolved in 2.00 mL ethyl acetate and 2.00 mL THF. Afterwards, 99.0 mg (20 wt%) palladium on activated charcoal **16** were added. Subsequently, the mixture was purged with hydrogen (3 balloons) and stirred under hydrogen atmosphere overnight. The heterogeneous catalyst was filtered off and the solvent was evaporated under reduced pressure. The product **17** was obtained as a pale highly viscous oil in a yield of 99.2% (365 mg, 843 µmol).

IR (ATR): ν / cm^{-1} = 3307.3 (w), 2926.2 (m), 2854.6 (w), 1736.9 (m), 1648.5 (m), 1535.7 (w), 1459.4 (w), 1379.7 (vw), 1175.9 (w), 1140.4 (w), 1047.1 (vw), 1006.5 (vw), 784.9 (vw), 722.2 (vw), 649.7 (vw).

^{1}H NMR (400 MHz, $CDCl_3$): δ / ppm = 5.97 (t, J = 5.9 Hz, 1 H, NH^1), 5.23 (d, J = 3.8 Hz, 1 H, CH^2), 3.62 – 3.52 (m, 2 H, $CH_2{}^3$), 3.30 – 3.11 (m, 2 H, $CH_2{}^4$), 2.56 (td, J = 7.1, 2.3 Hz, 2 H, $CH_2{}^5$), 2.27 (t,

J = 7.5 Hz, 2 H, CH_2^6), 2.13 – 2.01 (m, 2 H, CH_2^7), 1.83 – 1.72 (m, 1 H, CH^8), 1.60 – 1.51 (m, 2 H, CH_2^9), 1.47 – 1.09 (m, 18 H, CH_2^{10}), 0.91 – 0.79 (m, 6 H, CH_2^{11}).

^{13}C NMR (101 MHz, $CDCl_3$): δ / ppm = 179.16, 171.69, 169.77, 75.53, 44.09, 43.62, 39.41, 34.18, 31.22, 29.57, 29.46, 29.36, 29.23, 29.10, 27.46, 26.92, 24.83, 22.35, 22.00, 11.71, 11.68.

ESI-MS [*m/z*]: $[M + H]^+$ calculated for $^{12}C_{22}{}^{1}H_{40}{}^{16}O_5{}^{14}N^{35}Cl$, 434.2668; found, 434.2659, Δ = 0.9 mmu.

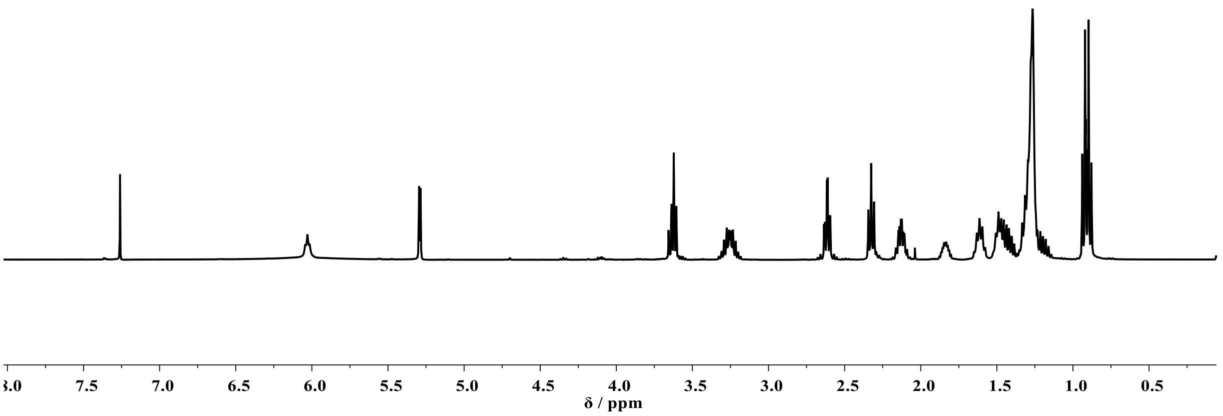

Supplementary Figure 4: ^{1}H-NMR of compound 17 measured in $CDCl_3$.

Passerini reaction

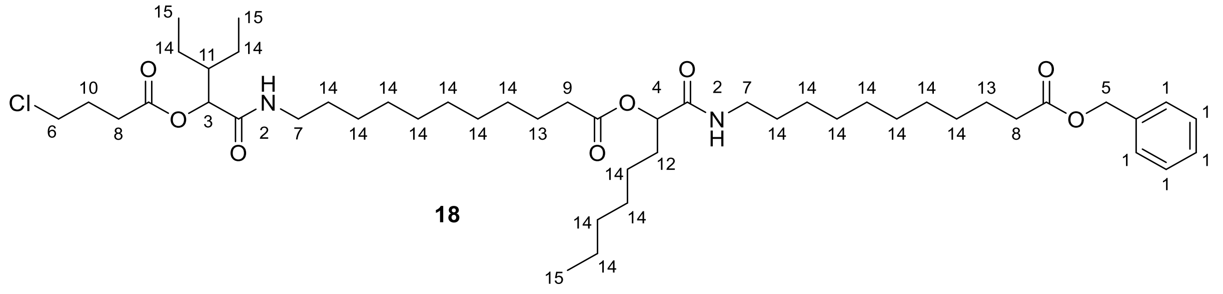

18

In a 50 mL round bottom flask, 313 mg **17** (722 µmol, 1.00 eq.) was stirred in 2.00 mL DCM, subsequently 151 µL heptanal **14b** (124 mg, 1.08 mmol, 1.50 eq.) and 326 mg of the monomer **M1** (1.08 mmol, 1.50 eq.) were added. The resulting reaction mixture was stirred at room temperature for 3 days. Afterwards, the crude mixture was dried under reduced pressure. The residue was adsorbed onto

celite® and purified *via* column chromatography on silica gel eluting with a gradual solvent mixture of ethyl acetate and cyclohexane (6:1 → 2:1) to yield the passerini product **18** as a yellow highly viscous oil. (533 mg, 630 µmol, 87.3%).

R_f: 0.20 in cyclohexane / ethyl acetate (3:1).

IR (ATR): ν / cm^{-1} = 3305.8 (vw), 2924.9 (s), 2854.1 (m), 1737.2 (vs), 1653.5 (s), 1533.8 (m), 1456.8 (m), 1377.3 (w), 1163.3 (s), 1005.5 (w), 732.4 (w), 696.8 (m), 651.4 (w).

^{1}H NMR (400 MHz, $CDCl_3$): δ / ppm = 7.40 – 7.28 (m, 5 H, CH_{Ar}^1), 6.04 – 5.95 (m, 2 H, NH^2), 5.28 (d, J = 3.8 Hz, 1 H, CH^3), 5.18 – 5.12 (m, 1 H, CH^4), 5.10 (s, 2 H, CH_2^5), 3.62 (t, J = 6.2 Hz, 2 H, CH_2^6), 3.33 – 3.17 (m, 4 H, CH_2^7), 2.61 (td, J = 7.1, 2.7 Hz, 2 H, CH_2^8), 2.41 – 2.30 (m, 4 H, CH_2^9), 2.18 – 2.07 (m, 2 H, CH_2^{10}), 1.90 – 1.74 (m, 3 H, CH^{11}, CH_2^{12}), 1.68 – 1.57 (m, 4 H, CH_2^{13}), 1.54 – 1.15 (m, 40H, CH_2^{14}), 0.96 – 0.82 (m, 9 H, CH_3^{15}).

^{13}C NMR (101 MHz, $CDCl_3$): δ / ppm = 173.79, 172.57, 171.62, 169.96, 169.62, 136.23, 128.64, 128.27, 128.26, 75.52, 74.04, 66.17, 44.06, 43.64, 39.35, 39.30, 34.43, 32.02, 31.73, 31.20, 29.65, 29.62, 29.55, 29.45, 29.32, 29.28, 29.20, 27.46, 26.95, 26.94, 25.07, 25.04, 24.81, 22.63, 22.37, 22.01, 14.15, 11.70, 11.69.

ESI-MS [*m*/z]: [M+H]$^+$ calculated for $^{12}C_{48}{}^{1}H_{81}{}^{16}O_{8}{}^{14}N_{2}{}^{35}Cl$: 849.5754; found: 849.5734; Δ = 2.0 mmu.

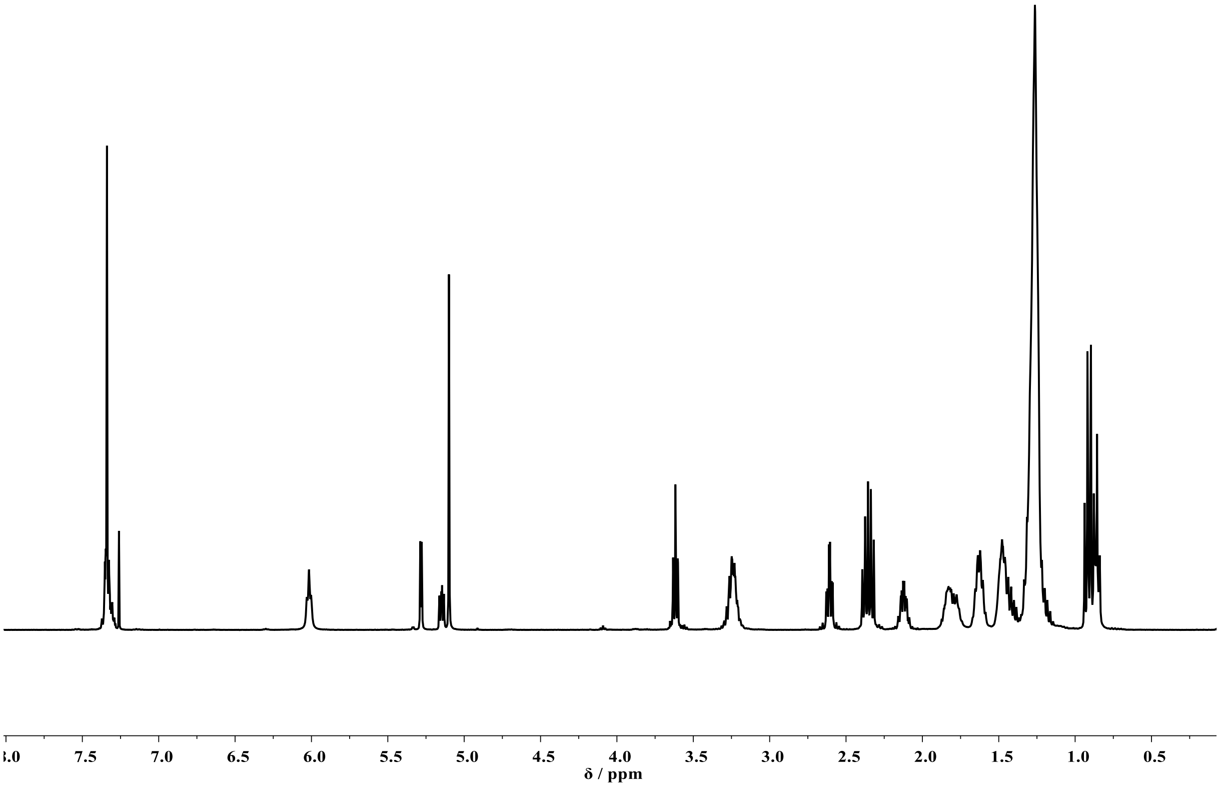

Supplementary Figure 5: ^{1}H-NMR of compound 18 measured in $CDCl_3$.

Deprotection

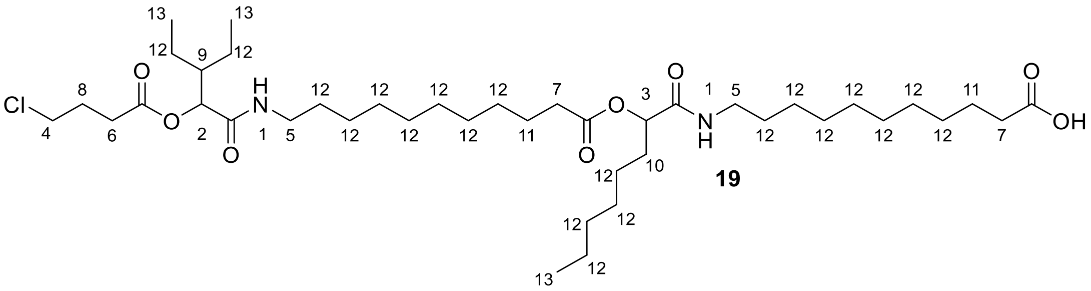

19

In a 50 mL round bottom flask, 474 mg of passerini product **18** (560 µmol, 1.00 eq.) were dissolved in 3.00 mL ethyl acetate and 3.00 mL THF. Afterwards, 95.0 mg (20 wt%) palladium on activated charcoal **16** were added. Subsequently, the mixture was purged with hydrogen (3 balloons) and stirred under hydrogen atmosphere overnight. The heterogeneous catalyst was filtered off and the solvent was evaporated under reduced pressure. The product **19** was obtained as a pale highly viscous oil in a yield of 96.2% (407 mg, 537 µmol).

IR (ATR): ν / cm^{-1} = 3307.0 (vw), 2924.9 (s), 2854.2 (m), 1738.8 (m), 1650.5 (m), 1536.4 (w), 1459.5 (w), 1376.3 (w), 1142.4 (m), 722.2 (vw), 643.9 (vw), 384.8 (vw).

^{1}H NMR (400 MHz, $CDCl_3$): δ / ppm = 6.15 – 6.02 (m, 2 H, NH^{1}), 5.28 (d, J = 3.9 Hz, 1 H, CH^{2}), 5.21 – 5.10 (m, 1 H, CH^{3}), 3.61 (t, J = 6.1 Hz, 2 H, $CH_2{}^{4}$), 3.33 – 3.15 (m, 4 H, $CH_2{}^{5}$), 2.61 (td, J = 7.1, 2.8 Hz, 2 H, $CH_2{}^{6}$), 2.37 (t, J = 7.5 Hz, 2 H, $CH_2{}^{7}$), 2.31 (t, J = 7.5 Hz, 2 H, $CH_2{}^{7}$), 2.18 – 2.05 (m, 2 H, $CH_2{}^{8}$), 1.88 – 1.74 (m, 3 H, CH^{9}, $CH_2{}^{10}$), 1.69 – 1.54 (m, 4 H, $CH_2{}^{11}$), 1.52 – 1.14 (m, 40 H, $CH_2{}^{12}$), 0.95 – 0.81 (m, 9 H, $CH_3{}^{13}$).

^{13}C NMR (101 MHz, $CDCl_3$): δ / ppm = 178.33, 172.62, 171.70, 170.11, 169.81, 75.50, 74.03, 44.07, 43.59, 39.41, 39.31, 34.43, 34.16, 31.99, 31.72, 31.20, 29.58, 29.56, 29.47, 29.37, 29.33, 29.28, 29.25, 29.21, 29.12, 29.01, 27.45, 26.95, 26.89, 25.08, 24.88, 24.81, 22.63, 22.33, 21.97, 14.15, 11.67, 11.65.

ESI-MS [*m/z*]: $[M + H]^+$ calculated for $^{12}C_{41}{}^{1}H_{75}{}^{16}O_{8}{}^{14}N_{2}{}^{35}Cl$, 759.5285; found, 759.5267, Δ = 1.8 mmu.

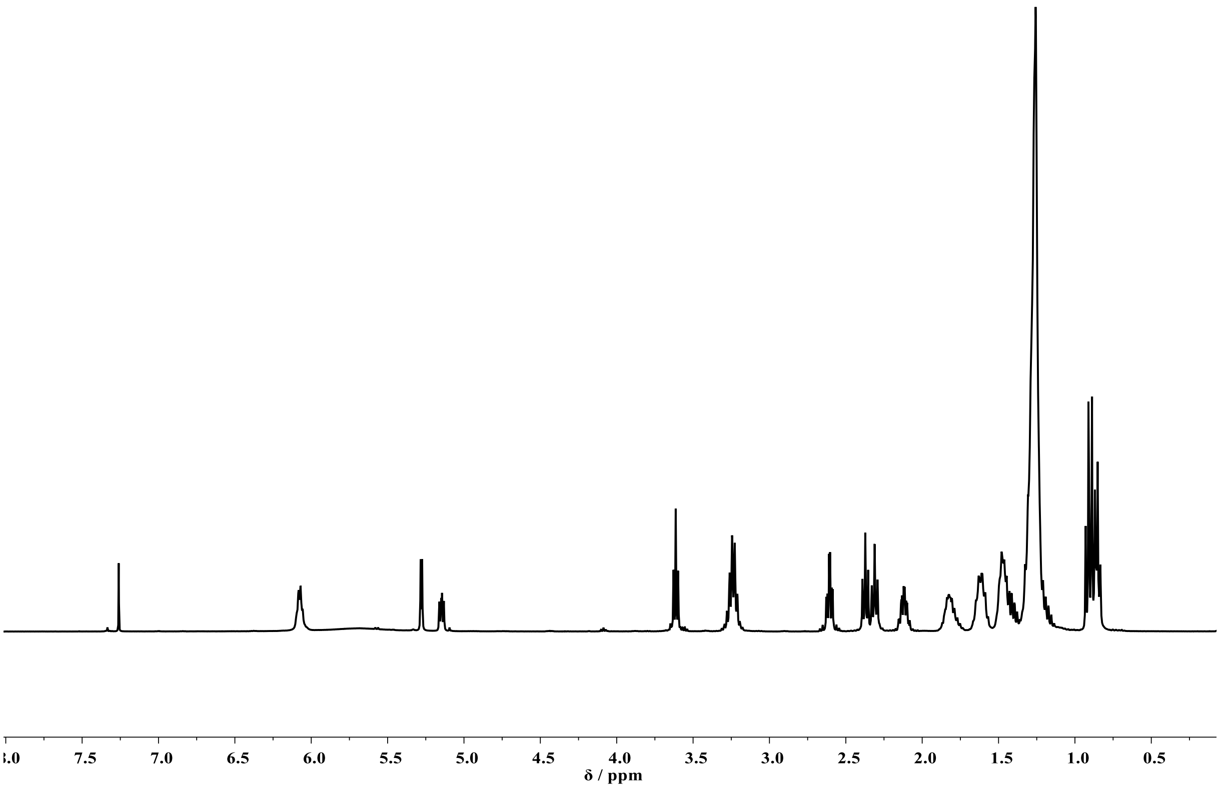

Supplementary Figure 6: ^{1}H-NMR of compound 19 measured in $CDCl_3$.

Passerini reaction

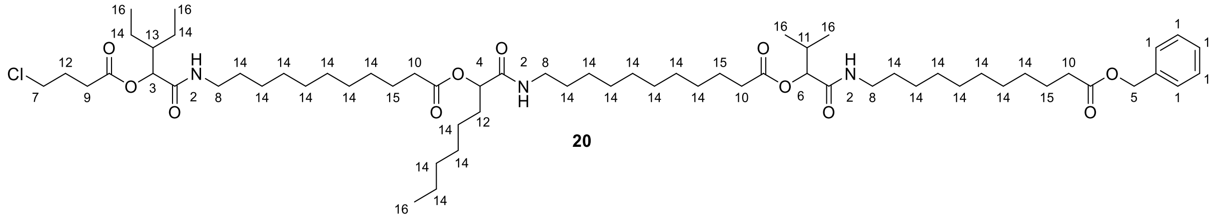

20

In a 50 mL round bottom flask, 325 mg **19** (460 µmol, 1.00 eq.) was stirred in 4.00 mL DCM. Subsequently, 63.0 µL isobutyraldehyde **14c** (50.0 mg, 690 µmol, 1.50 eq.) and 210 mg of the monomer **M1** (690 µmol, 1.50 eq.) were added. The resulting reaction mixture was stirred at room temperature for 3 days. Afterwards, the crude mixture was dried under reduced pressure. The residue was adsorbed onto celite® and purified *via* column chromatography on silica gel eluting with a gradual solvent mixture of ethyl acetate and cyclohexane (5:1 → 2:1) to yield the passerini product **20** as a yellow highly viscous oil. (460 mg, 410 µmol, 89.1%).

R_f: 0.13 in cyclohexane / ethyl acetate (2:1).

IR (ATR): ν / cm^{-1} = 3306.7 (vw), 2924.8 (s), 2853.8 (m), 1737.6 (vs), 1652.7 (vs), 1533.9 (m), 1458.0 (w), 1373.7 (w), 1162.4 (s), 1905.3 (w), 724.8 (w), 697.0 (w), 650.1 (w).

^{1}H NMR (400 MHz, $CDCl_3$): *δ* / ppm = 7.35 – 7.23 (m, 5 H, $CH_{Ar}{}^{1}$), 6.05 – 5.82 (m, 3 H, NH^{2}), 5.23 (d, *J* = 3.8 Hz, 1 H, CH^{3}), 5.12 – 5.05 (m, 1 H, CH^{4}), 5.04 (s, 2 H, $CH_2{}^{5}$), 4.98 (d, *J* = 4.4 Hz, 1 H, $CH_2{}^{6}$), 3.56 (t, *J* = 6.2 Hz, 2 H, $CH_2{}^{7}$), 3.27 – 3.09 (m, 6 H, $CH_2{}^{8}$), 2.55 (td, *J* = 7.1, 3.9 Hz, 2 H, $CH_2{}^{9}$), 2.39 – 2.18 (m, 7 H, CH^{10}, $CH_2{}^{11}$), 2.12 – 2.02 (m, 2 H, $CH_2{}^{12}$), 1.87 – 1.67 (m, 5 H, CH^{13}, $CH_2{}^{14}$), 1.66 – 1.51 (m, 6 H, $CH_2{}^{15}$), 1.48 – 1.08 (m, 54 H, $CH_2{}^{14}$), 0.91 – 0.77 (m, 15 H, $CH_3{}^{16}$).

^{13}C NMR (101 MHz, $CDCl_3$): *δ* / ppm = 173.82, 172.69, 172.60, 171.64, 170.00, 169.64, 169.39, 136.22, 128.66, 128.27, 78.03, 75.50, 74.03, 66.19, 60.52, 44.09, 43.63, 39.36, 39.29, 39.28, 34.43, 34.40, 32.02, 31.74, 31.20, 30.63, 29.69, 29.68, 29.63, 29.57, 29.55, 29.47, 29.47, 29.33, 29.32, 29.24, 29.21, 29.03, 27.46, 26.95, 26.93, 25.12, 25.08, 25.05, 24.82, 22.65, 22.35, 22.00, 21.18, 18.90, 17.07, 14.32, 14.17, 11.71, 11.70.

ESI-MS [*m*/z]: $[M+Na]^+$ calculated for $^{12}C_{64}{}^{1}H_{110}{}^{16}O_{11}{}^{14}N_3{}^{35}Cl$: 1154.7721 found: 1154.7698; Δ = 2.3 mmu.

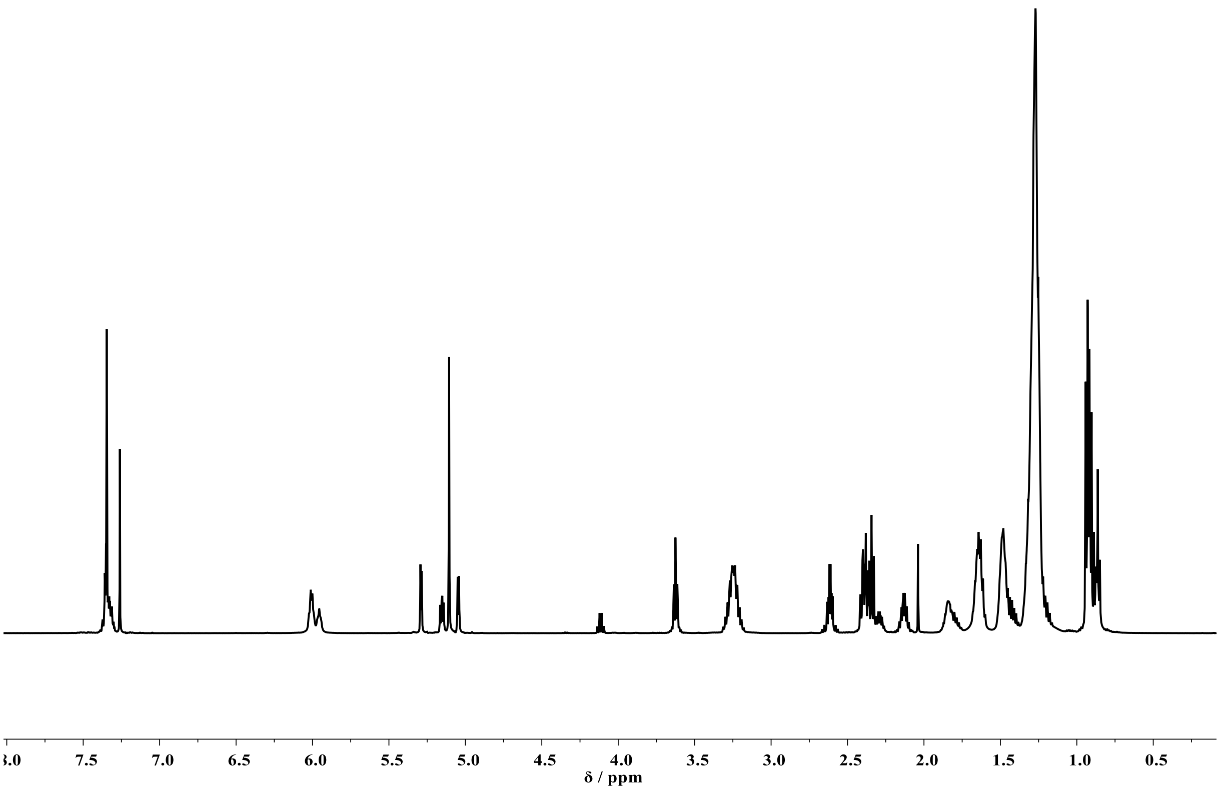

Supplementary Figure 7: ^{1}H-NMR of compound 20 measured in $CDCl_3$.

Deprotection

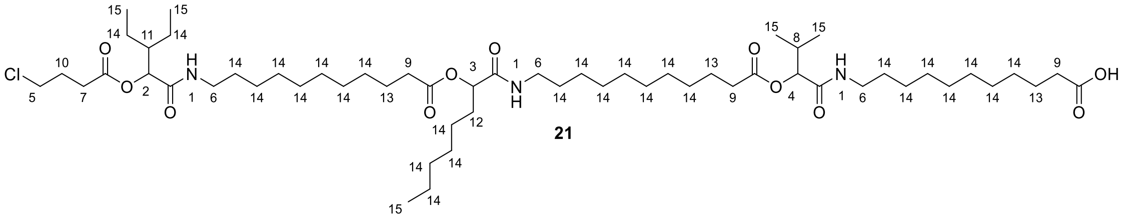

21

In a 50 mL round bottom flask, 400 mg of the passerini product **20** (353 µmol, 1.00 eq.) were dissolved in 2.00 mL ethyl acetate and 2.00 mL THF. Afterwards, 80.0 mg (20 wt%) palladium on activated charcoal **16** were added. Subsequently, the mixture was purged with hydrogen (3 balloons) and stirred under hydrogen atmosphere overnight. The heterogeneous catalyst was filtered off and the solvent was evaporated under reduced pressure. The product **21** was obtained as a pale highly viscous oil in a yield of 98.6% (363 mg, 348 µmol).

IR (ATR): ν / cm^{-1} = 3305.7 (vw), 2924.5 (s), 2853.6 (m), 1738.3 (s), 1650.5 (s), 1536.1 (s), 1460.6 (m), 1371.4 (w), 1165.6 (s), 1008.1 (w), 722.1 (w), 650.1 (w).

^{1}H NMR (400 MHz, $CDCl_3$): δ / ppm = 6.08 (t, J = 5.9 Hz, 1 H, NH^{1}), 6.06 – 5.98 (m, 2 H, NH^{1}), 5.29 (d, J = 3.8 Hz, 1 H, CH^{2}), 5.18 – 5.13 (m, 1 H, CH^{3}), 5.05 (d, J = 4.5 Hz, 1 H, CH^{4}), 3.62 (t, J = 6.2 Hz, 2 H, CH_2^{5}), 3.34 – 3.18 (m, 6 H, CH_2^{6}), 2.62 (td, J = 7.1, 2.6 Hz, 2 H, CH_2^{7}), 2.44 – 2.25 (m, 7 H, CH^{8},CH_2^{9}), 2.18 – 2.08 (m, 2, CH_2^{10}), 1.94 – 1.74 (m, 3 H, CH^{11}, CH_2^{12}), 1.71 – 1.57 (m, 6 H, CH_2^{13}), 1.37 – 1.14 (m, 54 H, CH_2^{14}), 0.98 – 0.82 (m, 15 H, CH_3^{15}).

^{13}C NMR (101 MHz, $CDCl_3$): δ / ppm = 172.71, 172.70, 171.67, 170.17, 169.74, 169.49, 78.09, 75.55, 74.07, 44.10, 43.65, 39.41, 39.39, 39.27, 34.46, 34.44, 32.02, 31.76, 31.23, 30.62, 29.67, 29.62, 29.51, 29.48, 29.42, 29.37, 29.28, 29.23, 29.21, 29.10, 29.04, 27.49, 26.97, 26.90, 25.17, 25.10, 24.92, 24.85 22.67, 22.39, 22.03, 18.92, 17.10, 14.18, 11.73.

ESI-MS [*m/z*]: $[M + H]^{+}$ calculated for $^{12}C_{57}{}^{1}H_{104}{}^{16}O_{11}{}^{14}N_3{}^{35}Cl$, 1042.7432; found, 1042.7412, Δ = 2.0 mmu.

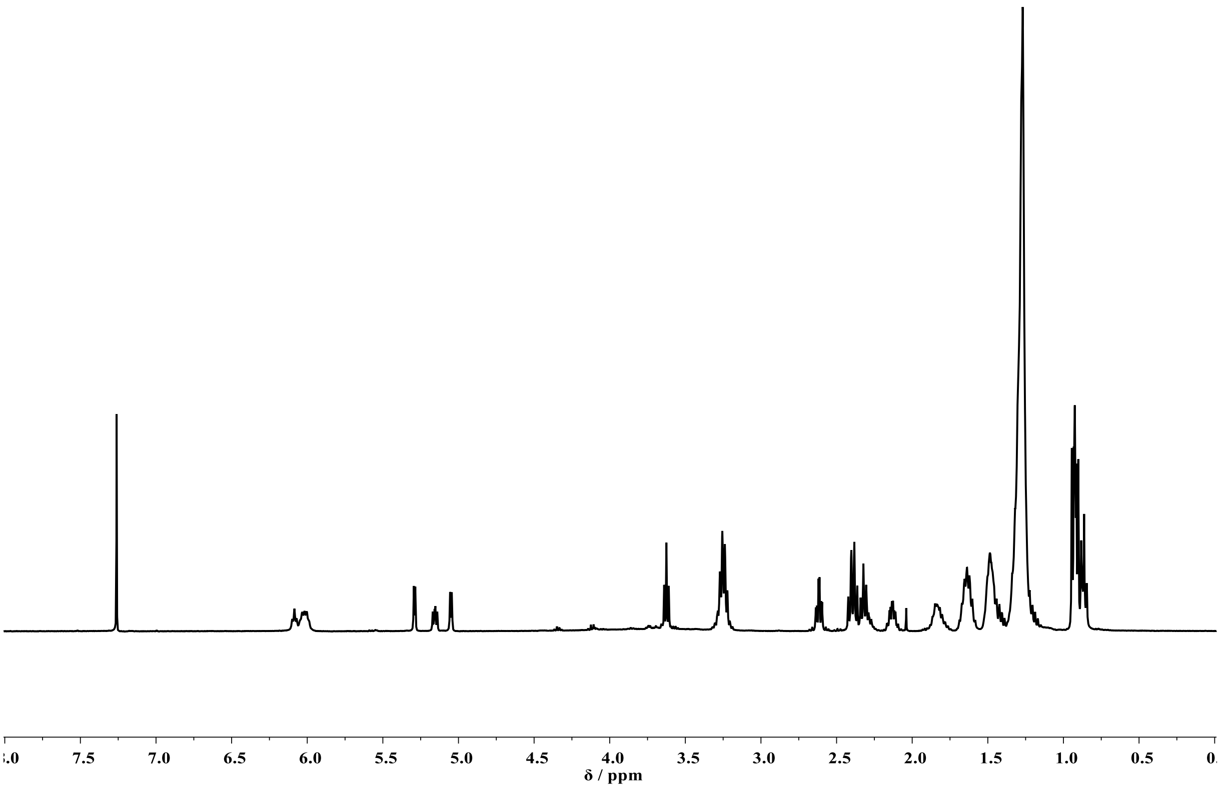

Supplementary Figure 8: ^{1}H-NMR of compound 21 measured in $CDCl_3$.

Passerini reaction

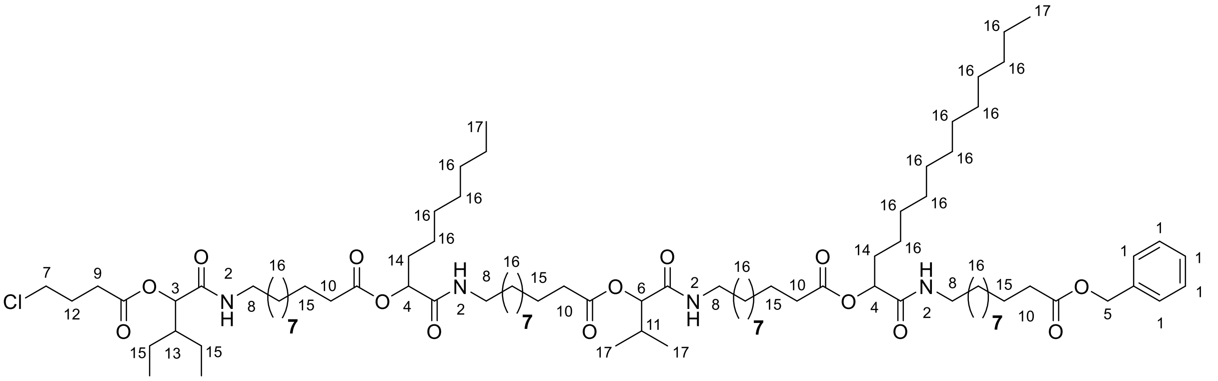

T1

In a 50 mL round bottom flask, 688 mg **21** (659 µmol, 1.00 eq.) was stirred in 2.00 mL DCM. Subsequently, 235 µL tridecanal (196 mg, 989 µmol, 1.50 eq.) and 298 mg of the monomer **M1** (989 µmol, 1.50 eq.) were added. The resulting reaction mixture was stirred at room temperature for 2 days. Afterwards, the crude mixture was dried under reduced pressure. The residue was adsorbed onto celite® and purified *via* column chromatography on silica gel eluting with a gradual solvent mixture of ethyl acetate and cyclohexane (5:1 → 1:1) to yield the passerini product **T1** as a yellow highly viscous oil. (981 mg, 635 µmol, 96.4%).

R_f: 0.75 in cyclohexane / ethyl acetate (1:1).

IR (ATR): *ν* / cm^{-1} = 3304.4 (vw), 2923.0 (s), 2852.8 (m), 1738.5 (s), 1653.1 (s), 1534.7 (m), 1458.5 (m), 1372.7 (w), 1163.1 (s), 1006.7 (w), 723.1 (w), 696.9 (w).

^{1}H NMR (500 MHz, $CDCl_3$): *δ* / ppm = 7.43 – 7.29 (m, 5 H, CH_{Ar}^{1}), 6.08 – 5.93 (m, 4 H, NH^{2}), 5.29 (d, *J* = 3.8 Hz, 1 H, CH^{3}), 5.17 – 5.12 (m, 2 H, CH^{4}), 5.11 (s, 2 H, CH_2^{5}), 5.04 (d, *J* = 4.5 Hz, 1 H, CH^{6}), 3.63 (t, *J* = 6.2 Hz, 2 H, CH_2^{7}), 3.33 – 3.17 (m, 8 H, CH_2^{8}), 2.68 – 2.56 (m, 2 H, CH_2^{9}), 2.43 – 2.26 (m, 9 H, CH^{10}, CH_2^{11}), 2.18 – 2.08 (m, 2 H, CH_2^{12}), 1.91 – 1.74 (m, 5 H, CH^{13}, CH_2^{14}), 1.69 – 1.58 (m, 12 H, CH_2^{15}), 1.54 – 1.16 (m, 84 H, CH_2^{16}), 0.96 – 0.82 (m, 18 H, CH_3^{17}).

^{13}C NMR (126 MHz, $CDCl_3$): *δ* / ppm = 173.82, 172.70, 172.61, 171.65, 169.99, 169.98, 169.64, 169.40, 136.25, 128.67, 128.30, 128.29, 78.05, 75.53, 74.07, 74.05, 66.20, 44.10, 43.65, 39.37, 39.31, 39.28, 34.45, 34.42, 32.05, 31.76, 31.22, 30.65, 29.80, 29.78, 29.76, 29.73, 29.69, 29.68, 29.65, 29.58, 29.50, 29.49, 29.39, 29.35, 29.33, 29.32, 29.31, 29.26, 29.24, 29.04, 27.47, 26.97, 26.96, 25.13, 25.09, 25.07, 24.89, 24.84, 22.82, 22.66, 22.38, 22.02, 18.92, 17.09, 14.26, 14.18, 11.73, 11.71.

ESI-MS [*m*/z]: [M+Na]$^{+}$ calculated for $^{12}C_{89}{}^{1}H_{157}{}^{16}O_{14}{}^{14}N_4{}^{35}Cl$: 1564.1277; found: 1564.1282; Δ = 0.5 mmu.

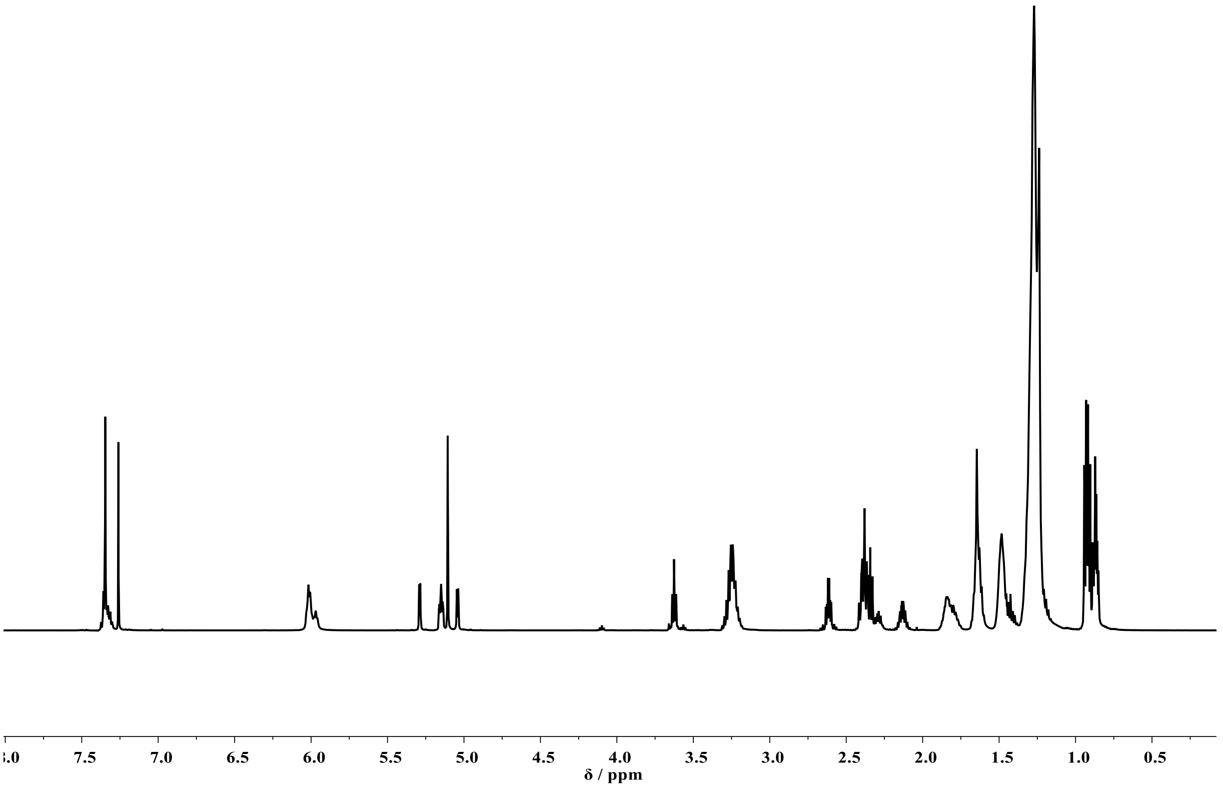

Supplementary Figure 9: ^{1}H-NMR of compound T1 measured in $CDCl_3$.

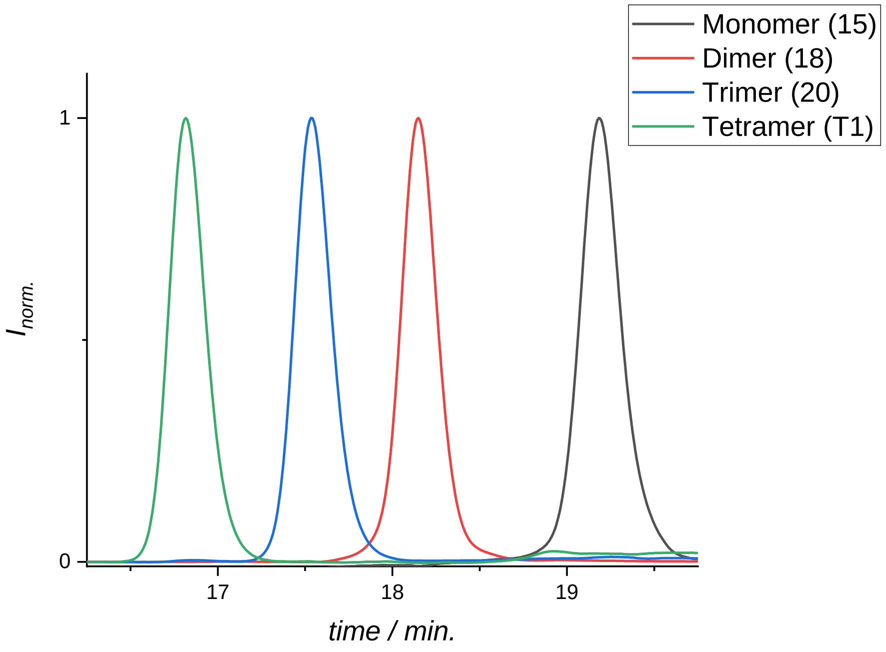

Supplementary Figure 10: SEC traces of the intermediates after each P3CR in the synthesis of product T1.

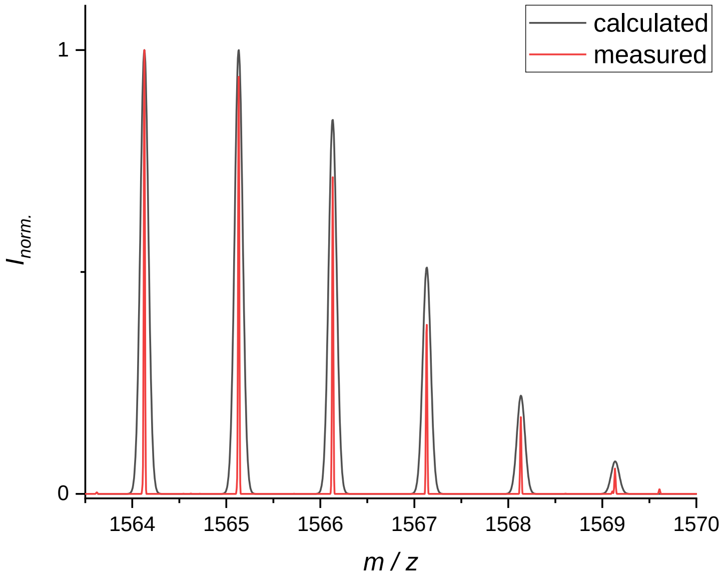

Supplementary Figure 11: High resolution ESI-MS measurement of T1. The observed isotopic pattern is compared with the calculated isotopic pattern from mMass (black).

```
.csv, maximum is 1.000000 found for mass 1564.124010
matching mass 1564.12401
cutoff 0.50000: 0 solutions (12 peaks)
cutoff 0.25000: 0 solutions (35 peaks)
cutoff 0.12500: 0 solutions (74 peaks)
cutoff 0.06250: 0 solutions (144 peaks)
cutoff 0.03125: 0 solutions (229 peaks)
cutoff 0.01562: 0 solutions (326 peaks)
cutoff 0.00781: 1 solutions (467 peaks)
1564.12401 ≈ 121.005630 + 311.246050 + 325.261700 + 283.214750 + 409.355600 + 91.054780 (sides 2-Ethylbutanal, Heptanal,
 Isobutyraldehyde, Tridecanal; error -22.98550)
Press ENTER to quit ...
```

Supplementary Figure 12: Screenshot of the automated read-out of T1, sodium trifluoroacetate was used as additive during the measurement.

1.3.3.2 Synthesis of hexamer H1

Deprotection

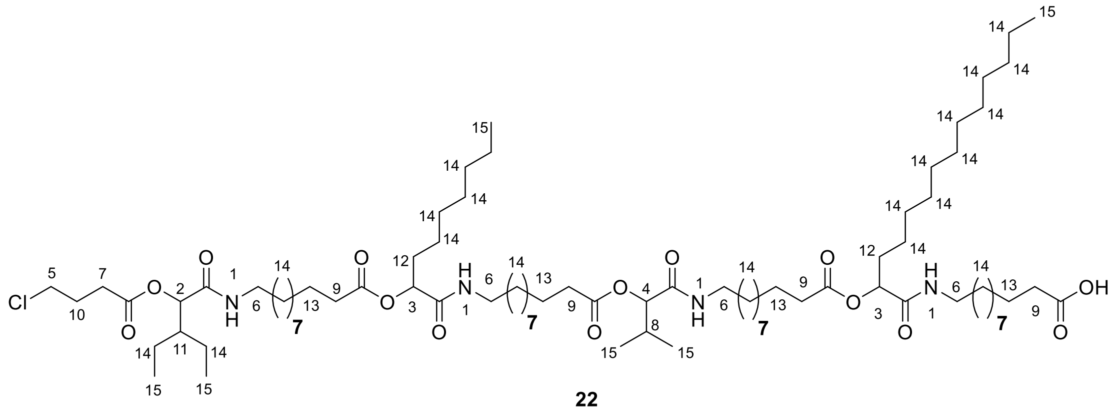

22

In a 25 mL round bottom flask, 874 mg of the passerini product **T1** (566 µmol, 1.00 eq.) was dissolved in 5.00 mL ethyl acetate and 5.00 mL THF. Subsequently, 174 mg (20 wt.-%) palladium on activated charcoal **16** were added to the solution. The resulting mixture was purged with hydrogen gas and stirred for one day at room temperature under hydrogen atmosphere (balloon). The crude reaction mixture was filtered over celite® and flushed with 50 mL dichloromethane. After evaporation of the solvents and drying under reduced pressure the corresponding acid **22** was obtained as a colorless solid. (806 mg, 555 µmol, 98.1%).

IR (ATR): ν / cm^{-1} = 3305.8 (vw), 2922.9 (vs), 2852.8 (s), 1739.6 (s), 1651.6 (s), 1536.8 (m), 1461.1 (m), 1371.7 (w), 1163.7 (s), 722.0 (w), 652.9 (w).

^{1}H NMR (400 MHz, $CDCl_3$): δ / ppm = 6.14 – 5.98 (m, 4 H, NH^{1}), 5.29 (d, J = 3.8 Hz, 1 H, CH^{2}), 5.19 – 5.11 (m, 2 H, CH^{3}), 5.04 (d, J = 4.5 Hz, 1 H, CH^{4}), 3.62 (t, J = 6.2 Hz, 2 H, CH_2^{5}), 3.33 – 3.16 (m, 8 H, CH_2^{6}), 2.68 – 2.57 (m, 2 H, CH_2^{7}), 2.50 – 2.23 (m, 9 H, CH^{8}, CH_2^{9}), 2.21 – 2.06 (m, 2 H, CH_2^{10}), 1.94 – 1.73 (m, 5 H, CH^{10}, CH_2^{12}), 1.73 – 1.56 (m, 8 H, CH_2^{13}), 1.57 – 1.17 (m, 88 H, CH_2^{14}), 0.98 – 0.82 (m, 18 H, CH_3^{15}).

^{13}C NMR (101 MHz, $CDCl_3$): δ / ppm = 177.00, 172.80, 172.63, 171.67, 170.11, 170.08, 169.72, 169.58, 78.08, 75.53, 74.09, 74.04, 44.09, 43.64, 39.39, 39.34, 39.30, 34.45, 34.41, 33.95, 32.04, 32.03, 32.01, 31.75, 31.22, 30.62, 29.79, 29.77, 29.75, 29.69, 29.67, 29.63, 29.60, 29.57, 29.50, 29.48, 29.44, 29.38, 29.35, 29.32, 29.25, 29.23, 29.10, 29.03, 27.48, 26.97, 26.94, 26.89, 25.12, 25.09, 24.91, 24.90, 24.84, 22.82, 22.66, 22.37, 22.01, 18.89, 17.11, 14.25, 14.17, 11.71, 11.70.

ESI-MS [m/z]: [M + H]$^{+}$ calculated for $^{12}C_{82}{}^{1}H_{151}{}^{16}O_{14}{}^{14}N_4{}^{35}Cl$, 1452.0988; found, 1452.0990, Δ = 0.2 mmu.

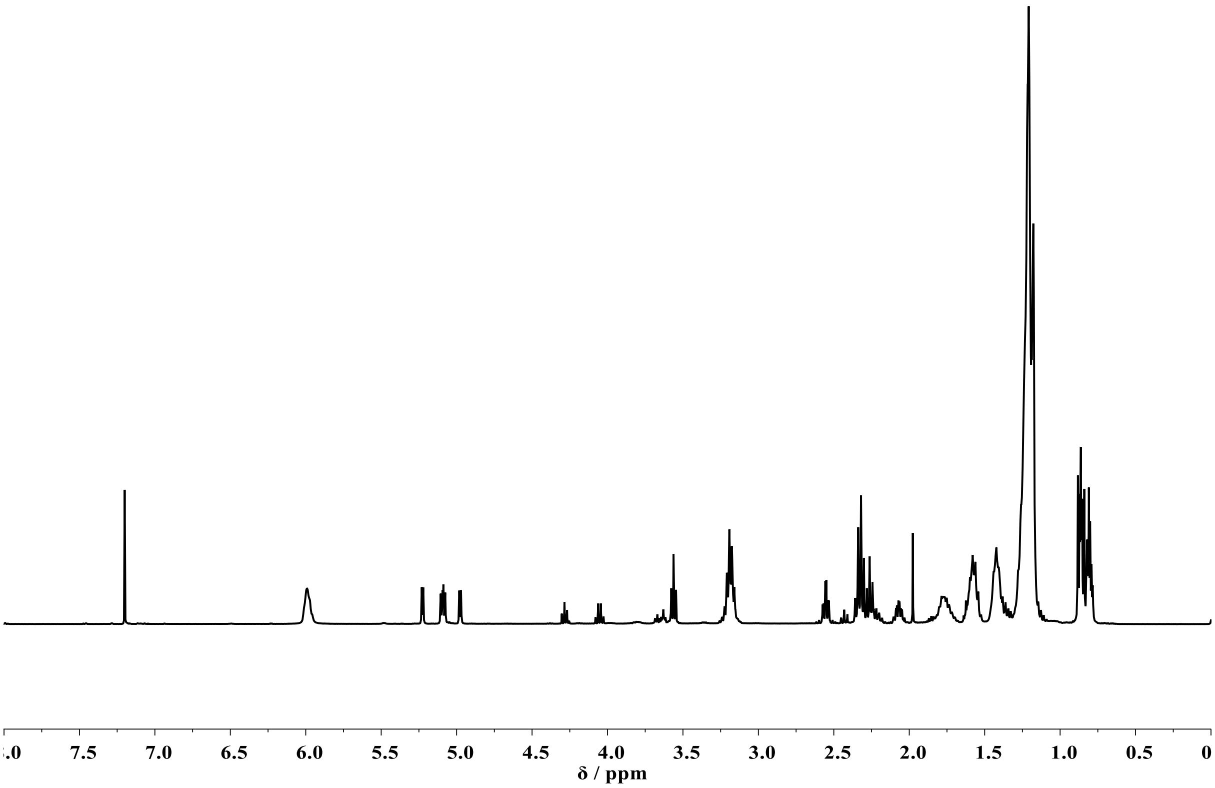

Supplementary Figure 13: ^{1}H-NMR of compound 22 measured in $CDCl_3$.

Passerini reaction

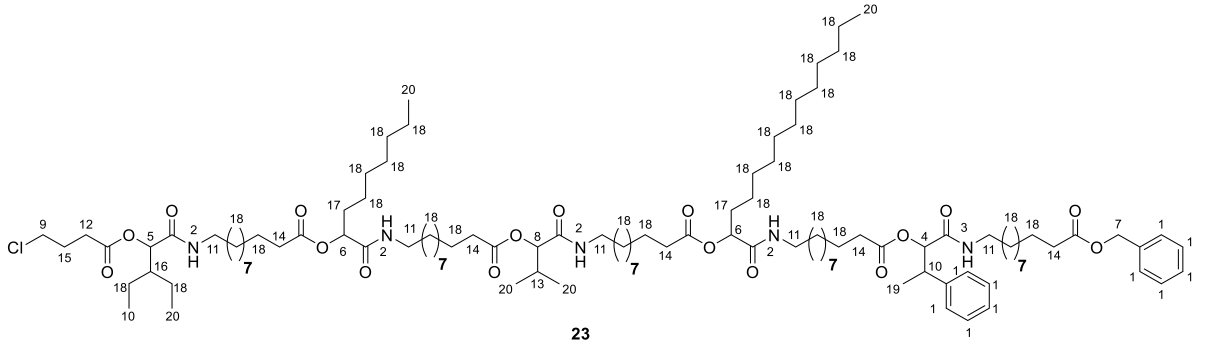

23

In a 50 mL round bottom flask 752 mg **22** (518 µmol, 1.00 eq.) was stirred in 2.00 mL DCM. Subsequently, 104 µL 2-phenylpropionaldehyde **14e** (104 mg, 776 µmol, 1.50 eq.) and 234 mg of the monomer **M1** (776 µmol, 1.50 eq.) were added. The resulting reaction mixture was stirred at room temperature for 2 days. Afterwards, the crude mixture was dried under reduced pressure. The residue was adsorbed onto celite® and purified *via* column chromatography on silica gel eluting with a gradual solvent mixture of ethyl acetate and cyclohexane (4:1 → 1:1) to yield the passerini product **23** as a yellow highly viscous oil (911 mg, 482 µmol, 93.2%).

R_f: 0.41 in cyclohexane / ethyl acetate (3:2).

IR (ATR): ν / cm^{-1} = 3306.0 (vw), 3087.6 (vw), 2923.2 (vs), 2853.0 (s), 2314.0 (vw), 2078.9 (vw), 1948.6 (vw), 1738.7 (vw), 1652.5 (vs), 1534.6 (s), 1456.2 (m), 1373.7 (w), 1231.6 (m), 1161.3 (s), 1107.7 (m), 1005.2 (w), 722.3 (w), 698.6 (m), 653.3 (w), 537.7 (vw), 429.7 (vw), 400.4 (vw).

^{1}H NMR (400 MHz, $CDCl_3$): δ / ppm = 7.44 – 7.16 (m, 10 H, $CH_{Ar}{}^{1}$), 6.12 – 5.93 (m, 4 H, NH^{2}), 5.67 (t, J = 5.9 Hz, 0.5 H, NH^{3a}), 5.64 – 5.58 (m, 0.5 H, NH^{3b}), 5.31 (d, J = 5.4 Hz, 0.5 H, CH^{4a}), 5.29 (d, J = 3.8 Hz, 1 H, CH^{5}), 5.21 (d, J = 5.4 Hz, 0.5 H, CH^{4b}), 5.17 – 5.12 (m, 2 H, CH^{6}), 5.10 (s, 2 H, $CH_2{}^{7}$), 5.04 (d, J = 4.4 Hz, 1 H, CH^{8}), 3.75 – 3.53 (m, 1 H, CH^{9}), 3.50 – 3.39 (m, 10 H, $CH_2{}^{10}$), 3.34 – 2.95 (m, 2 H, $CH_2{}^{11}$), 2.69 – 2.55 (m, 2 H, $CH_2{}^{12}$), 2.52 – 2.22 (m, 11 H, CH^{13}, $CH_2{}^{14}$), 2.20 – 2.06 (m, 2 H, $CH_2{}^{15}$), 1.96 – 1.72 (m, 5 H, CH^{16}, $CH_2{}^{17}$), 1.72 – 1.04 (m, 115 H, $CH_2{}^{18}$, $CH_3{}^{19}$), 0.97 – 0.82 (m, 18 H, $CH_3{}^{20}$).

^{13}C NMR (101 MHz, $CDCl_3$): δ / ppm = 173.79, 172.69, 172.60, 172.43, 171.63, 169.98, 169.61, 169.38, 168.82, 168.61, 141.73, 141.20, 136.26, 128.66, 128.52, 128.31, 128.30, 128.29, 127.97, 127.12, 127.05, 78.06, 77.86, 75.54, 74.06, 66.19, 44.08, 43.66, 41.58, 41.30, 39.36, 39.30, 39.28, 39.21, 34.45, 34.41, 34.35, 34.30, 32.04, 31.75, 31.22, 30.64, 29.79, 29.77, 29.74, 29.70, 29.67, 29.65, 29.58, 29.52, 29.51, 29.47, 29.38, 29.34, 29.33, 29.32, 29.24, 29.18, 29.16, 29.03, 27.48, 26.96, 29.65, 26.87, 26.80, 25.13, 25.09, 25.07, 24.98, 24.91, 24.84, 22.81, 22.65, 22.39, 22.03, 18.91, 17.63, 17.10, 15.34, 14.25, 14.17, 11.72, 11.71.

ESI-MS [m/z]: $[M+H]^+$ calculated for $^{12}C_{110}{}^{1}H_{188}{}^{16}O_{17}{}^{14}N_5{}^{35}Cl$: 1887.3762; found 1887.3793; Δ = 3.1 mmu.

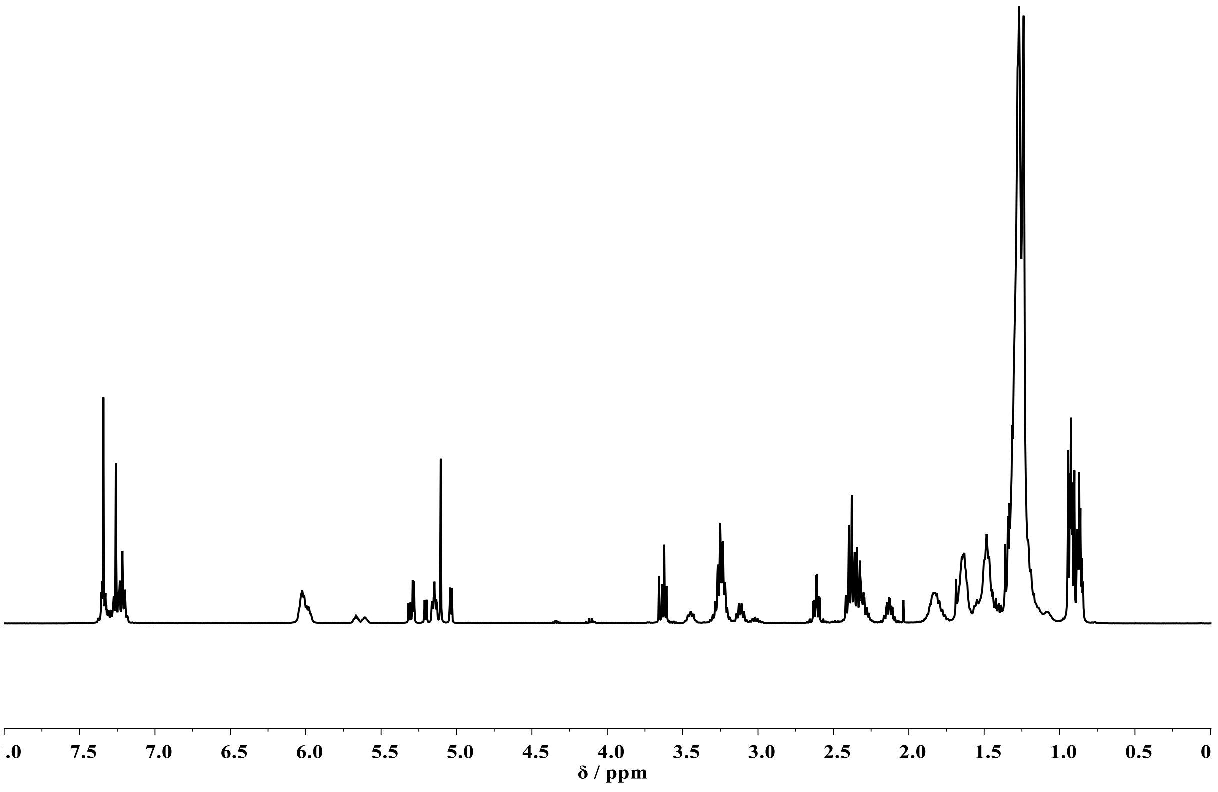

Supplementary Figure 14: ^{1}H-NMR of compound 23 measured in $CDCl_3$.

Deprotection

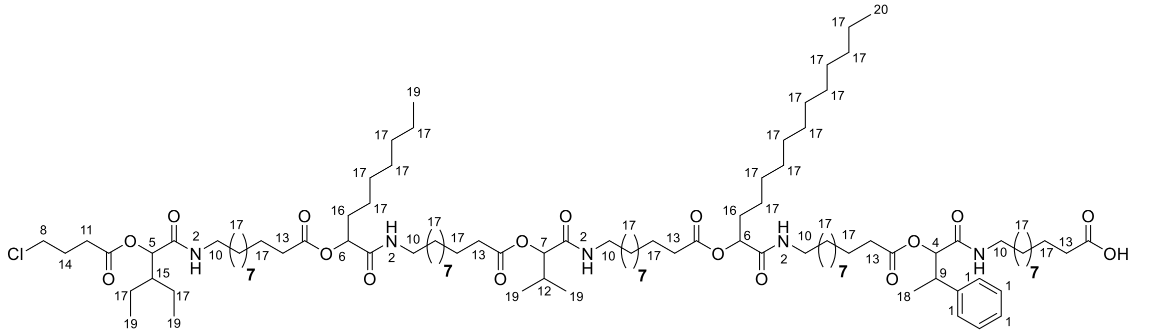

24

In a 50 mL round bottom flask, 718 mg of **24** (414 µmol, 1.00 eq.) were dissolved in 3.00 mL ethyl acetate and 3.00 mL THF. Afterwards, 156 mg (20 wt%) palladium on activated charcoal **16** were added. Subsequently, the mixture was purged with hydrogen (3 balloons) and stirred under hydrogen atmosphere overnight. The heterogeneous catalyst was filtered off and the solvent was evaporated under reduced pressure. The product **24** was obtained as a high viscous oil in a yield of 99.3% (739 mg, 411 µmol).

IR (ATR): ν / cm^{-1} = 3305.9 (vw), 3085.2 (vw), 2923.4 (vs), 2853.1 (s), 2075.0 (vw), 1739.2 (s), 1460.2 (m), 1373.3 (w), 1237.3 (m), 1162.2 (s), 1106.6 (m), 1021.1 (w), 923.9 (vw), 761.0 (vw), 721.2 (w), 699.8 (m), 653.9 (w), 538.2 (vw).

^{1}H NMR (400 MHz, $CDCl_3$): δ / ppm = 7.34 – 7.15 (m, 5 H, $CH_{Ar}{}^{1}$), 6.15 – 5.94 (m, 4 H, NH^{2}), 5.76 – 5.59 (m, 1 H, NH^{3}), 5.34 – 5.27 (m, 0.5 H, 1 H, CH^{4a}, CH^{5}), 5.22 (d, J = 5.5 Hz, 0.5 H, CH^{4b}), 5.18 – 5.12 (m, 2 H, CH^{6}), 5.04 (d, J = 4.5 Hz, 1 H, CH^{7}), 3.67 – 3.59 (m, 2 H, $CH_2{}^{8}$), 3.52 – 3.40 (m, 1 H, CH^{9}), 3.35 – 2.97 (m, 10 H, $CH_2{}^{10}$), 2.68 – 2.56 (m, 2 H, $CH_2{}^{11}$), 2.44 – 2.23 (m, 11 H, CH^{12}, $CH_2{}^{13}$), 2.18 – 2.08 (m, 2 H, $CH_2{}^{14}$), 1.93 – 1.73 (m, 5 H, CH^{15}, $CH_2{}^{16}$), 1.71 – 1.04 (m, 115 H, $CH_2{}^{17}$, $CH_3{}^{18}$), 0.99 – 0.82 (m, 18 H, $CH_3{}^{19}$).

^{13}C NMR (101 MHz, $CDCl_3$): δ / ppm = 176.41, 172.74, 172.71, 172.63, 171.66, 170.07, 169.69, 169.51, 168.71, 141.19, 128.53, 128.33, 127.98, 127.14, 127.07, 78.07, 77.87, 75.55, 74.08, 74.06, 44.09, 43.66, 41.56, 41.26, 39.39, 39.34, 39.33, 39.26, 39.17, 34.46, 34.42, 34.41, 34.36, 34.24, 33.87, 32.05, 31.76, 31.23, 30.65, 29.80, 29.78, 29.76, 29.72, 29.68, 29.65, 29.59, 29.49, 29.39, 29.36, 29.33, 29.27, 29.24, 29.20, 29.14, 29.08, 29.04, 27.49, 26.97, 26.78, 26.71, 25.14, 25.10, 24.92, 24.91, 24.85, 22.82, 22.67, 22.40, 22.03, 18.92, 17.65, 17.12, 1j5.31, 14.26, 14.18, 11.73, 11.71.

ESI-MS [m/z]: $[M + H]^{+}$ calculated for $^{12}C_{103}{}^{1}H_{182}{}^{16}O_{17}{}^{14}N_{5}{}^{35}Cl$, 1797.3292; found, 1797.3300, Δ = 1.8 mmu.

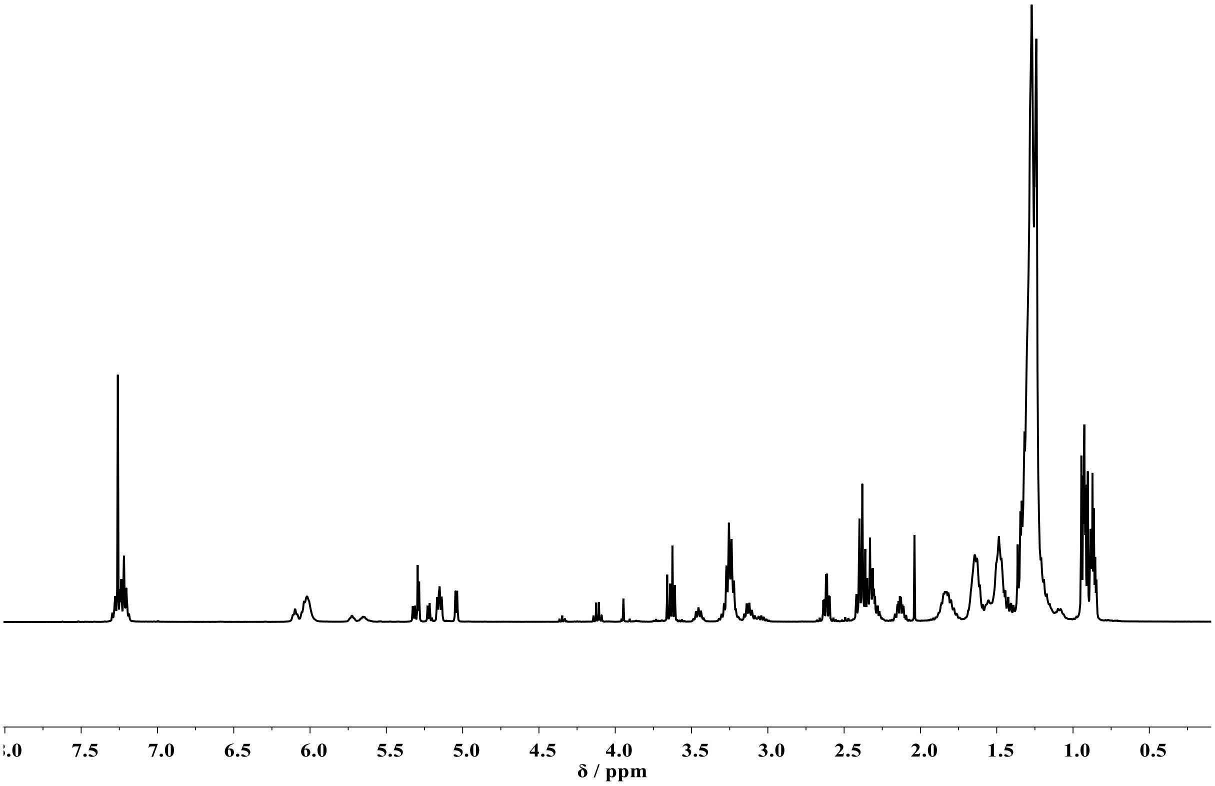

Supplementary Figure 15: ^{1}H-NMR of compound 24 measured in $CDCl_3$.

Passerini reaction

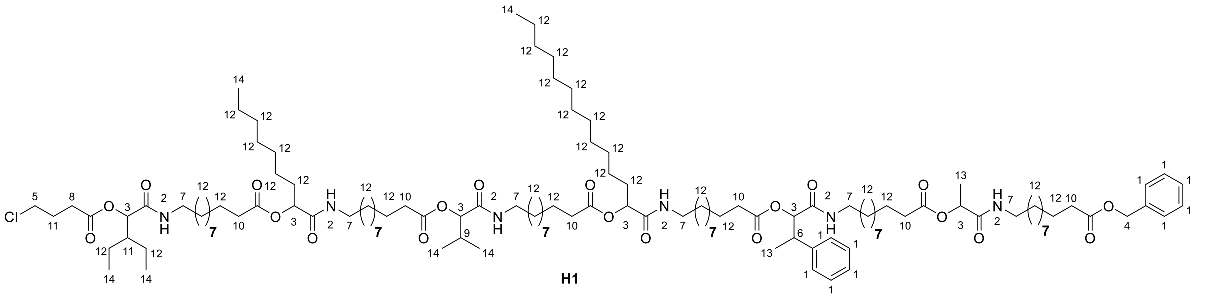

H1

In a 25 mL round bottom flask, 641 mg **24** (356 µmol, 1.00 eq.) was stirred in 3.00 mL DCM. Subsequently, 59.8 µL acetaldehyde **14f** (47.1 mg, 1.07 mmol, 3.00 eq.) and 161 mg of the monomer **M1** (535 µmol, 1.50 eq.) were added. The resulting reaction mixture was stirred at room temperature for 3 days. Afterwards, the crude mixture was dried under reduced pressure. The residue was adsorbed onto celite® and purified *via* column chromatography on silica gel eluting with a gradual solvent mixture of ethyl acetate and cyclohexane (4:1 → 1:2) to yield the passerini product **H1** as a pale highly viscous oil. (433.1 mg, 626 µmol, 56.7%).

R_f = 0.39 in cyclohexane / ethyl acetate (1:1).

IR (ATR): ν / cm^{-1} = 3304.9 (vw), 2323.4 (vs), 2852.9 (s), 1738.7 (vs), 1652.6 (vs), 1535.3 (s), 1456.1 (m), 1372.2 (w), 1232.5 (m), 1161.6 (s), 1105.5 (m), 722.1 (w), 698.9 (m).

^{1}H NMR (400 MHz, $CDCl_3$): δ / ppm = 7.46 – 7.13 (m, 10 H, CH_{Ar}^1), 6.20 – 5.92 (m, 5 H, NH^2), 5.75 – 5.59 (m, 1 H, NH^2), 5.34 – 5.08 (m, 7 H, CH^3, CH_2^4), 5.04 (d, *J* = 4.5 Hz, 1 H, CH^3), 3.69 – 3.58 (m, 2 H, CH_2^5), 3.49 – 3.39 (m, 1 H, CH^6), 3.34 – 2.97 (m, 12 H, CH_2^7), 2.66 – 2.57 (m, 2 H, CH_2^8), 2.45 – 2.23 (m, 13 H, CH^9, CH_2^{10}), 2.19 – 2.07 (m, 2 H, CH_2^{11}), 1.92 – 1.05 (m, 138 H, CH^{12}, CH_2^{12}, CH_3^{13}), 0.99 – 0.82 (m, 18 H, CH_3^{14}).

^{13}C NMR (101 MHz, $CDCl_3$): δ / ppm = 173.80, 172.71, 172.61, 172.46, 172.38, 171.64, 170.43, 170.00, 169.63, 169.40, 168.85, 168.65, 141.74, 141.23, 136.26, 128.67, 128.53, 128.31, 128.28, 127.97, 127.13, 127.05, 78.07, 77.87, 75.55, 74.07, 70.59, 66.19, 44.09, 43.66, 41.59, 41.30, 39.37, 39.32, 39.31, 39.22, 34.45, 34.42, 34.36, 34.30, 32.05, 31.75, 31.23, 30.65, 29.80, 29.75, 29.70, 29.69, 29.58, 29.53, 29.48, 29.35, 29.28, 29.26, 29.23, 29.21, 29.16, 29.04, 27.49, 26.97, 26.87, 26.81, 25.14, 25.10, 25.07, 25.01, 24.92, 24.85, 22.82, 22.66, 22.39, 22.03, 18.91, 18.10, 17.64, 17.11, 15.35, 14.25, 14.17, 11.73, 11.71.

ESI-MS [*m/z*]: $[M + H]^+$ calculated for $^{12}C_{124}{}^{1}H_{213}{}^{16}O_{20}{}^{14}N_6{}^{35}Cl$, 2142.5596; found, 2142.5607, Δ = 1.1 mmu.

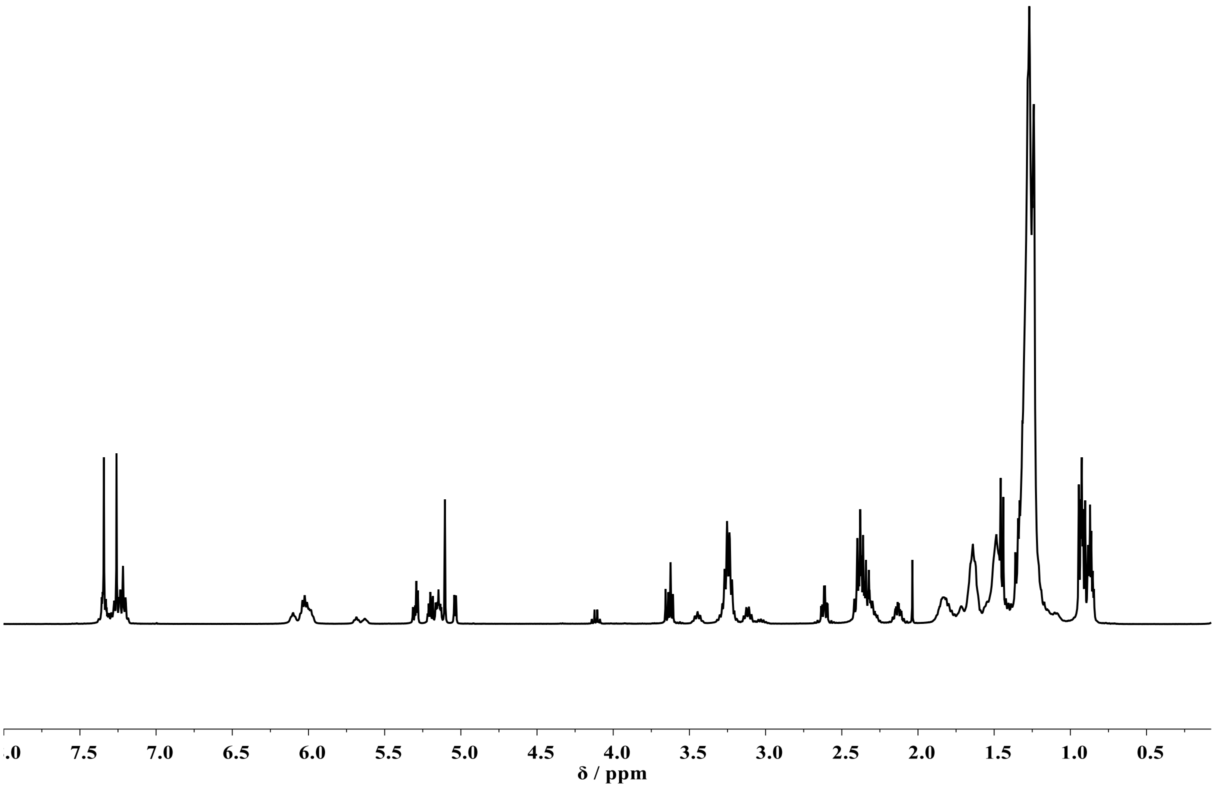

Supplementary Figure 16: ^{1}H-NMR of compound H1 measured in $CDCl_3$.

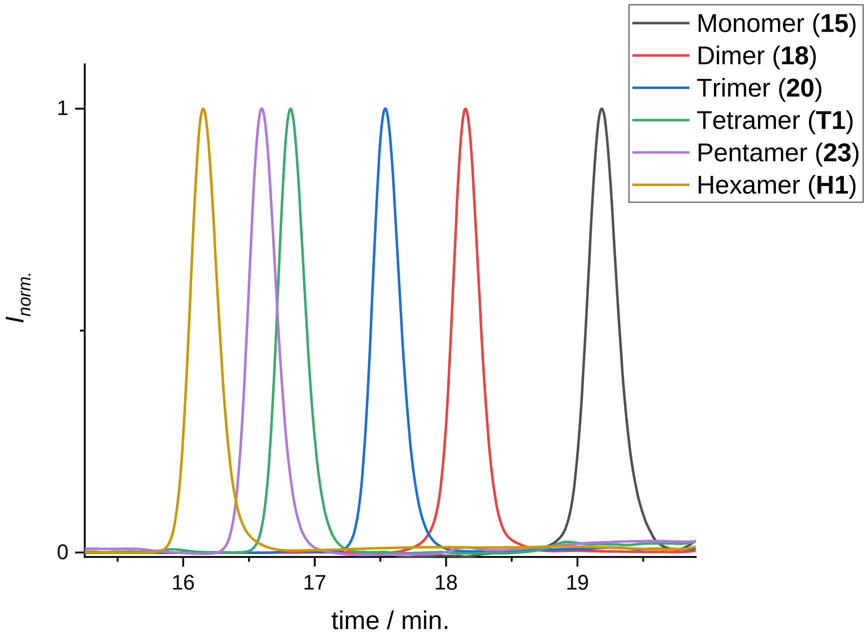

Supplementary Figure 17: SEC traces of the intermediates obtained after each P3CR in the synthesis of product H1.

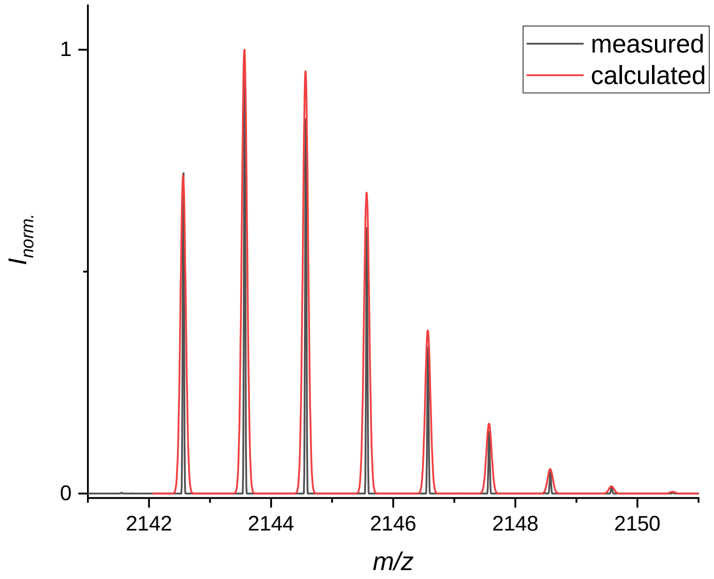

Supplementary Figure 18: High resolution ESI-MS measurement of H1. The isotopic observed isotopic pattern is compared with the calculated isotopic pattern obtained from mMass (red).

```
found 24339 values in C:\Users\Maxi\Desktop\MF 222 with 5 nce 19.CSV, maximum is 1.000000 found for mass 2142.562410
matching mass 2142.56241
cutoff 0.50000: 0 solutions (18 peaks)
cutoff 0.25000: 0 solutions (62 peaks)
cutoff 0.12500: 0 solutions (128 peaks)
cutoff 0.06250: 0 solutions (268 peaks)
cutoff 0.03125: 0 solutions (469 peaks)
cutoff 0.01562: 1 solutions (787 peaks)
2142.56241 ≈ 105.017020 + 311.246050 + 325.261700 + 283.214750 + 409.355600 + 345.230400 + 255.183450 + 107.049690 (sides
2-Ethylbutanal, Heptanal, Isobutyraldehyde, Tridecanal, 2-Phenylpropionaldehyde, Acetaldehyde; error -1.00375)
Press ENTER to quit ...
```

Supplementary Figure 19: Screenshot of the automated read-out of H1.

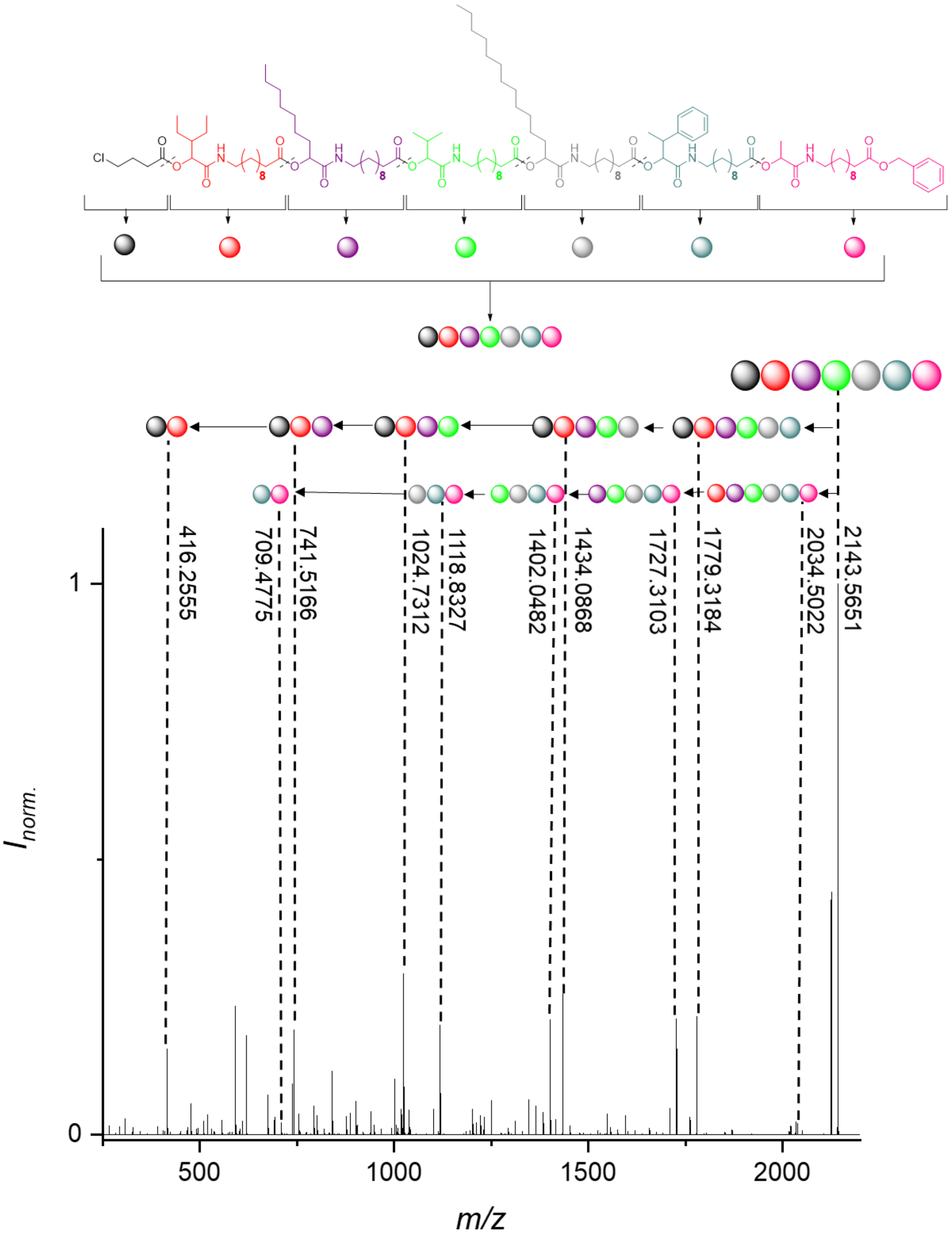

Supplementary Figure 20: Read-out of the sequence-defined hexamer H1. Read-out of the hexamer **H1** via tandem ESI-MS/MS with an NCE of 18. In the spectrum, the read-out from both ends of the oligomer using the fragmentation next to the carbonyl are shown.

1.3.3.3 Synthesis of tetramer T2

Passerini reaction

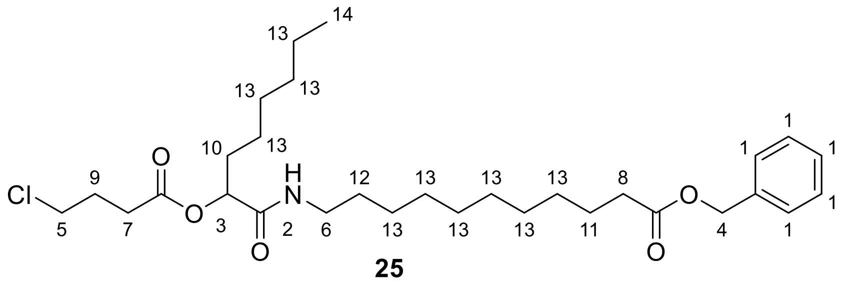

25

In a 50 mL round bottom flask, 74.5 mg 4-chlorobutyric acid **TAG3** (608 µmol, 1.00 eq.) was dissolved in 2.00 mL DCM and 130 µL heptanal **14b** (104 mg, 912 µmol, 1.50 eq.) and 275 mg of monomer **M1** (912 µmol, 1.50 eq.) were added. The mixture was stirred at room temperature for 3 days. Subsequently, the solvent was removed under reduced pressure. The crude product was purified by column chromatography (cyclohexane / ethyl acetate 8:1 →·6:1) to afford product **25** as a high viscous oil in a yield of 78.9% (258 mg, 480 µmol).

R_f = 0.32 in cyclohexane / ethyl acetate (3:1).

IR (ATR): ν / cm^{-1} = 3306.6 (vw), 2924.7 (s), 2854.1 (m), 1735.9 (vs), 1655.6 (s), 535.1 (m), 1455.4 (w), 1377.2 (w), 1143.9 (s), 734.2 (w), 696.9 (m), 648.9 (w), 401.0 (vw).

^{1}H NMR (400 MHz, $CDCl_3$): δ / ppm = 7.42 – 7.29 (m, 5 H, CH_{Ar}^{1}), 6.01 (t, J = 5.9 Hz, 1 H, NH^{2}), 5.18 – 5.14 (m, 1 H, CH^{3}), 5.11 (s, 2 H, CH_2^{4}), 3.62 (td, J = 6.3, 1.4 Hz, 2 H, CH_2^{5}), 3.36 – 3.17 (m, 2 H, CH_2^{6}), 2.61 (td, J = 7.1, 1.5 Hz, 2 H, CH_2^{7}), 2.35 (t, J = 7.5 Hz, 2 H, CH_2^{8}), 2.18 – 2.06 (m, 2 H, CH_2^{9}), 1.93 – 1.74 (m, 2 H, CH_2^{10}), 1.69 – 1.59 (m, 2 H, CH_2^{11}), 1.54 – 1.44 (m, 2 H, CH_2^{12}), 1.40 – 1.13 (m, 20 H, CH_2^{13}), 0.91 – 0.83 (m, 3 H, CH_3^{14}).

^{13}C NMR (101 MHz, $CDCl_3$): δ / ppm = 173.81, 171.58, 169.74, 136.25, 128.66, 128.28, 74.47; 66.20, 44.07, 39.37, 34.45, 32.05, 31.73, 31.25, 29.65, 29.56, 29.47, 29.33, 29.23, 29.03, 27.50, 26.95, 25.07, 24.86, 22.66, 14.17.

ESI-MS [m/z]: $[M + H]^+$ calculated for $^{12}C_{30}{}^{1}H_{48}{}^{16}O_5{}^{14}N^{35}Cl$, 538.3294; found, 538.3282, Δ = 1.2 mmu.

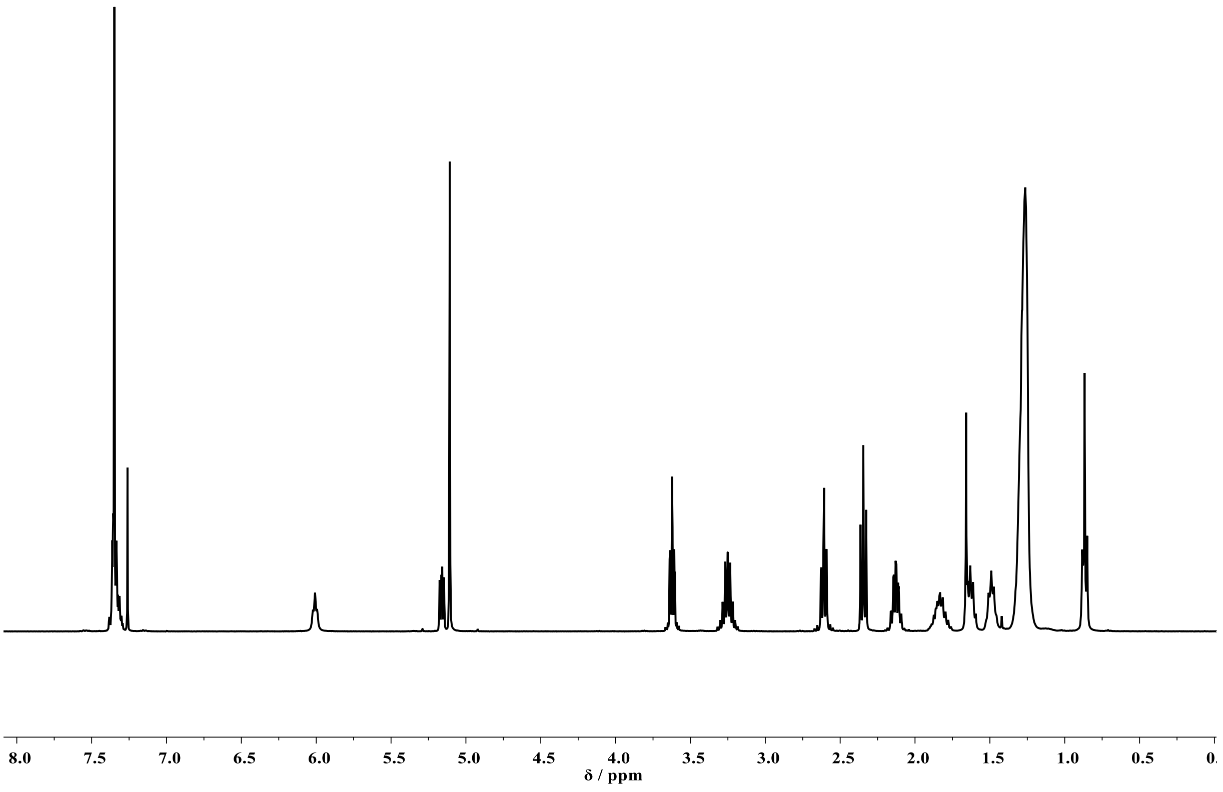

Supplementary Figure 21: ^{1}H-NMR of compound 25 measured in $CDCl_3$.

Deprotection

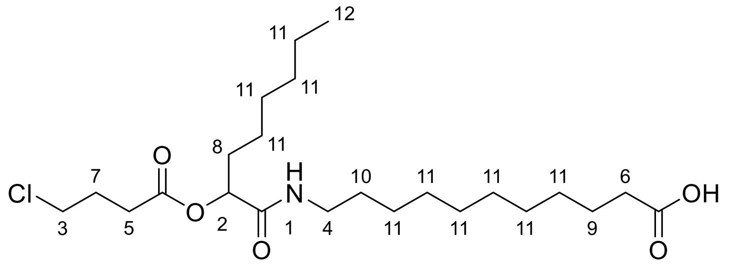

26

In a 50 mL round bottom flask, 209 mg of **25** (389 µmol, 1.00 eq.) were dissolved in 2.00 mL ethyl acetate and 2.00 mL THF. Afterwards, 41.8 mg (20 wt%) palladium on activated charcoal **16** were added. Subsequently, the mixture was purged with hydrogen (3 balloons) and stirred under hydrogen atmosphere overnight. The heterogeneous catalyst was filtered off and the solvent was evaporated under reduced pressure. The product **26** was obtained as a pale highly viscous oil in a yield of 99.0% (172 mg, 385 µmol).

IR (ATR): ν / cm^{-1} = 3296.9 (w), 2918.7 (vs), 2850.5 (s), 1731.7 (vs), 1694.2 (vs), 1651.8 (vs), 1557.9 (s), 1468.6 (m), 1430.0 (m), 1378.9 (m), 1330.2 (m), 1277.1 (s), 1219.4 (vs), 1173.2 (vs), 1082.2 (m), 925.5 (m), 794.1 (w), 722.8 (m), 683.4 (m), 650.5 (m), 473.8 (vw), 437.5 (w), 388.7 (w).

^{1}H NMR (400 MHz, $CDCl_3$): δ / ppm = 6.11 – 6.01 (m, 1 H, NH^1), 5.20 – 5.11 (m, 1 H, CH^2), 3.62 (t, J = 6.1 Hz, 2 H, CH_2^3), 3.35 – 3.17 (m, 2 H, CH_2^4), 2.60 (t, J = 6.6 Hz, 2 H, CH_2^5), 2.36 – 2.28 (m, 2 H, CH_2^6), 2.19 – 2.06 (m, 2 H, CH_2^7), 1.92 – 1.75 (m, 2 H, CH_2^8), 1.67 – 1.56 (m, 2 H, CH_2^9), 1.56 – 1.43 (m, 2 H, CH_2^{10}), 1.38 – 1.12 (m, 20 H, CH_2^{11}), 0.92 – 0.81 (m, 3 H, CH_3^{12}).

^{13}C NMR (101 MHz, $CDCl_3$): δ / ppm = 179.09, 171.64, 169.91, 74.47, 44.05, 39.41, 34.13, 32.03, 31.72, 31.26, 29.59, 29.48, 29.37, 29.25, 29.11, 29.02, 27.51, 26.91, 24.85, 24.83, 22.65, 14.15.

ESI-MS [m/z]: $[M + Na]^+$ calculated for $^{12}C_{23}{}^{1}H_{42}{}^{16}O_5{}^{14}N^{35}Cl$, 470.2644; found, 470.2639, Δ = 0.5 mmu.

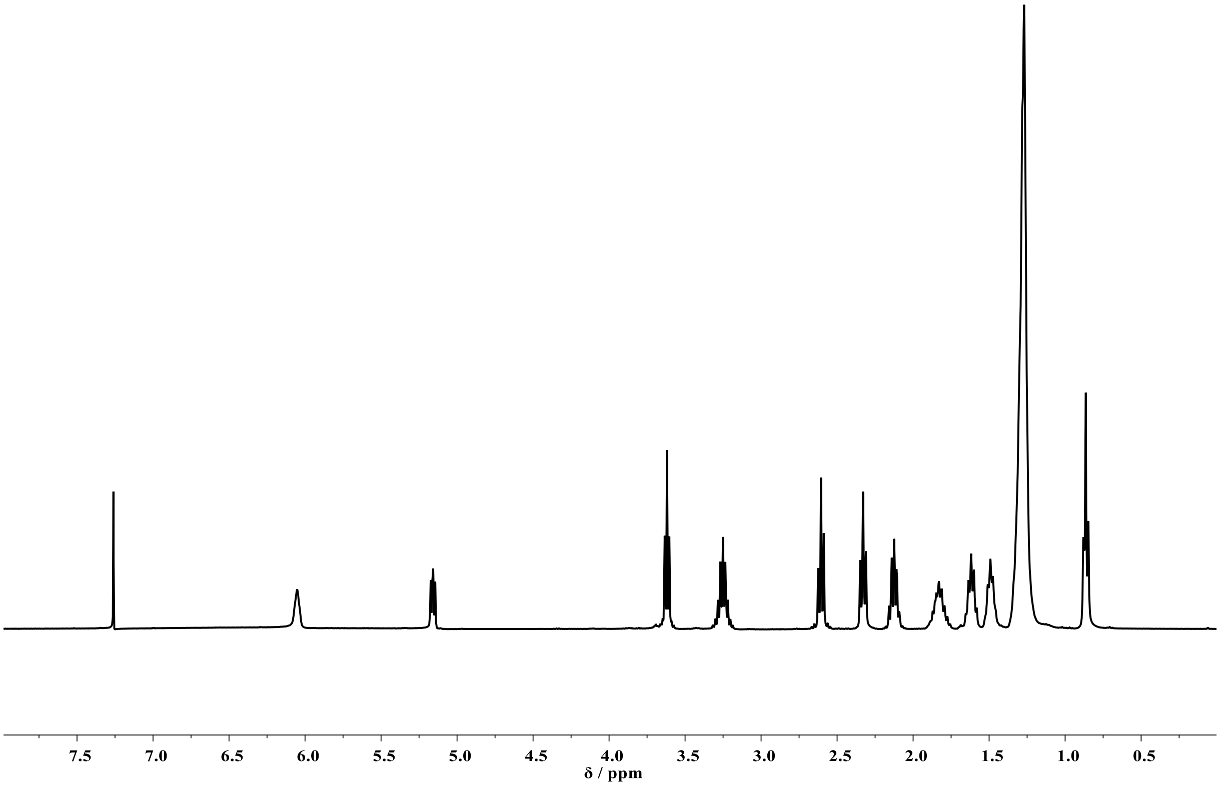

Supplementary Figure 22: ^{1}H-NMR of compound 26 measured in $CDCl_3$.

Passerini reaction

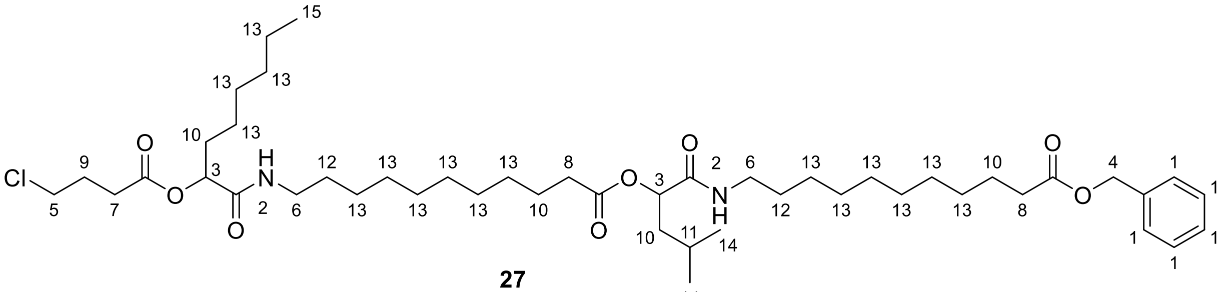

27

In a 50 mL round bottom flask, 172 mg **26** (384 µmol, 1.00 eq.) was dissolved in 2.00 mL DCM and 62.1 µL 3-methylbutanal **14g** (49.7 mg, 577 µmol, 1.50 eq.) and 174 mg of monomer **M1** (577 µmol,

1.50 eq.) were added. The mixture was stirred at room temperature for 3 days. Subsequently, the solvent was removed under reduced pressure. The crude product was purified by column chromatography (cyclohexane / ethyl acetate 5:1 → 2:1) to afford product **27** as a high viscous oil in a yield of 70.6% (227 mg, 271 µmol).

R_f = 0.39 in cyclohexane / ethyl acetate (2:1).

IR (ATR): ν / cm^{-1} = 304.3 (vw), 2924.8 (s), 2854.1 (m), 1737.4 (vs), 1654.9 (vs), 1536.0 (m), 1456.3 (m), 1371.1 (w), 1164.8 (s), 1061.8 (w), 732.8 (w), 697.0 (m).

^{1}H NMR (400 MHz, $CDCl_3$): δ / ppm = 7.37 – 7.21 (m, 5 H, CH_{Ar}^{1}), 5.98 – 5.91 (m, 1 H, NH^{2}), 5.90 – 5.81 (m, 1 H,NH^{2}), 5.16 – 5.06 (m, 2 H, CH^{3}), 5.04 (s, 2 H, CH_2^{4}), 3.56 (t, J = 5.7 Hz, 2 H, CH_2^{5}), 3.26 – 3.11 (m, 4 H, CH_2^{6}), 2.58 – 2.49 (m, 2 H, CH_2^{7}), 2.35 – 2.24 (m, 4 H, CH_2^{8}), 2.11 – 2.00 (m, 2 H, CH_2^{9}), 1.85 – 1.52 (m, 9 H, CH^{10}, CH_2^{11}), 1.47 – 1.36 (m, 4 H, CH_2^{12}), 1.31 – 1.12 (m, 32 H, CH_2^{13}), 0.86 (t, J = 5.6 Hz, 6 H, CH_3^{14}), 0.83 – 0.78 (m, 3 H, CH_3^{15}).

^{13}C NMR (101 MHz, $CDCl_3$): δ / ppm = 173.84, 172.78, 171.61, 170.34, 169.78, 128.69, 128.31, 74.49, 72.80, 66.22, 44.09, 40.99, 39.38, 34.47, 34.45, 32.07, 31.74, 31.26, 29.67, 29.58, 29.49, 29.35, 29.25, 29.24, 29.04, 27.51, 26.96, 26.95, 25.08, 24.88, 24.70, 23.28, 22.67, 21.95, 14.18.

ESI-MS [m/z]: $[M + H]^+$ calculated for $^{12}C_{47}{}^{1}H_{79}{}^{6}O_8{}^{14}N_2{}^{35}Cl$, 835.5598; found, 835.5588, Δ = 1.0 mmu.

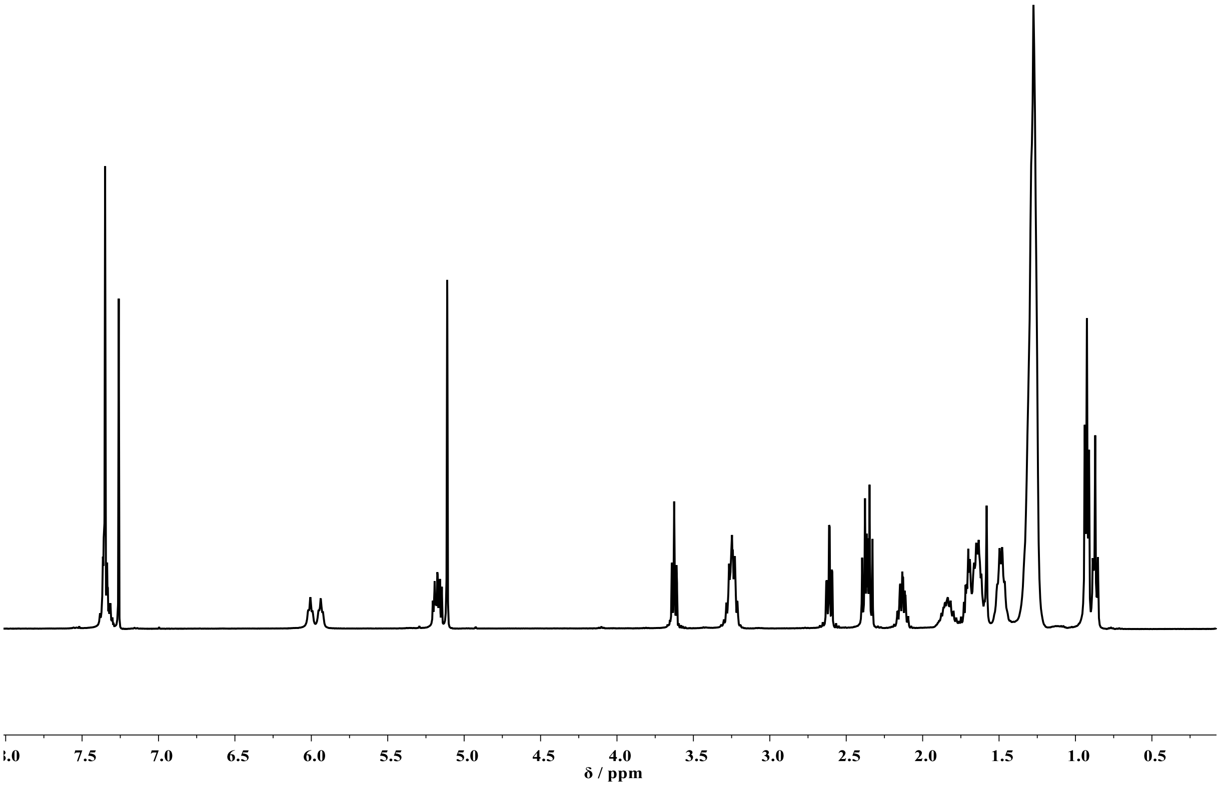

Supplementary Figure 23: ^{1}H-NMR of compound 27 measured in $CDCl_3$.

Deprotection

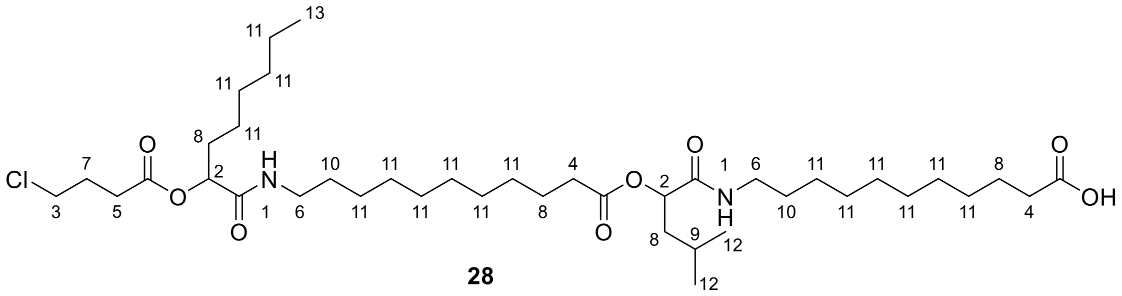

28

In a 50 mL round bottom flask, 176 mg of **27** (211 µmol, 1.00 eq.) were dissolved in 2.00 mL of ethyl acetate and 2.00 mL THF. Afterwards, 35.2 mg (20 wt%) palladium on activated charcoal **16** were added. Subsequently, the mixture was purged with hydrogen (3 balloons) and stirred under hydrogen atmosphere overnight. The heterogeneous catalyst was filtered off and the solvent was evaporated under reduced pressure. The product **28** was obtained as a pale highly viscous oil in a yield of 97.6% (153 mg, 206 mmol).

IR (ATR): ν / cm^{-1} = 3306.8 (vw), 2924.2 (s), 2853.7 (m), 1738.2 (s), 1651.7 (s), 1540.2 (m), 1463.1 (w), 1370.1 (w), 1143.5 (s), 1061.2 (w), 722.6 (w), 650.3 (w).

^{1}H NMR (400 MHz, $CDCl_3$): δ / ppm = 6.12 – 6.03 (m, 1 H, NH^{1}), 5.98 (t, J = 5.8 Hz, 1 H, NH^{1}), 5.16 – 5.07 (m, 2 H, CH^{2}), 3.62 – 3.52 (m, 2 H, CH_2^{3}), 3.24 – 3.13 (m, 4 H, CH_2^{4}), 2.59 – 2.50 (m, 2 H, CH_2^{5}), 2.36 – 2.22 (m, 4 H, CH_2^{6}), 2.11 – 2.01 (m, 2 H, CH_2^{7}), 1.85 – 1.69 (m, 2 H, CH_2^{8}), 1.68 – 1.50 (m, 7 H, CH^{9}, CH_2^{8}), 1.50 – 1.34 (m, 4 H, CH_2^{10}), 1.31 – 1.13 (m, 32 H, CH_2^{11}), 0.86 (t, J = 5.8 Hz, 6 H, CH_3^{12}), 0.83 – 0.77 (m, 3 H, CH_3^{13}).

^{13}C NMR (101 MHz, $CDCl_3$): δ / ppm = 178.33, 172.81, 171.67, 170.47, 169.97, 74.42, 72.75, 44.05, 40.92, 39.40, 39.35, 34.40, 34.11, 32.01, 31.70, 31.22, 29.60, 29.56, 29.53, 29.46, 29.45, 29.36, 29.31, 29.30, 29.23, 29.21, 29.10, 29.00, 27.47, 26.93, 26.86, 25.04, 24.86, 24.84, 24.65, 23.23, 22.63, 21.89, 14.15.

ESI-MS [*m/z*]: $[M + H]^+$ calculated for $^{12}C_{40}{}^{1}H_{73}{}^{6}O_8{}^{14}N_2{}^{35}Cl^{23}$, 745.5128; found, 745.5113, Δ = 1.5 mmu.

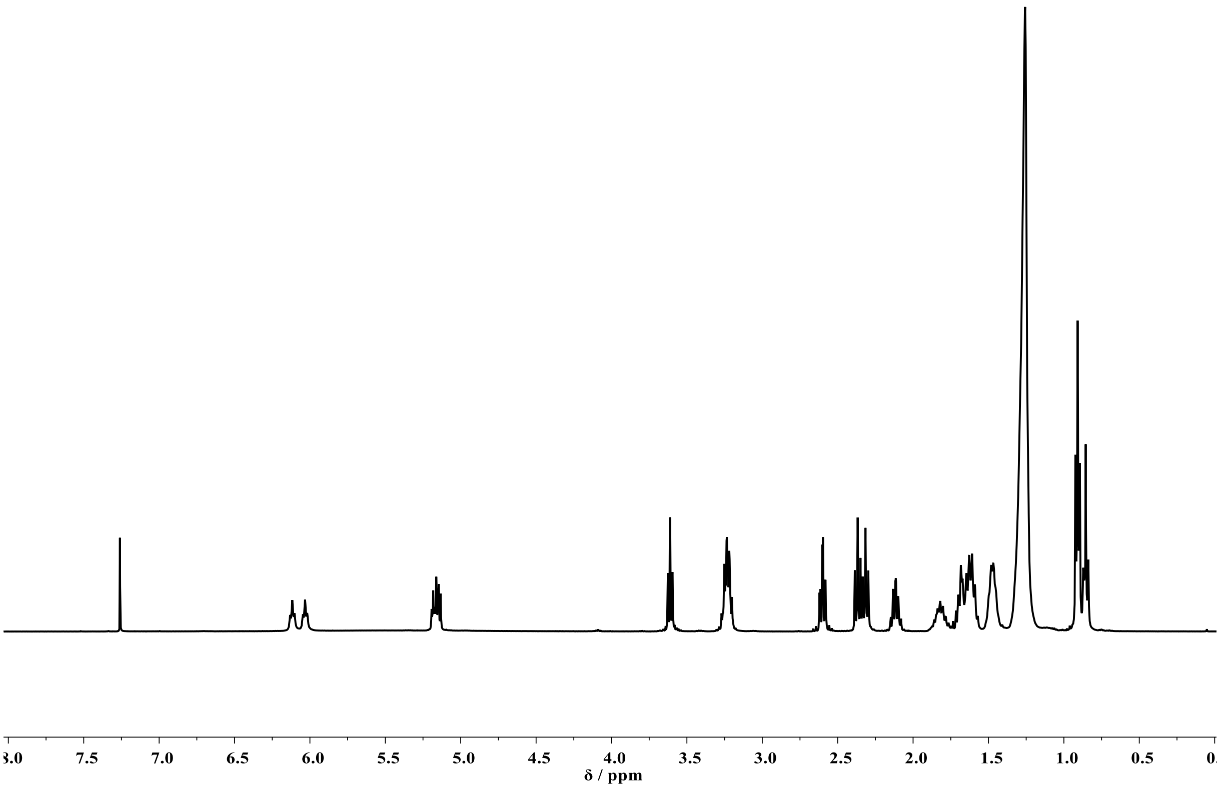

Supplementary Figure 24: ^{1}H-NMR of compound 28 measured in $CDCl_3$.

Passerini reaction

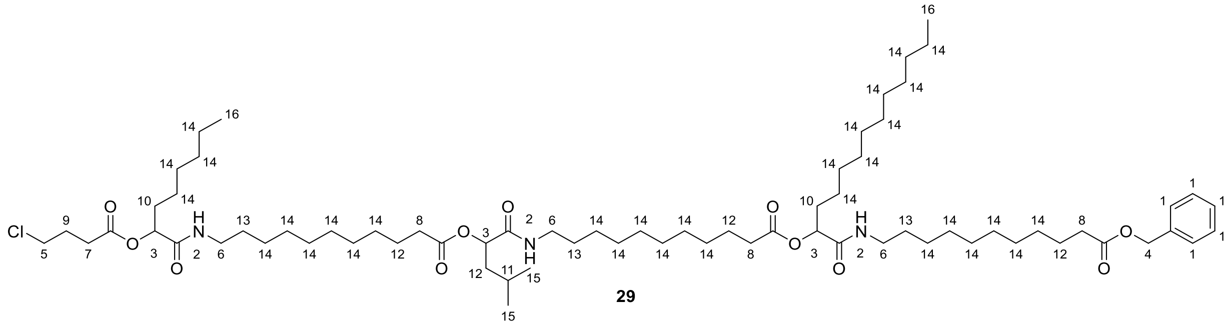

In a 50 mL round bottom flask, 91.2 mg **28** (122 µmol, 1.00 eq.) was dissolved in 2.00 mL DCM and 40.8 µL dodecanal **14h** (33.8 mg, 184 µmol, 1.50 eq.) and 55.3 mg of monomer **M1** (184 µmol, 1.50 eq.) were added. The mixture was stirred at room temperature for 3 days. Subsequently, the solvent was removed under reduced pressure. The crude product was purified by column chromatography (cyclohexane / ethyl acetate 8:1 →6:1) to afford product **29** as a high viscous oil in a yield of 82.8% (124 mg, 101 µmol).

R_f = 0.77 in cyclohexane / ethyl acetate (1:1).

IR (ATR): ν / cm^{-1} = 3294.1 (w), 2923.0 (s), 2852.9 (m), 1738.2 (s), 1654.5 (s), 1535.6 (m), 1457.0 (w), 1371.2 (w), 1163.3 (s), 723.5 (w), 697.0 (w).

^{1}H NMR (400 MHz, $CDCl_3$): δ / ppm = 7.36 – 7.23 (m, 5 H, CH_{Ar}^{1}), 6.04 – 5.88 (m, 3 H, NH^{2}), 5.16 – 5.05 (m, 3 H, CH^{3}), 5.04 (s, 2 H, CH_2^{4}), 3.62 – 3.53 (m, 2 H, CH_2^{5}), 3.27 – 3.12 (m, 6 H, CH_2^{6}), 2.59 – 2.50 (m, 2 H, CH_2^{7}), 2.34 – 2.24 (m, 6 H, CH_2^{8}), 2.12 – 2.01 (m, 2 H, CH_2^{9}), 1.84 – 1.67 (m, 4 H, CH_2^{10}), 1.64 – 1.51 (m, 9 H, CH^{11}, CH_2^{12}), 1.47 – 1.37 (m, 6 H, CH_2^{13}), 1.30 – 1.14 (m, 62 H, CH_2^{14}), 0.86 (t, J = 5.8 Hz, 6 H, CH_3^{15}), 0.83 – 0.77 (m, 6 H, CH_3^{16}).

^{13}C NMR (101 MHz, $CDCl_3$): δ / ppm = 173.82, 172.78, 172.61, 171.61, 170.36, 169.99, 169.78, 136.23, 128.66, 128.29, 128.28, 74.44, 74.06, 72.75, 66.19, 44.07, 40.97, 39.33, 39.32, 34.44, 32.03, 31.72, 31.22, 29.74, 29.66, 29.65, 29.57, 29.55, 29.47, 29.38, 29.34, 29.32, 29.23, 29.21, 29.02, 27.48, 26.95, 26.92, 25.08, 25.05, 25.04, 24.88, 24.86, 24.66, 23.26, 22.81, 22.65, 21.91, 14.25, 14.16.

ESI-MS [*m/z*]: $[M + H]^+$ calculated for $^{12}C_{71}{}^{1}H_{124}{}^{16}O_{11}{}^{14}N_{3}{}^{35}Cl$, 1230.8997; found, 1230.8976, Δ = 2.1 mmu.

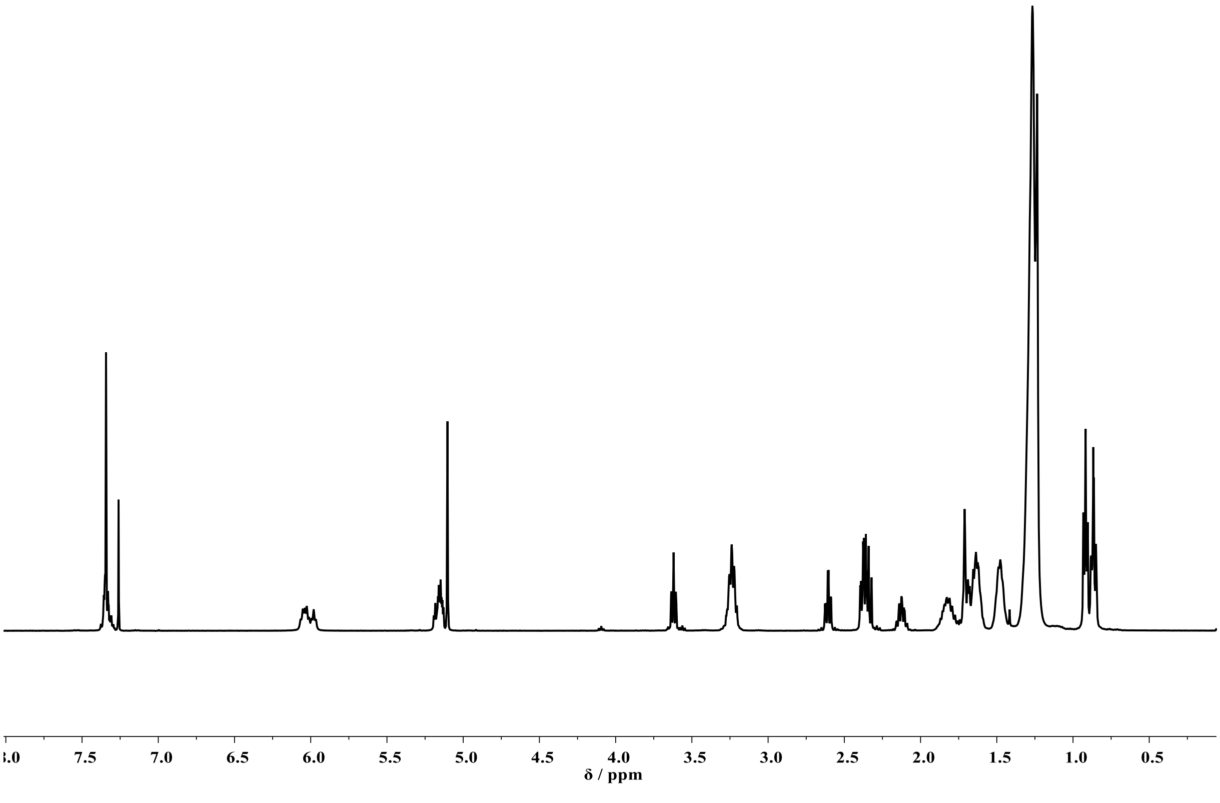

Supplementary Figure 25: ^{1}H-NMR of compound 29 measured in $CDCl_3$.

Deprotection

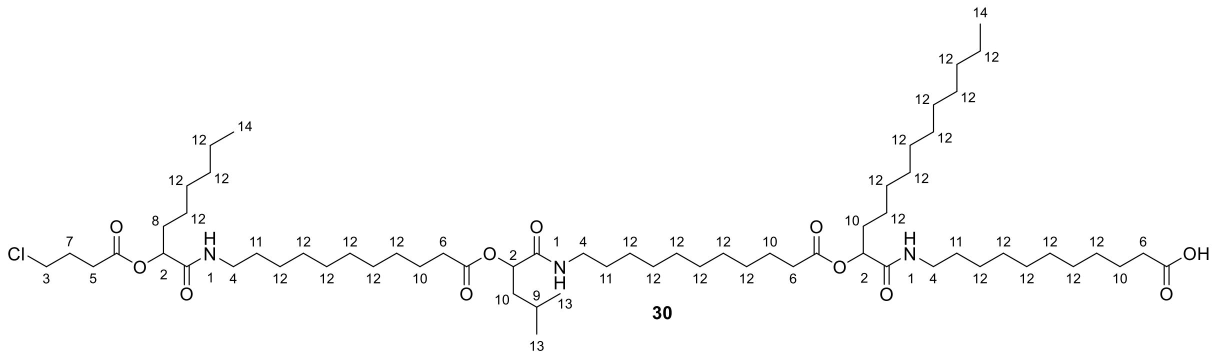

In a 50 mL round bottom flask, 75.3 mg of **29** (61.0 µmol, 1.00 eq.) were dissolved in 2.00 mL ethyl acetate and 2.00 mL THF. Afterwards, 15.6 mg (20 wt%) palladium on activated charcoal **16** were added. Subsequently, the mixture was purged with hydrogen (3 balloons) and stirred under hydrogen atmosphere overnight. The heterogeneous catalyst was filtered off and the solvent was evaporated under reduced pressure. The product **30** was obtained as a pale highly viscous oil in a yield of 89.2% (62.3 mg, 54.4 mmol).

IR (ATR): ν / cm^{-1} = 3292.6 (vw), 2822.8 (vs), 2852.9 (s), 1739.4 (s), 1652.5 (s), 1539.6 (m), 1463.5 (w), 1370.3 (w), 1165.3 (m), 722.0 (w).

^{1}H NMR (400 MHz, $CDCl_3$): δ / ppm = 6.10 – 5.95 (m, 3 H, NH^{1}), 5.18 – 5.01 (m, 3 H, CH^{2}), 3.61 – 3.49 (m, 2 H, CH_2^{3}), 3.25 – 3.12 (m, 6 H, CH_2^{4}), 2.60 – 2.50 (m, 2 H, CH^{5}), 2.36 – 2.20 (m, 6 H, CH_2^{6}), 2.13 – 2.00 (m, 2 H, CH_2^{7}), 1.85 – 1.67 (m, 4 H, CH_2^{8}), 1.67 – 1.50 (m, 9 H, CH^{9}, CH_2^{10}), 1.48 – 1.37 (m, 6 H, CH_2^{11}), 1.30 – 1.13 (m, 22 H, CH_2^{12}), 0.86 (t, *J* = 5.9 Hz, 6 H, CH_3^{13}), 0.84 – 0.77 (m, 6 H, CH_3^{14}).

^{13}C NMR (101 MHz, $CDCl_3$): δ / ppm = 177.57, 172.86, 172.62, 171.63, 170.50, 170.09, 169.90, 74.44, 74.08, 72.77, 44.04, 40.93, 39.40, 39.39, 39.31, 34.44, 34.40, 34.01, 32.02, 31.99, 31.71, 31.24, 29.73, 29.65, 29.61, 29.57, 29.55, 29.49, 29.45, 29.36, 29.30, 29.24, 29.20, 29.11, 29.00, 27.50, 26.92, 26.91, 26.89, 25.10, 25.02, 24.89, 24.87, 24.85, 24.66, 23.23, 22.79, 22.63, 21.91, 14.22, 14.14.

ESI-MS [*m/z*]: $[M + H]^+$ calculated for $^{12}C_{64}{}^{1}H_{118}{}^{16}O_{11}{}^{14}N_3{}^{35}Cl^{23}$, 1140.8528; found, 1140.8505, Δ = 2.3 mmu.

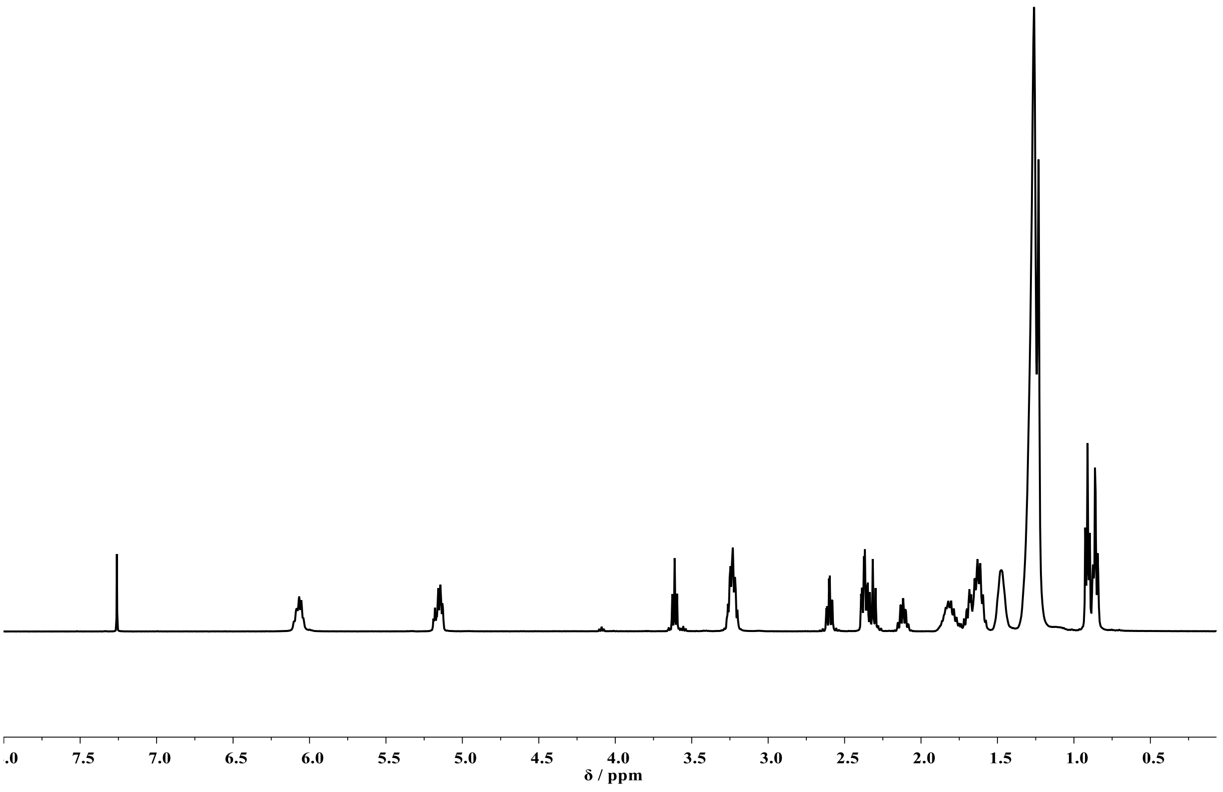

Supplementary Figure 26: ^{1}H-NMR of compound 30 measured in $CDCl_3$.

Passerini reaction

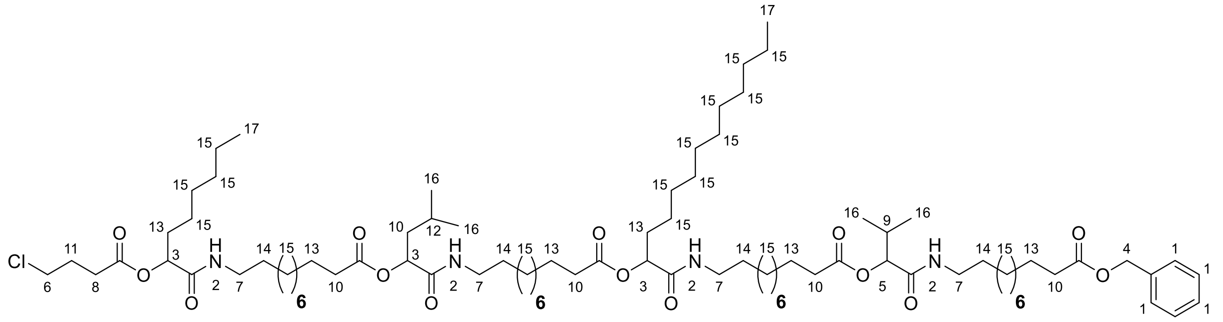

T2

In a 50 mL round bottom flask, 62.0 mg **30** (54.0 µmol, 1.00 eq.) was dissolved in 2.00 mL DCM and 8.00 µL isobutyraldehyde **14c** (5.90 mg, 81.0 µmol, 1.50 eq.) and 29.2 mg of monomer **M1** (81.0 µmol, 1.50 eq.) were added. The mixture was stirred at room temperature for 24 hours. Subsequently, the solvent was removed under reduced pressure. The crude product was purified by column chromatography (hexane / ethyl acetate 4:1 → 2:1) to afford product **T2** as a high viscous oil in a yield of 70.7% (58.2 mg, 38.2 µmol).

R_f = 0.53 in cyclohexane / ethyl acetate (2:1).

IR (ATR): ν / cm^{-1} = 3293.2541 (vw), 2919.9 (m), 2851.4 (w), 1736.3 (m), 1654.9 (s), 1556.3 (w), 1466.3 (w), 1374.3 (w), 1243.1 (w), 1207.6(w), 1176.0 (m), 1059.7 (w), 1000.8 (w), 879.3 (vw), 841.2 (vw), 721.1395 (w), 689.2593 (w), 430.7457 (vw).

^{1}H NMR (400 MHz, $CDCl_3$): δ / ppm = 7.35 – 7.23 (m, 5 H, CH_{Ar}^{1}), 6.07 – 5.89 (m, 4 H, NH^{2}), 5.16 – 5.07 (m, 3 H, CH^{3}), 5.04 (s, 2 H, CH_2^{4}), 4.98 (d, J = 4.4 Hz, 1 H, CH^{5}), 3.56 (t, J = 6.1 Hz, 2 H, CH_2^{6}), 3.27 – 3.09 (m, 8 H, CH_2^{7}), 2.60 – 2.50 (m, 2 H, CH_2^{8}), 2.38 – 2.18 (m, 9 H, CH^{9}, CH_2^{10}), 2.11 – 2.02 (m, 2 H, CH_2^{11}), 1.85 – 1.51 (m, 15 H, CH^{12}, CH_2^{13}), 1.48 – 1.37 (m, 8 H, CH_2^{14}), 1.32 – 1.10 (m, 74 H, CH_2^{15}), 0.90 – 0.83 (m, 12 H, CH_2^{16}), 0.83 – 0.78 (m, 6 H, CH_2^{17}).

^{13}C NMR (101 MHz, $CDCl_3$): δ / ppm = 173.81, 172.78, 172.69, 172.62, 171.61, 170.36, 170.00, 169.78, 169.39, 136.24, 128.65, 128.28, 128.27, 125.63, 78.05, 74.44, 74.06, 72.75, 66.18, 44.06, 40.97, 39.34, 39.28, 34.43, 34.40, 32.03, 31.72, 31.23, 30.64, 30.44, 29.74, 29.69, 29.68, 29.65, 29.57, 29.55, 29.48, 29.46, 29.38, 29.37, 29.35, 29.34, 29.33, 29.31, 29.25, 29.23, 29.22, 29.01, 27.49, 26.96, 26.92, 25.12, 25.07, 25.05, 25.04, 24.90, 24.86, 24.66, 23.25, 22.80, 22.64, 21.91, 18.90, 17.09, 14.24, 14.15.

ESI-MS [*m/z*]: [M + H]$^{+}$ calculated for $^{12}C_{87}{}^{1}H_{153}{}^{16}O_{14}{}^{14}N_{4}{}^{35}Cl$, 1514.1145; found, 1514.1137, Δ = 0.8 mmu.

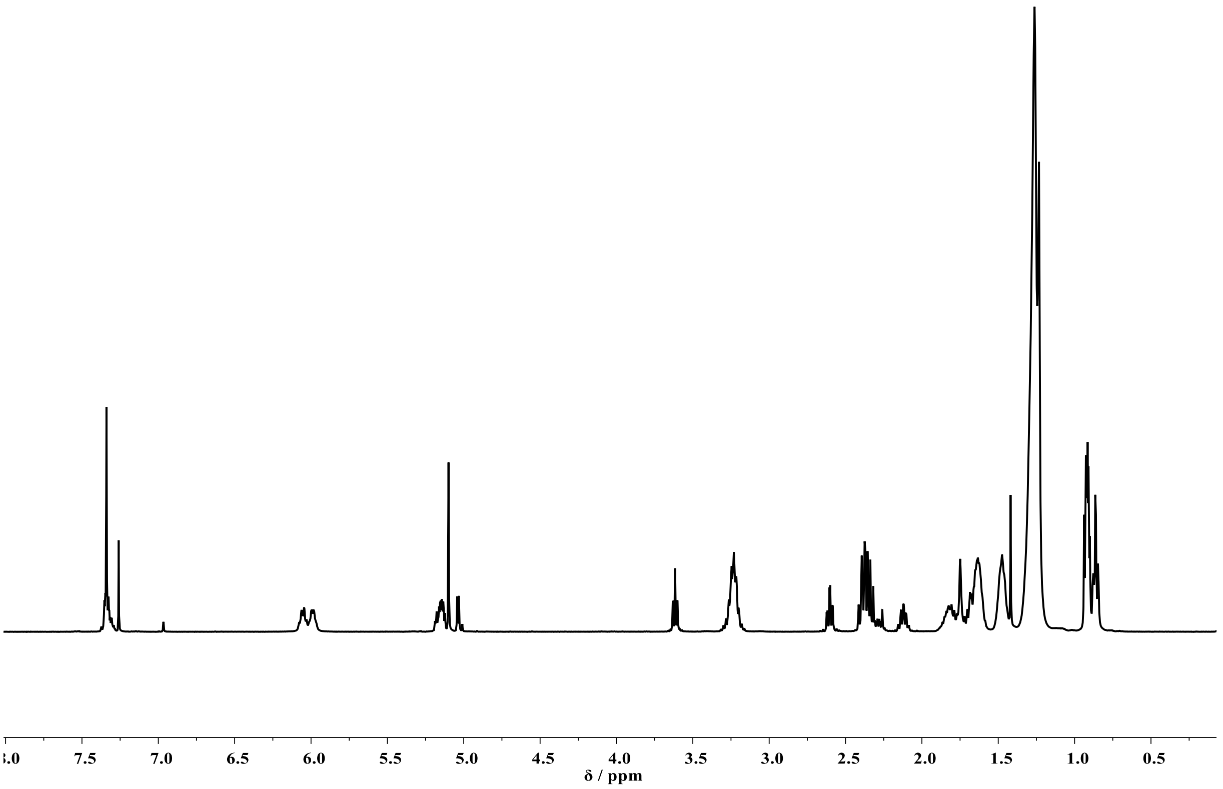

Supplementary Figure 27: ^{1}H-NMR of compound T2 measured in $CDCl_3$.

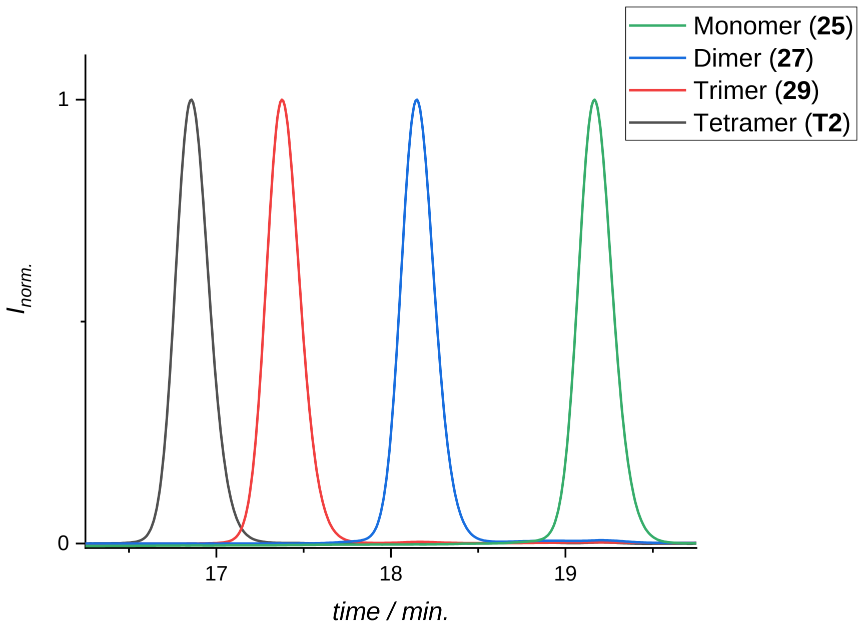

Supplementary Figure 28: SEC traces of the intermediates after each P3CR in the synthesis of product T2.

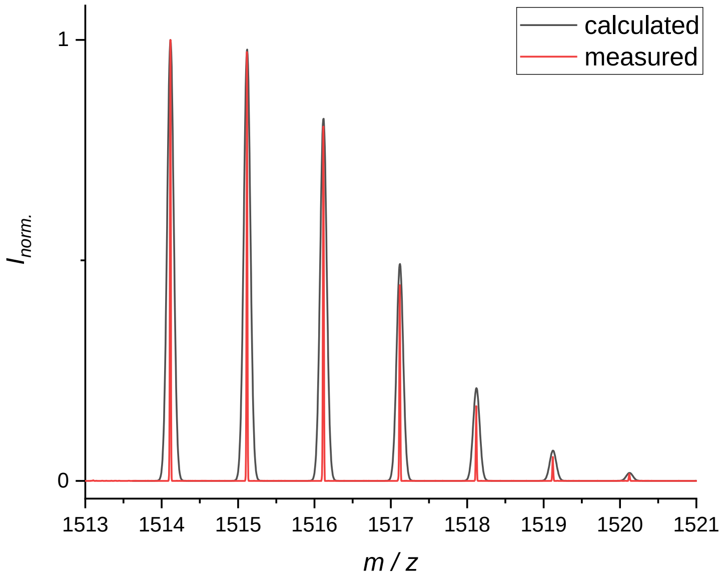

Supplementary Figure 29: High resolution ESI-MS measurement of T2. The observed isotopic pattern is compared with the calculated isotopic pattern obtained from mMass (black).

```
er.CSV, maximum is 100.000000 found for mass 1536.089620
matching mass 1536.08962
cutoff 0.50000: 0 solutions (22 peaks)
cutoff 0.25000: 0 solutions (75 peaks)
cutoff 0.12500: 0 solutions (140 peaks)
cutoff 0.06250: 1 solutions (239 peaks)
1536.08962 ≈ 121.005630 + 325.261700 + 297.230400 + 395.339950 + 283.214750 + 91.054780 (sides Heptanal, 3-Methylbutanal
, Dodecanal, Isobutyraldehyde; error -22.98241)
Press ENTER to quit ...
```

Supplementary Figure 30: Screenshot of the automated read-out of T2, sodium trifluoroacetate was used as additive during the measurement.

1.3.3.4 Synthesis of tetramer T3

Passerini reaction

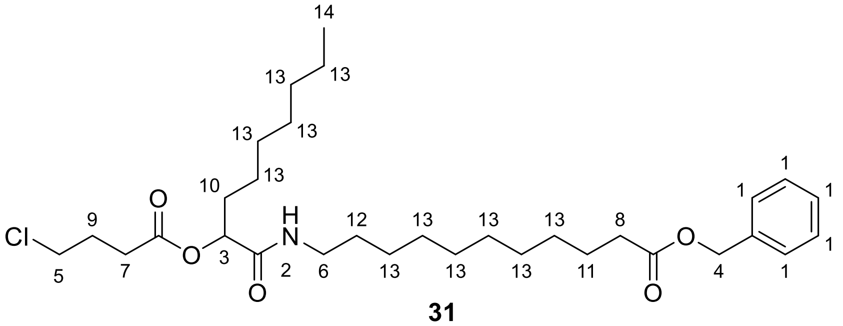

31

In a 50 mL round bottom flask, 161 mg 4-chlorobutyric acid **TAG3** (1.31 mmol, 1.00 eq.) was stirred in 2.00 mL DCM. Subsequently, 308 µL octanal **14i** (252 mg, 1.97 mmol, 1.50 eq.) and 594 mg of the monomer **M1** (1.97 mmol, 1.50 eq.) were added. The resulting reaction mixture was stirred at room temperature for 3 days. Afterwards, the crude mixture was dried under reduced pressure. The residue was adsorbed onto celite® and purified *via* column chromatography on silica gel eluting with a gradual solvent mixture of ethyl acetate and cyclohexane (8:1 → 6:1) to yield the passerini product **31** as a yellow highly viscous oil. (675 mg, 1.22 mmol, 86.2%).

R_f = 0.34 in cyclohexane / ethyl acetate (3:1).

IR (ATR): ν / cm^{-1} = 3306.6 (vw), 2924.1 (s), 2853.7 (m), 1736.1 (vs), 1655.7 (s), 1535.9 (m), 1455.5 (w), 1377.0 (w), 1144.0 (s), 733.8 (m), 697.0 (m), 650.8 (w). 1455.5 (w), 1377.0 (w), 1144.0 (s), 733.8 (m), 697.0 (m), 650.8 (w).

^{1}H NMR (400 MHz, $CDCl_3$): δ / ppm = 7.41 – 7.27 (m, 5 H, $CH_{Ar}{}^{1}$), 6.09 (t, *J* = 5.9 Hz, 1 H, NH^{2}), 5.18 – 5.12 (m, 1 H, CH^{3}), 5.09 (s, 2 H, $CH_2{}^{4}$), 3.60 (td, *J* = 6.3, 1.2 Hz, 2 H, $CH_2{}^{5}$), 3.32 – 3.16 (m, 2 H, $CH_2{}^{6}$), 2.59 (td, *J* = 7.1, 1.7 Hz, 2 H, $CH_2{}^{7}$), 2.33 (t, *J* = 7.5 Hz, 2 H, CH^{8}), 2.16 – 2.06 (m, 2 H, $CH_2{}^{9}$), 1.95 – 1.73 (m, 2 H, $CH_2{}^{10}$), 1.66 – 1.56 (m, 2 H, $CH_2{}^{11}$), 1.52 – 1.45 (m, 2 H, $CH_2{}^{12}$), 1.37 – 1.14 (m, 22 H, $CH_2{}^{13}$), 0.92 – 0.81 (m, 3 H, $CH_3{}^{14}$).

^{13}C NMR (101 MHz, $CDCl_3$): δ / ppm = 173.74, 171.54, 169.72, 136.19, 128.59, 128.20, 74.37, 66.11, 44.00, 39.30, 34.37, 31.99, 31.78, 31.16, 29.57, 29.49, 29.39, 29.26, 29.15, 29.14, 27.44, 26.88, 24.99, 24.85, 22.66, 14.13.

ESI-MS [*m*/z]: $[M+H]^+$ calculated for $^{12}C_{31}{}^{1}H_{50}{}^{16}O_5{}^{14}N^{35}Cl$, 552.3450; found: 552.3438; Δ = 1.2 mmu.

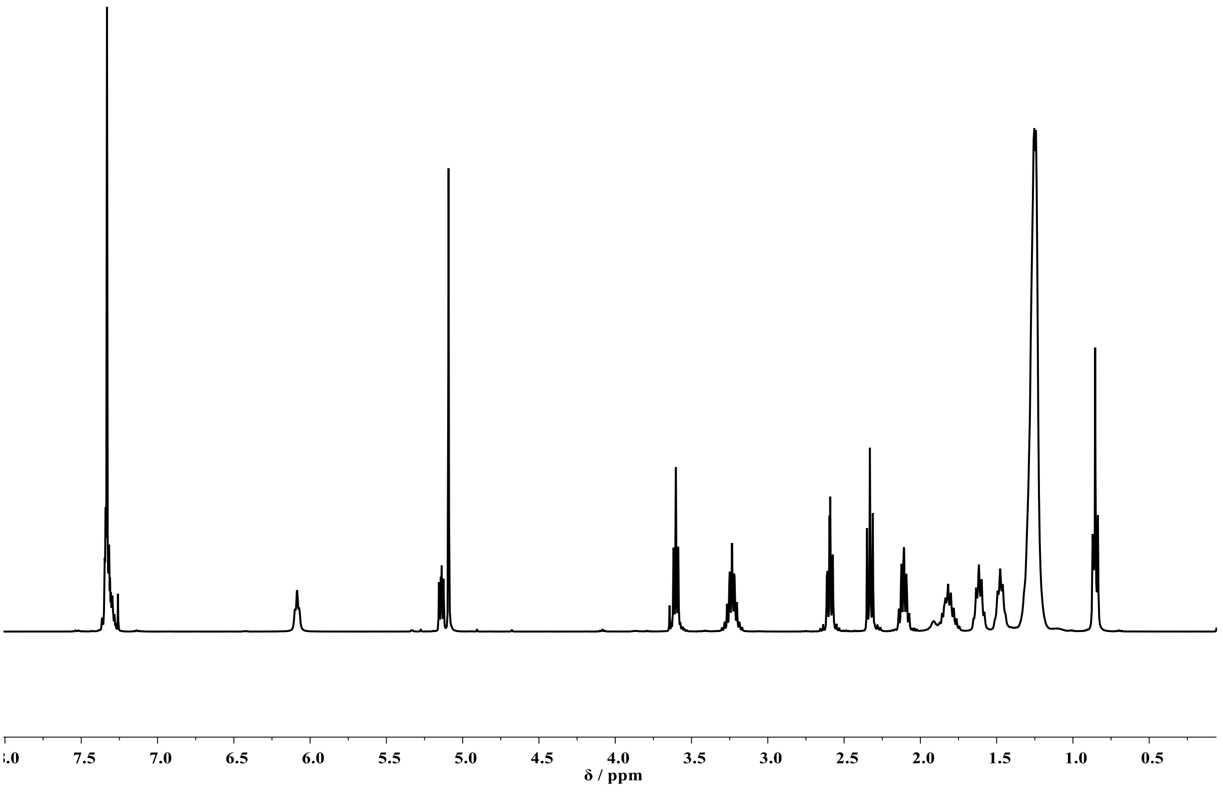

Supplementary Figure 31: ^{1}H-NMR of compound 31 measured in $CDCl_3$.

Deprotection

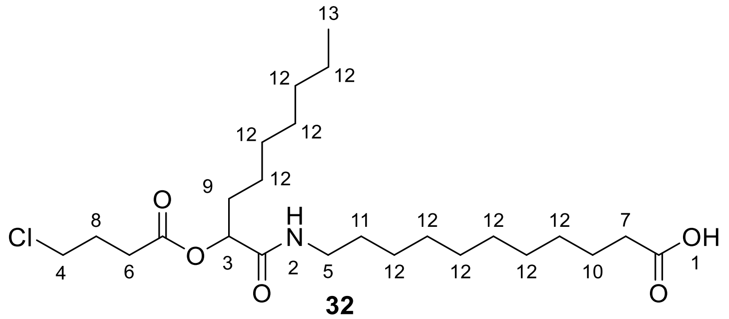

32

In a 50 mL round bottom flask, 606 mg of **31** (1.10 mmol, 1.00 eq.) were dissolved in 2.00 mL ethyl acetate and 2.00 mL THF. Afterwards, 121 mg (20 wt%) palladium on activated charcoal **16** were added. Subsequently, the mixture was purged with hydrogen (3 balloons) and stirred under hydrogen atmosphere overnight. The heterogeneous catalyst was filtered off and the solvent was evaporated under reduced pressure. The product **32** was obtained as a pale highly viscous oil in a yield of 98.2% (502 mg, 1.08 mmol).

IR (ATR): ν / cm^{-1} = 3294.8 (vw), 2923.7 (s), 2853.7 (m), 1737.3 (s), 1649.0 (s), 1541.6 (m), 1457.9 (w), 1376.1 (w), 1296.9 (w), 1173.7 (s), 1141.8 (s), 787.3 (w), 722.9 (w), 652.1 (w), 427.9 (vw).

^{1}H NMR (400 MHz, $CDCl_3$): δ / ppm = 8.65 (s (broad), 1 H, OH^1), 6.08 (t, J = 5.9 Hz, 1 H, NH^2), 5.13 – 5.02 (m, 1 H, CH^3), 3.61 – 3.51 (m, 2 H, $CH_2{}^4$), 3.28 – 3.11 (m, 2 H, $CH_2{}^5$), 2.62 – 2.49 (m, 2 H, $CH_2{}^6$), 2.26 (t, J = 7.5 Hz, 2 H, $CH_2{}^7$), 2.13 – 2.01 (m, 2 H, $CH_2{}^8$), 1.86 – 1.69 (m, 2 H, $CH_2{}^9$), 1.61 – 1.50 (m, 2 H, $CH_2{}^{10}$), 1.47 – 1.38 (m, 2 H, $CH_2{}^{11}$), 1.32 – 1.13 (m, 22 H, $CH_2{}^{12}$), 0.84 – 0.76 (m, 3 H, $CH_3{}^{13}$).
^{13}C NMR (101 MHz, $CDCl_3$): δ / ppm = 179.33, 171.62, 169.96, 74.37, 44.02, 39.37, 34.23, 31.98, 31.80, 31.18, 29.53, 29.47, 29.37, 29.27, 29.24, 29.16, 29.10, 27.44, 26.87, 24.85, 24.82, 22.68, 14.15.
ESI-MS [*m/z*]: $[M + H]^+$ calculated for $^{12}C_{24}{}^{1}H_{44}{}^{16}O_5{}^{14}N^{35}Cl$, 462.2981; found, 462.2971, Δ = 1.0 mmu.

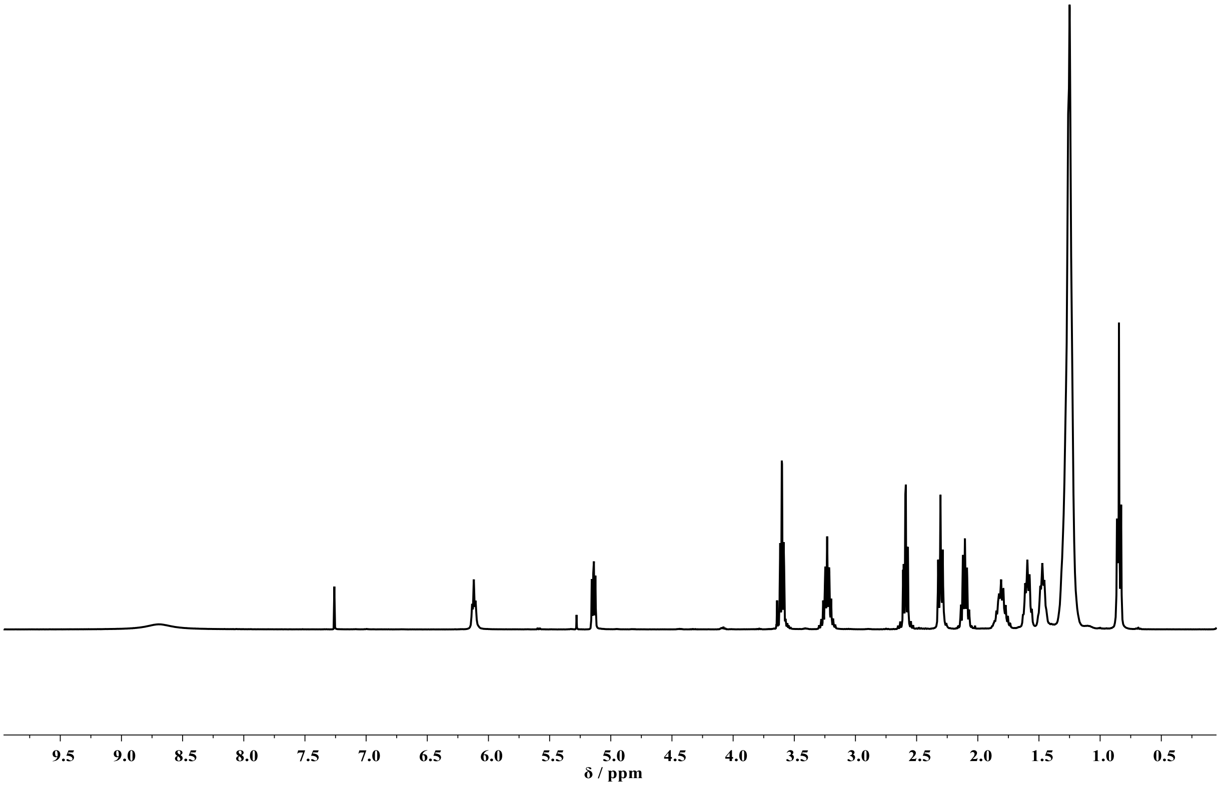

Supplementary Figure 32: ^{1}H-NMR of compound 32 measured in $CDCl_3$.

Passerini reaction

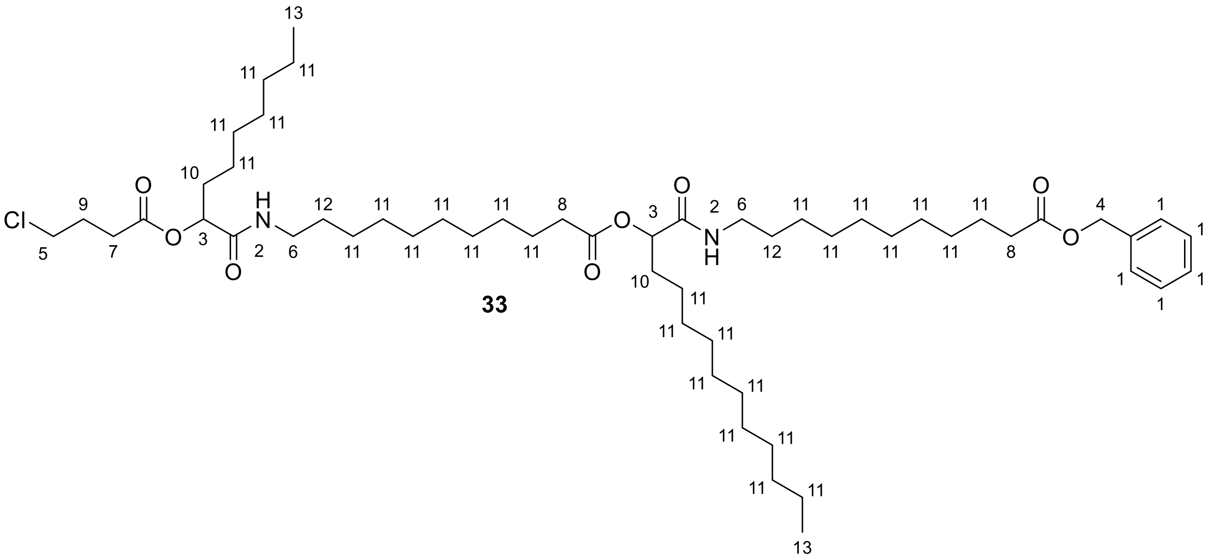

33

In a 50 mL round bottom flask, 882 mg **32** (815 µmol, 1.00 eq.) was stirred in 3.00 mL DCM. Subsequently, 564 mg dodecanal **14h** (1.22 mmol, 1.50 eq.) and 922 mg of the monomer **M1**(1.22 mmol, 1.50 eq.) were added. The resulting reaction mixture was stirred at room temperature for 3 days. Afterwards, the crude mixture was dried under reduced pressure. The residue was adsorbed onto celite® and purified *via* column chromatography on silica gel eluting with a gradual solvent mixture of ethyl acetate and cyclohexane (7:1 → 4:1) to yield the passerini product **33** as a yellow highly viscous oil. (1.65 g, 755 µmol, 92.6%).

R_f: 0.21 in cyclohexane / ethyl acetate (3:1).

IR (ATR): ν / cm^{-1} = 3304.4 (w), 2922.2 (s), 2852.3 (m), 1737.3 (s), 1654.0 (s), 1536.8 (m), 1456.1 (w), 1376.6 (w), 1166.4 (m), 723.0 (w), 696.9 (w).

^{1}H NMR (400 MHz, $CDCl_3$): δ / ppm = 7.41 – 7.29 (m, 5 H, CH_{Ar}^{1}), 6.08 – 5.92 (m, 2 H, NH^{2}), 5.21 – 5.14 (m, 2 H, CH^{3}), 5.11 (s, 2 H, CH^{4}), 3.66 – 3.59 (m, 2 H, CH_2^{5}), 3.32 – 3.19 (m, 4 H, CH_2^{6}), 2.61 (td, J = 7.1, 1.7 Hz, 2 H, CH_2^{7}), 2.41 – 2.31 (m, 4 H, CH_2^{8}), 2.19 – 2.07 (m, 2 H, CH_2^{9}), 1.92 – 1.73 (m, 4 H, CH_2^{10}), 1.69 – 1.57 (m, 8 H, CH_2^{11}), 1.54 – 1.42 (m, 4 H, CH_2^{12}), 1.36 – 1.19 (m, 48 H, CH_2^{11}), 0.91 – 0.83 (m, 6 H, CH_3^{13}).

^{13}C NMR (101 MHz, $CDCl_3$): δ / ppm = 172.68, 171.45, 170.46, 168.84, 168.62, 135.11, 127.52, 127.15, 73.32, 72.93, 65.05, 42.92, 38.21, 38.18, 33.30, 30.89, 30.70, 30.09, 28.60, 28.53, 28.52, 28.43, 28.42, 28.34, 28.33, 28.24, 28.19, 28.18, 28.09, 28.06, 26.35, 25.81, 23.94, 23.92, 23.77, 23.74, 21.67, 21.59, 13.10, 13.05.

ESI-MS [*m*/z]: $[M+H]^+$ calculated for $^{12}C_{55}{}^{1}H_{95}{}^{16}O_{8}{}^{14}N_{2}{}^{35}Cl$, 947.6850; found: 947.6823; Δ = 1.8 mmu.

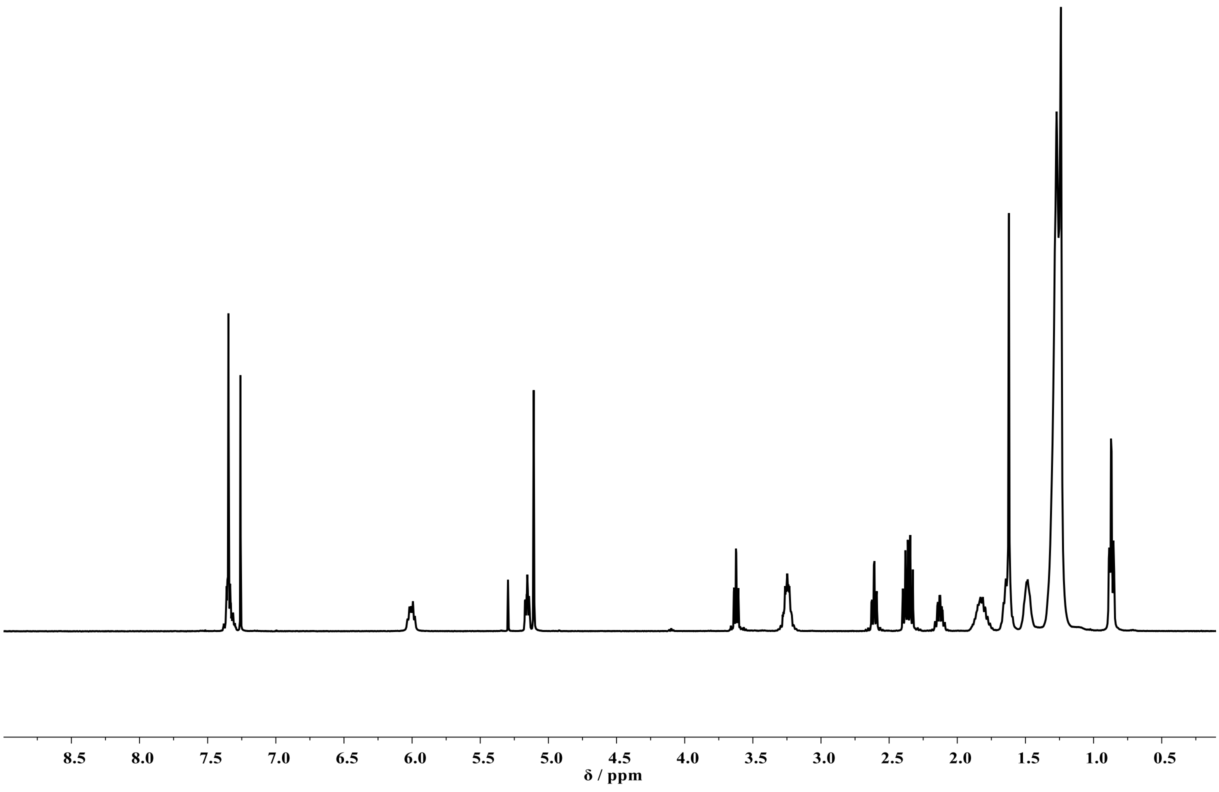

Supplementary Figure 33: ^{1}H-NMR of compound 33 measured in $CDCl_3$.

Deprotection

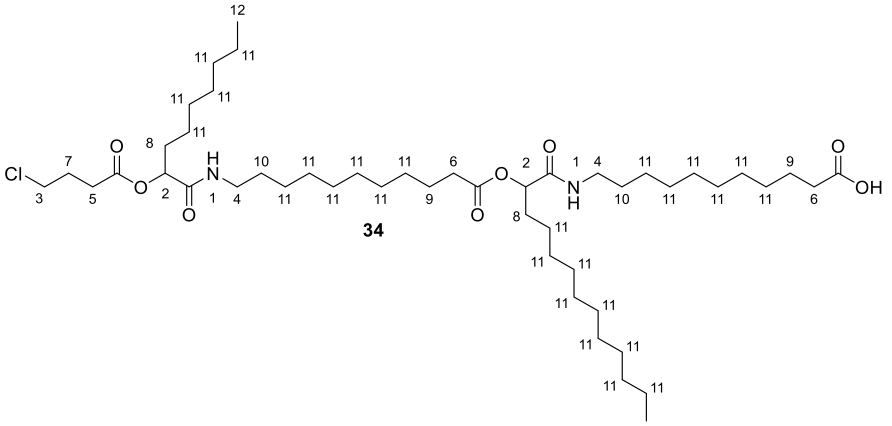

34

In a 50 mL round bottom flask, 525 mg of **33** (610 µmol, 1.00 eq.) were dissolved in 3.00 mL ethyl acetate and 3.00 mL THF. Afterwards, 117 mg (20 wt%) palladium on activated charcoal **16** were added. Subsequently, the mixture was purged with hydrogen (3 balloons) and stirred under hydrogen atmosphere overnight. The heterogeneous catalyst was filtered off and the solvent was evaporated under reduced pressure. The product **34** was obtained as a pale highly viscous oil in a yield of 96.7% (502 mg, 590 µmol).

IR (ATR): ν / cm^{-1} = 3291.2 (w), 2919.5 (vs), 2850.7 (s), 1736.6 (s), 1698.2 (m), 1655.5 (vs), 1560.4 (m), 1466.9 (m), 1377.2 (w), 1302.3 (w), 1170.6 (s), 938.1 (vw), 721.1 (w), 445.9 (vw).

^{1}H NMR (400 MHz, $CDCl_3$): δ / ppm = 6.04 (t, J = 5.9 Hz, 1 H, NH^{1}), 5.99 (t, J = 5.8 Hz, 1 H, NH^{1}), 5.14 – 5.06 (m, 2 H, CH^{2}), 3.67 – 3.48 (m, 2 H, $CH_2{}^{3}$), 3.25 – 3.10 (m, 4 H, $CH_2{}^{4}$), 2.55 (td, J = 7.1, 1.9 Hz, 2 H, $CH_2{}^{5}$), 2.32 (t, J = 7.5 Hz, 2 H, $CH_2{}^{6}$), 2.27 (t, J = 7.5 Hz, 2 H, $CH_2{}^{6}$), 2.11 – 2.00 (m, 2 H, $CH_2{}^{7}$), 1.87 – 1.65 (m, 4 H, $CH_2{}^{8}$), 1.65 – 1.49 (m, 4 H, $CH_2{}^{9}$), 1.50 – 1.31 (m, 4 H, $CH_2{}^{10}$), 1.32 – 0.98 (m, 52 H, $CH_2{}^{11}$), 0.84 – 0.74 (m, 6 H, $CH_3{}^{12}$).

^{13}C NMR (101 MHz, $CDCl_3$): δ / ppm = 178.05, 172.63, 171.68, 170.11, 169.97, 74.45, 74.08, 44.06, 39.42, 39.32, 34.45, 34.05, 32.04, 31.84, 31.24, 29.75, 29.66, 29.63, 29.58, 29.57, 29.50, 29.47, 29.37, 29.32, 29.24, 29.20, 29.11, 27.49, 26.95, 26.89, 25.10, 24.90, 24.89, 24.87, 22.81, 22.73, 14.25, 14.20.

ESI-MS [*m/z*]: $[M + H]^+$ calculated for $^{12}C_{48}{}^{1}H_{89}{}^{16}O_{8}{}^{14}N_{2}{}^{35}Cl$, 857.6380 found, 857.6366, Δ = 1.4 mmu.

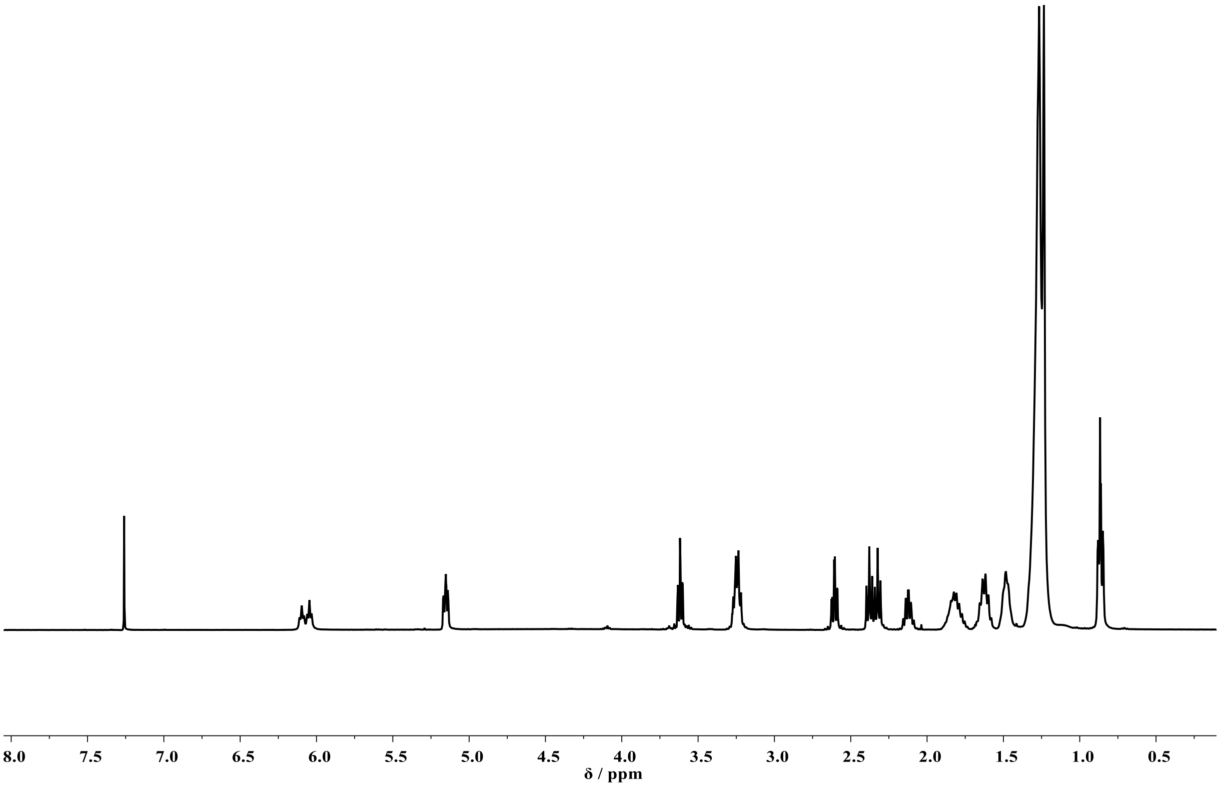

Supplementary Figure 34: ^{1}H-NMR of compound 34 measured in $CDCl_3$.

Passerini reaction

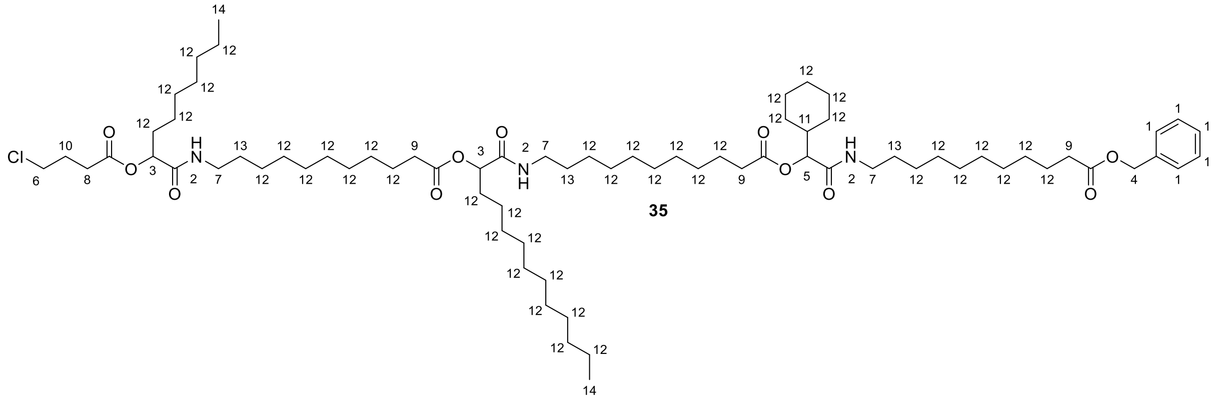

35

In a 50 mL round bottom flask, 1.35 g **34** (1.58 mmol, 1.00 eq.) was stirred in 2.00 mL DCM. Subsequently, 286 µL cyclohexanecarboxaldehyde **14j** (265 mg, 2.37 mmol, 1.50 eq.) and 714 mg of the monomer **M1** (2.37 mmol, 1.50 eq.) were added. The resulting reaction mixture was stirred at room temperature for 3 days. Afterwards, the crude mixture was dried under reduced pressure. The residue was adsorbed onto celite® and purified *via* column chromatography on silica gel eluting with a gradual solvent mixture of ethyl acetate and cyclohexane (4:1 → 2:1) to yield the passerini product **35** as a yellow highly viscous oil. (1.88 g, 1.48 mmol, 93.7%).

R_f: 0.20 in cyclohexane / ethyl acetate (3:1).

IR (ATR): ν / cm^{-1} = 3293.1 (w), 2918.0 (vs), 2850.4 (s), 1733.1 (vs), 1655.0 (vs), 1558.1 (m), 1466.2 (m), 1378.1 (m), 1238.1 (m), 1207.0 (s), 1172.8 (vs), 980.7 (w), 721.9 (m), 695.9 (m), 453.1 (vw).

^{1}H NMR (400 MHz, $CDCl_3$): δ / ppm = 7.41 – 7.29 (m, 5 H, CH_{Ar}^{1}), 6.09 – 6.00 (m, 2 H, NH^{2}), 5.95 (t, *J* = 6.0 Hz, 1 H, NH^{2}), 5.18 – 5.12 (m, 2 H, CH^{3}), 5.10 (s, 2 H, CH_2^{4}), 5.02 (d, *J* = 4.6 Hz, 1 H, CH^{5}), 3.67 – 3.58 (m, 2 H, CH_2^{6}), 3.32 – 3.16 (m, 6 H, CH_2^{7}), 2.61 (td, *J* = 7.1, 1.9 Hz, 2 H, CH_2^{8}), 2.44 – 2.29 (m, 6 H, CH_2^{9}), 2.19 – 2.07 (m, 2 H, CH_2^{10}), 2.00 – 1.58 (m, 17 H, CH^{11}, CH_2^{12}), 1.54 – 1.42 (m, 6 H, CH_2^{13}), 1.39 – 1.05 (m, 68 H, CH_2^{12}), 0.91 – 0.82 (m, 6 H, CH_3^{14}).

^{13}C NMR (101 MHz, $CDCl_3$): δ / ppm = 173.81, 172.67, 172.60, 171.61, 170.00, 169.77, 169.33, 136.23, 128.66, 128.29, 128.28, 77.74, 74.44, 74.05, 66.19, 44.07, 40.09, 39.35, 39.30, 39.27, 34.44, 34.41, 32.04, 32.03, 31.84, 31.22, 29.74, 29.68, 29.66, 29.58, 29.57, 29.49, 29.46, 29.38, 29.35, 29.32, 29.25, 29.22, 29.20, 27.49, 27.41, 26.96, 26.95, 26.19, 26.11, 26.00, 25.11, 25.08, 25.06, 24.90, 24.89, 22.81, 22.73, 14.25, 14.20.

ESI-MS [*m*/z]: $[M+Na]^+$ calculated for $^{12}C_{74}{}^{1}H_{128}{}^{16}O_{11}{}^{14}N_3{}^{35}Cl$, 1292.9130; found: 1292.9134; Δ = 0.4 mmu.

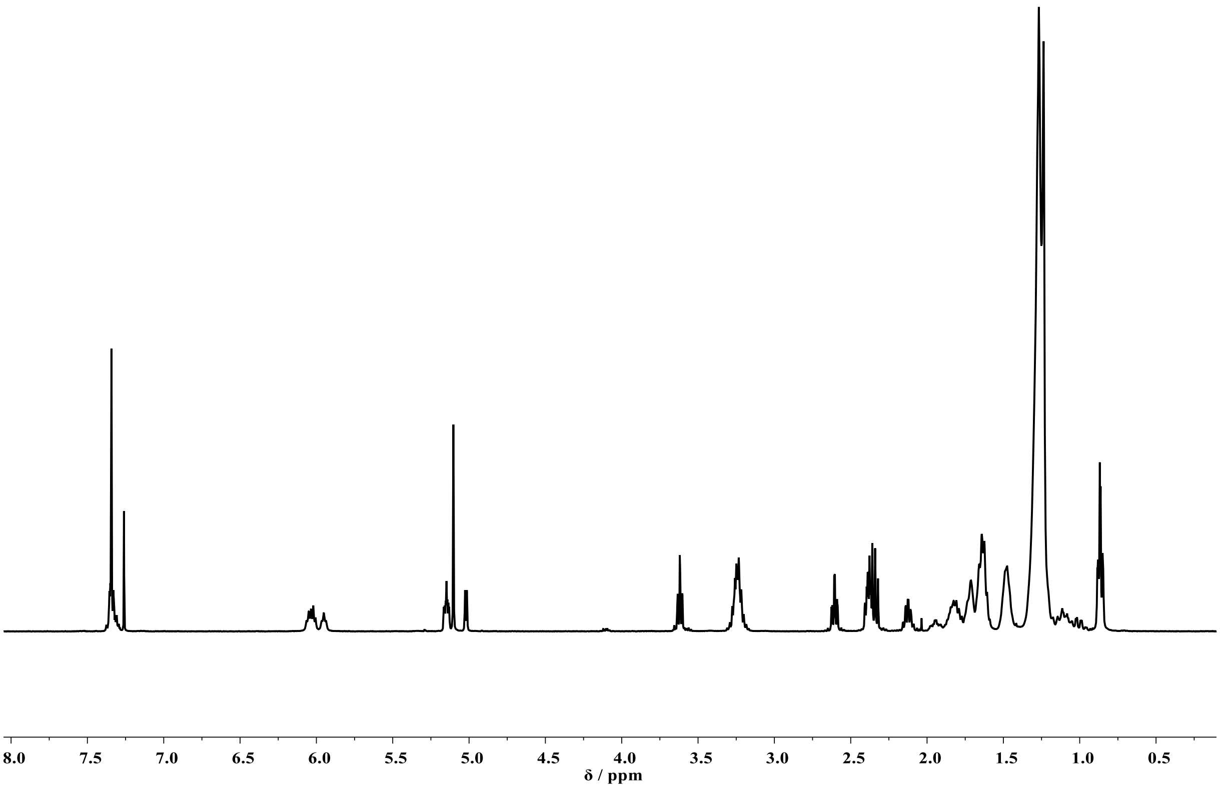

Supplementary Figure 35: ^{1}H-NMR of compound 35 measured in $CDCl_3$.

Deprotection

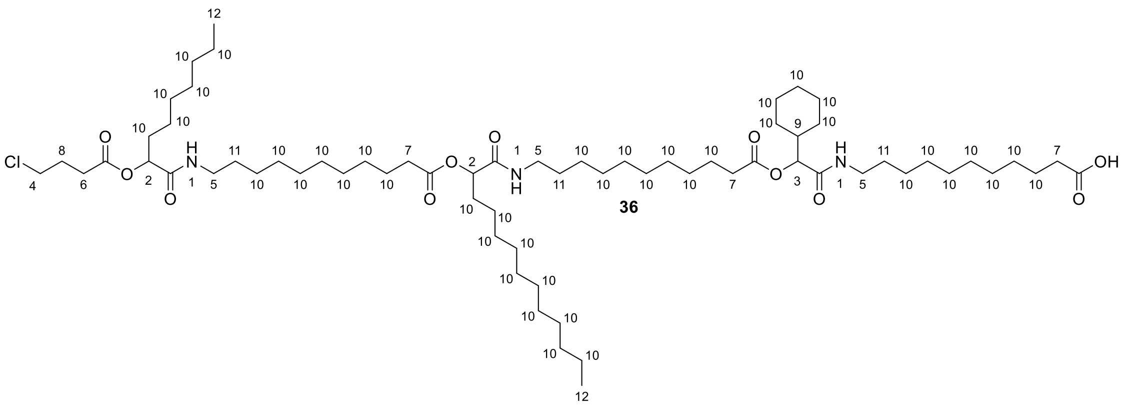

36

In a 50 mL round bottom flask, 1.49 g of **35** (1.17 mmol, 1.00 eq.) were dissolved in 4.00 mL ethyl acetate and 4.00 mL THF. Afterwards, 149 mg (10 wt%) palladium on activated charcoal **16** were added. Subsequently, the mixture was purged with hydrogen (3 balloons) and stirred under hydrogen atmosphere overnight. The heterogeneous catalyst was filtered off and the solvent was evaporated under reduced pressure. The product **36** was obtained as a pale highly viscous oil in a yield of 99.1% (1.37 g, 1.16 mmol).

IR (ATR): ν / cm^{-1} = 3292.4 (w), 2918.6 (vs), 2850.6 (vs), 1735.6 (s), 1654.9 (vs), 1556.3 (m), 1466.0 (m), 1377.5 (m), 1244.8 (m), 1207.9 (s), 1177.3 (vs), 941.3 (vw), 721.6 (w), 454.0 (vw).

^{1}H NMR (400 MHz, $CDCl_3$): δ / ppm = 6.06 – 5.97 (m, 2 H, NH^{1}), 5.92 (t, J = 6.0 Hz, 1 H, NH^{1}), 5.13 – 5.06 (m, 2 H, CH^{2}), 4.97 (d, J = 4.6 Hz, 1 H, CH^{3}), 3.62 – 3.51 (m, 2 H, CH_2^{4}), 3.24 – 3.09 (m, 6 H, CH_2^{5}), 2.55 (td, J = 7.1, 1.8 Hz, 2 H, CH_2^{6}), 2.36 – 2.22 (m, 6 H, CH_2^{7}), 2.12 – 2.01 (m, 2 H, CH_2^{8}), 1.93 – 1.51 (m, 15 H, CH^{9}, CH_2^{10}), 1.50 – 1.38 (m, 6 H, CH_2^{11}), 1.32 – 0.93 (m, 70 H, CH_2^{10}), 0.85 – 0.74 (m, 6 H, CH_3^{12}).

^{13}C NMR (101 MHz, $CDCl_3$): δ / ppm = 172.71, 171.65, 170.19, 169.90, 169.44, 77.78, 74.46, 74.08, 44.08, 40.06, 39.40, 39.24, 34.45, 33.96, 32.05, 32.01, 31.86, 31.25, 29.76, 29.68, 29.65, 29.61, 29.58, 29.53, 29.50, 29.49, 29.41, 29.38, 29.35, 29.34, 29.29, 29.24, 29.21, 29.20, 29.08, 27.50, 27.42, 26.96, 26.89, 26.20, 26.12, 26.01, 25.16, 25.09, 24.92, 24.91, 22.82, 22.74, 14.26, 14.21.

ESI-MS [*m/z*]: $[M + H]^+$ calculated for $^{12}C_{67}{}^{1}H_{122}{}^{16}O_{11}{}^{14}N_{3}{}^{35}Cl$, 1180.8841 found, 1180.8837, Δ = 0.4 mmu.

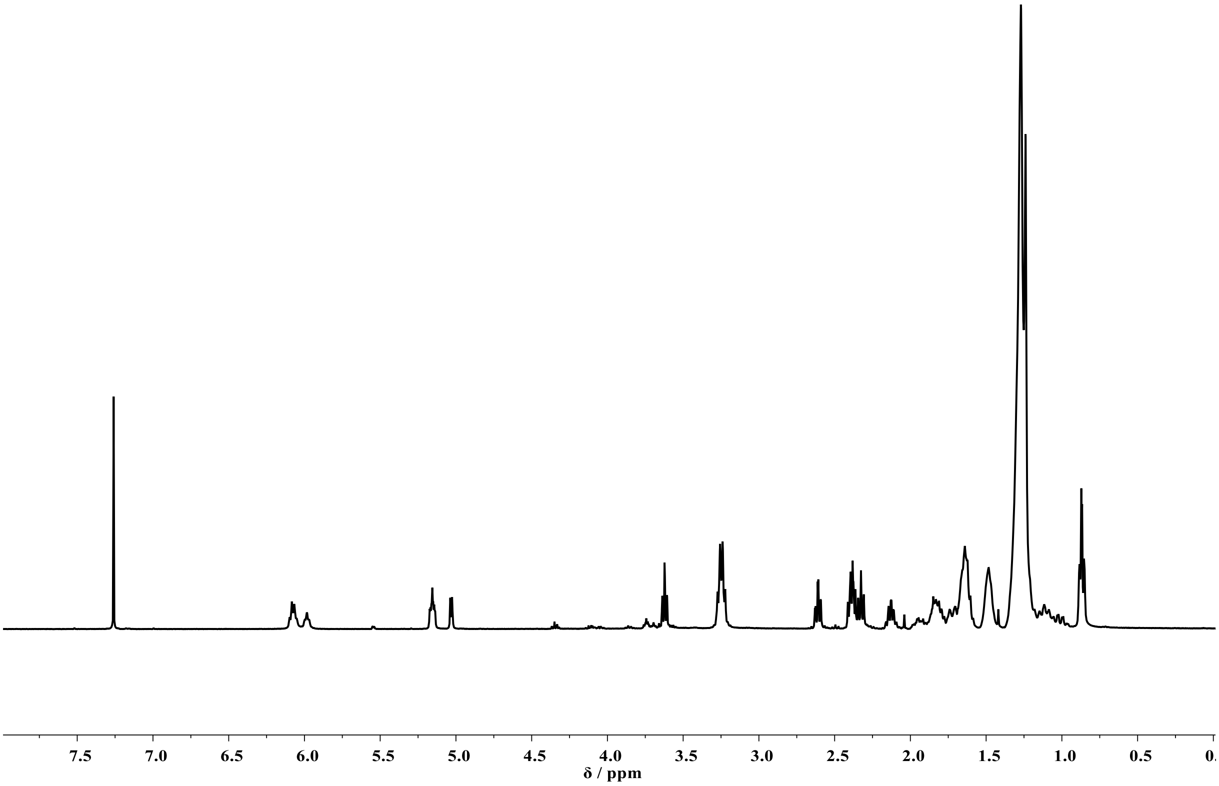

Supplementary Figure 36: ^{1}H-NMR of compound 36 measured in $CDCl_3$.

Passerini reaction

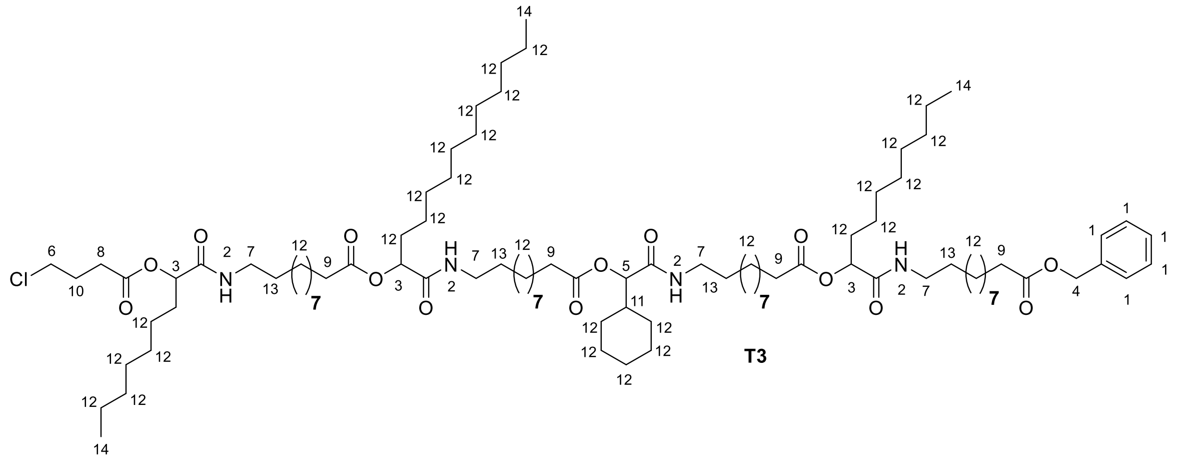

In a 50 mL round bottom flask, 415 mg **36** (351 mol, 1.00 eq.) was stirred in 2.00 mL DCM. Subsequently, 91.6 µL nonanal **14k** (74.9 mg, 527 µmol, 1.50 eq.) and 159 mg of the monomer **M1** (517 µmol, 1.50 eq.) were added. The resulting reaction mixture was stirred at room temperature for 3 days. Afterwards, the crude mixture was dried under reduced pressure. The residue was adsorbed onto celite® and purified *via* column chromatography on silica gel eluting with a gradual solvent mixture of ethyl acetate and cyclohexane (5:1 → 2:1) to yield the passerini product **T3** as a yellow highly viscous oil. (527 mg, 323 µmol, 92.0%).

R_f: 0.17 in cyclohexane / ethyl acetate (2:1).

IR (ATR): ν / cm^{-1} =3291.6 (w), 2920.0 (vs), 2851.1 (s), 1736.4 (vs), 1654.9 8 (vs), 1557.0 (m), 1465.8 (m), 1377.3 (w), 1243.8 (m), 1207.8 (m), 1173.6 (vs), 1112.7 (m), 721.9 (w), 696.4 (m), 453.3 (vw), 384.2 (vw).

^{1}H NMR (500 MHz, $CDCl_3$): δ / ppm = 7.44 – 7.31 (m, 5 H, CH_{Ar}^1), 6.10 – 5.99 (m, 3 H, NH^2), 5.97 – 5.87 (m, 1 H, NH^2), 5.18 – 5.13 (m, 3 H, CH^3), 5.11 (s, 2 H, CH_2^4), 5.02 (d, J = 4.6 Hz, 1 H, CH^5), 3.67 – 3.56 (m, 2 H, CH_2^6), 3.33 – 3.16 (m, 8 H, CH_2^7), 2.67 – 2.56 (m, 2 H, CH_2^8), 2.44 – 2.29 (m, 8 H, CH_2^9), 2.19 – 2.10 (m, 2 H, CH_2^{10}), 2.01 – 1.58 (m, 23 H, CH^{11}, CH_2^{12}), 1.54 – 1.44 (m, 8 H, CH_2^{13}), 1.38 – 0.97 (m, 88 H, CH_2^{12}), 0.94 – 0.80 (m, 9 H, CH_3^{14}).

^{13}C NMR (126 MHz, $CDCl_3$): δ / ppm = 173.83, 172.70, 172.61, 171.62, 170.01, 169.98, 169.78, 169.35, 136.26, 128.68, 128.30, 77.76, 74.47, 74.07, 66.21, 44.09, 40.11, 34.46, 34.43, 32.07, 32.06, 31.96, 31.86, 31.24, 30.46, 29.76, 29.73, 29.71, 29.70, 29.68, 29.60, 29.58, 29.53, 29.51, 29.49, 29.40, 29.36, 29.35, 29.33, 29.28, 29.25, 29.22, 27.50, 27.43, 26.99, 26.97, 26.21, 26.13, 26.02, 25.13, 25.10, 25.08, 24.92, 24.89, 22.83, 22.78, 22.75, 14.27, 14.25, 14.22.

ESI-MS [*m*/z]: $[M+Na]^+$ calculated for $^{12}C_{95}{}^{1}H_{167}{}^{16}O_{14}{}^{14}N_4{}^{35}Cl$, 1646.2060; found: 1646.2066; Δ = 0.6 mmu.

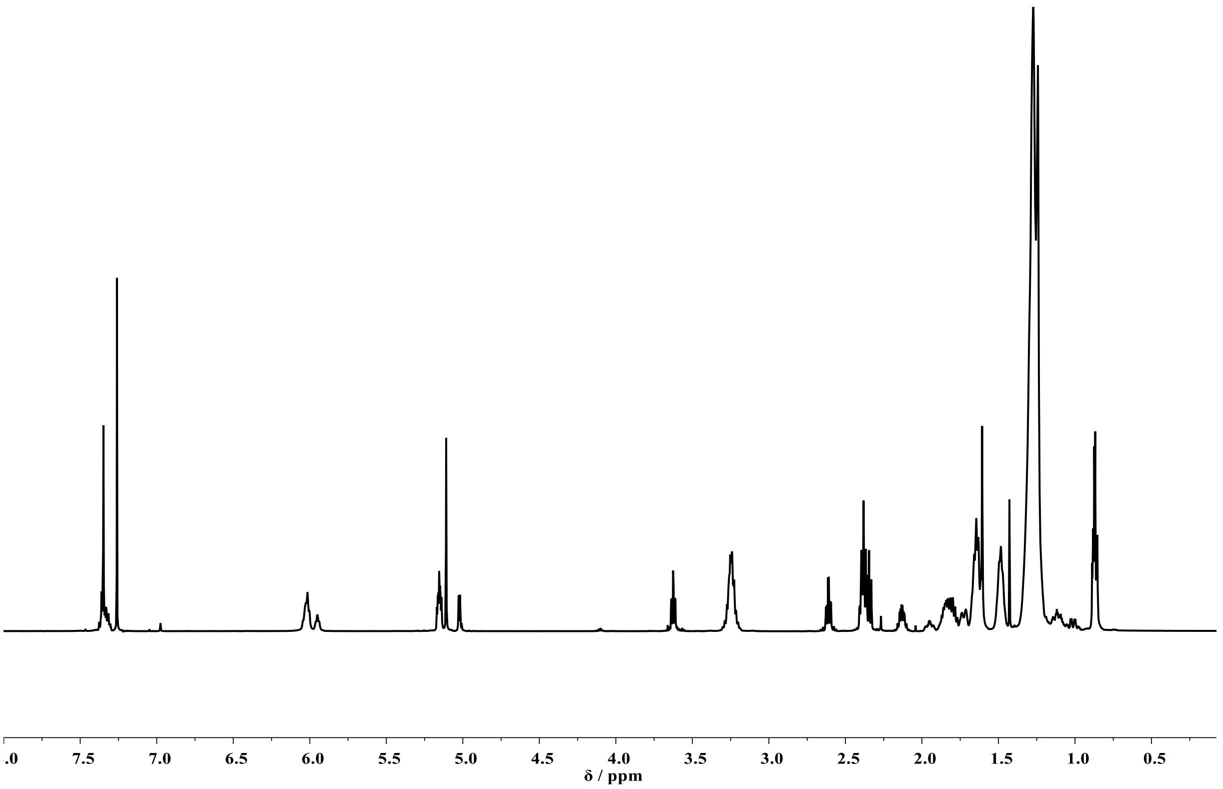

Supplementary Figure 37: ^{1}H-NMR of compound T3 measured in $CDCl_3$.

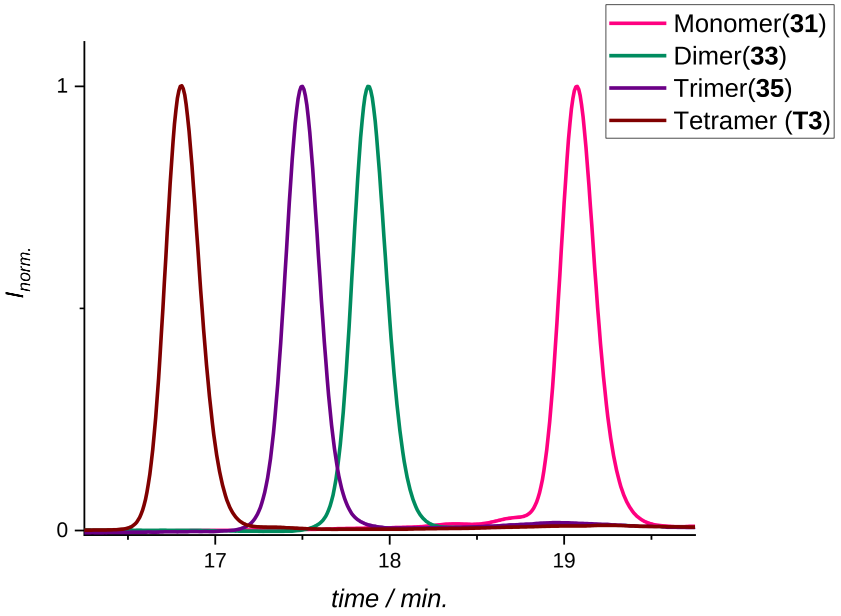

Supplementary Figure 38: SEC traces of the intermediates after each P3CR in the synthesis of product T3.

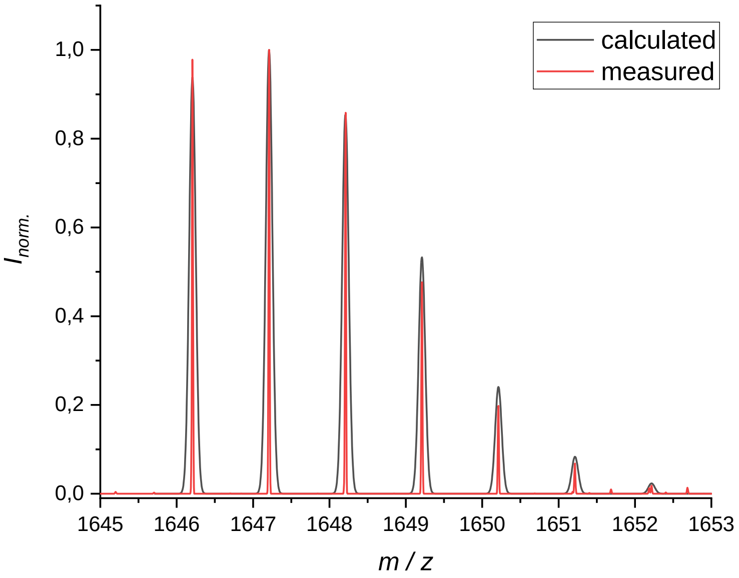

Supplementary Figure 39: High resolution ESI-MS measurement of T3. The observed isotopic pattern is compared with the calculated isotopic pattern obtained from mMass (black).

```
tramer.CSV, maximum is 1.000000 found for mass 1647.207720
matching mass 1647.20772
cutoff 0.50000: 0 solutions (10 peaks)
cutoff 0.25000: 0 solutions (24 peaks)
cutoff 0.12500: 0 solutions (62 peaks)
cutoff 0.06250: 0 solutions (121 peaks)
cutoff 0.03125: 0 solutions (193 peaks)
cutoff 0.01562: 0 solutions (305 peaks)
cutoff 0.00781: 1 solutions (436 peaks)
1647.20772 ≈ 121.005630 + 339.277350 + 395.339950 + 323.246050 + 353.293000 + 91.054780 (sides Octanal, Dodecanal,
 Cyclohexancarboxaldehyde, Nonanal; error -23.99096)
Press ENTER to quit ...
```

Supplementary Figure 40: Screenshot of the automated read-out of T3, sodium trifluoroacetate was used as additive during the measurement.

1.3.3.5 Synthesis of tetramer T4

Passerini reaction

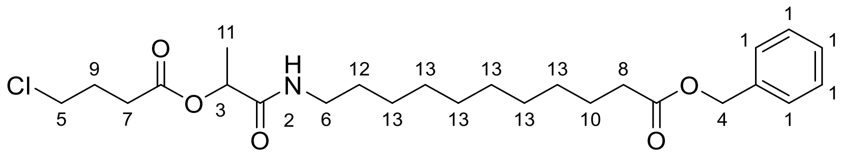

37

In a 50 mL round bottom flask, 125 mg 4-chlorobutyric acid **TAG3** (1.02 mmol, 1.00 eq.) was dissolved in 2.00 mL dichloromethane and 171 µL acetaldehyde **14f** (461 mg, 3.06 mmol, 3.00 eq.) and 461 mg of monomer **M1** (1.53 mol, 3.00 eq.) were added. The mixture was stirred at room temperature for 3 days. Subsequently, the solvent was removed under reduced pressure. The residue was adsorbed onto celite® and purified *via* column chromatography on silica gel eluting with a gradual solvent mixture of ethyl acetate and cyclohexane (6:1 → 2:1) to yield the passerini product **37** as a pale highly viscous oil with 92.3% (441 mg, 941 µmol).

R_f = 0.16 in cyclohexane / ethyl acetate (3:1).

IR (ATR): ν / cm^{-1} = 3317.5 (vw), 2926.7 (w), 2854.4 (w), 1734.8 (s), 1660.0 (m), 1538.4 (w), 1498.1 (vw), 1454.9 (w), 1373.5 (w), 1298.4 (w), 1166.8 (m), 1144.5 (s), 1097.3 (w), 1031.5 (vw), 878.2 (vw), 787.6 (vw), 735.8 (w), 697.2 (m), 648.5 (vw), 432.2 (vw).

^{1}H NMR (400 MHz, $CDCl_3$): δ / ppm = 7.40 – 7.28 (m, 5 H, CH^1), 6.10 (t, J = 5.9 Hz, 1H, NH^2), 5.20 (q, J = 6.8 Hz, 1 H, CH^3), 5.10 (s, 2 H, CH_2^4), 3.71 – 3.55 (m, 2 H, CH_2^5), 3.32 – 3.19 (m, 2 H, CH_2^6), 2.59 (td, J = 7.1, 2.7 Hz, 2 H, CH_2^7), 2.34 (t, J = 7.5 Hz, 2 H, CH_2^8), 2.21 – 2.04 (m, 2 H, CH_2^9), 1.69 – 1.56 (m, 2 H, CH_2^{10}), 1.55 – 1.43 (m, 5 H, CH^{11}, CH_2^{12}), 1.34 – 1.21 (m, 12 H, CH_2^{13}).

^{13}C NMR (101 MHz, $CDCl_3$): δ / ppm = 173.81, 171.36, 170.21, 136.23, 128.65, 128.27, 70.93, 66.18, 44.06, 39.39, 34.43, 31.29, 29.60, 29.52, 29.43, 29.31, 29.30, 29.20, 27.46, 26.92, 25.04, 18.09.

ESI-MS [*m/z*]: $[M + H]^+$ calculated for $^{12}C_{25}{}^{1}H_{38}{}^{16}O_5{}^{14}N^{35}Cl$, 468.2511; found, 468.2511, Δ = 0.0 mmu.

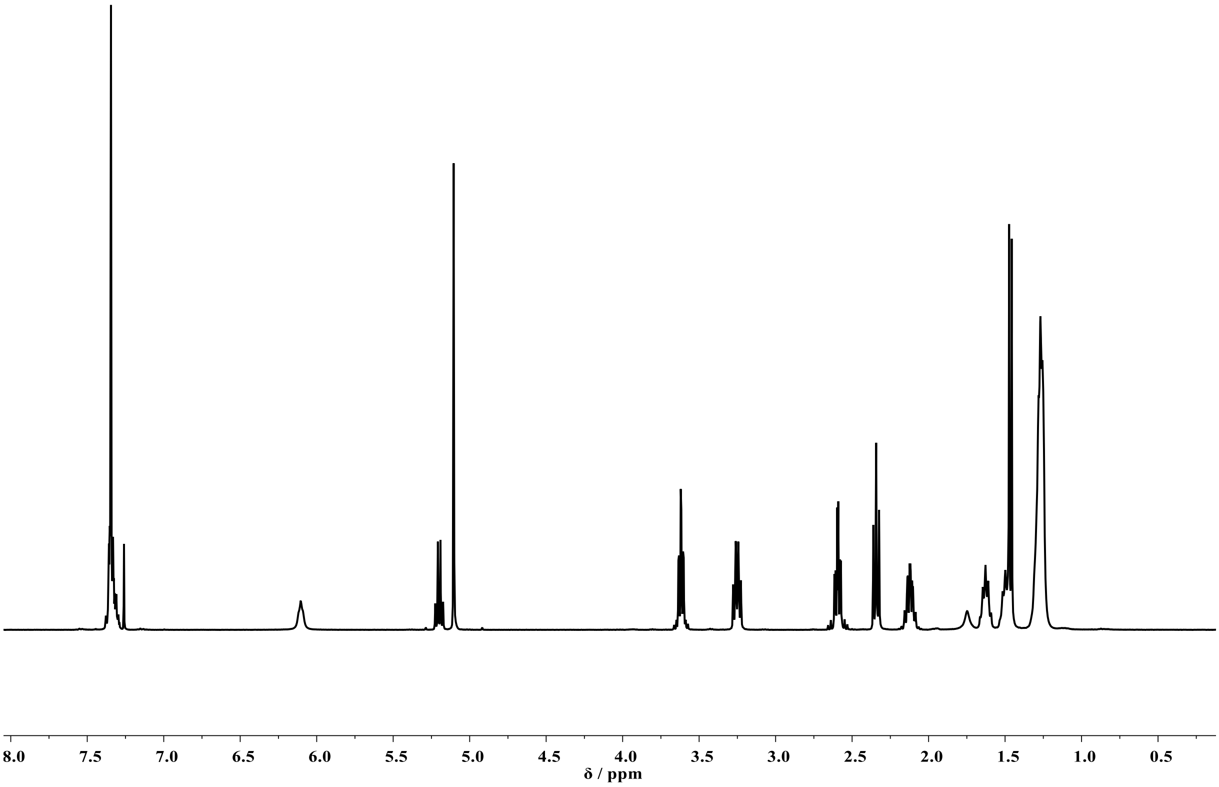

Supplementary Figure 41: ^{1}H-NMR of compound 37 measured in $CDCl_3$.

Deprotection

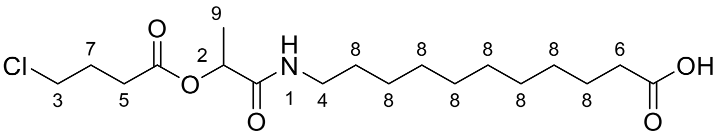

38

In a 25 mL round bottom flask equipped with a magnetic stir bar, 394 mg of passerini product **37** (841 µmol, 1.00 eq.) was dissolved in 4.00 mL ethyl acetate and 4.00 mL THF. Subsequently, 99.4 mg (20 wt.-%) palladium on activated charcoal **16** was added to the solution. The resulting mixture was purged with hydrogen gas and stirred for 2 d at room temperature under hydrogen atmosphere (3 balloons). The heterogeneous catalyst was filtered over celite® and the solvent was evaporated under reduced pressure. The product **38** was obtained as a pale highly viscous oil in a yield of 97.5% (310 mg, 820 µmol).

IR (ATR): ν / cm^{-1} = 3295.2790 (w), 2919.4 (m), 2850.1 (w), 1729.4 (w), 1690.9 (w), 1653.4 (m), 1550.8 (m), 1453.5 (w), 1435.5 (w), 1407.6 (w), 1372.9 (w), 1330.3 (w), 1278.1 (w), 1256.2 (w), 1235.8 (w), 1187.4 (w), 1145.0(w), 1092.2 (w), 1057.9 (w), 1037.6 (w), 999.7 (w), 880.21 (vw), 840.8 (vw), 721.4 (w), 686.6 (w), 542.3(vw), 431.0 (vw).

^{1}H NMR (300 MHz, CDCl$_3$): δ / ppm = 6.26 – 5.72 (m, 1 H, NH^1), 5.27 – 5.06 (m, 1 H, CH^2), 3.92 – 3.53 (m, 2 H, CH$_2$^{3}), 3.49 – 3.17 (m, 2 H, CH$_2$^{4}), 2.69 – 2.45 (m, 2 H, CH$_2$^{5}), 2.32 (t, J = 7.5 Hz, 2 H, CH$_2$^{6}), 2.19 – 1.86 (m, 2 H, CH$_2$^{7}), 2.03 – 0.77 (m, 19 H, CH$_2$^{8}, CH$_3$^{9}).
^{13}C NMR (126 MHz, CDCl$_3$): δ / ppm = 178.74, 171.41, 170.44, 70.89, 67.31, 67.12, 64.18, 62.76, 62.38, 44.05, 39.43, 34.08, 31.27, 29.92, 29.51, 29.43, 29.34, 29.22, 29.08, 27.44, 26.86, 24.80, 18.06.
ESI-MS [m/z]: [M + H]$^+$ calculated for $^{12}C_{18}{}^{1}H_{33}{}^{16}O_5{}^{14}N^{35}Cl$, 378.2042; found, 378.2030, Δ = 1.2 mmu.

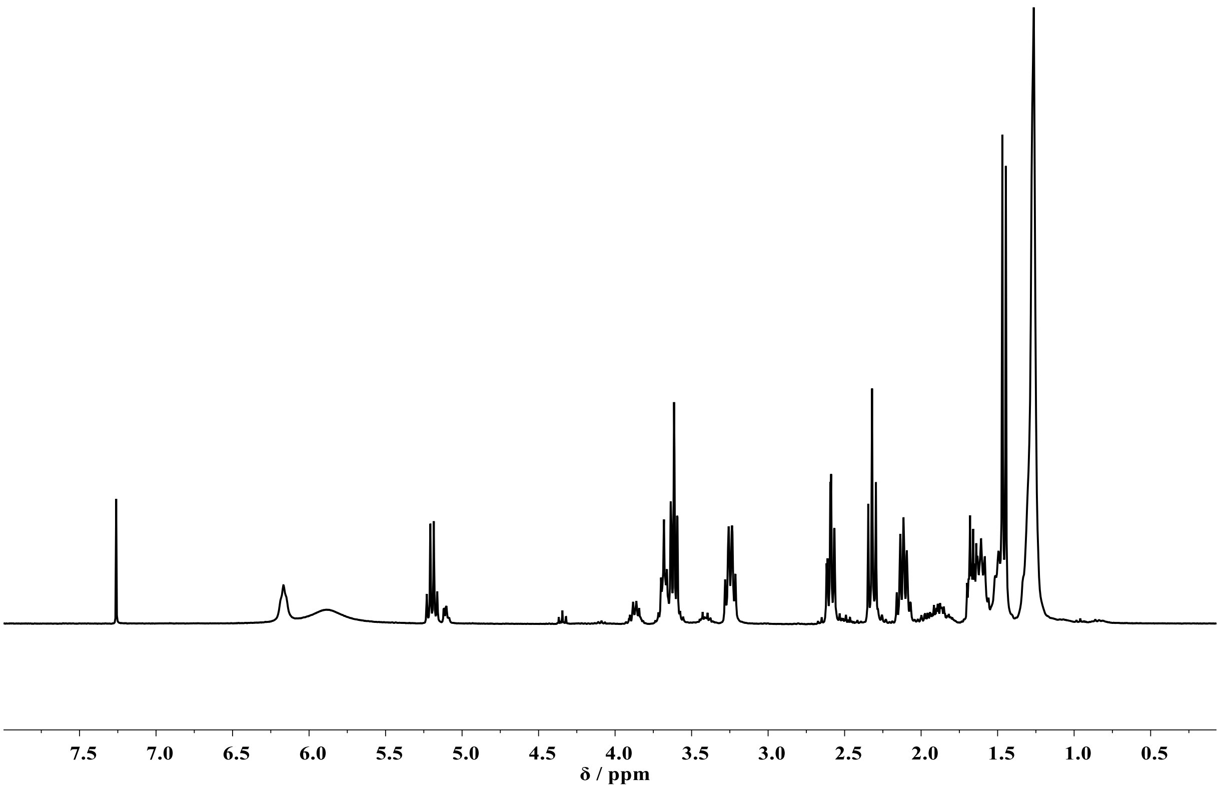

Supplementary Figure 42: ^{1}H-NMR of compound 38 measured in CDCl$_3$.

Passerini reaction

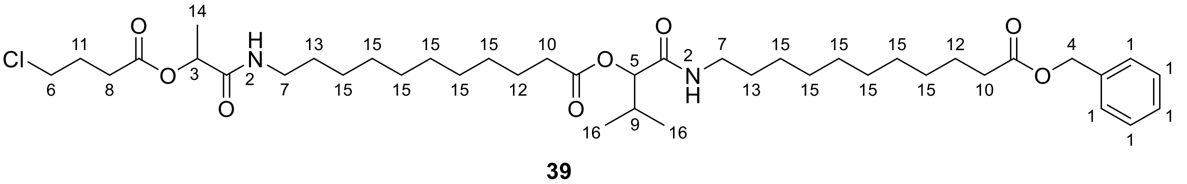

39

In a 25 mL round bottom flask, 263 mg of **38** (695 µmol, 1.00 eq.) was dissolved in 3.00 mL dichloromethane (DCM) and 37.6 µL isobutyraldehyde **14c** (30.1 mg, 417 µmol, 0.60 eq.) and 126 mg of monomer **M1** (417 µmol, 0.60 eq.) were added. The mixture was stirred at room temperature for 24 hours and subsequently the solvent was removed under reduced pressure. The residue was adsorbed onto celite® and purified *via* column chromatography on silica gel eluting with a gradual solvent mixture

of ethyl acetate and cyclohexane (4:1 → 2:1) to yield the passerini product **39** with 73.9% (232 mg, 514 µmol).

R_f = 0.64 in cyclohexane / ethyl acetate (1:1).

IR (ATR): ν / cm^{-1} = 3304.8 (vw), 2926.0 (m), 2854.2 (m), 1737.0 (vs), 1656.0 (s), 1537.2 (m), 1455.2 (w), 1371.1 (w), 1166.4 (s), 1098.9 (m), 1034.3 (w), 734.6 (w), 697.3 (m), 646.2 (vw).

^{1}H-NMR (500 MHz, $CDCl_3$): δ / ppm = 7.35 – 7.22 (m, 5 H, CH_{Ar}^1), 6.13 – 6.00 (m, 1 H, NH^2), 5.96 – 5.79 (m, 1 H, NH^2), 5.14 (q, J = 6.8 Hz, 1 H, CH^3), 5.04 (s, 2 H, CH_2^4), 4.98 (d, J = 4.4 Hz, 1 H, CH^5), 3.69 – 3.46 (m, 2 H, CH_2^6), 3.28 – 3.07 (m, 4 H, CH_2^7), 2.58 – 2.43 (m, 2 H, CH_2^8), 2.41 – 2.16 (m, 5 H, CH^9, CH_2^{10}), 2.11 – 1.97 (m, 2 H, CH_2^{11}), 1.65 – 1.46 (m, 4 H, CH_2^{12}), 1.49 – 1.37 (m, 7 H, CH_2^{13}, CH_3^{14}), 1.32 – 1.08 (m, 4 H, CH_2^{15}), 0.87 (t, J = 6.5 Hz, 6 H, CH_3^{16}).

^{13}C NMR (101 MHz, $CDCl_3$): δ / ppm = 173.83, 172.68, 171.39, 170.24, 169.40, 136.27, 128.68, 128.29, 78.06, 70.97, 66.20, 44.08, 39.40, 39.30, 34.46, 34.43, 31.32, 30.81, 30.65, 29.98, 29.71, 29.64, 29.56, 29.47, 29.33, 29.24, 27.49, 26.97, 26.94, 25.89, 25.14, 25.07, 24.09, 23.98, 18.92, 18.10, 17.09.

ESI-MS [*m/z*]: $[M + H]^+$ calculated for $^{12}C_{41}{}^{1}H_{67}{}^{16}O_8{}^{14}N_2{}^{35}Cl$, 751.4659; found, 751.4636, Δ = 2.3 mmu.

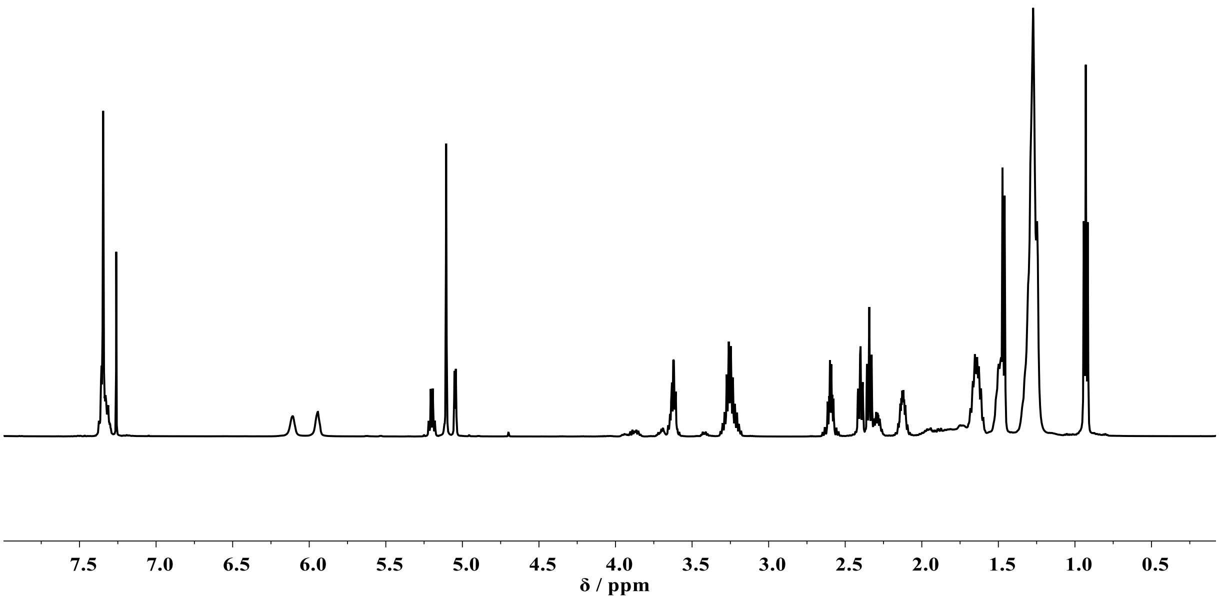

Supplementary Figure 43: ^{1}H-NMR of compound 39 measured in $CDCl_3$.

Deprotection

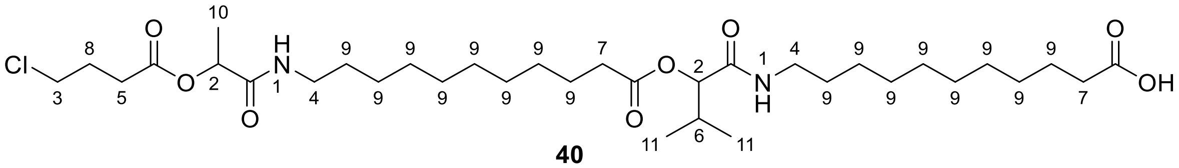

40

In a 25 mL round bottom flask, 186 mg of **39** (247 µmol, 1.00 eq.) was dissolved in 2.00 mL ethyl acetate and 2.00 mL THF. Subsequently, 37.2 mg (20 wt.-%) palladium on activated charcoal **16** was added to the solution. The resulting mixture was purged with hydrogen gas and stirred for 2 days at room temperature under hydrogen atmosphere (balloon). The crude reaction mixture was filtered over celite®. After evaporation of the solvents and drying under reduced pressure, the corresponding acid **40** was obtained in a yield of 94.8% (155 mg, 234 µmol).

IR (ATR): ν / cm^{-1} = 3311.0 (vw), 2925.9 (w), 2854.2 (vw), 2123.5 (vw), 2050.5 (vw), 2031.8 (vw), 2012.5 (vw), 1984.6 (vw), 1738.9 (w), 1652.2 (w), 1541.2 (vw), 1462.4 (vw), 1371.6 (vw), 1171.43 (vw), 1145.1 (vw), 1099.1 (vw), 921.6 (vw), 723.7 (vw), 649.7 (vw), 451.9 (vw), 405.9 (vw).

^{1}H-NMR (300 MHz, $CDCl_3$): δ / ppm = 6.19 – 6.08 (m, 1 H, NH^{1}), 6.01 – 5.85 (m, 1 H, NH^{1}), 5.21 – 4.96 (m, 2 H, CH^{2}), 3.70 – 3.51 (m, 2 H, $CH_2{}^{3}$), 3.42 – 3.08 (m, 4 H, $CH_2{}^{4}$), 2.65 – 2.43 (m, 2 H, $CH_2{}^{5}$), 2.40 – 2.16 (m, 5 H, CH^{6}, $CH_2{}^{7}$), 2.14 – 2.00 (m, 2 H, $CH_2{}^{8}$), 1.93 – 1.31 (m, 13 H, $CH_2{}^{9}$, $CH_3{}^{10}$), 1.32 – 1.08 (m, 22 H, $CH_2{}^{9}$), 0.87 (dd, J = 6.8, 4.1 Hz, 6 H, $CH_3{}^{11}$).

^{13}C NMR (101 MHz, $CDCl_3$): δ / ppm =177.46, 172.72, 171.45, 170.44, 169.51, 78.06, 70.94, 44.09, 39.46, 39.27, 34.43, 31.30, 30.61, 29.61, 29.59, 29.57, 29.40, 29.35, 29.31, 29.26, 29.19, 27.46, 26.93, 26.88, 25.15, 24.85, 18.92, 18.09, 17.08.

ESI-MS [*m/z*]: $[M + H]^+$ calculated for $^{12}C_{34}{}^{1}H_{61}{}^{16}O_8{}^{14}N_2{}^{35}Cl$; 661.4189 found, 661.4197, Δ = 0.8 mmu.

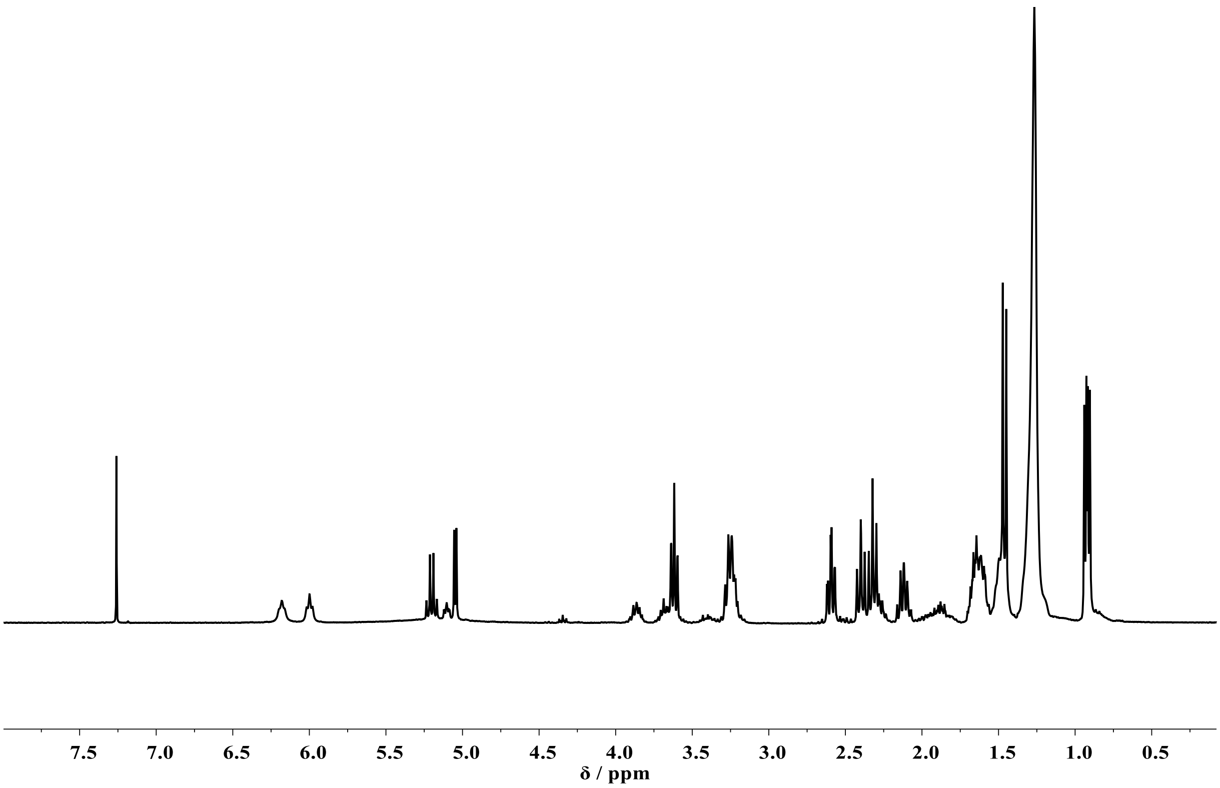

Supplementary Figure 44: ^{1}H-NMR of compound 40 measured in $CDCl_3$.

Passerini reaction

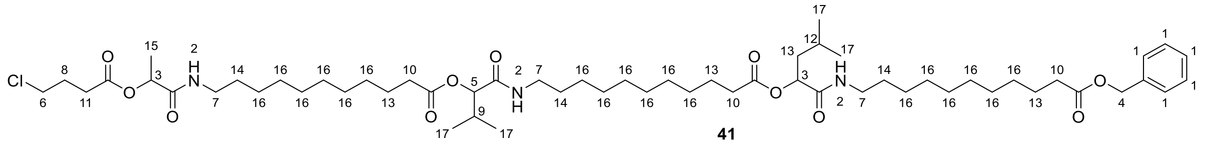

41

In a 25 mL round bottom flask, 117 mg of **40** (177 µmol, 1.00 eq.) was dissolved in 2.00 mL DCM and 28.5 µL 3-methylbutanal **14g** (22.8 mg, 265 µmol, 1.50 eq.) and 79.9 mg of monomer **M1** (265 µmol, 1.50 eq.) were added. The mixture was stirred at room temperature for 1 day and subsequently the solvent was removed under reduced pressure. The residue was adsorbed onto celite® and purified *via* column chromatography on silica gel eluting with a gradual solvent mixture of ethyl acetate and cyclohexane (3:1 → 1:1) to yield the passerini product **41** with 64.1% (119 mg, 113 µmol).

R_f = 0.64 in cyclohexane / ethyl acetate (1:1).

IR (ATR): ν / cm^{-1} = 3292.6 (vw), 2925.4 (w), 2853.9 (vw), 1737.8 (w), 1655.1 (w), 1536.4 (w), 1456.2 (vw), 1370.4 (vw), 1164.8 (w), 1100.7 (vw), 733.0 (vw), 697.6 (vw), 650.2 (vw), 463.2 (vw).

^{1}H-NMR (400 MHz, $CDCl_3$): δ / ppm = 7.42 – 7.29 (m, 5 H, $CH_{Ar}{}^{1}$), 6.11 (s, 1 H, NH^{2}), 5.96 (d, J = 6.3 Hz, 2 H, NH^{2}), 5.26 – 5.16 (m, 2 H, CH^{3}), 5.11 (s, 2 H, $CH_2{}^{4}$), 5.05 (d, J = 4.4 Hz, 1 H, CH^{5}), 3.63 (td, J = 6.2, 1.6 Hz, 2 H, $CH_2{}^{6}$), 3.32 – 3.15 (m, 6 H, $CH_2{}^{7}$), 2.60 (td, J = 7.1, 3.0 Hz, 2 H, $CH_2{}^{8}$), 2.47 – 2.23

(m, 7 H, CH^9, CH_2^{10}), 2.21 – 2.08 (m, 2 H, CH_2^{11}), 1.86 – 1.59 (m, 9 H, CH^{12}, CH_2^{13}), 1.55 – 1.42 (m, 9 H, CH_2^{14}, CH_3^{15}), 1.36 – 1.15 (m, 36 H, CH_2^{16}), 1.07 – 0.57 (m, 12 H, CH_3^{17}).

^{13}C NMR (101 MHz, $CDCl_3$): δ / ppm = 173.83, 172.78, 172.70, 171.40, 170.34, 170.24, 169.42, 136.26, 128.69, 128.30, 78.06, 72.78, 70.97, 66.21, 44.09, 41.00, 39.41, 39.37, 39.29, 34.46, 34.44, 31.32, 30.66, 29.73, 29.66, 29.58, 29.49, 29.35, 29.34, 29.25, 27.49, 26.97, 26.95, 25.15, 25.08, 24.69, 23.28, 21.94, 18.93, 18.12, 17.10.

ESI-MS [*m/z*]: [M + H]$^+$ calculated for $^{12}C_{58}{}^{1}H_{98}{}^{16}O_{11}{}^{14}N_3{}^{35}Cl$, 1048.6963; found, 1048.6944, Δ = 1.9 mmu.

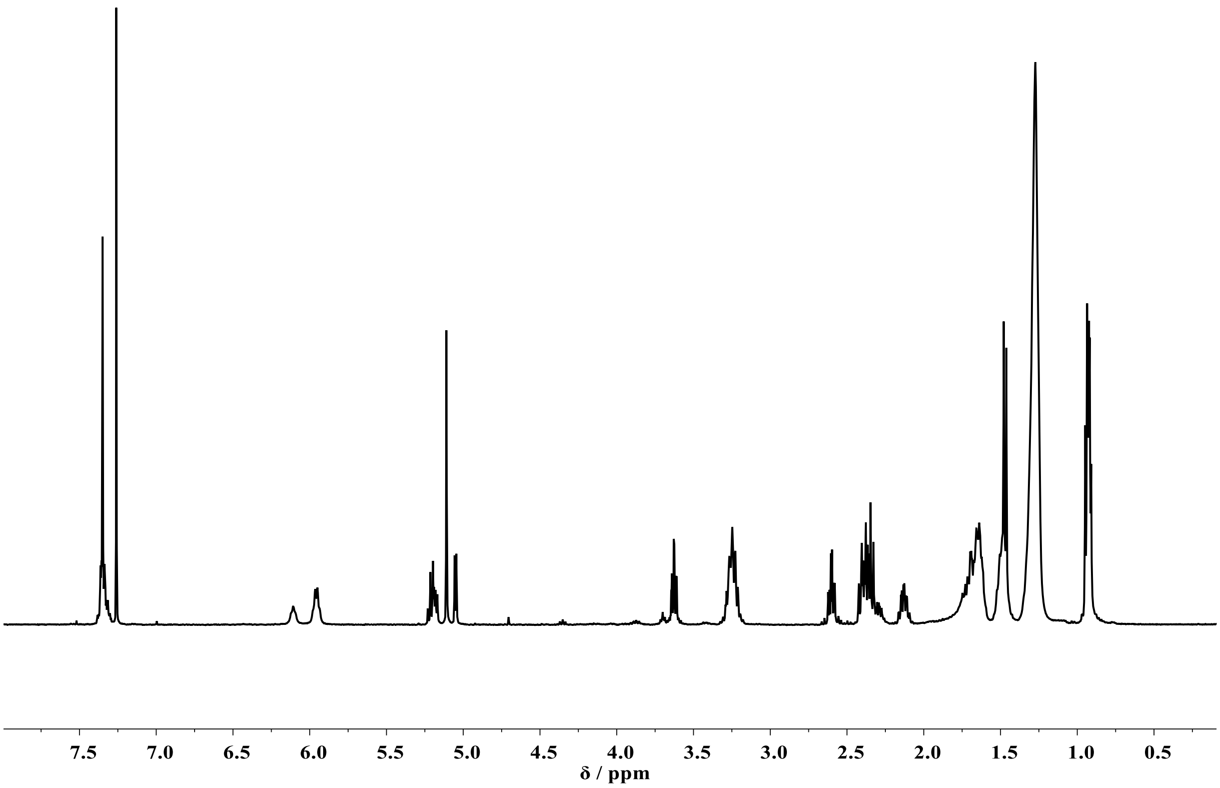

Supplementary Figure 45: ^{1}H-NMR of compound 41 measured in $CDCl_3$.

Deprotection

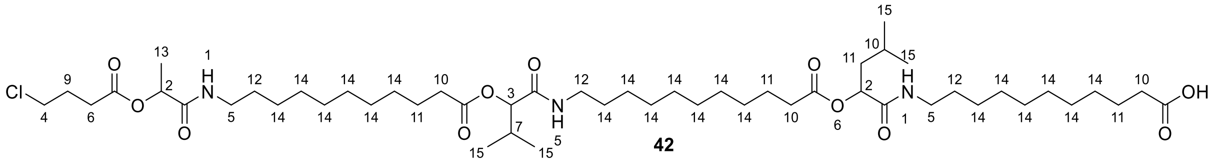

42

In a 25 mL round bottom flask, 88.9 mg of **41** (85.0 µmol, 1.00 eq.) was dissolved in 2.00 mL ethyl acetate and 2.00 mL THF. Subsequently, 17.8 mg (20 wt.-%) palladium on activated charcoal **16** was added to the solution. The resulting mixture was purged with hydrogen gas and stirred for 2 days at room temperature under hydrogen atmosphere (balloon). The crude reaction mixture was filtered over celite®. After evaporation of the solvents and drying under reduced pressure the product **42** was obtained in a yield of 98.2% (79.8 mg, 83.0 µmol).

IR (ATR): ν / cm^{-1} = 3306.7 (vw), 2925.3 (m), 2854.0 (w), 1739.2 (m), 1653.2 (m), 1539.6 (w), 1463.9 (w), 1370.3 (w), 1168.9 (m), 722.3 (vw), 650.7 (vw), 426.7 (vw).

^{1}H-NMR (400 MHz, $CDCl_3$): δ / ppm = 6.25 – 6.11 (m, 1 H, NH^{1}), 6.09 – 5.98 (m, 2 H, NH^{1}), 5.26 – 5.14 (m, 2 H, CH^{2}), 5.04 (d, *J* = 4.5 Hz, 1 H, CH^{3}), 3.62 (td, *J* = 6.4, 1.6 Hz, 2 H, $CH_2{}^{4}$), 3.32 – 3.17 (m, *J* = 6.1 Hz, 6 H, $CH_2{}^{5}$), 2.59 (td, *J* = 7.1, 3.2 Hz, 2 H, $CH_2{}^{6}$), 2.44 – 2.23 (m, 7 H, CH^{7}, $CH_2{}^{8}$), 2.19 – 2.06 (m, 2 H, $CH_2{}^{9}$), 1.74 – 1.56 (m, 9 H, CH^{10}, $CH_2{}^{11}$), 1.54 – 1.40 (m, 9 H, $CH_2{}^{12}$, $CH_3{}^{13}$), 1.39 – 1.12 (m, 36 H, $CH_2{}^{14}$), 1.01 – 0.85 (m, 12 H, $CH_3{}^{15}$).

^{13}C NMR (101 MHz, $CDCl_3$): δ / ppm = 177.43, 172.82, 171.42, 170.45, 170.38, 169.59, 78.07, 72.79, 70.93, 44.08, 40.94, 39.43, 39.34, 34.42, 33.98, 31.30, 30.62, 29.68, 29.61, 29.57, 29.53, 29.48, 29.45, 29.34, 29.31, 29.22, 29.09, 27.48, 26.96, 26.92, 26.86, 25.11, 25.07, 24.87, 24.68, 23.25, 21.92, 18.89, 18.09, 17.11.

ESI-MS [*m/z*]: $[M + H]^+$ calculated for $^{12}C_{51}{}^{1}H_{92}{}^{16}O_{11}{}^{14}N_3{}^{35}Cl$, 958.6493; found, 958.6476, Δ = 1.7 mmu.

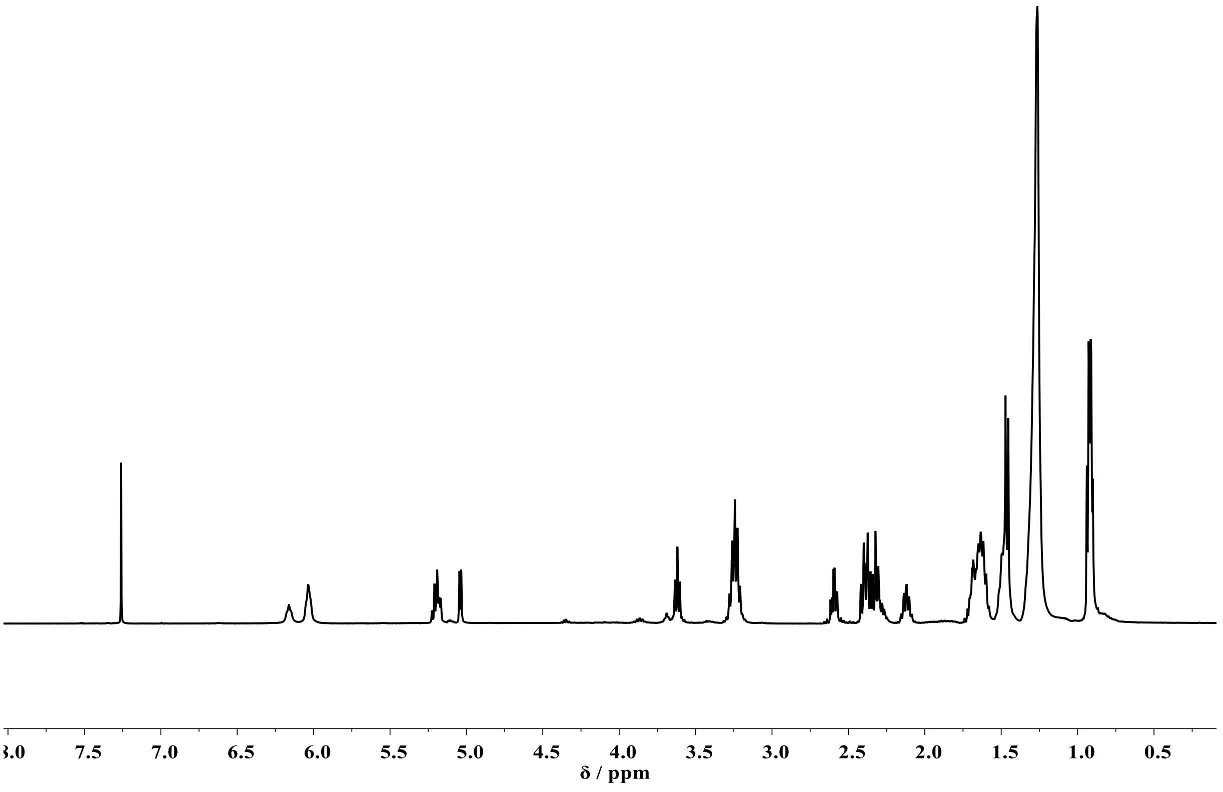

Supplementary Figure 46: ^{1}H-NMR of compound 42 measured in $CDCl_3$.

Passerini reaction

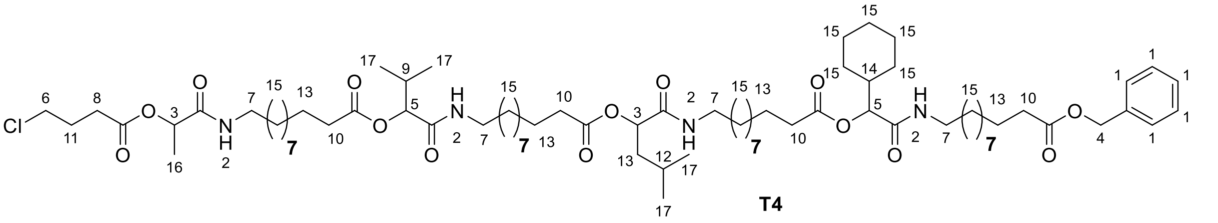

In a 25 mL round bottom flask, 51.6 mg of **42** (54.0 µmol, 1.00 eq.) was dissolved in 2.00 mL DCM and 9.10 mg cyclohexancarboxaldehyde **14j** (81.0 µmol, 1.50 eq.) and 24.3 mg of monomer **M1** (81.0 µmol, 1.50 eq.) were added. The mixture was stirred at room temperature for 1 day and subsequently the solvent was removed under reduced pressure. The residue was adsorbed onto celite® and purified *via* column chromatography on silica gel eluting with a gradual solvent mixture of ethyl acetate and cyclohexane (2:1 → 0:1) to yield the passerini product **T4** with 88.5% (65.4 mg, 48 µmol). R_f = 0.76 in cyclohexane / ethyl acetate (1:2).

IR (ATR): ν / cm^{-1} = 3299.7 (vw), 2924.9 (w), 2853.4 (w), 1738.5 (w), 1654.9 (w), 1536.8 (w), 1453.9 (vw), 1370.5 (vw), 1165.7 (w), 1101.3 (vw), 722.8 (vw), 697.9 (vw), 488.6 (vw), 461.0 (vw), 417.6 (vw).

^{1}H NMR (500 MHz, $CDCl_3$): δ / ppm = 7.40 – 7.28 (m, 5 H, CH_{Ar}^{1}), 6.15 (t, J = 6.0 Hz, 1 H, NH^{2}), 6.06 – 5.90 (m, 3 H, NH^{2}), 5.25 – 5.13 (m, 2 H, CH^{3}), 5.10 (s, 2 H, CH_2^{4}), 5.05 – 4.98 (m, 2 H, CH^{5}), 3.67 – 3.57 (m, 2 H, CH_2^{6}), 3.35 – 3.14 (m, 8 H, CH_2^{7}), 2.66 – 2.53 (m, 2 H, CH_2^{8}), 2.44 – 2.25 (m, 9 H, CH^{9}, CH_2^{10}), 2.16 – 2.06 (m, 2 H, CH_2^{11}), 2.01 – 1.90 (m, 1 H, CH^{12}), 1.84 – 1.58 (m, 18 H, CH_2^{13}), 1.56 – 1.38 (m, 12 H, CH^{14}, CH_2^{15}, CH_3^{16}), 1.35 – 1.05 (m, 50 H, CH_2^{15}), 0.98 – 0.88 (m, 12 H, CH_3^{17}).
^{13}C NMR (101 MHz, $CDCl_3$): δ / ppm = 173.73, 172.72, 172.64, 171.35, 170.31, 170.21, 169.35, 169.28, 136.16, 135.86, 128.58, 128.20, 77.96, 77.66, 72.66, 70.82, 66.11, 44.01, 40.91, 40.03, 39.31, 39.27, 39.20, 34.36, 34.32, 31.22, 30.56, 30.37, 29.61, 29.49, 29.39, 29.24, 29.15, 27.42, 27.35, 26.87, 26.12, 26.05, 25.93, 25.66, 25.04, 24.98, 24.59, 23.19, 21.84, 18.84, 18.02, 17.03.
ESI-MS [*m/z*]: $[M + H]^+$ calculated for $^{12}C_{77}{}^{1}H_{131}{}^{16}O_{14}{}^{14}N_4{}^{35}Cl$, 1371.9423; found, 1371.9403, Δ = 2.0 mmu.

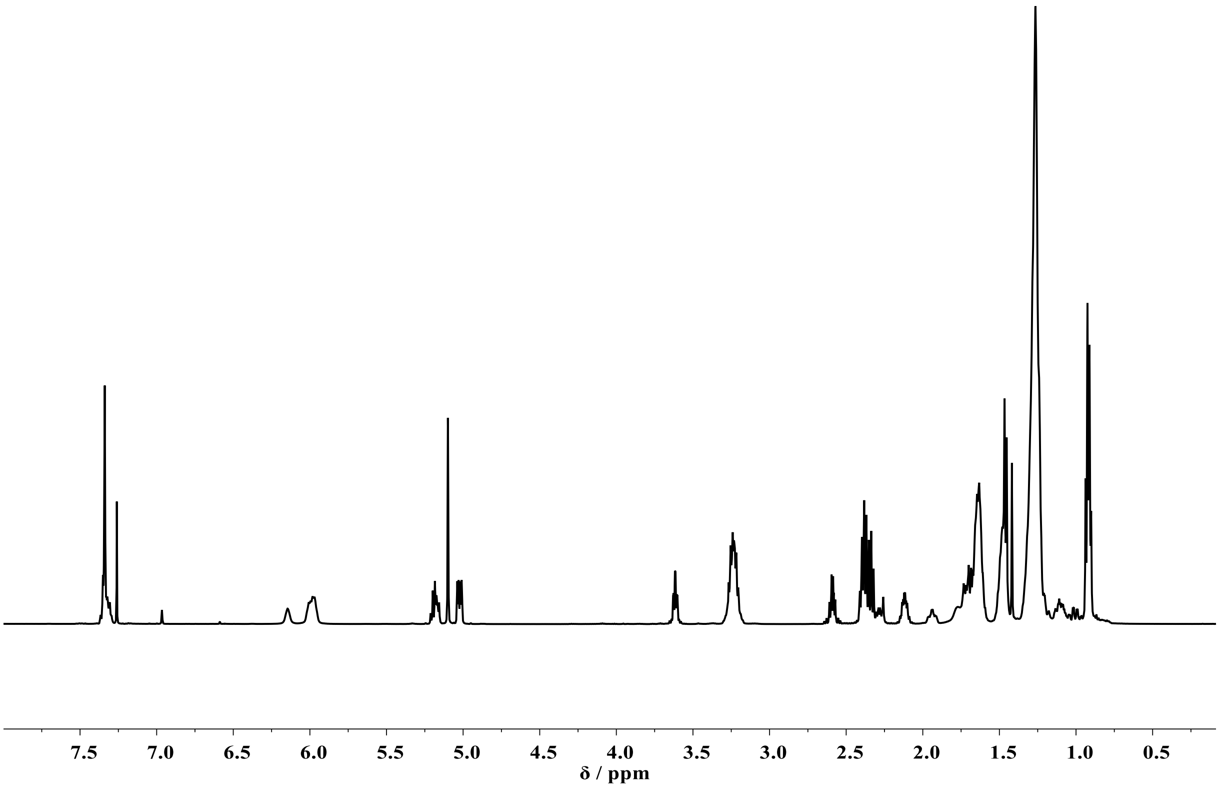

Supplementary Figure 47: ^{1}H-NMR of compound T4 measured in $CDCl_3$.

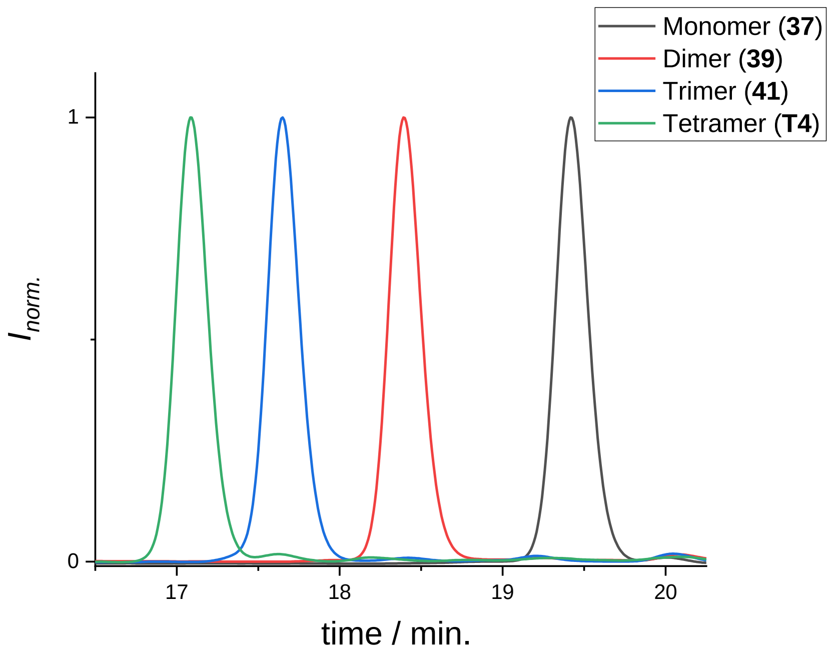

Supplementary Figure 48: SEC traces of the intermediates after each P3CR in the synthesis of product T4.

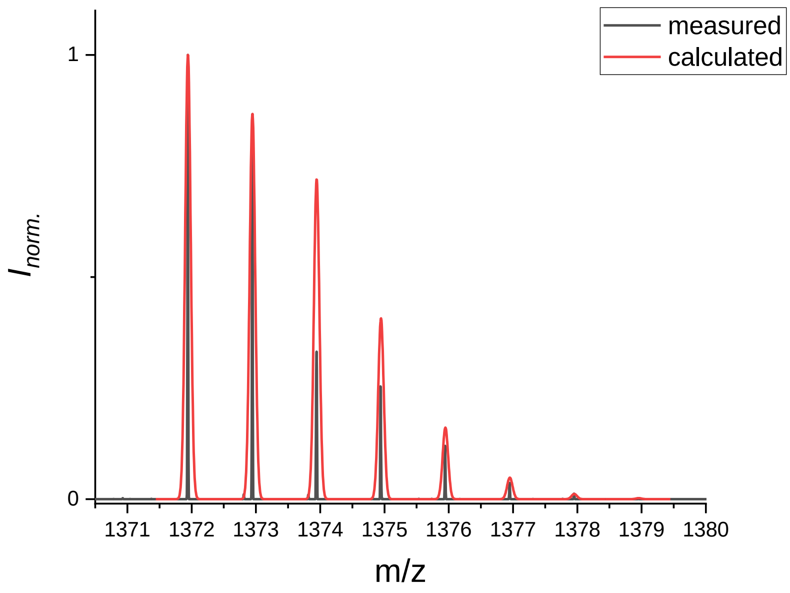

Supplementary Figure 49: High resolution ESI-MS measurement of T4. The observed isotopic pattern is compared with the calculated isotopic pattern obtained from mMass (red).

```
ximum is 1.000000 found for mass 1371.940480
matching mass 1371.94048
cutoff 0.50000: 0 solutions (7 peaks)
cutoff 0.25000: 0 solutions (27 peaks)
cutoff 0.12500: 0 solutions (60 peaks)
cutoff 0.06250: 0 solutions (105 peaks)
cutoff 0.03125: 0 solutions (175 peaks)
cutoff 0.01562: 0 solutions (281 peaks)
cutoff 0.00781: 1 solutions (454 peaks)
1371.94048 ≈ 105.017020 + 255.183450 + 283.214750 + 297.230400 + 323.246050 + 107.049690 (sides
Acetaldehyde, Isobutyraldehyde, 3-Methylbutanal, Cyclohexancarboxaldehyde; error -0.99912)
Press ENTER to quit ...
```

Supplementary Figure 50: Screenshot of the automated read-out of T4.

1.3.4 Oligomer synthesis with TAG1

1.3.4.1 Synthesis of tetramer T5

Passerini reaction

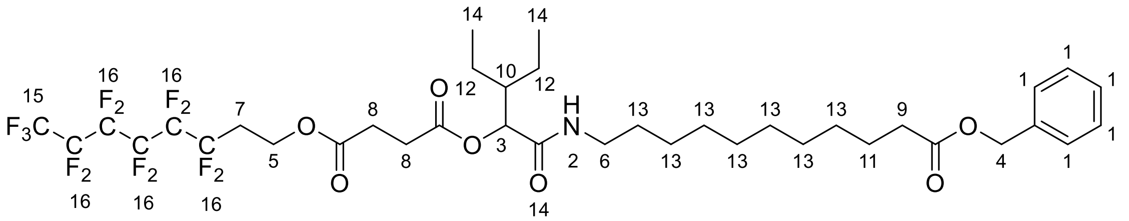

43

In a 10 mL round bottom flask, 1.20 g **TAG1** (2.59 mmol, 1.00 eq.) was stirred in 3.00 mL DCM. Subsequently, 477 µL 2-ethylbutyraldehyde **14a** (388 mg, 3.88 mmol, 1.50 eq.) and 1.17 g of the monomer **M1** (3.88 mmol, 1.50 eq.) were added. The resulting reaction mixture was stirred at room temperature for 6 days. Afterwards, the crude mixture was dried under reduced pressure. The residue was adsorbed onto celite® and purified *via* column chromatography on silica gel eluting with a gradual solvent mixture of ethyl acetate and cyclohexane (7:1 → 5:1) to yield the Passerini product **43** as a pale highly viscous oil. (2.16 g, 2.49 mmol, 96.3%).

R_f = 0.46 in cyclohexane / ethyl acetate (3:1).

IR (ATR): ν / cm^{-1} = 2929.0 (w), 2856.2 (w), 1737.2 (s), 1656.7 (m), 1533.7 (w), 1458.1 (w), 1358.2 (w), 1234.5 (vs), 1191.7 (vs), 1144.2 (vs), 1005.0 (m), 841.9 (w), 808.7 (w), 732.9 (m), 697.4 (s), 651.0 (w).

^{1}H NMR (400 MHz, $CDCl_3$): δ / ppm = 7.41 – 7.20 (m, 5 H, CH_{Ar}^{1}), 6.39 (t, J = 5.8 Hz, 1 H, NH^{2}), 5.25 (d, J = 3.1 Hz, 1 H, CH^{3}), 5.04 (s, 2 H, CH_2^{4}), 4.47 – 4.18 (m, 2 H, CH_2^{5}), 3.34 – 3.05 (m, 2 H, CH_2^{6}), 2.81 – 2.54 (m, 2 H, CH_2^{7}), 2.50 – 2.33 (m, 4 H, CH_2^{8}), 2.27 (t, J = 7.5 Hz, 2 H, CH_2^{9}), 1.85 (td, J = 5.0, 3.0 Hz, 1 H, CH^{10}), 1.61 – 1.52 (m, 2 H, CH_2^{11}), 1.50 – 1.35 (m, 4 H, CH_2^{12}), 1.29 – 1.04 (m, 14 H, CH_2^{13}), 0.85 (t, J = 7.3, 3 H, CH_3^{14}), 0.83 (t, J = 7.3, 3 H, CH_3^{14}).

^{13}C NMR (101MHz, $CDCl_3$): δ / ppm = 174.57, 173.59, 171.98, 170.36, 137.03, 129.42, 129.04, 76.56, 66.95, 57.75, 44.12, 40.25, 35.21, 31.56, 31.34, 31.12, 30.60, 30.36, 30.26, 30.25, 30.12, 30.11, 30.00, 29.96, 29.91, 27.75, 25.83, 23.32, 22.93), 12.62, 12.50.

^{19}F NMR (376 MHz, $CDCl_3$): δ / ppm = -84.73 – -85.64 (m, 3 F, CF_3^{15}), -117.60 – -119.25 (m, 2 F, CF_2^{16}), -126.03 – -126.50 (m, 2 F, CF_2^{16}), -126.75 – -127.62 (m, 2 F, CF_2^{16}), -127.46 – -128.50 (m, 2 F, CF_2^{16}), -130.04 – -131.52 (m, 2 F, CF_2^{16}). Total integral of CF_2 region normalized with respect to the CF_3^{15} group = 10.

ESI-MS [*m/z*]: $[M + Na]^+$ calculated for $^{12}C_{37}{}^{1}H_{48}{}^{16}O_7{}^{14}N^{19}F_{13}$, 888.3115; found, 888.3144, Δ = 2.9 mmu.

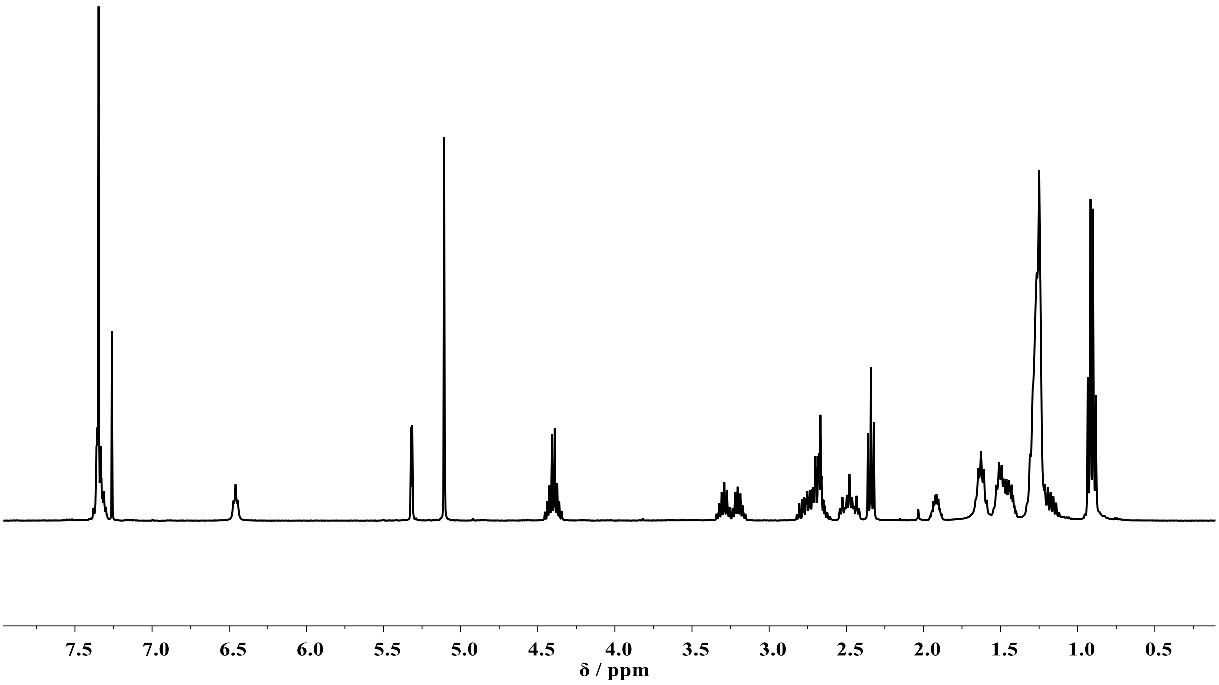

Supplementary Figure 51: ^{1}H-NMR of compound 43 measured in $CDCl_3$.

Deprotection

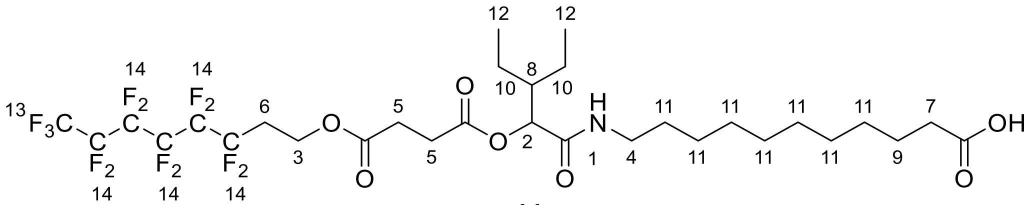

44

In a 50 mL round bottom flask, 2.02 g of **43** (2.33 mmol, 1.00 eq.) were dissolved in 3.00 mL ethyl acetate and 3.00 mL THF. Afterwards, 403 mg (20 wt%) palladium on activated charcoal **16** were added. Subsequently, the mixture was purged with hydrogen (3 balloon) and stirred under hydrogen atmosphere overnight. The heterogeneous catalyst was filtered off and the solvent was evaporated under reduced pressure. The product **44** was obtained as a pale highly viscous oil in a quantitative yield.

IR (ATR): ν / cm^{-1} = 2928.2 (m), 2856.4 (w), 1738.7 (s), 1649.6 (m), 1540.7 (w), 1461.4 (w), 1360.9 (w), 1234.0 (vs), 1192.5 (vs), 1144.2 (vs), 1082.8 (m), 1006.9 (m), 841.9 (w), 808.8 (w), 732.4 (w), 697.7 (w), 651.4 (w), 530.7 (w).

^{1}H NMR (400 MHz, $CDCl_3$): δ / ppm = 6.51 (t, J = 5.8 Hz, 1 H, NH^1), 5.32 (d, J = 3.1 Hz, 1 H, CH^2), 4.48 – 4.28 (m, 2 H, CH$_2^3$), 3.39 – 3.11 (m, 2 H, CH$_2^4$), 2.87 – 2.60 (m, 4 H, CH$_2^5$), 2.59 – 2.40 (m, 2

H, CH_2^6), 2.33 (t, *J* = 7.5 Hz, 2 H, CH_2^7), 1.97 – 1.86 (m, 1 H, CH^8), 1.72 – 1.56 (m, 2 H, CH_2^9), 1.58 – 1.38 (m, 4 H, CH_2^{10}), 1.39 – 1.14 (m, 14 H, CH_2^{11}), 0.99 – 0.79 (m, 6 H, CH_3^{12}).

^{13}C NMR (101 MHz, $CDCl_3$): *δ* / ppm = 178.81, 172.91, 171.29, 169.78, 75.76, 57.01, 43.32, 39.51, 34.01, 30.56 (t, *J* = 21.8 Hz), 29.46, 29.40, 29.34, 29.23, 29.20, 29.15, 29.06, 26.92, 24.93, 24.78, 22.15, 11.85, 11.73.

^{19}F NMR (376 MHz, $CDCl_3$): *δ* / ppm = -85.11 (t, *J* = 10.2 Hz, 3 F, CF_3^{13}), -117.83 – -118.35 (m, 3 F, CF_3^{14}), -126.02 – -126.56 (m, 2 F, CF_2^{14}), -126.73 – -127.51 (m, 2 F, CF_2^{14}), -127.64 – -128.11 (m, 2 F, CF_2^{14}), -130.37 – -130.95 (m, 2 F, CF_2^{14}). Total integral of CF_2 region normalized with respect to the CF_3^{13} group = 10.

ESI-MS [*m/z*]: $[M + H]^+$ calculated for $^{12}C_{30}{}^{1}H_{42}{}^{16}O_7{}^{14}N^{19}F_{13}$, 776.2826; found, 776.2811, Δ = 1.5 mmu.

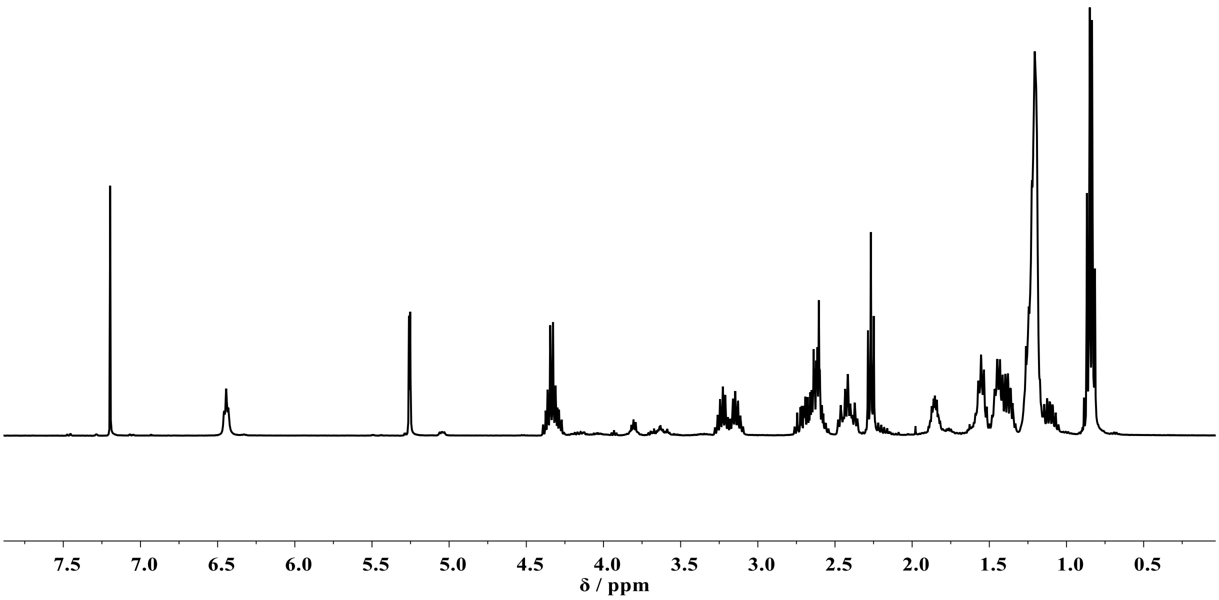

Supplementary Figure 52: ^{1}H-NMR of compound 44 measured in $CDCl_3$.

Passerini reaction

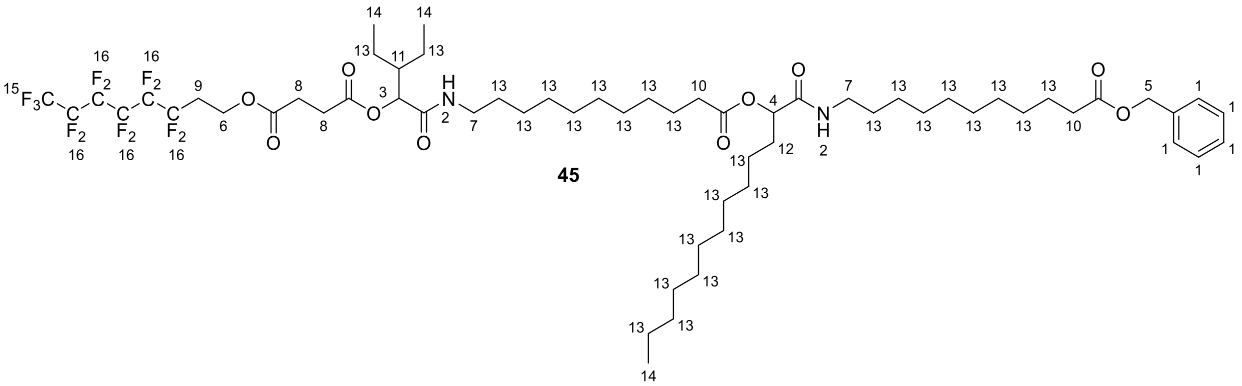

45

In a 50 mL round bottom flask, 1.20 g of **44** (1.55 mmol, 1.00 eq.) was dissolved in 3.00 mL DCM and 395 mg octanal **14i** (2.32 mmol, 1.50 eq.) and 699 mg of monomer **M1** (2.32 mmol, 1.50 eq.) were added. The mixture was stirred at room temperature for 3 days. Subsequently, the solvent was removed under reduced pressure. The crude product was purified by column chromatography (cyclohexane / ethyl acetate 6:1 →4:1) to afford product **45** as a pale highly viscous oil in a yield of 96.4% (1.88 g, 1.49 mmol).

R_f = 0.29 in cyclohexane / ethyl acetate (3:1).

IR (ATR): ν / cm^{-1} = 3296.1 (w), 2916.7 (s), 2849.1 (m), 1741.0 (vs), 1655.0 (vs), 1560.8 (w), 1468.4 (w), 1359.5 (w), 1235.4 (vs), 1204.7 (vs), 1143.0 (vs), 1083.7 (s), 1010.3 (m), 949.2 (w), 841.9 (vw), 803.4 (vw), 746.5 (w), 696.8 (vs), 652.0 (m), 566.1 (w), 528.3 (w), 439.6 (vw), 389.8 (vw).

^{1}H NMR (400 MHz, $CDCl_3$): δ / ppm = 7.48 – 7.27 (m, 5 H, $CH_{Ar}{}^{1}$), 6.47 (t, *J* = 5.8 Hz, 1 H, NH^{2}), 6.00 (t, *J* = 5.8 Hz, 1 H, NH^{2}), 5.31 (d, *J* = 3.1 Hz, 1 H, CH^{3}), 5.17 – 5.13 (m, 1 H, CH^{4}), 5.11 (s, 2 H, $CH_2{}^{5}$), 4.47 – 4.33 (m, 2 H, $CH_2{}^{6}$), 3.34 – 3.15 (m, 4 H, $CH_2{}^{7}$), 2.85 – 2.61 (m, 4 H, $CH_2{}^{8}$), 2.58 – 2.40 (m, 2 H, $CH_2{}^{9}$), 2.41 – 2.30 (m, 4 H, $CH_2{}^{10}$), 1.97 – 1.73 (m, 3 H, CH^{11}, $CH_2{}^{12}$), 1.70 – 1.58 (m, 6 H, $CH_2{}^{13}$), 1.57 – 1.38 (m, 6 H, $CH_2{}^{13}$), 1.36 – 1.10 (m, 42 H, $CH_2{}^{13}$), 0.96 – 0.80 (m, 9 H, $CH_3{}^{14}$).

^{13}C NMR (101 MHz, $CDCl_3$): δ / ppm = 173.80, 172.83, 172.57, 171.22, 169.98, 169.61, 136.27, 128.67, 128.28, 75.79, 74.07, 66.20, 56.99, 43.37, 39.47, 39.33, 34.45, 32.05, 30.59 (t, *J* = 21.9 Hz), 29.75, 29.69, 29.67, 29.62, 29.58, 29.57, 29.51, 29.49, 29.48, 29.39, 29.37, 29.35, 29.25, 29.20, 29.15, 26.99, 26.97, 25.08, 24.89, 22.82, 22.56, 22.18.

^{19}F NMR (376 MHz, $CDCl_3$): δ / ppm = -84.73 – -85.52 (m, 3 F, $CF_3{}^{15}$), -117.70 – -118.32 (m 2 F, $CF_2{}^{16}$), -125.97 – -126.42 (m 2 F, $CF_2{}^{16}$), -126.99 – -127.39 (m 2 F, $CF_2{}^{16}$), -127.77 – -128.04 (m 2 F, $CF_2{}^{16}$), -130.30 – -130.60 (m 2 F, $CF_2{}^{16}$). Total integral of CF_2 region normalized with respect to the $CF_3{}^{15}$ group = 10.

ESI-MS [*m/z*]: $[M + H]^{+}$ calculated for $^{12}C_{61}{}^{1}H_{93}{}^{16}O_{10}{}^{14}N_2{}^{19}F_{13}$, 1261.6695 found, 1261.6692, Δ = 0.3 mmu.

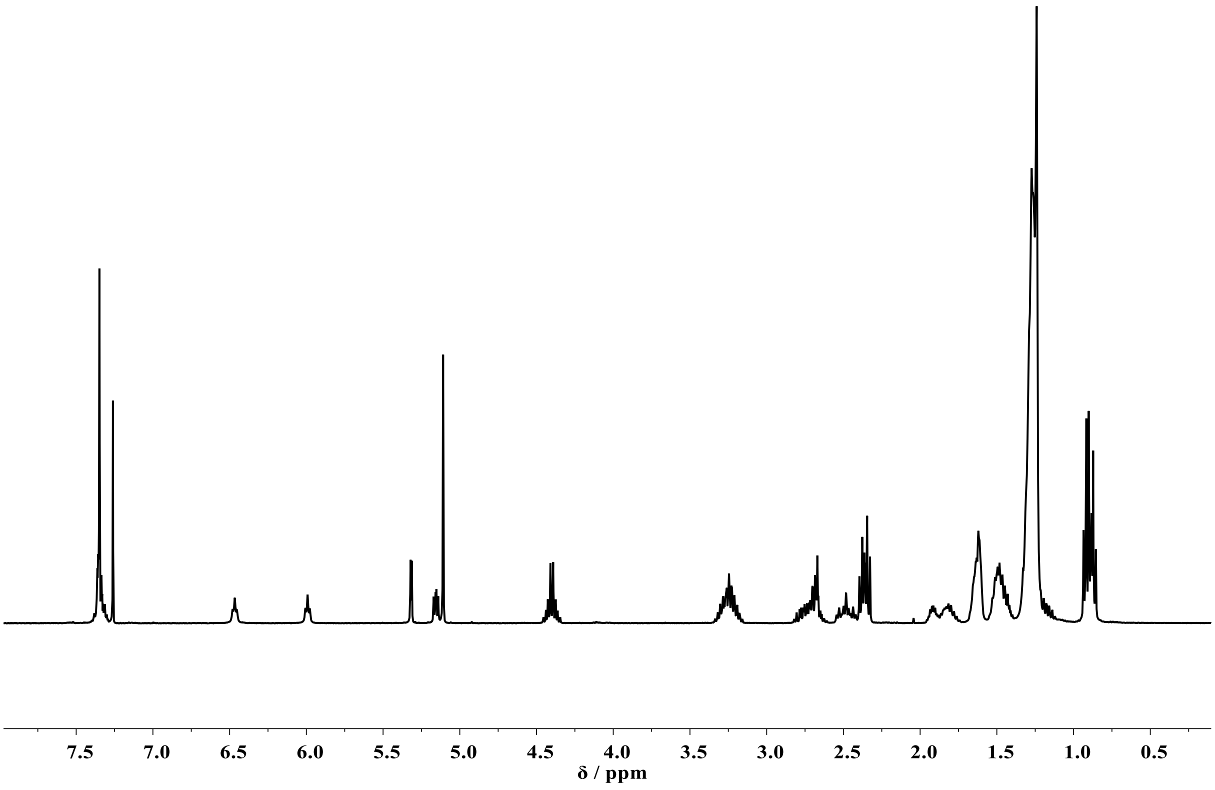

ijSupplementary Figure 53: ^{1}H-NMR of compound 45 measured in $CDCl_3$.

Deprotection

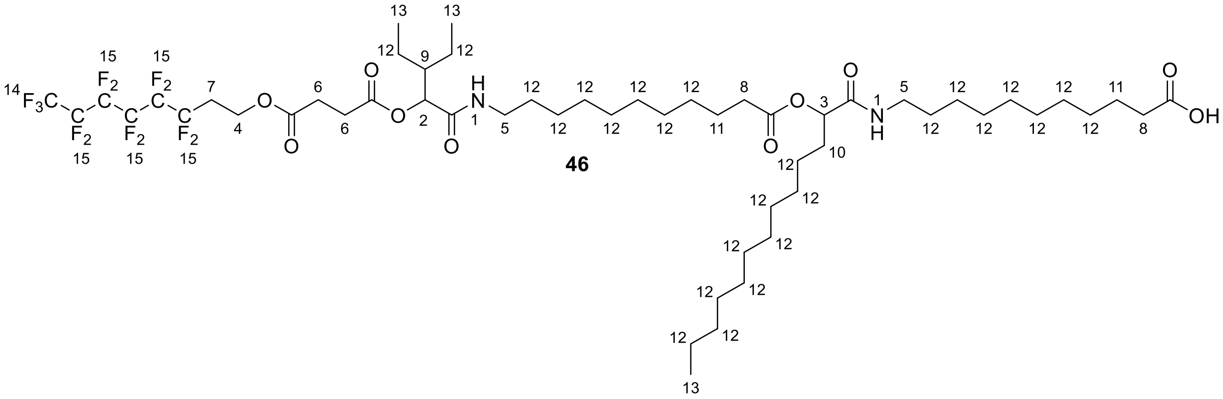

In a 50 mL round bottom flask, 1.80 g of **45** (1.43 mmol, 1.00 eq.) were dissolved in 3.00 mL ethyl acetate and 3.00 mL THF. Afterwards, 360 mg (20 wt%) palladium on activated charcoal **16** were added. Subsequently, the mixture was purged with hydrogen (3 balloons) and stirred under hydrogen atmosphere overnight. The heterogeneous catalyst was filtered off and the solvent was evaporated under reduced pressure. The product **46** was obtained as a high viscos oil in a yield of 98.6% (1.65 g, 1.41 mmol).

IR (ATR): ν / cm^{-1}] = 2924.6 (s), 2854.3 (m), 1740.3 (s), 1654.0 (m), 1539.1 (w), 1462.3 (w), 1361.4 (w), 1235.6 (vs), 1197.6 (vs), 1144.7 (vs), 1007.1 (w), 841.9 (vw), 808.5 (w), 697.6 (w), 651.5 (w).

^{1}H NMR (500 MHz, $CDCl_3$): δ / ppm = 6.54 (t, J = 5.8 Hz, 1 H, NH[1]), 6.03 (t, J = 5.8 Hz, 1 H, NH[1]), 5.32 (d, J = 3.2 Hz, 1 H, CH[2]), 5.22 – 5.12 (m, 1 H, CH[3]), 4.48 – 4.32 (m, 2 H, CH_2[4]), 3.36 – 3.14 (m, 4 H, CH_2[5]), 2.81 – 2.62 (m, 4 H, CH_2[6]), 2.55 – 2.44 (m, 2 H, CH_2[7]), 2.43 – 2.19 (m, 4 H, CH_2[8]), 1.97 – 1.75 (m, 3 H, CH[9], CH_2[10]), 1.71 – 1.58 (m, 4 H, CH_2[11]), 1.56 – 1.40 (m, 6 H, CH_2[12]), 1.37 – 1.15 (m, 44 H, CH_2[12]), 0.96 – 0.82 (m, 9 H, CH_3[13]).

^{13}C NMR (126 MHz, $CDCl_3$): δ / ppm = 177.43, 172.92, 172.61, 171.29, 170.10, 169.87, 75.76, 74.09, 57.02, 43.31, 39.56, 39.30, 34.47, 33.94, 32.05, 32.01, 30.57 (t, J = 21.8 Hz), 29.76, 29.68, 29.65, 29.57, 29.56, 29.54, 29.49, 29.44, 29.40, 29.36, 29.34, 29.28, 29.21, 29.21, 29.15, 29.08, 27.00, 26.88, 25.13, 24.90, 24.87, 22.83, 22.54, 22.16, 14.25, 11.85, 11.73.

^{19}F NMR (376 MHz, $CDCl_3$): δ / ppm =-85.09 (t, J = 9.7 Hz, 3 F, CF_3[14]), -117.79 – -118.40 (m, 2 F, CF_2[15]), -125.89 – -126.53 (m, 2 F, CF_2[15]), -126.69 – -127.45 (m, 2 F, CF_2[15]), -127.74 – -128.14 (m, 2 F, CF_2[15]), -130.00 – -130.81 (m, 2 F, CF_2[15]).Total integral of CF_2 region normalized with respect to the CF_3[14] group = 10.

ESI-MS [m/z]: $[M + H]^+$ calculated for $^{12}C_{54}{}^{1}H_{87}{}^{16}O_{10}{}^{14}N_2{}^{19}F_{13}$, 1171.6226; found, 1171.6216, Δ = 1.0mmu.

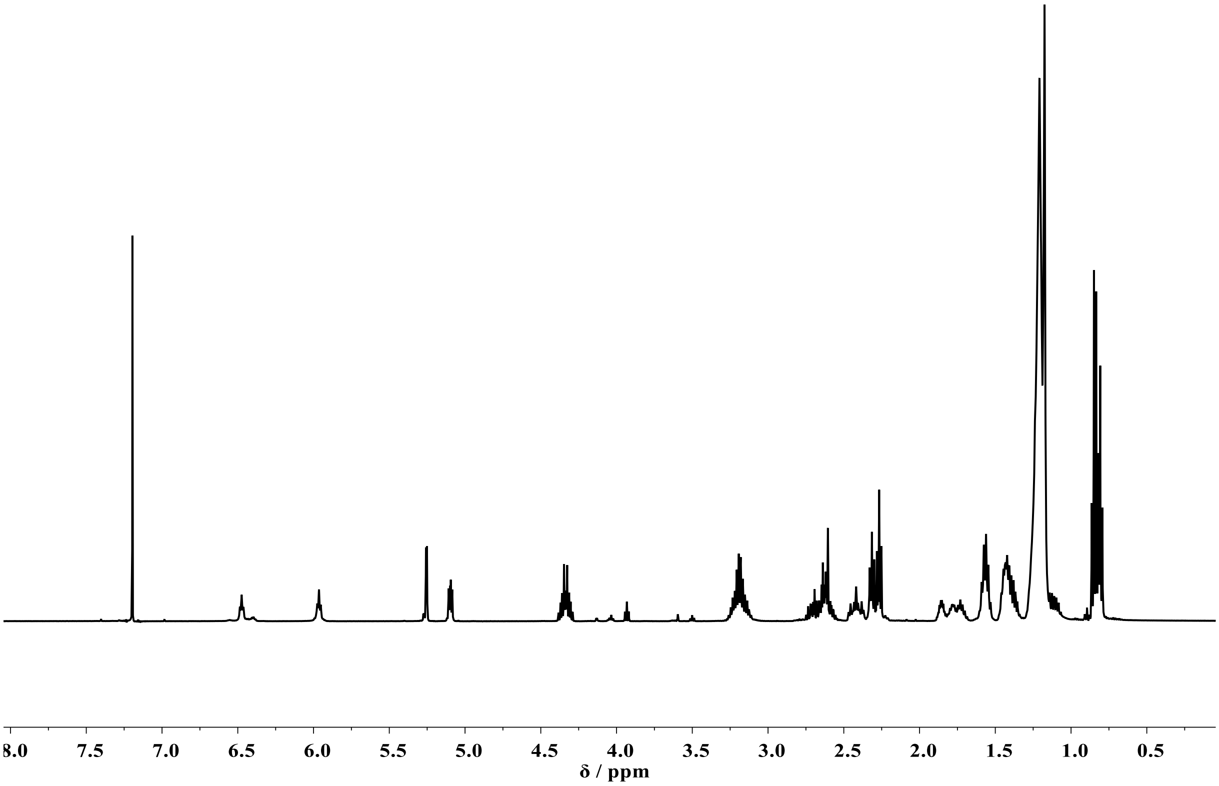

Supplementary Figure 54: ^{1}H-NMR of compound 46 measured in $CDCl_3$.

Passerini reaction

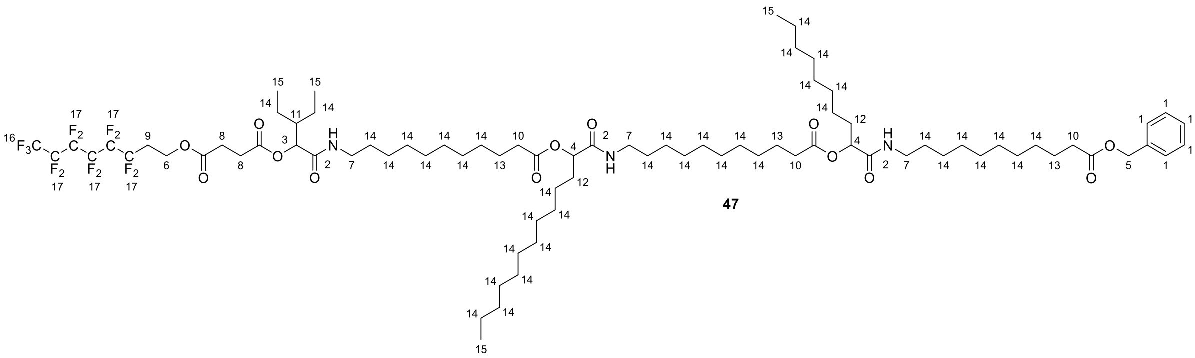

47

In a 50 mL round bottom flask, 1.58 g of passerini **46** (1.35 mmol, 1.00 eq.) was dissolved in 5.00 mL DCM and 347 µL nonanal **14k** (287 mg, 2.02 mmol, 1.50 eq.) and 608 mg of monomer **M1** (2.02 mmol, 1.50 eq.) were added. The mixture was stirred at room temperature for 2 d and subsequently the solvent was removed under reduced pressure. The crude product was purified by column chromatography (cyclohexane / ethyl acetate 5:1→ 2:1) to afford product **47** as a white solid in a yield of 91.9% (1.99 g, 1.24 mmol).

R_f = 0.26 in cyclohexane / ethyl acetate (3:1).

IR (ATR): ν / cm^{-1}] = 3300.8 (vw), 2919.0 (s), 2851.1 (m), 1737.1 (vs), 1655.0 (vs), 1541.3 (w), 1465.7 (w), 1362.8 (w), 1236.9 (s), 1204.7 (s), 1145.0 (vs), 1005.9 (w), 842.1 (vw), 809.1 (vw), 697.4 (m), 652.7 (w).

^{1}H NMR (400 MHz, $CDCl_3$): δ / ppm = 7.39 – 7.29 (m, 5 H, $CH_{Ar}{}^1$), 6.47 (t, *J* = 5.7 Hz, 1 H, NH^2), 6.07 – 5.98 (m, 2 H, NH^2), 5.31 (d, *J* = 3.1 Hz, 1 H, CH^3), 5.17 – 5.13 (m, 2 H, CH^4), 5.11 (s, 2 H, $CH_2{}^5$), 4.46 – 4.34 (m, 2 H, $CH_2{}^6$), 3.35 – 3.14 (m, 6 H, $CH_2{}^7$), 2.85 – 2.62 (m, 4 H, $CH_2{}^8$), 2.57 – 2.42 (m, 2 H, $CH_2{}^9$), 2.42 – 2.30 (m, 6 H, $CH_2{}^{10}$), 1.95 – 1.75 (m, 5 H, $CH_2{}^{11}$, CH^{12}), 1.70 – 1.58 (m, 6 H, $CH_2{}^{13}$), 1.54 – 1.40 (m, 8 H, $CH_2{}^{14}$), 1.38 – 1.15 (m, 68 H, $CH_2{}^{14}$), 0.98 – 0.79 (m, 12 H, $CH_3{}^{15}$).

^{13}C NMR (101 MHz, $CDCl_3$): δ / ppm = 173.80, 172.83, 172.59, 171.22, 170.00, 169.98, 169.62, 136.27, 128.67, 128.28, 75.79, 74.08, 66.20, 56.99, 43.38, 39.47, 39.33, 34.46, 32.05, 31.96, 30.82, 30.60, 30.38, 29.75, 29.72, 29.70, 29.67, 29.63, 29.62, 29.59, 29.57, 29.52, 29.50, 29.48, 29.39, 29.38, 29.35, 29.32, 29.26, 29.25, 29.20, 29.15, 26.99, 26.97, 25.10, 25.09, 25.08, 24.91, 24.90, 22.82, 22.78, 22.57, 22.10, 14.23, 11.87, 11.75.

^{19}F NMR (376 MHz, $CDCl_3$): δ / ppm = -84.62 – -85.83 (m, 3 F, $CF_3{}^{16}$), -117.63 – -118.90 (m, 2 F, $CF_2{}^{17}$), -126.02 – -126.52 (m, 2 F, $CF_2{}^{17}$), -126.81 – -127.48 (m, 2 F, $CF_2{}^{17}$), -127.63 – -128.33 (m, 2 F, $CF_2{}^{17}$), -130.01 – -131.02 (m, 2 F, $CF_2{}^{17}$). Total integral of CF_2 region normalized with respect to the $CF_3{}^{17}$group = 10.

ESI-MS [*m/z*]: [M + Na]$^+$ calculated for $^{12}C_{82}{}^{1}H_{132}{}^{16}O_{13}{}^{14}N_3{}^{19}F_{13}$, 1636.9445; found, 1636.9430, Δ = 1.5 mmu.

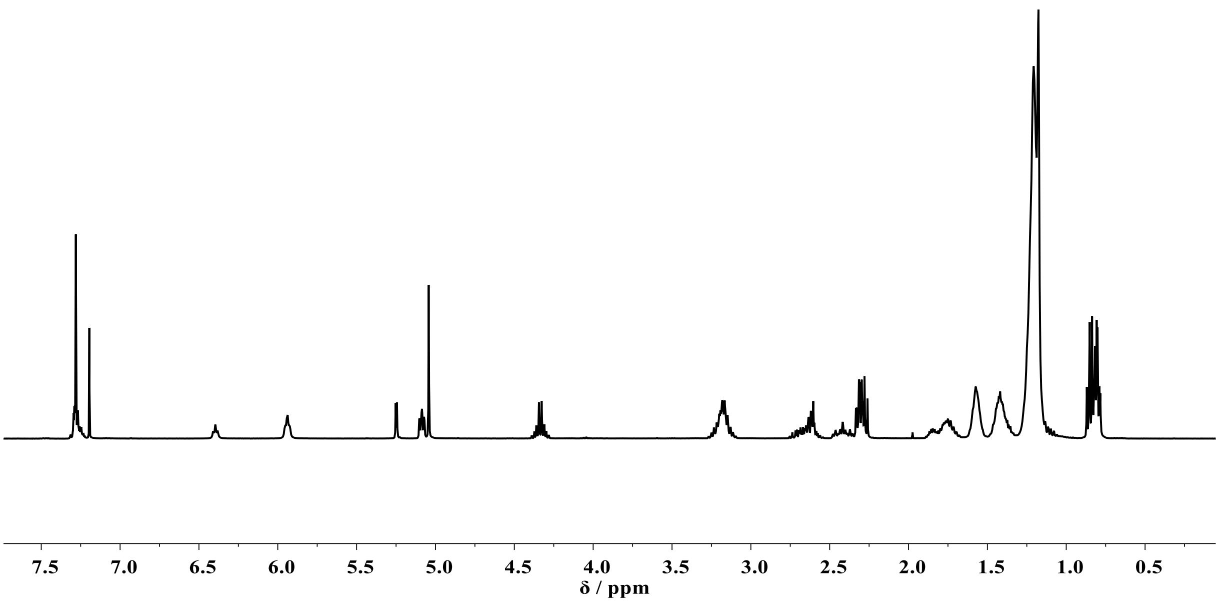

Supplementary Figure 55: ^{1}H-NMR of compound 47 measured in $CDCl_3$.

Deprotection

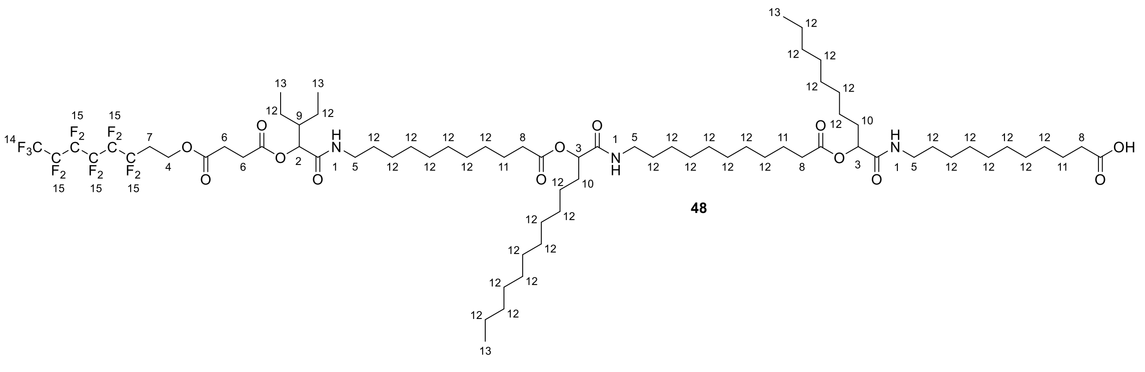

In a 50 mL round bottom flask, 1.94 g of **47** (1.20 mmol, 1.00 eq.) were dissolved in 4.00 mL ethyl acetate and 4.00 mL THF. Afterwards, 388 mg (20 wt%) palladium on activated charcoal **16** were added. Subsequently, the mixture was purged with hydrogen (3 balloons) and stirred under hydrogen atmosphere overnight. The heterogeneous catalyst was filtered off and the solvent was evaporated under reduced pressure. The product **48** was obtained as a high viscous oil in a yield of 97.5% (1.79 g, 1.17 mmol).

IR (ATR): ν / cm^{-1} = 3297.8 (vw), 2919.6 (s), 2851.4 (m), 1738.1 (s), 1655.5 (vs), 1555.1 (w), 1465.1 (w), 1364.1 (w), 1236.6 (vs), 1144.6 (vs), 1007.4 (w), 842.2 (vw), 809.3 (vw), 697.4 (w), 651.8 (w), 566.0 (vw), 530.6 (vw).

^{1}H NMR (400 MHz, $CDCl_3$): δ / ppm = 6.49 – 6.39 (m, 1 H, NH^{1}), 6.06 – 5.94 (m, 2 H, NH^{1}), 5.25 (d, J = 3.2 Hz, 1 H, CH^{2}), 5.12 – 5.04 (m, 2 H, CH^{3}), 4.43 – 4.26 (m, 2 H, CH_2^{4}), 3.28 – 3.09 (m, 6 H, CH_2^{5}), 2.79 – 2.55 (m, 4 H, CH_2^{6}), 2.51 – 2.37 (m, 2 H, CH_2^{7}), 2.36 – 2.20 (m, 6 H, CH_2^{8}), 1.92 – 1.67 (m, 5 H, CH^{9}, CH_2^{10}), 1.64 – 1.50 (m, 6 H, CH_2^{11}), 1.51 – 1.30 (m, 8 H, CH_2^{12}), 1.31 – 1.02 (m, 68H, CH_2^{12}), 0.89 – 0.76 (m, 12 H, CH_3^{13}).

^{13}C NMR (101 MHz, $CDCl_3$): δ / ppm = 176.95, 172.88, 172.70, 172.62, 171.25, 170.19, 170.08, 169.75, 75.77, 74.10, 74.07, 57.01, 43.35, 39.51, 39.40, 39.30, 34.48, 34.45, 33.96, 32.05, 32.02, 31.97, 29.76, 29.68, 29.63, 29.57, 29.56, 29.53, 29.51, 29.49, 29.43, 29.39, 29.37, 29.34, 29.28, 29.25, 29.22, 29.16, 29.09, 26.98, 26.89, 25.14, 25.08, 24.91, 22.83, 22.79, 22.56, 22.17, 14.25, 14.24, 11.87, 11.74.

^{19}F NMR (376 MHz, $CDCl_3$): δ / ppm = -84.04 – -86.23 (m, 3 F, CF_3^{14}), -117.79 – -118.50 (m, 2 F, CF_2^{15}), -125.95 – -126.50 (m, 2 F, CF_2^{15}), -127.05 – -127.38 (m, 2 F, CF_2^{15}), -127.70 – -128.23 (m, 2 F, CF_2^{15}), -130.34 – -130.71 (m, 2 F, CF_2^{15}). Total integral of CF_2 region normalized with respect to the CF_3^{14} group = 10.

ESI-MS [*m/z*]: $[M + Na]^+$ calculated for $^{12}C_{75}{}^{1}H_{126}{}^{16}O_{13}{}^{19}F_{13}{}^{14}N_3$, 1546.8975; found, 1546.8952, Δ = 2.3 mmu.

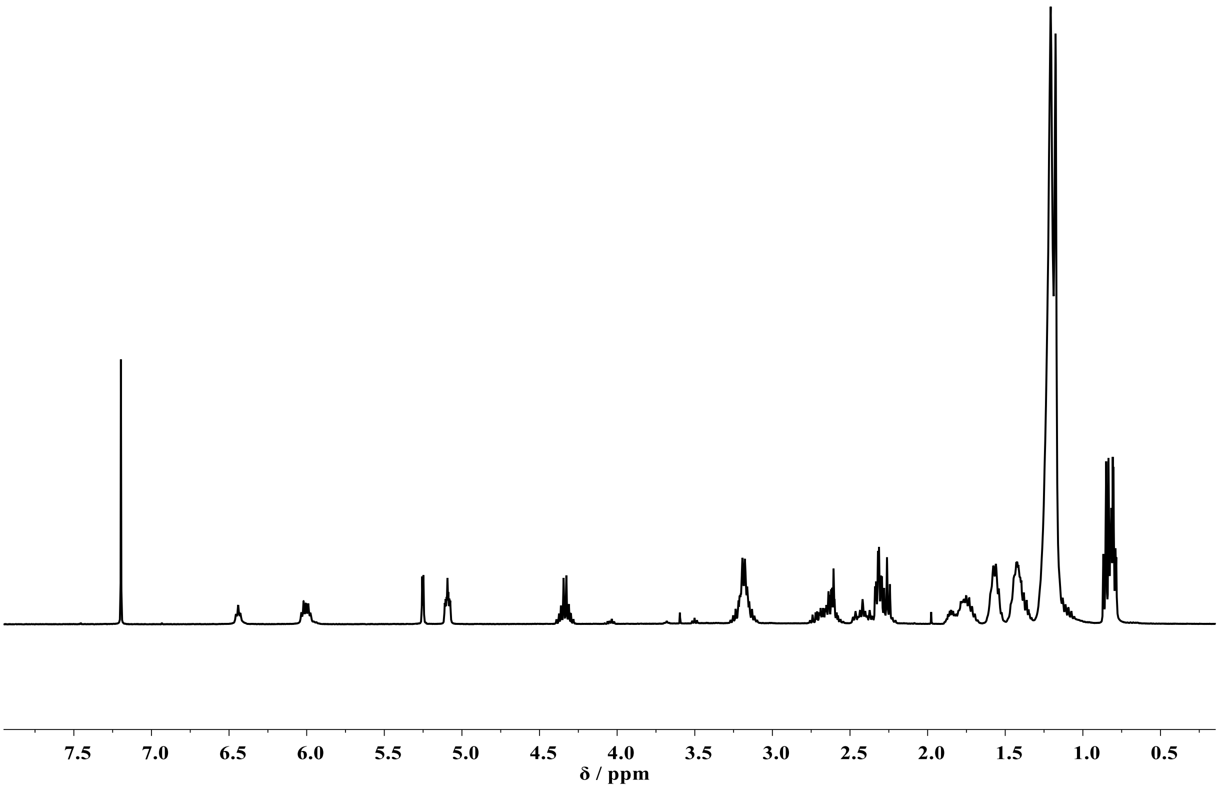

Supplementary Figure 56: ^{1}H-NMR of compound 48 measured in $CDCl_3$.

Passerini reaction

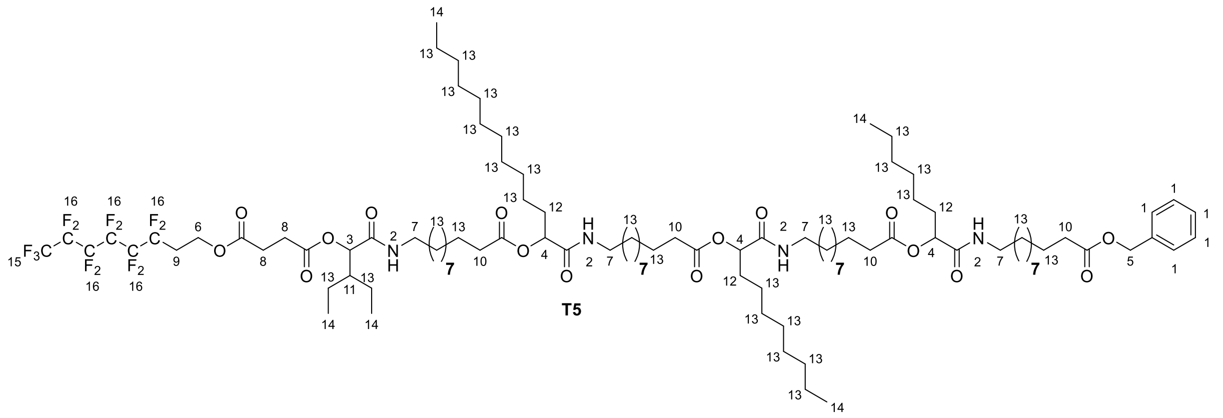

In a 50 mL round bottom flask, 1.71 g of **48** (1.12 mmol, 1.00 eq.) was dissolved in 5.00 mL DCM and 235 µL heptanal **14b** (192 mg, 1.69 mmol, 1.50 eq.) and 508 mg of monomer **M1** (1.69 mmol, 1.50 eq.) were added. The mixture was stirred at room temperature for 2 d and subsequently the solvent was removed under reduced pressure. The crude product was purified by column chromatography (cyclohexane / ethyl acetate 5:1→ 1:1) to afford product **T5** as a white solid in a yield of 94.6% (2.06 g, 1.06 mmol).

R_f = 0.21 in cyclohexane / ethyl acetate (2:1).

IR (ATR): ν / cm^{-1}] = 3304.4 (vw), 2923.3 (s), 2853.1 (m), 1738.7 (s), 1655.1 (s), 1535.6 (m), 1464.1 (w), 1362.8 (w), 1236.8 (s), 1145.3 (vs), 697.1 (w), 651.1 (w), 396.3 (vw).

^{1}H NMR (500 MHz, $CDCl_3$): δ / ppm = 7.45 – 7.28 (m, 5 H, CH_{Ar}^{1}), 6.47 (t, J = 5.8 Hz, 1 H, NH^{2}), 6.06 – 5.96 (m, 3 H, NH^{2}), 5.31 (d, J = 3.2 Hz, 1 H, CH^{3}), 5.17 – 5.13 (m, 3 H, CH^{4}), 5.10 (s, 2 H, CH_2^{5}), 4.47 – 4.33 (m, 2 H, CH_2^{6}), 3.33 – 3.14 (m, 8 H, CH_2^{7}), 2.82 – 2.60 (m, 4 H, CH_2^{8}), 2.56 – 2.41 (m, 2 H, CH_2^{9}), 2.42 – 2.30 (m, 8 H, CH_2^{10}), 1.96 – 1.74 (m, 7 H, CH^{11}, CH_2^{12}), 1.71 – 1.59 (m, 12 H, CH_2^{13}), 1.55 – 1.40 (m, 14 H, CH_2^{13}), 1.38 – 1.11 (m, 80 H, CH_2^{13}), 0.96 – 0.82 (m, 15 H, CH_3^{14}).

^{13}C NMR (126 MHz, $CDCl_3$): δ / ppm = 173.70, 172.74, 172.48, 171.11, 169.88, 169.85, 169.51, 136.13, 128.55, 128.17, 75.64, 73.93, 66.08, 56.86, 43.23, 39.33, 39.19, 34.32, 31.92, 31.83, 31.63, 30.44 (t, J = 21.8 Hz), 29.62, 29.58, 29.56, 29.54, 29.49, 29.46, 29.44, 29.39, 29.39, 29.35, 29.26, 29.22, 29.20, 29.13, 29.06, 29.01, 28.92, 26.84, 24.97, 24.96, 24.77, 24.71, 22.69, 22.65, 22.54, 22.42, 22.04, 14.12, 14.11, 14.05, 11.74, 11.62.

^{19}F NMR (376 MHz, $CDCl_3$): δ / ppm = -80.74 (t, J = 10.0 Hz, 3 F, CF_3^{15}), -113.42 – -113.95 (m, 2 F, CF_2^{16}), -121.56 – -122.14 (m, 2 F, CF_2^{16}), -122.38 – -123.21 (m, 2 F, CF_2^{16}), -123.25 – -123.72 (m, 2 F, CF_2^{16}), -125.70 – -126.66 (m, 2 F, CF_2^{16}). Total integral of CF_2 region normalized with respect to the CF_3^{16}group = 10.

ESI-MS [*m/z*]: [M + Na]$^{+}$ calculated for $^{12}C_{101}{}^{1}H_{167}{}^{19}F_{13}{}^{14}N_{4}{}^{16}O_{16}$, 1962.2062; found, 1962.2090, Δ = 2.8 mmu.

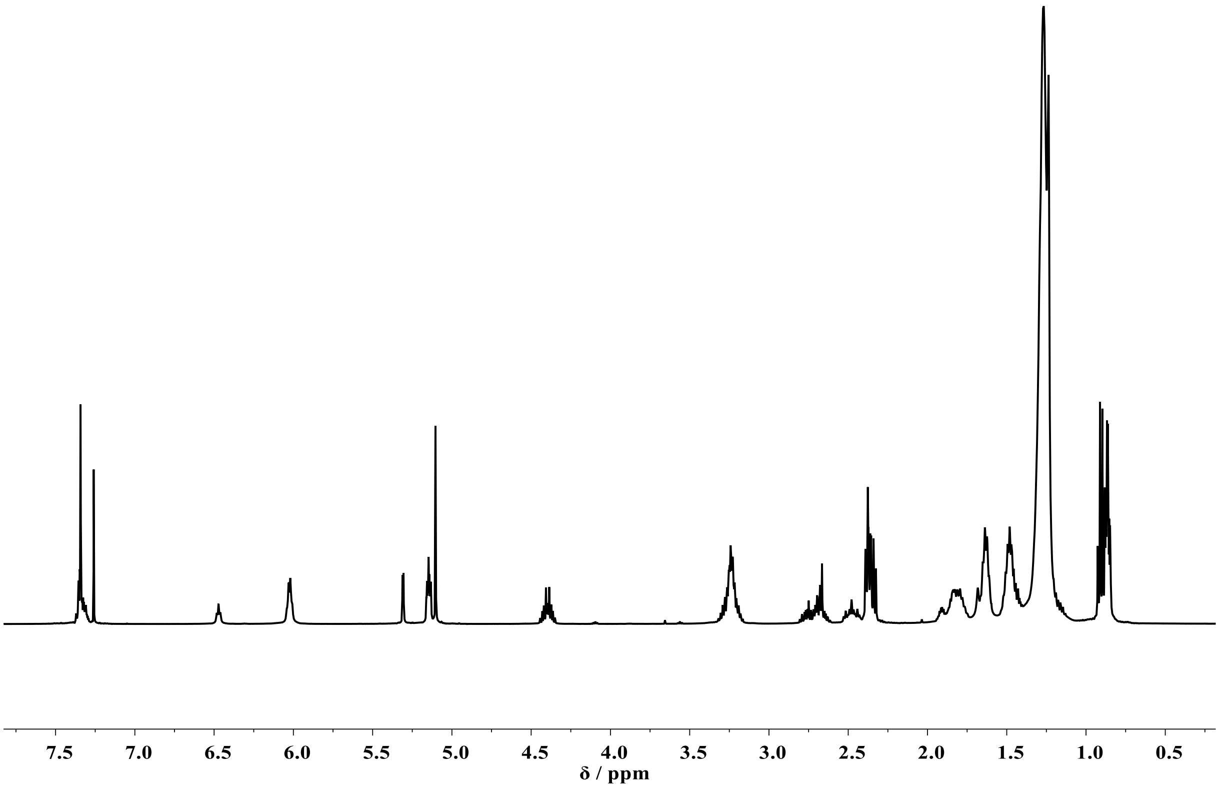

Supplementary Figure 57: ^{1}H-NMR of compound T5 measured in $CDCl_3$.

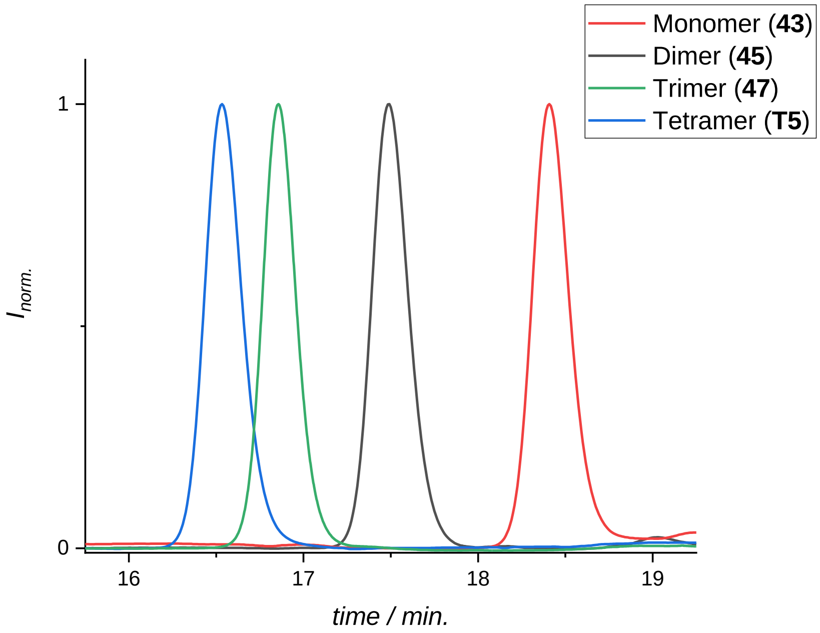

Supplementary Figure 58: SEC traces of the intermediates after each P3CR in the synthesis of product T5.

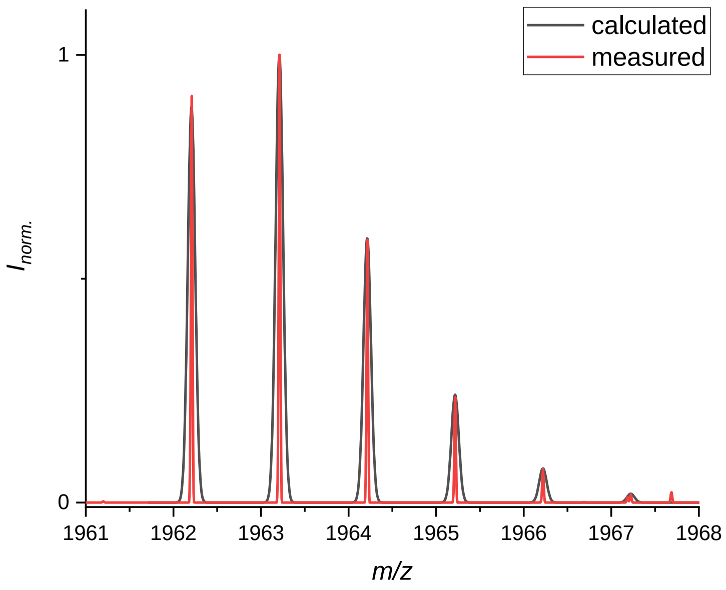

Supplementary Figure 59: High resolution ESI-MS measurement of T5. The observed isotopic pattern is compared with the calculated isotopic pattern obtained from mMass (black).

```
aximum is 1.000000 found for mass 1963.205820
matching mass 1963.20582
cutoff 0.50000: 0 solutions (12 peaks)
cutoff 0.25000: 0 solutions (51 peaks)
cutoff 0.12500: 0 solutions (109 peaks)
cutoff 0.06250: 0 solutions (179 peaks)
cutoff 0.03125: 0 solutions (262 peaks)
cutoff 0.01562: 1 solutions (354 peaks)
1963.20582 ≈ 463.021500 + 311.246050 + 395.339950 + 353.293000 + 325.261700 + 91.054780 (sides 2-Ethylbutanal, Dodecanal,
 Nonanal, Heptanal; error -23.98884)
Press ENTER to quit ...
```

Supplementary Figure 60: Screenshot of the automated read-out of T5, sodium trifluoroacetate was used as additive during the measurement.

1.3.4.2 Synthesis of hexamer H2

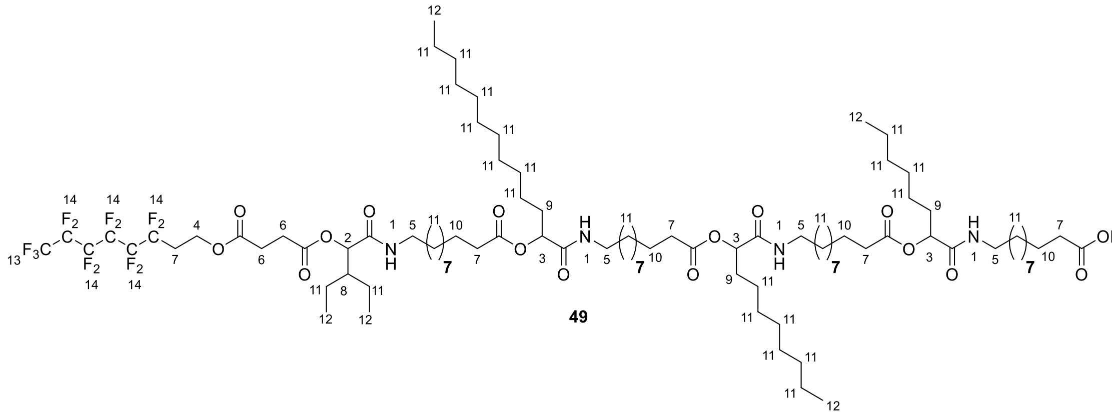

49

In a 50 mL round bottom flask, 1.45 g of **T5** (748 µmol, 1.00 eq.) were dissolved in 5.00 mL ethyl acetate and 5.00 mL THF. Afterwards, 290 mg (20 wt%) palladium on activated charcoal **16** were added. Subsequently, the mixture was purged with hydrogen (3 balloons) and stirred under hydrogen atmosphere overnight. The heterogeneous catalyst was filtered off and the solvent was evaporated under reduced pressure. The product **49** was obtained as a pale highly viscous oil in a yield of 99.5% (1.38 g, 745 µmol).

IR (ATR): ν / cm^{-1} = 3293.4 (vw), 2922.3 (vs), 2852.7 (s), 1738.6 (s), 1655.5 (vs), 1540.7 (m), 1465.3 (w), 1364.0 (w), 1236.5 (vs), 1145.1 (vs), 842.2 (vw), 697.5 (w), 651.7 (w).

^{1}H NMR (400 MHz, $CDCl_3$): δ / ppm = 6.43 (t, J = 5.8 Hz, 1 H, NH^{1}), 6.09 – 5.94 (m, 3 H, NH^{1}), 5.25 (d, J = 3.2 Hz, 1 H, CH^{2}), 5.14 – 5.05 (m, 3 H, CH^{3}), 4.43 – 4.24 (m, 2 H, CH_2^{4}), 3.30 – 3.05 (m, 8 H, CH_2^{5}), 2.78 – 2.54 (m, 4 H, CH_2^{6}), 2.51 – 2.16 (m, 10 H, CH_2^{7}), 1.91 – 1.66 (m, 7 H, CH^{8}, CH_2^{9}), 1.65 – 1.51 (m, 8 H, CH_2^{10}), 1.49 – 0.98 (m, 101 H, CH_2^{11}), 0.92 – 0.74 (m, 15 H, CH_3^{12}).

^{13}C NMR (101 MHz, $CDCl_3$): δ / ppm = 177.11, 172.86, 172.68, 172.62, 171.24, 170.17, 170.11, 170.07, 169.72, 75.75, 74.07, 74.05, 56.98, 43.34, 34.43, 33.97, 32.03, 31.95, 31.75, 30.57, 29.74, 29.67, 29.61, 29.56, 29.51, 29.48, 29.46, 29.36, 29.35, 29.24, 29.18, 29.13, 29.10, 29.03, 26.96, 26.89, 25.11, 25.08, 24.89, 24.84, 22.80, 22.77, 22.65, 22.54, 22.15, 14.23, 14.16, 11.85, 11.72.

^{19}F NMR (376 MHz, $CDCl_3$): δ / ppm = -80.74 (t, J = 9.9 Hz, 3 F, CF_3^{13}), -113.52 – -113.84 (m, 2 F, CF_2^{14}), -121.68 – -121.98 (m, 2 F, CF_2^{14}), -122.48 – -122.99 (m, 2 F, CF_2^{14}), -123.39 – -123.65 (m, 2 F, CF_2^{14}), -125.48 – -126.44 (m, 2 F, CF_2^{14}). Total integral of CF_2 region normalized with respect to the CF_3^{14} group = 10.

ESI-MS [*m/z*]: $[M + H]^{+}$ calculated for $^{12}C_{94}{}^{1}H_{161}{}^{16}O_{16}{}^{14}N_{4}{}^{19}F_{13}$, 1850.1773; found, 1850.1772, Δ = 0.1 mmu.

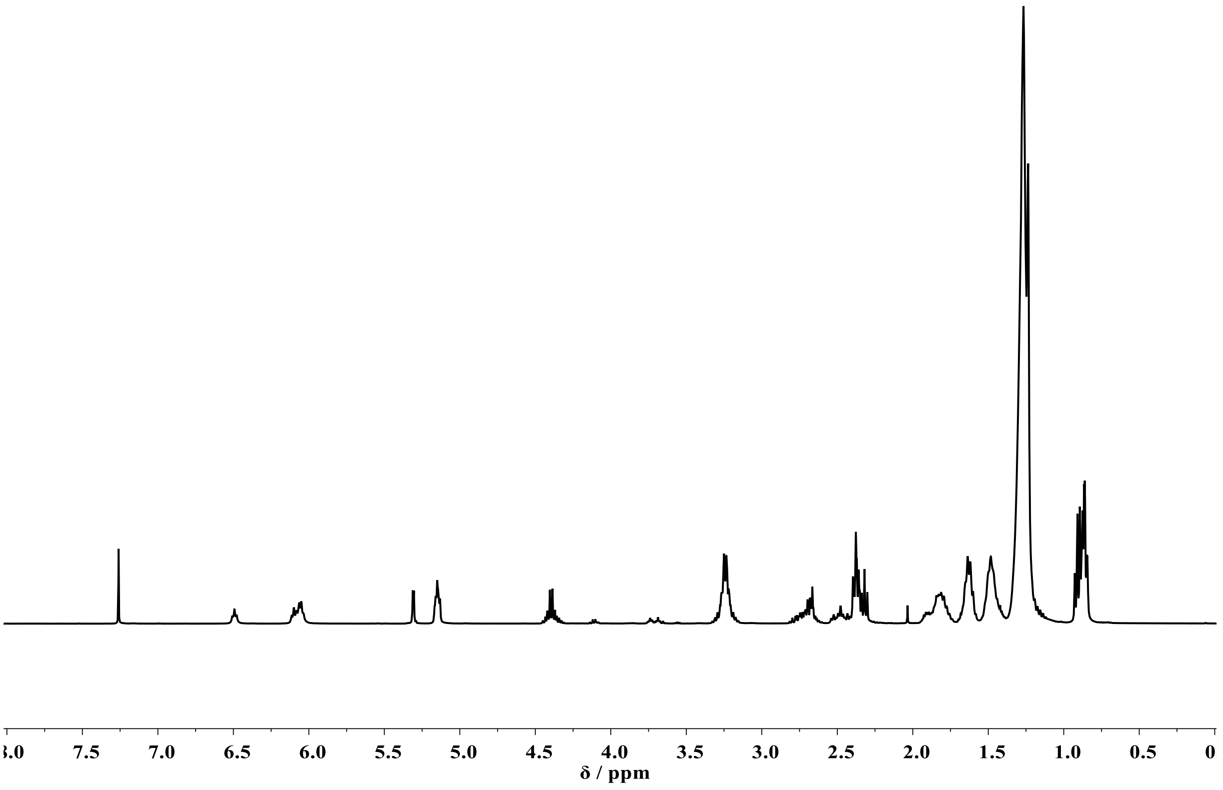

Supplementary Figure 61: ^{1}H-NMR of compound 49 measured in $CDCl_3$.

Passerini reaction

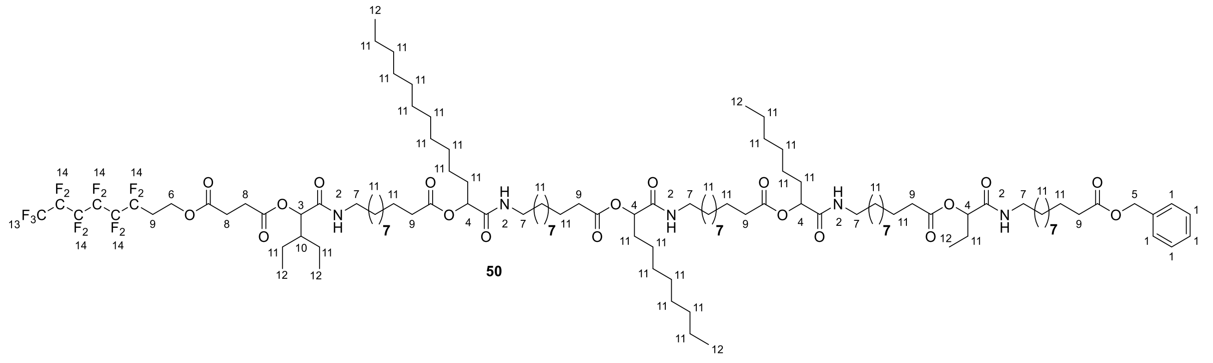

In a 10 mL round bottom flask, 1.28 g of **50** (690 µmol, 1.00 eq.) was stirred in 2.10 mL DCM. Subsequently, 148 µL propionaldehyde **14l** (120 mg, 2.07, 3.00 eq.) and 312 mg of the monomer **M1** (1.04 mmol, 1.50 eq.) were added. The resulting reaction mixture was stirred at room temperature for 3 days. Afterwards, the crude mixture was dried under reduced pressure. The residue was adsorbed onto celite® and purified *via* column chromatography on silica gel eluting with a gradual solvent mixture of ethyl acetate and cyclohexane (4:1 → 1:1) to yield the passerini product **50** as a pale highly viscous oil. (1.39 g, 626 µmol, 90.7%).

R_f = 0.61 in cyclohexane / ethyl acetate (3:2).

IR (ATR): ν / cm^{-1} = 3304.8 (vw), 2923.9 (s), 2853.8 (m), 1739.6 (s), 1654.1 (s), 1535.4 (m), 1459.0 (w), 1374.5 (w), 1236.5 (s), 1145.2 (vs), 697.1 (w), 651.2 (w).

^{1}H NMR (400 MHz, $CDCl_3$): δ / ppm = 7.39 – 7.23 (m, 5 H, CH_{Ar}^{1}), 6.42 (t, J = 5.8 Hz, 1 H, NH^{2}), 6.12 – 5.89 (m, 4 H, NH^{2}), 5.25 (d, J = 3.1 Hz, 1 H, CH^{3}), 5.16 – 4.98 (m, 6 H, CH^{4}, CH_2^{5}), 4.45 – 4.26 (m, 2 H, CH_2^{6}), 3.33 – 3.08 (m, 10 H, CH_2^{7}), 2.79 – 2.53 (m, 4 H, CH_2^{8}), 2.50 – 2.24 (m, 12 H, CH_2^{9}), 1.90 – 1.51 (m, 21 H, CH^{10}, CH_2^{11}), 1.50 – 1.02 (m, 112 H, CH_2^{11}), 0.90 – 0.70 (m, 18 H, CH_3^{12}).

^{13}C NMR (101MHz, $CDCl_3$): δ / ppm = 173.80, 172.84, 172.60, 172.55, 171.22, 170.00, 169.74, 169.62, 136.24, 128.66, 128.27, 75.75, 74.92, 74.04, 66.18, 56.97, 43.34, 39.45, 39.30, 34.43, 32.03, 31.94, 31.74, 29.73, 29.69, 29.65, 29.60, 29.57, 29.55, 29.50, 29.46, 29.37, 29.33, 29.24, 29.22, 29.18, 29.12, 29.03, 26.97, 26.95, 25.21, 25.08, 24.88, 24.83, 22.80, 22.76, 22.65, 22.53, 22.15, 14.22, 14.22, 14.16, 11.85, 11.73, 9.13.

^{19}F NMR (376 MHz, $CDCl_3$): δ / ppm = -80.64 – -81.03 (m, 3 F, CF_3^{13}), -113.54 – -113.82 (m, 2 F, CF_2^{14}), -121.66 – -122.01 (m 2 F, CF_2^{14}), -122.72 – -122.99 (m 2 F, CF_2^{14}), -123.41 – -123.70 (m 2 F, CF_2^{14}), -125.95 – -126.19 (m 2 F, CF_2^{14}). Total integral of CF_2 region normalized with respect to the CF_3^{15} group = 10.

ESI-MS [*m/z*]: $[M + H]^{+}$ calculated for $^{12}C_{116}{}^{1}H_{194}{}^{16}O_{19}{}^{14}N^{19}F_{13}$, 2209.4233; found, 2209.4261, Δ = 2.8 mmu.

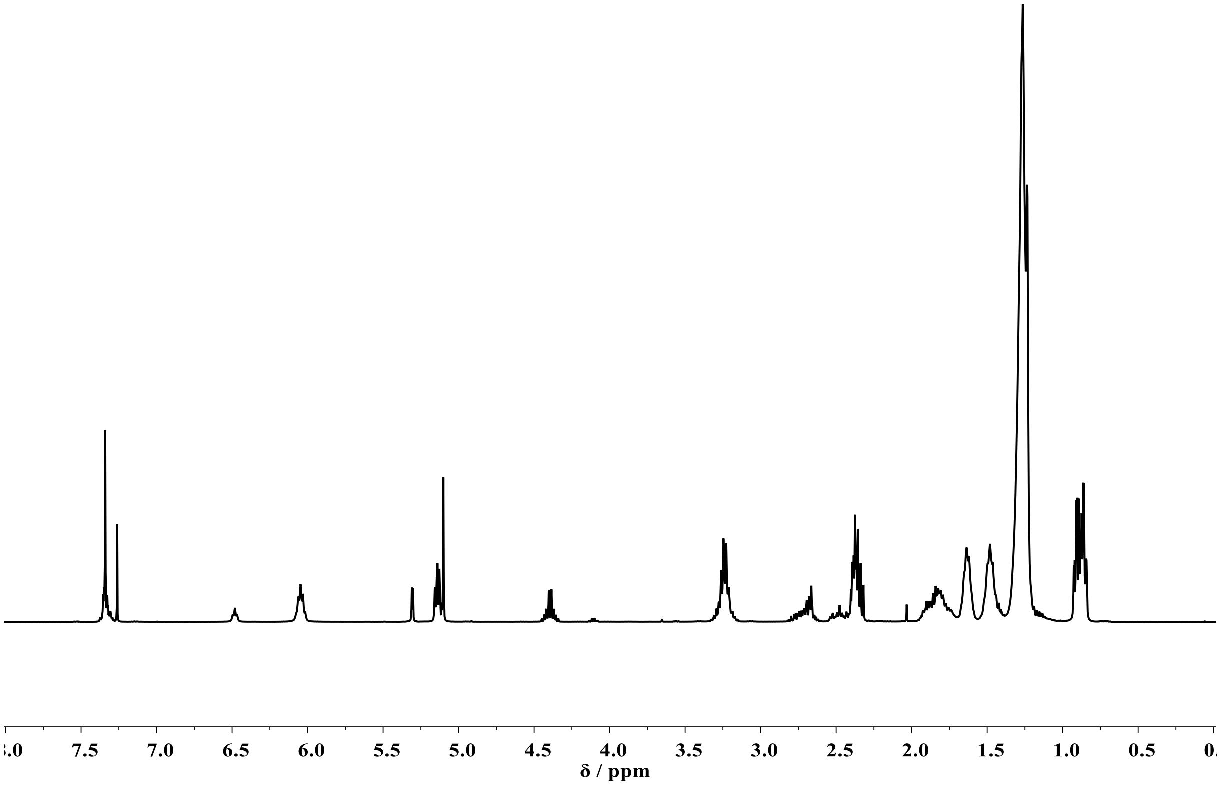

Supplementary Figure 62: ^{1}H-NMR of compound 50 measured in $CDCl_3$.

Deprotection

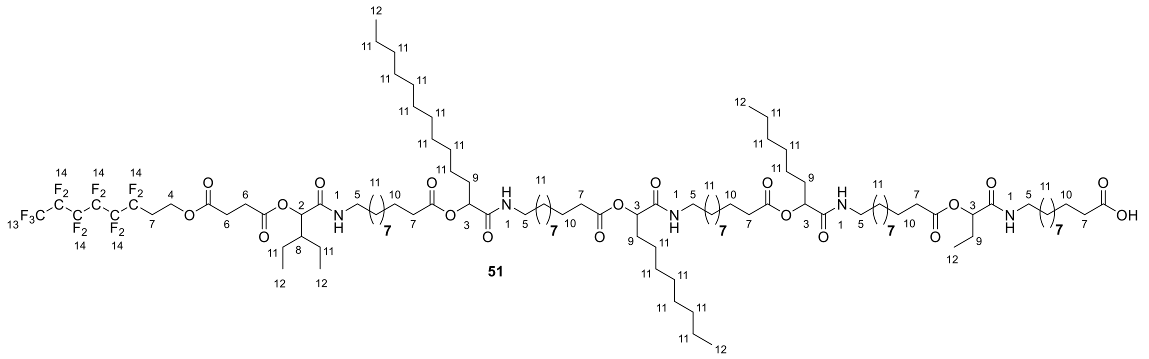

51

In a 50 mL round bottom flask, 1.04 g of **50** (469 µmol, 1.00 eq.) were dissolved in 5.00 mL ethyl acetate and 5.00 mL THF. Afterwards, 207 mg (20 wt%) palladium on activated charcoal **16** were added. Subsequently, the mixture was purged with hydrogen (3 balloons) and stirred under hydrogen atmosphere overnight. The heterogeneous catalyst was filtered off and the solvent was evaporated under reduced pressure. The product **51** was obtained as a pale highly viscous oil in a yield of 98.7% (981 mg, 462 µmol).

IR (ATR): ν / cm^{-1} = 3305.4 (vw), 2924.0 (s), 2854.0 (m), 1740.7 (s), 1654.4 (s), 1539.7 (m), 1463.5 (w), 1374.8 (w), 1236.9 (vs), 1145.3 (vs), 1008.3 (w), 841.8 (vw), 808.9 (vw), 720.6 (w), 651.0 (w), 400.3 (vw).

^{1}H NMR (400 MHz, $CDCl_3$): δ / ppm = 6.49 (t, J = 5.8 Hz, 1 H, NH^1), 6.17 – 5.99 (m, 4 H, NH^1), 5.31 (d, J = 3.2 Hz, 1 H, CH^2), 5.16 – 5.08 (m, 4 H, CH^3), 4.49 – 4.30 (m, 2 H, CH_2^4), 3.35 – 3.14 (m, 10 H, CH_2^5), 2.84 – 2.58 (m, 4 H, CH_2^6), 2.55 – 2.25 (m, 12 H, CH_2^7), 1.98 – 1.72 (m, 9 H, CH^8, CH_2^9), 1.70 – 1.56 (m, 10 H, CH_2^{10}), 1.53 – 1.12 (m, 127 H, CH_2^{11}), 0.96 – 0.82 (m, 18 H, CH_3^{12}).

^{13}C NMR (101 MHz, $CDCl_3$): δ / ppm = ^{13}C NMR (126 MHz, Chloroform-*d*) δ 176.86, 172.87, 172.70, 172.64, 172.63, 172.59, 171.25, 170.18, 170.11, 170.09, 169.86, 169.70, 75.74, 74.94, 74.04, 62.92, 56.98, 43.34, 39.48, 39.37, 39.34, 39.29, 34.43, 33.96, 32.02, 32.01, 31.95, 31.75, 30.73, 30.56, 30.39, 30.00, 29.74, 29.69, 29.66, 29.61, 29.59, 29.56, 29.52, 29.50, 29.47, 29.42, 29.37, 29.37, 29.33, 29.24, 29.21, 29.18, 29.13, 29.09, 29.03, 26.98, 26.95, 26.87, 25.19, 25.11, 25.08, 24.91, 24.90, 24.84, 22.66, 22.53, 22.15, 14.24, 14.23, 14.17, 11.85, 11.73, 9.15.

^{19}F NMR (376 MHz, $CDCl_3$): δ / ppm = -80.56 – -80.94 (m, 3 F, CF_3), -113.44 – -113.80 (m, 2 F, CF_2), -121.63 – -122.01 (m), -122.60 – -123.02 (m), -123.25 – -123.84 (m), -125.82 – -126.33 (m). Total integral of CF_2 region normalized with respect to the CF_3^{14} group = 10.

ESI-MS [*m/z*]: $[M + H]^+$ calculated for $^{12}C_{109}{}^{1}H_{188}{}^{16}O_{19}{}^{14}N_5{}^{19}F_{13}$, 2119.3764; found, 2119.3829, Δ = 6.5 mmu.

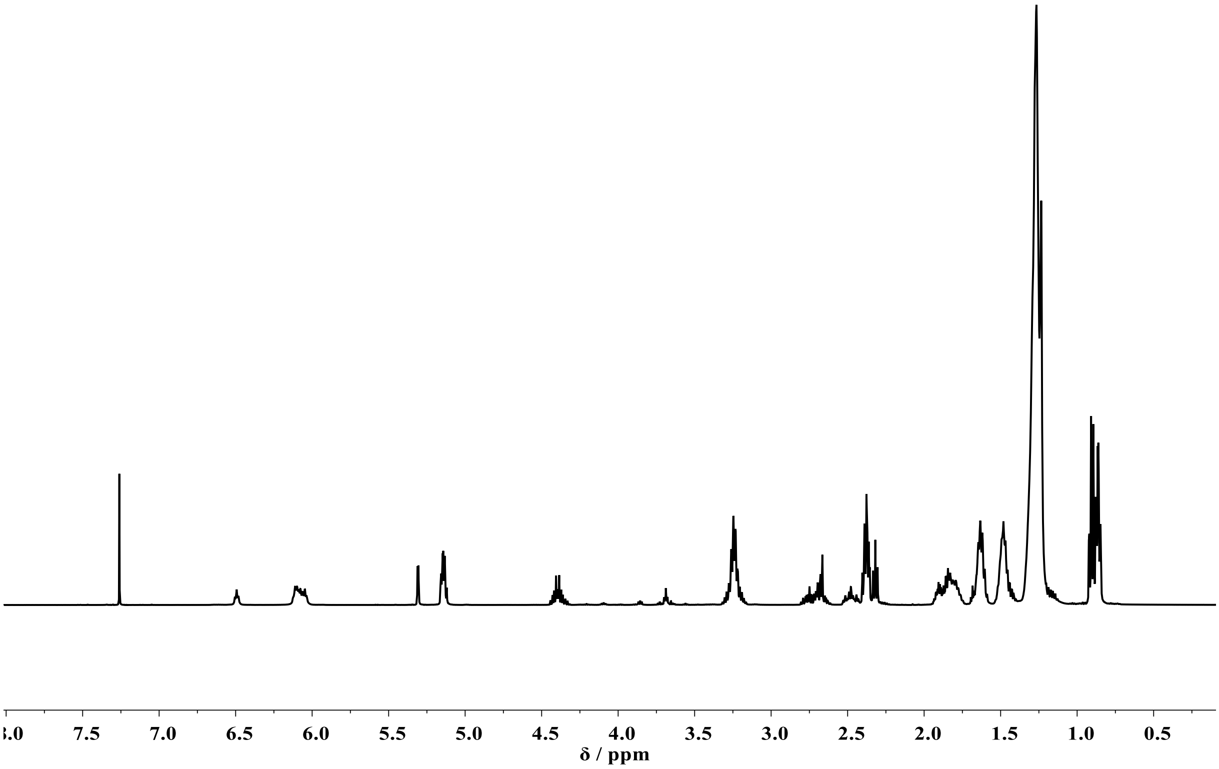

Supplementary Figure 63: ^{1}H-NMR of compound 51 measured in $CDCl_3$.

Passerini reaction

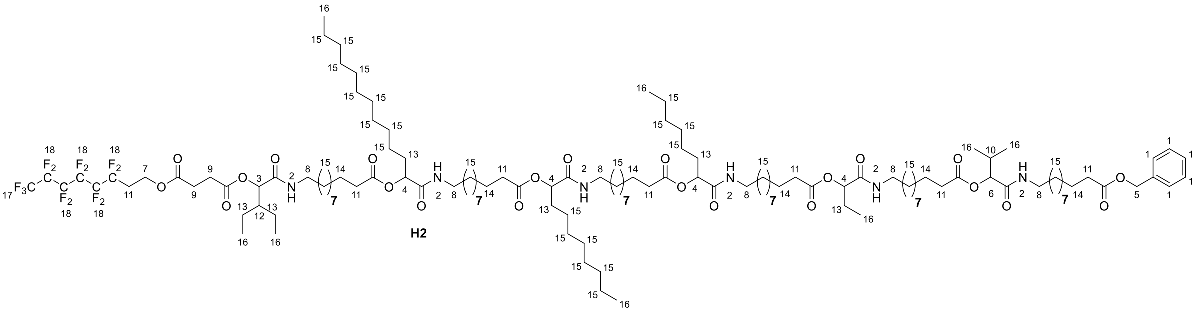

In a 50 mL round bottom flask, 877 mg of **51** (414 µmol, 1.00 eq.) was dissolved in 2.50 mL DCM and 57.0 µL isobutyraldehyde **14c** (44.8 mg, 621 µmol, 1.50 eq.) and 187 mg of monomer **M1** (621 µmol, 1.50 eq.) were added. The mixture was stirred at room temperature for 2 days and subsequently the solvent was removed under reduced pressure. The crude product was purified by column chromatography (cyclohexane / ethyl acetate 4:1→ 1:2) to afford product **H2** as a white solid in a yield of 83.8% (863 mg, 347 µmol).

R_f = 0.71 in cyclohexane / ethyl acetate (1:1).

IR (ATR): ν / cm^{-1} = 3306.1 (vw), 2924.1 (s), 2853.9 (m), 1740.5 (s), 1654.5 (s), 1535.9 (m), 1462.7 (w), 1370.2 (w), 1237.3 (s), 1145.6 (s), 697.4 (w).

^{1}H NMR (400 MHz, $CDCl_3$): δ / ppm = 7.43 – 7.31 (m, 5 H, CH_{Ar}^{1}), 6.50 (t, J = 5.8 Hz, 1 H, NH^{2}), 6.13 – 5.96 (m, 5 H, NH^{2}), 5.33 (d, J = 3.1 Hz, 1 H, CH^{3}), 5.20 – 5.14 (m, 4 H, CH^{4}), 5.12 (s, 2 H, CH_2^{5}), 5.07 (d, J = 4.5 Hz, 1 H, CH^{6}), 4.50 – 4.33 (m, 2 H, CH_2^{7}), 3.38 – 3.16 (m, 12 H, CH_2^{8}), 2.84 – 2.61 (m, 4 H, CH_2^{9}), 2.58 – 2.25 (m, 15 H, CH^{10}, CH_2^{11}), 1.99 – 1.75 (m, 9 H, CH^{12}, CH_2^{13}), 1.71 – 1.60 (m, 12 H, CH_2^{14}), 1.57 – 1.11 (m, 126 H, CH_2^{15}), 0.98 – 0.83 (m, 24 H, CH_3^{16}).

^{13}C NMR (101 MHz, $CDCl_3$): δ / ppm = 173.81, 172.85, 172.68, 172.60, 172.57, 171.23, 169.99, 169.75, 169.61, 169.38, 136.24, 128.66, 128.28, 78.03, 75.74, 74.92, 74.04, 66.19, 56.97, 43.34, 39.45, 39.30, 39.28, 34.44, 34.41, 32.04, 31.95, 31.75, 30.64, 29.74, 29.70, 29.66, 29.61, 29.58, 29.56, 29.51, 29.47, 29.37, 29.33, 29.31, 29.24, 29.22, 29.18, 29.13, 29.03, 26.96, 25.21, 25.12, 25.08, 25.06, 24.89, 24.84, 22.80, 22.76, 22.65, 22.53, 22.15, 18.91, 17.08, 14.23, 14.21, 14.17, 11.86, 11.73, 9.15.

^{19}F NMR (376 MHz, $CDCl_3$): δ / ppm = -85.08 (t, J = 9.9 Hz, 3 F, CF_3^{17}), -117.53 – -118.42 (m, 2 F, CF_2^{18}), -125.96 – -126.57 (m, 2 F, CF_2^{18}), -126.82 – -127.46 (m, 2 F, CF_2^{18}), -127.67 – -128.17 (m, 2 F, CF_2^{18}), -129.91 – -130.82 (m, 2 F, CF_2^{18}). Total integral of CF_2 region normalized with respect to the CF_3^{18}group = 10.

ESI-MS [*m/z*]: $[M + H]^+$ calculated for $^{12}C_{132}{}^{1}H_{223}{}^{19}F_{13}{}^{14}N_6{}^{16}O_{22}$, 2492.6381; found, 2492.6446, Δ = 6.5 mmu.

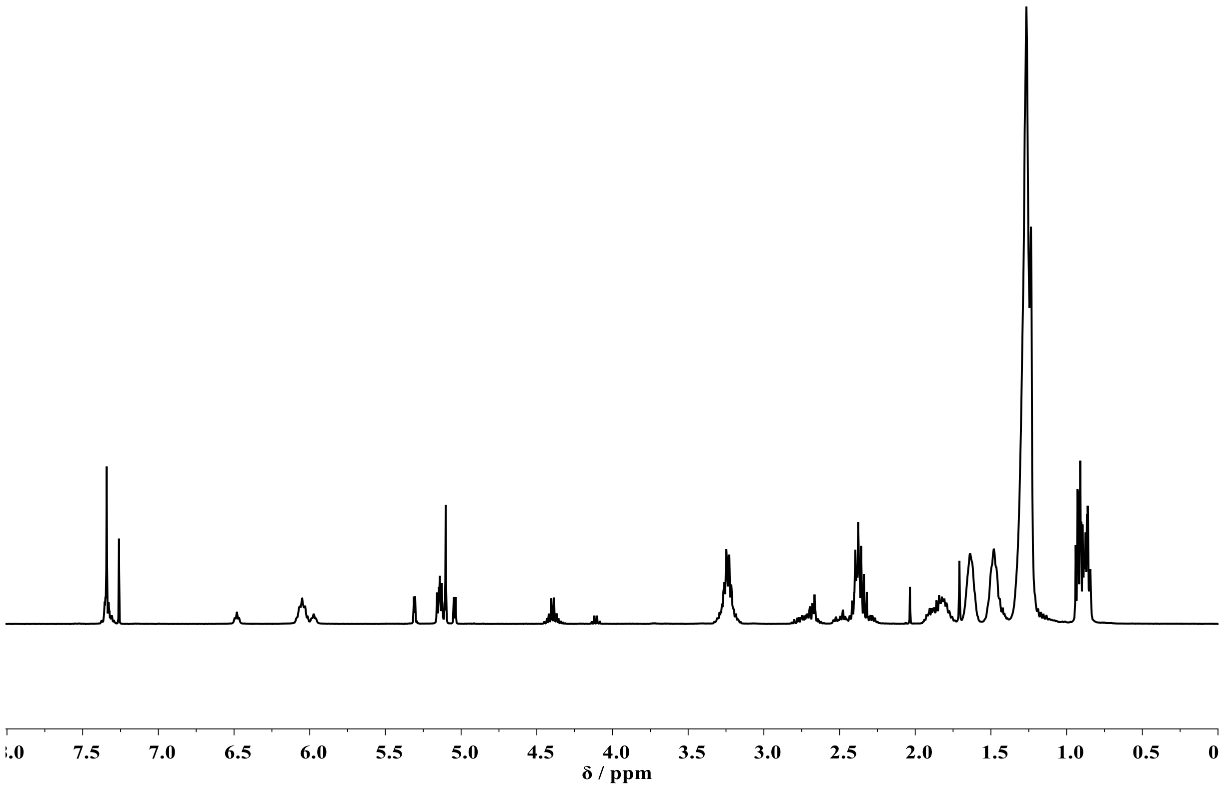

Supplementary Figure 64: ^{1}H-NMR of compound H2 measured in $CDCl_3$.

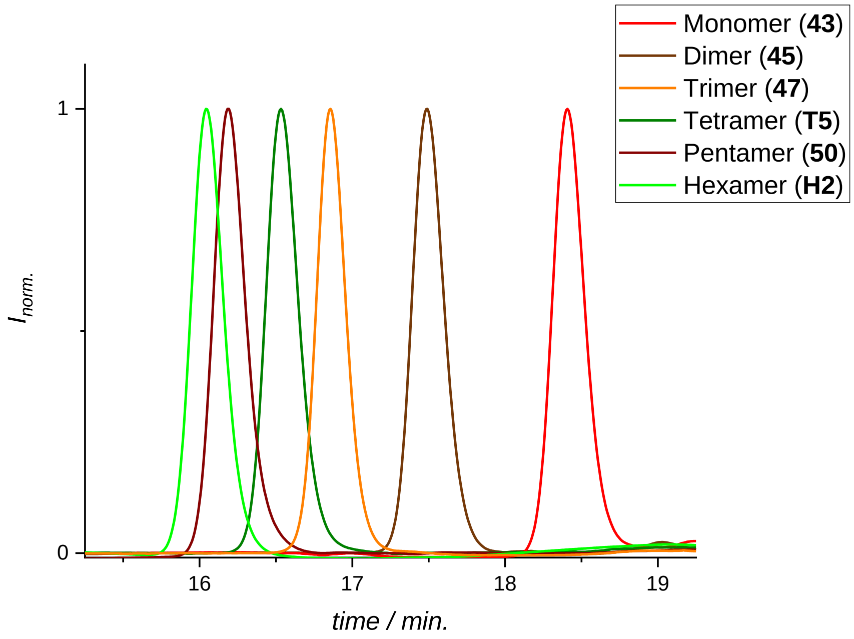

Supplementary Figure 65: SEC traces of the intermediates after each P3CR in the synthesis of product H2

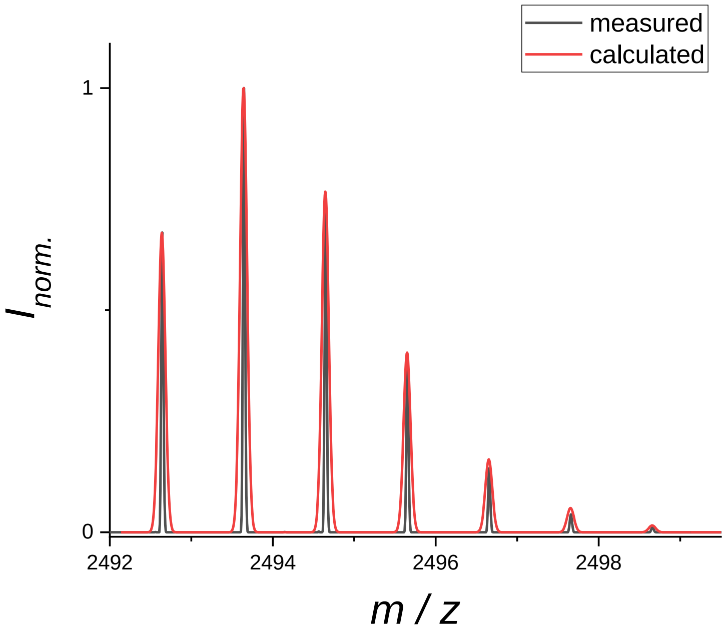

Supplementary Figure 66: High resolution ESI-MS measurement of H2. The observed isotopic pattern is compared with the calculated isotopic pattern obtained from mMass (black).

```
found 22260 values in C:\Users\Maxi\Documents\Mixture 2492 NCE 18 with 3.CSV, maximum is 1.000000 found for mass 2492.647
480
matching mass 2492.64748
cutoff 0.50000: 0 solutions (4 peaks)
cutoff 0.25000: 0 solutions (11 peaks)
cutoff 0.12500: 0 solutions (37 peaks)
cutoff 0.06250: 0 solutions (69 peaks)
cutoff 0.03125: 0 solutions (119 peaks)
cutoff 0.01562: 0 solutions (230 peaks)
cutoff 0.00781: 0 solutions (375 peaks)
cutoff 0.00391: 0 solutions (623 peaks)
cutoff 0.00195: 0 solutions (975 peaks)
cutoff 0.00098: 1 solutions (1465 peaks)
2492.64748 ≈ 447.026590 + 311.246050 + 395.339950 + 353.293000 + 325.261700 + 269.199100 + 283.214750 + 107.049690 (sides
 2-Ethylbutanal, Dodecanal, Nonanal, Heptanal, Propionaldehyde, Isobutyraldehyde; error -1.01665)
Press ENTER to quit ...
```

Supplementary Figure 67: Screenshot of the automated read-out of H2.

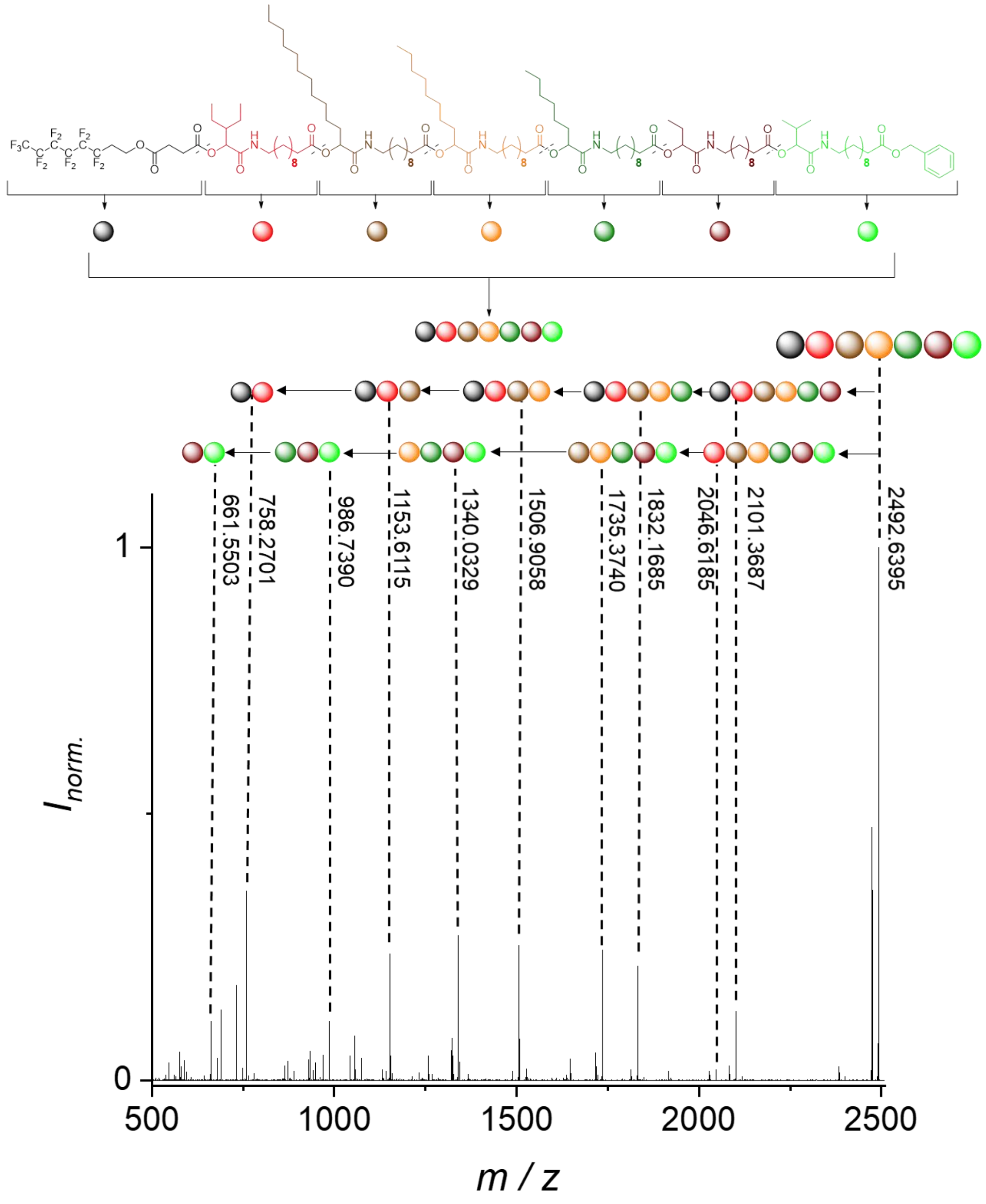

Supplementary Figure 68: Read-out of the sequence-defined hexamer H2. Read-out of the hexamer H2 via tandem ESI-MS/MS with an NCE of 18. In the spectrum, the read-out from both ends of the oligomer using the fragmentation next to the carbonyl are shown.

1.3.4.3 Synthesis of tetramer T6

Passerini reaction

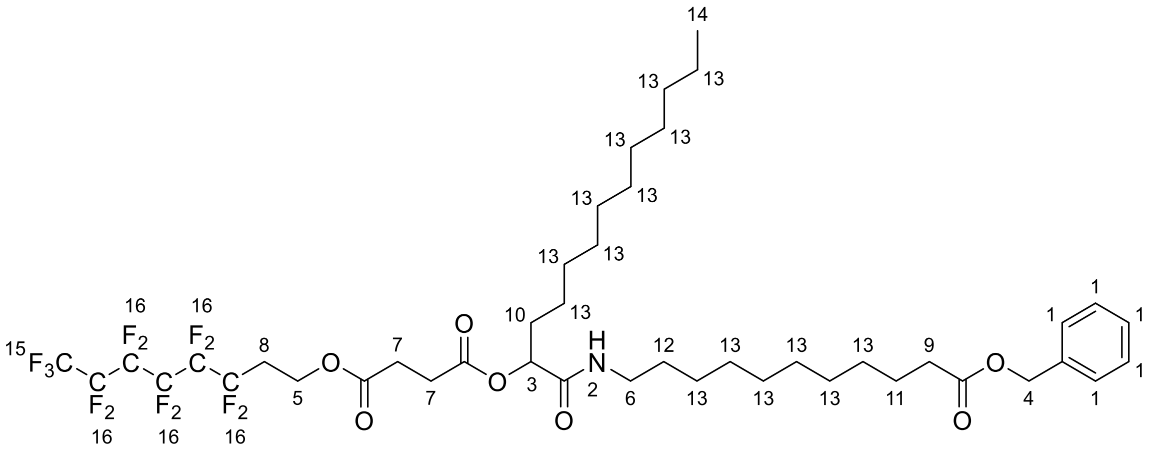

52

In 50.0 mL round bottom flaks, 1.00 g **TAG1** (2.15 mmol, 1.00 eq.) were dissolved in 6.00 mL DCM and 550 mg dodecanal **14h** (3.23 mmol, 1.50 eq.) and 974 mg of monomer **M1** (3.23 mol, 1.50 eq.) were added. The mixture was stirred at room temperature for 2 days. Subsequently, the solvent was removed under reduced pressure. The crude product was purified by column chromatography (hexane / ethyl acetate 6:1 → 4:1) to afford product **52** as a yellow oil in a yield of 94.0% (1.93 g, 2.02 mmol).

R_f = 0.60 in cyclohexane / ethyl acetate (3:1).

IR (ATR): ν / cm^{-1}] = 2925.0 (m), 2854.4 (w), 1738.1 (s), 1658.1 (w), 1535.5 (w), 1457.7 (w), 1359.5 (w), 1235.3 (vs), 12028m.1 (vs), 1144.6 (vs), 1081.9 (m), 1005.2 (w), 842.0 (vw), 808.9 (vw), 732.4 (w), 697.2 (m), 651.2 (w).

^{1}H NMR (400 MHz, $CDCl_3$): δ / ppm = 7.41 – 7.28 (m, 5 H, CH_{Ar}^{1}), 6.37 (t, J = 5.8 Hz, 1 H, NH^{2}), 5.19 – 5.15 (m, 1 H, CH^{3}), 5.11 (s, 2 H, CH_2^{4}), 4.48 – 4.32 (m, 2 H, CH_2^{5}), 3.34 – 3.15 (m, 2 H, CH_2^{6}), 2.82 – 2.60 (m, 4 H, CH_2^{7}), 2.48 (s, 2 H, CH_2^{8}), 2.34 (t, J = 7.5 Hz, 2 H, CH_2^{9}), 1.96 – 1.74 (m, 2 H, CH_2^{10}), 1.68 – 1.58 (m, 2 H, CH_2^{11}), 1.56 – 1.44 (m, 2 H, CH_2^{12}), 1.36 – 1.19 (m, 30 H, CH_2^{13}), 0.87 (t, J = 6.8 Hz, 3 H, CH_3^{14}).

^{13}C NMR (101 MHz, $CDCl_3$): δ / ppm = 173.81, 172.63, 171.24, 169.71, 136.26, 128.66, 128.28, 74.75, 66.19, 56.95, 39.46, 34.44, 32.04, 31.94, 30.57, 29.75, 29.69, 29.60, 29.58, 29.53, 29.49, 29.48, 29.38, 29.35, 29.24, 29.22, 29.13, 26.97, 25.06, 25.03, 22.82, 14.24.

^{19}F NMR (376 MHz, $CDCl_3$): δ / ppm = -83.76 – -86.21 (m, 3 F, CF_3^{15}), -117.01 – -118.71 (m, 2 F, CF_2^{16}), -126.05 – -126.35 (m, 2 F, CF_2^{16}), -127.05 – -127.34 (m, 2 F, CF_2^{16}), -127.79 – -128.06 (m, 2 F, CF_2^{16}), -130.36 – -130.58 (m, 2 F, CF_2^{16}). Total integral of CF_2 region normalized with respect to the CF_3^{15} group = 10.

ESI-MS [m/z]: $[M + H]^+$ calculated for $^{12}C_{43}{}^{1}H_{60}{}^{16}O_7{}^{14}N^{19}F_{13}$, 950.4235; found, 950.4210, Δ = 2.5 mmu.

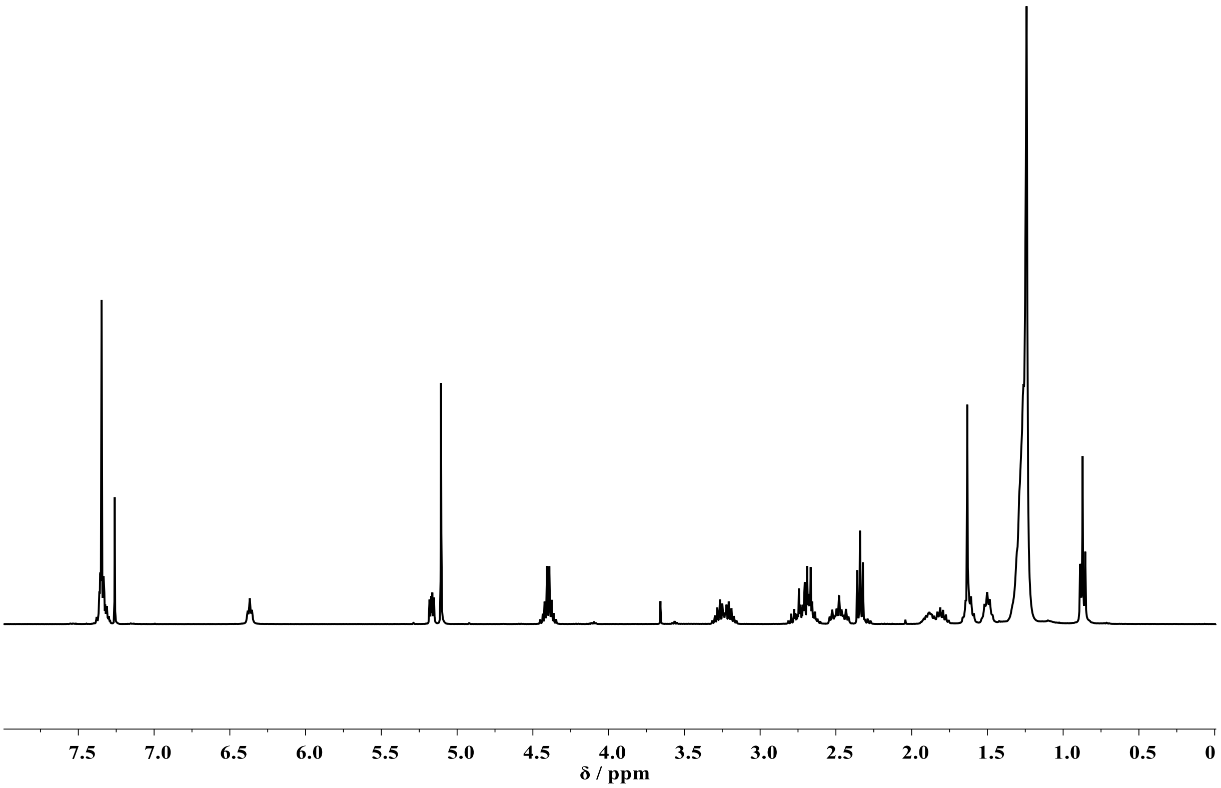

Supplementary Figure 69: ^{1}H-NMR of compound 51 measured in $CDCl_3$.

Deprotection

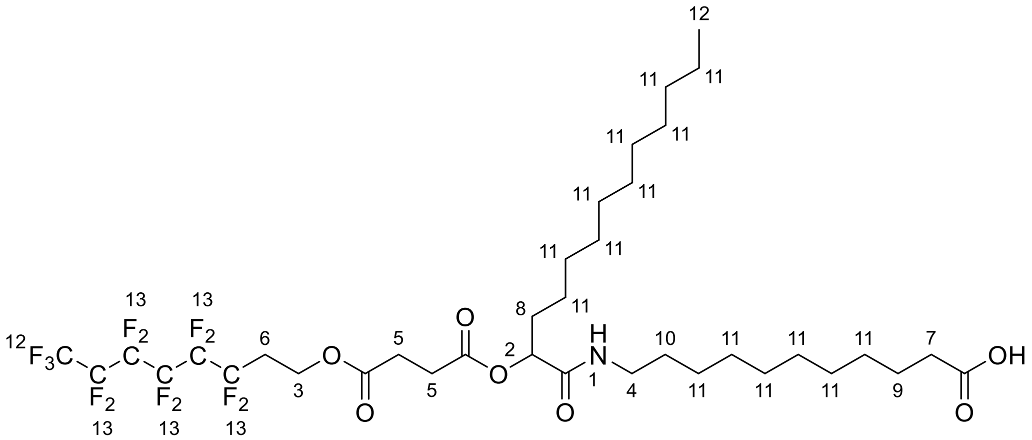

52

In a 50 mL round bottom flask, 1.82 g of **51** (1.91 mmol, 1.00 eq.) were dissolved in 3.00 mL ethyl acetate and 3.00 mL THF. Afterwards, 363 mg (20 wt%) palladium on activated charcoal **16** were added. Subsequently, the mixture was purged with hydrogen (3 balloons) and stirred under hydrogen atmosphere overnight. The heterogeneous catalyst was filtered off and the solvent was evaporated under reduced pressure. The product **52** was obtained as a high viscous oil in a yield of 96.3% (1.58 g, 1.84 mmol).

IR (ATR): ν / cm^{-1} = 3291.4 (w), 2913.2 (vs), 2847.8 (s), 1740.8 (vs), 1695.6 (s), 1659.5 (vs), 1556.4 (m), 1469.3 (m), 1414.0 (w), 1360.7 (m), 1188.8 (vs), 1162.5 (vs), 1141.8 (vs), 1080.1 (s), 905.0 (w), 839.7 (w), 808.4 (w), 732.6 (m), 698.7 (s), 651.0 (m), 565.9 (w), 529.5 (w), 460.5 (w).

^{1}H NMR (400 MHz, $CDCl_3$): δ / ppm = 6.41 (t, J = 5.8 Hz, 1 H, NH^1), 5.21 – 5.13 (m, 1 H, CH^2), 4.50 – 4.31 (m, 2 H, $CH_2{}^3$), 3.33 – 3.14 (m, 2 H, $CH_2{}^4$), 2.84 – 2.60 (m, 4 H, $CH_2{}^5$), 2.56 – 2.40 (m, 2 H, $CH_2{}^6$), 2.33 (t, J = 7.5 Hz, 2 H, $CH_2{}^7$), 1.96 – 1.74 (m, 2 H, $CH_2{}^8$), 1.67 – 1.56 (m, 2 H, $CH_2{}^9$), 1.54 – 1.45 (m, 2 H, $CH_2{}^{10}$), 1.39 – 1.17 (m, 30 H, $CH_2{}^{11}$), 0.87 (t, J = 6.7 Hz, 3 H, $CH_3{}^{12}$).

^{13}C NMR (101 MHz, $CDCl_3$): δ / ppm = 178.65, 172.69, 171.28, 74.76, 56.98, 39.49, 33.99, 32.05, 31.93, 30.60, 29.76, 29.70, 29.59, 29.49, 29.46, 29.38, 29.35, 29.25, 29.23, 29.21, 29.15, 29.08, 26.91, 25.04, 24.79, 22.82, 14.24.

^{19}F NMR (376 MHz, $CDCl_3$): δ / ppm = -84.77 – -85.47 (m, 3 F, $CF_3{}^{12}$), -117.91 – -118.15 (m, 2 F, $CF_2{}^{13}$), -126.04 – -126.37 (m, 2 F, $CF_2{}^{13}$), -127.07 – -127.35 (m, 2 F, $CF_2{}^{13}$), -127.78 – -128.08 (m, 2 F, $CF_2{}^{13}$), -130.31 – -130.62 (m, 2 F, $CF_2{}^{13}$). Total integral of CF_2 region normalized with respect to the $CF_3{}^{13}$ group = 10.

ESI-MS [m/z]: [M + Na]$^+$ calculated for $^{12}C_{36}{}^{1}H_{54}{}^{16}O_7{}^{14}N^{19}F_{13}$, 882.3585; found, 882.3559, Δ = 2.6 mmu.

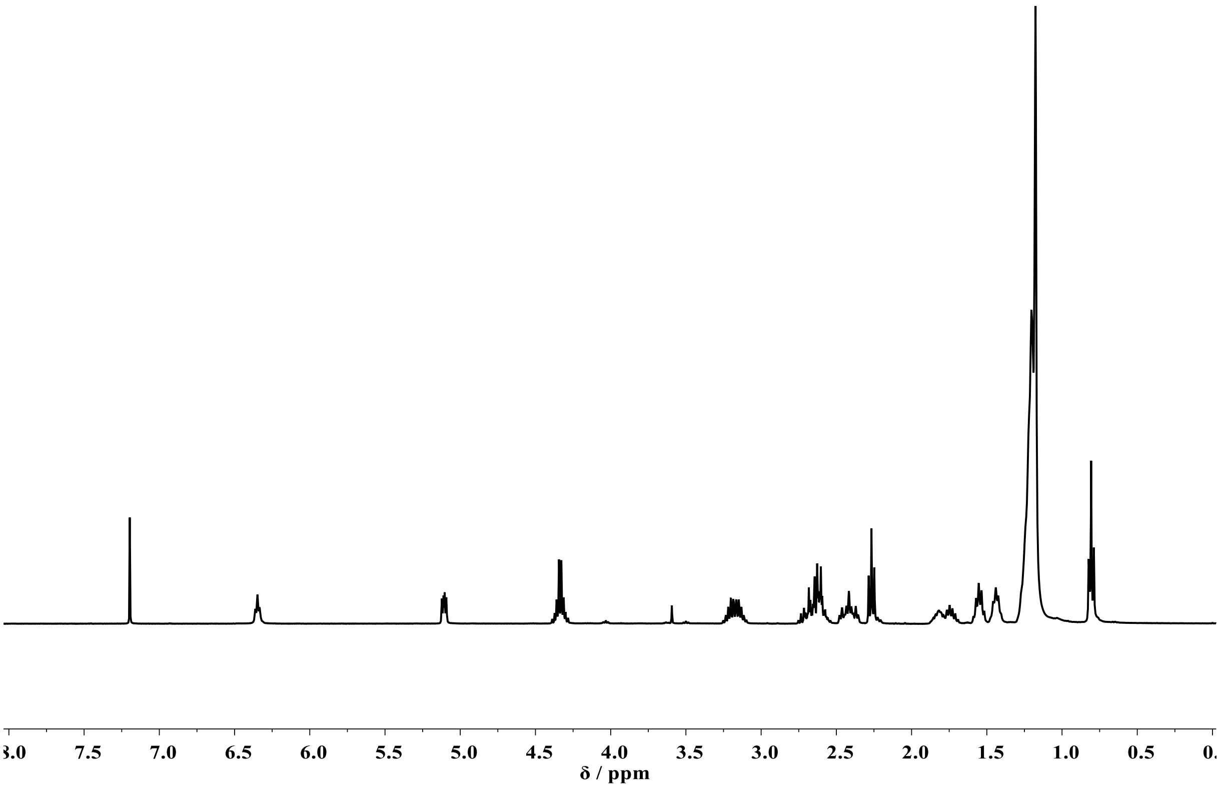

Supplementary Figure 70: ^{1}H-NMR of compound 52 measured in $CDCl_3$.

Passerini reaction

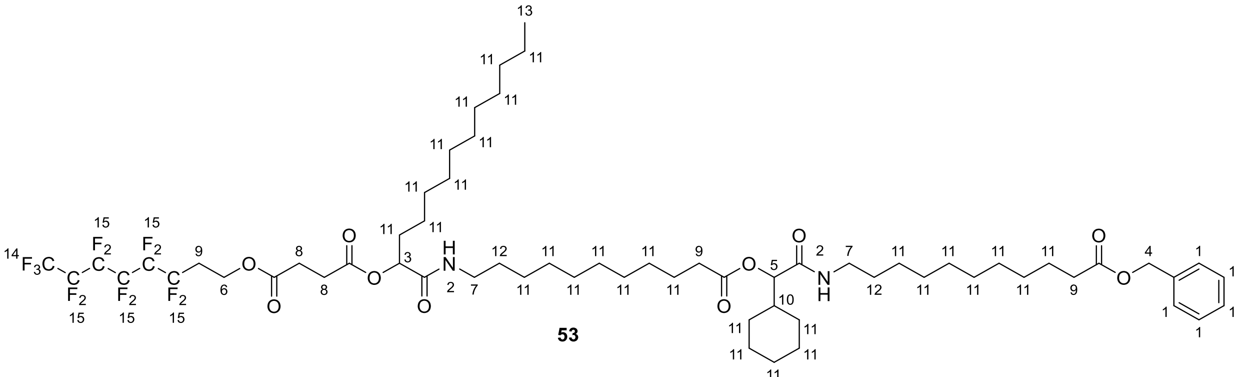

In a 50 mL round bottom flask, 1.53 g of **52** (1.78 mmol, 1.00 eq.) was dissolved in 4.00 mL DCM and 323 µL cyclohexanecarboxaldeyhde **14j** (319 mg, 2.67 mmol, 1.50 eq.) and 857 mg of monomer **M1** (2.67 mmol, 1.50 eq.) were added. The mixture was stirred at room temperature for 3 days. Subsequently, the solvent was removed under reduced pressure. The crude product was purified by column chromatography (cyclohexane / ethyl acetate 5:1 → 2:1) to afford product **53** as a pale highly viscous oil in a yield of 91.1% (2.06 g, 1.62 mmol).

R_f = 0.50 in cyclohexane / ethyl acetate (3:1).

IR (ATR): ν / cm^{-1} = 3285.3 (vw), 2919.4 (s), 2850.8 (m), 1737.6 (vs), 1652.4 (s), 1552.7 (w), 1466.8 (w), 1362.9 (w), 1235.9 (vs), 1143.8 (vs), 1082.8 (m), 1005.2 (w), 842.4 (vw), 809.6 (vw), 697.3 (s), 652.3 (w), 567.2 (vw), 455.5 (vw).

^{1}H NMR (400 MHz, $CDCl_3$): δ / ppm = 7.36 – 7.22 (m, 5 H, $CH_{Ar}{}^{1}$), 6.32 (t, J = 5.8 Hz, 1 H, NH^{2}), 5.86 (t, J = 5.9 Hz, 1 H, NH^{2}), 5.12 – 5.07 (m, 1 H, CH^{3}), 5.04 (s, 2 H, $CH_2{}^{4}$), 4.97 (d, J = 4.6 Hz, 1 H, CH^{5}), 4.43 – 4.25 (m, 2 H, $CH_2{}^{6}$), 3.25 – 3.06 (m, 4 H, $CH_2{}^{7}$), 2.78 – 2.55 (m, 4 H, $CH_2{}^{8}$), 2.51 – 2.21 (m, 6 H, $CH_2{}^{9}$), 1.95 – 1.51 (m, 13 H, CH^{10}, $CH_2{}^{11}$), 1.47 – 1.37 (m, 4 H, $CH_2{}^{12}$), 1.30 – 0.93 (m, 46 H, $CH_2{}^{11}$), 0.84 – 0.76 (m, 3 H, $CH_3{}^{13}$).

^{13}C NMR (101 MHz, $CDCl_3$): δ / ppm = 173.83, 172.65, 171.25, 169.74, 169.34, 136.26, 128.67, 128.29, 77.74, 74.75, 66.20, 56.94, 40.12, 39.44, 39.27, 34.45, 34.42, 32.04, 31.94, 30.57 (t, J = 21.6 Hz), 29.75, 29.70, 29.68, 29.61, 29.57, 29.54, 29.52, 29.47, 29.36, 29.32, 29.27, 29.24, 29.22, 29.13, 27.39, 27.05, 26.97, 26.19, 26.12, 26.01, 25.12, 25.07, 25.04, 22.81, 14.24.

^{19}F NMR (376 MHz, $CDCl_3$): δ / ppm = -84.31 – -85.80 (m, 3 F, $CF_3{}^{14}$), -117.77 – -118.47 (m, 2 F, $CF_2{}^{15}$), -125.71 – -126.57 (m, 2 F, $CF_2{}^{15}$), -126.86 – -127.43 (m, 2 F, $CF_2{}^{15}$), -127.48 – -128.34 (m, 2 F, $CF_2{}^{15}$), -129.95 – -130.75 (m, 2 F, $CF_2{}^{15}$). Total integral of CF_2 region normalized with respect to the $CF_3{}^{14}$group = 10.

ESI-MS [*m/z*]: $[M + Na]^+$ calculated for $^{12}C_{62}{}^{1}H_{93}{}^{16}O_{10}{}^{14}N_2{}^{19}F_{13}$, 1295.6515; found, 1261.6500, Δ = 1.5 mmu.

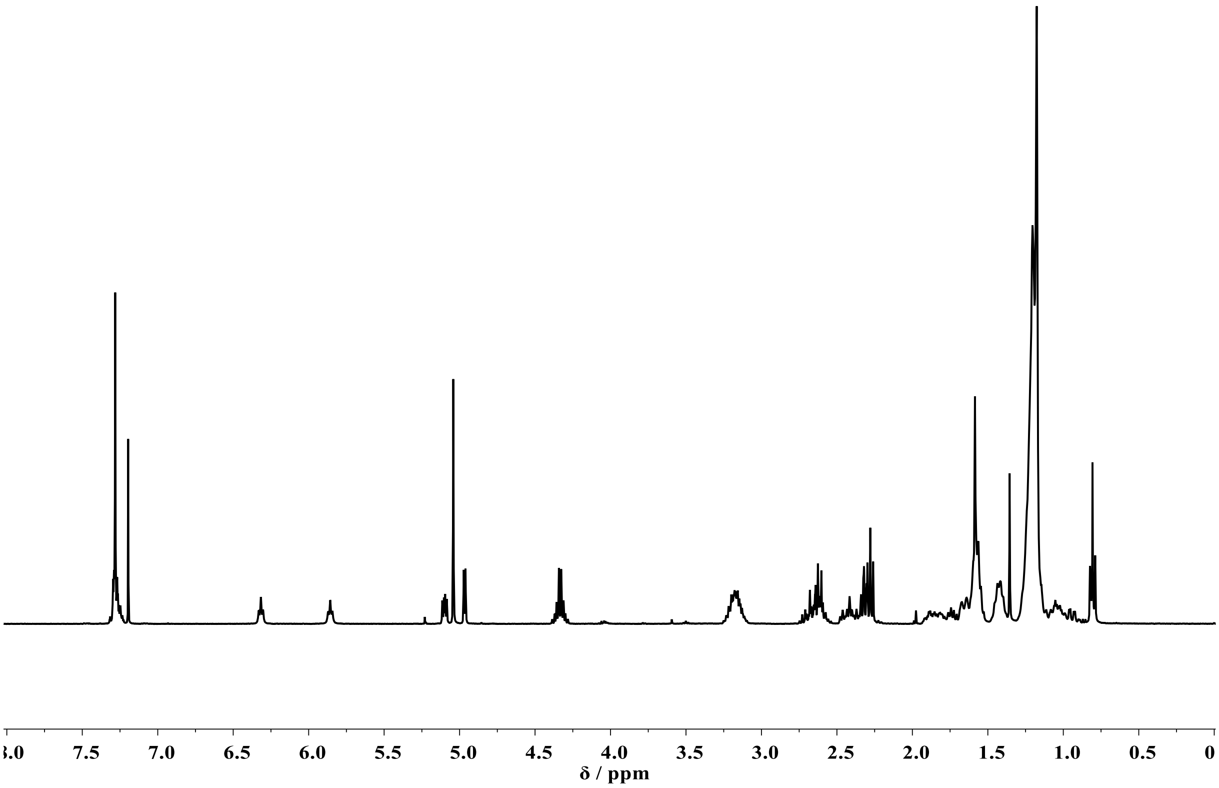

Supplementary Figure 71: ^{1}H-NMR of compound 53 measured in $CDCl_3$.

Deprotection

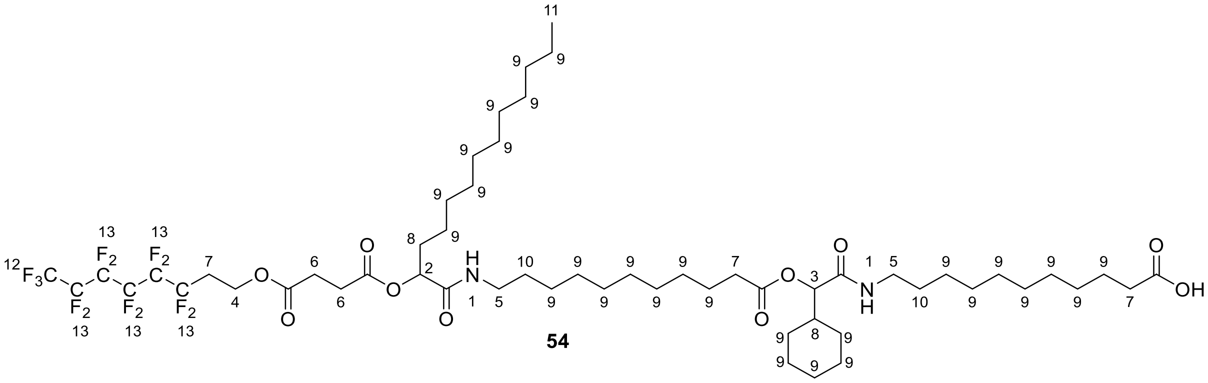

In a 50 mL round bottom flask, 1.98 g of **54** (1.56 mmol, 1.00 eq.) were dissolved in 4.00 mL ethyl acetate and 4.00 mL THF. Afterwards, 396 mg (20 wt%) palladium on activated charcoal **16** were added. Subsequently, the mixture was purged with hydrogen (3 balloons) and stirred under hydrogen atmosphere overnight. The heterogeneous catalyst was filtered off and the solvent was evaporated under reduced pressure. The product **54** was obtained as a high viscous oil in a yield of 98.7% (1.82 g, 1.54 mmol).

IR (ATR): ν / cm^{-1} = 3297.6 (w), 2919.4 (s), 2851.0 (m), 1736.5 (vs), 1655.2 (vs), 1555.0 (m), 1466.3 (w), 1364.5 (m), 1235.5 (vs), 1164.6 (vs), 1143.4 (vs), 1082.3 (m), 1006.9 (w), 842.1 (vw), 810.2 (vw), 697.9 (m), 652.2 (w), 567.0 (vw), 530.7 (vw), 453.2 (vw), 394.1 (vw).

^{1}H NMR (400 MHz, $CDCl_3$): δ / ppm = 6.45 (t, J = 5.8 Hz, 1 H, NH^{1}), 5.97 (t, J = 5.8 Hz, 1 H, NH^{1}), 5.19 – 5.13 (m, 1 H, CH^{2}), 5.03 (d, J = 4.6 Hz, 1 H, CH^{3}), 4.46 – 4.33 (m, 2 H, CH_2^{4}), 3.31 – 3.15 (m, 4 H, CH_2^{5}), 2.81 – 2.58 (m, 4 H, CH_2^{6}), 2.56 – 2.28 (m, 6 H, CH_2^{7}), 2.00 – 1.56 (m, 13 H, CH^{8}, CH_2^{9}), 1.55 – 1.42 (m, 4 H, CH_2^{10}), 1.37 – 0.97 (m, 46 H, CH_2^{9}), 0.93 – 0.82 (m, 3 H, CH_3^{11}).

^{13}C NMR (101 MHz, $CDCl_3$): δ / ppm = 178.65, 173.46, 172.07, 170.72, 170.23, 78.52, 75.49, 57.73, 40.84, 40.28, 40.02, 35.19, 34.81, 32.80, 32.67, 31.34 (t, J = 21.8 Hz), 30.51, 30.44, 30.38, 30.35, 30.33, 30.28, 30.27, 30.23, 30.21, 30.12, 30.04, 29.99, 29.97, 29.89, 29.86, 28.17, 27.73, 27.66, 26.95, 26.87, 26.76, 25.90, 25.79, 25.64, 23.57, 14.98.

^{19}F NMR (376 MHz, $CDCl_3$): δ / ppm = -85.10 (t, J = 9.8 Hz, 3 F, CF_3^{12}), -117.60 – -118.27 (m, 2 F, CF_2^{13}), -125.80 – -126.54 (m, 2 F, CF_2^{13}), -126.84 – -127.47 (m, 2 F, CF_2^{13}), -127.62 – -128.11 (m, 2 F, CF_2^{13}), -130.25 – -130.87 (m, 2 F, CF_2^{13}). Total integral of CF_2 region normalized with respect to the CF_3^{12}group = 10.

ESI-MS [*m/z*]: [M + Na]$^{+}$ calculated for $^{12}C_{55}{}^{1}H_{87}{}^{16}O_{10}{}^{14}N_{2}{}^{19}F_{13}$, 1205.6045; found, 1205.6026, Δ = 1.9 mmu.

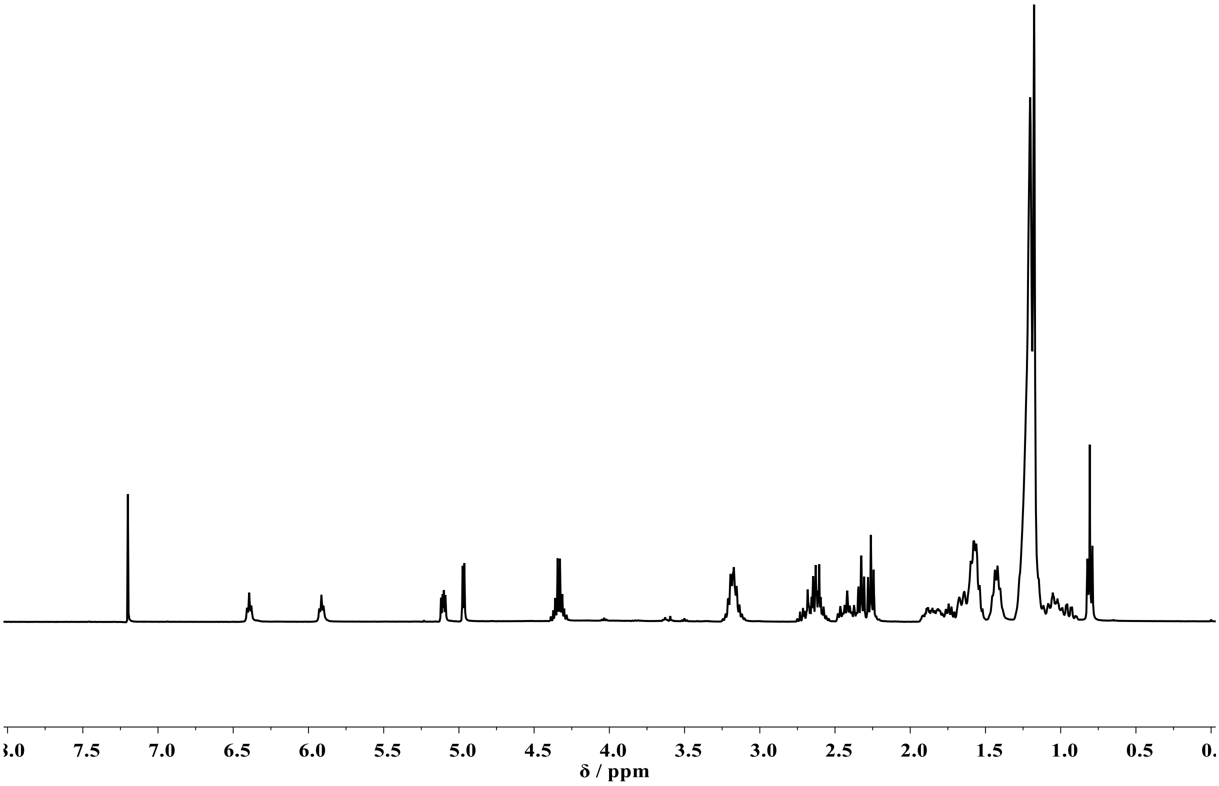

Supplementary Figure 72: ^{1}H-NMR of compound 54 measured in $CDCl_3$.

Passerini reaction

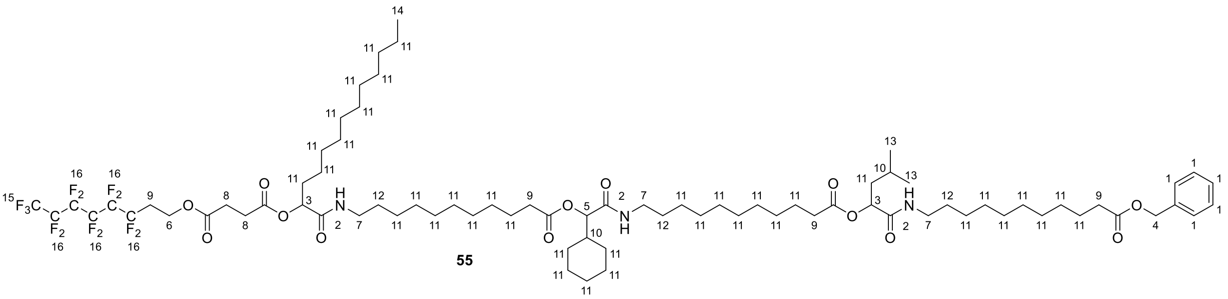

55

In a 50 mL round bottom flask, 1.05 g **54** (886 µmol, 1.00 eq.) was dissolved in 2.00 mL DCM and 143 µL 3-methylbutyraldehyde **14g** (114 mg, 1.33 mmol, 1.50 eq.) and 401 mg of monomer **M1** (1.33 mmol, 1.50 eq.) were added. The mixture was stirred at room temperature for 3 days. Subsequently, the solvent was removed under reduced pressure. The crude product was purified by column chromatography (cyclohexane / ethyl acetate 5:1 → 2:1) to afford product **55** as a pale highly viscous oil in a yield of 86.2% (1.20 g, 764 µmol).

R_f = 0.34 in cyclohexane / ethyl acetate (3:1).

IR (ATR): ν / cm^{-1} = 3299.8 (vw), 2920.7 (s), 2851.3 (m), 1736.8 (s), 1655.3 (vs), 1553.2 (w), 1465.8 (w), 1365.0 (w), 1236.2 (s), 1206.9 (s), 1143.7 (vs), 1005.2 (w), 697.1 (m), 652.1 (w), 567.5 (vw), 450.7 (vw), 394.2 (vw).

^{1}H NMR (400 MHz, $CDCl_3$): δ / ppm = 7.35 – 7.22 (m, 5 H, CH_{Ar}^{1}), 6.33 (t, J = 5.8 Hz, 1 H, NH^{2}), 5.97 – 5.82 (m, 2 H, NH^{2}), 5.15 – 5.06 (m, 2 H, CH^{3}), 5.04 (s, 2 H, CH_2^{4}), 4.96 (d, J = 4.6 Hz, 1 H, CH^{5}), 4.42 – 4.25 (m, 2 H, CH_2^{6}), 3.25 – 3.07 (m, 6 H, CH^{7}), 2.76 – 2.53 (m, 4 H, CH_2^{8}), 2.50 – 2.23 (m, 8 H, CH_2^{9}), 1.95 – 1.51 (m, 16 H, CH^{10}, CH_2^{11}), 1.48 – 1.35 (m, 6 H, CH_2^{12}), 1.32 – 0.96 (m, 60 H, CH_2^{11}), 0.86 (t, J = 5.7 Hz, 6 H, CH_3^{13}), 0.81 (t, J = 6.8 Hz, 3 H, CH_3^{14}).

^{13}C NMR (101 MHz, $CDCl_3$): δ / ppm = 173.82, 172.77, 172.67, 172.65, 171.25, 170.34, 169.74, 169.36, 136.25, 128.66, 128.28, 77.74, 74.73, 72.76, 66.19, 56.94, 40.98, 40.10, 39.44, 39.35, 39.26, 34.44, 34.41, 32.03, 31.93, 30.57 (t, J = 21.7 Hz), 29.74, 29.70, 29.67, 29.64, 29.60, 29.56, 29.53, 29.51, 29.47, 29.35, 29.33, 29.29, 29.26, 29.22, 29.12, 27.40, 26.96, 26.92, 26.19, 26.11, 26.00, 25.11, 25.06, 25.05, 25.03, 24.67, 23.25, 22.80, 21.91, 14.23.

^{19}F NMR (376 MHz, $CDCl_3$): δ / ppm = -85.09 (t, J = 9.9 Hz, 3 F, CF_3^{15}), -117.70 – -118.56 (m, 2 F, CF_2^{16}), -126.00 – -126.49 (m, 2 F, CF_2^{16}), -126.86 – -127.42 (m, 2 F, CF_2^{16}), -127.72 – -127.97 (m, 2 F, CF_2^{16}), -130.25 – -130.99 (m, 2 F, CF_2^{16}).Total integral of CF_2 region normalized with respect to the CF_3^{16} group = 10.

ESI-MS [m/z]: $[M + H]^+$ calculated for $^{12}C_{79}{}^{1}H_{124}{}^{16}O_{13}{}^{14}N_3{}^{19}F_{13}$, 1570.8999; found, 1570.8988, Δ = 1.1 mmu.

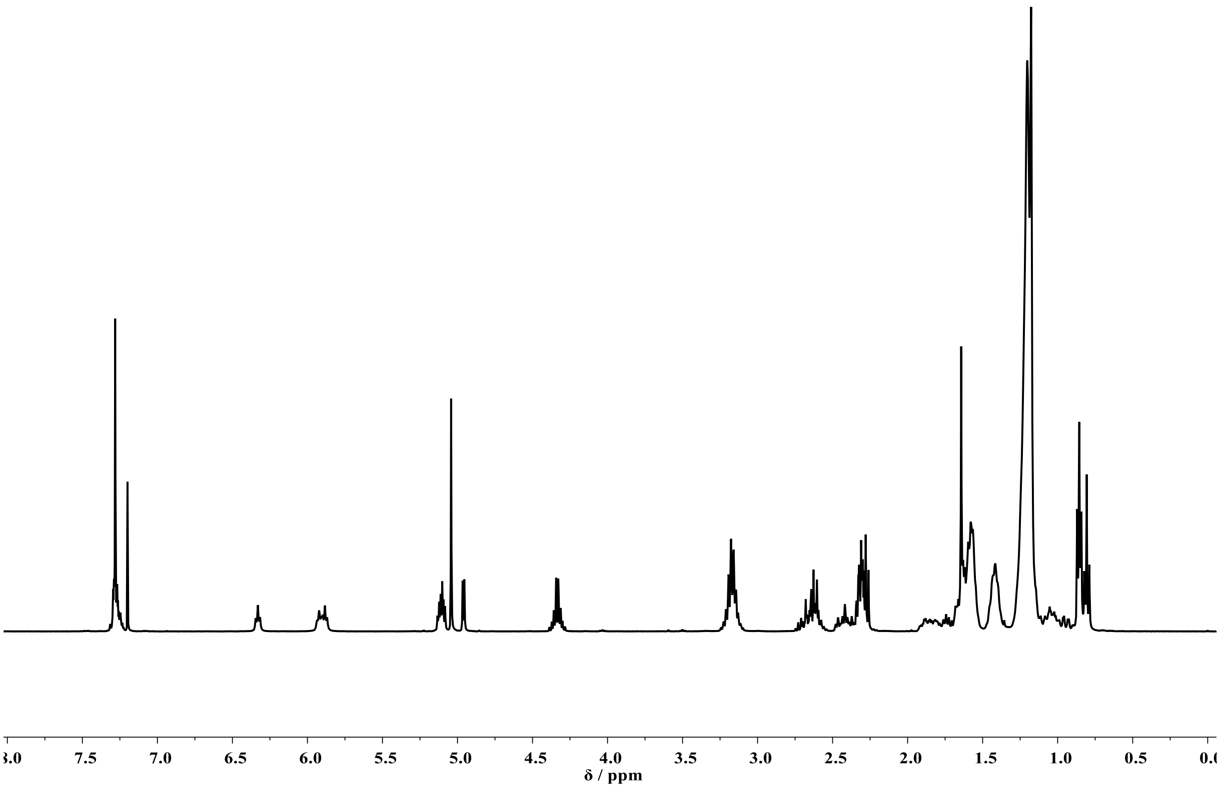

Supplementary Figure 73: ^{1}H-NMR of compound 55 measured in $CDCl_3$.

Deprotection

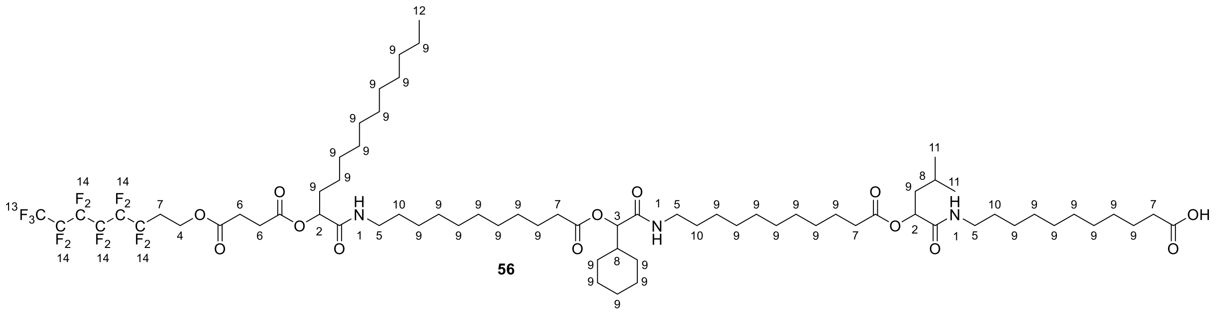

In a 50 mL round bottom flask, 1.51 g of **55** (964 µmol, 1.00 eq.) were dissolved in 7.00 mL ethyl acetate and 7.00 mL THF. Afterwards, 303 mg (20 wt%) palladium on activated charcoal **16** were added. Subsequently, the mixture was purged with hydrogen (3 balloons) and stirred under hydrogen atmosphere overnight. The heterogeneous catalyst was filtered off and the solvent was evaporated under reduced pressure. The product **56** was obtained as a high viscous oil in a yield of 99.5% (1.42 g, 959 µmol).

IR (ATR): ν / cm^{-1} = 3307.3 (vw), 2924.4 (s), 2853.4 (m), 1739.3 (s), 1655.0 (s), 1540.6 (m), 1465.2 (w), 1366.2 (w), 1235.8 (vs), 1144.5 (vs), 842.9 (vw), 808.8 (vw), 697.6 (w), 651.3 (w), 396.6 (vw).

^{1}H NMR (400 MHz, $CDCl_3$): δ / ppm = 6.43 (t, J = 5.8 Hz, 1 H, NH^1), 6.09 – 5.99 (m, 2 H, NH^1), 5.23 – 5.13 (m, 2 H, CH^2), 5.02 (d, J = 4.7 Hz, 1 H, CH^3), 4.46 – 4.33 (m, 2 H, CH_2^4), 3.35 – 3.16 (m, 6 H, CH_2^5), 2.82 – 2.59 (m, 4 H, CH_2^6), 2.55 – 2.28 (m, 8 H, CH_2^7), 2.00 – 1.56 (m, 16 H, CH^8, CH_2^9), 1.54 – 1.41 (m, 6 H, CH_2^{10}), 1.38 – 1.01 (m, 60 H, CH_2^9), 0.91 (t, J = 5.8 Hz, 6 H, CH_3^{11}), 0.89 – 0.83 (m, 3 H, CH_3^{12}).

^{13}C NMR (101 MHz, $CDCl_3$): δ / ppm = 177.49, 172.81, 172.78, 172.68, 171.27, 170.45, 169.88, 169.52, 77.75, 74.71, 72.78, 56.95, 40.93, 40.06, 39.47, 39.34, 39.32, 34.42, 34.40, 34.01, 32.03, 31.92, 30.57 (t, J = 21.7 Hz), 29.74, 29.67, 29.59, 29.56, 29.53, 29.50, 29.46, 29.43, 29.34, 29.29, 29.24, 29.23, 29.12, 29.10, 27.42, 26.95, 26.86, 26.18, 26.09, 25.98, 25.10, 25.07, 25.02, 24.88, 24.67, 23.24, 22.80, 21.90, 14.22.

^{19}F NMR (376 MHz, $CDCl_3$): δ / ppm = -85.10 (t, J = 9.8 Hz, 3 F, CF_3^{13}), -117.52 – -118.47 (m, 2 F, CF_2^{14}), -125.86 – -126.49 (m, 2 F, CF_2^{14}), -126.92 – -127.34 (m, 2 F, CF_2^{14}), -127.44 – -128.06 (m, 2 F, CF_2^{14}), -130.27 – -130.61 (m, 2 F, CF_2^{14}). Total integral of CF_2 region normalized with respect to the CF_3^{13} group = 10.

ESI-MS [*m/z*]: $[M + H]^+$ calculated for $^{12}C_{72}{}^{1}H_{118}{}^{16}O_{13}{}^{14}N_2{}^{19}F_{13}$, 1480.8530; found, 1480.8527, Δ = 0.3 mmu.

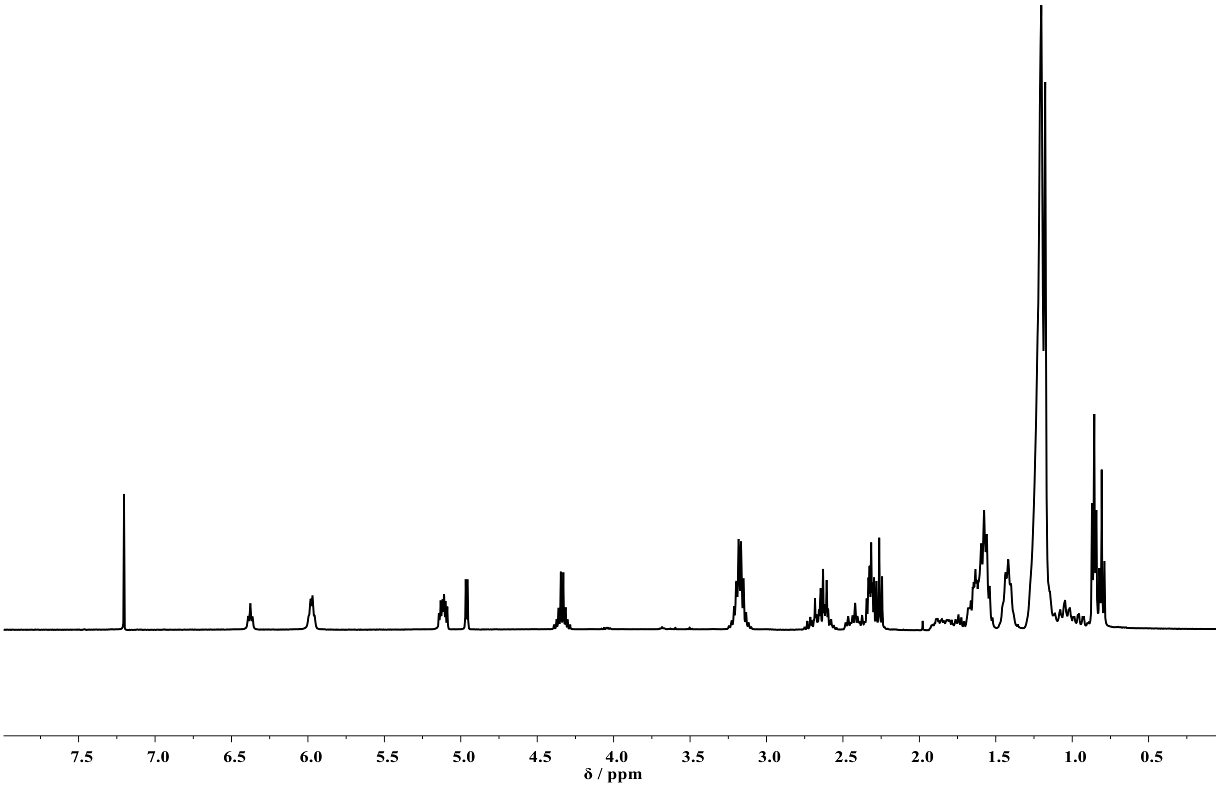

Supplementary Figure 74: ^{1}H-NMR of compound 56 measured in $CDCl_3$.

Passerini reaction

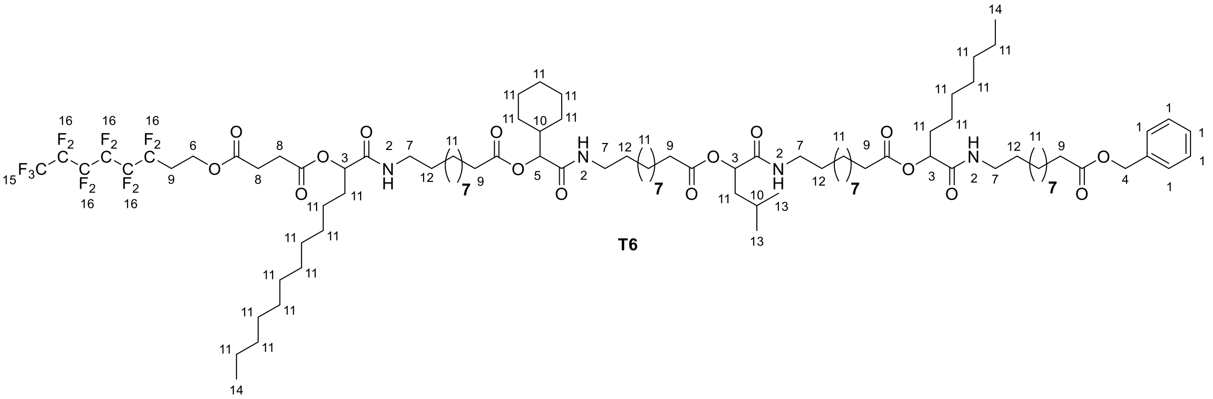

T6

In a 25 mL round bottom flask equipped with a magnetic stir bar, 1.34 g of **56** (853 µmol, 1.00 eq.) was dissolved in 5.00 mL DCM. Afterwards, 200 µL octanal **14i** (164 mg, 1.28 mol, 1.50 eq.) and 386 mg of monomer **M1** (1.28 mmol, 1.50 eq.) were added. The mixture was stirred at room temperature for 3 days and subsequently the solvent was removed under reduced pressure. The crude product was purified by column chromatography (cyclohexane / ethyl acetate 4:1 → 1:1) to afford product **T6** as a white solid in a yield of 89.4% (1.46 g, 763 µmol).

IR (ATR): ν / cm^{-1} = 3302.5 v(w), 2922.1 (s), 2851.9 (m), 2364.7 (vw), 2354.7 (vw), 2324.5 (vw), 1738.1 (s), 1655.8 (vs), 1555.9 (w), 1465.9 (w), 1365.9 (w), 1237.3 (s), 1207.8 (s), 1164.7 (vs), 1144.9 (vs), 1006.8 (vw), 697.6 (w), 653.2 (w), 568.5 (vw), 457.0 (vw), 389.9 (vw).

^{1}H NMR (400 MHz, $CDCl_3$): δ / ppm = 7.41 – 7.28 (m, 5 H, CH_{Ar}^{1}), 6.38 (t, J = 5.7 Hz, 1 H, NH^{2}), 6.09 – 5.88 (m, 3 H, NH^{2}), 5.22 – 5.12 (m, 3 H, CH^{3}), 5.10 (s, 2 H, CH_2^{4}), 5.02 (d, J = 4.6 Hz, 1 H, CH^{5}), 4.47 – 4.32 (m, 2 H, CH_2^{6}), 3.32 – 3.14 (m, 8 H, CH^{7}), 2.85 – 2.59 (m, 4 H, CH_2^{8}), 2.58 – 2.27 (m, 10 H, CH_2^{9}), 1.99 – 1.57 (m, 22 H, CH^{10}, CH_2^{11}), 1.55 – 1.40 (m, 8 H, CH_2^{12}), 1.36 – 1.03 (m, 80 H, CH_2^{11}), 0.92 (t, J = 5.8 Hz, 6 H, CH_3^{13}), 0.89 – 0.80 (m, 6 H, CH_3^{14}).

^{13}C NMR (101 MHz, $CDCl_3$): δ / ppm = 173.81, 172.77, 172.65, 172.64, 172.59, 171.24, 170.35, 169.98, 169.74, 169.35, 136.24, 128.66, 128.28, 77.73, 74.73, 74.06, 72.76, 66.19, 56.94, 40.98, 40.10, 39.44, 39.34, 39.32, 39.26, 34.44, 34.41, 32.03, 31.94, 31.86, 29.74, 29.71, 29.68, 29.61, 29.59, 29.52, 29.49, 29.48, 29.35, 29.23, 29.13, 27.41, 26.96, 26.19, 26.12, 26.00, 25.11, 25.09, 25.08, 25.06, 24.89, 24.67, 23.26, 22.81, 22.74, 21.91, 14.23, 14.21.

^{19}F NMR (376 MHz, $CDCl_3$): δ / ppm = -84.86 – -85.51 (m, 3 F, CF_3^{15}), -117.86 – -118.51 (m, 2 F, CF_2^{16}), -125.81 – -126.56 (m, 2 F, CF_2^{16}), -126.89 – -127.43 (m, 2 F, CF_2^{16}), -127.81 – -128.18 (m, 2 F, CF_2^{16}), -130.22 – -130.82 (m, 2 F, CF_2^{16}). Total integral of CF_2 region normalized with respect to the CF_3^{16} group = 10.

ESI-MS [m/z]: [M + Na]$^{+}$ calculated for $^{12}C_{99}{}^{1}H_{161}{}^{16}O_{16}{}^{14}N_{4}{}^{19}F_{13}$, 1932.1592; found, 1932.1591 Δ = 0.1 mmu.

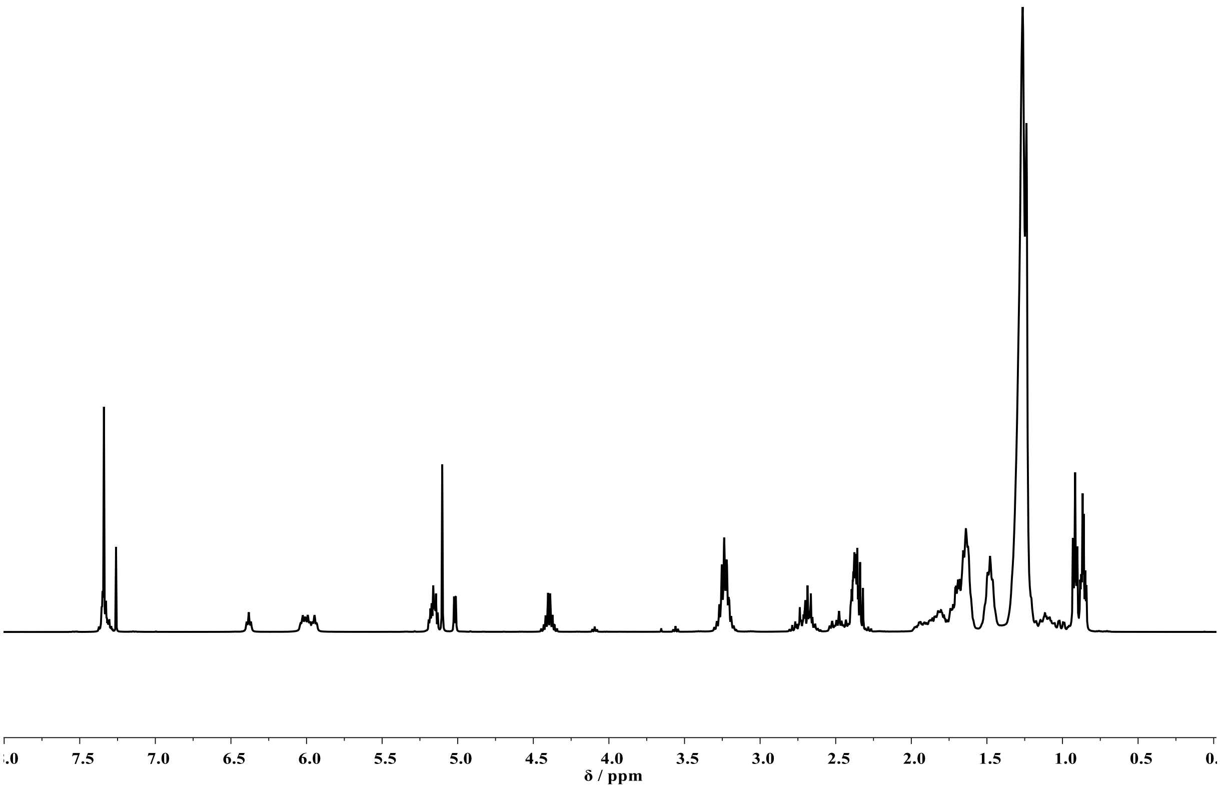

Supplementary Figure 75: ^{1}H-NMR of compound T6 measured in $CDCl_3$.

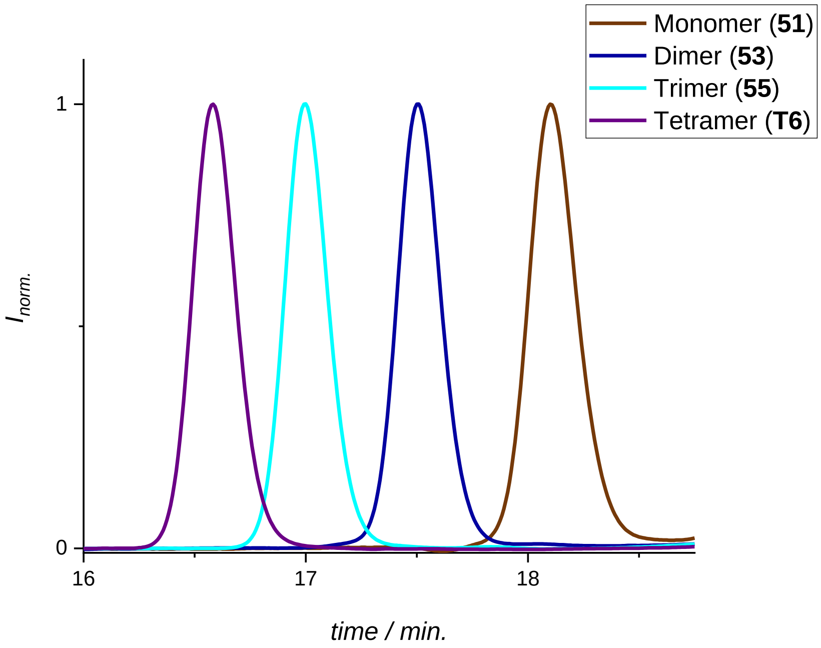

Supplementary Figure 76: SEC traces of the intermediated in the synthesis after each P3CR of product T6

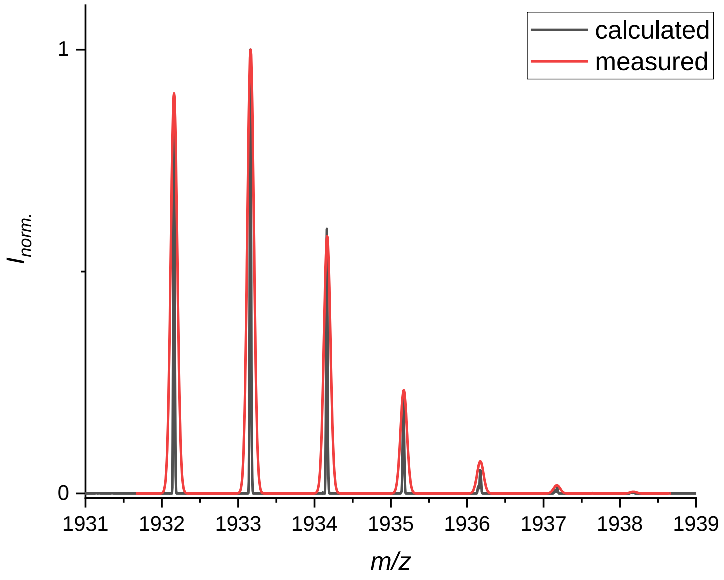

Supplementary Figure 77: High resolution ESI-MS measurement of T6. The observed isotopic pattern is compared with the calculated isotopic pattern obtained from mMass (black).

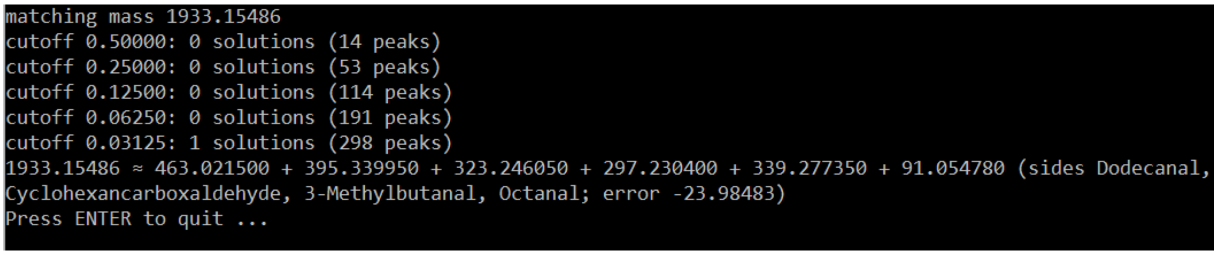

```
matching mass 1933.15486
cutoff 0.50000: 0 solutions (14 peaks)
cutoff 0.25000: 0 solutions (53 peaks)
cutoff 0.12500: 0 solutions (114 peaks)
cutoff 0.06250: 0 solutions (191 peaks)
cutoff 0.03125: 1 solutions (298 peaks)
1933.15486 ≈ 463.021500 + 395.339950 + 323.246050 + 297.230400 + 339.277350 + 91.054780 (sides Dodecanal,
Cyclohexancarboxaldehyde, 3-Methylbutanal, Octanal; error -23.98483)
Press ENTER to quit ...
```

Supplementary Figure 78: Screenshot of the automated read-out of T6, sodium trifluoroacetate was used as additive during the measurement.

1.3.4.4 Synthesis of tetramer T7

Passerini reaction

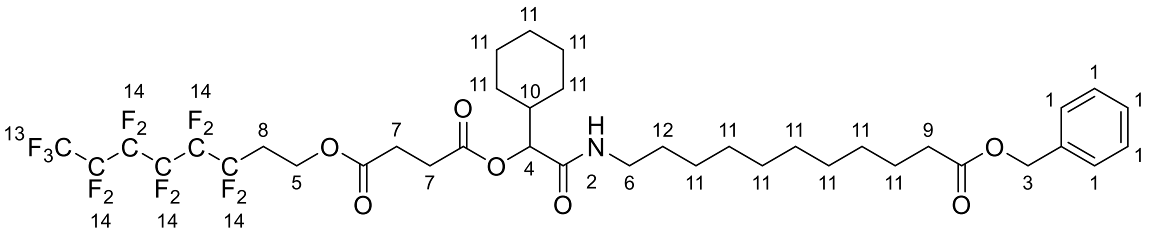

57

In a 50 mL round bottom flask, 300 mg **TAG1** (646 µmol, 1.00 eq.) was stirred in 2.00 mL DCM. Subsequently, 117 µL cyclohexanecarboxaldehyde **14j** (109 mg, 969 µmol, 1.50 eq.) and 292 mg of the monomer **M1** (969 µmol, 1.50 eq.) were added. The resulting reaction mixture was stirred at room temperature for 6 days. Afterwards, the crude mixture was dried under reduced pressure. The residue was adsorbed onto celite® and purified *via* column chromatography on silica gel eluting with a gradual solvent mixture of ethyl acetate and cyclohexane (6:1 → 5:1) to yield the passerini product **57** as a pale highly viscous oil. (489 mg, 557 µmol, 86.2%).

R_f = 0.77 in cyclohexane / ethyl acetate (2:1).

IR (ATR): ν / cm^{-1} = 2927.3 (m), 2854.9 (w), 1737.0 (vs), 1656.6 (m), 1534.7 (w), 1453.1 (w), 1359.5 (w), 1234.2 (vs), 1144.0 (vs), 1082.6 (s), 1002.9 (m), 842.2 (w), 808.9 (w), 733.0 (m), 697.3 (s), 651.4 (m), 566.0 (m).

^{1}H NMR (400 MHz, $CDCl_3$): δ / ppm = 7.43 – 7.30 (m, 5 H, CH_{Ar}^{1}), 6.34 (t, J = 5.7 Hz, 1 H, NH^{2}), 5.11 (s, 2 H, CH_2^{3}), 5.05 (d, J = 3.9 Hz, 1 H, CH^{4}), 4.47 – 4.33 (m, 2 H, CH_2^{5}), 3.35 – 3.13 (m, 2 H, CH_2^{6}), 2.83 – 2.62 (m, 4 H, CH_2^{7}), 2.56 – 2.40 (m, 2 H, CH_2^{8}), 2.34 (t, J = 7.6 Hz, 2 H, CH_2^{9}), 2.08 – 1.96 (m, 1 H, CH^{10}), 1.79 – 1.58 (m, 8 H, CH_2^{11}), 1.55 – 1.47 (m, 2 H, CH_2^{12}), 1.35 – 0.96 (m, 16 H, CH_2^{11}).

^{13}C NMR (101 MHz, $CDCl_3$): δ / ppm = 173.82, 172.69, 171.24, 169.03, 136.26, 128.66, 128.28, 78.44, 66.19, 56.96, 39.88, 39.41, 34.45, 30.57, 29.59, 29.55, 29.48, 29.35, 29.24, 29.17, 29.14, 27.04, 26.98, 26.18, 26.16, 26.04, 25.07.

^{19}F NMR (376 MHz, $CDCl_3$): δ / ppm = -84.01 – -86.07 (m, 3 F, CF_3^{13}), -117.1 – -118.64 (m, 2 F, CF_2^{14}), -126.05 – -126.37 (m, 2 F, CF_2^{14}), -127.08 – -127.35 (m, 2 F, CF_2^{14}), -127.75 – -128.08 (m, 2 F, CF_2^{14}), -130.35 – -130.63 (m, 2 F, CF_2^{14}). Total integral of CF_2 region normalized with respect to the CF_3^{13} group = 10.

ESI-MS [*m/z*]: $[M + H]^+$ calculated for $^{12}C_{38}{}^{1}H_{48}{}^{16}O_{7}{}^{14}N^{19}F_{13}$, 878.3296; found, 878.3271, Δ = 2.5 mmu.

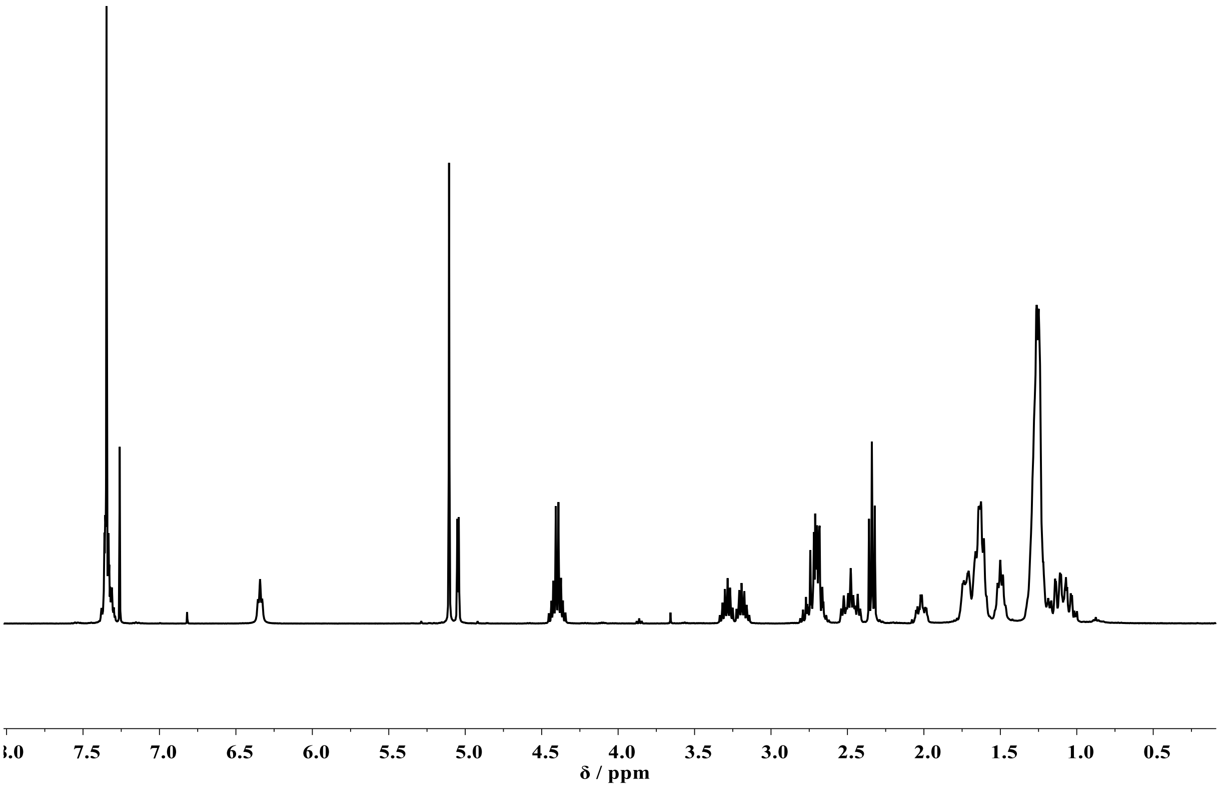

Supplementary Figure 79: ^{1}H-NMR of compound 57 measured in $CDCl_3$.

Deprotection

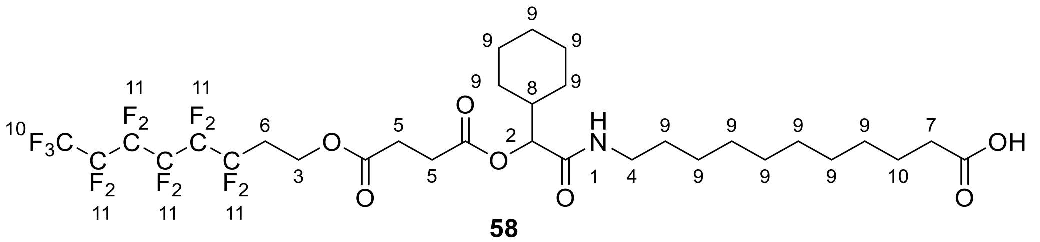

58

In a 50 mL round bottom flask, 419 mg of **57** (478 µmol, 1.00 eq.) were dissolved in 3.00 mL ethyl acetate and 3.00 mL THF. Afterwards, 83.8 mg (20 wt%) palladium on activated charcoal **16** were added. Subsequently, the mixture was purged with hydrogen (3 balloons) and stirred under hydrogen atmosphere overnight. The heterogeneous catalyst was filtered off and the solvent was evaporated under reduced pressure. The product **58** was obtained as a pale highly viscous oil in a yield of 98.7% (372 mg, 472 µmol).

IR (ATR): ν / cm^{-1} = 2927.4 (m), 2855.3 (w), 1737.9 (s), 1650.1 (m), 1541.7 (w), 1451.4 (w), 1362.1 (w), 1233.6 (vs), 1192.2 (vs), 1143.8 (vs), 1082.9 (m), 1003.7 (w), 842.5 (w), 808.9 (w), 732.4 (w), 697.6 (m), 651.3 (w), 566.3 (w), 531.4 (w).

^{1}H NMR (400 MHz, $CDCl_3$): *δ* / ppm = 6.38 (t, *J* = 5.8 Hz, 1 H, NH^{1}), 5.05 (d, *J* = 3.9 Hz, 1 H, CH^{2}), 4.49 – 4.32 (m, 2 H, CH_2^{3}), 3.38 – 3.12 (m, 2 H, CH_2^{4}), 2.84 – 2.62 (m, 4 H, CH_2^{5}), 2.57 – 2.40 (m, 2 H, CH_2^{6}), 2.33 (t, *J* = 7.5 Hz, 2 H, CH_2^{7}), 2.11 – 1.94 (m, 1 H, CH^{8}), 1.78 – 1.68 (m, 2 H, CH_2^{9}), 1.68 – 1.54 (m, 4 H, $CH_2^{9,10}$), 1.56 – 1.43 (m, 2 H, CH_2^{9}), 1.37 – 0.96 (m, 18 H, CH_2^{9}).

^{13}C NMR (126 MHz, $CDCl_3$): *δ* / ppm = 179.59, 173.49, 172.03, 169.93, 79.19, 57.73, 40.61, 40.18, 34.78, 31.34, 30.22, 30.09, 29.98, 29.96, 29.94, 29.90, 29.83, 27.80, 27.67, 26.93, 26.91, 26.78, 25.54.

^{19}F NMR (376 MHz, $CDCl_3$): *δ* / ppm = -85.12 (t, *J* = 9.9 Hz, 3 F, CF_3^{10}), -117.58 – -118.68 (m, 2 F, CF_2^{11}), -125.95 – -126.43 (m, 2 F, CF_2^{11}), -127.08 – -127.31 (m, 2 F, CF_2^{11}), -127.70 – -128.07 (m, 2 F, CF_2^{11}), -130.32 – -131.42 (m, 2 F, CF_2^{11}).Total integral of CF_2 region normalized with respect to the CF_3^{10} group = 10.

ESI-MS [*m/z*]: $[M + H]^+$ calculated for $^{12}C_{31}{}^{1}H_{42}{}^{16}O_7{}^{14}N^{19}F_{13}$, 788.2826; found, 788.2803, Δ = 2.3 mmu.

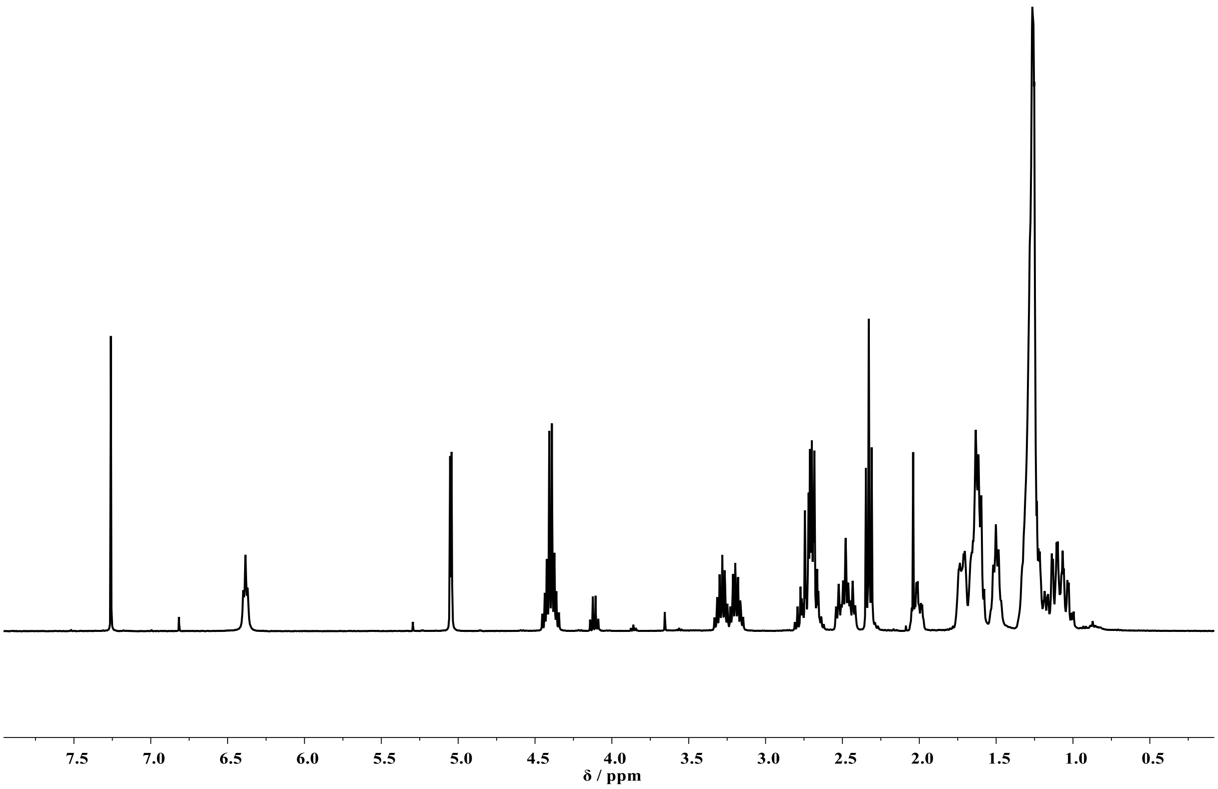

Supplementary Figure 80: ^{1}H-NMR of compound 58 measured in $CDCl_3$.

Passerini reaction

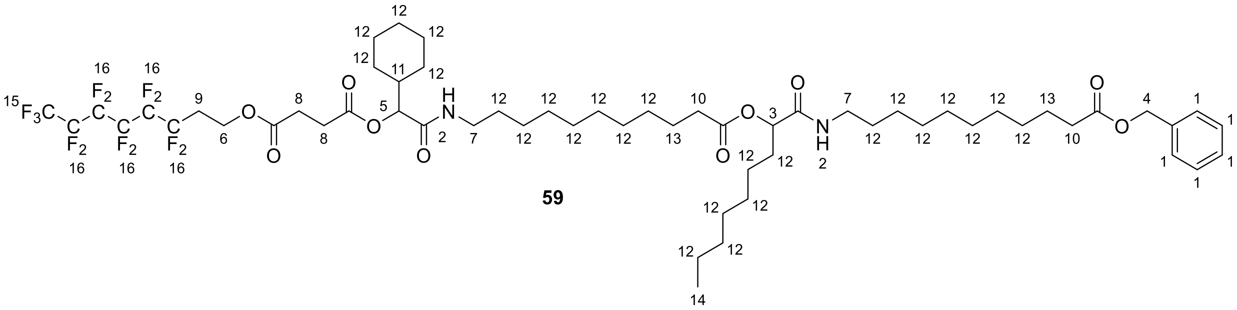

In a 50 mL round bottom flask, 338 mg of **58** (429 µmol, 1.00 eq.) was stirred in 2.00 mL DCM. Subsequently, 101 µL octanal **14i** (109 mg, 644 µmol, 1.50 eq.) and 194 mg of the monomer **M1** (644 µmol, 1.50 eq.) were added. The resulting reaction mixture was stirred at room temperature for 6 days. Afterwards, the crude mixture was dried under reduced pressure. The residue was adsorbed onto celite® and purified *via* column chromatography on silica gel eluting with a gradual solvent mixture of ethyl acetate and cyclohexane (6:1 → 5:1) to yield the passerini product **59** as a pale highly viscous oil. (420 mg, 344 µmol, 80.2%).

R_f = 0.50 in cyclohexane / ethyl acetate (2:1).

IR (ATR): ν / cm^{-1} = 3293.3 (vw), 2918.1 (s), 2851.0 (m), 1736.5 (vs), 1678.6 (m), 1651.2 (s), 1532.6 (m), 1466.9 (w), 1362.3 (w), 1235.6 (vs), 1143.9 (vs), 1082.2 (s), 1005.7 (m), 843.0 (w), 808.4 (w), 733.5 (m), 697.4 (s), 651.3 (w), 567.1 (vw), 531.6 (vw), 445.8 (vw).

^{1}H NMR (400 MHz, $CDCl_3$): δ / ppm = 7.41 – 7.28 (m, 5 H, CH_{Ar}^{1}), 6.35 (t, J = 5.8 Hz, 1 H, NH^{2}), 6.00 (t, J = 5.9 Hz, 1 H, NH^{2}), 5.18 – 5.13 (m, 1 H, CH^{3}), 5.10 (s, 2 H, CH_2^{4}), 5.04 (d, J = 3.9 Hz, 1 H, CH^{5}), 4.48 – 4.33 (m, 2 H, CH_2^{6}), 3.33 – 3.15 (m, 4 H, CH_2^{7}), 2.79 – 2.64 (m, 4 H, CH_2^{8}), 2.54 – 2.41 (m, 2 H, CH_2^{9}), 2.41 – 2.31 (m, 4 H, CH_2^{10}), 2.08 – 1.96 (m, 1 H, CH^{11}), 1.90 – 1.57 (m, 12 H, CH_2^{12}), 1.55 – 1.43 (m, 4 H, CH_2^{13}), 1.36 – 0.99 (m, 38 H, CH_2^{12}), 0.91 – 0.82 (m, 3 H, CH_3^{14}).

^{13}C NMR (101 MHz, $CDCl_3$): δ / ppm = 173.81, 172.69, 172.57, 171.23, 169.97, 169.04, 136.25, 128.66, 128.28, 78.43, 74.05, 66.20, 56.95, 39.87, 39.38, 39.32, 34.45, 32.04, 31.86, 30.57, 29.69, 29.61, 29.58, 29.55, 29.49, 29.48, 29.35, 29.34, 29.24, 29.23, 29.17, 29.13, 27.04, 26.97, 26.96, 26.17, 26.15, 26.03, 25.09, 25.07, 24.88, 22.74, 14.19.

^{19}F NMR (376 MHz, $CDCl_3$): δ / ppm = -85.01 – -85.18 (m, 3 F, CF_3^{15}), -117.85 – -118.19 (m, 2 F, CF_2^{16}), -126.01 – -126.38 (m, 2 F, CF_2^{16}), -127.05 – -127.36 (m, 2 F, CF_2^{16}), -127.74 – -128.06 (m, 2 F, CF_2^{16}), -130.32 – -130.63 (m, 2 F, CF_2^{16}). Total integral of CF_2 region normalized with respect to the CF_3^{15} group = 10.

ESI-MS [*m/z*]: $[M + H]^+$ calculated for $^{12}C_{58}{}^{1}H_{85}{}^{16}O_{10}{}^{14}N_2{}^{19}F_{13}$, 1217.6069; found, 1217.6050, Δ = 1.9 mmu.

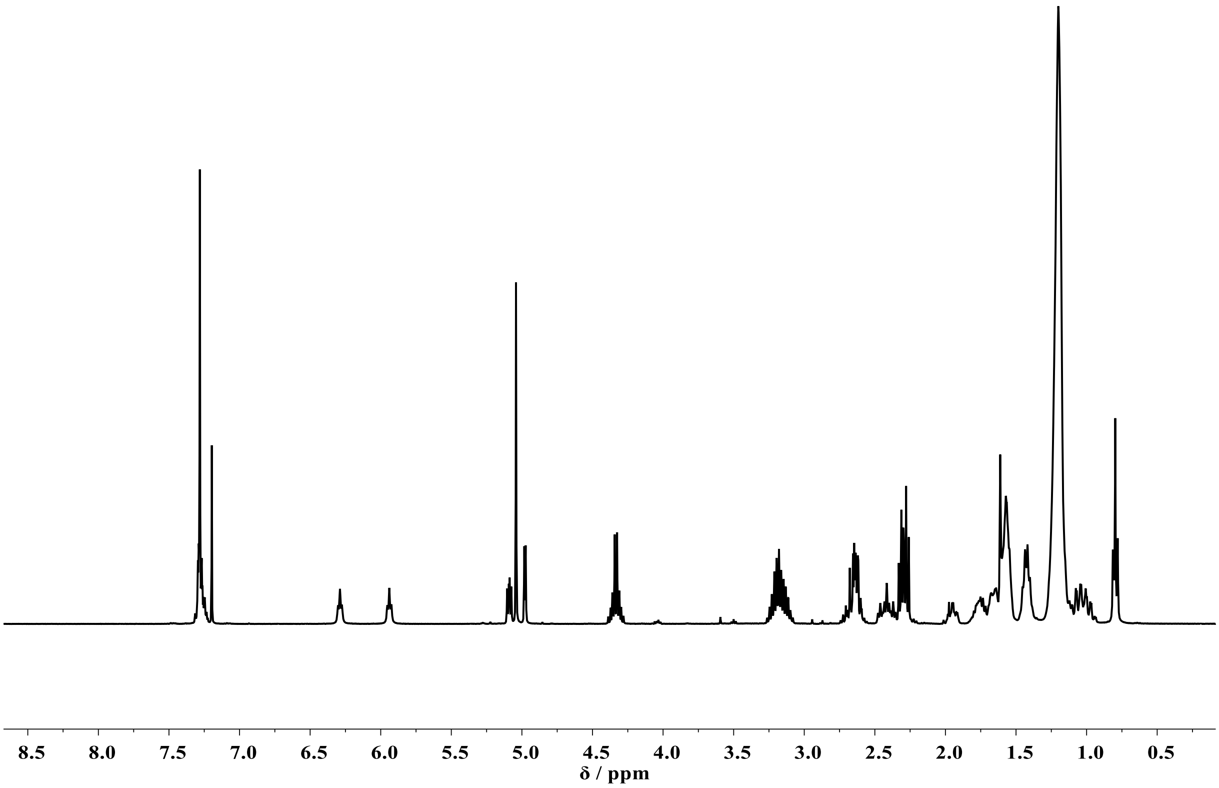

Supplementary Figure 81: ^{1}H-NMR of compound 59 measured in $CDCl_3$.

Deprotection

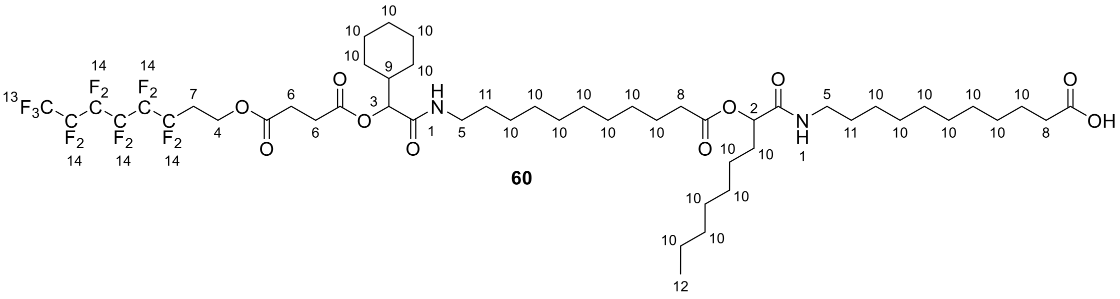

In a 50 mL round bottom flask, 333 mg of **59** (273 µmol, 1.00 eq.) were dissolved in 3.00 mL ethyl acetate and 3.00 mL THF. Afterwards, 66.6 mg (20 wt%) palladium on activated charcoal **16** were added. Subsequently, the mixture was purged with hydrogen (3 balloons) and stirred under hydrogen atmosphere overnight. The heterogeneous catalyst was filtered off and the solvent was evaporated under reduced pressure. The product **60** was obtained as a pale highly viscous oil in a yield of 74.4% (230 mg, 203 µmol).

IR (ATR): ν / cm^{-1} = 2917.1 (s), 2849.7 (m), 1738.4 (vs), 1655.0 (m), 1552.6 (m), 1467.3 (w), 1364.4 (w), 1234.4 (vs), 1190.7 (vs), 1143.9 (vs), 1091.8 (s), 1007.4 (w), 843.1 (vw), 809.9 (vw), 732.7 (w), 697.9 (w), 651.6 (w), 619.5 (w), 531.3 (vw), 445.6 (vw), 388.9 (vw).

^{1}H NMR (400 MHz, $CDCl_3$): δ / ppm = 6.42 (t, J = 5.8 Hz, 1 H, NH^1), 6.04 (t, J = 5.8 Hz, 1 H, NH^1), 5.19 – 5.12 (m, 1 H, CH^2), 5.04 (d, J = 3.9 Hz, 1 H, CH^3), 4.49 – 4.33 (m, 2 H, CH_2^4), 3.34 – 3.13 (m, 4

H, CH_2[5]), 2.83 – 2.62 (m, 4 H, CH_2[6]), 2.55 – 2.27 (m, 6 H, CH_2[8,7]), 2.06 – 1.96 (m, 1 H, CH[9]), 1.91 – 1.55 (m, 12 H, CH_2[10]), 1.54 – 1.42 (m, 4 H, CH_2[11]), 1.38 – 0.98 (m, 38 H, CH_2[10]), 0.89 – 0.83 (m, 3 H, CH_3[12]).

^{13}C NMR (101 MHz, $CDCl_3$): δ / ppm = 178.71, 173.51, 173.37, 172.06, 170.86, 170.01, 79.18, 74.84, 57.73, 40.61, 40.22, 40.07, 35.22, 34.79, 32.77, 32.62, 31.57, 31.35, 31.13, 30.38, 30.34, 30.29, 30.27, 30.23, 30.13, 30.09, 30.01, 29.98, 29.93, 29.90, 29.87, 27.82, 27.74, 27.66, 26.93, 26.91, 26.78, 25.87, 25.64, 23.49.

^{19}F NMR (376 MHz, $CDCl_3$): δ / ppm = -85.11 (t, J = 10.0 Hz, 3 F, CF_3[13]), -117.41 – -118.41 (m, 2 F, CF_2[14]), -125.94 – -126.53 (m, 2 F, CF_2[14]), -126.87 – -127.33 (m, 2 F, CF_2[14]), -127.67 – -128.39 (m, 2 F, CF_2[14]), -130.26 – -130.98 (m, 2 F, CF_2[14]). Total integral of CF_2 region normalized with respect to the CF_3[13] group = 10.

ESI-MS [*m/z*]: $[M + H]^+$ calculated for $^{12}C_{51}{}^{1}H_{79}{}^{16}O_{10}{}^{14}N_2{}^{19}F_{13}$, 1127.5600; found, 1127.5581, Δ = 1.9 mmu.

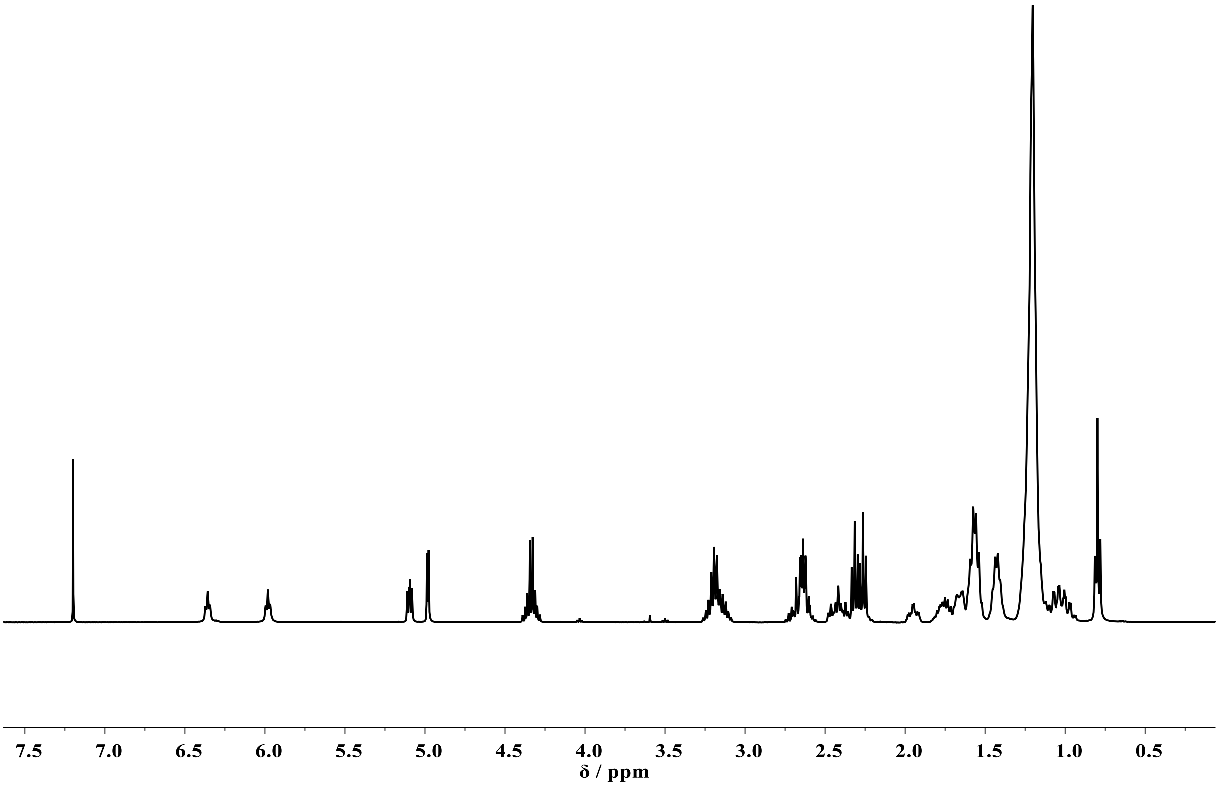

Supplementary Figure 82: ^{1}H-NMR of compound 60 measured in $CDCl_3$.

Passerini reaction

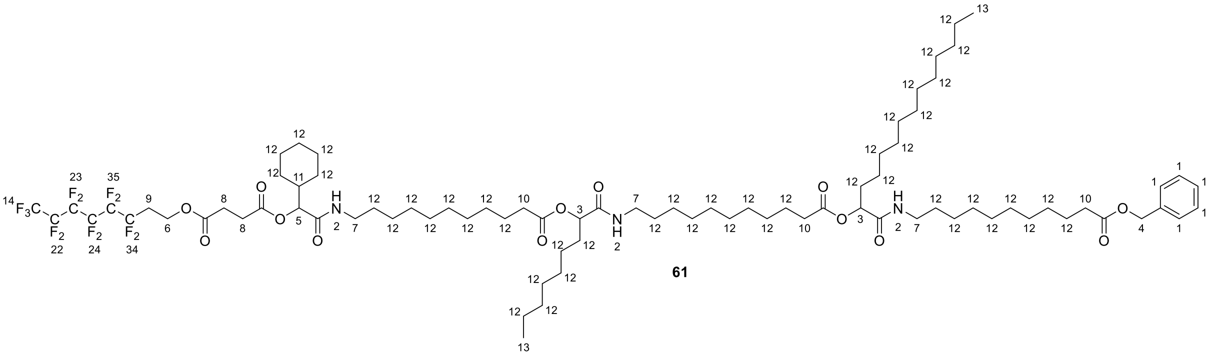

61

In a 50 mL round bottom flask, 252 mg of **60** (224 µmol, 1.00 eq.) was stirred in 2.00 mL DCM. Subsequently, 79.7 µL tridecanal **14d** (66.5 mg, 335 µmol, 1.50 eq.) and 101 mg of the monomer **M1** (335 µmol, 1.50 eq.) were added. The resulting reaction mixture was stirred at room temperature for 3 days. Afterwards, the crude mixture was dried under reduced pressure. The residue was adsorbed onto celite® and purified *via* column chromatography on silica gel eluting with a gradual solvent mixture of ethyl acetate and cyclohexane (4:1 → 2:1) to yield the passerini product **61** as a pale highly viscous oil. (255 mg, 157 µmol, 70.1%).

R_f = 0.15 in cyclohexane / ethyl acetate (2:1).

IR (ATR): ν / cm^{-1} = 3292.0 (w), 2917.4 (vs), 2850.3 (s), 1737.0 (vs), 1654.5 (vs), 1556.9 (m), 1466.7 (w), 1362.8 (w), 1236.8 (vs), 1205.0 (vs), 1144.7 (vs), 1082.6 (m), 1007.7 (w), 809.0 (vw), 696.6 (m), 652.3 (w), 566.2 (vw), 529.3 (vw), 440.8 (vw).

^{1}H NMR (400 MHz, $CDCl_3$): δ / ppm = 7.36 – 7.23 (m, 5 H, $CH_{Ar}{}^{1}$), 6.31 (t, J = 5.8 Hz, 1 H, NH^{2}), 5.97 (t, J = 5.9 Hz, 2 H, NH^{2}), 5.17 – 5.12 (m, 2 H, CH^{3}), 5.04 (s, 2 H, $CH_2{}^{4}$), 4.98 (d, J = 3.9 Hz, 1 H, CH^{5}), 4.42 – 4.25 (m, 2 H, $CH_2{}^{6}$), 3.28 – 3.07 (m, 6 H, $CH_2{}^{7}$), 2.77 – 2.55 (m, 4 H, $CH_2{}^{8}$), 2.49 – 2.21 (m, 8 H, $CH_2{}^{9,10}$), 2.08 – 1.91 (m, 1 H, CH^{11}), 1.90 – 0.94 (m, 92 H, $CH_2{}^{12}$), 0.84 – 0.74 (m, 6 H, $CH_3{}^{13}$).

^{13}C NMR (101 MHz, $CDCl_3$): δ / ppm = 174.59, 173.47, 173.37, 172.02, 170.78, 170.76, 169.83, 137.02, 129.43, 129.05, 79.20, 74.84, 74.82, 66.96, 57.72, 40.64, 40.15, 40.09, 35.21, 32.81, 32.62, 31.55, 31.34, 31.12, 30.56, 30.54, 30.52, 30.47, 30.45, 30.37, 30.34, 30.32, 30.28, 30.25, 30.16, 30.11, 30.01, 29.99, 29.93, 29.90, 27.82, 27.74, 27.73, 26.94, 26.91, 26.80, 25.85, 25.85, 25.66, 23.58, 23.50, 15.01, 14.95.

^{19}F NMR (376 MHz, $CDCl_3$): δ / ppm = -85.10 (t, J = 10.0 Hz, 3 F, $CF_3{}^{14}$), -117.98 – -118.64 (m, 2 F, $CF_2{}^{15}$), -126.01 – -126.41 (m, 2 F, $CF_2{}^{15}$), -126.96 – -127.26 (m, 2 F, $CF_2{}^{15}$), -127.76 – -128.52 (m, 2 F, $CF_2{}^{15}$), -130.27 – -130.87 (m, 2 F, $CF_2{}^{15}$) Total integral of CF_2 region normalized with respect to the $CF_3{}^{14}$ group = 10.

ESI-MS [*m/z*]: $[M + Na]^{+}$ calculated for $^{12}C_{83}{}^{1}H_{132}{}^{16}O_{13}{}^{14}N_{3}{}^{9}F_{13}$, 1648.9445; found, 1648.9487, Δ = 4.2 mmu.

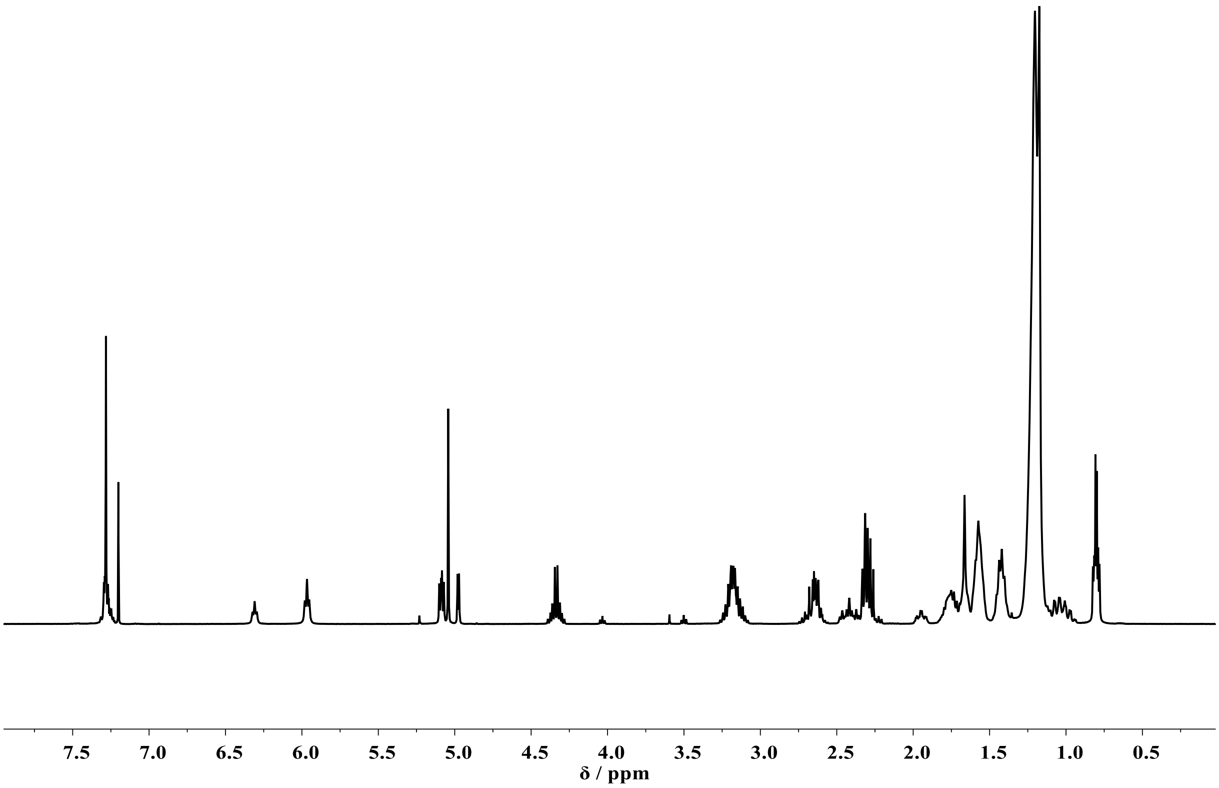

Supplementary Figure 83: ^{1}H-NMR of compound 61 measured in $CDCl_3$.

Deprotection

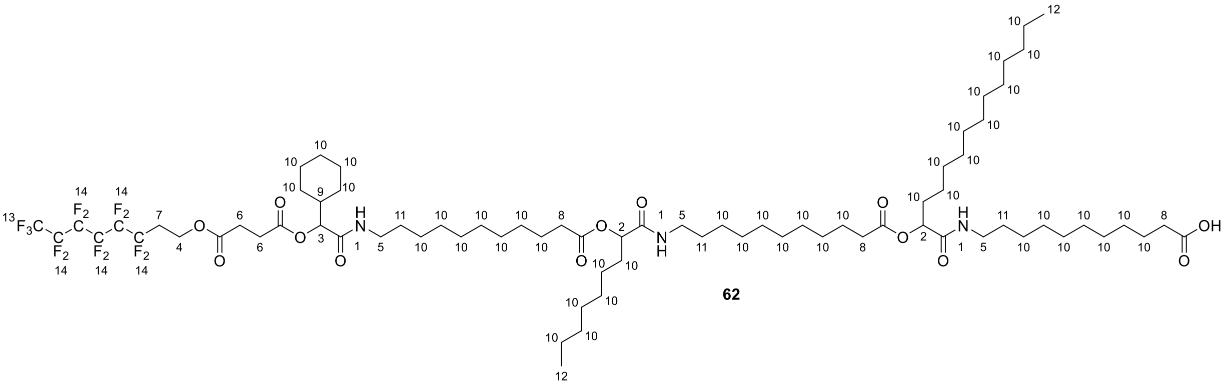

62

In a 25 mL round bottom flask equipped with a magnetic stir bar, 175 mg of **61** (108 µmol, 1.00 eq.) was dissolved in 2.00 mL ethyl acetate and 2.00 mL THF. Subsequently, 35.2 mg (20 wt%) palladium on activated charcoal **16** was added to the solution. The resulting mixture was purged with hydrogen gas and stirred for 1 day at room temperature under hydrogen atmosphere (3 balloons). The heterogeneous catalyst was filtered off and the solvent was evaporated under reduced pressure. The product **62** was obtained as a yellow highly viscous oil in a yield of 97.2% (160 mg, 105 µmol).

IR (ATR): ν / cm^{-1} = 3270.4 (vw), 2917.7 (m), 2850.0 (w), 2355.9 (vw), 2329.9 (vw), 1741.8 (m), 1651.7 (w), 1547.9 (w), 1466.9 (vw), 1365.0 (vw), 1238.2 (m), 1204.7 (m), 1146.3 (m), 1120.5 (w), 809.9 (vw), 721.8 (vw), 653.6 (vw).

^{1}H NMR (400 MHz, $CDCl_3$): δ / ppm = 6.39 (t, J = 5.8 Hz, 1 H, NH^{1}), 6.15 – 6.00 (m, 2 H, NH^{1}), 5.21 – 5.10 (m, 2 H, CH^{2}), 5.04 (d, J = 3.8 Hz, 1 H, CH^{3}), 4.49 – 4.31 (m, 2 H, CH_2^{4}), 3.33 – 3.12 (m, 6 H, CH_2^{5}), 2.82 – 2.65 (m, 4 H, CH_2^{6}), 2.57 – 2.26 (m, 8 H, $CH_2^{7,8}$), 2.06 – 1.96 (m, 1 H, CH^{9}), 1.90 – 1.56 (m, 14 H, CH_2^{10}), 1.54 – 1.43 (m, 6 H, CH_2^{11}), 1.43 – 1.03 (m, 72 H, CH_2^{10}), 0.95 – 0.80 (m, 6 H, CH_3^{12}).

^{13}C NMR (101 MHz, $CDCl_3$): δ / ppm = 177.15, 172.73, 172.70, 172.63, 171.27, 170.19, 170.10, 169.18, 78.42, 74.10, 74.06, 56.97, 39.87, 39.42, 39.39, 39.30, 34.46, 34.45, 33.95, 32.05, 32.01, 31.86, 30.80, 30.58, 30.37, 29.79, 29.78, 29.68, 29.62, 29.57, 29.54, 29.49, 29.44, 29.39, 29.34, 29.27, 29.22, 29.17, 29.14, 29.10, 27.05, 26.97, 26.89, 26.17, 26.15, 26.03, 25.12, 25.08, 24.89, 22.82, 22.74, 14.24, 14.19.

^{19}F NMR (376 MHz, $CDCl_3$): δ / ppm = -83.45 – -86.27 (m, 3 F, CF_3^{13}), -117.14 – -118.77 (m, 2 F, CF_2^{14}), -125.77 – -126.36 (m, 2 F, CF_2^{14}), -126.88 – -127.40 (m, 2 F, CF_2^{14}), -127.40 – -128.01 (m, 2 F, CF_2^{14}), -130.23 – -131.00 (m, 2 F, CF_2^{14}). Total integral of CF_2 region normalized with respect to the CF_3^{13}group = 10.

ESI-MS [*m/z*]: [M + H]$^+$ calculated for $^{12}C_{76}{}^{1}H_{126}{}^{16}O_{13}{}^{14}N_{3}{}^{19}F_{13}$, 1536.9156; found, 1536.9133, Δ = 2.3 mmu.

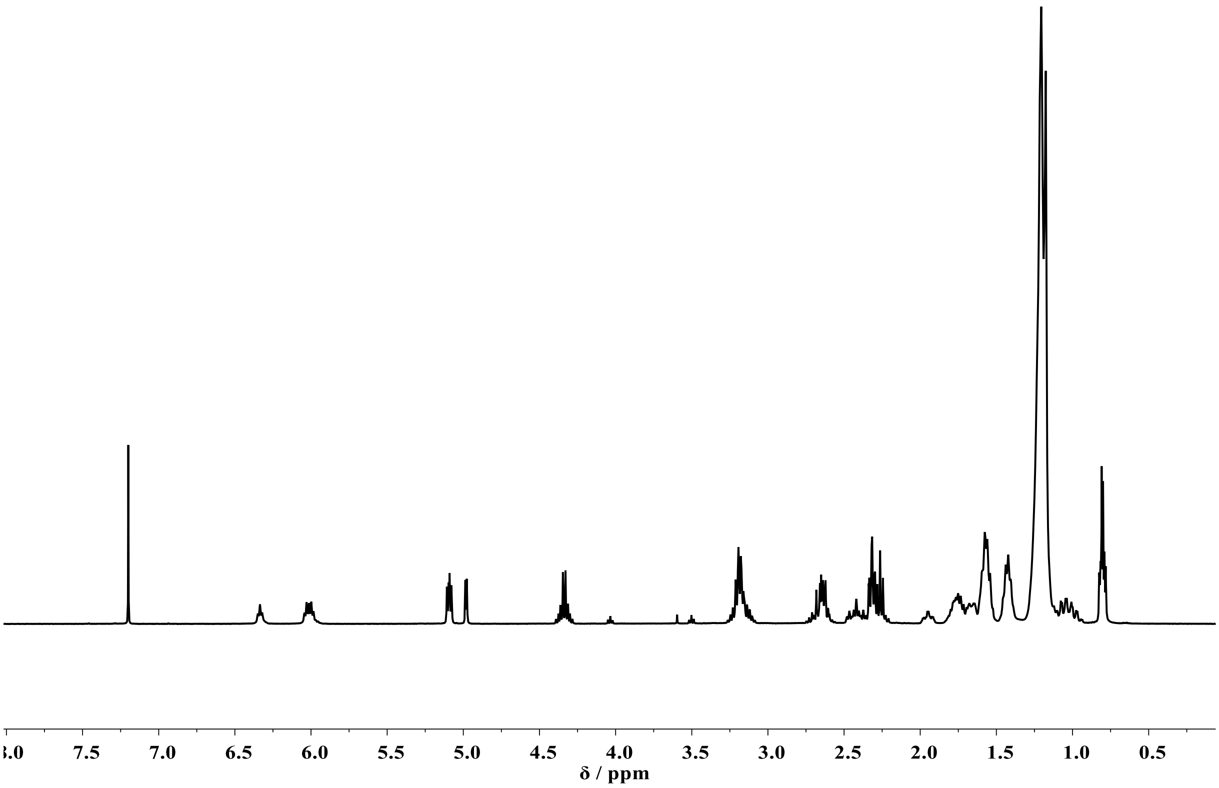

Supplementary Figure 84: ^{1}H-NMR of compound 62 measured in $CDCl_3$.

Passerini reaction

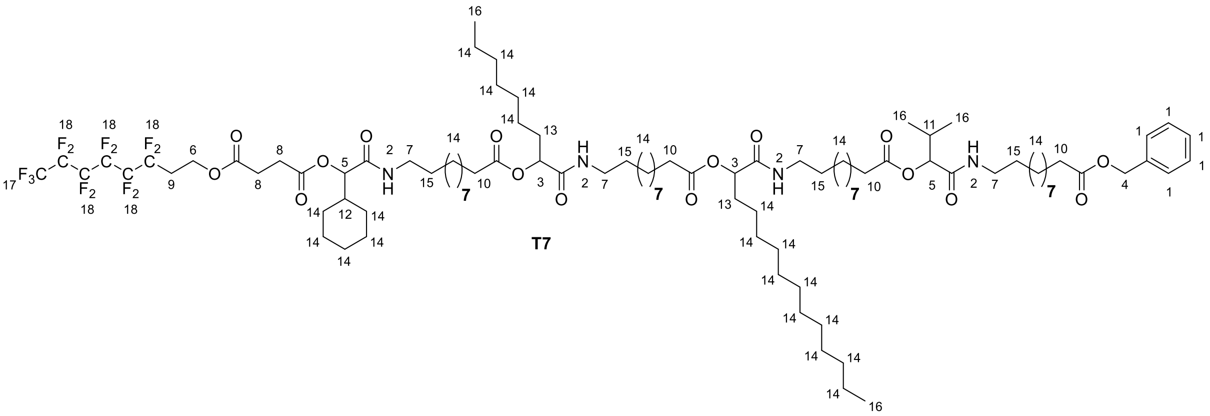

In a 50 mL round bottom flask, 118 mg of **62** (77.0 µmol, 1.00 eq.) was stirred in 2.00 mL DCM. Subsequently, 10.5 µL isobutyraldehyde **14c** (8.30 mg, 115 µmol, 1.50 eq.) and 34.7 mg of the monomer **M1** (115 µmol, 1.50 eq.) were added. The resulting reaction mixture was stirred at room temperature for 3 days. Afterwards, the crude mixture was dried under reduced pressure. The residue was adsorbed onto celite® and purified *via* column chromatography on silica gel eluting with a gradual solvent mixture of ethyl acetate and cyclohexane (3:1 → 1:1) to yield the passerini product **T7** as a pale highly viscous oil. (45.5 mg, 23.9 µmol, 31.0%).

R_f = 0.55 in cyclohexane / ethyl acetate (3:2).

IR (ATR): ν / cm^{-1} = 3292.9 (vw), 2920.8 (m), 2851.5 (m), 1737.1 (m), 1655.5 (m), 1555.5 (w), 1465.7 (w), 1364.5 (vw), 1237.2 (m), 1206.0 (m), 1145.5 (m), 1008.4 (vw), 808.7 (vw), 697.2 (w).

^{1}H NMR (400 MHz, $CDCl_3$): δ / ppm = 7.42 – 7.29 (m, 5 H, CH_{Ar}^{1}), 6.44 – 6.30 (m, 1 H, NH^{2}), 6.11 – 5.92 (m, 3 H, NH^{2}), 5.19 – 5.09 (m, 2 H, CH^{3}), 5.11 (s, 2 H, CH_2^{4}), 5.08 – 5.02 (m, 2 H, CH^{5}), 4.49 – 4.34 (m, 2 H, CH_2^{6}), 3.37 – 3.15 (m, 8 H, CH_2^{7}), 2.82 – 2.63 (m, 4 H, CH_2^{8}), 2.57 – 2.44 (m, 2 H, CH_2^{9}), 2.44 – 2.24 (m, 9 H, CH^{10}, CH_2^{11}), 2.10 – 1.96 (m, 1 H, CH^{12}), 1.90 – 1.57 (m, 20 H, CH_2^{13}, CH_2^{14}), 1.55 – 1.44 (m, 8 H, CH_2^{15}), 1.37 – 1.02 (m, 80, CH_2^{14}), 0.97 – 0.82 (m, 12 H, CH_3^{16}).

^{13}C NMR (101 MHz, $CDCl_3$): δ/ ppm = 173.83, 172.71, 172.71, 172.61, 171.25, 170.01, 169.40, 169.06, 136.24, 128.67, 128.29, 125.65, 78.42, 78.04, 74.05, 74.03, 66.20, 56.95, 39.87, 39.38, 39.31, 39.31, 34.44, 34.42, 32.05, 31.86, 31.57, 30.65, 30.44, 30.31, 29.83, 29.80, 29.78, 29.76, 29.71, 29.68, 29.61, 29.59, 29.57, 29.52, 29.50, 29.49, 29.38, 29.35, 29.33, 29.24, 29.17, 29.13, 27.03, 26.97, 26.17, 26.14, 26.03, 25.14, 25.09, 25.07, 24.91, 24.90, 22.82, 22.74, 18.92, 17.08, 14.26, 14.20.

^{19}F NMR (376 MHz, $CDCl_3$): δ / ppm = -83.70 – -85.90 (m, 3 F, CF_3^{17}), -117.79 – -118.30 (m, 2 F, CF_2^{18}), -125.87 – -126.56 (m, 2 F, CF_2^{18}), -127.01 – -127.46 (m, 2 F, CF_2^{18}), -127.81 – -128.10 (m, 2 F, CF_2^{18}), -130.33 – -130.97 (m, 2 F, CF_2^{18}). Total integral of CF_2 region normalized with respect to the CF_3^{17} group = 10.

ESI-MS [*m/z*]: $[M + H]^+$ calculated for $^{12}C_{99}{}^{1}H_{161}{}^{16}O_{16}{}^{14}N_4{}^{9}F_{13}$, 1910.1773; found, 1910.1826, Δ = 5.3 mmu.

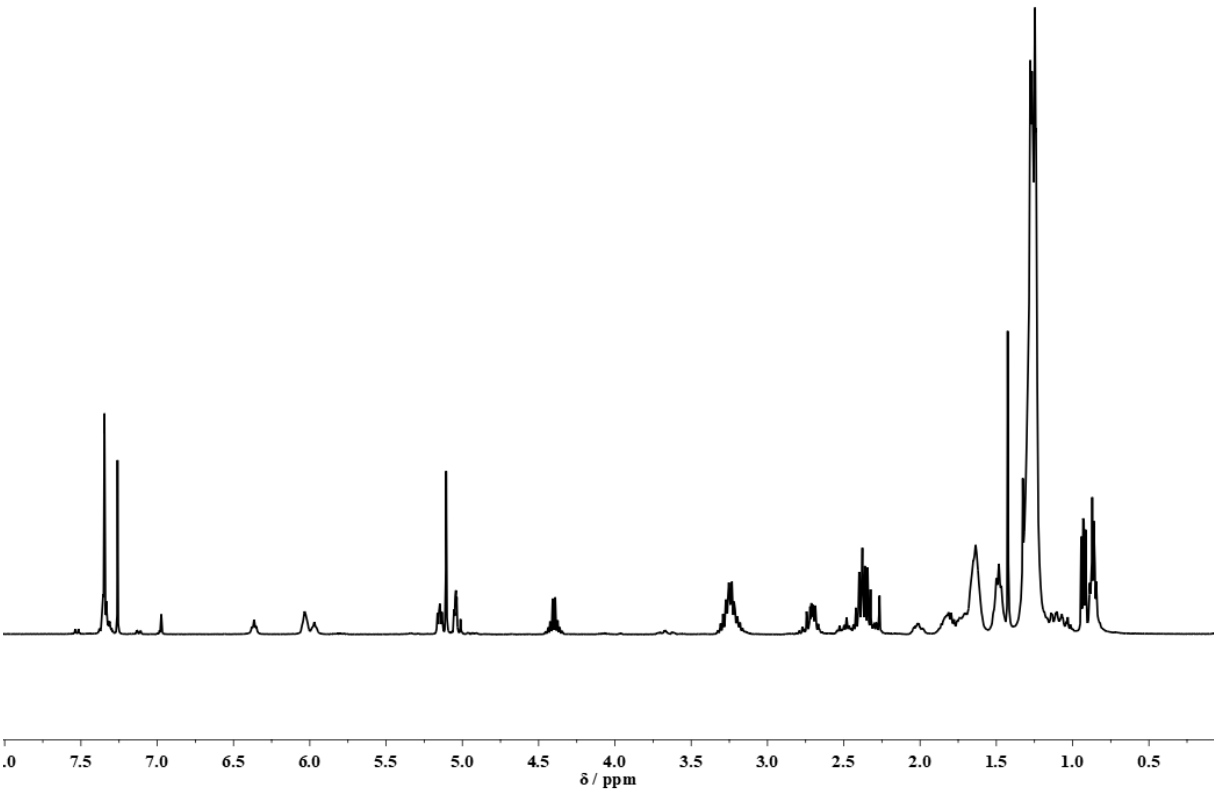

Supplementary Figure 85: ^{1}H-NMR of compound T7 measured in $CDCl_3$.

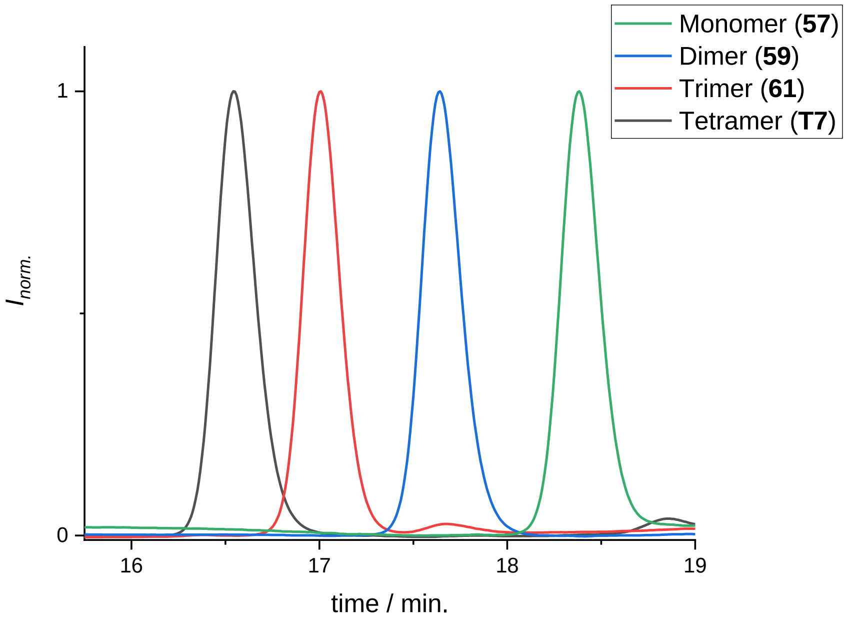

Supplementary Figure 86: SEC traces of the intermediates after each P3CR in the synthesis of product T7

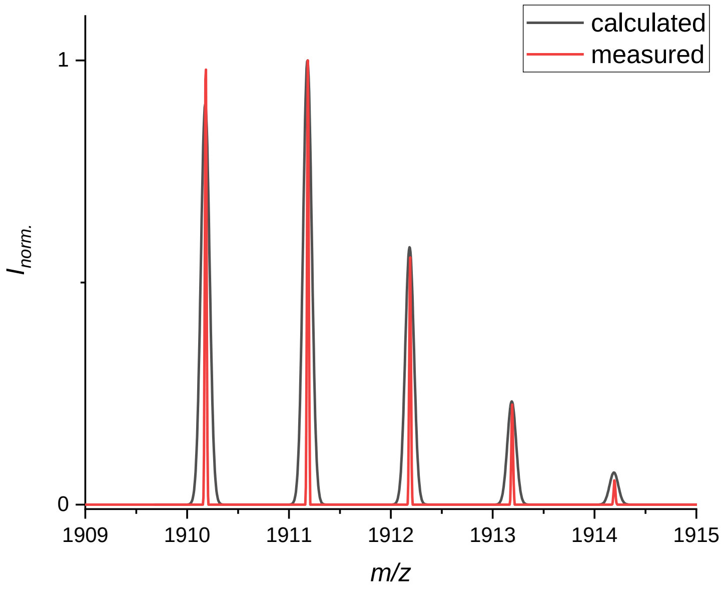

Supplementary Figure 87: High resolution ESI-MS measurement of T7. The observed isotopic pattern is compared with the calculated isotopic pattern obtained from mMass (black).

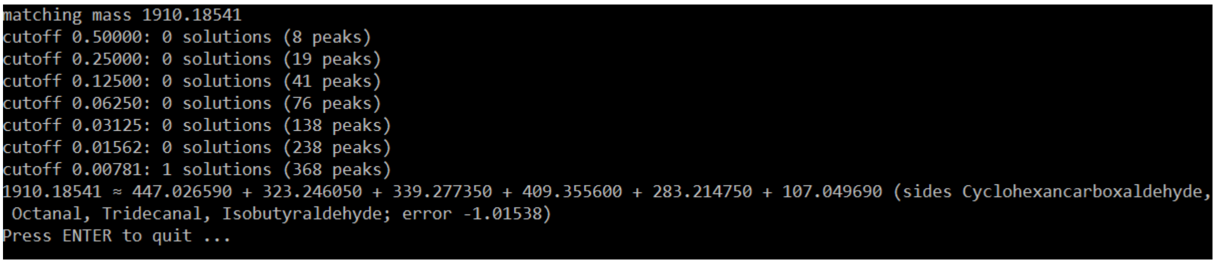

```
matching mass 1910.18541
cutoff 0.50000: 0 solutions (8 peaks)
cutoff 0.25000: 0 solutions (19 peaks)
cutoff 0.12500: 0 solutions (41 peaks)
cutoff 0.06250: 0 solutions (76 peaks)
cutoff 0.03125: 0 solutions (138 peaks)
cutoff 0.01562: 0 solutions (238 peaks)
cutoff 0.00781: 1 solutions (368 peaks)
1910.18541 ≈ 447.026590 + 323.246050 + 339.277350 + 409.355600 + 283.214750 + 107.049690 (sides Cyclohexancarboxaldehyde,
 Octanal, Tridecanal, Isobutyraldehyde; error -1.01538)
Press ENTER to quit ...
```

Supplementary Figure 88: Screenshot of the automated read-out of T7, sodium trifluoroacetate was used as additive during the measurement.

1.3.4.5 Synthesis of tetramer T8

Passerini reaction

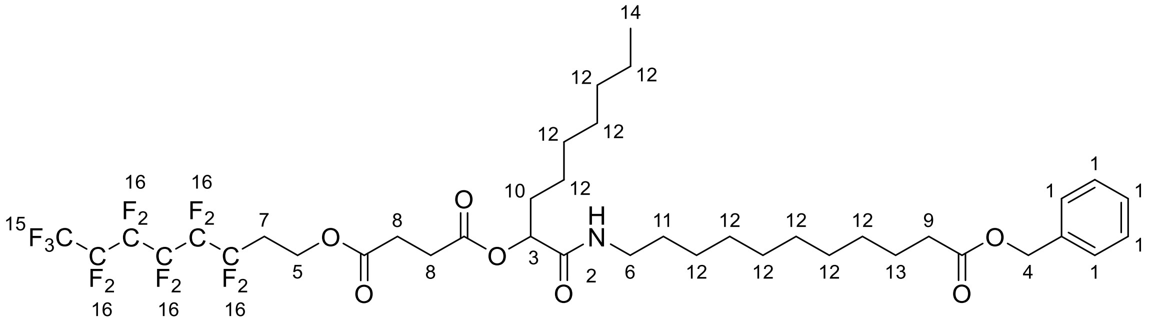

63

In a 50.0 mL round bottom flaks, 300 mg **TAG1** (646 µmol, 1.00 eq.) were dissolved in 2.00 mL DCM and 151 µL octanal **14i** (109 mg, 969 µmol, 1.50 eq.) and 292 mg of monomer **M1** (969 µmol, 1.50 eq.) were added. The mixture was stirred at room temperature for 3 days. Subsequently, the solvent was removed under reduced pressure. The crude product was purified by column chromatography (hexane / ethyl acetate 6:1 → 5:1) to afford product **63** as a yellow oil in a yield of 97.1% (560 mg, 627 µmol).

R_f = 0.40 in cyclohexane / ethyl acetate (3:1).

IR (ATR): ν / cm^{-1} = 2927.0 (m), 2855.8 (w), 1737.6 (s), 1659.3 (w), 1536.1 (w), 1456.7 (vw), 1359.7 (w), 1234.7 (vs), 1190.6 (vs), 1144.1 (vs), 1081.2 (m), 1003.3 (w), 841.5 (vw), 808.5 (vw), 732.4 (w), 696.9 (m), 650.7 (w), 565.4 (vw), 530.2 (vw).

^{1}H NMR (400 MHz, $CDCl_3$): δ / ppm = 7.43 – 7.28 (m, 5 H, CH_{Ar}^{1}), 6.37 (t, J = 5.8 Hz, 1 H, NH^{2}), 5.17 (q, J = 7.6, 4.3 Hz, 1 H, CH^{3}), 5.11 (s, 2 H, CH_2^{4}), 4.47 – 4.33 (m, 2 H, CH_2^{5}), 3.34 – 3.14 (m, 2 H, CH_2^{6}), 2.85 – 2.60 (m, 2 H, CH_2^{7}), 2.55 – 2.40 (m, 4 H, CH_2^{8}), 2.34 (t, J = 7.6 Hz, 2 H, CH_2^{9}), 1.95 – 1.74 (m, 2 H, CH_2^{10}), 1.69 – 1.58 (m, 4 H, $CH_2^{11,12}$), 1.55 – 1.43 (m, 2 H, CH_2^{13}), 1.38 – 1.21 (m, 20 H, CH_2^{12}), 0.86 (t, J = 7.0 Hz, 3 H, CH_3^{14})

^{13}C NMR (101 MHz, $CDCl_3$): δ / ppm = 173.82, 172.64, 171.24, 169.71, 136.27, 128.67, 128.29, 76.84, 66.20, 56.96, 39.47, 34.45, 31.94, 31.88, 30.80, 30.59, 30.37, 29.60, 29.54, 29.50, 29.39, 29.35, 29.33, 29.25, 29.23, 29.15, 26.98, 25.07, 25.02, 22.74.

^{19}F NMR (376 MHz, $CDCl_3$): δ / ppm = -83.86 – -86.34 (m, 3 F, CF_3^{15}), -117.65 – -118.28 (m, 2 F, CF_2^{16}), -125.99 – -126.38 (m, 2 F, CF_2^{16}), -127.05 – -127.38 (m, 2 F, CF_2^{16}), -127.71 – -128.09 (m, 2 F, CF_2^{16}), -130.31 – -130.71 (m, 2 F, CF_2^{16}). Total integral of CF_2 region normalized with respect to the CF_3^{15} group = 10.

ESI-MS [*m/z*]: $[M + H]^+$ calculated for $^{12}C_{39}{}^{1}H_{52}{}^{16}O_7{}^{14}N^{19}F_{13}$, 894.3609; found, 894.3597, Δ = 1.2 mmu.

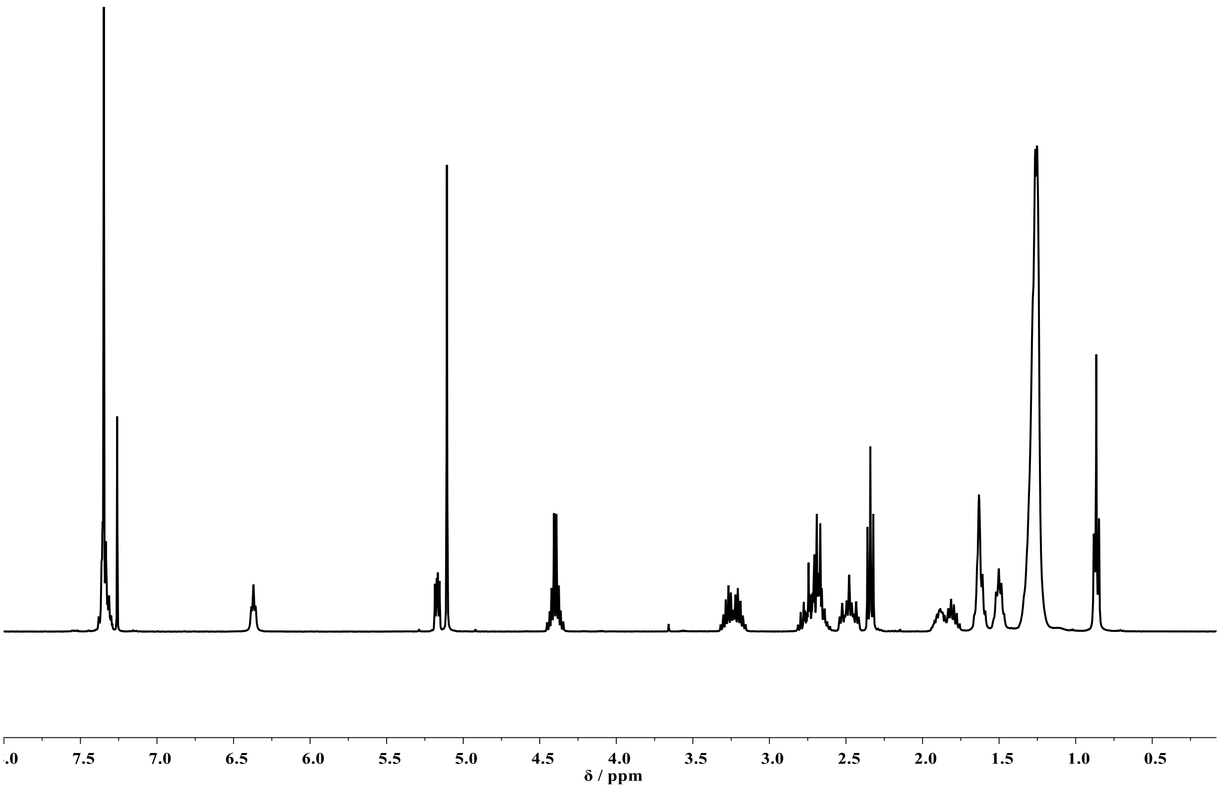

Supplementary Figure 89: ¹H-NMR of compound 63 measured in CDCl3.

Deprotection

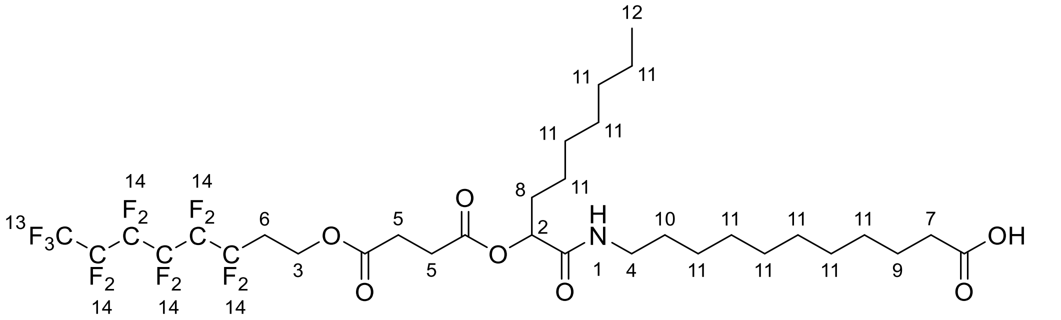

64

In a 25 mL round bottom flask equipped with a magnetic stir bar, 457 mg of the **63** (511 µmol, 1.00 eq.) was dissolved in 2.00 mL ethyl acetate and 2.00 mL THF. Subsequently, 91.4 mg (20 wt%) palladium on activated charcoal **16** was added to the solution. The resulting mixture was purged with hydrogen gas and stirred for 5 days at room temperature under hydrogen atmosphere (3 balloons). The heterogeneous catalyst was filtered off and the solvent was evaporated under reduced pressure. The product **64** was obtained as a yellow highly viscous oil in a yield of 94.3%. (388 mg, 482 µmol).

IR (ATR): ν / cm^{-1} = 3296.2 (w), 2923.7 (s), 2851.9 (m), 1742.9 (vs), 1696.1 (s), 1653.5 (s), 1560.3 (w), 1468.4 (w), 1411.5 (w), 1358.8 (m), 1233.0 (vs), 1189.4 (vs), 1141.7 (vs), 1082.3 (vs), 1009.6 (w), 938.0 (w), 841.3 (w), 698.3 (vs), 651.3 (s), 567.5 (w), 528.7 (w), 437.6 (w), 389.0 (vw).

^{1}H NMR (400 MHz, $CDCl_3$): *δ* / ppm = 6.35 (t, *J* = 5.9 Hz, 1 H, NH^1), 5.19 – 5.01 (m, 1 H, CH^2), 4.41 – 4.23 (m, 2 H, CH_2^3), 3.34 – 3.02 (m, 2 H, CH_2^4), 2.77 – 2.54 (m, 4 H, CH_2^5), 2.49 – 2.35 (m, 2 H, CH_2^6), 2.30 – 2.23 (m, 2 H, CH_2^7), 1.90 – 1.67 (m, 2 H, CH_2^8), 1.61 – 1.49 (m, 2 H, CH_2^9), 1.49 – 1.39 (m, 2 H, CH_2^{10}) 1.34 – 1.14 (m, 22 H, CH_2^{11}), 0.80 (t, *J* = 6.7 Hz, 3 H, CH_3^{12}).

^{13}C NMR (101 MHz, $CDCl_3$): *δ* / ppm = 173.43, 172.02, 170.60, 116.31, 75.50, 57.72, 40.23, 34.77, 32.67, 32.63, 31.56, 31.34, 31.13, 30.20, 30.10, 30.08, 29.98, 29.90, 29.83, 27.66, 25.77, 25.55, 23.49, 14.93.

^{19}F NMR (376 MHz, $CDCl_3$): *δ* / ppm = -85.01 – -85.29 (m, 3 F, CF_3^{13}), -117.90 – -118.20 (m, 2 F, CF_2^{14}), -125.96 – -126.43 (m, 2 F, CF_2^{14}), -126.99 – -127.42 (m, 2 F, CF_2^{14}), -127.82 – -128.11 (m, 2 F, CF_2^{14}), -130.29 – -130.76 (m, 2 F, CF_2^{14}). Total integral of CF_2 region normalized with respect to the CF_3^{13} group = 10.

ESI-MS [*m/z*]: $[M + H]^+$ calculated for $^{12}C_{32}{}^{1}H_{46}{}^{16}O_7{}^{14}N^{19}F_{13}$, 804.3139; found, 804.3115, Δ = 2.4 mmu.

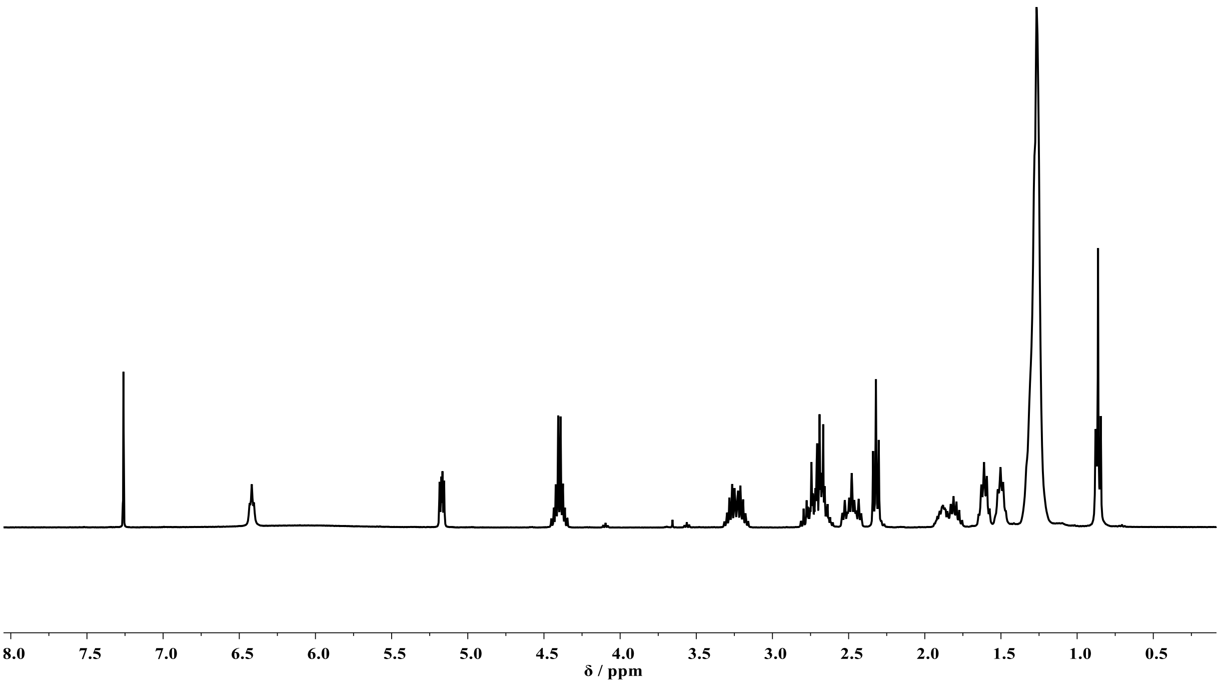

Supplementary Figure 90: ^{1}H-NMR of compound 64 measured in $CDCl_3$.

Passerini reaction

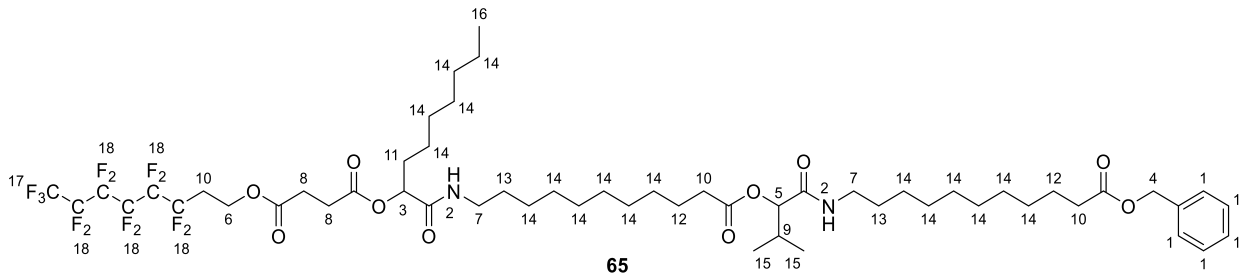

65

In a 50 mL round bottom flask, 413 mg of **64** (514 µmol, 1.00 eq.) was dissolved in 2.00 mL DCM and 70.4 µL isobutyraldehyde **14c** (55.6 mg, 772 µmol, 1.50 eq.) and 233 mg of monomer **M1** (772 mmol, 1.50 eq.) were added. The mixture was stirred at room temperature for 3 days. Subsequently, the solvent was removed under reduced pressure. The crude product was purified by column chromatography (cyclohexane / ethyl acetate 5:1 →1:1) to afford product **65** as a pale highly viscous oil in a yield of 96.5% (555 mg, 496 µmol).

R_f = 0.20 in cyclohexane / ethyl acetate (3:1).

IR (ATR): ν / cm^{-1} = 3315.8 (vw), 2923.9 (m), 2852.2 (w), 1736.6 (vs), 1656.1 (vs), 1548.7 (w), 1466.1 (w), 1363.6 (w), 1236.4 (vs), 1144.2 (vs), 1009.0 (m), 841.8 (w), 809.6 (vw), 732.3 (w), 697.3 (m), 652.0 (w), 567.3 (vw).

^{1}H NMR (400 MHz, $CDCl_3$): δ / ppm = 7.41 – 7.28 (m, 5 H, CH_{Ar}^{1}), 6.39 (t, J = 5.8 Hz, 1 H, NH^{2}), 5.94 (t, J = 5.9 Hz, 1 H, NH^{2}), 5.20 – 5.14 (m, 1 H, CH^{3}), 5.11 (s, 2 H, CH^{4}), 5.05 (d, J = 4.4 Hz, 1 H, CH^{5}), 4.46 – 4.34 (m, 2 H, CH^{6}), 3.36 – 3.15 (m, 4 H, CH^{7}), 2.83 – 2.60 (m, 4 H, CH_2^{8}), 2.56 – 2.24 (m, 7 H, CH^{9}, CH_2^{10}), 1.97 – 1.74 (m, 2 H, CH_2^{11}), 1.72 – 1.61 (m, 4 H, CH_2^{12}), 1.55 – 1.45 (m, 4 H, CH_2^{13}), 1.38 – 1.20 (m, 34 H, CH_2^{14}), 0.95 – 0.90 (m, 6 H, CH_3^{15}), 0.89 – 0.84 (m, 3 H, CH_3^{16}).

^{13}C NMR (101 MHz, $CDCl_3$): δ / ppm = 173.82, 172.66, 172.64, 171.24, 169.72, 169.39, 136.25, 128.66, 128.28, 78.03, 74.74, 66.20, 56.94, 39.44, 39.29, 34.45, 34.42, 31.93, 31.87, 30.65, 29.72, 29.60, 29.56, 29.54, 29.50, 29.47, 29.35, 29.33, 29.26, 29.23, 29.14, 26.97, 25.14, 25.07, 25.02, 22.74, 18.90, 17.05, 14.19.

^{19}F NMR (376 MHz, $CDCl_3$): δ / ppm = -84.77 – -85.55 (m, 3 F, CF_3^{17}), -117.72 – -118.51 (m, 2 F, CF_2^{18}), -126.00 – -126.49 (m, 2 F, CF_2^{18}), -126.94 – -127.43 (m, 2 F, CF_2^{18}), -127.58 – -128.18 (m, 2 F, CF_2^{18}), -129.96 – -130.99 (m, 2 F, CF_2^{18}). Total integral of CF_2 region normalized with respect to the CF_3^{17} group = 10.

ESI-MS [m/z]: $[M + Na]^+$ calculated for $^{12}C_{55}{}^{1}H_{81}{}^{16}O_{10}{}^{14}N_2{}^{19}F_{13}$, 1199.5576; found, 1199.5555, Δ = 2.1 mmu.

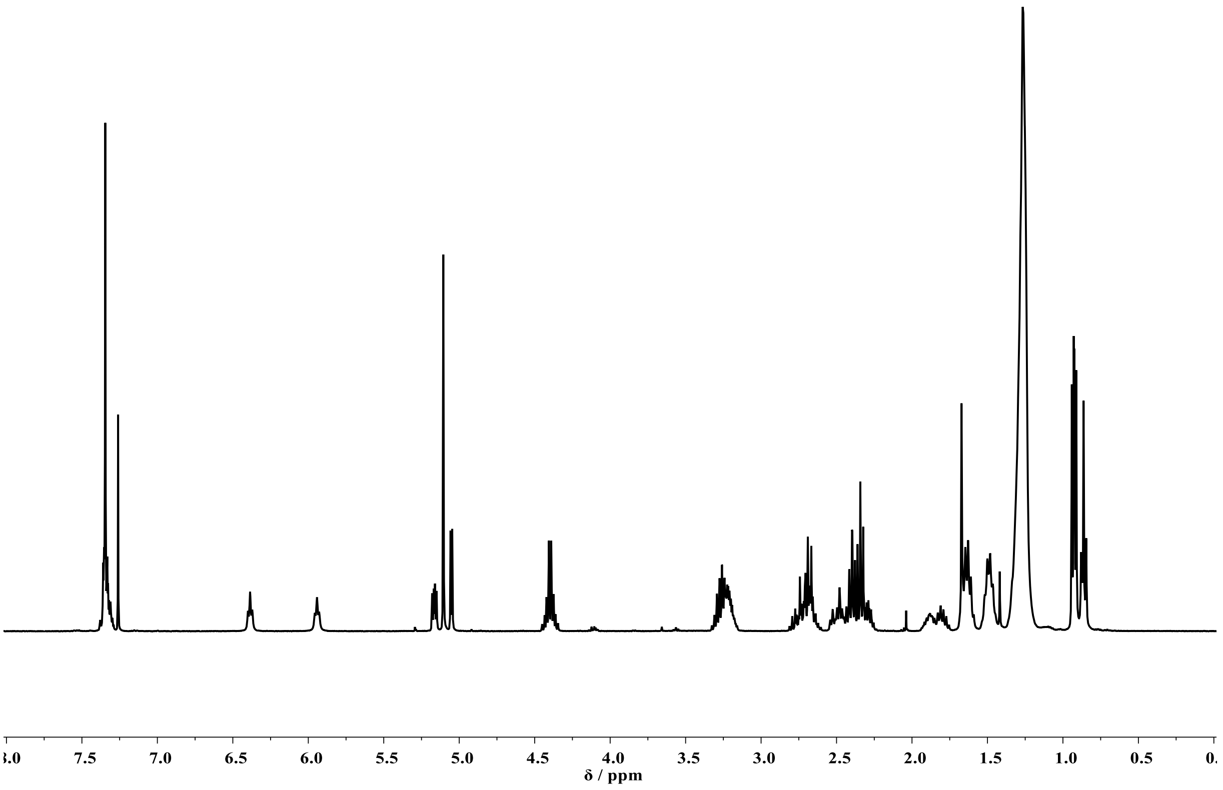

Supplementary Figure 91: ^{1}H-NMR of compound 65 measured in $CDCl_3$.

Deprotection

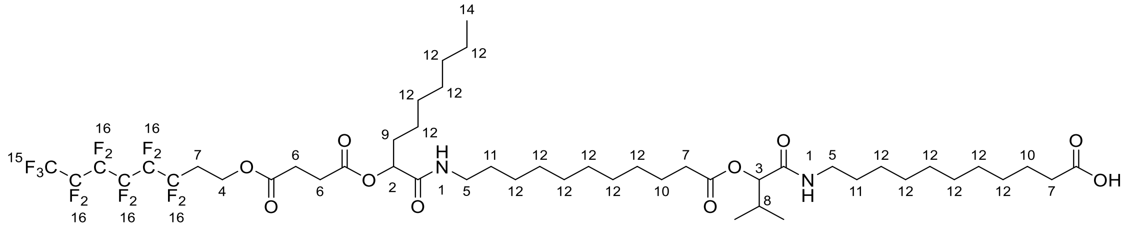

66

In a 25 mL round bottom flask, 505 mg of **65** (429 µmol, 1.00 eq.) was dissolved in 2.00 mL ethyl acetate and 2.00 mL THF. Subsequently, 101 mg (20 wt%) palladium on activated charcoal **16** was added to the solution. The resulting mixture was purged with hydrogen gas and stirred for 1 day at room temperature under hydrogen atmosphere (3 balloons). The heterogeneous catalyst was filtered off and the solvent was evaporated under reduced pressure. The product **66** was obtained as a yellow highly viscous oil in a yield of 97.4%. (445 mg, 418 µmol).

IR (ATR): ν / cm^{-1} = 3304.8 (vw), 2925.6 (m), 2854.6 (m), 1738.4 (s), 1654.9 (s), 1540.6 (w), 1464.3 (w), 1365.1 (w), 1235.0 (vs), 1144.6 (vs), 1007.2 (w), 841.4 (vw), 808.8 (w), 697.7 (w), 651.2 (w).

^{1}H NMR (400 MHz, $CDCl_3$): δ / ppm = 6.46 (t, J = 5.8 Hz, 1 H, NH^1), 5.98 (t, J = 5.9 Hz, 1 H, NH^1), 5.21 – 5.12 (m, 1 H, CH^2), 5.06 (d, J = 4.4 Hz, 1 H, CH^3), 4.48 – 4.32 (m, 2 H, $CH_2{}^4$), 3.34 – 3.15 (m, 4

H, CH_2^5), 2.83 – 2.58 (m, 4 H, CH_2^6), 2.56 – 2.20 (m, 7 H, CH^7, CH_2^8), 1.95 – 1.72 (m, 2 H, CH_2^9), 1.70 – 1.55 (m, 4 H, CH_2^{10}), 1.54 – 1.40 (m, 4 H, CH_2^{11}), 1.39 – 1.14 (m, 34 H, CH_2^{12}), 0.97 – 0.90 (m, 6 H, CH_3^{13}), 0.89 – 0.78 (m, 3 H, CH_3^{14}).

^{13}C NMR (101 MHz, $CDCl_3$): δ / ppm = 172.72, 171.32, 169.97, 169.52, 78.07, 74.73, 56.98, 39.52, 39.26, 34.43, 34.01, 31.90, 31.87, 30.62, 29.62, 29.58, 29.51, 29.41, 29.37, 29.35, 29.32, 29.29, 29.21, 29.14, 29.08, 26.97, 26.89, 25.16, 25.02, 24.88, 22.74, 18.90, 17.05, 14.18.

^{19}F NMR (376 MHz, $CDCl_3$): δ / ppm = -85.09 (t, J = 9.9 Hz, 3 F, CF_3^{13}), -117.74 – -118.31 (m, 2 F, CF_2^{14}), -125.91 – -126.37 (m, 2 F, CF_2^{14}), -126.61 – -127.43 (m, 2 F, CF_2^{14}), -127.48 – -128.13 (m, 2 F, CF_2^{14}), -130.00 – -130.75 (m, 2 F, CF_2^{14}). Total integral of CF_2 region normalized with respect to the CF_3^{13} group = 10.

ESI-MS [*m/z*]: [M + Na]$^+$ calculated for $^{12}C_{48}{}^{1}H_{75}{}^{16}O_{10}{}^{14}N_2{}^{19}F_{13}$, 1109.5106; found, 1109.5092, Δ = 1.4 mmu.

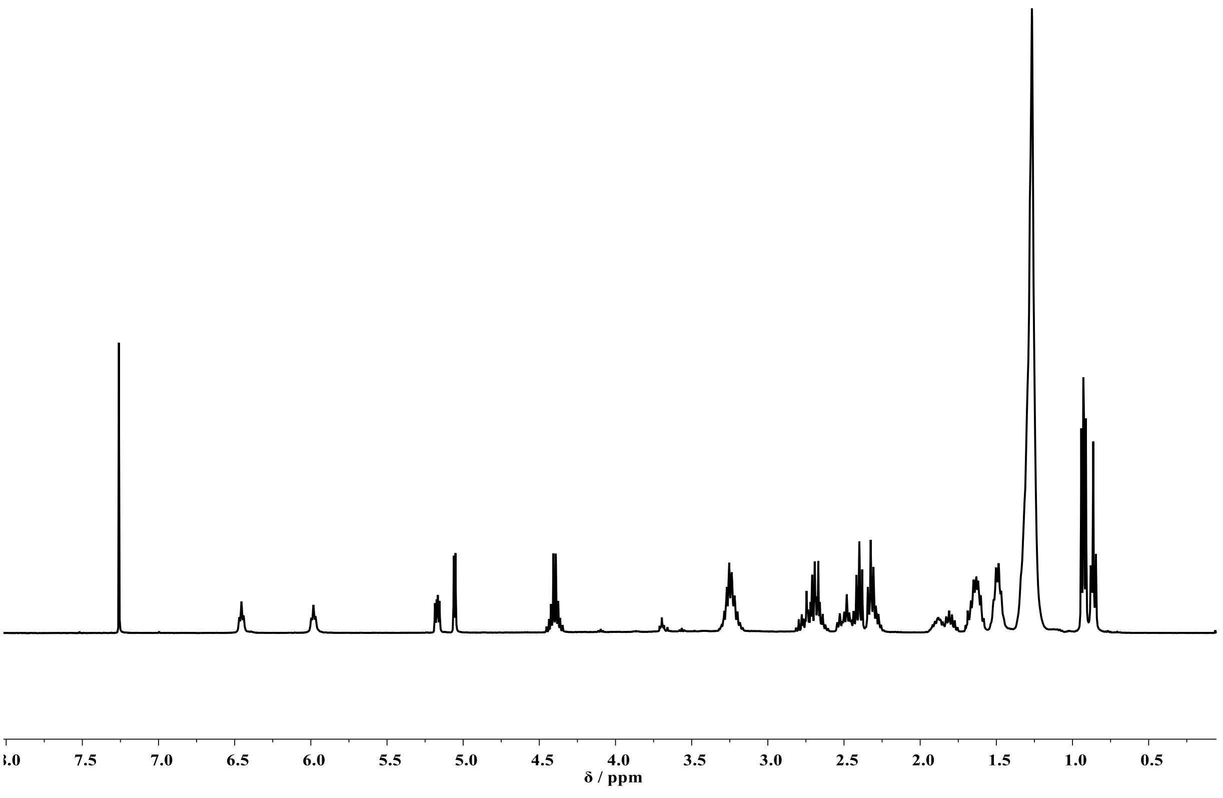

Supplementary Figure 92: ^{1}H-NMR of compound 66 measured in $CDCl_3$.

Passerini reaction

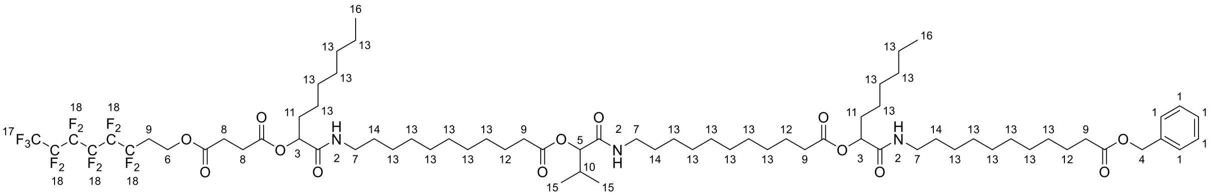

67

In a 50 mL round bottom flask, 274 mg of **66** (232 µmol, 1.00 eq.) was dissolved in 3.00 mL DCM and 41.9 µL heptanal **14b** (39.7 mg, 348 µmol, 1.50 eq.) and 105 mg of monomer **M1** (348 µmol, 1.50 eq.) were added. The mixture was stirred at room temperature for 3 days. Subsequently, the solvent was removed under reduced pressure. The crude product was purified by column chromatography (cyclohexane / ethyl acetate 4:1 →2:1) to afford product **67** as a pale highly viscous oil in a yield of 90.1% (314 mg, 209 µmol).

R_f = 0.13 in cyclohexane / ethyl acetate (2:1).

IR (ATR): ν / cm^{-1} = 3291.1 (vw), 2923.5 (s), 2852.7 (m), 1736.5 (vs), 1655.0 (vs), 1540.3 (m), 1465.1 (w), 1365.4 (w), 1235.0 (vs), 1144.9 (vs), 1005.5 (w), 842.2 (vw), 697.7 (m), 652.8 (w).

^{1}H NMR (400 MHz, CDCl$_3$): δ / ppm = 7.44 – 7.29 (m, 5 H, CH_{Ar}^{1}), 6.39 (t, J = 5.8 Hz, 1 H, NH^{2}), 6.02 (t, J = 5.9 Hz, 1 H, NH^{2}), 5.96 (t, J = 5.9 Hz, 1H, NH^{2}), 5.22 – 5.15 (m, 2 H, CH^{3}), 5.10 (s, 2 H, CH_2^{4}), 5.05 (d, J = 4.4 Hz, 1 H, CH^{5}), 4.49 – 4.34 (m, 2 H, CH_2^{6}), 3.32 – 3.14 (m, 6 H, CH_2^{7}), 2.83 – 2.58 (m, 4 H, CH_2^{8}), 2.55 – 2.23 (m, 9 H, CH^{9}, CH_2^{10}), 1.96 – 1.75 (m, 4 H, CH_2^{11}), 1.75 – 1.56 (m, 8 H, CH_2^{12}, CH_2^{13}), 1.56 – 1.39 (m, 6 H, CH_2^{14}), 1.37 – 1.16 (m, 52 H, CH_2^{13}), 0.96 – 0.90 (m, 6 H, CH_3^{15}), 0.89 – 0.83 (m, 6 H, CH_2^{16}).

^{13}C NMR (101 MHz, CDCl$_3$): δ / ppm = 173.82, 172.69, 172.66, 172.60, 171.25, 169.99, 169.74, 169.42, 136.26, 128.67, 128.29, 78.04, 74.74, 74.06, 66.20, 56.94, 39.44, 39.32, 39.28, 34.45, 34.41, 32.04, 31.93, 31.86, 31.75, 30.64, 30.57, 29.72, 29.68, 29.60,29.58, 29.53, 29.49, 29.35, 29.32, 29.26, 29.23, 29.22, 29.13, 29.04, 26.96, 25.13, 25.09, 25.07, 25.02, 24.83, 22.73, 22.66, 18.89, 17.06, 14.17.

^{19}F NMR (376 MHz, CDCl$_3$): δ / ppm = -85.09 (t, J = 9.9 Hz, 3 F, CF_3^{17}), -117.75 – -118.21 (m, 2 F, CF_2^{18}), -125.91 – -126.45 (m, 2 F, CF_2^{18}), -126.96 – -127.40 (m, 2 F, CF_2^{18}), -127.70 – -128.26 (m, 2 F, CF_2^{18}), -130.02 – -130.86 (m, 2 F, CF_2^{18}). Total integral of CF_2 region normalized with respect to the CF_3^{17} group = 10.

ESI-MS [*m/z*]: [M + Na]$^+$ calculated for $^{12}C_{74}{}^{1}H_{116}{}^{16}O_{13}{}^{14}N_{3}{}^{19}F_{13}$, 1524.8193; found, 1524.8176, Δ = 1.7 mmu.

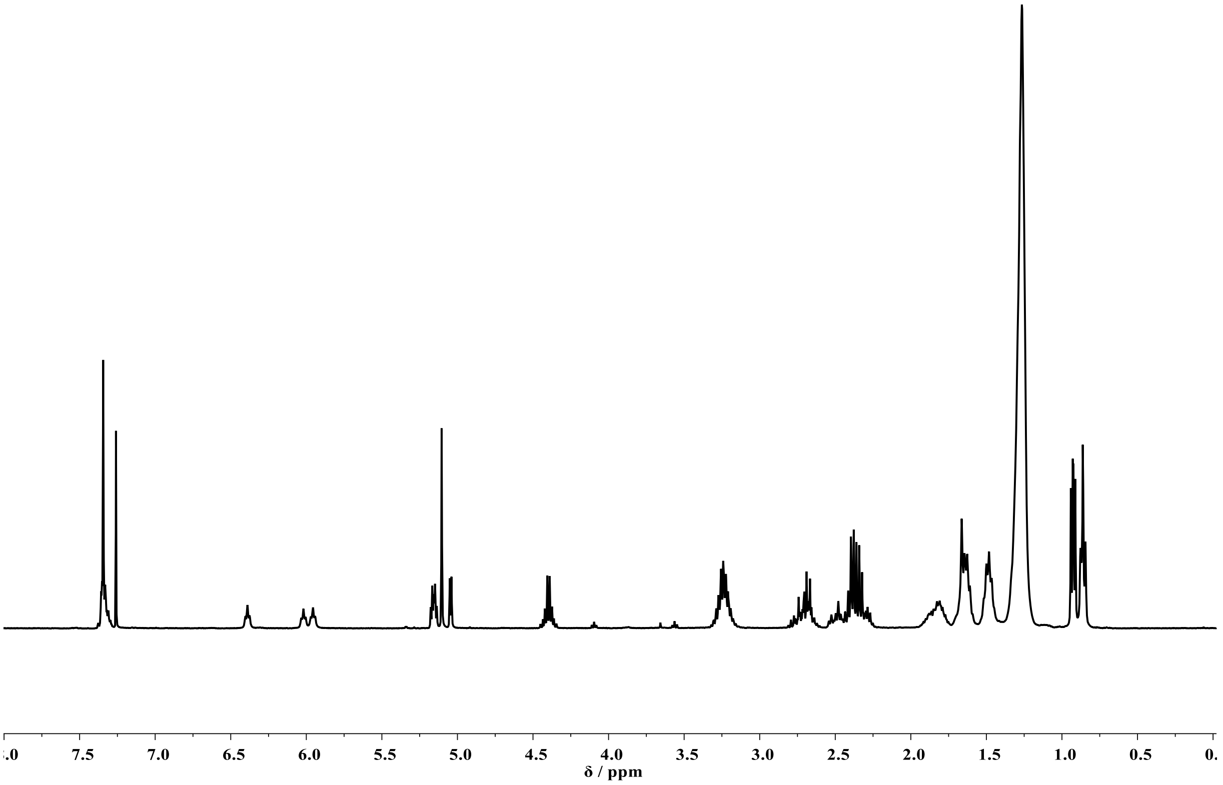

Supplementary Figure 93: ^{1}H-NMR of compound 67 measured in $CDCl_3$.

Deprotection

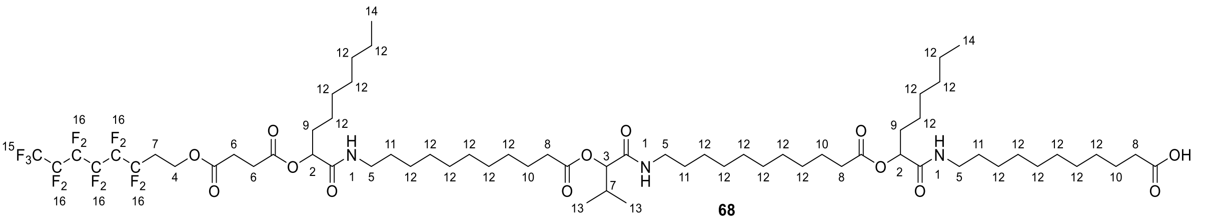

In a 25 mL round bottom flask, 225 mg of **67** (150 µmol, 1.00 eq.) was dissolved in 3.00 mL ethyl acetate and 3.00 mL THF. Subsequently, 91.4 mg (20 wt%) palladium on activated charcoal **16** was added to the solution. The resulting mixture was purged with hydrogen gas and stirred for 1 day at room temperature under hydrogen atmosphere (3 balloons). The heterogeneous catalyst was filtered off and the solvent was evaporated under reduced pressure. The product **68** was obtained as a pale highly viscous oil in a yield of 95.3%. (201 mg, 143 µmol).

IR (ATR): ν / cm^{-1} = 3290.0 (vw), 2922.6 (m), 2852.3 (m), 2335.9 (vw), 1737.7 (s), 1655.3 (s), 1554.2 (w), 1466.3 (w), 1366.0 (w), 1235.6 (s), 1144.9 (vs), 1008.2 (w), 842.6 (vw), 809.8 (vw), 698.5 (w), 652.2 (w), 567.4 (vw).

^{1}H NMR (400 MHz, $CDCl_3$): δ / ppm = 6.43 (t, J = 5.8 Hz, 1 H, NH[1]), 6.13 – 5.93 (m, 2 H, NH[1]), 5.21– 5.12 (m, 2 H, CH_2[2]), 5.04 (d, J = 4.5 Hz, 1 H, CH[3]), 4.48 – 4.32 (m, 2 H, CH_2[4]), 3.35 – 3.15 (m, 6 H, CH_2[5]), 2.84 – 2.59 (m, 4 H, CH_2[6]), 2.56 – 2.22 (m, 9 H, CH[7], CH_2[8]), 1.98 – 1.70 (m, 4 H, CH_2[9]), 1.70 – 1.56 (m, 6 H, CH_2[10]), 1.54 – 1.41 (m, 6 H, CH_2[11]), 1.40 – 1.16 (m, 54 H, CH_2[12]), 0.95 – 0.90 (m, 6 H, CH_3[13]), 0.89 – 0.83 (m, 6 H, CH_3[14]).

^{13}C NMR (101 MHz, $CDCl_3$): δ / ppm = 177.26, 172.80, 172.68, 172.64, 171.27, 170.10, 169.88, 169.61, 78.07, 74.73, 74.09, 56.96, 39.48, 39.35, 39.30, 34.47, 34.41, 34.07, 32.01, 31.92, 31.87, 31.75, 30.62, 29.69, 29.59, 29.55, 29.51, 29.43, 29.37, 29.34, 29.32, 29.25, 29.22, 29.14, 29.10, 29.04, 26.97, 26.96, 26.88, 25.12, 25.01, 24.94, 24.85, 22.73, 22.66, 18.88, 17.08, 14.17.

^{19}F NMR (376 MHz, $CDCl_3$): δ / ppm = -85.09 (t, J = 9.8 Hz, 3 F, CF_3[15]), -117.60 – -118.73 (m, 2 F, CF_2[16]), -125.73 – -126.37 (m, 2 F, CF_2[16]), -126.86 – -127.47 (m, 2 F, CF_2[16]), -127.73 – -128.10 (m, 2 F, CF_2[16]), -130.03 – -130.85 (m, 2 F, CF_2[16]). Total integral of CF_2 region normalized with respect to the CF_3[15] group = 10.

ESI-MS [*m/z*]: $[M + Na]^+$ calculated for $^{12}C_{67}{}^{1}H_{110}{}^{16}O_{13}{}^{14}N_3{}^{19}F_{13}{}^{23}Na$, 1434.7723; found, 1434.7705, Δ = 1.8 mmu.

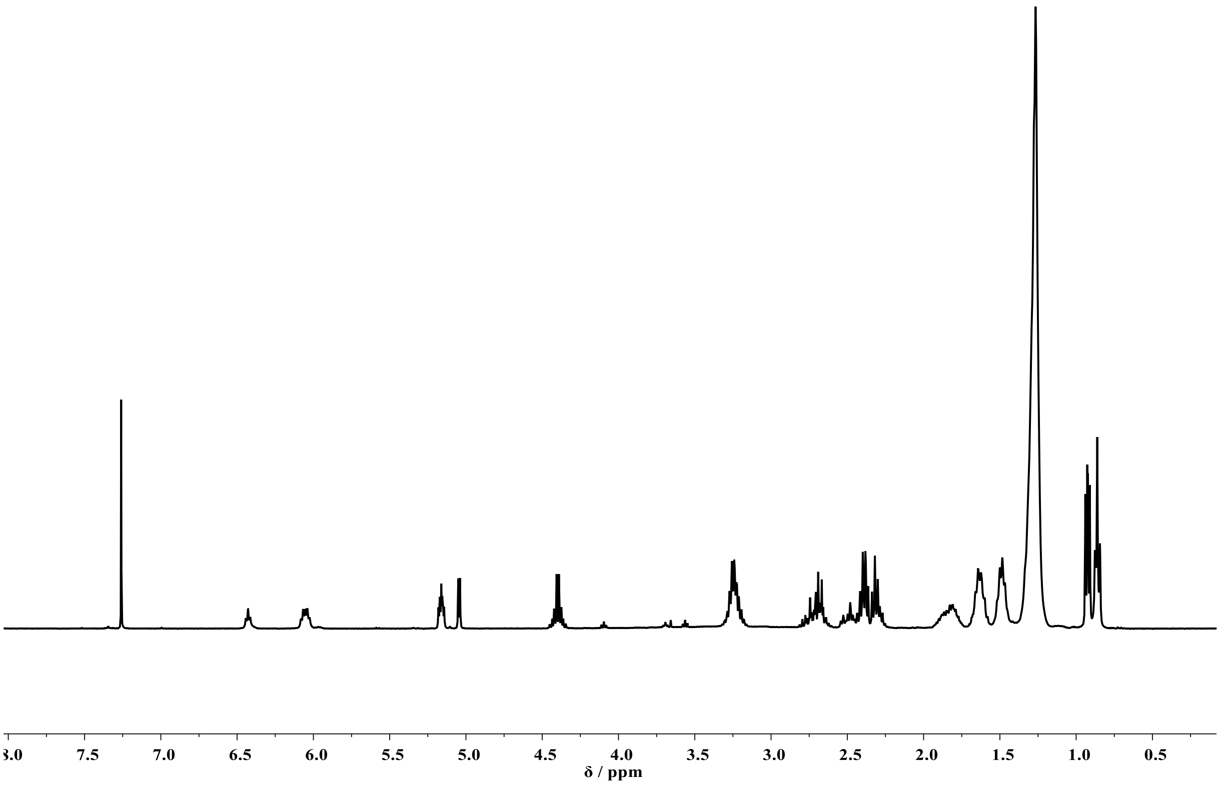

Supplementary Figure 94: ^{1}H-NMR of compound 68 measured in $CDCl_3$.

Passerini reaction

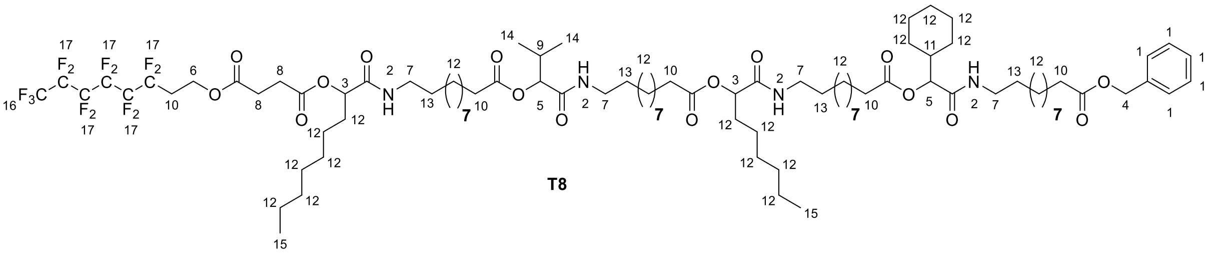

In a 50 mL round bottom flask, 164 mg **68** (116 µmol, 1.00 eq.) was dissolved in 2.00 mL DCM and 21.1 µL cyclohexancarboxaldehyde **14j** (19.5 mg, 174 µmol, 1.50 eq.) and 52.5 mg of monomer **M1** (174 µmol, 1.50 eq.) were added. The mixture was stirred at room temperature for 3 days. Subsequently, the solvent was removed under reduced pressure. The crude product was purified by column chromatography (cyclohexane / ethyl acetate 4:1 →1:1) to afford product **T8** as a pale highly viscous oil in a yield of 75.5% (160 mg, 87.6 µmol).

R_f = 0.53 in cyclohexane / ethyl acetate (3:2).

IR (ATR): ν / cm^{-1} = 3293.4 8 (w), 2922.3 (s), 2851.6 (m), 1735.9 (vs), 1654.5 8 (vs), 1537.5 (m), 1466.3 (w), 1362.4 (w), 1236.3 (vs), 1145.2 (vs), 1009.2 (w), 809.0 (vw), 697.2 (m), 652.2 (w), 566.9 (vw), 451.0 (vw).

^{1}H NMR (400 MHz, $CDCl_3$): δ / ppm = 7.41 – 7.30 (m, 5 H, $CH_{Ar}{}^{1}$), 6.39 (t, J = 5.8 Hz, 1 H, NH^{2}), 6.09 – 5.91 (m, 3 H, NH^{2}), 5.19 – 5.13 (m, 2 H, $CH_2{}^{3}$), 5.11 (s, 2 H, $CH_2{}^{4}$), 5.07 – 4.98 (m, 2 H, $CH_2{}^{5}$), 4.47 – 4.34 (m, 2 H, $CH_2{}^{6}$), 3.33 – 3.15 (m, 8 H, $CH_2{}^{7}$), 2.83 – 2.62 (m, 4 H, $CH_2{}^{8}$), 2.56 – 2.24 (m, 11 H, CH^{9}, $CH_2{}^{10}$), 2.00 – 1.59 (m, 21 H, CH^{11}, $CH_2{}^{12}$), 1.54 – 1.44 (m, 8 H, $CH_2{}^{13}$), 1.38 – 1.06 (m, 68 H, $CH_2{}^{12}$), 0.97 – 0.90 (m, 6 H, $CH_2{}^{14}$), 0.90 – 0.82 (m, 6 H, $CH_2{}^{15}$).

^{13}C NMR (101 MHz, $CDCl_3$): δ / ppm = 173.84, 172.68, 172.62, 171.54, 171.53, 171.26, 170.00, 169.74, 169.42, 169.34, 136.26, 128.68, 128.30, 78.04, 77.75, 74.74, 74.06, 66.21, 40.11, 39.44, 39.32, 39.28, 34.46, 34.43, 32.05, 31.94, 31.87, 31.76, 30.65, 30.45, 29.85, 29.74, 29.71, 29.59, 29.54, 29.51, 29.48, 29.35, 29.33, 29.27, 29.24, 29.23, 29.14, 29.05, 27.41, 26.97, 26.21, 26.13, 26.01, 25.13, 25.10, 25.08, 25.03, 24.85, 22.74, 22.67, 18.91, 17.06, 14.19.

^{19}F NMR (376 MHz, $CDCl_3$): δ / ppm = -84.73 – -85.47 (m, 3 F, $CF_3{}^{16}$), -117.67 – -118.35 (m, 2 F, $CF_2{}^{17}$), -125.60 – -126.44 (m, 2 F, $CF_2{}^{17}$), -126.83 – -127.22 (m, 2 F, $CF_2{}^{17}$), -127.54 – -128.48 (m, 2 F, $CF_2{}^{17}$), -130.28 – -131.34 (m, 2 F, $CF_2{}^{17}$).

Total integral of CF_2 region normalized with respect to the $CF_3{}^{16}$ group = 10.

ESI-MS [*m/z*]: $[M + H]^+$ calculated for $^{12}C_{93}{}^{1}H_{149}{}^{16}O_{16}{}^{14}N_4{}^{19}F_{13}$, 1826.0834; found, 1826.0885 Δ = 5.1 mmu.

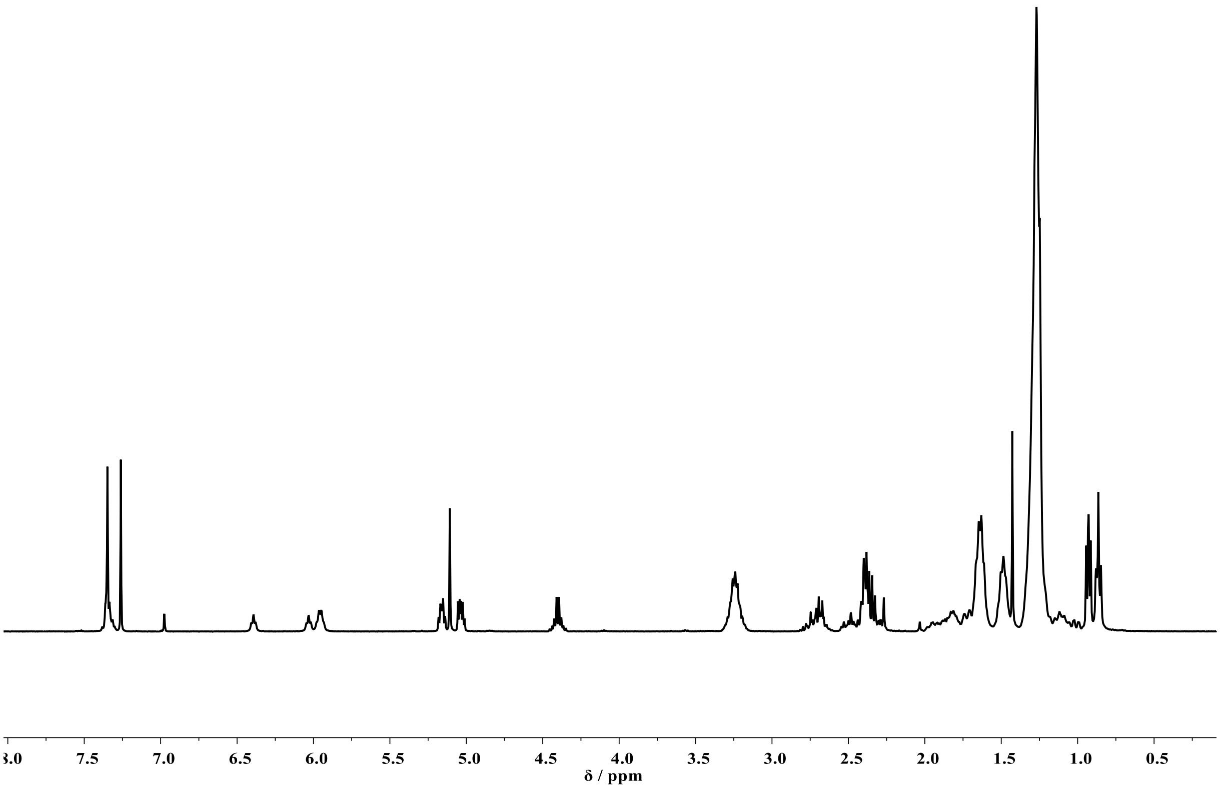

Supplementary Figure 95: ^{1}H-NMR of compound T8 measured in $CDCl_3$.

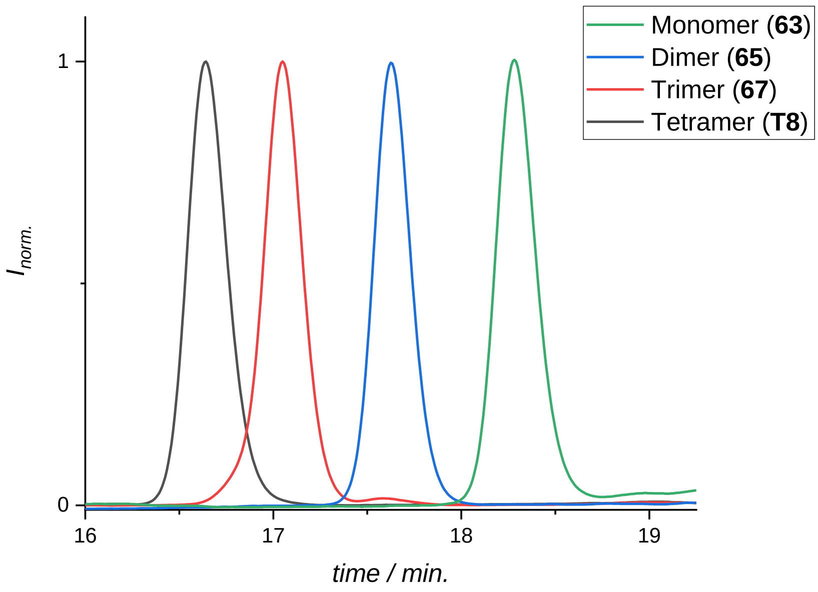

Supplementary Figure 96: SEC traces of the intermediates after each P3CR in the synthesis of product T8.

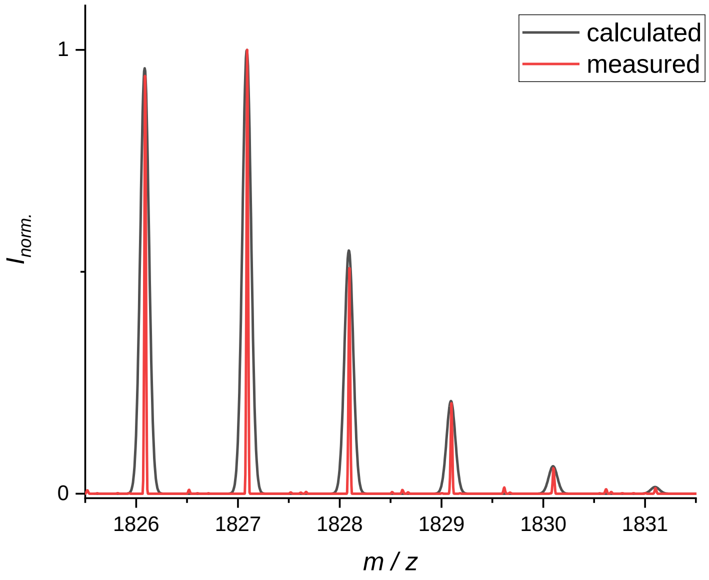

Supplementary Figure 97: High resolution ESI-MS measurement of T8. The observed isotopic pattern is compared with the calculated isotopic pattern obtained from mMass (black).

```
imum is 1.000000 found for mass 1827.090720
matching mass 1827.09072
cutoff 0.50000: 0 solutions (9 peaks)
cutoff 0.25000: 0 solutions (20 peaks)
cutoff 0.12500: 0 solutions (54 peaks)
cutoff 0.06250: 0 solutions (103 peaks)
cutoff 0.03125: 0 solutions (155 peaks)
cutoff 0.01562: 0 solutions (287 peaks)
cutoff 0.00781: 0 solutions (423 peaks)
cutoff 0.00391: 0 solutions (568 peaks)
cutoff 0.00195: 1 solutions (1147 peaks)
1827.09072 ≈ 447.026590 + 339.277350 + 283.214750 + 325.261700 + 323.246050 + 107.049690 (sides Octanal,
 Isobutyraldehyde, Heptanal, Cyclohexancarboxaldehyde; error -2.01459)
Press ENTER to quit ...
```

Supplementary Figure 98: Screenshot of the automated read-out of T8.

1.3.5 Oligomer synthesis with TAG2

1.3.5.1 Synthesis of tetramer T9

Passerini reaction

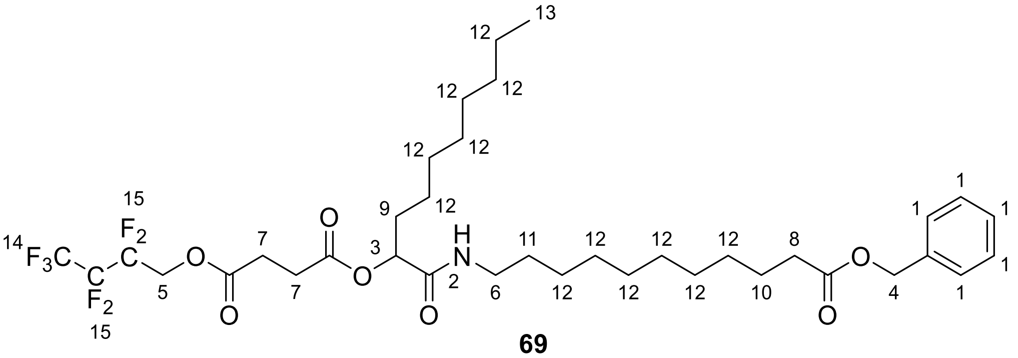

69

In a 50 mL round bottom flask, 1.10 g of **TAG2** (3.68 mmol, 1.00 eq.) was stirred in 4.00 mL dichloromethane. Subsequently, 2.01 mL nonanal **14k** (758 mg, 5.52 mmol, 1.50 eq.) and 1.66 g of the monomer **M1** (5.52 mmol, 1.50 eq.) were added. The resulting reaction mixture was stirred at room temperature for 2 days. Afterwards, the crude mixture was dried under reduced pressure. The residue was adsorbed onto celite® and purified *via* column chromatography on silica gel eluting with a gradual solvent mixture of ethyl acetate and cyclohexane (9:1 → 4:1) to yield the passerini product **69** as a pale highly viscous oil (2.65 g, 3.56 mmol, 96.7%).

R_f = 0.32 in cyclohexane / ethyl acetate (3:1).

IR (ATR): ν / cm^{-1} = 3303.5 (vw), 2925.5 (m), 2854.8 (w), 1739.1 (s), 1657.5 (m), 1536.4 (w), 1456.0 (w), 1352.3 (w), 1226.4 (vs), 1144.5 (vs), 1020.1 (m),), 909.6 (w), 736.0 (m), 697.2 (m).

^{1}H NMR (500 MHz, $CDCl_3$): δ / ppm = 7.42 – 7.32 (m, 5 H, CH_{Ar}^{1}), 6.24 (t, J = 5.8 Hz, 1 H, NH^{2}), 5.23 – 5.16 (m, 1 H, CH^{3}), 5.13 (s, 2 H, CH_2^{4}), 4.74 – 4.52 (m, 2 H, CH_2^{5}), 3.36 – 3.17 (m, 2 H, CH_2^{6}), 2.92 – 2.66 (m, 4 H, CH_2^{7}), 2.37 (t, J = 7.6 Hz, 2 H, CH_2^{8}), 1.96 – 1.78 (m, 2 H, CH_2^{9}), 1.70 – 1.61 (m, 2 H, CH_2^{10}), 1.57 – 1.47 (m, 2 H, CH_2^{11}), 1.40 – 1.21 (m, 24 H, CH_2^{12}), 0.89 (t, J = 6.9 Hz, 3 H, CH_3^{13}).

^{13}C NMR (126 MHz, $CDCl_3$): δ / ppm = 173.70, 171.37, 170.77, 169.48, 136.14, 128.55, 128.17, 74.76, 66.07, 59.56 (t, J = 26.9 Hz), 39.32, 34.32, 31.82, 29.46, 29.42, 29.38, 29.37, 29.24, 29.21, 29.20, 29.11, 28.92, 28.66, 26.83, 24.94, 24.85, 22.64, 14.09.

^{19}F NMR (376 MHz, $CDCl_3$): δ / ppm =-80.89 (t, J = 9.3 Hz, 3F, CF_3^{14}), -119.83 – -121.39 (m, 2 F, CF_2^{15}), -127.31 – -128.95 (m, 2 F, CF_2^{15}). Total integral of CF_2 region normalized with respect to the CF_3^{14} group = 4.

ESI-MS [*m/z*]: $[M + H]^+$ calculated for $^{12}C_{36}{}^{1}H_{52}{}^{16}O_7{}^{14}N^{19}F_7$, 744.3705; found, 744.3693, Δ = 1.2 mmu.

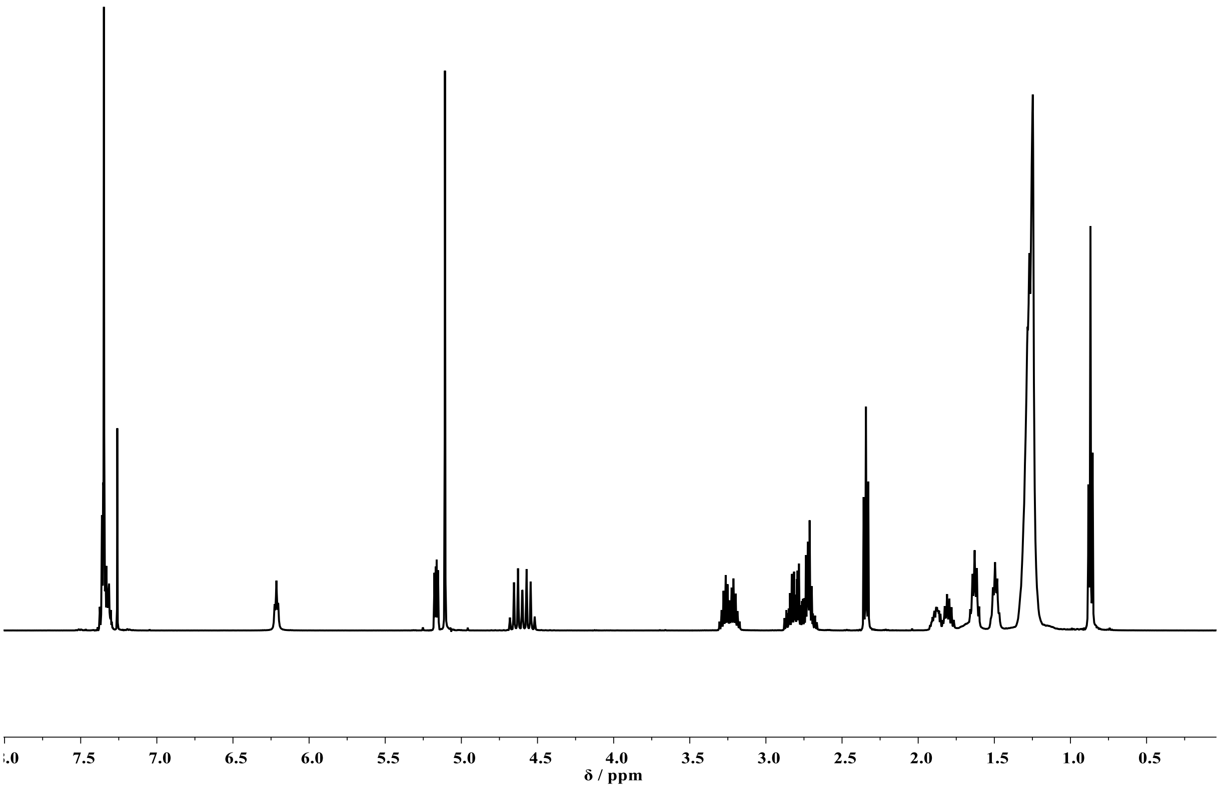

Supplementary Figure 99: ^{1}H-NMR of compound 69 measured in $CDCl_3$.

Deprotection

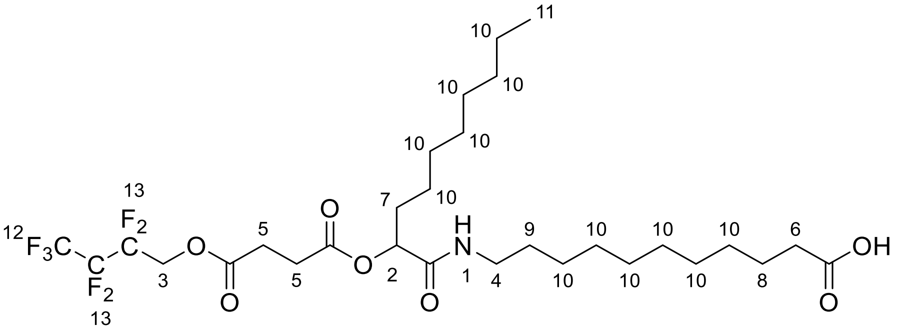

70

In a 25 mL round bottom flask, 2.08 g of **69** (3.29 mmol, 1.00 eq.) was dissolved in 4.00 mL ethyl acetate and 4.00 mL THF. Subsequently, 488 mg (20 wt.-%) palladium on activated charcoal **16** were added to the solution. The resulting mixture was purged with hydrogen gas and stirred for one day at room temperature under hydrogen atmosphere (balloon). The crude reaction mixture was filtered over celite® and flushed with 50 mL dichloromethane. After evaporation of the solvents and drying under reduced pressure the corresponding acid **70** was obtained as a colorless solid (2.11 g, 3.22 mol, 98.0%).

IR (ATR): ν / cm^{-1} = 3298.2 (w), 2920.6 (s), 2850.8 (s), 1744.3 (vs), 1692.7 (vs), 1651.4 (vs), 1557.3 (m), 1468.7 (w), 1412.0 (m), 1218.3 (vs), 1159.3 (vs), 1022.3 (s), 911.8 (m), 723.0 (w), 671.8 (w), 535.7 (w).

^{1}H NMR (400 MHz, $CDCl_3$): δ / ppm = 6.26 (t, J = 5.8 Hz, 1H, NH^{1}), 5.22 – 5.09 (m, 1 H, CH^{2}), 4.70 – 4.49 (m, 2 H, CH_2^{3}), 3.34 – 3.15 (m, 2 H, CH_2^{4}), 2.92 – 2.64 (m, 4 H, CH_2^{5}), 2.33 (t, J = 7.5 Hz, 2 H, CH_2^{6}), 1.95 – 1.74 (m, 2 H, CH_2^{7}), 1.68 – 1.57 (m, 2 H, CH_2^{8}), 1.56 – 1.42 (m, 2 H, CH_2^{9}), 1.41 – 1.17 (m, 24 H, CH_2^{10}), 0.91 – 0.79 (m, 3 H, CH_3^{11}).

^{13}C NMR (101 MHz, $CDCl_3$): δ / ppm = 179.20, 171.52, 170.93, 169.77, 74.87, 59.69 (t, J = 26.8 Hz), 39.47, 34.08, 31.94, 31.92, 29.49, 29.47, 29.38, 29.35, 29.32, 29.27, 29.24, 29.10, 29.04, 28.79, 26.90, 24.97, 24.79, 22.76, 14.20.

^{19}F NMR (376 MHz, $CDCl_3$): δ / ppm = -80.84 (t, J = 9.3 Hz, 3 F, CF_3^{12}), -120.14 – -121.45 (m, 2 F, CF_2^{13}), -127.34 – -128.12 (m, 2 F, CF_2^{13}). Total integral of CF_2 region normalized with respect to the CF_3^{12} group = 4.

ESI-MS [*m/z*]: $[M + H]^+$ calculated for $^{12}C_{29}{}^{1}H_{46}{}^{16}O_7{}^{14}N^{19}F_7$, 654.3235; found, 654.3219, Δ = 1.4 mmu.

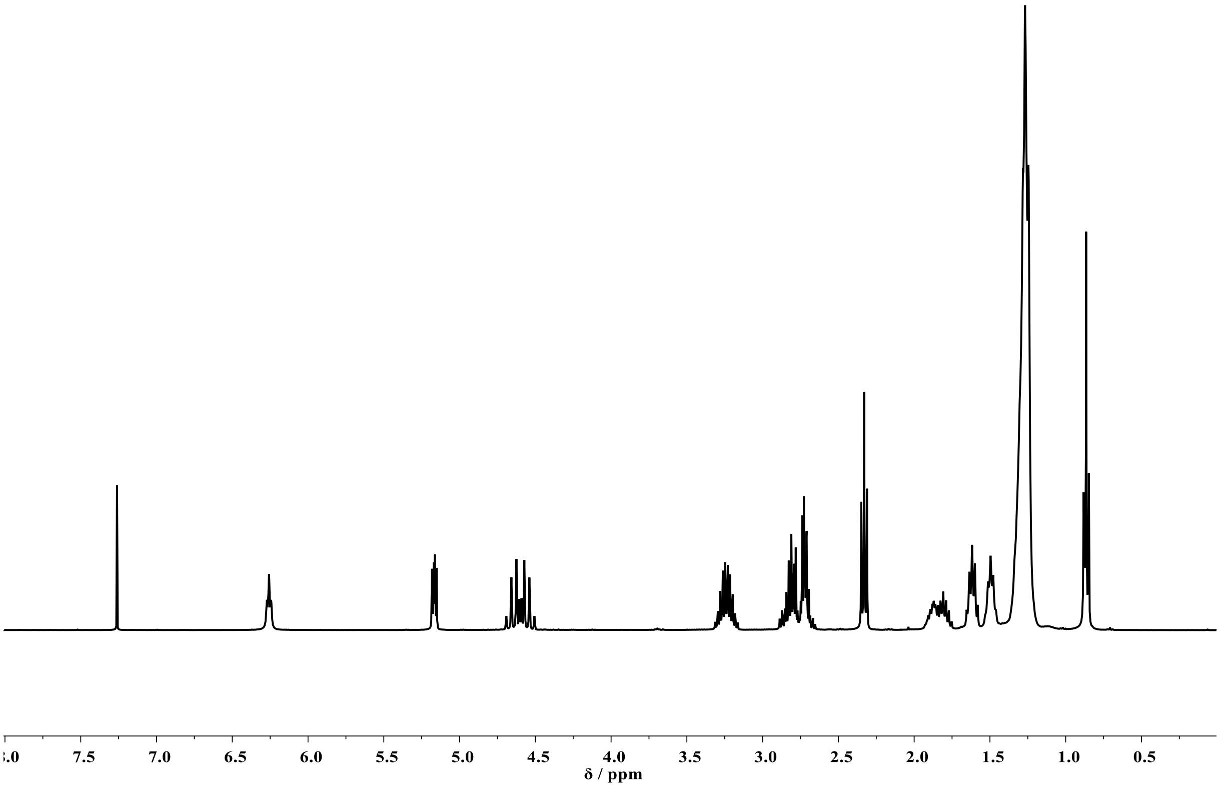

Supplementary Figure 100: ^{1}H-NMR of compound 70 measured in $CDCl_3$.

Passerini reaction

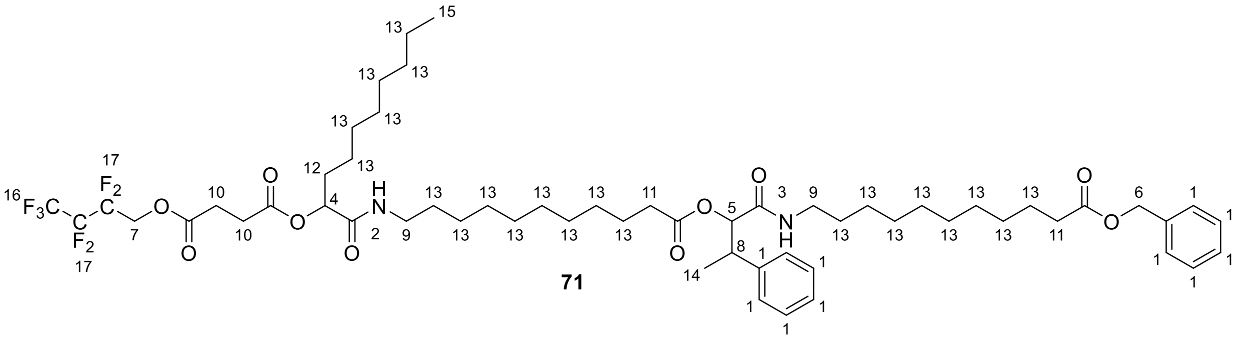

In a 50 mL round bottom flask, 2.05 g of **71** (3.13 mmol, 1.00 eq.) was stirred in 3.00 mL dichloromethane. Subsequently, 629 µL 2-phenylpropionaldehyde **14e** (630 mg, 4.70 mmol, 1.50 eq.) and 1.42 g of the monomer **M1** (4.70 mmol, 1.50 eq.) were added. The resulting reaction mixture was stirred at room temperature for 2 days. Afterwards, the crude mixture was dried under reduced pressure. The residue was adsorbed onto celite® and purified *via* column chromatography on silica gel eluting with a gradual solvent mixture of ethyl acetate and cyclohexane (7:1 → 1:1) to yield the passerini product **71** as a pale highly viscous oil. (3.41 g, 3.13 mmol, 99.9%).

R_f = 0.30 in cyclohexane / ethyl acetate (1:1).

IR (ATR): ν / cm^{-1} = 3307.3 (vw), 2925.3 (s), 2854.5 (m), 1738.9 (s), 1655.6 (s), 1535.1 (w), 1496.9 (vw), 1454.8 (w), 1352.6 (w), 1226.4 (vw), 1144.0 (vs), 1020.0 (m), 909.5 (w), 735.3 (w), 698.2 (s), 534.5 (w).

^{1}H NMR (400 MHz, $CDCl_3$): δ / ppm = 7.43 – 7.15 (m, 10 H, CH_{Ar}^{1}), 6.23 (t, J = 5.8 Hz, 1 H, NH^{2}), 5.64 (t, J = 5.8 Hz, 0.5 H, NH^{3a}), 5.58 (t, J = 5.9 Hz, 0.5 H, NH^{3b}), 5.32 (d, J = 5.4 Hz, 0.5 H, CH^{4a}), 5.21 (d, J = 5.4 Hz, 0.5 H, CH^{4b}), 5.19 – 5.14 (m, 1 H, CH^{5}), 5.11 (s, 2 H, CH_2^{6}), 4.73 – 4.46 (m, 2 H, CH_2^{7}), 3.52 – 3.39 (m, 1 H, CH^{8}), 3.35 – 2.96 (m, 4 H, CH_2^{9}), 2.90 – 2.64 (m, 4 H, CH_2^{10}), 2.41 – 2.28 (m, 4 H, CH_2^{11}), 1.96 – 1.74 (m, 2 H, CH_2^{12}), 1.72 – 1.00 (m, 47 H, CH_2^{13}, CH_3^{14}), 0.91 – 0.83 (m, 3 H, CH_3^{15}).

^{13}C NMR (101 MHz, $CDCl_3$): δ / ppm = 173.80, 172.57, 172.42, 171.47, 170.88, 169.61, 168.82, 168.61, 141.73, 141.18, 136.26, 128.66, 128.52, 128.32, 128.28, 127.97, 127.13, 127.05, 77.85, 74.88, 66.19, 59.67 (t, J = 27.0 Hz), 41.58, 41.29, 39.43, 39.29, 39.20, 34.44, 34.35, 34.30, 31.94, 29.59, 29.55, 29.51, 29.52, 29.48, 29.47, 29.39, 29.35, 29.30, 29.23, 29.18, 29.16, 29.04, 28.78, 26.96, 26.86, 26.80, 25.06, 24.98, 24.91, 22.75, 17.62, 15.30, 14.20.

^{19}F NMR (376 MHz, $CDCl_3$): δ / ppm = -80.81 (t, J = 9.1 Hz, 3 F, CF_3), -120.24 – -120.74 (m, 2 F, CF_2), -127.46 – -127.96 (m, 2 F, CF_2). Total integral of CF_2 region normalized with respect to the CF_3^{14} group = 4.

ESI-MS [*m/z*]: $[M + H]^+$ calculated for $^{12}C_{57}{}^{1}H_{83}{}^{16}O_{10}{}^{14}N_2{}^{19}F_7$, 1089.6009; found, 1089.5993, Δ = 1.6 mmu.

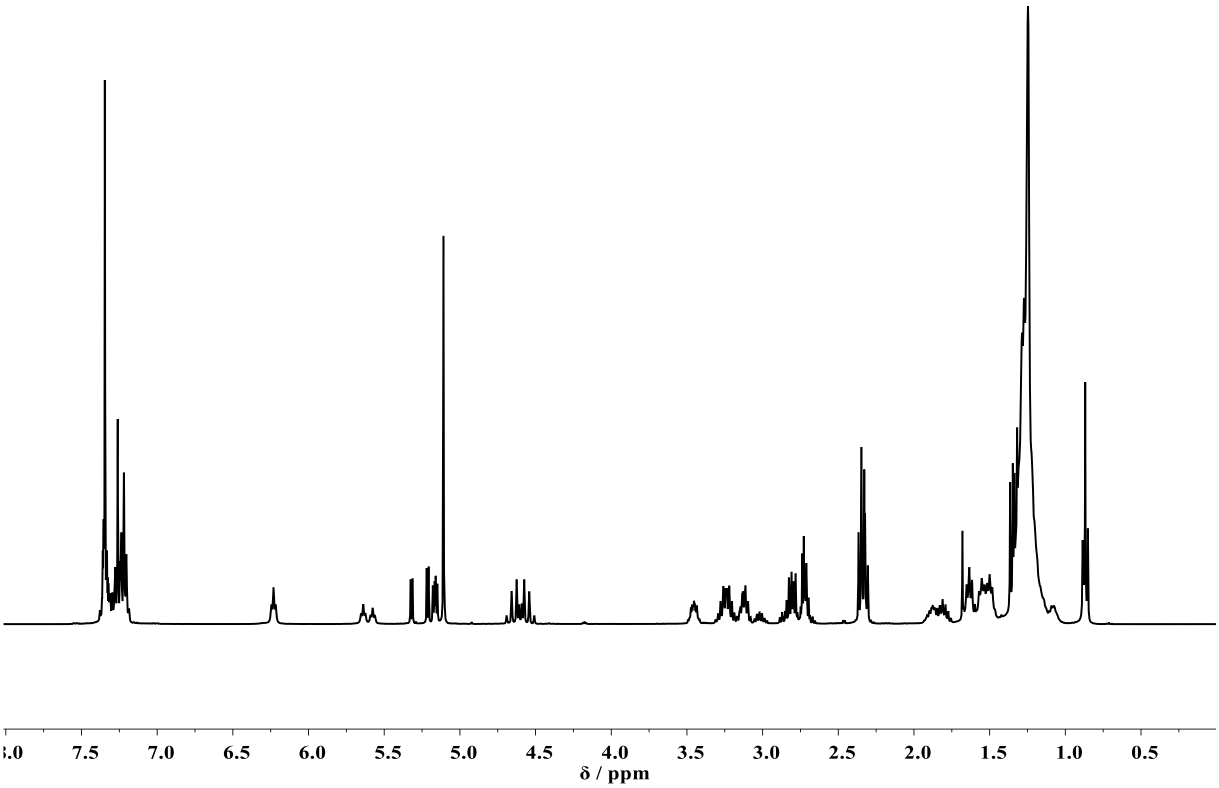

Supplementary Figure 101: ^{1}H-NMR of compound 71 measured in $CDCl_3$.

Deprotection

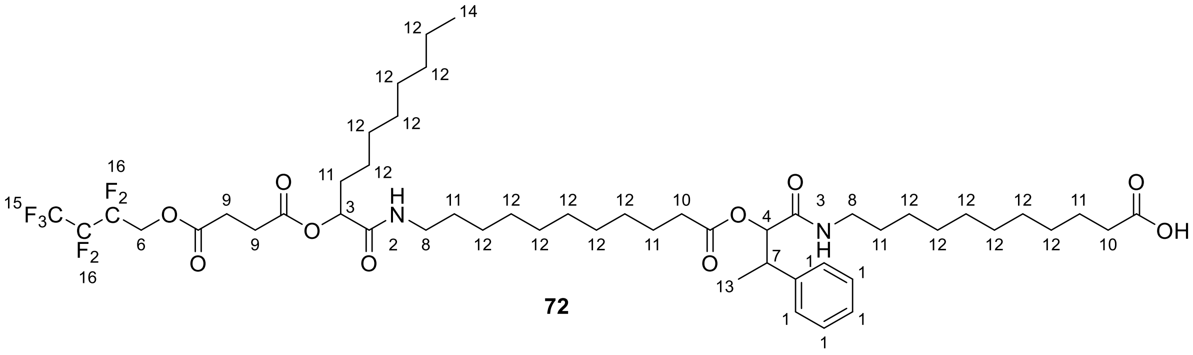

72

In a 25 mL round bottom flask, 3.26 g of **71** (2.99 mmol, 1.00 eq.) was dissolved in 5.00 mL ethyl acetate and 5.00 mL THF. Subsequently, 652 mg (20 wt.-%) palladium on activated charcoal **16** were added to the solution. The resulting mixture was purged with hydrogen gas and stirred for one day at room temperature under hydrogen atmosphere (balloon). The crude reaction mixture was filtered over celite® and flushed with 50 mL dichloromethane. After evaporation of the solvents and drying under reduced pressure the product **72** was obtained as a colorless solid (2.97 g, 2.97 mol, 99.3%).

IR (ATR): ν / cm^{-1} = 3306.6 (vw), 2924.9 (d), 2854.3 (m), 2164.0 (vw), 2111.2 (vw), 2016.9 (vw), 1741.2 (s), 1651.4 (s), 1540.4 (m), 1495.6 (vw), 1454.8 (w), 1410.5 (w),1352.8 (w), 1226.4 (vs), 1179.0

(vs), 1144.2 (vs), 1020.6 (m), 979.1 (w), 909.8 (w), 759.9 (w), 735.8 (w), 700.2 (m), 630.7 (w), 536.7 (w), 411.1 (vw).

^{1}H NMR (400 MHz, $CDCl_3$): δ / ppm = 7.40 – 7.20 (m, 5 H, CH_{Ar}^{1}), 6.35 (t, J = 5.8 Hz, 1 H, NH^{2}), 5.76 (t, J = 5.8 Hz, 0.5 H, NH^{3a}), 5.69 (t, J = 5.9 Hz, 0.5 H, NH^{3b}), 5.37 (d, J = 5.3 Hz, 0.5 H, CH^{4a}), 5.26 (d, J = 5.3 Hz, 0.5 H, CH^{4b}), 5.23 – 5.14 (m, 1 H, CH^{5}), 4.78 – 4.53 (m, 2 H, CH_2^{6}), 3.56 – 3.43 (m, 1 H, CH^{7}), 3.38 – 3.00 (m, 4 H, CH_2^{8}), 2.95 – 2.68 (m, 4 H, CH_2^{9}), 2.43 – 2.27 (m, 4 H, CH_2^{10}), 2.03 – 1.45 (m, 10 H, CH_2^{11}), 1.44 – 1.06 (m, 39 H, CH_2^{12}, CH_3^{13}), 0.91 (t, J = 6.7 Hz, 3 H, CH_3^{14}).

^{13}C NMR (101 MHz, $CDCl_3$): δ / ppm = 178.45, 172.60, 172.45, 171.51, 170.92, 169.81, 168.97, 168.74, 141.66, 141.12, 128.50, 128.29, 127.95, 127.11, 127.05, 77.82, 74.82, 59.65 (t, J = 26.8 Hz), 41.54, 41.23, 39.47, 39.28, 39.19, 34.33, 34.27, 34.08, 31.91, 31.89, 29.57, 29.49, 29.46, 29.43, 29.37, 29.32, 29.25, 29.19, 29.16, 29.14, 29.11, 29.01, 28.76, 26.93, 26.79, 26.72, 24.95, 24.89, 24.84, 22.73, 17.59, 15.26, 14.18.

^{19}F NMR (376 MHz, $CDCl_3$): δ / ppm = -85.17 (t, J = 9.2 Hz, 3 F, CF_3^{15}), -124.68 – -125.12 (m, 2 F, CF_2^{16}), -131.78 – -132.20 (m, 2 F, CF_2^{16}). Total integral of CF_2 region normalized with respect to the CF_3^{15} group = 4.

ESI-MS [*m/z*]: $[M + H]^+$ calculated for $^{12}C_{50}{}^{1}H_{77}{}^{16}O_{10}{}^{14}N_2{}^{19}F_7$, 999.5539; found, 999.5526, Δ = 1.3 mmu.

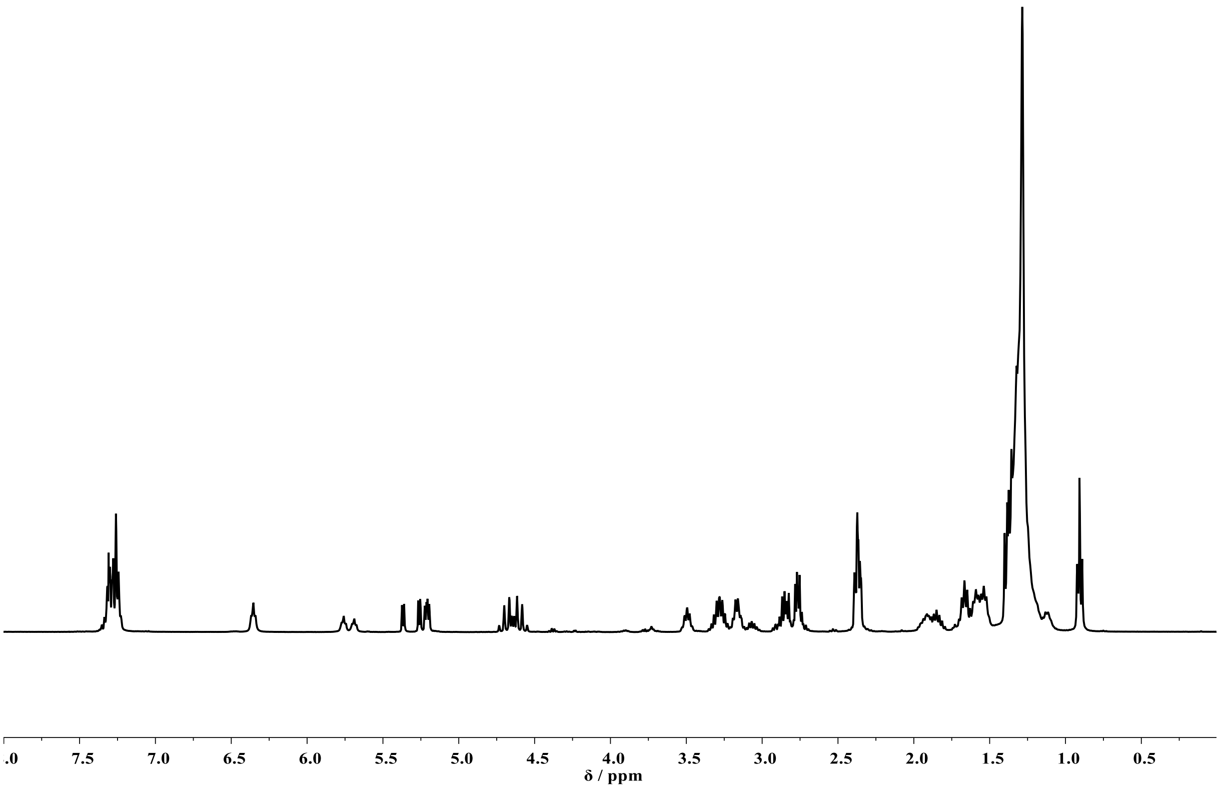

Supplementary Figure 102: ^{1}H-NMR of compound 72 measured in $CDCl_3$.

Passerini reaction

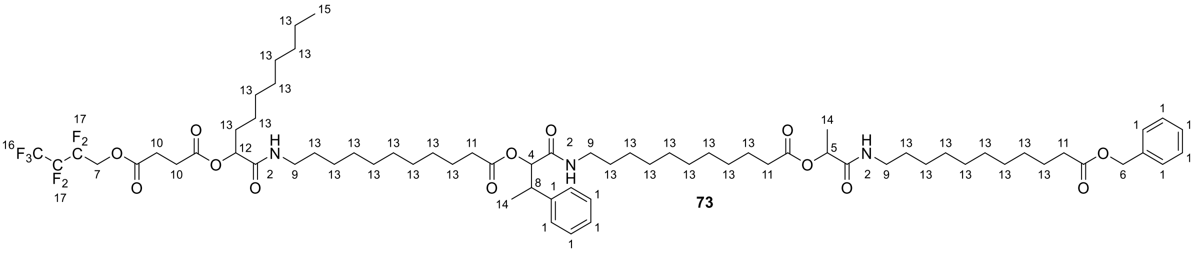

73

In a 50 mL round bottom flask, 2.84 g of **72** (2.84 mmol, 1.00 eq.) was stirred in 3.00 mL dichloromethane. Subsequently, 482 µL acetaldehyde **14f** (376 mg, 8.53 mmol, 3.00 eq.) and 1.29 g of the monomer **M1** (4.27 mmol, 1.50 eq.) were added. The resulting reaction mixture was stirred at room temperature for 1 day. Afterwards, the crude mixture was dried under reduced pressure. The residue was adsorbed onto celite® and purified *via* column chromatography on silica gel eluting with a gradual solvent mixture of ethyl acetate and cyclohexane (4:1 → 1:1) to yield the passerini product **73** as a pale highly viscous oil. (3.50 g, 2.60 mmol, 91.6%).

R_f = 0.60 in cyclohexane / ethyl acetate (1:1).

IR (ATR): ν / cm^{-1} = 3305.7 (vw), 3086.0 (vw), 2925.0 (s), 2853.9 (m), 2097.9 (vw), 1739.2 (s), 1655.6 (s), 1535.4 (m), 1497.2 (vw), 1454.8 (w), 1353.3 (w), 1226.9 (vs), 1145.6 (vs), 1020.8 (m), 909.8 (w), 736.0 (w), 699.1 (m), 538.7 (vw), 405.4 (vw).

^{1}H NMR (400 MHz, $CDCl_3$): δ / ppm = 7.44 – 7.15 (m, 10 H, CH_{Ar}^{1}), 6.24 (t, *J* = 5.8 Hz, 1 H, NH^{2}), 6.09 (t, *J* = 5.8 Hz, 1 H, NH^{2}), 5.65 (t, *J* = 5.9 Hz, 0.5 H, NH^{3a}), 5.59 (t, *J* = 5.9 Hz, 0.5 H, NH^{3b}), 5.31 (d, *J* = 5.4 Hz, 0.5 H, CH^{4a}), 5.25 – 5.14 (m, 0.5 H, 2 H, CH^{4b}, CH^{5}), 5.11 (s, 2 H, CH_2^{6}), 4.72 – 4.49 (m, 2 H, CH_2^{7}), 3.51 – 3.39 (m, 1 H, CH^{8}), 3.34 – 2.97 (m, 6 H, CH_2^{9}), 2.90 – 2.64 (m, 4 H, CH^{10}), 2.43 – 2.26 (m, 6 H, CH_2^{11}), 1.95 – 1.73 (m, 2 H, CH_2^{12}), 1.71 – 1.05 (m, 66 H, CH_2^{13}, CH_3^{14}), 0.90 – 0.82 (m, 3 H, CH_3^{15}).

^{13}C NMR (101 MHz, $CDCl_3$): δ / ppm = 173.80, 172.58, 172.44, 172.38, 171.48, 170.89, 170.42, 169.62, 168.85, 168.64, 141.74, 141.21, 136.27, 128.67, 128.53, 128.32, 128.29, 127.97, 127.13, 127.05, 77.86, 74.88, 70.59, 66.19, 59.68 (t, *J* = 26.9 Hz), 41.58, 41.29, 39.43, 39.36, 39.29, 39.20, 34.46, 34.45, 34.36, 34.30, 31.94, 29.67, 29.59, 29.56, 29.53, 29.49, 29.47, 29.41, 29.33, 29.31, 29.28, 29.23, 29.20, 29.19, 29.16, 29.04, 28.79, 26.95, 26.86, 26.80, 25.06, 25.01, 24.98, 24.92, 22.76, 18.09, 17.63, 15.31, 14.21.

^{19}F NMR (376 MHz, $CDCl_3$): δ / ppm = -80.81 (t, *J* = 9.3 Hz, 3 F, CF_3^{16}), -120.19 – -120.65 (m, 2 F, CF_2^{17}), -127.59 – -127.72 (m, 2 F, CF_2^{17}). Total integral of CF_2 region normalized with respect to the CF_3^{17} group = 4.

ESI-MS [*m/z*]: $[M + H]^+$ calculated for $^{12}C_{71}{}^{1}H_{108}{}^{16}O_{13}{}^{14}N_3{}^{19}F_7$, 1344.7843; found, 1344.7813, Δ = 3.0 mmu.

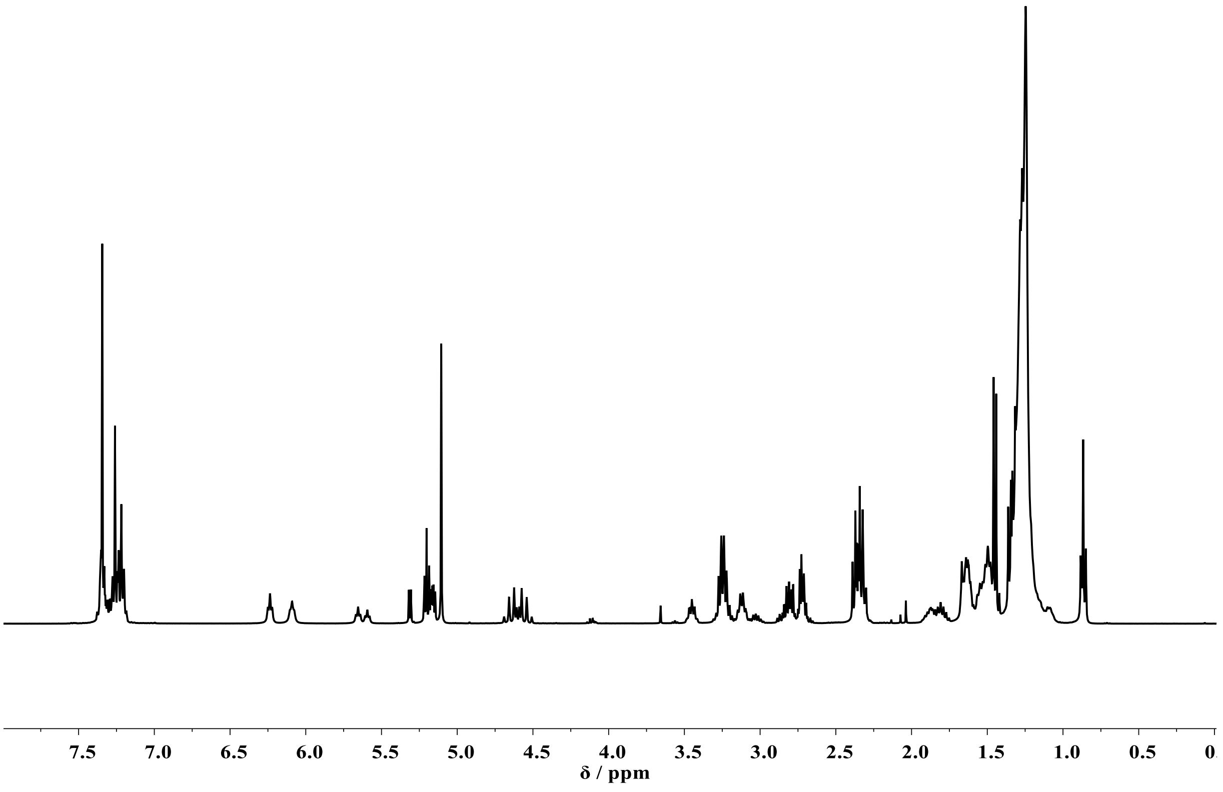

Supplementary Figure 103: ^{1}H-NMR of compound 73 measured in CDCl$_3$.

Deprotection

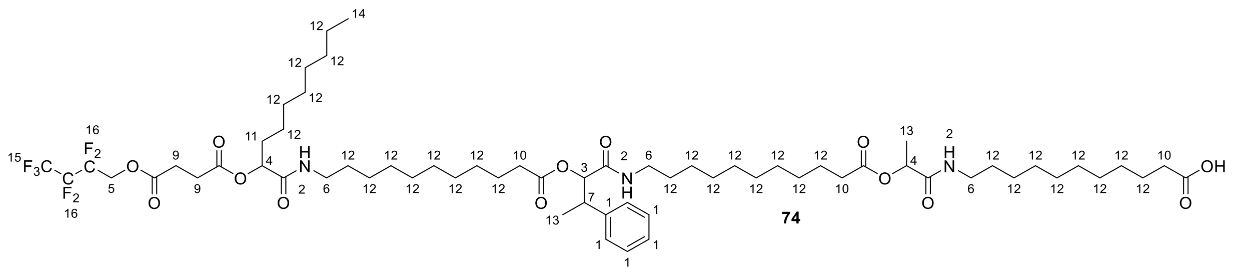

In a 25 mL round bottom flask, 3.32 g of **73** (2.47 mmol, 1.00 eq.) was dissolved in 6.00 mL ethyl acetate and 6.00 mL THF. Subsequently, 764 mg (20 wt.-%) palladium on activated charcoal **16** were added to the solution. The resulting mixture was purged with hydrogen gas and stirred for one day at room temperature under hydrogen atmosphere (3 balloons). The crude reaction mixture was filtered over celite® and flushed with 50 mL dichloromethane. After evaporation of the solvents and drying under reduced pressure the product **74** was obtained as a colorless solid (3.08 g, 2.46 mol, 99.5%).

IR (ATR): ν / cm^{-1} = 3305.5 (vw), 2924.5 (s), 2854.0 (m), 1740.6 (s), 1654.0 (s), 1539.0 (m), 1454.6 (w), 1371.5 (w), 1226.7 (vs), 1145.2 (vs), 1020.8 (m), 909.9 (w), 759.8 (w), 735.7 (w), 700.3 (m), 536.7 (vw).

^{1}H NMR (500 MHz, $CDCl_3$): δ / ppm = 7.25 – 7.10 (m, 5 H, CH_{Ar}^{1}), 6.21 (t, J = 5.9 Hz, 1 H, NH^{2}), 6.07 (t, J = 6.0 Hz, 1 H, NH^{2}), 5.76 – 5.60 (m, 0.5 H, CH^{3a}), 5.30 – 5.21 (m, 2.5 H, CH^{3b}, CH^{4}), 5.18 – 5.06 (m, 2 H, CH_2^{5}), 4.68 – 4.43 (m, 6 H, CH_2^{6}), 3.42 – 3.35 (m, 1 H, CH^{7}), 3.29 – 2.93 (m, 6 H, CH_2^{8}), 2.81 – 2.60 (m, 4 H, CH_2^{9}), 2.39 – 2.22 (m, 6 H, CH_2^{10}), 1.90 – 1.68 (m, 2 H, CH_2^{11}), 1.62 – 0.98 (m, 12H, CH_2^{12}, CH_3^{13}), 0.80 (t, J = 6.8 Hz, 3 H, CH_3^{14}).

^{13}C NMR (126 MHz, $CDCl_3$): δ / ppm = 177.35, 172.71, 172.58, 172.42, 171.52, 170.93, 170.56, 169.76, 169.04, 168.82, 141.66, 141.17, 128.54, 128.31, 127.97, 127.14, 127.06, 77.88, 74.85, 70.59, 59.68 (t, J = 26.9 Hz), 41.55, 41.27, 39.47, 39.34, 39.27, 34.48, 34.35, 34.29, 33.96, 31.94, 31.92, 29.58, 29.53, 29.49, 29.43, 29.35, 29.31, 29.28, 29.22, 29.17, 29.14, 29.09, 29.03, 28.79, 26.95, 26.86, 26.85, 26.80, 25.04, 24.98, 24.96, 24.90, 24.87, 22.76, 18.06, 17.61, 15.33, 14.21.

^{19}F NMR (376 MHz, $CDCl_3$): δ / ppm = -80.81 (t, J = 9.3 Hz, 3 F, CF_3^{15}), -119.20 – -121.40 (m, 2 F, CF_2^{16}), -126.52 – -130.07 (m, 2 F, CF_2^{16}). Total integral of CF_2 region normalized with respect to the CF_3^{15} group = 4.

ESI-MS [m/z]: $[M + H]^+$ calculated for $^{12}C_{64}{}^{1}H_{102}{}^{16}O_{13}{}^{14}N_3{}^{19}F_7$, 1254.7374; found, 1254.7351, Δ = 2.3 mmu.

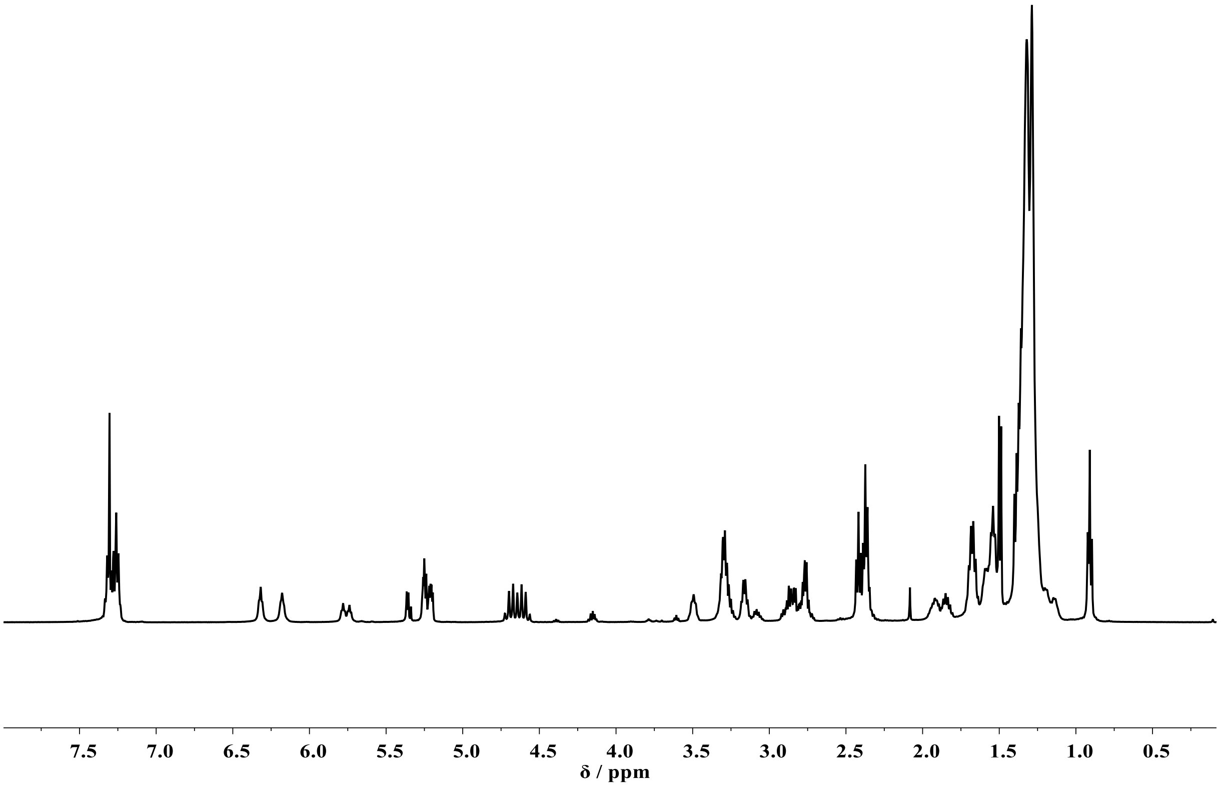

Supplementary Figure 104: ^{1}H-NMR of compound 74 measured in $CDCl_3$.

Passerini reaction

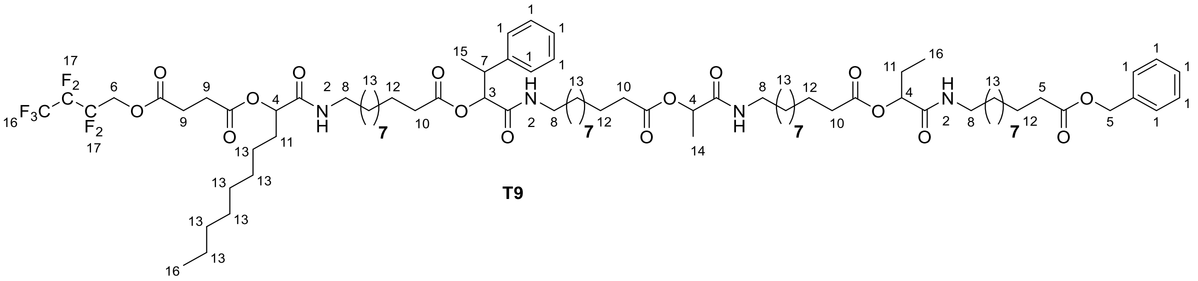

In a 50 mL round bottom flask, 428 g of **74** (341 µmol, 1.00 eq.) was stirred in 2.00 mL DCM. Subsequently, 36.7 µL propionaldehyde **14l** (29.7 mg, 512 µmol, 3.00 eq.) and 154 mg of the monomer **M1** (512 µmol, 1.50 eq.) were added. The resulting reaction mixture was stirred at room temperature for 1 day. Afterwards, the crude mixture was dried under reduced pressure. The residue was adsorbed onto celite® and purified *via* column chromatography on silica gel eluting with a gradual solvent mixture of ethyl acetate and cyclohexane (3:1 → 1:1) to yield the passerini product **T9** as a pale highly viscous oil. (372 mg, 230 µmol, 67.6%).

R_f = 0.52 in cyclohexane / ethyl acetate (1:1).

IR (ATR): ν / cm^{-1} = 3304.8 (w), 2924.6 (s), 2853.8 (m), 1738.8 (s), 1655.0 (s), 1535.6 (m), 1455.2 (w), 1373.2 (w), 1227.0 (s), 1146.1 (vs), 1020.8 (m), 735.6 (w), 699.1 (m).

^1H NMR (500 MHz, $CDCl_3$): δ / ppm = 7.41 – 7.16 (m, 10 H, CH_{Ar}^1), 6.25 (t, J = 5.8 Hz, 1 H, NH^2), 6.11 (t, J = 5.9 Hz, 1 H, NH^2), 6.05 (t, J = 6.0 Hz, 1 H, NH^2), 5.73 – 5.56 (m, 1 H, NH^2), 5.31 (d, J = 5.4 Hz, 0.5 H, CH^{3a}), 5.24 – 5.07 (m, 5.5 H, CH^{3a}, CH^4, CH_2^5), 4.69 – 4.51 (m, 2 H, CH_2^6), 3.49 – 3.38 (m, 1 H, CH^7), 3.32 – 2.97 (m, 8 H, CH_2^8), 2.89 – 2.66 (m, 4 H, CH_2^9), 2.43 – 2.25 (m, 8 H, CH_2^{10}), 1.95 – 1.75 (m, 4 H, CH_2^{11}), 1.71 – 1.40 (m, 19 H, $CH_2^{12,13}$, CH_3^{14}), 1.38 – 1.06 (m, 28 H, CH_2^{13}, CH_3^{15}), 0.95 – 0.84 (m, 6 H, CH_3^{16}).

^{13}C NMR (126 MHz, $CDCl_3$): δ / ppm = 173.83, 172.61, 172.57, 172.46, 172.41, 171.50, 170.91, 170.46, 169.75, 169.64, 168.87, 168.66, 141.72, 141.19, 136.25, 128.67, 128.53, 128.32, 128.30, 128.29, 127.97, 127.13, 127.05, 77.85, 74.93, 74.87, 70.58, 66.20, 59.67 (t, J = 26.5 Hz), 41.57, 41.28, 39.43, 39.35, 39.32, 39.29, 39.20, 34.45, 34.43, 34.35, 34.30, 31.94, 29.70, 29.68, 29.59, 29.56, 29.49, 29.47, 29.41, 29.35, 29.33, 29.28, 29.23, 29.18, 29.16, 29.04, 28.78, 26.95, 26.86, 26.80, 25.22, 25.08, 25.06, 25.00, 24.98, 24.91, 22.76, 17.62, 15.30, 14.22, 9.15.

^{19}F NMR (376 MHz, $CDCl_3$): δ / ppm = -80.81 (t, J = 9.1 Hz, 3 F, CF_3^{16}), -119.77 – -123.82 (m, 2 F, CF_2^{17}), -124.53 – -130.56 (m, 2 F, CF_2^{17}). Total integral of CF_2 region normalized with respect to the CF_3^{17} group = 4.

ESI-MS [*m/z*]: $[M + H]^+$ calculated for $^{12}C_{86}{}^1H_{135}{}^{16}O_{16}{}^{14}N_4{}^{19}F_7$, 1613.9834; found, 1613.9821, Δ = 1.3 mmu.

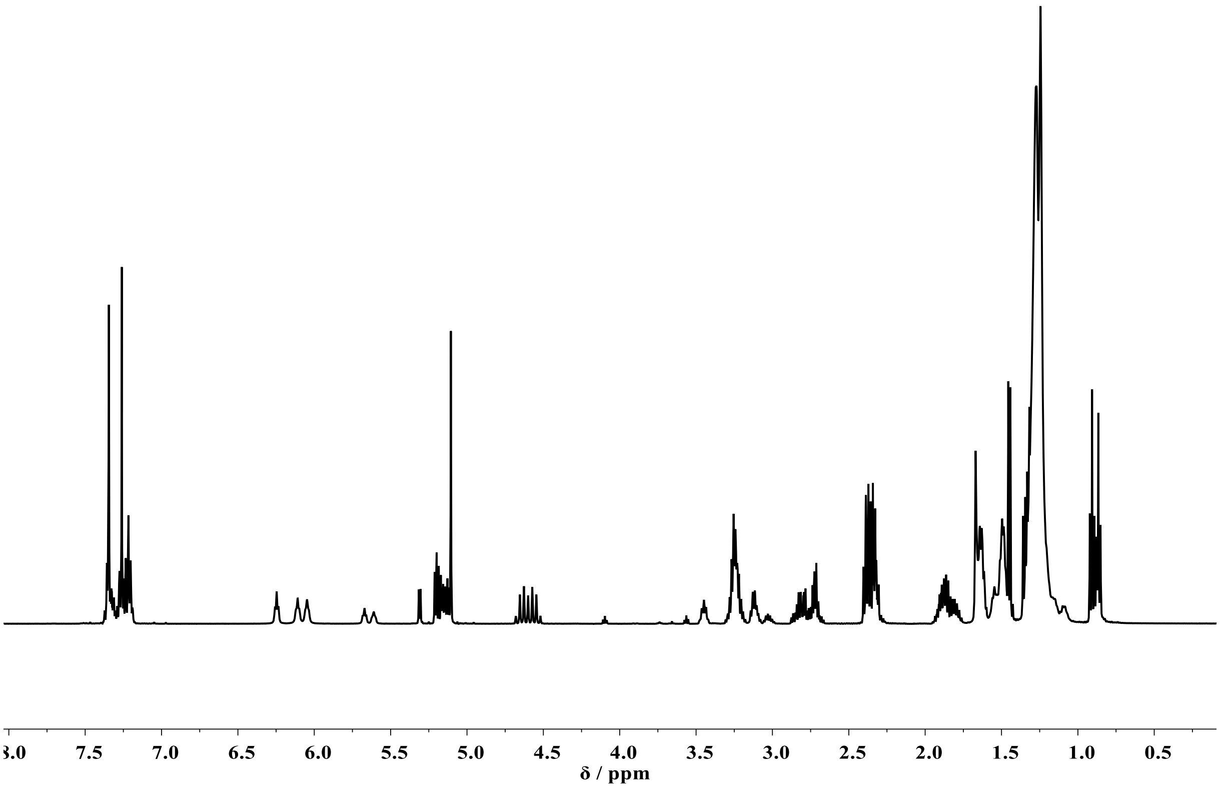

Supplementary Figure 105: ^{1}H-NMR of compound T9 measured in $CDCl_3$.

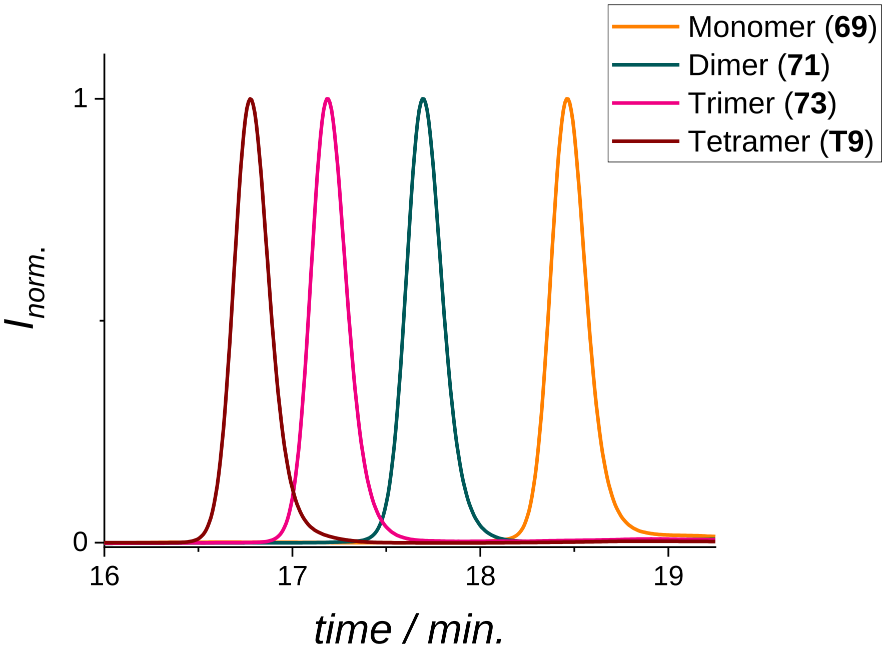

Supplementary Figure 106: SEC traces of the intermediates after each P3CR in the synthesis of product T9.

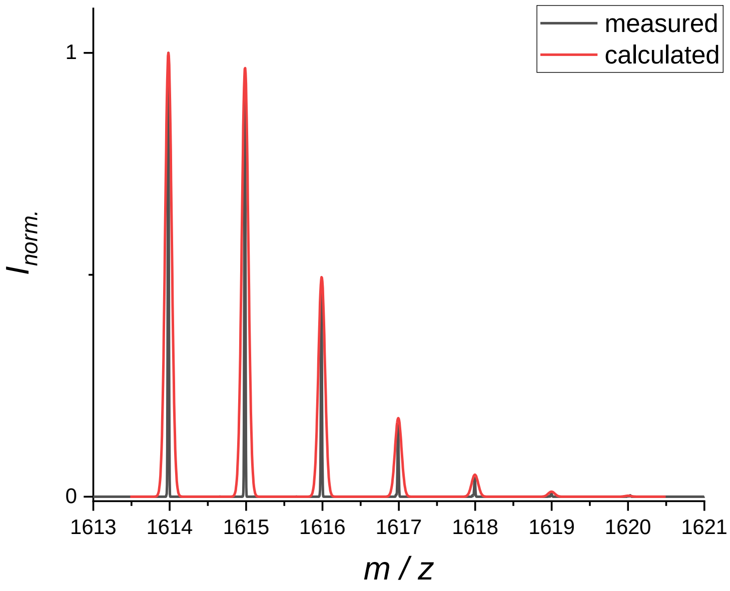

Supplementary Figure 107: High resolution ESI-MS measurement of T9. The observed isotopic pattern is compared with the calculated isotopic pattern obtained from mMass (red).

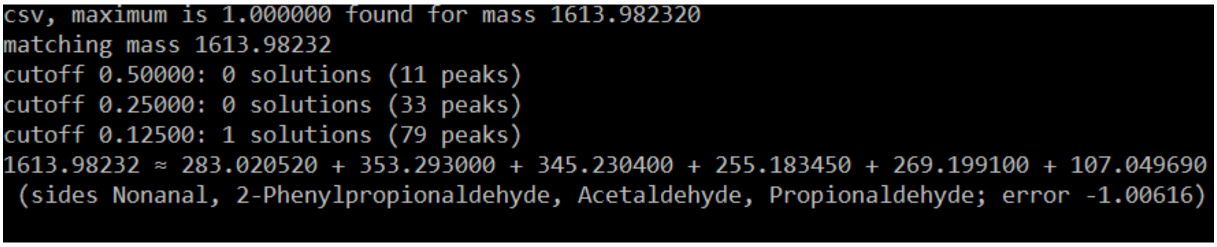

```
csv, maximum is 1.000000 found for mass 1613.982320
matching mass 1613.98232
cutoff 0.50000: 0 solutions (11 peaks)
cutoff 0.25000: 0 solutions (33 peaks)
cutoff 0.12500: 1 solutions (79 peaks)
1613.98232 ≈ 283.020520 + 353.293000 + 345.230400 + 255.183450 + 269.199100 + 107.049690
 (sides Nonanal, 2-Phenylpropionaldehyde, Acetaldehyde, Propionaldehyde; error -1.00616)
```

Supplementary Figure 108: Screenshot of the automated read-out of T9.

1.3.5.2 Synthesis of hexamer H3

Deprotection

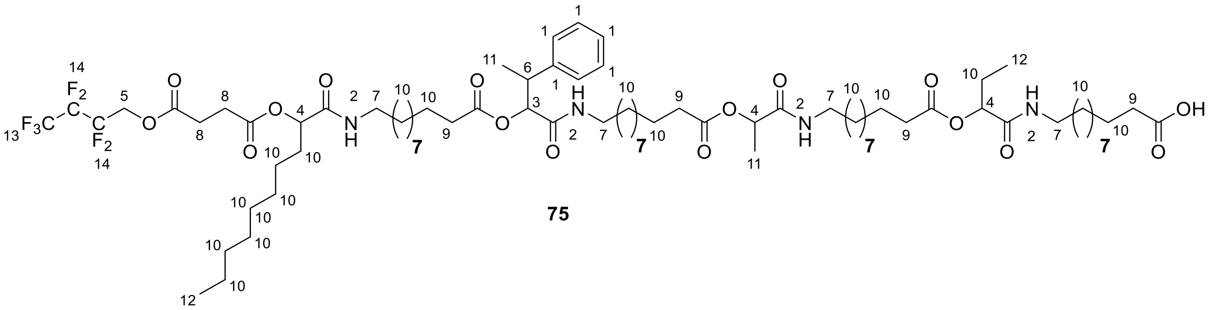

In a 25 mL round bottom flask, 323 mg of **T9** (200 µmol, 1.00 eq.) was dissolved in 3.00 mL ethyl acetate and 3.00 mL THF. Subsequently, 64.5 mg (20 wt.-%) palladium on activated charcoal **16** were added to the solution. The resulting mixture was purged with hydrogen gas and stirred for one day at room temperature under hydrogen atmosphere (3 balloons). The crude reaction mixture was filtered over celite® and flushed with 50 mL dichloromethane. After evaporation of the solvents and drying under reduced pressure the product **75** was obtained as a colorless solid (296 mg, 1940 µmol, 97.1%).

IR (ATR): ν / cm^{-1} = 3300.1 (vw), 2926.1 (w), 2854.6 (vw), 1741.9 (w), 1655.1 (w), 1541.6 (vw), 1457.2 (vw), 1228.3 (w), 1147.3 (vw), 700.5 (vw), 426.6 (vw).

^{1}H NMR (400 MHz, $CDCl_3$): δ / ppm = 7.38 – 7.18 (m, 5 H, CH_{Ar}^{1}), 6.31 (t, J = 5.9 Hz, 1 H, NH^{2}), 6.22 (t, J = 5.9 Hz, 1 H, NH^{2}), 6.13 (t, J = 6.0 Hz, 1 H, NH^{2}), 5.81 – 5.61 (m, 1 H, NH^{2}), 5.36 (d, J = 5.5 Hz, 0.5 H, CH^{3a}), 5.29 – 5.12 (m, 3.5 H, CH^{3b}, CH^{4}), 4.79 – 4.51 (m, 2 H, CH_2^{5}), 3.61 – 3.43 (m, 1 H, CH^{6}), 3.38 – 2.99 (m, 8 H, CH_2^{7}), 2.95 – 2.67 (m, 4 H, CH_2^{8}), 2.48 – 2.26 (m, 8 H, CH_2^{9}), 2.02 – 1.07 (m, 86 H, CH_2^{10}, CH_3^{11}), 1.04 – 0.82 (m, 6 H, CH_3^{12}).

^{13}C NMR (126 MHz, $CDCl_3$): δ / ppm = 176.95, 172.65, 172.61, 172.51, 172.48, 171.52, 170.93, 170.66, 169.86, 169.74, 168.99, 168.77, 141.69, 141.18, 128.54, 128.31, 127.97, 127.14, 127.07, 77.85, 74.95, 74.86, 70.56, 59.67 (t, J = 26.9 Hz), 41.57, 41.27, 39.46, 39.41, 39.30, 39.23, 34.44, 34.35, 34.29, 33.94, 31.93, 29.64, 29.57, 29.53, 29.49, 29.41, 29.35, 29.33, 29.26, 29.23, 29.20, 29.08, 29.03, 28.78, 26.95, 26.94, 26.86, 26.79, 25.18, 25.11, 24.98, 24.90, 22.76, 18.07, 17.62, 15.30, 14.21, 9.15.

^{19}F NMR (376 MHz, $CDCl_3$): δ / ppm = -80.81 (t, J = 9.2 Hz, 3 F, CF_3^{13}), -117.22 – -122.54 (m, 2 F, CF_2^{14}), -125.88 – -129.43 (m, 2 F, CF_2^{14}). Total integral of CF_2 region normalized with respect to the CF_3^{13} group = 4.

ESI-MS [*m/z*]: $[M + H]^+$ calculated for $^{12}C_{79}{}^{1}H_{129}{}^{16}O_{16}{}^{14}N_4{}^{19}F_7$, 1523.9365; found, 1523.9361, Δ = 0.4 mmu.

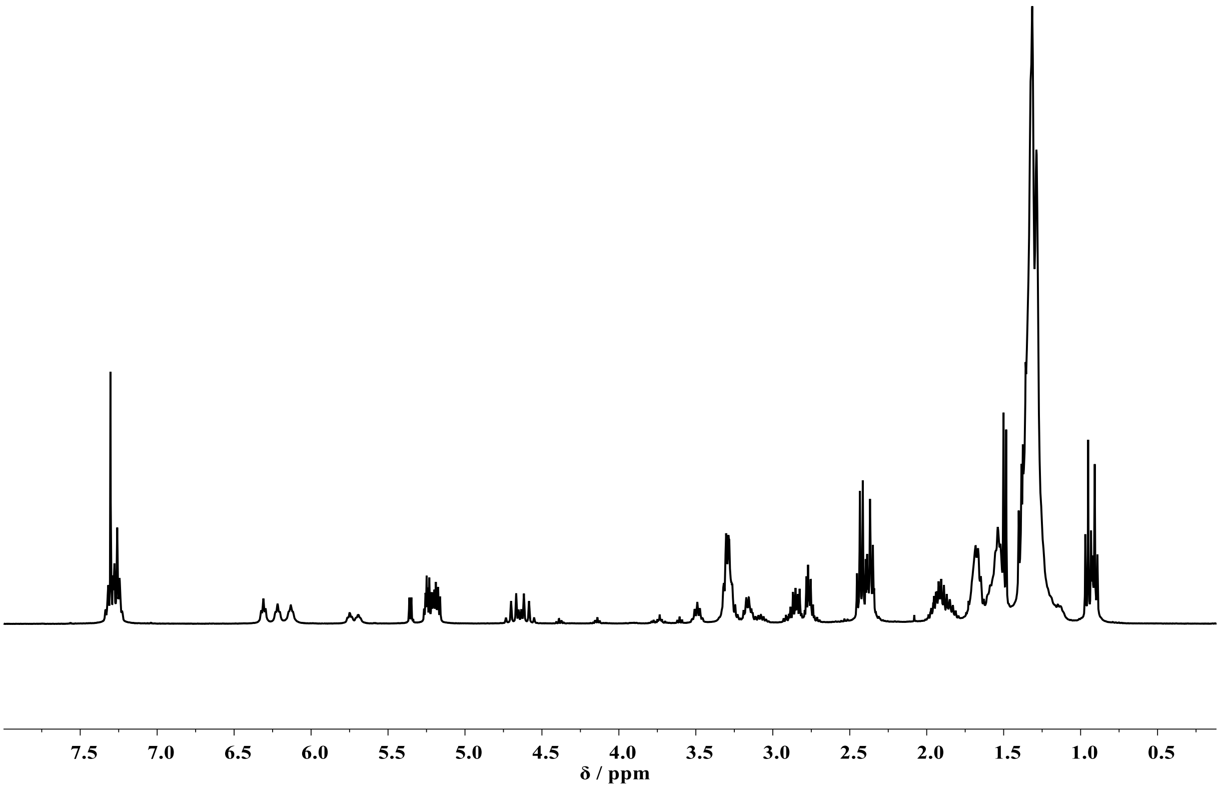

Supplementary Figure 109: ^{1}H-NMR of compound 75 measured in $CDCl_3$.

Passerini reaction

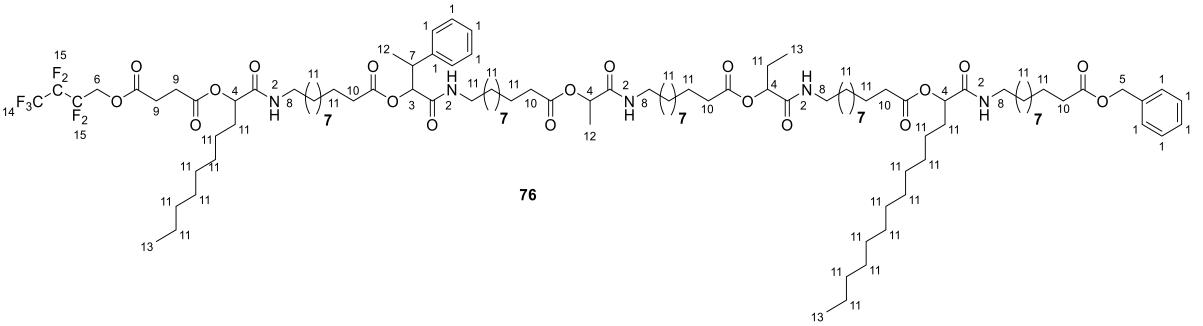

In a 50 mL round bottom flask, 260 mg of **75** (171 µmol, 1.00 eq.) was stirred in 2.00 mL DCM. Subsequently, 40.5 µL tridecanal **14d** (33.8 mg, 171 µmol, 1.00 eq.) and 51.4 mg of the monomer **M1** (171 µmol, 1.50 eq.) were added. The resulting reaction mixture was stirred at room temperature for 1 day. Afterwards, the crude mixture was dried under reduced pressure. The residue was adsorbed onto celite® and purified *via* column chromatography on silica gel eluting with a gradual solvent mixture of ethyl acetate and cyclohexane (3:1 → 1:1) to yield the passerini product **76** as a pale highly viscous oil. (183 mg, 90.6 µmol, 53.1%).

R_f = 0.33 in cyclohexane / ethyl acetate (1:1).

IR (ATR): ν / cm^{-1} = 2925.3 (m), 2854.3 (m), 1741.7 (w), 1649.3 (m), 1587.1 (m), 1534.1 (s), 1454.9 (m), 1337.8 (w), 1225.8 (s), 1145.7 (m), 981.3 (w), 782.1 (w), 725.8 (w), 699.6 m), 635.1 (w).

^{1}H NMR (400 MHz, $CDCl_3$): δ / ppm = 7.39 – 7.14 (m, 10 H, $CH_{Ar}{}^{1}$), 6.27 (t, J = 5.8 Hz, 1 H, NH^{2}), 6.19 – 5.99 (m, 3 H, NH^{2}), 5.76 – 5.60 (m, 1 H, NH^{2}), 5.29 (d, J = 5.5 Hz, 0.5 H, CH^{3a}), 5.24 – 5.04 (m, 6.5 H, CH^{3a}, CH^{4}, $CH_2{}^{5}$), 4.71 – 4.47 (m, 2 H, $CH_2{}^{6}$), 3.52 – 3.37 (m, 1 H, CH^{7}), 3.33 – 2.98 (m, 10 H, $CH_2{}^{8}$), 2.90 – 2.63 (m, 4 H, $CH_2{}^{9}$), 2.42 – 2.26 (m, 10 H, $CH_2{}^{10}$), 1.99 – 1.72 (m, 8 H, $CH_2{}^{11}$), 1.68 – 1.01 (m, 116 H, $CH_2{}^{11}$, $CH_3{}^{12}$), 0.96 – 0.80 (m, 9 H, $CH_3{}^{13}$).

^{13}C NMR (101 MHz, $CDCl_3$): δ / ppm = 172.38, 171.45, 170.87, 170.45, 169.97, 169.75, 169.62, 168.85, 168.64, 141.69, 141.18, 136.21, 128.62, 128.48, 128.26, 128.25, 128.23, 127.92, 127.08, 127.01, 77.81, 74.89, 74.82, 74.03, 70.52, 66.15, 59.62 (t, J = 26.7 Hz), 41.55, 41.26, 39.40, 39.31, 39.28, 39.17, 34.31, 34.25, 32.00, 31.90, 29.75, 29.72, 29.67, 29.63, 29.52, 29.43, 29.35, 29.31, 29.23, 29.19, 29.19, 29.11, 29.00, 28.74, 26.91, 26.90, 26.82, 26.76, 25.18, 25.04, 24.95, 24.86, 22.77, 22.71, 18.05, 17.59, 15.27, 14.20, 14.16, 9.12.

^{19}F NMR (376 MHz, $CDCl_3$): δ / ppm = -80.82 (t, J = 9.1 Hz, 3 F, $CF_3{}^{14}$), -119.33 – -121.63 (m, 2 F, $CF_2{}^{15}$), -127.21 – -128.37 (m, 2 F, $CF_2{}^{15}$). Total integral of CF_2 region normalized with respect to the $CF_3{}^{16}$ group = 4.

ESI-MS [m/z]: [M + H]$^+$ calculated for $^{12}C_{111}{}^{1}H_{182}{}^{16}O_{19}{}^{14}N_5{}^{19}F_7$, 2023.3390; found, 2023.3392, Δ = 0.2 mmu.

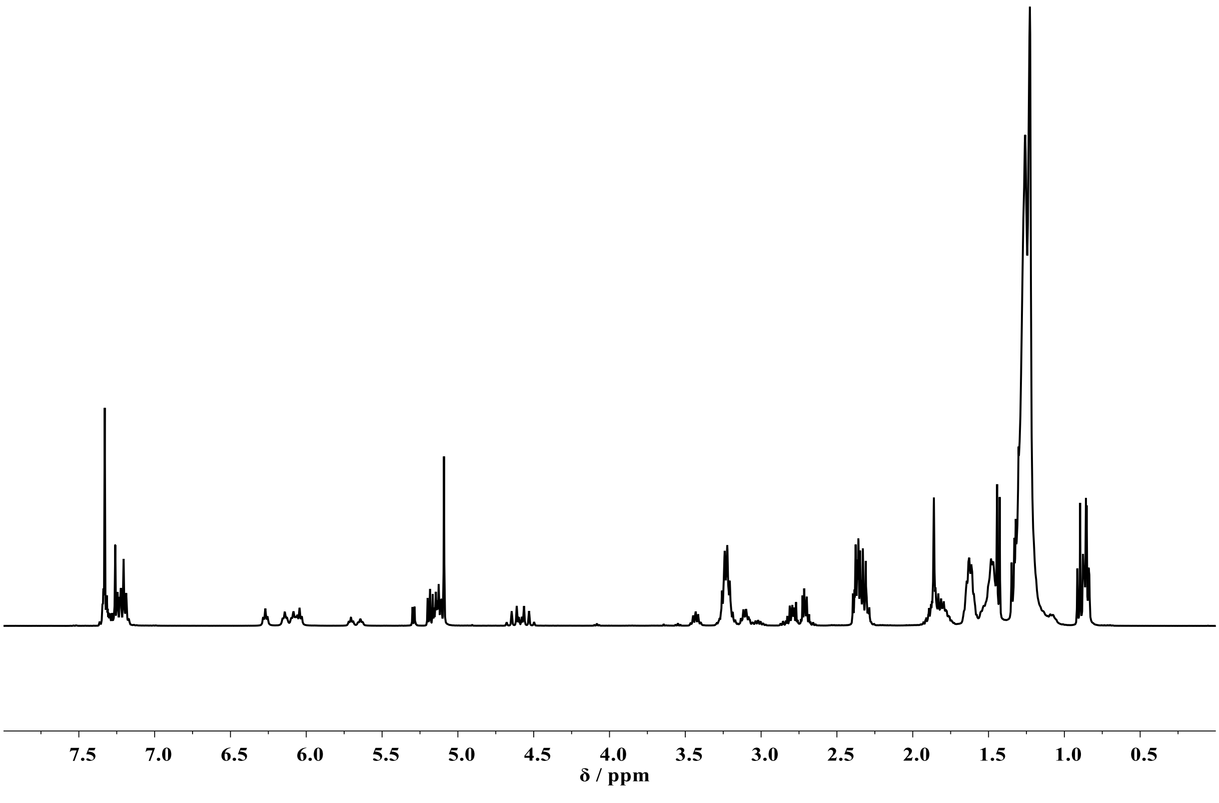

Supplementary Figure 110: ^{1}H-NMR of compound 76 measured in $CDCl_3$.

Deprotection

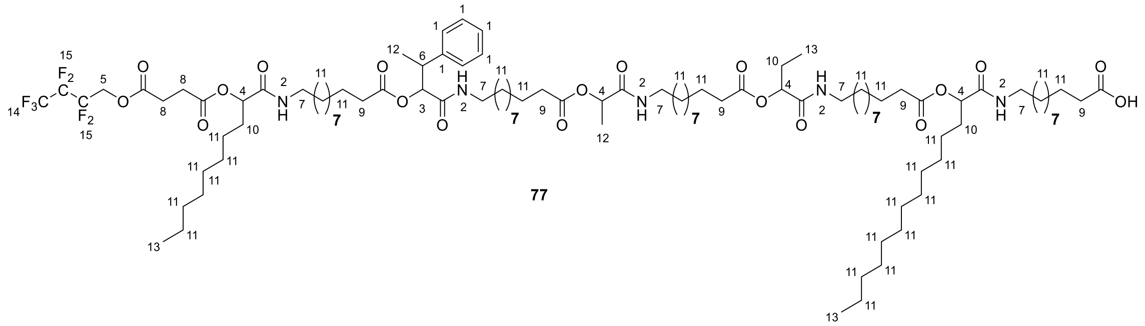

77

In a 25 mL round bottom flask, 77.1 mg of **76** (38.0 µmol, 1.00 eq.) was dissolved in 2.00 mL ethyl acetate and 2.00 mL THF. Subsequently, 15.4 mg (20 wt.-%) palladium on activated charcoal **16** were added to the solution. The resulting mixture was purged with hydrogen gas and stirred for one day at room temperature under hydrogen atmosphere (3 balloons). The crude reaction mixture was filtered over celite® and flushed with 50 mL dichloromethane. After evaporation of the solvents and drying under reduced pressure the product **77** was obtained as a colorless solid (67.2 mg, 34.7 µmol, 91.2%).

IR (ATR): ν / cm^{-1} = 3291.0 (vw), 2923.9 (s), 2853.7 (m), 1741.6 (m), 1650.2 (s), 1535.7 (s), 1456.1 (m), 1373.8 (w), 1226.7 (s), 1145.4 (s), 1021.5 (w), 980.5 (w), 781.8 (vw), 723.6 (w), 699.8 (m), 634.8 (w).

^{1}H NMR (400 MHz, $CDCl_3$): δ / ppm = 7.41 – 7.15 (m, 5 H, $CH_{Ar}{}^{1}$), 6.33 (t, J = 5.8 Hz, 1 H, NH^{2}), 6.26 – 6.16 (m, 2 H, NH^{2}), 6.13 (t, J = 5.9 Hz, 1 H, NH^{2}), 5.74 (dt, J = 24.0, 6.1 Hz, 1 H, NH^{2}), 5.35 (d, J = 5.5 Hz, 0.5 H, CH^{3a}), 5.28 – 5.14 (m, 4.5 H, CH^{3b}, CH^{4}), 4.75 – 4.52 (m, 2 H, $CH_2{}^{5}$), 3.56 – 3.43 (m, 1 H, CH^{6}), 3.38 – 3.01 (m, 10 H, $CH_2{}^{7}$), 2.95 – 2.68 (m, 4 H, $CH_2{}^{8}$), 2.49 – 2.30 (m, 10 H, $CH_2{}^{9}$), 2.00 – 1.77 (m, 6 H, $CH_2{}^{10}$), 1.75 – 1.05 (m, 118 H, $CH_2{}^{11}$, $CH_3{}^{12}$), 1.00 – 0.81 (m, 9 H, $CH_3{}^{13}$).

^{13}C NMR (101 MHz, $CDCl_3$): δ / ppm = 176.94, 172.66, 172.64, 172.49, 172.43, 171.50, 170.91, 170.59, 170.09, 169.95, 169.72, 168.96, 168.74, 141.68, 141.17, 128.52, 128.29, 127.95, 127.12, 127.04, 77.84, 77.36, 74.91, 74.84, 74.07, 70.54, 59.65 (t, J = 26.6 Hz), 41.56, 39.44, 39.37, 39.29, 39.21, 34.43, 34.40, 34.33, 34.28, 33.97, 32.03, 32.00, 31.92, 29.78, 29.76, 29.74, 29.66, 29.63, 29.58, 29.55, 29.51, 29.46, 29.45, 29.34, 29.31, 29.30, 29.26, 29.23, 29.19, 29.16, 29.14, 29.10, 29.02, 28.77, 26.94, 26.91, 26.88, 26.84, 26.78, 25.19, 25.09, 25.05, 24.98, 24.97, 24.89, 22.80, 22.74, 18.07, 17.60, 15.29, 14.24, 14.20, 9.14.

ESI-MS [*m/z*]: $[M + H]^{+}$ calculated for $^{12}C_{104}{}^{1}H_{176}{}^{16}O_{19}{}^{14}N_{5}{}^{19}F_{7}$, 193.2921; found, 193.2935, Δ = 1.4 mmu.

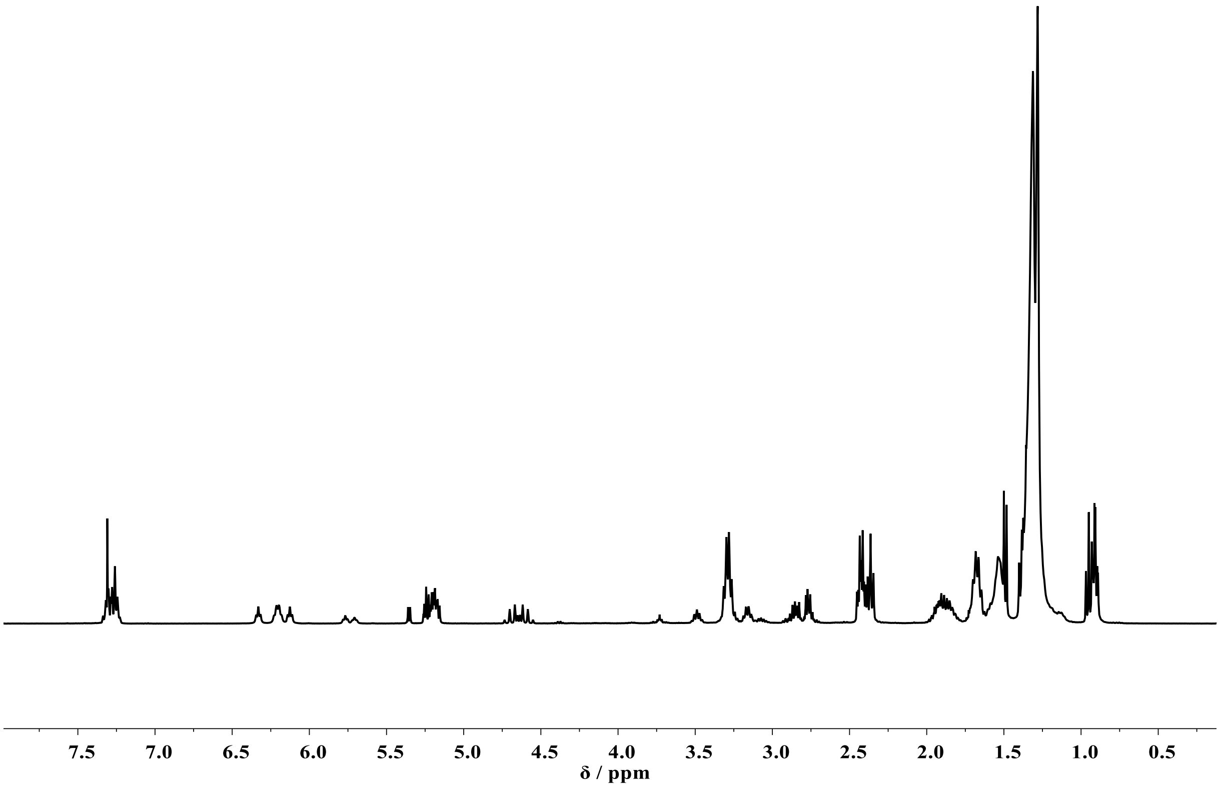

Supplementary Figure 111: ^{1}H-NMR of compound 77 measured in $CDCl_3$.

Passerini reaction

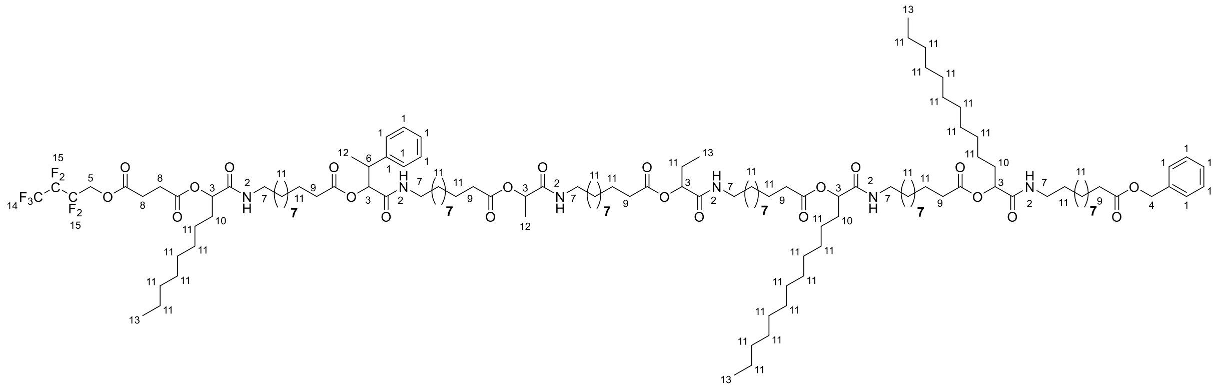

In a 10 mL round bottom flask, 56.2 mg of **77** (29.1 µmol, 1.00 eq.) was stirred in 1.00 mL DCM. Subsequently, 8.04 mg dodecanal **14h** (43.6 µmol, 1.00 eq.) and 13.1 mg of the monomer **M1** (43.6 µmol, 1.50 eq.) were added. The resulting reaction mixture was stirred at room temperature for 2 days. Afterwards, the crude mixture was dried under reduced pressure. The residue was adsorbed onto celite® and purified *via* column chromatography on silica gel eluting with a gradual solvent mixture of ethyl acetate and cyclohexane (3:1 → 1:1) to yield the passerini product **H3** as a pale highly viscous oil. (28.7 mg, 11.8 µmol, 40.5%).

R_f = 0.46 in cyclohexane / ethyl acetate (1:1)

IR (ATR): ν / cm^{-1} = 3138.8 (w), 2962.1 (w), 1648.2 (w), 1587.1 (m), 1535.6 (m), 1456.1 (w), 1338.2 (w), 1278.7 (w), 1224.0 (w), 1146.3 (w), 1063.5 (w), 981.8 (vw), 791.7 (vw), 726.0 (vw), 700.9 (w), 635.7 (vw).

^{1}H NMR (400 MHz, $CDCl_3$): δ / ppm = 7.33 – 7.09 (m, 10 H, CH_{Ar}^{1}), 6.18 (t, J = 5.7 Hz, 1 H, NH^{2}), 6.09 – 5.90 (m, 4 H, NH^{2}), 5.66 – 5.50 (m, 1 H, NH^{2}), 5.31 – 5.00 (m, 8 H, CH^{3}, CH_2^{4}), 4.66 – 4.43 (m, 2 H, CH_2^{5}), 3.47 – 3.32 (m, 1 H, CH^{6}), 3.27 – 2.91 (m, 12 H, CH_2^{7}), 2.88 – 2.59 (m, 4 H, CH_2^{8}), 2.38 – 2.23 (m, 12 H, CH_2^{9}), 1.91 – 1.67 (m, 8 H, CH_2^{10}), 1.66 – 0.93 (m, 152 H, CH_2^{11}, CH_3^{12}), 0.89 – 0.71 (m, 12 H, CH_3^{13}).

^{13}C NMR (101 MHz, $CDCl_3$): δ / ppm = 173.83, 172.62, 172.43, 171.51, 170.91, 170.49, 170.02, 169.79, 169.65, 168.88, 136.27, 128.69, 128.55, 128.33, 128.31, 127.98, 77.88, 77.36, 74.95, 74.90, 74.09, 70.60, 66.22, 41.60, 39.46, 39.37, 39.34, 39.22, 34.47, 32.06, 31.96, 29.81, 29.79, 29.77, 29.72, 29.70, 29.61, 29.59, 29.50, 29.41, 29.37, 29.26, 29.06, 28.81, 27.06, 26.98, 26.88, 26.82, 25.24, 25.10, 25.02, 25.01, 24.93, 22.83, 22.78, 18.11, 15.33, 14.27, 14.23, 9.17.

^{19}F NMR (376 MHz, $CDCl_3$): δ / ppm = -83.96 – -86.17 (m, 3 F, CF_3^{14}), -124.42 – -125.22 (m, 2 F, CF_2^{15}), -131.64 – -132.55 (m, 2 F, CF_2^{15}). Total integral of CF_2 region normalized with respect to the CF_3^{16} group = 4.

ESI-MS [*m/z*]: $[M + H]^+$ calculated for $^{12}C_{135}{}^{1}H_{227}{}^{16}O_{22}{}^{14}N_6{}^{19}F_7$, 2418.6789; found, 2418.6818, Δ = 2.9 mmu.

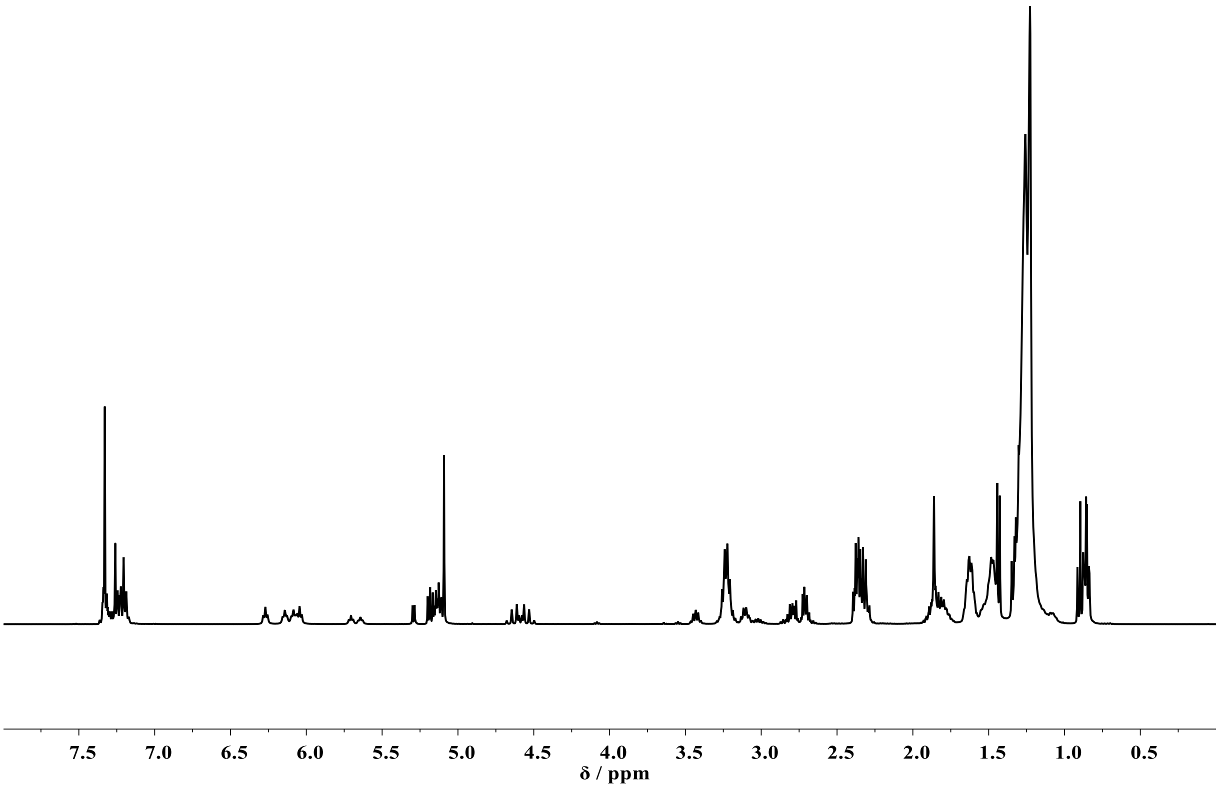

Supplementary Figure 112: ^{1}H-NMR of compound H3 measured in $CDCl_3$.

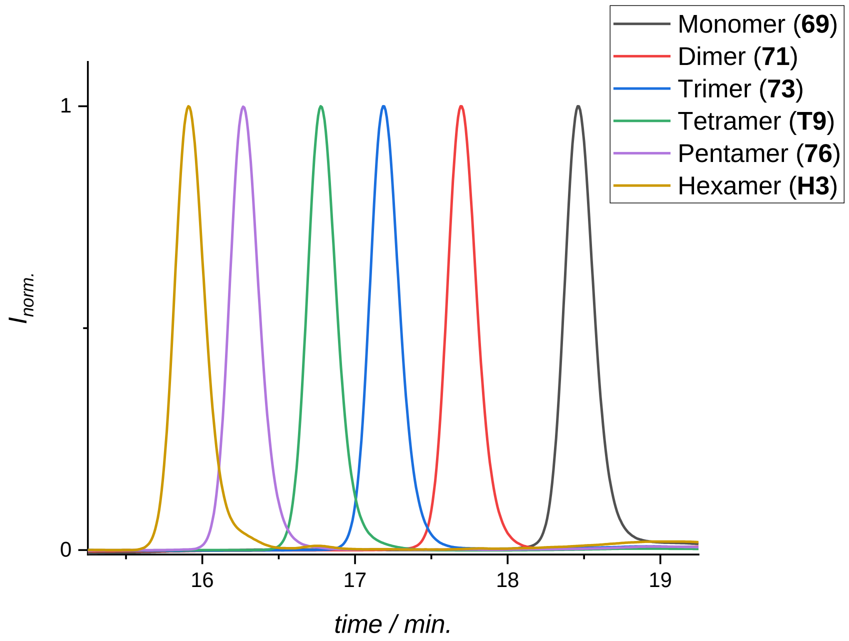

Supplementary Figure 113: SEC traces of the intermediates after each P3CR in the synthesis of product H3.

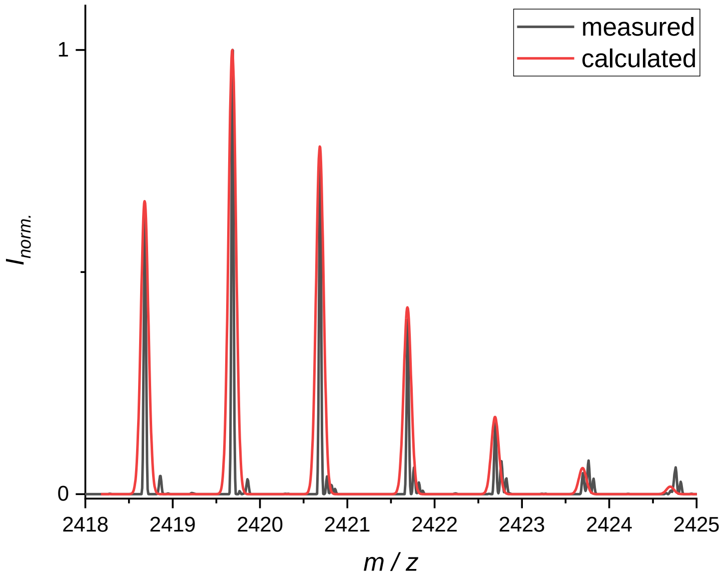

Supplementary Figure 114: High resolution ESI-MS measurement of H3. The observed isotopic pattern is compared with the calculated isotopic pattern obtained from mMass (red).

```
matching mass 2418.68173
cutoff 0.50000: 0 solutions (7 peaks)
cutoff 0.25000: 0 solutions (12 peaks)
cutoff 0.12500: 0 solutions (34 peaks)
cutoff 0.06250: 0 solutions (68 peaks)
cutoff 0.03125: 0 solutions (117 peaks)
cutoff 0.01562: 0 solutions (217 peaks)
cutoff 0.00781: 0 solutions (328 peaks)
cutoff 0.00391: 1 solutions (513 peaks)
2418.68173 ≈ 283.020520 + 353.293000 + 345.230400 + 255.183450 + 269.199100 + 409.355600 + 395.339950 + 107.049690 (sides
 Nonanal, 2-Phenylpropionaldehyde, Acetaldehyde, Propionaldehyde, Tridecanal, Dodecanal; error -1.01002)
Press ENTER to quit ...
```

Supplementary Figure 115: Screenshot of the automated read-out of H3.

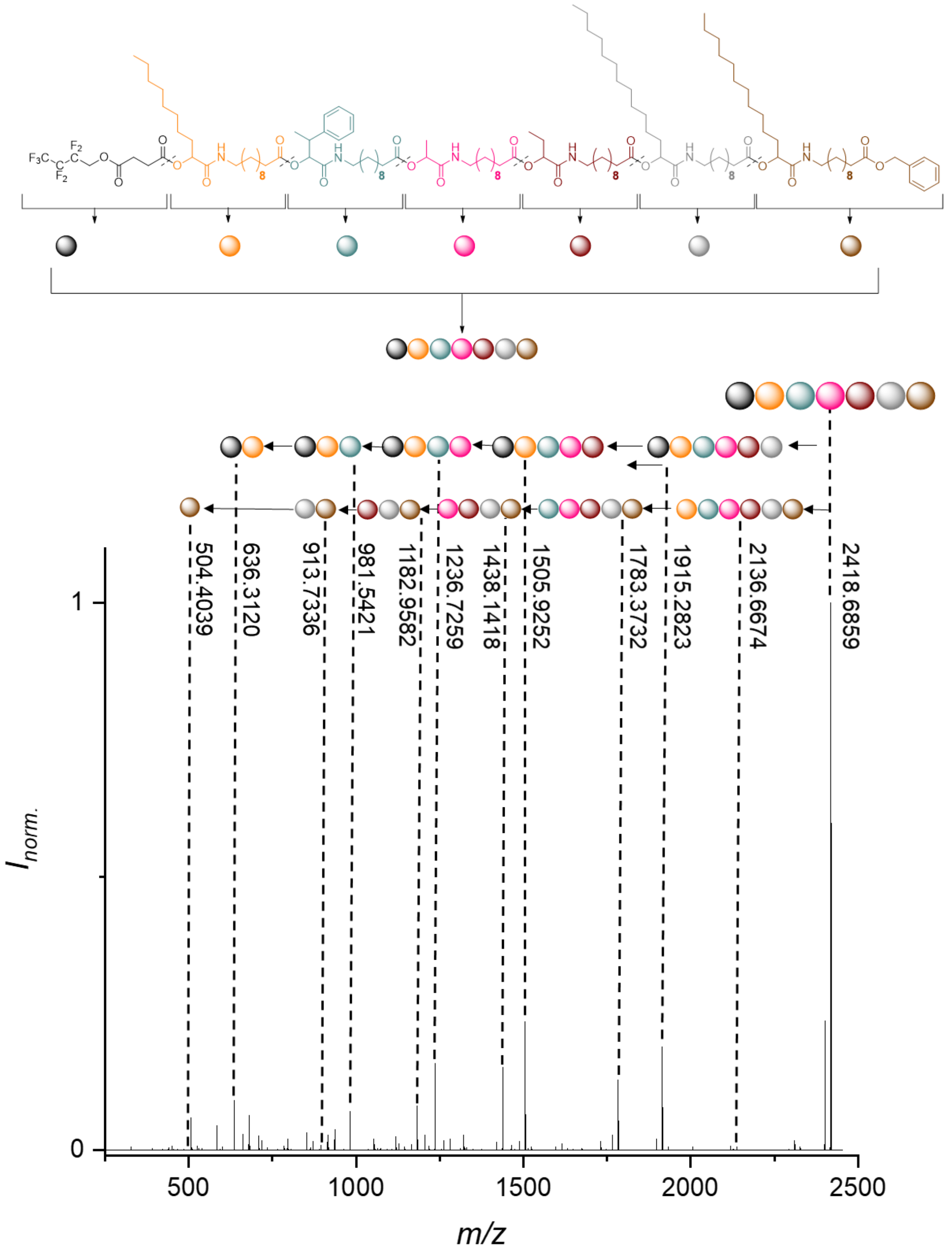

Supplementary Figure 116: Read-out of the sequence-defined hexamer H2. Read-out of the hexamer H3 via tandem ESI-MS/MS with an NCE of 17. In the spectrum, the read-out from both ends of the oligomer using the fragmentation next to the carbonyl are shown.

1.3.5.3 Synthesis of tetramer T10

Passerini reaction

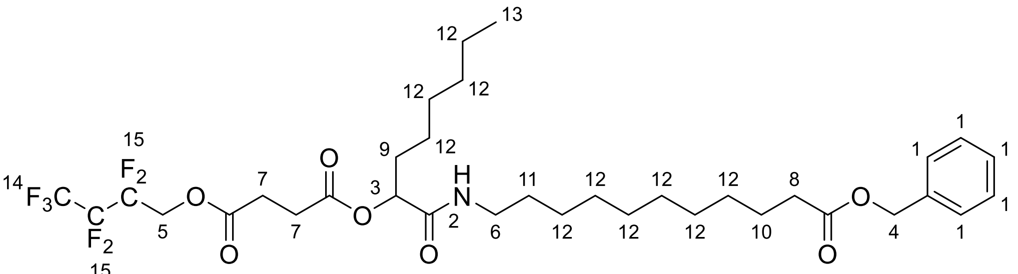

78

In a 50 mL round bottom flask 500 mg, **TAG2** (1.67 mmol, 1.00 eq.) was stirred in 5.00 mL DCM. Subsequently, 372 µL heptanal **14b** (321 mg, 2.50 mmol, 1.50 eq.) and 754 mg of the monomer **M1** (2.50 mmol, 1.50 eq.) were added. The resulting reaction mixture was stirred at room temperature for 2 days. Afterwards, the crude mixture was dried under reduced pressure. The residue was adsorbed onto celite® and purified *via* column chromatography on silica gel eluting with a gradual solvent mixture of ethyl acetate and cyclohexane (6:1 → 4:1) to yield the passerini product **78** as a pale highly viscous oil. (1.04 g, 1.46 mmol, 87.4%).

R_f = 0.38 in cyclohexane / ethyl acetate (3:1).

IR (ATR): ν / cm^{-1} = 3306.1 (vw), 2927.1 (m), 2856.0 (w), 1738.6 (s), 1657.5 (m), 1536.5 (w), 1456.0 (w), 1352.2 (w), 1226.3 (vs), 1144.2 (vs), 1019.9 (m), 909.7 (w), 735.9 (m), 697.2 (m).

^{1}H NMR (400 MHz, $CDCl_3$): δ / ppm = 7.41 – 7.29 (m, 5 H, CH_{Ar}^1), 6.22 (t, *J* = 5.8 Hz, 1 H, NH^2), 5.19 – 5.14 (m, 1 H, CH^3), 5.11 (s, 2 H, CH_2^4), 4.71 – 4.48 (m, 2 H, CH_2^5), 3.32 – 3.11 (m, 2 H, CH_2^6), 2.94 – 2.64 (m, 4 H, CH_2^7), 2.34 (t, *J* = 7.6 Hz, 2 H, CH_2^8), 1.95 – 1.76 (m, 2 H, CH_2^9), 1.67 – 1.57 (m, 2 H, CH_2^{10}), 1.54 – 1.43 (m, 2 H, CH_2^{11}), 1.39 – 1.13 (m, 20 H, CH_2^{12}), 0.87 (t, *J* = 6.6 Hz, 3 H, CH_3^{13}).

^{13}C NMR (101 MHz, $CDCl_3$): δ / ppm = 174.58, 172.23, 171.64, 170.37, 137.03, 129.42, 129.04, 75.64, 66.95, 60.45, 40.21, 35.21, 32.70, 32.49, 30.34, 30.30, 30.24, 30.13, 30.09, 29.99, 29.81, 29.77, 29.55, 27.72, 25.83, 25.69, 23.42, 14.90.

^{19}F NMR (376 MHz, $CDCl_3$): δ / ppm = -84.54 – -86.48 (m, 3 F, CF_3^{14}), -124.50 – -126.45 (m, 2 F, CF_2^{15}), -131.22 – -133.88 (m, 2 F, CF_2^{15}). Total integral of CF_2 region normalized with respect to the CF_3^{14} group = 4.

ESI-MS [*m/z*]: $[M + H]^+$ calculated for $^{12}C_{34}{}^{1}H_{48}{}^{16}O_7{}^{14}N^{19}F_7$, 716.3392; found, 716.3371, Δ = 2.1 mmu.

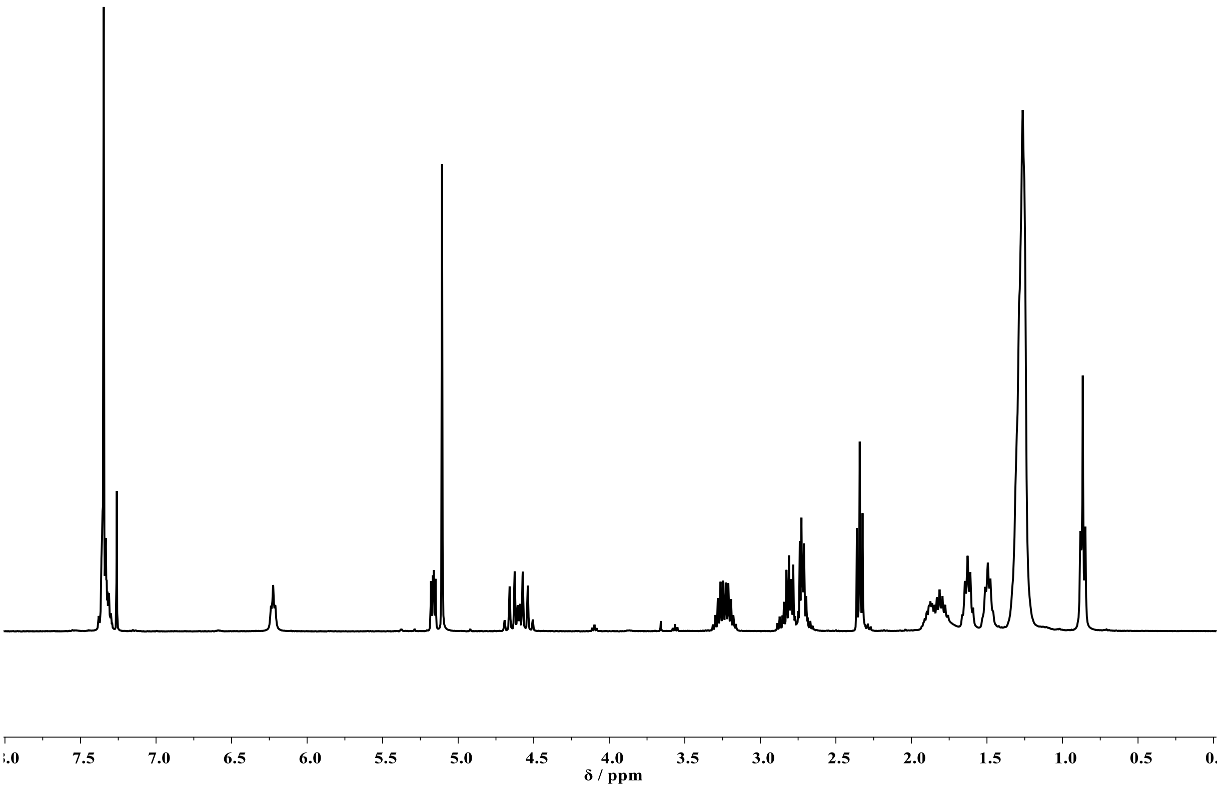

Supplementary Figure 117: ^{1}H-NMR of compound 78 measured in $CDCl_3$.

Deprotection

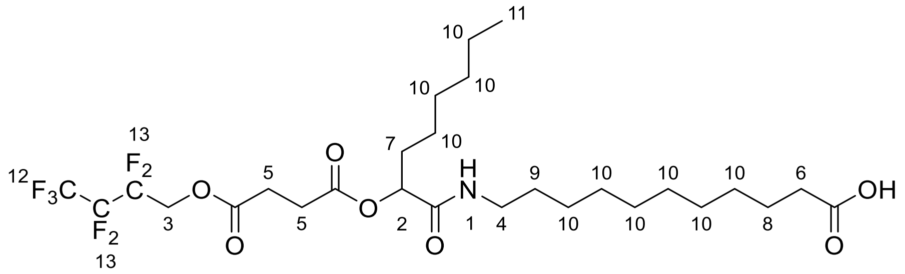

79

In a 50 mL round bottom flask, 995 mg of **78** (1.39 mmol, 1.00 eq.) were dissolved in 3.00 mL ethyl acetate and 3.00 mL THF. Afterwards, 199 mg (20 wt%) palladium on activated charcoal **16** were added. Subsequently, the mixture was purged with hydrogen (3 balloons) and stirred under hydrogen atmosphere overnight. The heterogeneous catalyst was filtered off and the solvent was evaporated under reduced pressure. The product **79** was obtained as a pale highly viscous oil in a yield of 96.4% (835 mg, 1.34 mmol).

IR (ATR): ν / cm^{-1} = 2926.9 (m), 2856.3 (m), 1743.1 (s), 1652.0 (m), 1542.6 (w), 1352.4 (w), 1226.0 (vs), 1179.7 (vs), 1144.2 (vs), 1020.0 (m), 909.4 (w), 735.9 (w), 628.5 (w).

^{1}H NMR (400 MHz, $CDCl_3$): *δ* / ppm = 6.27 (t, *J* = 5.8 Hz, 1 H, NH^{1}), 5.22 – 5.10 (m, 1 H, CH^{2}), 4.71 – 4.49 (m, 2 H, CH^{3}), 3.31 – 3.13 (m, 2 H, CH_2^{4}), 2.93 – 2.63 (m, 4 H, CH_2^{5}), 2.32 (t, *J* = 7.5 Hz, 2 H, CH_2^{6}), 2.00 – 1.74 (m, 2 H, CH_2^{7}), 1.69 – 1.56 (m, 2 H, CH_2^{8}), 1.54 – 1.43 (m, 2 H, CH_2^{9}), 1.39 – 1.16 (m, 20 H, CH_2^{10}), 0.98 – 0.80 (m, 3 H, CH_3^{11}).
^{13}C NMR (101 MHz, $CDCl_3$): *δ* / ppm = 178.91, 171.52, 170.93, 169.78, 74.88, 59.70 (t, *J* = 26.8 Hz), 39.48, 34.12, 31.92, 31.73, 29.47, 29.37, 29.26, 29.22, 29.10, 29.05, 29.01, 28.80, 26.90, 24.92, 24.84, 22.66, 14.14.179.16, 171.69, 169.77, 75.53, 44.09, 43.62, 39.41, 34.18, 31.22, 29.57, 29.46, 29.36, 29.23, 29.10, 27.46, 26.92, 24.83, 22.35, 22.00, 11.71, 11.68.
^{19}F NMR (376 MHz, $CDCl_3$): *δ* / ppm = -80.85 (t, *J* = 9.2 Hz, 3 F, CF_3^{12}), -120.39 – -120.91 (m, 2 F, CF_2^{13}), -127.07 – -129.04 (m, 2 F, CF_2^{13}). Total integral of CF_2 region normalized with respect to the CF_3^{12} group = 4.
ESI-MS [*m/z*]: $[M + Na]^+$ calculated for $^{12}C_{27}{}^{1}H_{42}{}^{16}O_7{}^{14}N^{19}F_7$, 648.2742; found, 648.2729, Δ = 1.3 mmu.

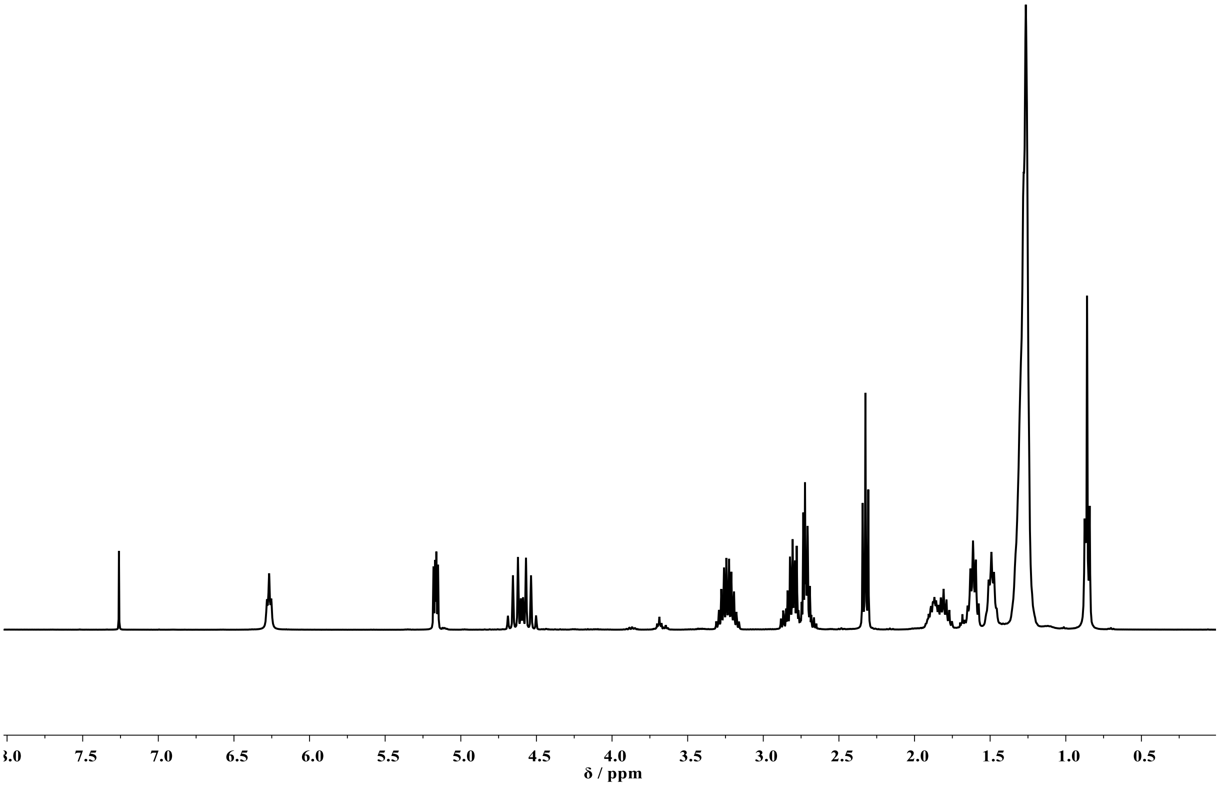

Supplementary Figure 118: ^{1}H-NMR of compound 79 measured in $CDCl_3$.

Passerini reaction

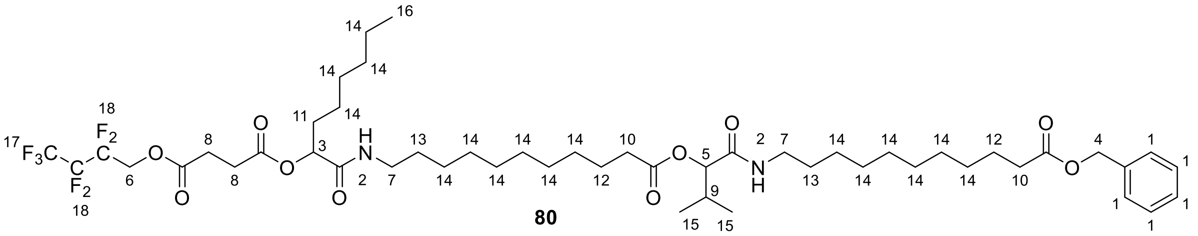

80

In a 50 mL round bottom flask, 794 mg of **79** (1.27 mmol, 1.00 eq.) was stirred in 4.00 mL DCM. Subsequently, 174 µL isobutyraldehyde **14c** (137 mg, 1.90 mmol, 1.50 eq.) and 577 mg of the monomer **M1** (1.90 mmol, 1.50 eq.) were added. The resulting reaction mixture was stirred at room temperature for 2 days. Afterwards, the crude mixture was dried under reduced pressure. The residue was adsorbed onto celite® and purified *via* column chromatography on silica gel eluting with a gradual solvent mixture of ethyl acetate and cyclohexane (5:1 → 2:1) to yield the passerini product **80** as a pale highly viscous oil. (1.12 g, 1.12 mol, 88.2%).

R_f = 0.21 in cyclohexane / ethyl acetate (2:1).

IR (ATR): ν / cm^{-1} = 3308.0 (vw), 2926.1 (m), 2854.9 (w), 1738.8 (s), 1655.3 (s), 1534.8 (m), 1457.1 (w), 1352.5 (w), 1226.6 (vs), 1144.7 (vs), 1019.3 (m), 909.9 (w), 735.7 (w), 697.3 (w).

^{1}H NMR (400 MHz, $CDCl_3$): δ / ppm = 7.36 – 7.21 (m, 5 H, CH_{Ar}^{1}), 6.18 (t, J = 5.8 Hz, 1 H, NH^{2}), 5.88 (t, J = 5.9 Hz, 1 H, NH^{2}), 5.13 – 5.07 (m, 1 H, CH^{3}), 5.04 (s, 2 H, CH_2^{4}), 4.99 (d, J = 4.4 Hz, 1 H, CH^{5}), 4.66 – 4.43 (m, 2 H, CH_2^{6}), 3.30 – 3.09 (m, 4 H, CH^{7}), 2.83 – 2.59 (m, 4 H, CH_2^{8}), 2.39 – 2.19 (m, 5 H, CH^{9}, CH_2^{10}), 1.90 – 1.69 (m, 2 H, CH_2^{11}), 1.67 – 1.58 (m, 4 H, CH_2^{12}), 1.49 – 1.35 (m, 4 H, CH_2^{13}), 1.29 – 1.10 (m, 32 H, CH_2^{14}), 0.92 – 0.84 (m, 6 H, CH_3^{15}), 0.83 – 0.77 (m, 3 H, CH_3^{16}).

^{13}C NMR (101 MHz, $CDCl_3$): δ / ppm = 173.82, 172.68, 171.49, 170.89, 169.63, 169.40, 136.26, 128.67, 128.28, 78.05, 74.88, 66.20, 59.68 (t, J = 26.8 Hz), 39.43, 39.29, 34.45, 34.42, 31.94, 31.72, 30.65, 29.71, 29.58, 29.55, 29.54, 29.49, 29.46, 29.33, 29.25, 29.23, 29.04, 29.01, 28.79, 26.96, 26.95, 25.13, 25.07, 24.93, 22.65, 18.90, 17.07, 14.14.

^{19}F NMR (376 MHz, $CDCl_3$): δ / ppm = -85.16 (t, J = 9.3 Hz, 3 F, CF_3^{17}), -124.27 – -125.33 (m, 2 F, CF_2^{18}), -131.44 – -132.73 (m, 2 F, CF_2^{18}). Total integral of CF_2 region normalized with respect to the CF_3^{17} group = 4.

ESI-MS [*m/z*]: [M + Na]$^+$ calculated for $^{12}C_{50}{}^{1}H_{77}{}^{16}O_{10}{}^{14}N_2{}^{19}F_7$, 1021.5359; found, 1021.5337, Δ = 2.2 mmu.

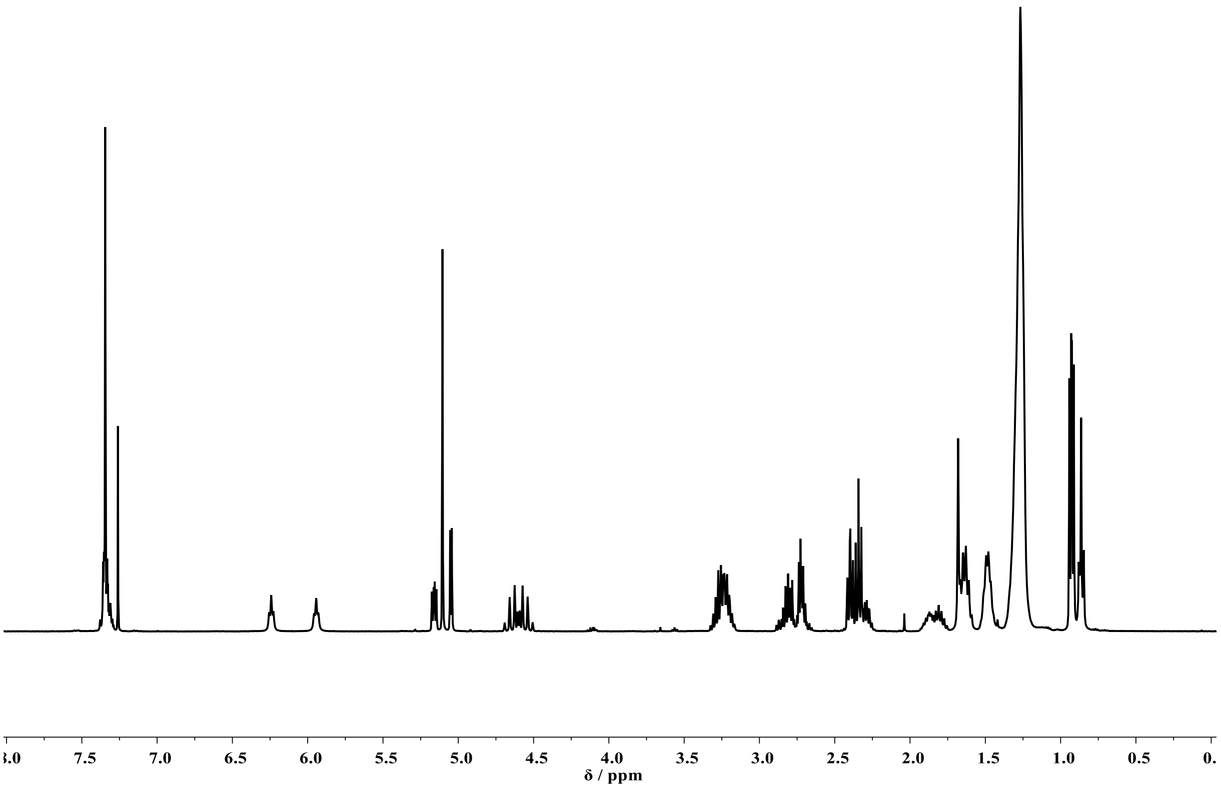

Supplementary Figure 119: ^{1}H-NMR of compound 80 measured in $CDCl_3$.

Deprotection

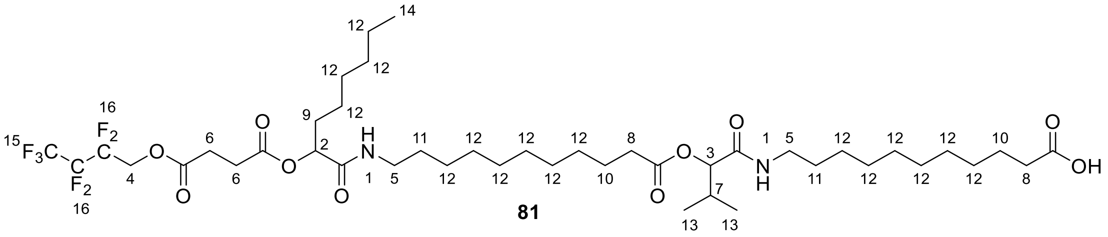

In a 50 mL round bottom flask, 1.03 g of **80** (1.03 mmol, 1.00 eq.) were dissolved in 5.00 mL ethyl acetate and 5.00mL THF. Afterwards, 206 mg (20 wt%) palladium on activated charcoal **16** were added. Subsequently, the mixture was purged with hydrogen (3 balloons) and stirred under hydrogen atmosphere overnight. The heterogeneous catalyst was filtered off and the solvent was evaporated under reduced pressure. The product **81** was obtained as a pale highly viscous oil in a yield of 98.1% (918 mg, 1.01 mmol).

IR (ATR): ν / cm^{-1} = 3305.8 (vw), 2926.4 (s), 2855.3 (m), 1741.5 (s), 1652.3 (s), 1540.2 (m), 1463.6 (w), 1353.0 (w), 1226.3 (vs), 1144.1 (vs), 1019.3 (m), 909.7 (w), 735.6 (w), 627.5 (vw).

^{1}H NMR (400 MHz, $CDCl_3$): δ / ppm = 6.22 (t, J = 5.8 Hz, 1 H, NH^1), 5.97 – 5.86 (m, 1 H, NH^2), 5.13 – 5.06 (m, 1 H, CH^2), 5.01 – 4.96 (m, 1 H, CH^3), 4.64 – 4.43 (m, 2 H, $CH_2{}^4$), 3.30 – 3.09 (m, 4 H,

CH_2^{5}), 2.85 – 2.59 (m, 4 H, CH_2^{6}), 2.37 – 2.17 (m, 5 H, CH^{7}, CH_2^{8}), 1.90 – 1.68 (m, 2 H, CH_2^{9}), 1.64 – 1.50 (m, 4 H, CH_2^{10}), 1.48 – 1.35 (m, 4 H, CH_2^{11}), 1.33 – 1.07 (m, 32 H, CH_2^{12}), 0.89 – 0.84 (m, 6 H, CH_3^{13}), 0.83 – 0.75 (m, 3 H, CH_3^{14}).

^{13}C NMR (101 MHz, $CDCl_3$): *δ* / ppm = 172.71, 171.53, 170.94, 169.83, 169.51, 78.08, 74.87, 59.70 (t, *J* = 26.9 Hz), 39.51, 39.28, 34.43, 34.00, 33.96, 31.91, 31.72, 30.62, 29.60, 29.52, 29.51, 29.43, 29.42, 29.36, 29.35, 29.28, 29.21, 29.20, 29.09, 29.05, 29.00, 28.80, 26.95, 26.90, 25.16, 24.93, 24.85, 22.66, 18.90, 17.07, 14.14.

^{19}F NMR (376 MHz, $CDCl_3$): *δ* / ppm = -84.58 – -86.46 (m, 3 F, CF_3^{15}), -124.16 – -125.27 (m, 2 F, CF_2^{16}), -131.44 – -132.90 (m, 2 F, CF_2^{16}). Total integral of CF_2 region normalized with respect to the CF_3^{15} group = 4.

ESI-MS [*m/z*]: [M + Na]$^+$ calculated for $^{12}C_{43}{}^{1}H_{71}{}^{16}O_{10}{}^{14}N_2{}^{19}F_7$, 931.4889; found, 931.4871, Δ = 1.8 mmu.

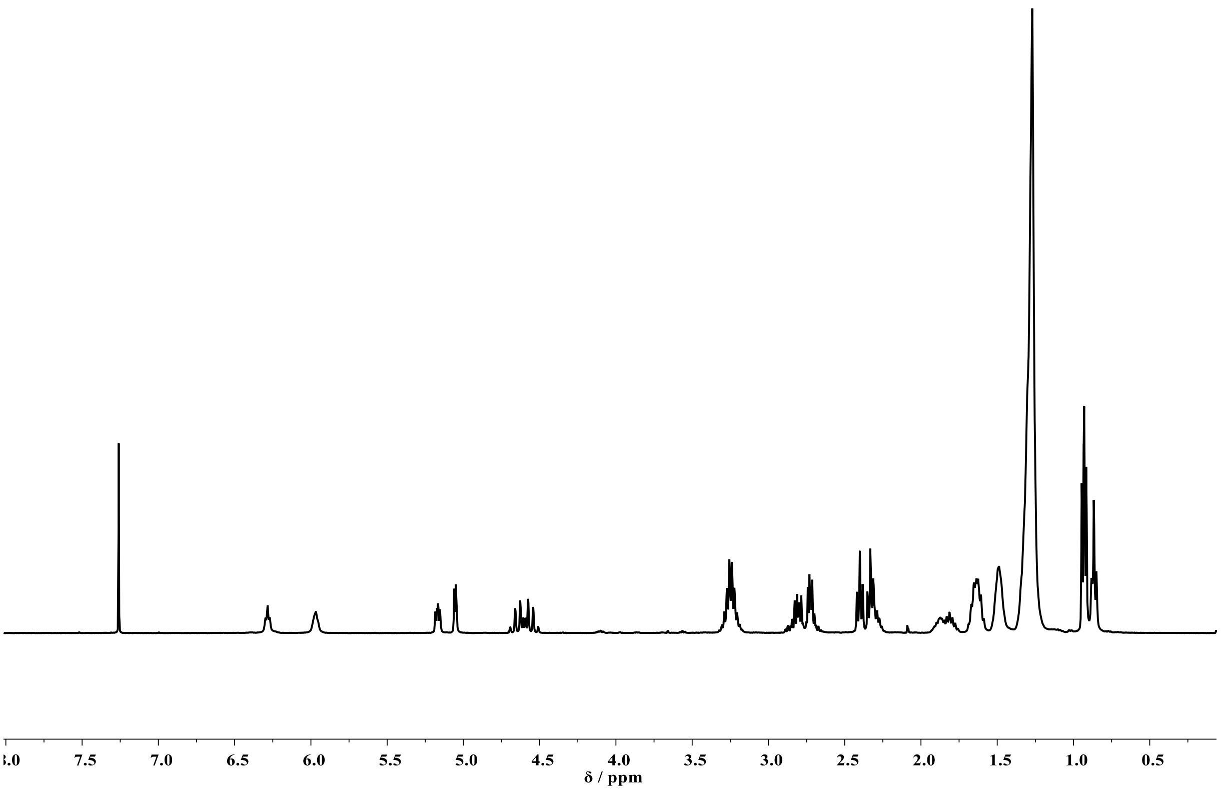

Supplementary Figure 120: ^{1}H-NMR of compound 81 measured in $CDCl_3$.

Passerini reaction

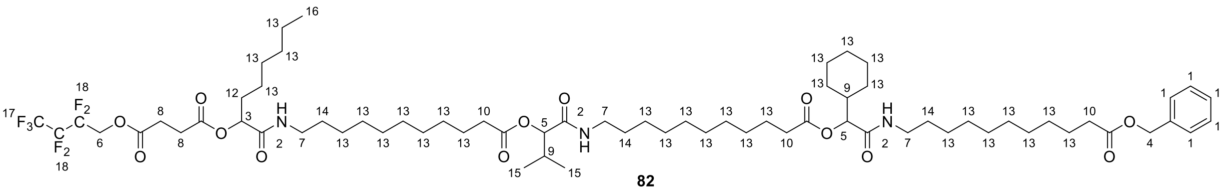

82

In a 50 mL round bottom flask, 831 mg **81** (895 µmol, 1.00 eq.) was stirred in 4.00 mL DCM. Subsequently, 163 µL cyclohexanecarboxaldehyde **14j** (151 mg, 1.34 mmol, 1.50 eq.) and 407 mg of the monomer **M1** (1.34 mmol, 1.50 eq.) were added. The resulting reaction mixture was stirred at room temperature for 2 days. Afterwards, the crude mixture was dried under reduced pressure. The residue was adsorbed onto celite® and purified *via* column chromatography on silica gel eluting with a gradual solvent mixture of ethyl acetate and cyclohexane (4:1 → 2:1) to yield the passerini product **82** as a pale highly viscous oil. (880 mg, 665 µmol, 74.3%).

R_f = 0.13 in cyclohexane / ethyl acetate (2:1).

IR (ATR): ν / cm^{-1} = 3306.2 (vw), 2925.2 (s), 2853.8 (m), 1738.9 (s), 1654.4 (s), 1534.8 (m), 1454.6 (w), 1352.8 (w), 1226.9 (s), 1145.7 (vs), 1019.3 (m), 735.6 (w), 697.4 (w).

^{1}H NMR (400 MHz, $CDCl_3$): δ / ppm = 7.43 – 7.28 (m, 5 H, CH_{Ar}^1), 6.25 (t, J = 5.8 Hz, 1 H, NH^2), 6.02 – 5.90 (m, 2 H, NH^2), 5.19 – 5.14 (m, 1 H, CH^3), 5.11 (s, 2 H, CH_2^4), 5.08 – 5.00 (m, 2 H, CH_2^5), 4.72 – 4.50 (m, 2 H, CH_2^6), 3.34 – 3.16 (m, 6 H, CH_2^7), 2.90 – 2.65 (m, 4 H, CH_2^8), 2.43 – 2.24 (m, 7 H, CH^9, CH_2^{10}), 2.01 – 1.77 (m, 3 H, CH^{11}, CH_2^{12}), 1.76 – 1.58 (m, 12 H, CH_2^{13}), 1.56 – 1.41 (m, 6 H, CH^{14}), 1.37 – 1.04 (m, 48 H, CH_2^{13}), 0.96 – 0.90 (m, 6 H, CH_3^{15}), 0.90 – 0.82 (m, 3 H, CH_3^{16}).

^{13}C NMR (101 MHz, $CDCl_3$): δ / ppm = 173.83, 172.70, 172.69, 171.51, 170.91, 169.65, 169.43, 169.35, 136.26, 128.68, 128.29, 78.06, 77.76, 74.88, 66.21, 59.69, 40.12, 39.44, 39.29, 34.46, 34.43, 31.94, 31.73, 30.65, 29.72, 29.72, 29.58, 29.55, 29.50, 29.48, 29.35, 29.31, 29.26, 29.24, 29.05, 29.02, 28.80, 27.43, 26.98, 26.97, 26.21, 26.13, 26.02, 25.13, 25.08, 24.94, 22.66, 18.91, 17.08, 14.15.

^{19}F NMR (376 MHz, $CDCl_3$): δ / ppm = -85.15 (t, J = 9.2 Hz, 3 F, CF_3^{17}), -124.51 – -125.62 (m, 2 F, CF_2^{18}), -131.79 – -133.84 (m, 2 F, CF_2^{18}). Total integral of CF_2 region normalized with respect to the CF_3^{17} group = 4.

ESI-MS [*m/z*]: $[M + H]^+$ calculated for $^{12}C_{69}{}^{1}H_{110}{}^{16}O_{13}{}^{14}N_3{}^{19}F_7$, 1322.8000; found, 1322.7981, Δ = 1.9 mmu.

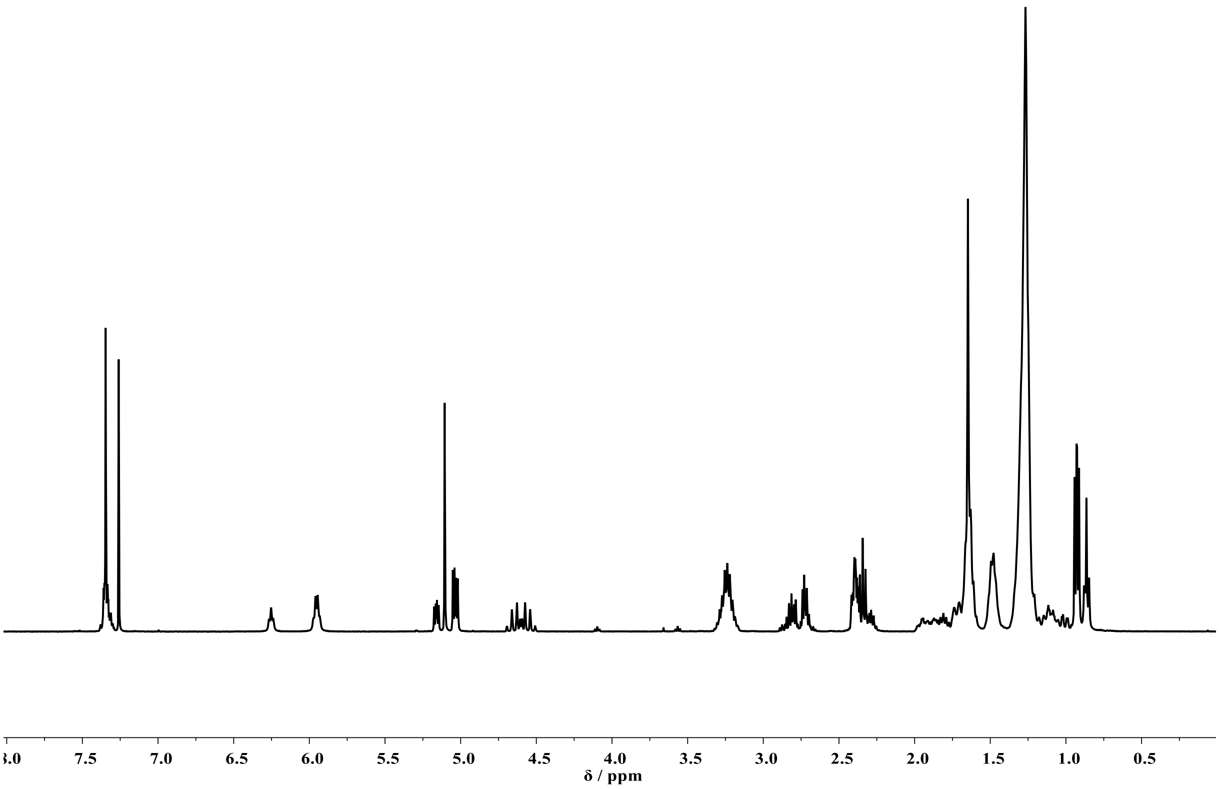

Supplementary Figure 121: ^{1}H-NMR of compound 82 measured in $CDCl_3$.

Deprotection

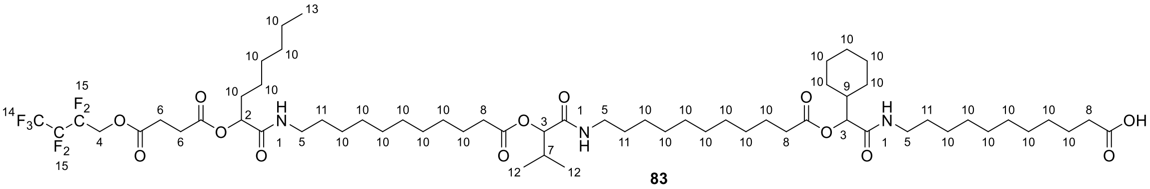

83

In a 50 mL round bottom flask, 829 mg of **82** (627 µmol, 1.00 eq.) were dissolved in 5.00 mL ethyl acetate and 5.00 mL THF. Afterwards, 166 mg (20 wt%) palladium on activated charcoal **16** were added. Subsequently, the mixture was purged with hydrogen (3 balloons) and stirred under hydrogen atmosphere overnight. The heterogeneous catalyst was filtered off and the solvent was evaporated under reduced pressure. The product **83** was obtained as a pale highly viscous oil in a quant. yield. (772 mg, 627 mmol).

IR (ATR): ν / cm^{-1} = 3294.0 (vw), 2925.3 (s), 2854.1 (m), 1740.3 (s), 1651.1 (s), 1538.6 (m), 1452.6 (w), 1370.2 (w), 1226.3 (vs), 1145.1 (vs), 1019.1 (w), 909.9 (w), 735.4 (w).

^{1}H NMR (400 MHz, $CDCl_3$): δ / ppm = 6.24 (t, J = 5.8 Hz, 1 H, NH^1), 6.06 – 5.88 (m, 2 H, NH^1), 5.14 – 5.07 (m, 1 H, CH^2), 5.03 – 4.92 (m, 2 H, CH^3), 4.70 – 4.39 (m, 2 H, $CH_2{}^4$), 3.26 – 3.08 (m, 6 H, $CH_2{}^5$), 2.84 – 2.58 (m, 4 H, $CH_2{}^6$), 2.39 – 2.16 (m, 7 H, CH^7, $CH_2{}^8$), 1.94 – 1.50 (m, 15 H, CH^9, $CH_2{}^{10}$),

1.48 – 1.33 (m, 6 H, CH_2^{11}), 1.33 – 0.94 (m, 48 H, CH_2^{10}), 0.91 – 0.83 (m, 6 H, CH_3^{12}), 0.83 – 0.77 (m, 3 H, CH_3^{13}).

^{13}C NMR (101 MHz, $CDCl_3$): δ / ppm = 172.77, 172.71, 171.52, 170.92, 169.77, 169.59, 169.45, 78.03, 77.74, 74.82, 59.65 (t, J = 27.0 Hz), 40.05, 39.45, 39.32, 39.25, 34.40, 34.08, 31.91, 31.70, 30.61, 29.67, 29.56, 29.49, 29.46, 29.34, 29.31, 29.29, 29.24, 29.21, 29.11, 29.02, 28.99, 28.77, 27.39, 26.94, 26.92, 26.90, 26.17, 26.10, 25.98, 25.12, 25.11, 24.90, 22.64, 18.87, 17.06, 14.13.

ESI-MS [*m/z*]: $[M + H]^+$ calculated for $^{12}C_{62}{}^{1}H_{104}{}^{16}O_{13}{}^{14}N_{3}{}^{19}F_{7}$, 1232.7530; found, 1232.7502, Δ = 2.8 mmu.

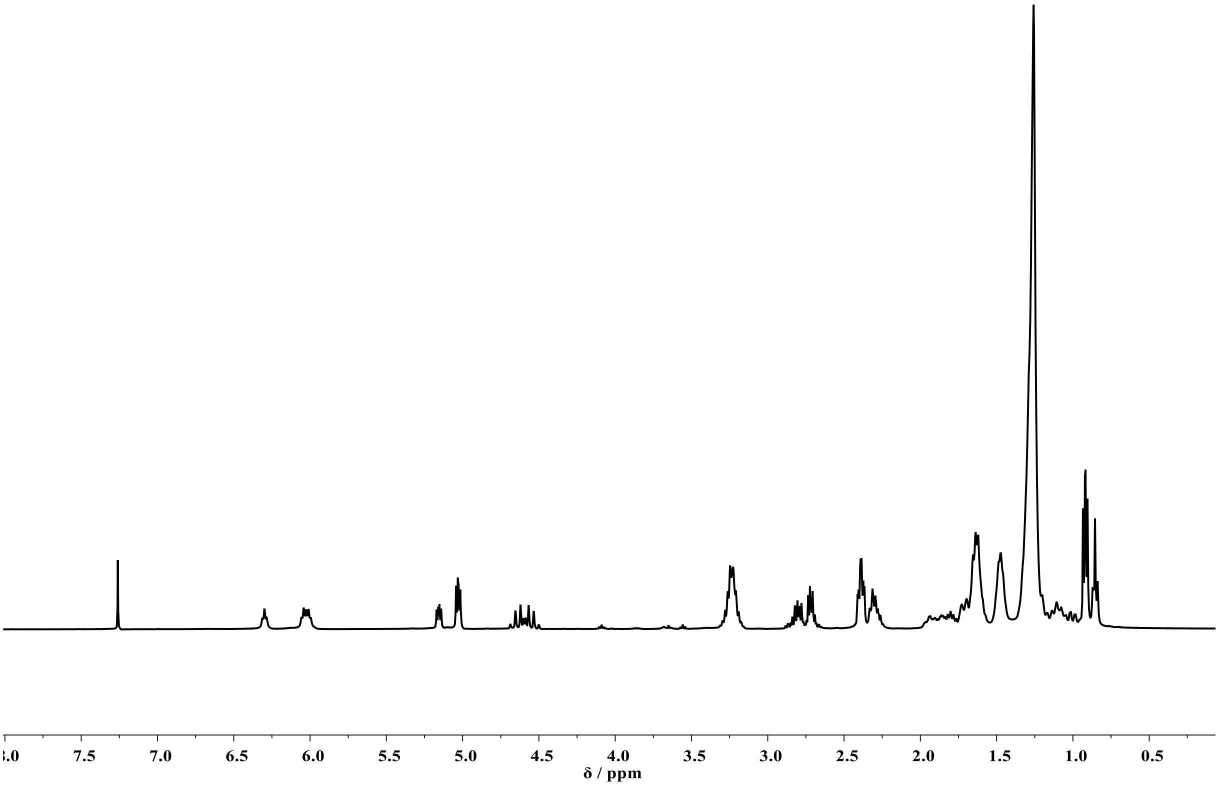

Supplementary Figure 122: ^{1}H-NMR of compound 83 measured in $CDCl_3$.

Passerini reaction

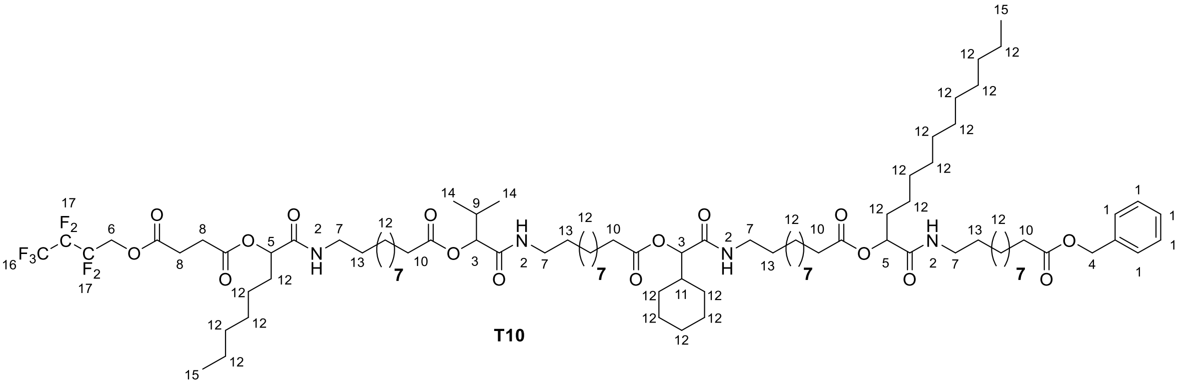

T10

In a 50 mL round bottom flask, 686 mg of **83** (557 µmol, 1.00 eq.) was stirred in 4.00 mL DCM. Subsequently, 154 mg dodecanal (835 µmol, 1.50 eq.) and 252 mg of the monomer **M1** (835 mmol, 1.50 eq.) were added. The resulting reaction mixture was stirred at room temperature for 1 day. Afterwards, the crude mixture was dried under reduced pressure. The residue was adsorbed onto celite® and purified *via* column chromatography on silica gel eluting with a gradual solvent mixture of ethyl acetate and cyclohexane (4:1 → 2:1) to yield the passerini product **T10** as a pale highly viscous oil. (707 mg, 412 µmol, 74.0%).

R_f = 0.66 in cyclohexane / ethyl acetate (1:1).

IR (ATR): ν / cm^{-1} = 3290.4 (vw), 2920.8 (m), 2851.4 (w), 1736.9 (m), 1655.8 (m), 1557.1 (w), 1466.8 (vw), 1377.1 (vw), 1228.5 (w), 1205.6 (w), 1174.5 (m), 1019.8 (vw), 722.6 (vw), 696.6 (vw).

^{1}H NMR (400 MHz, $CDCl_3$): δ / ppm = 7.41 – 7.28 (m, 5 H, CH_{Ar}^{1}), 6.26 (t, *J* = 5.8 Hz, 1 H, NH^{2}), 6.06 – 5.93 (m, 3 H, NH^{2}), 5.19 – 5.13 (m, 2 H, CH^{3}), 5.10 (s, 2 H, CH_2^{4}), 5.07 – 4.99 (m, 2 H, CH^{5}), 4.72 – 4.48 (m, 2 H, CH_2^{6}), 3.34 – 3.14 (m, 8 H, CH_2^{7}), 2.90 – 2.65 (m, 4 H, CH_2^{8}), 2.43 – 2.24 (m, 9 H, CH^{9}, CH_2^{10}), 2.00 – 1.58 (m, 19 H, CH^{11}, CH_2^{12}), 1.54 – 1.43 (m, 8 H, CH_2^{13}), 1.37 – 1.04 (m, 78 H, CH_2^{12}), 0.95 – 0.90 (m, 6 H, CH_3^{14}), 0.89 – 0.83 (m, 6 H, CH_3^{15}).

^{13}C NMR (101 MHz, $CDCl_3$): δ / ppm = 173.82, 172.69, 172.60, 171.50, 170.90, 169.98, 169.63, 169.41, 169.35, 136.24, 128.67, 128.28, 78.03, 77.74, 74.86, 74.06, 66.20, 59.66, 40.10, 39.42, 39.32, 39.27, 34.44, 32.04, 31.93, 31.72, 30.64, 29.75, 29.72, 29.68, 29.67, 29.54, 29.49, 29.49, 29.34, 29.31, 29.26, 29.24, 29.04, 29.01, 28.79, 27.41, 26.96, 26.95, 26.20, 26.12, 26.01, 25.12, 25.07, 24.93, 24.89, 22.81, 22.65, 18.91, 17.07, 14.25, 14.15.

^{19}F NMR (376 MHz, $CDCl_3$): δ / ppm = -83.46 – -86.58 (m, 3 F, CF_3^{16}), -123.51 – -126.45 (m, 2 F, CF_2^{16}), -131.44 – -133.84 (m, 2 F, CF_2^{17}).Total integral of CF_2 region normalized with respect to the CF_3^{16} group = 4.

ESI-MS [*m/z*]: $[M + H]^+$ calculated for $^{12}C_{93}{}^{1}H_{155}{}^{16}O_{16}{}^{14}N_{4}{}^{19}F_{7}$, 1718.1399; found, 1718.1400, Δ = 0.1 mmu.

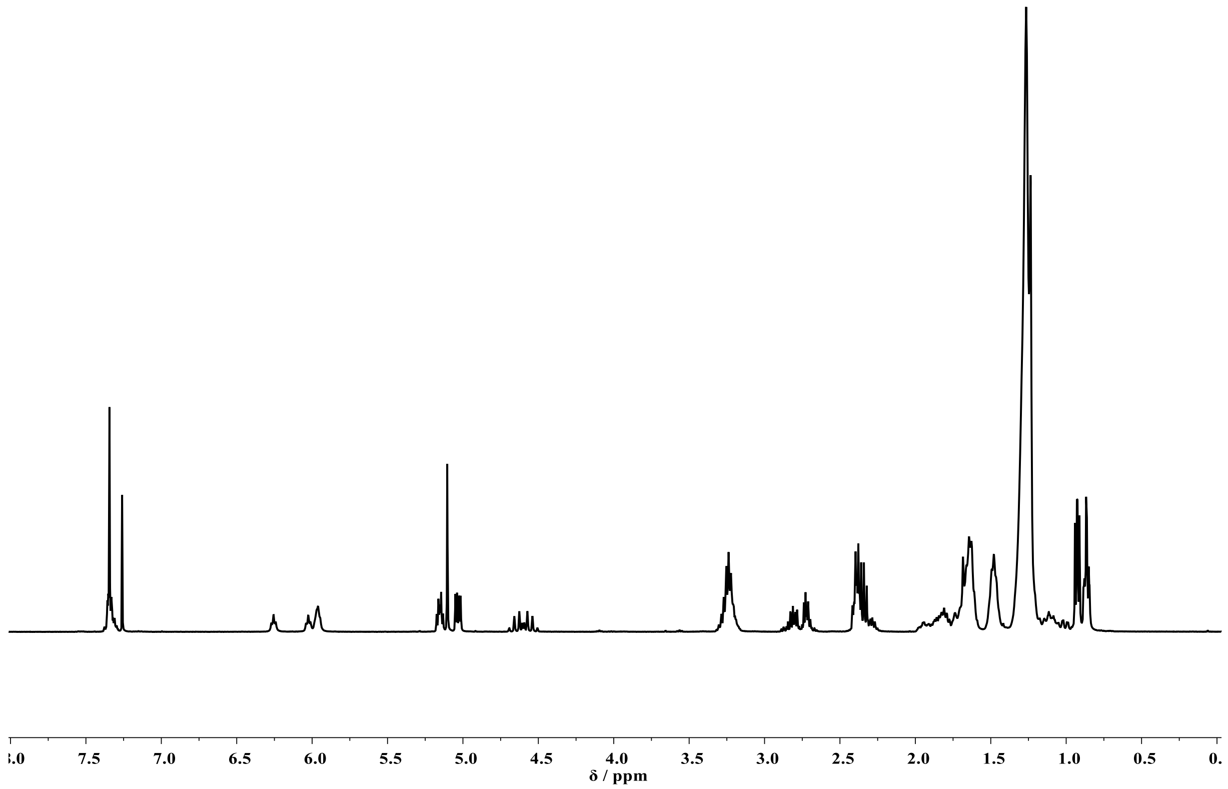

Supplementary Figure 123: ^{1}H-NMR of compound T10 measured in $CDCl_3$.

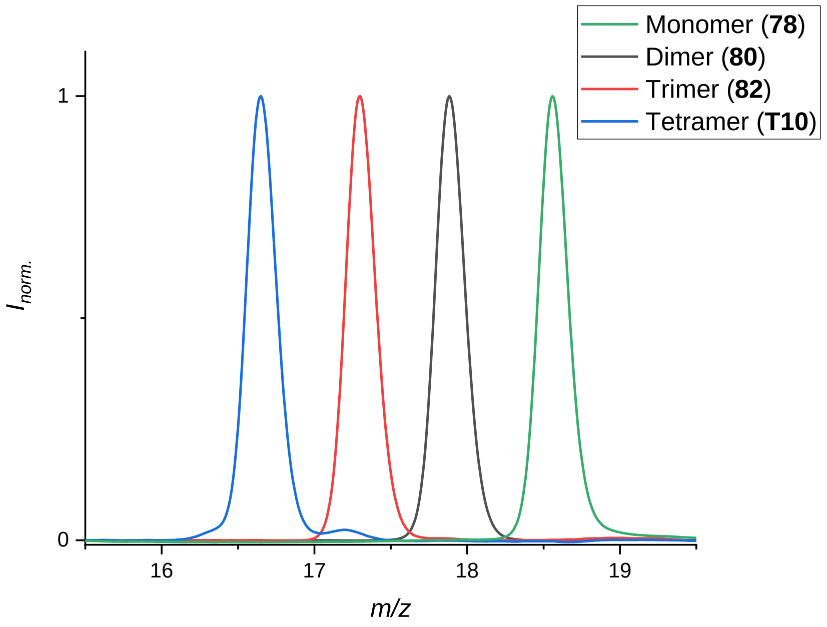

Supplementary Figure 124: SEC traces of the intermediates after each P3CR in the synthesis of product T10.

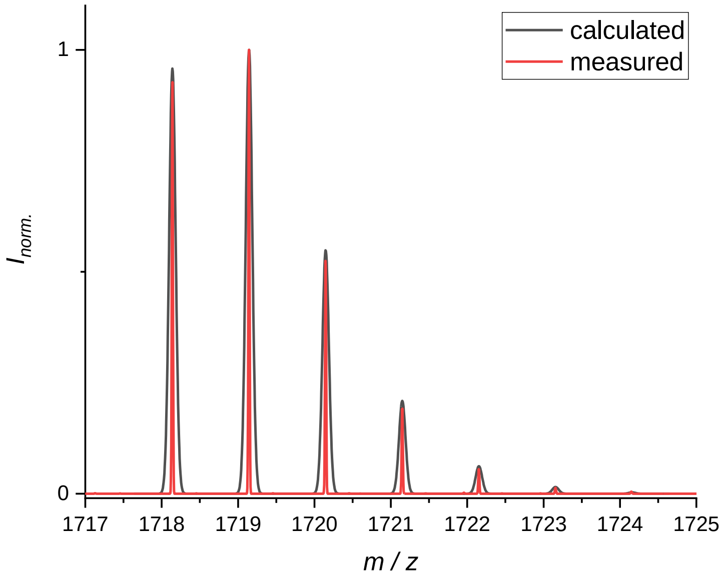

Supplementary Figure 125: High resolution ESI-MS measurement of T10. The observed isotopic pattern is compared with the calculated isotopic pattern obtained from mMass (black).

```
matching mass 1741.12135
cutoff 0.50000: 0 solutions (14 peaks)
cutoff 0.25000: 0 solutions (55 peaks)
cutoff 0.12500: 0 solutions (113 peaks)
cutoff 0.06250: 0 solutions (186 peaks)
cutoff 0.03125: 2 solutions (280 peaks)
cutoff 0.04688: 0 solutions (222 peaks)
cutoff 0.03906: 0 solutions (250 peaks)
cutoff 0.03516: 0 solutions (262 peaks)
cutoff 0.03320: 1 solutions (275 peaks)
1741.12135 ≈ 299.015430 + 325.261700 + 283.214750 + 323.246050 + 395.339950 + 91.054780 (sides Heptanal,
 Isobutyraldehyde, Cyclohexancarboxaldehyde, Dodecanal; error -23.98869)
Press ENTER to quit ...
```

Supplementary Figure 126: Screenshot of the automated read-out of T10, sodium trifluoroacetate was used as additive during the measurement.

1.3.5.4 Synthesis of tetramer T11

Passerini reaction

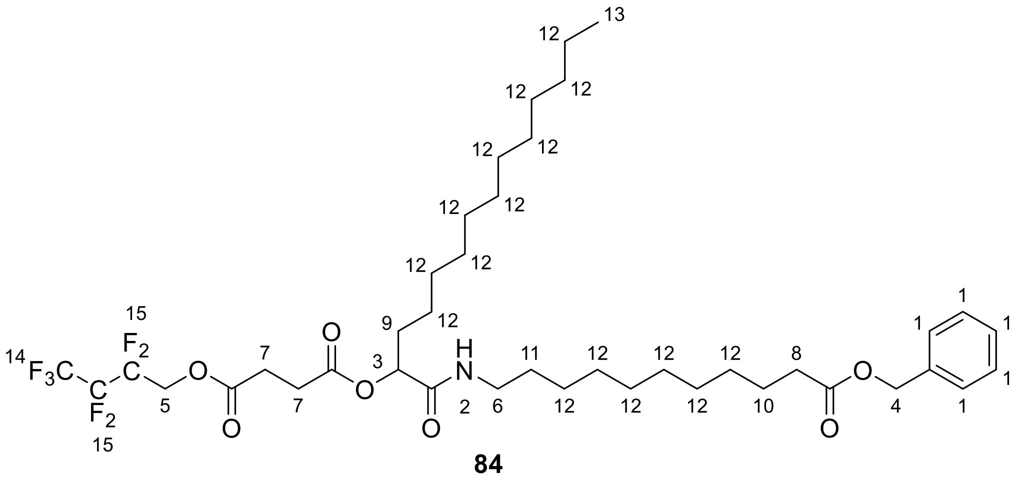

84

In a 50 mL round bottom flask, 500 mg **TAG2** (1.67 mmol, 1.00 eq.) was stirred in 2.00 mL DCM. Subsequently, 593 µL tridecanal **14d** (496 mg, 2.50 mmol, 1.50 eq.) and 754 mg of the monomer **M1** (2.50 mmol, 1.50 eq.) were added. The resulting reaction mixture was stirred at room temperature for 2 days. Afterwards, the crude mixture was dried under reduced pressure. The residue was adsorbed onto celite® and purified *via* column chromatography on silica gel eluting with a gradual solvent mixture of ethyl acetate and cyclohexane (7:1 → 5:1) to yield the passerini product **84** as a pale highly viscous oil. (1.03 g, 1.30 mmol, 78.0%).

R_f = 0.45 in cyclohexane / ethyl acetate (3:1).

IR (ATR): ν / cm^{-1} = 3305.5 (vw), 2924.2 (s), 2853.9 (m), 1739.8 (s), 1656.6 (m), 1535.6 (w), 1456.5 (w), 1352.2 (w), 1226.6 (vs), 1144.2 (vs), 1019.9 (m), 909.7 (w), 735.7 (m), 696.9 (m).

^{1}H NMR (400 MHz, $CDCl_3$): δ / ppm = 7.44 – 7.30 (m, 5 H, $CH_{Ar}{}^{1}$), 6.22 (t, J = 5.8 Hz, 1 H, NH^{2}), 5.19 – 5.13 (m, 1 H, CH^{3}), 5.11 (s, 2 H, $CH_2{}^{4}$), 4.73 – 4.49 (m, 2 H, $CH_2{}^{5}$), 3.34 – 3.15 (m, 2 H, $CH_2{}^{6}$), 2.91 – 2.66 (m, 4 H, $CH_2{}^{7}$), 2.34 (t, J = 7.6 Hz, 2 H, $CH_2{}^{8}$), 1.95 – 1.74 (m, 2 H, $CH_2{}^{9}$), 1.68 – 1.57 (m, 2 H, $CH_2{}^{10}$), 1.54 – 1.44 (m, 2 H, $CH_2{}^{11}$), 1.36 – 1.15 (m, 32 H, $CH_2{}^{12}$), 0.92 – 0.83 (m, 3 H, $CH_3{}^{13}$).

^{13}C NMR (101 MHz, $CDCl_3$): δ / ppm = 173.82, 171.48, 170.89, 169.62, 136.27, 128.67, 128.29, 74.90, 66.19, 59.69 (t, J = 27.1 Hz), 39.45, 34.45, 32.05, 31.95, 29.78, 29.77, 29.75, 29.68, 29.58, 29.56, 29.54, 29.48, 29.37, 29.33, 29.24, 29.04, 28.79, 26.96, 25.06, 25.00, 22.82, 14.24.

^{19}F NMR (376 MHz, $CDCl_3$): δ / ppm = -85.16 (t, J = 9.2, 3 F, $CF_3{}^{14}$),), -123.69 – -125.74 (m, 2 F, $CF_2{}^{15}$), -131.26 – -133.08 (m, 2 F, $CF_2{}^{15}$). Total integral of CF_2 region normalized with respect to the $CF_3{}^{14}$ group = 4.

ESI-MS [*m/z*]: $[M + Na]^+$ calculated for $^{12}C_{40}{}^{1}H_{60}{}^{16}O_7{}^{14}N^{19}F_7$, 822.4150; found, 822.4133, Δ = 1.7 mmu.

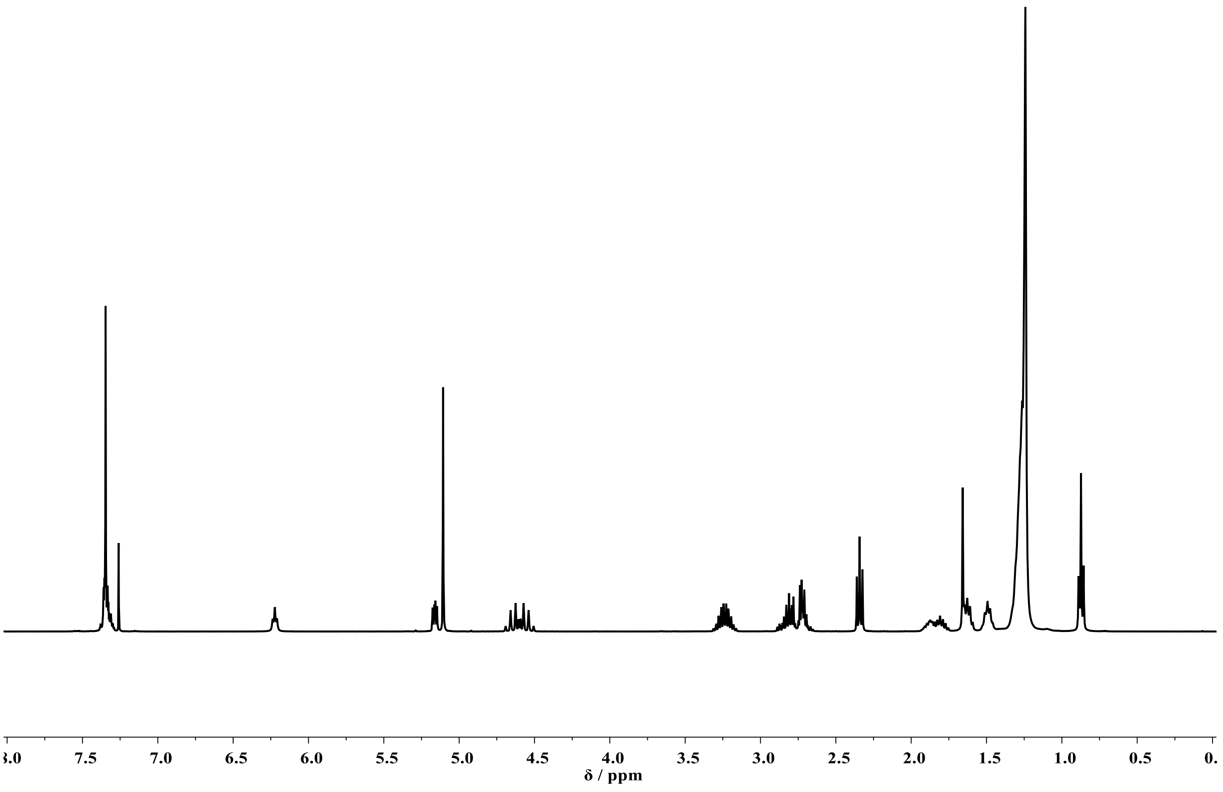

Supplementary Figure 127: ^{1}H-NMR of compound 84 measured in $CDCl_3$.

Deprotection

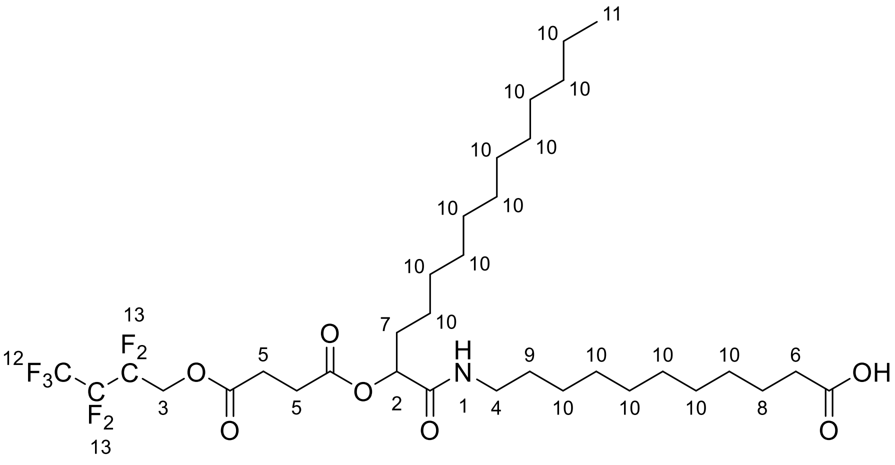

85

In a 50 mL round bottom flask, 1.03 g of **84** (1.28 mmol, 1.00 eq.) were dissolved in 4.00 mL ethyl acetate and 4.00 mL THF. Afterwards, 103 mg (10 wt%) palladium on activated charcoal **16** were added. Subsequently, the mixture was purged with hydrogen (3 balloons) and stirred under hydrogen atmosphere overnight. The heterogeneous catalyst was filtered off and the solvent was evaporated under

reduced pressure. The product **85** was obtained as a pale highly viscous oil in a yield of 98.4% (894 mg, 1.26 mmol).

IR (ATR): ν / cm^{-1} = 3293.4 (vw), 2918.0 (s), 2851.6 (s), 1736.1 (vs), 1694.9 (s), 1657.2 (vs), 1560.9 (w), 1470.0 (w), 1419.7 (w), 1343.8 (m), 1278.1 (m), 1220.9 (vs), 1176.8 (vs), 1131.7 (vs), 1021.9 (m), 929.3 (m), 800.9 (w), 722.1 (w), 678.8 (w), 624.1 (w), 528.8 (w), 462.8 (vw).

^{1}H NMR (400 MHz, $CDCl_3$): δ / ppm = 6.26 (t, J = 5.8 Hz, 1 H, NH^{1}), 5.21 – 5.13 (m, 1 H, CH^{2}), 4.75 – 4.48 (m, 2 H, CH_2^{3}), 3.35 – 3.15 (m, 2 H, CH_2^{4}), 2.91 – 2.64 (m, 4 H, CH_2^{5}), 2.33 (t, J = 7.5 Hz, 2 H, CH_2^{6}), 1.94 – 1.73 (m, 2 H, CH_2^{7}), 1.71 – 1.57 (m, 2 H, CH_2^{8}), 1.54 – 1.42 (m, 2 H, CH_2^{9}), 1.38 – 1.14 (m, 32 H, CH_2^{10}), 0.91 – 0.83 (m, 3 H, CH_3^{11}).

^{13}C NMR (101 MHz, $CDCl_3$): δ / ppm = 179.82, 172.29, 171.70, 170.54, 75.64, 60.46 (t, J = 26.9 Hz), 40.24, 34.86, 32.81, 32.69, 30.54, 30.54, 30.52, 30.45, 30.32, 30.24, 30.13, 30.03, 29.99, 29.86, 29.81, 29.55, 27.66, 25.76, 25.58, 23.58, 15.00.

^{19}F NMR (376 MHz, $CDCl_3$): δ / ppm = -85.17 (t, J = 9.2 Hz, 3 F, CF_3^{12}), -124.45 – -128.85 (m, 2 F, CF_2^{13}), -131.44 – -136.02 (m, 2 F, CF_2^{13}). Total integral of CF_2 region normalized with respect to the CF_3^{12} group = 4.

ESI-MS [*m/z*]: $[M + H]^+$ calculated for $^{12}C_{33}{}^{1}H_{54}{}^{16}O_7{}^{14}N^{19}F_7$, 710.3861; found, 710.3848, Δ = 1.3 mmu.

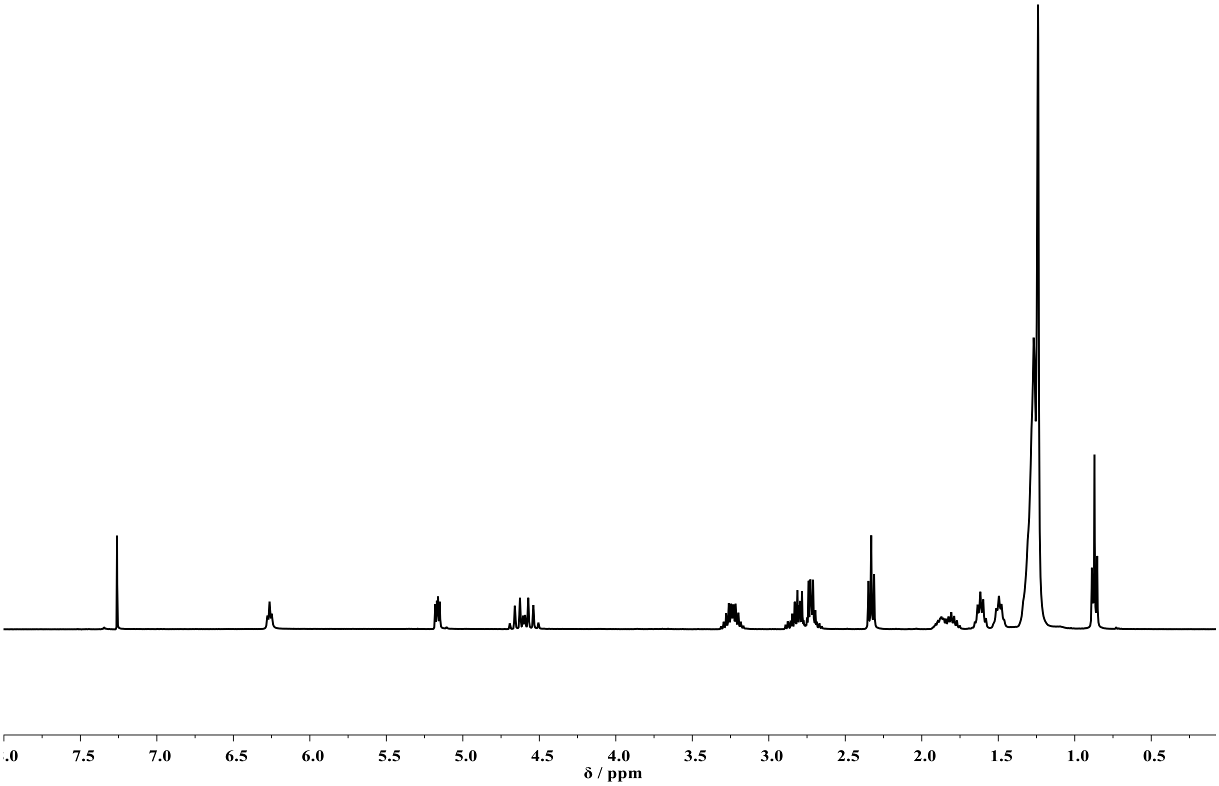

Supplementary Figure 128: ^{1}H-NMR of compound 85 measured in $CDCl_3$.

Passerini reaction

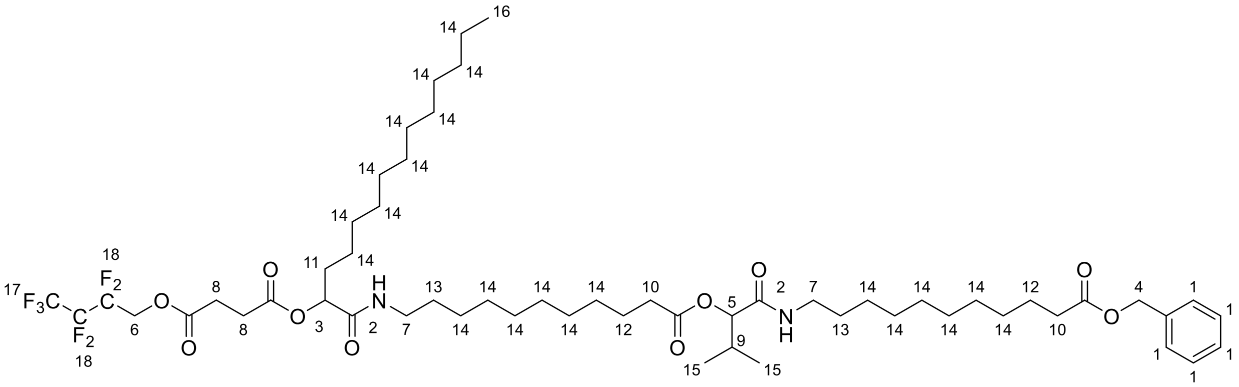

In a 50 mL round bottom flask, 420 mg **85** (592 µmol, 1.00 eq.) was stirred in 3.00 mL DCM. Subsequently, 81.1 µL isobutyraldehyde **14c** (64.0 mg, 888 µmol, 1.50 eq.) and 268 mg of the monomer **M1** (888 µmol, 1.50 eq.) were added. The resulting reaction mixture was stirred at room temperature for 2 days. Afterwards, the crude mixture was dried under reduced pressure. The residue was adsorbed onto celite® and purified *via* column chromatography on silica gel eluting with a gradual solvent mixture of ethyl acetate and cyclohexane (5:1 → 1:1) to yield the passerini product **86** as a pale highly viscous oil. (618 g, 571 µmol, 96.4%).

R_f = 0.36 in cyclohexane / ethyl acetate (2:1).

IR (ATR): ν / cm^{-1} = 3306.9 (w), 2917.5 (s), 2850.1 (s), 1732.5 (vs), 1656.2 (vs), 1544.3 (m), 1467.2 (w), 1312.7 (w), 1228.4 (vs), 1148.4 (vs), 1020.4 (m), 979.6 (m), 913.5 (m), 800.8 (vw), 735.0 (m), 696.5 (m), 536.6 (vw), 474.9 (vw), 422.4 (vw).

^{1}H NMR (400 MHz, $CDCl_3$): δ / ppm = 7.43 – 7.28 (m, 5 H, CH_{Ar}^{1}), 6.24 (t, *J* = 5.8 Hz, 1 H, NH^{2}), 5.95 (t, *J* = 5.9 Hz, 1 H, NH^{2}), 5.19 – 5.13 (m, 1 H, CH^{3}), 5.10 (s, 2 H, CH^{4}), 5.05 (d, *J* = 4.4 Hz, 1 H, CH^{5}), 4.71 – 4.49 (m, 2 H, CH_2^{6}), 3.36 – 3.14 (m, 4 H, CH_2^{7}), 2.89 – 2.65 (m, 4 H, CH_2^{8}), 2.44 – 2.22 (m, 5 H, CH^{9}, CH_2^{10}), 1.94 – 1.73 (m, 2 H, CH_2^{11}), 1.69 – 1.57 (m, 4 H, CH_2^{12}), 1.54 – 1.43 (m, 4 H, CH_2^{13}), 1.36 – 1.20 (m, 44 H, CH^{14}), 0.96 – 0.90 (m, 6 H, CH_3^{15}), 0.89 – 0.83 (m, 3 H, CH_2^{16}).

^{13}C NMR (101 MHz, $CDCl_3$): δ / ppm = 173.81, 172.66, 171.48, 170.89, 169.63, 169.39, 136.27, 128.66, 128.28, 78.05, 74.88, 66.19, 59.67 (t, *J* = 27.0 Hz), 39.43, 39.28, 34.44, 34.42, 32.04, 31.94, 30.65, 29.76, 29.74, 29.71, 29.67, 29.58, 29.55, 29.49, 29.47, 29.36, 29.33, 29.26, 29.23, 29.04, 28.79, 26.96, 26.95, 25.13, 25.06, 25.00, 22.81, 18.90, 17.06, 14.23.

^{19}F NMR (376 MHz, $CDCl_3$): δ / ppm = -85.16 (t, *J* = 9.2 Hz, 3 F, CF_3^{17}), -124.27 – -125.74 (m, 2 F, CF_2^{18}), -131.26 – -133.08 (m, 2 F, CF_2^{18}). Total integral of CF_2 region normalized with respect to the CF_3^{17} group = 4.

ESI-MS [*m/z*]: $[M + H]^+$ calculated for $^{12}C_{56}{}^{1}H_{89}{}^{16}O_{10}{}^{14}N_2{}^{19}F_7$, 1083.6478; found, 1083.6459, Δ = 1.9 mmu.

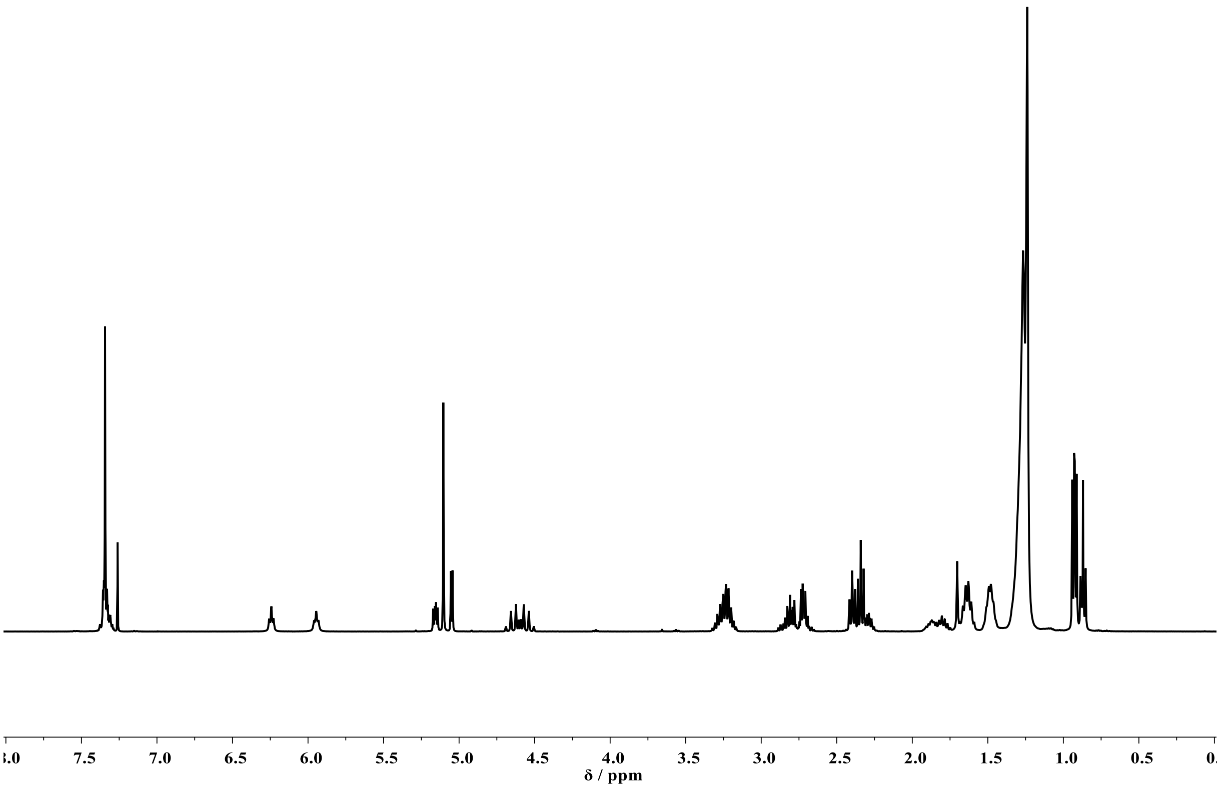

Supplementary Figure 129: ¹H-NMR of compound 86 measured in $CDCl_3$.

Deprotection

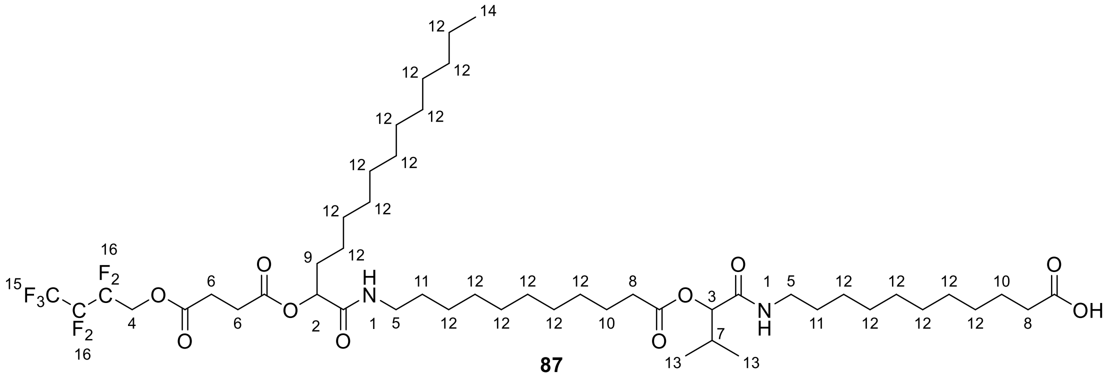

87

In a 50 mL round bottom flask, 537 mg of **86** (496 µmol, 1.00 eq.) were dissolved in 4.00 mL ethyl acetate and 4.00 mL THF. Afterwards, 107 mg (20 wt%) palladium on activated charcoal **16** were added. Subsequently, the mixture was purged with hydrogen (3 balloons) and stirred under hydrogen atmosphere overnight. The heterogeneous catalyst was filtered off and the solvent was evaporated under reduced pressure. The product **87** was obtained as a pale highly viscous oil in a yield of 98.2% (484 mg, 487 µmol).

IR (ATR): ν / cm^{-1} = 3324.9 (vw), 2920.4 (s), 2850.9 (s), 1741.3 (vs), 1702.3 (m), 1650.7 (vs), 1540.1 (m), 1466.9 (w), 1433.6 (w), 1354.3 (w), 1291.7 (m), 1228.4 (vs), 1145.5 (vs), 1020.8 (m), 955.5 (w), 721.0 (m), 658.5 (w), 534.0 (vw), 473.2 (vw), 403.9 (vw).

^{1}H NMR (400 MHz, $CDCl_3$): δ / ppm = 6.31 (t, J = 5.8 Hz, 1 H, NH^1), 6.00 (t, J = 5.8 Hz, 1 H, NH^1), 5.20 – 5.13 (m, 1 H, CH^2), 5.05 (d, J = 4.4 Hz, 1 H, CH^3), 4.72 – 4.49 (m, 2 H, $CH_2{}^4$), 3.36 – 3.14 (m, 4 H, $CH_2{}^5$), 2.92 – 2.64 (m, 4 H, $CH_2{}^6$), 2.45 – 2.24 (m, 5 H, CH^7, $CH_2{}^8$), 1.95 – 1.71 (m, 2 H, $CH_2{}^9$), 1.68 – 1.56 (m, 4 H, $CH_2{}^{10}$), 1.54 – 1.40 (m, 4 H, $CH_2{}^{11}$), 1.40 – 1.14 (m, 44 H, $CH_2{}^{12}$), 0.95 – 0.89 (m, 6 H, $CH_3{}^{13}$), 0.89 – 0.84 (m, 3 H, $CH_3{}^{14}$).

^{13}C NMR (101 MHz, $CDCl_3$): δ / ppm = 178.15, 172.71, 171.54, 170.96, 169.84, 169.53, 78.05, 74.85, 59.68 (t, J = 26.7 Hz), 39.49, 39.28, 34.41, 34.08, 32.04, 31.91, 30.61, 29.78, 29.77, 29.74, 29.67, 29.59, 29.55, 29.50, 29.47, 29.44, 29.34, 29.33, 29.27, 29.22, 29.10, 29.03, 28.78, 26.94, 26.90, 25.14, 24.99, 24.87, 22.81, 18.89, 17.05, 14.23.

^{19}F NMR (376 MHz, $CDCl_3$): δ / ppm = -85.16 (t, J = 9.2 Hz, 3 F, $CF_3{}^{15}$), -123.16 – -126.45 (m, 2 F, $CF_2{}^{16}$), -130.67 – -138.42 (m, 2 F, $CF_2{}^{16}$). Total integral of CF_2 region normalized with respect to the $CF_3{}^{15}$ group = 4.

ESI-MS [*m/z*]: $[M + H]^+$ calculated for $^{12}C_{49}{}^{1}H_{83}{}^{16}O_{10}{}^{14}N_2{}^{19}F_7{}^{23}Na$, 1015.5828; found, 1015.5812, Δ = 1.6 mmu.

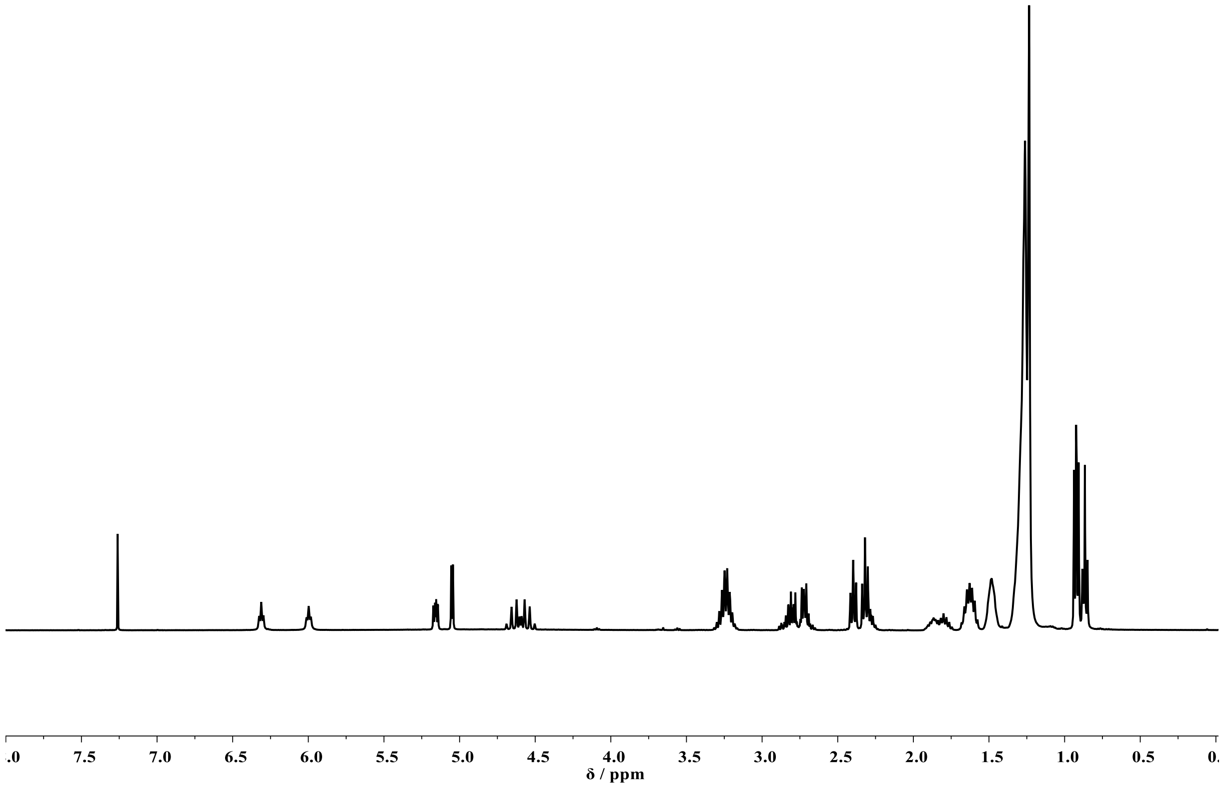

Supplementary Figure 130: ^{1}H-NMR of compound 87 measured in $CDCl_3$.

Passerini reaction

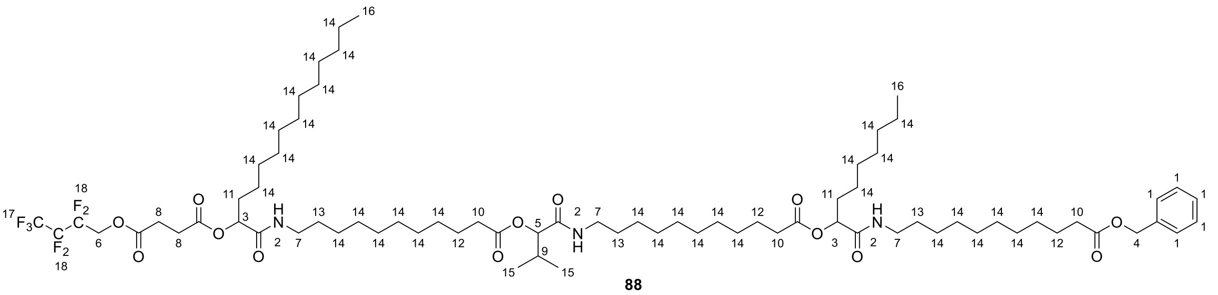

88

In a 50 mL round bottom flask, 403 mg of **87** (406 µmol, 1.00 eq.) was stirred in 4.00 mL dichloromethane. Subsequently, 95.2 µL octanal **14i** (78.1 mg, 609 µmol, 1.50 eq.) and 184 mg of the monomer **M1** (609 µmol, 1.50 eq.) were added. The resulting reaction mixture was stirred at room temperature for 2 days. Afterwards, the crude mixture was dried under reduced pressure. The residue was adsorbed onto celite® and purified *via* column chromatography on silica gel eluting with a gradual solvent mixture of ethyl acetate and cyclohexane (6:1 → 4:1) to yield the passerini product **88** as a pale highly viscous oil. (430 mg, 302 µmol, 74.4%).

R_f = 0.53 in cyclohexane / ethyl acetate (2:1).

IR (ATR): ν / cm^{-1} = 3300.5 (vw), 2919.6 (w), 2850.9 (w), 2367.7 (vw), 2358.7 (vw), 2339.6 (vw), 2123.3 (vw), 1739.2 (w), 1655.9 (w), 1556.2 (vw), 1466.6 (vw), 1366.9 (vw), 1301.3 (vw), 1225.7 (w), 1164.1 (w), 1118.0 (w), 1019.5 (vw), 912.3 (vw), 803.3 (vw), 738.2 (vw), 696.7 (vw), 417.8 (vw).

^{1}H NMR (500 MHz, CDCl$_3$): δ / ppm = 7.35 – 7.21 (m, 5 H, $CH_{Ar}{}^{1}$), 6.16 (t, J = 5.8 Hz, 1 H, NH^{2}), 5.94 (t, J = 5.9 Hz, 1 H, NH^{2}), 5.88 (t, J = 5.9 Hz, 1 H, NH^{2}), 5.12 – 5.07 (m, 2 H, CH^{3}), 5.04 (s, 2 H, $CH_2{}^{4}$), 4.98 (d, J = 4.4 Hz, 1 H, CH^{5}), 4.68 – 4.43 (m, 2 H, $CH_2{}^{6}$), 3.32 – 3.10 (m, 6 H, $CH_2{}^{7}$), 2.85 – 2.60 (m, 4 H, $CH_2{}^{8}$), 2.37 – 2.18 (m, 7 H, CH^{9}, $CH_2{}^{10}$), 1.91 – 1.67 (m, 4 H, $CH_2{}^{11}$), 1.66 – 1.54 (m, 6 H, $CH_2{}^{12}$), 1.48 – 1.37 (m, 6 H, $CH_2{}^{13}$), 1.32 – 1.11 (m, 66 H, CH^{14}), 0.87 (t, J = 6.5 Hz, 6 H, $CH_3{}^{15}$), 0.83 – 0.74 (m, 6 H, $CH_3{}^{16}$).

^{13}C NMR (126 MHz, CDCl$_3$): δ / ppm = 173.83, 172.69, 172.60, 171.52, 170.91, 169.98, 169.63, 169.41, 136.27, 128.69, 128.30, 78.05, 74.89, 74.08, 66.21, 59.69 (t, J = 26.9 Hz), 39.44, 39.33, 39.28, 34.46, 34.43, 32.06, 31.95, 31.87, 30.66, 29.80, 29.78, 29.76, 29.75, 29.70, 29.60, 29.57, 29.50, 29.36, 29.33, 29.28, 29.25, 29.05, 28.80, 26.98, 25.14, 25.11, 25.08, 25.02, 24.90, 22.83, 22.76, 18.92, 17.08, 14.26, 14.22.

^{19}F NMR (376 MHz, CDCl$_3$): δ / ppm = -84.05 – -85.87 (m, 3 F, $CF_3{}^{14}$), -123.33 – -127.03 (m, 2 F, $CF_2{}^{15}$), -131.26 – -133.96 (m, 2 F, $CF_2{}^{15}$). Total integral of CF_2 region normalized with respect to the $CF_3{}^{14}$ group = 4.

ESI-MS [*m/z*]: [M + H]$^+$ calculated for $^{12}C_{76}{}^{1}H_{126}{}^{16}O_{13}{}^{14}N_3{}^{19}F_7$, 1422.9252; found, 1422.9239, Δ = 1.3 mmu.

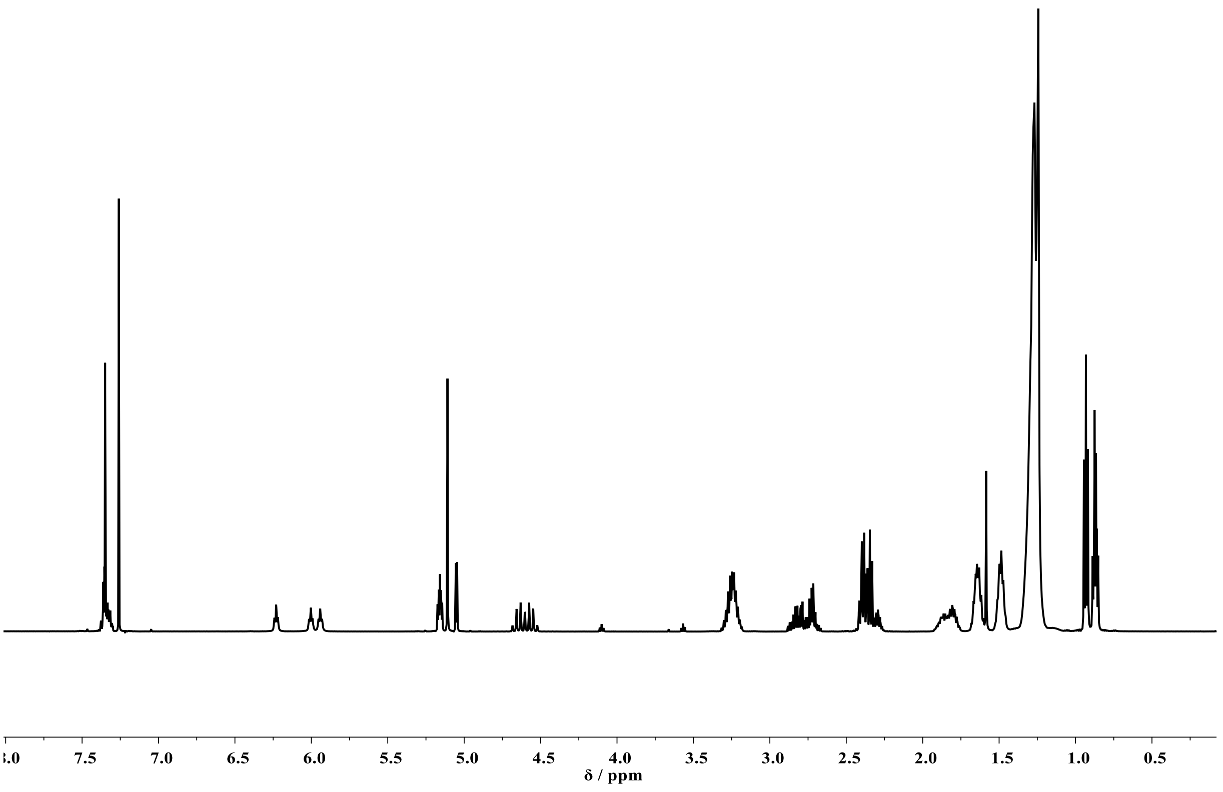

Supplementary Figure 131: ^{1}H-NMR of compound 88 measured in $CDCl_3$.

Deprotection

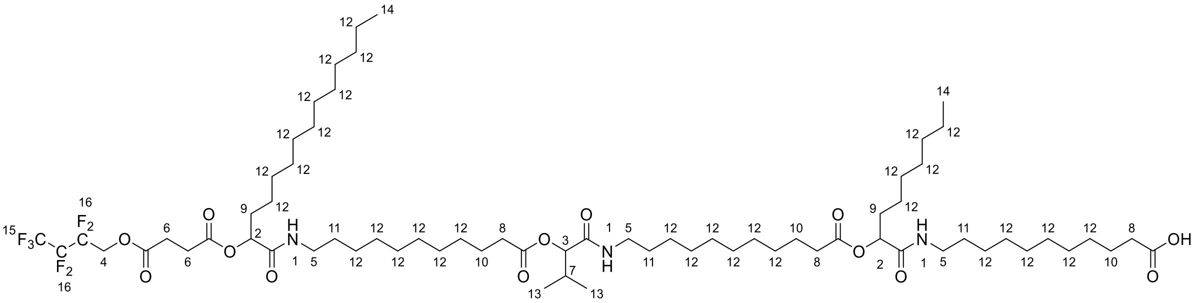

89

In a 50 mL round bottom flask, 363 mg of **88** (255 µmol, 1.00 eq.) were dissolved in 5.00 mL ethyl acetate and 5.00 mL THF. Afterwards, 166 mg (20 wt%) palladium on activated charcoal **16** were added. Subsequently, the mixture was purged with hydrogen (3 balloons) and stirred under hydrogen atmosphere overnight. The heterogeneous catalyst was filtered off and the solvent was evaporated under reduced pressure. The product **89** was obtained as a pale highly viscous oil in a yield of 97.3% (331 mg, 249 µmol).

IR (ATR): ν / cm^{-1} = 3274.6 (vw), 2919.2 (vs), 2850.9 (s), 1740.1 (vs), 1654.7 (vs), 1545.2 (m), 1466.5 (w), 1365.9 (w), 1227.7 (vs), 1146.3 (vs), 1020.3 (m), 911.5 (w), 721.6 (w), 535.5 (vw), 474.0 (vw), 407.2 (vw).

^{1}H NMR (500 MHz, $CDCl_3$): *δ* / ppm = 6.20 (t, *J* = 5.8 Hz, 1 H, NH^1), 6.03 – 5.93 (m, 2 H, NH^1), 5.14 – 5.07 (m, 2 H, CH^2), 4.98 (d, *J* = 4.5 Hz, 1 H, CH^3), 4.67 – 4.43 (m, 2 H, $CH_2{}^4$), 3.28 – 3.12 (m, 6 H, $CH_2{}^5$), 2.85 – 2.60 (m, 4 H, $CH_2{}^6$), 2.37 – 2.18 (m, 7 H, CH^7, $CH_2{}^8$), 1.91 – 1.68 (m, 4 H, $CH_2{}^9$), 1.64 – 1.50 (m, 6 H, $CH_2{}^{10}$), 1.48 – 1.37 (m, 6 H, $CH_2{}^{11}$), 1.33 – 1.06 (m, 66 H, $CH_2{}^{12}$), 0.87 (t, *J* = 6.4 Hz, 6 H, $CH_3{}^{13}$), 0.83 – 0.76 (m, 6 H, $CH_3{}^{14}$).

^{13}C NMR (126 MHz, $CDCl_3$): *δ* / ppm = 177.05, 172.80, 172.63, 171.54, 170.93, 170.08, 169.76, 169.61, 78.07, 74.87, 74.09, 59.69 (t, *J* = 26.9 Hz), 39.47, 39.35, 39.29, 34.47, 34.42, 33.91, 32.05, 32.01, 31.93, 31.87, 30.62, 29.79, 29.78, 29.75, 29.70, 29.69, 29.60, 29.58, 29.56, 29.52, 29.51, 29.49, 29.43, 29.38, 29.37, 29.33, 29.26, 29.24, 29.21, 29.08, 29.04, 28.79, 26.97, 26.94, 26.88, 25.13, 25.12, 25.01, 24.90, 24.88, 22.82, 22.75, 18.89, 17.09, 14.25, 14.21.

ESI-MS [*m/z*]: $[M + H]^+$ calculated for $^{12}C_{70}{}^{1}H_{120}{}^{16}O_{11}{}^{14}N_3{}^{35}Cl$, 1214.8684; found, 1214.8655, Δ = 2.9 mmu.

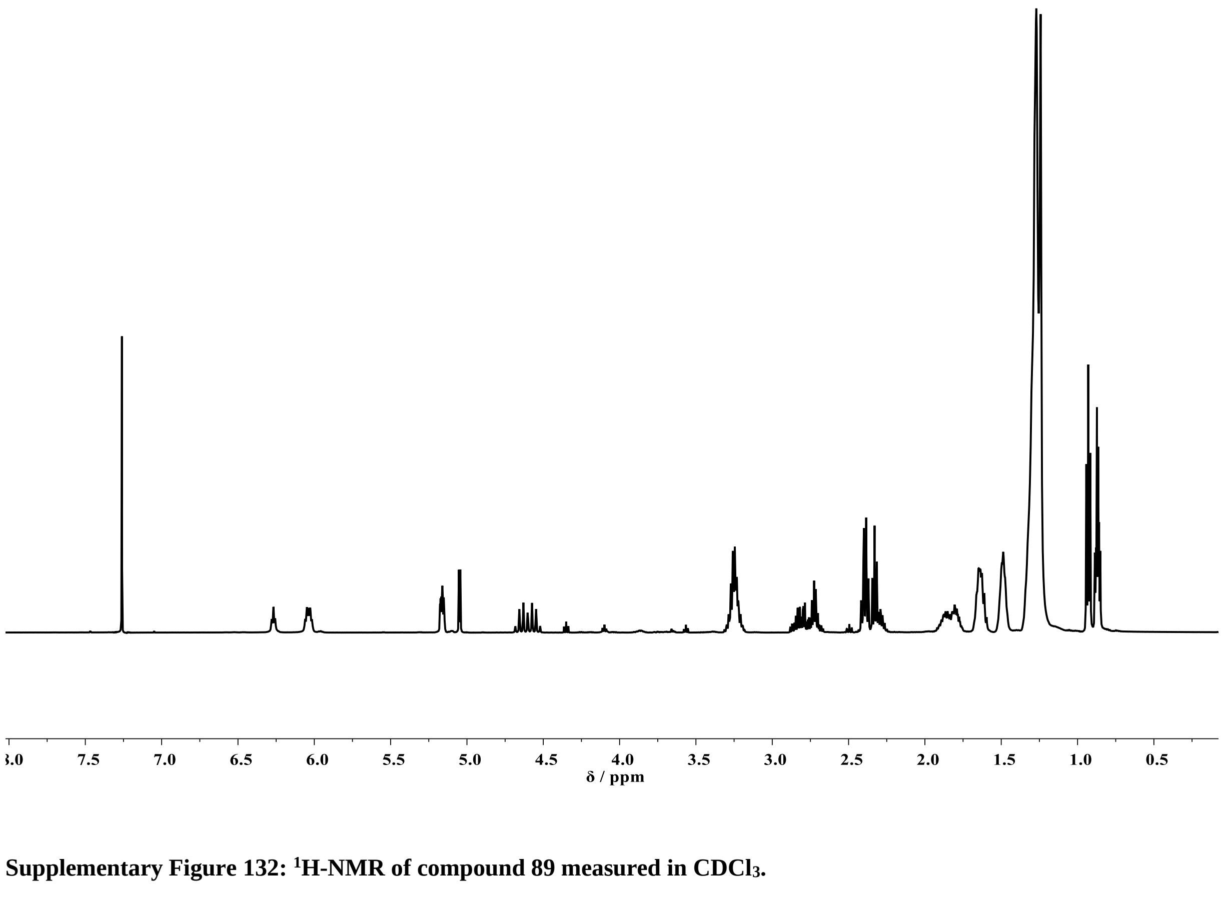

Supplementary Figure 132: ^{1}H-NMR of compound 89 measured in $CDCl_3$.

Passerini reaction

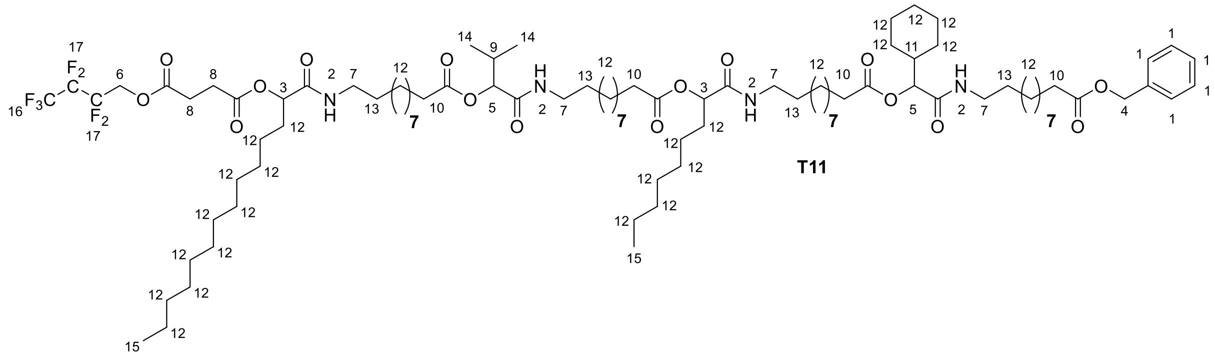

In a 50 mL round bottom flask, 281 mg of **89** (211 µmol, 1.00 eq.) was stirred in 3.00 mL dichloromethane. Subsequently, 38.3 µL cyclohexancarboxaldehyde **14j** (35.5 mg, 316 µmol, 1.50 eq.) and 95.3 mg of the monomer **M1** (316 µmol, 1.50 eq.) were added. The resulting reaction mixture was stirred at room temperature for 2 days. Afterwards, the crude mixture was dried under reduced pressure. The residue was adsorbed onto celite® and purified *via* column chromatography on silica gel eluting with a gradual solvent mixture of ethyl acetate and cyclohexane (4:1 → 2:1) to yield the passerini product **T11** as a pale highly viscous oil. (331 mg, 190 µmol, 90.0%).

R_f = 0.46 in cyclohexane / ethyl acetate (2:1).

IR (ATR): ν / cm^{-1} = 3302.4 (w), 2920.6 (vs), 2851.2 (s), 1735.2 (vs), 1655.3 (vs), 1555.1 (m), 1466.2 (m), 1377.7 (w), 1228.1 (vs), 1207.0 (vs), 1172.6 (vs), 1020.5 (w), 722.7 (w), 696.5 (m).

^{1}H NMR (500 MHz, $CDCl_3$): δ / ppm = 7.35 – 7.22 (m, 5 H, $CH_{Ar}{}^{1}$), 6.19 (t, *J* = 5.8 Hz, 1 H, NH^{2}), 5.97 (t, *J* = 5.9 Hz, 1 H, NH^{2}), 5.93 – 5.81 (m, 2 H, NH^{2}), 5.12 – 5.07 (m, 2 H, CH^{3}), 5.04 (s, 2 H, $CH_2{}^{4}$), 4.98 (d, *J* = 4.4 Hz, 1 H, CH^{5}), 4.96 (d, *J* = 4.6 Hz, 1 H, CH^{5}), 4.65 – 4.43 (m, 2 H, $CH_2{}^{6}$), 3.29 – 3.09 (m, 8 H, $CH_2{}^{7}$), 2.84 – 2.60 (m, 4 H, $CH_2{}^{8}$), 2.41 – 2.18 (m, 9 H, CH^{9},$CH_2{}^{10}$), 1.95 – 1.52 (m, 21 H, CH^{11}, $CH_2{}^{12}$), 1.48 – 1.36 (m, 8 H, $CH_2{}^{13}$), 1.30 – 0.94 (m, 18 H, $CH_2{}^{12}$), 0.87 (t, *J* = 6.5 Hz, 6 H, $CH_3{}^{14}$), 0.85 – 0.76 (m, 6 H, $CH_3{}^{15}$).

^{13}C NMR (126 MHz, $CDCl_3$): δ / ppm = 173.80, 172.67, 172.66, 172.60, 171.49, 170.89, 169.98, 169.62, 169.40, 169.32, 136.24, 128.66, 128.28, 128.27, 78.03, 77.74, 74.86, 74.05, 66.19, 59.66 (t, *J* = 27.0 Hz), 40.10, 39.43, 39.31, 39.27, 34.44, 34.41, 32.04, 31.94, 31.86, 30.64, 29.78, 29.77, 29.74, 29.72, 29.70, 29.67, 29.58, 29.57, 29.55, 29.54, 29.51, 29.49, 29.47, 29.36, 29.34, 29.32, 29.26, 29.23, 29.03, 28.78, 27.41, 26.97, 26.95, 26.20, 26.12, 26.01, 25.12, 25.09, 25.07, 25.01, 24.89, 22.81, 22.74, 18.90, 17.07, 14.25, 14.21.

^{19}F NMR (376 MHz, $CDCl_3$): δ / ppm = -85.14 (t, *J* = 9.2 Hz, 3 F, $CF_3{}^{16}$), -124.10 – -125.56 (m, 2 F, $CF_2{}^{17}$), -131.03 – -133.61 (m, 2 F, $CF_2{}^{17}$). Total integral of CF_2 region normalized with respect to the $CF_3{}^{16}$ group = 4.

ESI-MS [*m/z*]: [M + H]$^{+}$ calculated for $^{12}C_{95}{}^{1}H_{159}{}^{16}O_{16}{}^{14}N_4{}^{19}F_7$, 1746.1712; found, 1746.1708, Δ = 0.4 mmu.

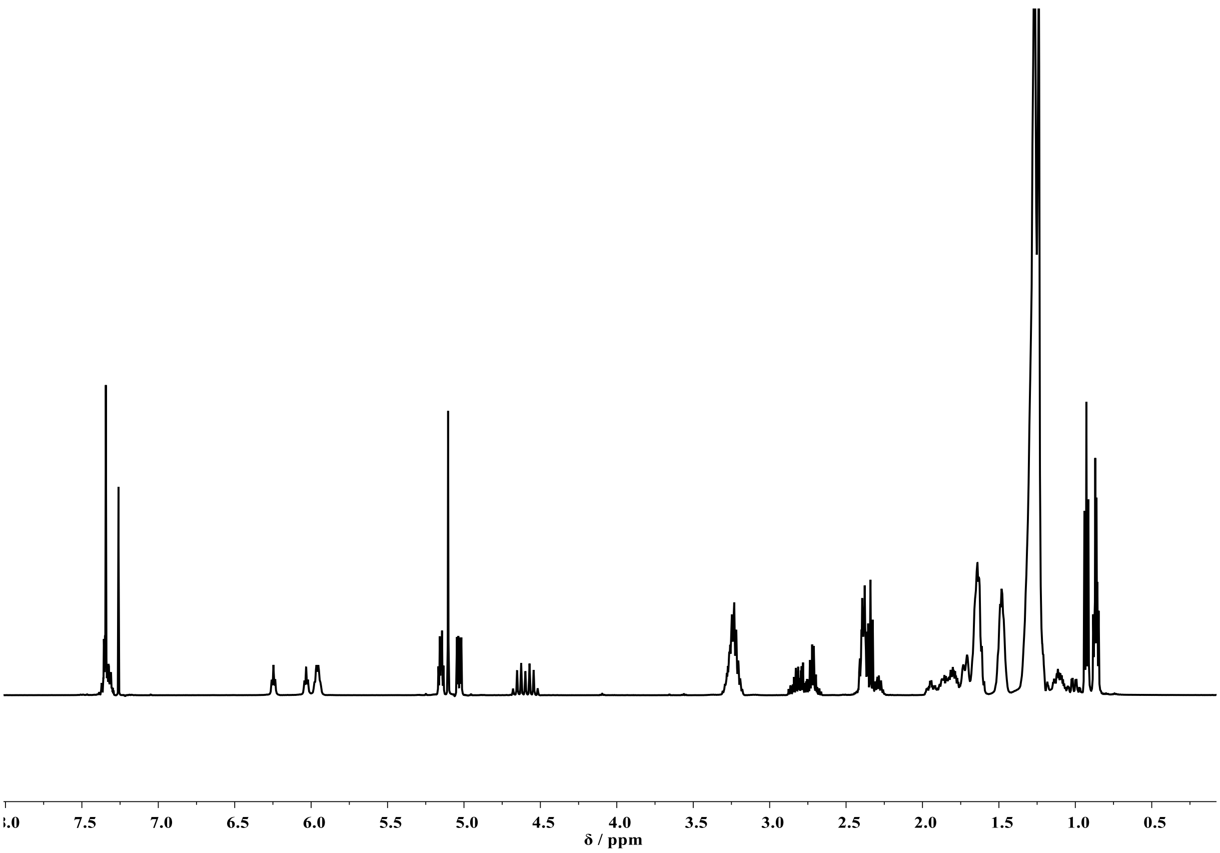

Supplementary Figure 133: ^{1}H-NMR of compound T11 measured in $CDCl_3$.

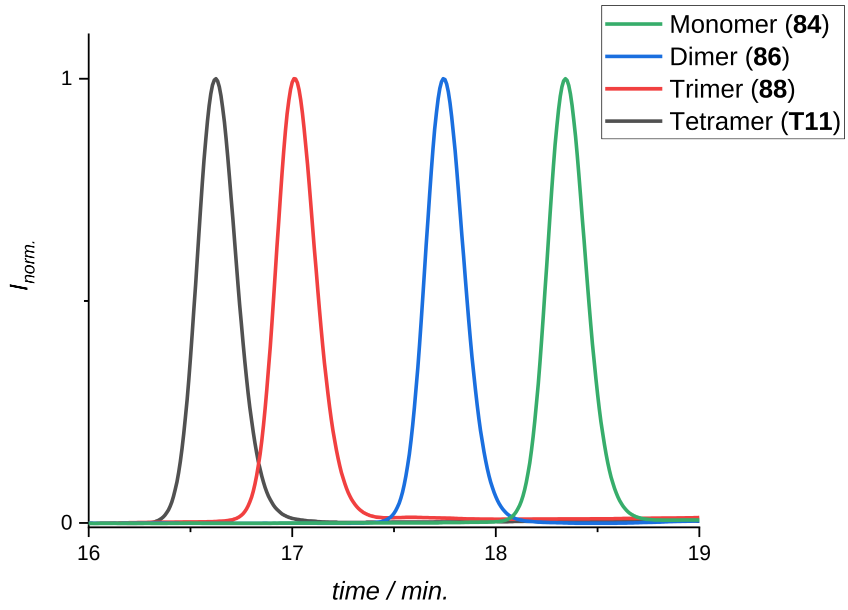

Supplementary Figure 134: SEC traces of the intermediates after each P3CR I the synthesis of product T11.

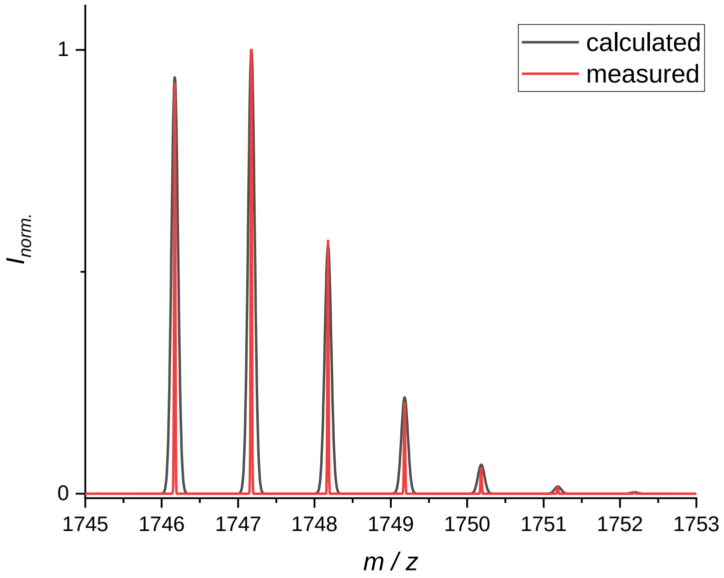

Supplementary Figure 135: High resolution ESI-MS measurement of T11. The observed isotopic pattern is compared with the calculated isotopic pattern obtained from mMass (black).

```
matching mass 1747.17426
cutoff 0.50000: 0 solutions (16 peaks)
cutoff 0.25000: 0 solutions (42 peaks)
cutoff 0.12500: 0 solutions (106 peaks)
cutoff 0.06250: 0 solutions (216 peaks)
cutoff 0.03125: 0 solutions (394 peaks)
cutoff 0.01562: 0 solutions (654 peaks)
cutoff 0.00781: 1 solutions (1071 peaks)
1747.17426 ≈ 283.020520 + 409.355600 + 283.214750 + 339.277350 + 323.246050 + 107.049690 (sides Tridecanal,
 Isobutyraldehyde, Octanal, Cyclohexancarboxaldehyde; error -2.01030)
Press ENTER to quit ...
```

Supplementary Figure 136: Screenshot of the automated read-out of T11.

1.3.5.5 Synthesis of tetramer T12

Passerini reaction

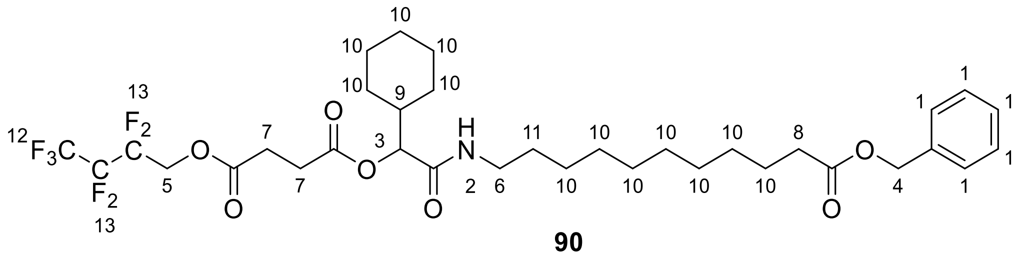

90

In a 50 mL round bottom flask, 304 mg **TAG2** (1.01 mmol, 1.00 eq.) was stirred in 3.00 mL dichloromethane. Subsequently, 204 µL cyclohexancarboxaldehyde **14j** (170 mg, 1.52 mmol, 1.50 eq.) and 458 mg of the monomer **M1** (1.52 mol, 1.50 eq.) were added. The resulting reaction mixture was stirred at room temperature for 2 days. Afterwards, the crude mixture was dried under reduced pressure. The residue was adsorbed onto celite® and purified *via* column chromatography on silica gel eluting with a gradual solvent mixture of ethyl acetate and cyclohexane (7:1 → 5:1) to yield the passerini product **90** as a pale highly viscous oil. (663 mg, 927 µmol, 91.8%).

R_f = 0.47 in cyclohexane / ethyl acetate (3:1).

IR (ATR): ν / cm^{-1} = 3306.8 (vw), 2927.0 (m), 2854.6 (w), 1737.7 (s), 1655.8 (m), 1534.5 (w), 1452.0 (w), 1351.8 (w), 1226.0 (vs), 1142.5 (vs), 1019.7 (m), 910.1 (m), 735.9 (m), 697.2 (m).

^{1}H NMR (400 MHz, $CDCl_3$): δ / ppm = 7.46 – 7.29 (m, 5 H, CH_{Ar}^{1}), 6.19 (t, J = 5.8 Hz, 1 H, NH^{2}), 5.11 (s, 2 H, CH_2^{3}), 5.04 (d, J = 4.0 Hz, 1 H, CH^{4}), 4.74 – 4.46 (m, 2 H, CH_2^{5}), 3.35 – 3.12 (m, 2 H, CH_2^{6}), 2.91 – 2.66 (m, 4 H, CH_2^{7}), 2.34 (t, J = 7.6 Hz, 2 H, CH_2^{8}), 2.07 – 1.92 (m, 1 H, CH_2^{9}), 1.80 – 1.57 (m, 8 H, CH_2^{10}), 1.55 – 1.42 (m, 2 H, CH_2^{11}), 1.37 – 0.96 (m, 16 H, CH_2^{10}).

^{13}C NMR (101 MHz, $CDCl_3$): δ / ppm = 173.83, 171.54, 170.93, 168.95, 136.27, 128.67, 128.29, 78.58, 66.19, 59.71 (t, J = 27.1 Hz), 39.91, 39.40, 34.45, 29.57, 29.56, 29.47, 29.42, 29.33, 29.23, 29.00, 28.80, 27.14, 26.97, 26.15, 26.01, 25.06.

^{19}F NMR (376 MHz, $CDCl_3$): δ / ppm = -85.16 (t, J = 9.2 Hz, 3 F, CF_3^{12}), -124.49 – -125.85 (m, 2 F, CF_2^{13}), -131.00 – -132.82 (m, 2 F, CF_2^{13}).Total integral of CF_2 region normalized with respect to the CF_3^{13} group = 4.

ESI-MS [*m/z*]: $[M + H]^+$ calculated for $^{12}C_{34}{}^{1}H_{46}{}^{16}O_{7}{}^{14}N^{19}F_{7}$, 714.3235; found, 714.3226, Δ = 0.9 mmu.

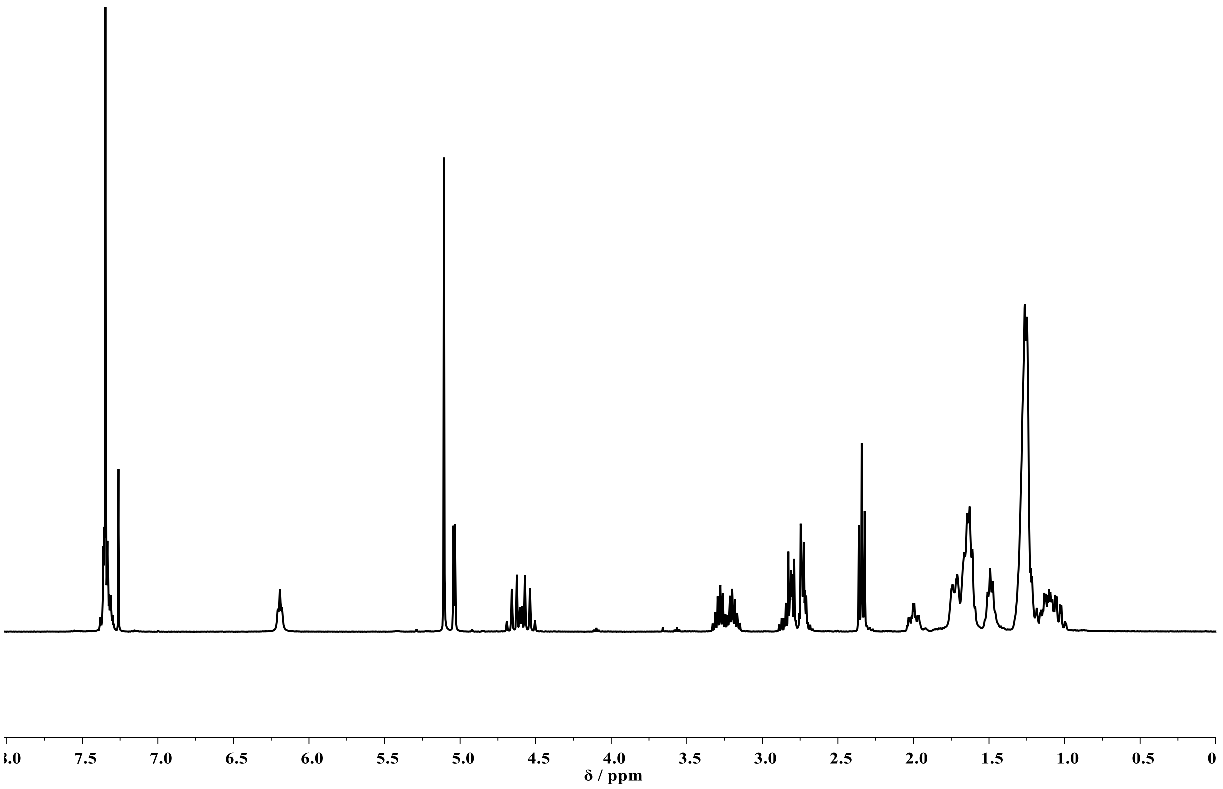

Supplementary Figure 137: ^{1}H-NMR of compound 90 measured in $CDCl_3$.

Deprotection

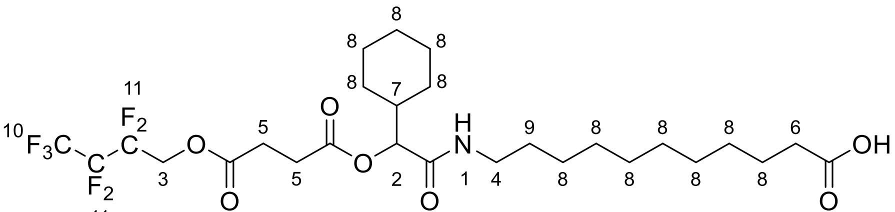

91

In a 50 mL round bottom flask, 625 mg of **90** (876 µmol, 1.00 eq.) were dissolved in 3.00 mL ethyl acetate and 3.00 mL THF. Afterwards, 125 mg (20 wt%) palladium on activated charcoal **16** were added. Subsequently, the mixture was purged with hydrogen (3 balloons) and stirred under hydrogen atmosphere overnight. The heterogeneous catalyst was filtered off and the solvent was evaporated under reduced pressure. The product **91** was obtained as a pale highly viscous oil in a yield of 99.6% (544 mg, 872 µmol).

IR (ATR): ν / cm^{-1} = 3313.2 (w), 2926.8 (s), 2853.3 (m), 1765.1 (s), 1736.3 (vs), 1686.1 (vs), 1655.2 (vs), 1552.0 (s), 1447.0 (w), 1381.9 (w), 1352.7 (m), 1298.0 (m), 1217.5 (vs), 1144.9 (vs), 1115.9 (vs), 1084.8 (m), 1023.4 (w), 984.8 (s), 955.5 (m), 912.4 (m), 872.9 (w), 843.8 (vw), 784.1 (w), 737.0 (vs), 671.5 (w), 573.5 (w), 540.4 (w), 451.3 (w), 381.1 (vw).

^{1}H NMR (400 MHz, $CDCl_3$): *δ* / ppm = 6.17 (t, *J* = 5.8 Hz, 1 H, NH^1), 4.98 (d, *J* = 4.1 Hz, 1 H, CH^2), 4.68 – 4.41 (m, 2 H, CH_2^3), 3.31 – 3.07 (m, 2 H, CH_2^4), 2.84 – 2.59 (m, 4 H, CH_2^5), 2.27 (t, *J* = 7.5 Hz, 2 H, CH_2^6), 2.00 – 1.87 (m, 1 H, CH^7), 1.73 – 1.51 (m, 6 H, CH_2^8), 1.47 – 1.37 (m, 2 H, CH_2^9), 1.30 – 0.90 (m, 18 H, CH_2^8).

^{13}C NMR (101 MHz, $CDCl_3$): *δ* / ppm = 179.13, 171.58, 170.98, 169.10, 78.56, 59.71 (t, *J* = 27.0 Hz), 39.88, 39.42, 34.12, 29.47, 29.40, 29.35, 29.23, 29.22, 29.09, 28.99, 28.79, 27.12, 26.91, 26.13, 26.00, 24.81.

^{19}F NMR (376 MHz, $CDCl_3$): *δ* / ppm = -85.18 (t, *J* = 9.2 Hz, 3 F, CF_3^{10}), -124.03 –125.46 (m, 2 F, CF_2^{11}), -131.58 – -132.63 (m, 2 F, CF_2^{11}). Total integral of CF_2 region normalized with respect to the CF_3^{10} group = 4.

ESI-MS [*m/z*]: $[M + H]^+$ calculated for $^{12}C_{27}{}^{1}H_{40}{}^{16}O_7{}^{14}N^{19}F_7$, 624.2766; found, 624.2752, Δ = 1.4 mmu.

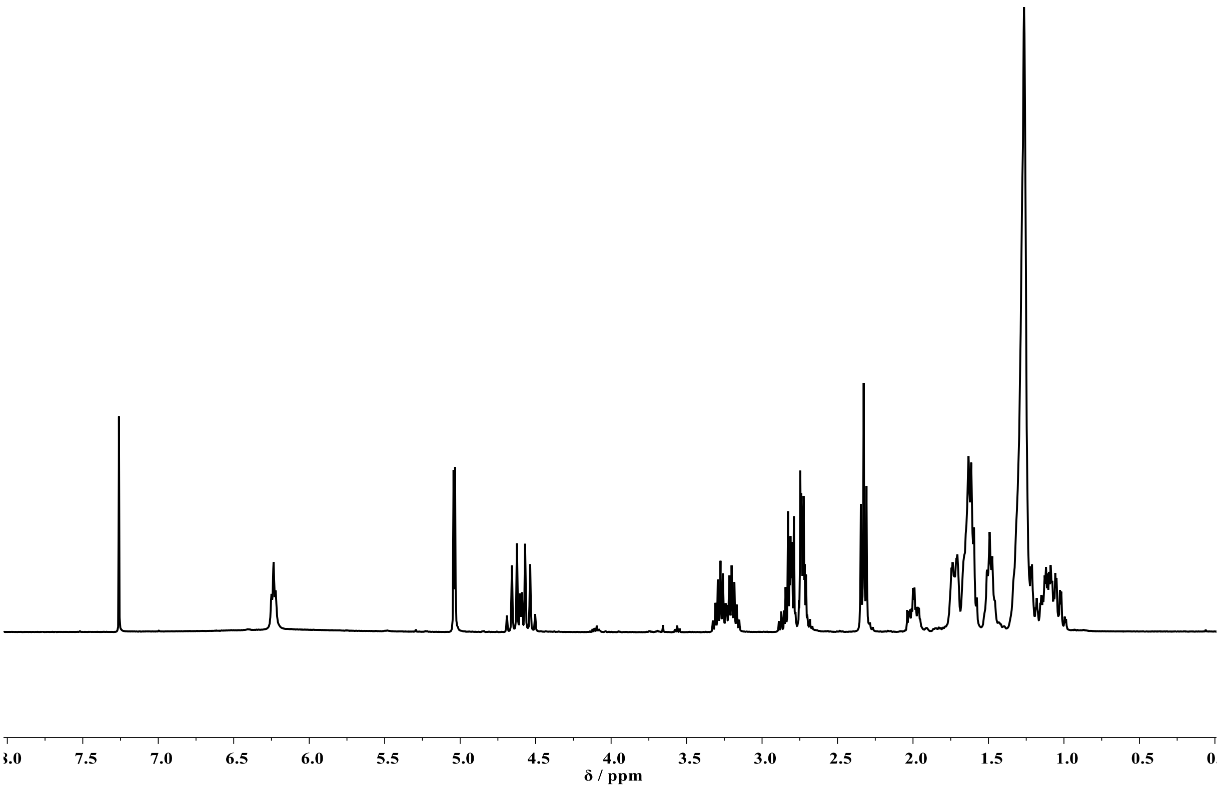

Supplementary Figure 138: ^{1}H-NMR of compound 91 measured in $CDCl_3$.

Passerini reaction

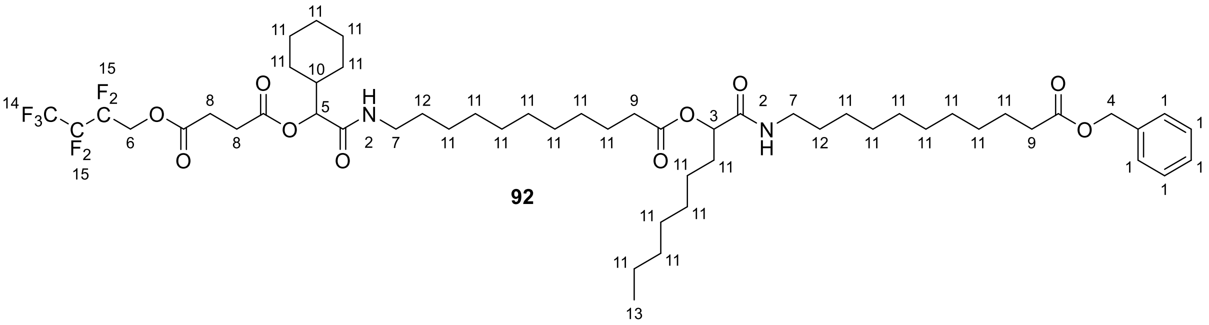

In a 50 mL round bottom flask, 419 mg of **91** (671 µmol, 1.00 eq.) was stirred in 3.00 mL dichloromethane. Subsequently, 194 µL octanal **14i** (160 mg, 1.25 mmol, 1.85 eq.) and 374 mg of the monomer **M1** (1.25 mol, 1.85 eq.) were added. The resulting reaction mixture was stirred at room temperature for 2 days. Afterwards, the crude mixture was dried under reduced pressure. The residue was adsorbed onto celite® and purified *via* column chromatography on silica gel eluting with a gradual solvent mixture of ethyl acetate and cyclohexane (4:1 → 2:1) to yield the passerini product **92** as a pale highly viscous oil. (663 mg, 927 µmol, 91.8%).

R_f = 0.32 in cyclohexane / ethyl acetate (2:1).

IR (ATR): ν / cm^{-1} = 3293.7 (w), 2918.5 (s), 2850.5 (s), 1728.6 (vs), 1679.6 (m), 1651.3 (vs), 1533.4 (m), 1466.9 (w), 1320.5 (m), 1227.7 (vs), 1158.9 (vs), 1021.9 (m), 957.0 (w), 909.9 (w), 803.6 (vw), 735.6 (w), 720.1 (w), 697.8 (m), 628.5 (w), 539.8 (w), 449.2 (vw), 383.7 (vw).

^{1}H NMR (400 MHz, $CDCl_3$): δ / ppm = 7.45 – 7.28 (m, 5 H, CH_{Ar}^{1}), 6.20 (t, J = 5.8 Hz, 1 H, NH^{2}), 6.00 (t, J = 5.8 Hz, 1 H, NH^{2}), 5.19 – 5.13 (m, 1 H, CH^{3}), 5.11 (s, 2 H, CH_2^{4}), 5.03 (d, J = 4.1 Hz, 1 H, CH^{5}), 4.72 – 4.48 (m, 2 H, CH_2^{6}), 3.36 – 3.13 (m, 4 H, CH_2^{7}), 2.90 – 2.66 (m, 4 H, CH_2^{8}), 2.44 – 2.30 (m, 4 H, CH_2^{9}), 2.08 – 1.93 (m, 1 H, CH^{10}), 1.90 – 1.56 (m, 14 H, CH_2^{11}), 1.56 – 1.44 (m, 4 H, CH_2^{12}), 1.38 – 0.98 (m, 36 H, CH_2^{11}), 0.90 – 0.83 (m, 3 H, CH_3^{13}).

^{13}C NMR (101 MHz, $CDCl_3$): δ / ppm = 173.83, 172.59, 171.55, 170.93, 169.99, 168.96, 136.26, 128.68, 128.29, 78.58, 74.07, 66.21, 59.84 (t, J = 27.0 Hz), 39.91, 39.39, 39.33, 34.46, 32.04, 31.86, 29.69, 29.60, 29.58, 29.57, 29.49, 29.42, 29.35, 29.33, 29.24, 29.23, 29.00, 28.80, 27.14, 27.05, 26.97, 26.15, 26.02, 25.09, 25.08, 24.88, 22.74, 14.20.

^{19}F NMR (376 MHz, $CDCl_3$): δ / ppm = -85.15 (t, J = 9.7 Hz, 3 F, CF_3^{14}), -124.10 – -125.74 (m, 2 F, CF_2^{15}), -130.32 – -133.43 (m, 2 F, CF_2^{15}). Total integral of CF_2 region normalized with respect to the CF_3^{14} group = 4.

ESI-MS [*m/z*]: $[M + H]^+$ calculated for $^{12}C_{54}{}^{1}H_{83}{}^{16}O_{10}{}^{14}N_2{}^{19}F_7$, 1053.6009; found, 1053.6000, Δ = 0.9 mmu.

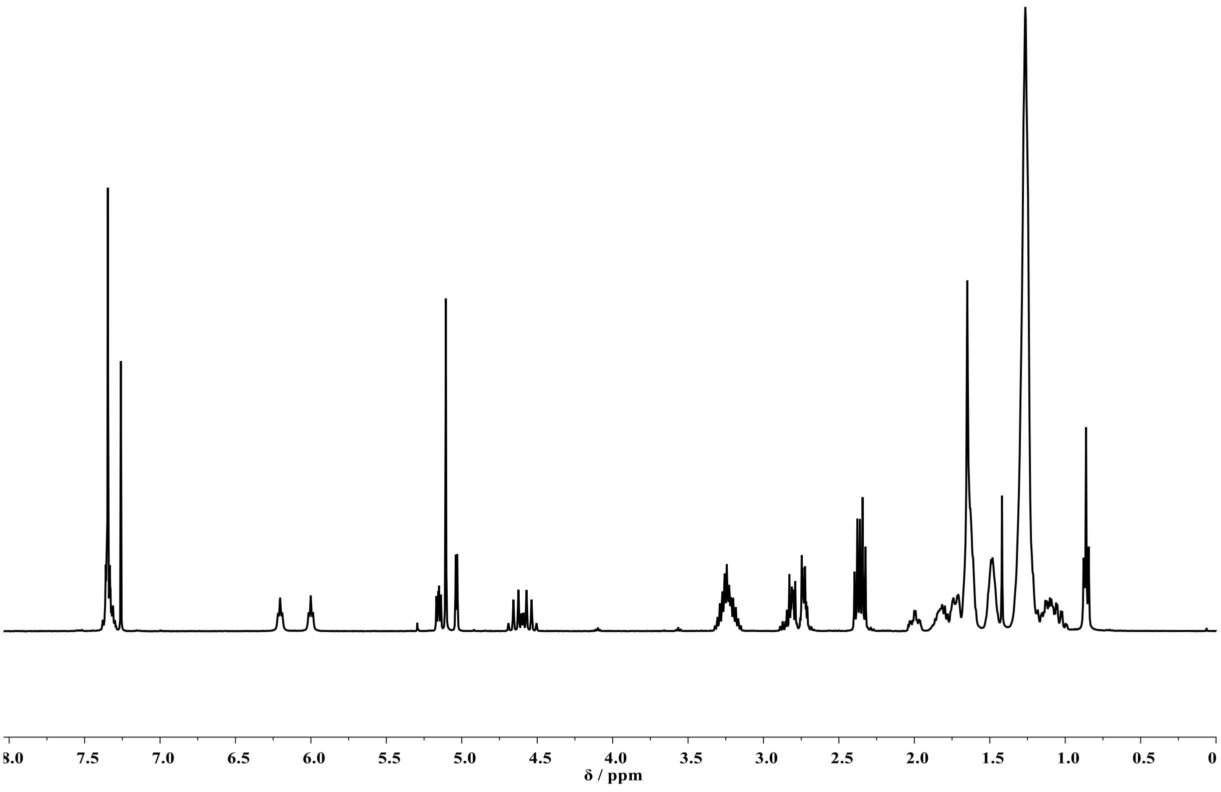

Supplementary Figure 139: ^{1}H-NMR of compound 92 measured in $CDCl_3$.

Deprotection

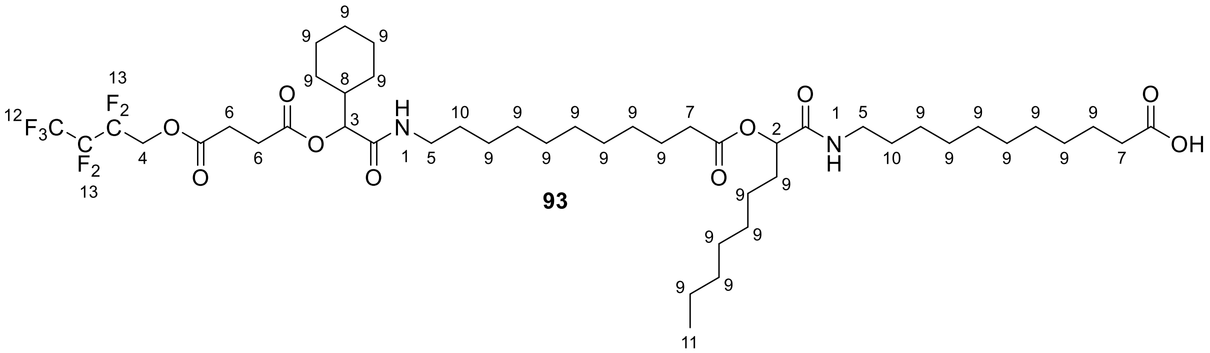

In a 25 mL round bottom flask, 497 mg of **92** (472 µmol, 1.00 eq.) was dissolved in 4.00 mL ethyl acetate and 4.00 mL THF. Subsequently, 99.4 mg (20 wt.-%) palladium on activated charcoal **16** was added to the solution. The resulting mixture was purged with hydrogen gas and stirred for 1 day at room temperature under hydrogen atmosphere (3 balloons). The heterogeneous catalyst was filtered off and the solvent was evaporated under reduced pressure. The product **93** was obtained as a yellow high viscos oil. (429 mg, 445 µmol, 94.4%).

IR (ATR): ν / cm^{-1} = 3316.2 (vw), 2927.8 (w), 2855.4 (vw), 2360.7 (w), 2343.5 (vw), 2328.7 (vw), 2154.8 (vw), 1743.0 (w), 1656.5 (vw), 1544.0 (vw), 1453.7 (vw), 1354.3 (vw), 1229.6 (w), 1181.7 (vw), 1146.8 (w), 1020.7 (vw), 736.0 (vw), 443.1 (vw), 418.4 (vw).

^{1}H NMR (400 MHz, $CDCl_3$): δ / ppm = 6.29 (t, J = 6.0 Hz, 1 H, NH^{1}), 6.07 (t, J = 6.0 Hz, 1 H, NH^{1}), 5.16 (t, J = 6.1 Hz, 1 H, CH^{2}), 5.05 (d, J = 4.2 Hz, 1 H, CH^{3}), 4.76 – 4.47 (m, 2 H, CH_2^{4}), 3.41 – 3.12 (m, 4 H, CH_2^{5}), 2.93 – 2.69 (m, 4 H, CH_2^{6}), 2.48 – 2.28 (m, 4 H, CH_2^{7}), 2.10 – 1.95 (m, 1 H, CH^{8}), 1.92 – 0.99 (m, 54 H, CH_2^{9}), 0.87 (t, J = 6.6 Hz, 3 H, CH_3^{10}).

^{13}C NMR (101 MHz, $CDCl_3$): δ / ppm = 178.20, 172.61, 171.54, 170.96, 170.11, 169.12, 78.57, 74.08, 59.71 (t, J = 27.1 Hz), 39.89, 39.44, 39.33, 34.45, 34.13, 32.01, 31.84, 29.59, 29.53, 29.48, 29.38, 29.35, 29.31, 29.25, 29.20, 29.13, 28.99, 28.79, 27.17, 26.96, 26.90, 26.13, 26.00, 25.10, 24.89, 22.72, 14.16.

^{19}F NMR (376 MHz, $CDCl_3$): δ / ppm = -84.58 – -85.52 (m, 3 F, CF_3^{11}), -124.10 – -125.92 (m, 2 F, CF_2^{12}), -130.67 – -133.43 (m, 2 F, CF_2^{12}). Total integral of CF_2 region normalized with respect to the CF_3^{11} group = 4.

ESI-MS [m/z]: [M + Na]$^{+}$ calculated for $^{12}C_{47}{}^{1}H_{77}{}^{16}O_{10}{}^{14}N_2{}^{19}F_7{}^{23}Na$, 985.5359; found, 985.5341, Δ = 1.8 mmu.

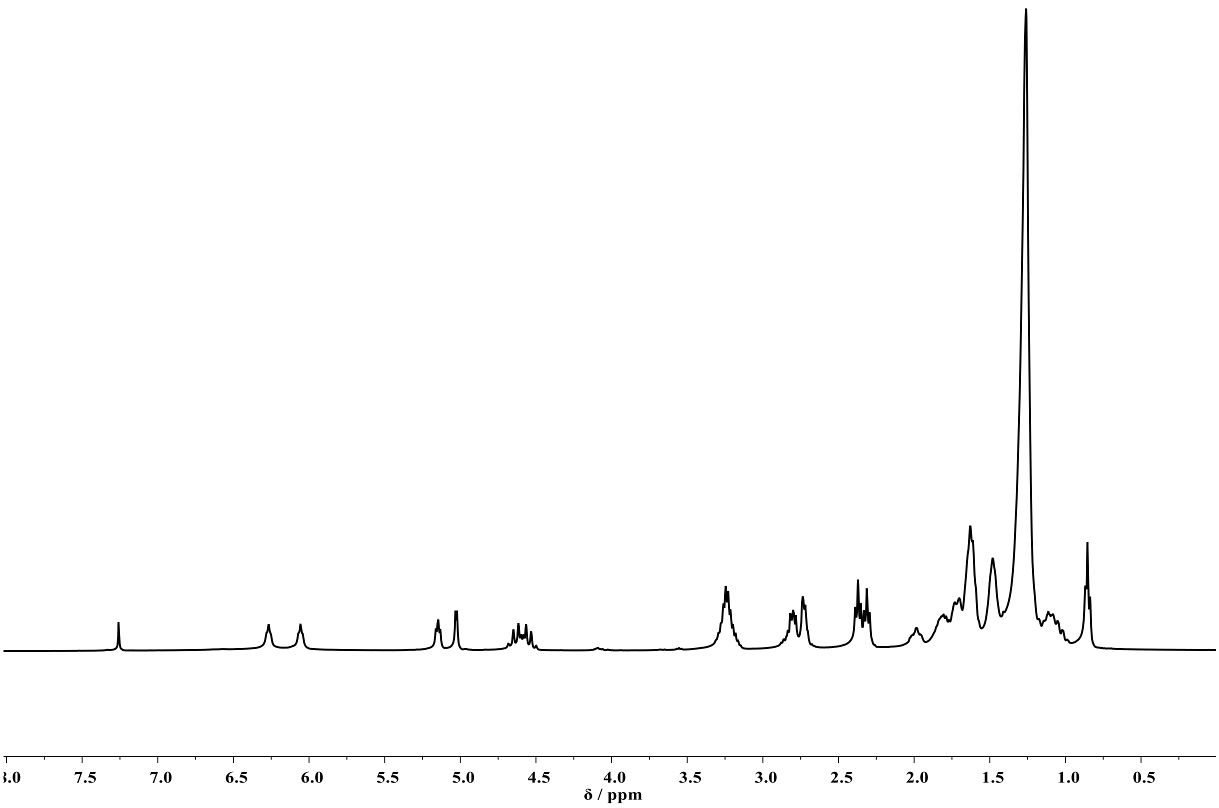

Supplementary Figure 140: ^{1}H-NMR of compound 93 measured in $CDCl_3$.

Passerini reaction

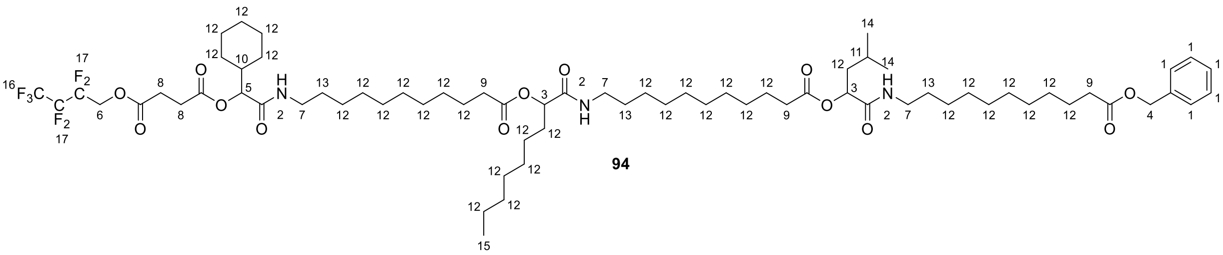

94

In a 50 mL round bottom flask, 361 mg of **94** (375 µmol, 1.00 eq.) was stirred in 4.00 mL dichloromethane. Subsequently, 60.5 µL 3-methylbutyraldehyde **14g** (48.4 mg, 562 µmol, 1.50 eq.) and 169 mg of the monomer **M1** (562 µmol, 1.50 eq.) were added. The resulting reaction mixture was stirred at room temperature for 2 days. Afterwards, the crude mixture was dried under reduced pressure. The residue was adsorbed onto celite® and purified *via* column chromatography on silica gel eluting with a gradual solvent mixture of ethyl acetate and cyclohexane (5:1 → 2:1) to yield the passerini product **94** as a pale highly viscous oil. (377 mg, 279 µmol, 74.5%).

R_f = 0.30 in cyclohexane / ethyl acetate (2:1).

IR (ATR): ν / cm^{-1} = 3307.8 (vw), 2926.3 (w), 2854.6 (w), 1740.3 (w), 1654.7 (w), 1537.7 (w), 1454.8 (vw), 1353.3 (vw), 1227.3 (m), 1144.9 (m), 1020.0 (vw), 910.7 (vw), 735.7 (vw), 697.4 (vw), 453.0 (vw), 431.1 (vw).

^{1}H-NMR (400 MHz, $CDCl_3$): δ / ppm = 7.44 – 7.28 (m, 5 H, CH_{Ar}^{1}), 6.21 (t, J = 5.8 Hz, 1 H, NH^{2}), 6.07 – 5.91 (m, 2 H, NH^{2}), 5.26 – 5.14 (m, 2 H, CH^{3}), 5.11 (s, 2 H, CH_2^{4}), 5.04 (d, J = 4.0 Hz, 1 H, CH^{5}), 4.74 – 4.45 (m, 2 H, CH_2^{6}), 3.35 – 3.14 (m, 6 H, CH_2^{7}), 2.91 – 2.68 (m, 4 H, CH_2^{8}), 2.44 – 2.30 (m, 6 H, CH_2^{9}), 2.08 – 1.91 (m, 1 H, CH^{10}), 1.90 – 1.56 (m, 17 H, CH^{11}, CH_2^{12}), 1.56 – 1.38 (m, 6 H, CH_2^{13}), 1.38 – 0.99 (m, 50 H, CH_2^{12}), 0.92 (t, J = 5.7 Hz, 6 H, CH_3^{14}), 0.89 – 0.79 (m, 3 H, CH_3^{15}).

^{13}C NMR (101 MHz, $CDCl_3$): δ / ppm = 173.82, 172.77, 172.60, 171.55, 170.93, 170.34, 170.00, 168.95, 136.27, 128.68, 128.30, 78.58, 74.07, 72.78, 66.21, 59.71, 40.99, 39.91, 39.39, 39.37, 39.32, 34.46, 32.05, 31.86, 30.23, 29.70, 29.66, 29.59, 29.57, 29.50, 29.48, 29.43, 29.34, 29.24, 29.00, 28.81, 27.14, 26.97, 26.94, 26.15, 26.02,25.09, 25.08, 25.06, 24.90, 24.69, 23.82, 23.27, 22.74, 21.94, 14.20.

^{19}F NMR (376 MHz, $CDCl_3$): δ / ppm = -85.14 (t, J = 9.5 Hz, 3 F, CF_3^{16}), -124.79 – -124.91 (m, 2 F, CF_2^{17}), -131.94 – -132.04 (m, 2 F, CF_2^{17}). Total integral of CF_2 region normalized with respect to the CF_3^{16} group = 4.

ESI-MS [*m/z*]: $[M + H]^+$ calculated for $^{12}C_{71}{}^{1}H_{114}{}^{16}O_{13}{}^{14}N_3{}^{19}F_7$, 1350.8313; found, 1350.8312, Δ = 0.1 mmu.

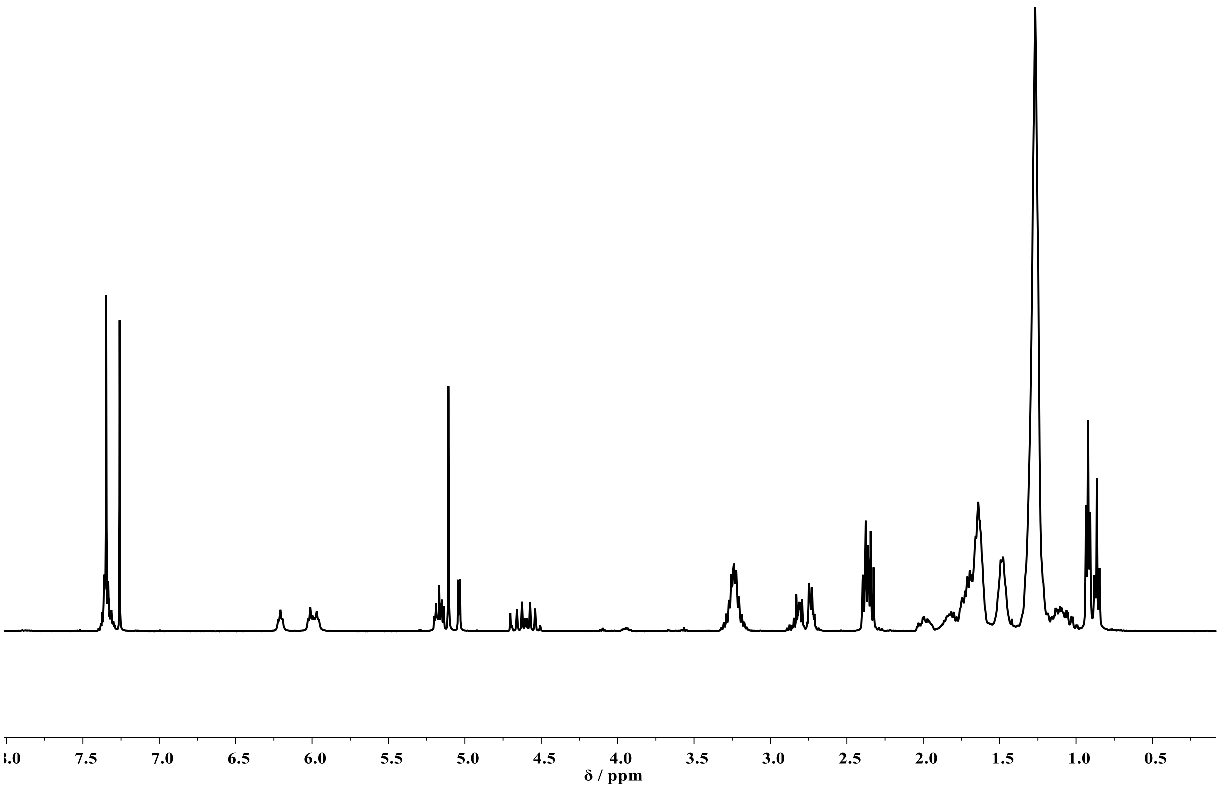

Supplementary Figure 141: ^{1}H-NMR of compound 94 measured in $CDCl_3$.

Deprotection

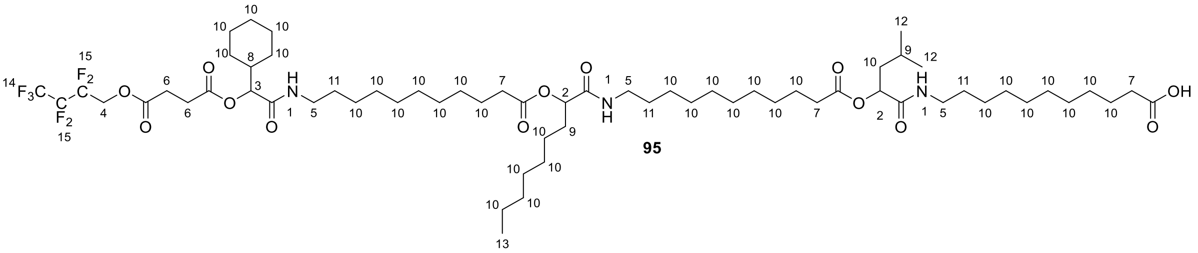

In a 25 mL round bottom flask, 335 mg of **94** (248 µmol, 1.00 eq.) was dissolved in 4.00 mL ethyl acetate and 4.00 mL THF. Subsequently, 84.6 mg (20 wt.-%) palladium on activated charcoal **16** was added to the solution. The resulting mixture was purged with hydrogen gas and stirred for 1 day at room temperature under hydrogen atmosphere (3 balloons). The heterogeneous catalyst was filtered off and the solvent was evaporated under reduced pressure. The product **95** was obtained as a yellow high viscos oil (308 mg, 244 µmol, 98.5%).

IR (ATR): ν / cm^{-1} = 3309.2, 2925.5, 2854.6, 2036.6, 1987.3, 1742.0, 1654.5, 1540.1, 1465.2, 1369.3, 1227.8, 1145.9, 1021.0, 910.8, 735.9, 472.7, 422.0.

^{1}H NMR (400 MHz, $CDCl_3$): δ / ppm = 6.28 – 6.17 (m, 1 H, NH^1), 6.11 – 6.07 (m, 1 H, NH^1), 6.03 – 5.97 (m, 1 H, NH^1), 5.24 – 5.11 (m, 2 H, CH^2), 5.04 (d, J = 4.0 Hz, 1 H, CH^3), 4.71 – 4.48 (m, 2 H, $CH_2{}^4$), 3.33 – 3.15 (m, 6 H, $CH_2{}^5$), 2.91 – 2.67 (m, 4 H, $CH_2{}^6$), 2.43 – 2.29 (m, 6 H, $CH_2{}^7$), 2.04 – 1.94

(m, 1 H, CH^8), 1.93 – 1.59 (m, 17 H, CH^9, CH_2^{10}), 1.56 – 1.42 (m, 6 H, CH_2^{11}), 1.34 – 0.90 (m, 50 H, CH_2^{10}), 0.92 (t, *J* = 5.7 Hz, 6 H, CH_3^{12}), 0.87 (t, *J* = 6.8 Hz, 3 H, CH_3^{13}).

^{13}C NMR (101 MHz, $CDCl_3$): *δ* / ppm = 176.62, 172.82, 172.72, 171.59, 170.96, 170.43, 170.21, 169.08, 78.56, 74.07, 72.80, 40.93, 39.89, 39.42, 39.32, 34.45, 33.85, 32.01, 31.87, 29.67, 29.61, 29.60, 29.55, 29.52, 29.51, 29.42, 29.38, 29.34, 29.32, 29.29, 29.27, 29.23, 29.18, 29.05, 29.00, 28.81, 27.13, 26.97, 26.84, 26.15, 26.02, 25.10, 25.09, 24.89, 24.69, 23.27, 22.75, 21.92, 14.21.

^{19}F NMR (376 MHz, $CDCl_3$): *δ* / ppm = -85.14 (t, *J* = 9.6 Hz, 3 F, CF_3^{14}), -124.79 – -124.90 (m, 2 F, CF_2^{15}), -131.90 – -132.10 (m, 2 F, CF_2^{15}). Total integral of CF_2 region normalized with respect to the CF_3^{14} group = 4.

ESI-MS [*m/z*]: $[M + H]^+$ calculated for $^{12}C_{64}{}^{1}H_{108}{}^{16}O_{13}{}^{14}N_3{}^{19}F_7$, 1260.7843; found, 1260.7826, Δ = 1.7 mmu

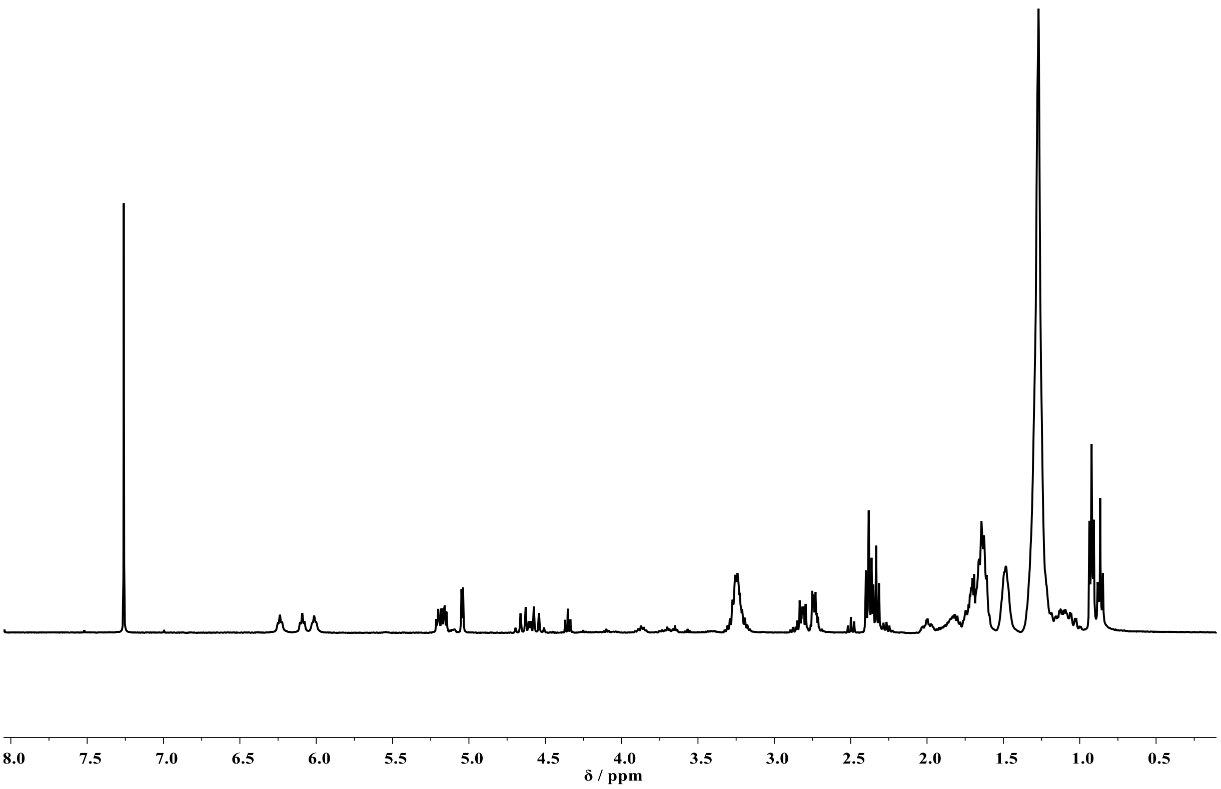

Supplementary Figure 142: ^{1}H-NMR of compound 95 measured in $CDCl_3$.

Passerini reaction

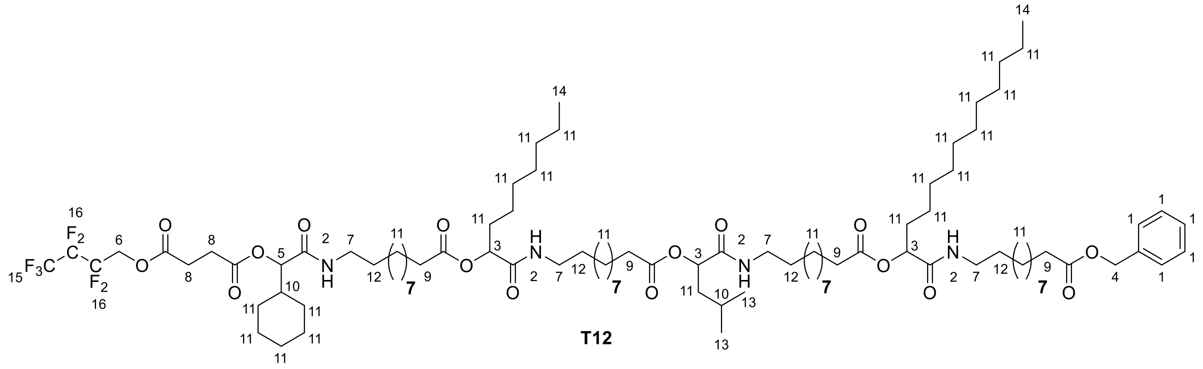

In a 50 mL round bottom flask, 264 mg of **95** (210 µmol, 1.00 eq.) was stirred in 3.00 mL DCM. Subsequently, 57.9 mg dodecanal **14h** (314 µmol, 1.50 eq.) and 94.7 mg of the monomer **M1** (314 µmol, 1.50 eq.) were added. The resulting reaction mixture was stirred at room temperature for 2 days. Afterwards, the crude mixture was dried under reduced pressure. The residue was adsorbed onto celite® and purified *via* column chromatography on silica gel eluting with a gradual solvent mixture of ethyl acetate and cyclohexane (3:1 → 2:1) to yield the passerini product **T12** as a pale highly viscous oil. (292 mg, 167 µmol, 79.7%).

R_f = 0.68 in cyclohexane / ethyl acetate (1:1).

IR (ATR): ν / cm^{-1} = 3302.0, 2922.6, 2852.3, 2165.9, 1739.0, 1656.3, 1540.6, 1466.3, 1369.9, 1228.7, 1171.8, 1022.2, 722.6, 697.9 (vw).

^{1}H-NMR (400 MHz, $CDCl_3$): δ / ppm = 7.41 – 7.28 (m, 5 H, CH_{Ar}^1), 6.21 (t, J = 5.9 Hz, 1 H, NH^2), 6.08 – 5.93 (m, 3 H, NH^2), 5.22 – 5.13 (m, 3 H, CH^3), 5.11 (s, 2 H, CH_2^4), 5.04 (d, J = 4.1 Hz, 1 H, CH^5), 4.72 – 4.46 (m, 2 H, CH_2^6), 3.34 – 3.14 (m, 8 H, CH_2^7), 2.91 – 2.68 (m, 4 H, CH_2^8), 2.43 – 2.29 (m, 8 H, CH_2^9), 2.03 – 1.57 (m, 26 H, CH^{10}, CH_2^{11}), 1.55 – 1.43 (m, 8 H, CH_2^{12}), 1.38 – 1.00 (m, 76 H, CH_2^{11}), 0.92 (t, J = 5.8 Hz, 6 H, CH_3^{13}), 0.90 – 0.84 (m, 6 H, CH_3^{14}).

^{13}C NMR (101 MHz, $CDCl_3$): δ / ppm = 173.82, 172.79, 172.61, 171.56, 170.93, 170.36, 170.01, 169.99, 168.96, 136.26, 128.68, 128.30, 78.58, 74.07, 72.77, 66.21, 42.13, 40.99, 39.91, 39.39, 39.36, 39.33, 34.46, 32.05, 31.86, 29.76, 29.71, 29.68, 29.60, 29.58, 29.51, 29.48, 29.43, 29.40, 29.35, 29.24 (d, J = 1.9 Hz), 29.00, 28.81, 27.15, 26.97, 26.94, 26.15, 26.02, 25.10, 25.08, 25.06, 24.90, 24.69, 23.27, 22.82, 22.75, 21.93, 14.26, 14.21.

^{19}F NMR (376 MHz, $CDCl_3$): δ / ppm = -85.14 (t, J = 9.6 Hz, 3 F, CF_3^{15}), -123.51 – -127.56 (m, 2 F, CF_2^{16}), -130.32 – -134.20 (m, 2 F, CF_2^{16}). Total integral of CF_2 region normalized with respect to the CF_3^{15} group = 4.

ESI-MS [*m/z*]: [M + H]$^+$ calculated for $^{12}C_{95}{}^{1}H_{159}{}^{16}O_{16}{}^{14}N_4{}^{19}F_7$, 1746.1712; found, 1746.1712, Δ = 0.0 mmu.

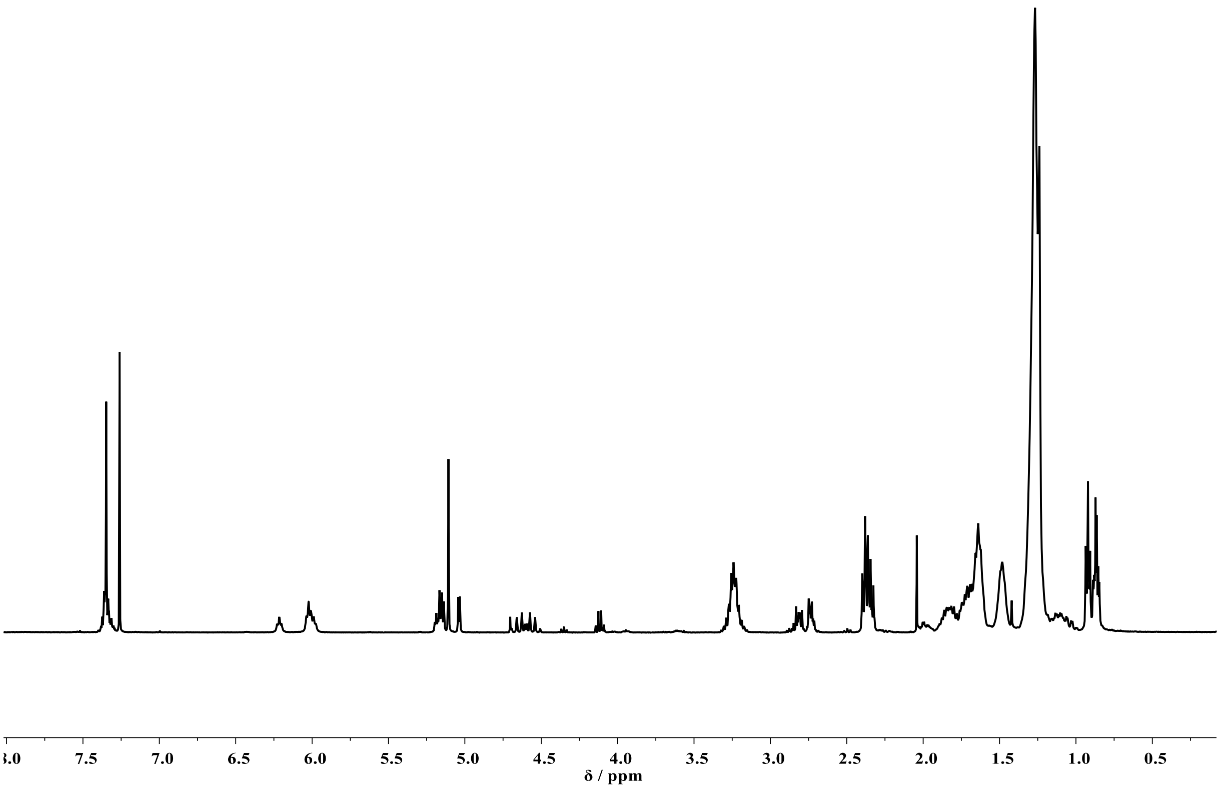

Supplementary Figure 143: ^{1}H-NMR of compound T12 measured in $CDCl_3$.

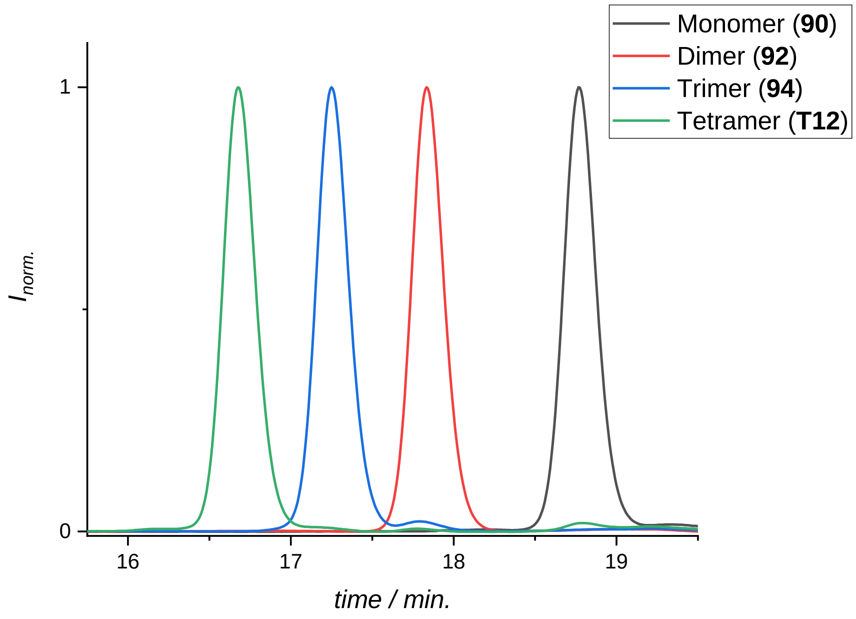

Supplementary Figure 144: SEC traces of the intermediates after each P3CR in the synthesis of product T12.

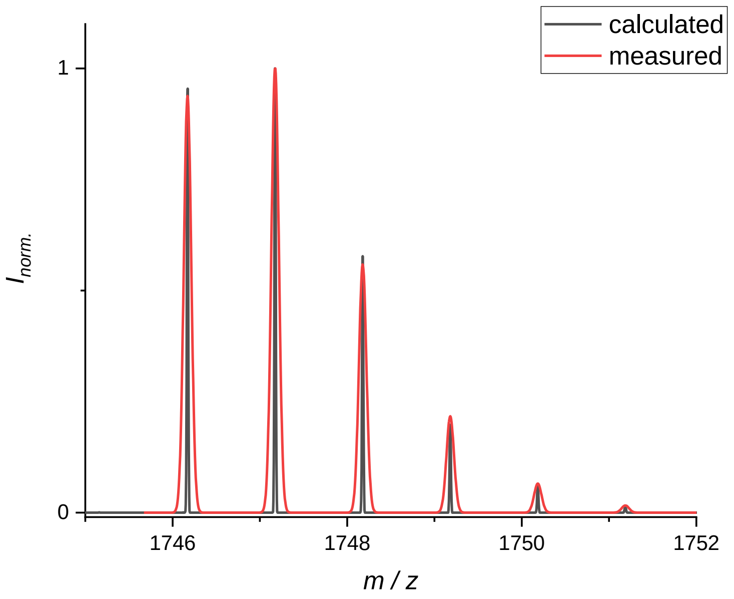

Supplementary Figure 145: High resolution ESI-MS measurement of T12. The observed isotopic pattern is compared with the calculated isotopic pattern obtained from mMass (black).

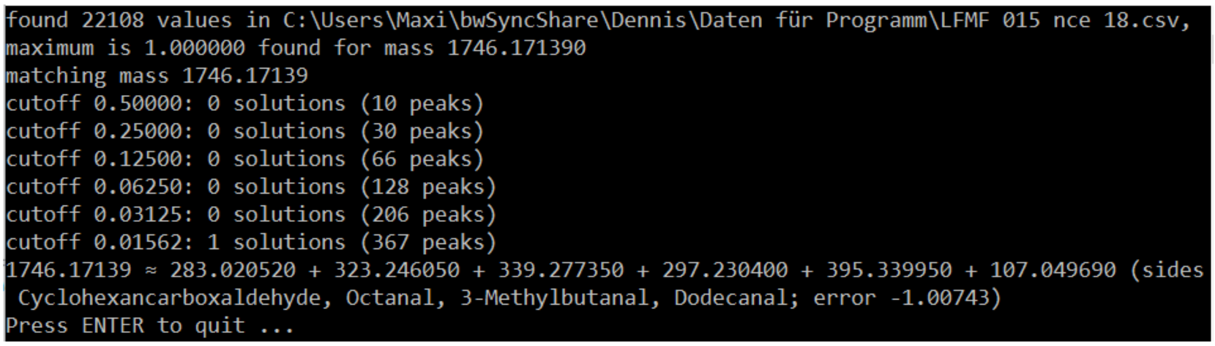

```
found 22108 values in C:\Users\Maxi\bwSyncShare\Dennis\Daten für Programm\LFMF 015 nce 18.csv,
maximum is 1.000000 found for mass 1746.171390
matching mass 1746.17139
cutoff 0.50000: 0 solutions (10 peaks)
cutoff 0.25000: 0 solutions (30 peaks)
cutoff 0.12500: 0 solutions (66 peaks)
cutoff 0.06250: 0 solutions (128 peaks)
cutoff 0.03125: 0 solutions (206 peaks)
cutoff 0.01562: 1 solutions (367 peaks)
1746.17139 ≈ 283.020520 + 323.246050 + 339.277350 + 297.230400 + 395.339950 + 107.049690 (sides
 Cyclohexancarboxaldehyde, Octanal, 3-Methylbutanal, Dodecanal; error -1.00743)
Press ENTER to quit ...
```

Supplementary Figure 146: Screenshot of the automated read-out of T12.

1.3.6 Equations

$$[M_{Molecule} + H]^{+} = \left[\left(M_{Start} + n * (M_{Backbone}) + \sum_{i=1}^{i=n} M_{Sidechain}^{i} + M_{End} + y * M(H)\right) + \mathrm{H}\right]^{+}$$

(Supplementary Equation 1)

n = number of repeating units,

y = (n-1)

M_{Start} = M (**Tag X**)

M_{End} = M (C_7H_7)

$M_{Backbone}$ = (M (monomer **M1**)) – M(C_7H_7)

$M_{Sidechain}$ = M (**2a**) or M (**2b**) or M (**2c**) or M (**2d**) or M (**2e**) or M (**2f**) or M (**2g**) or M (**2h**) or M (**2i**) or M (**2j**) or M (**2k**) or M (2**l**)

$M_{Backbone}$ is calculated with the mass of the monomer, which incorporates the protected acid (benzyl ester); however, in the iterative cycle, the benzyl ester is deprotected and further converted as the free acid compound. In order to take this into consideration in the formula, y is introduced as additional summand.

1.3.7 Tetramer mixture

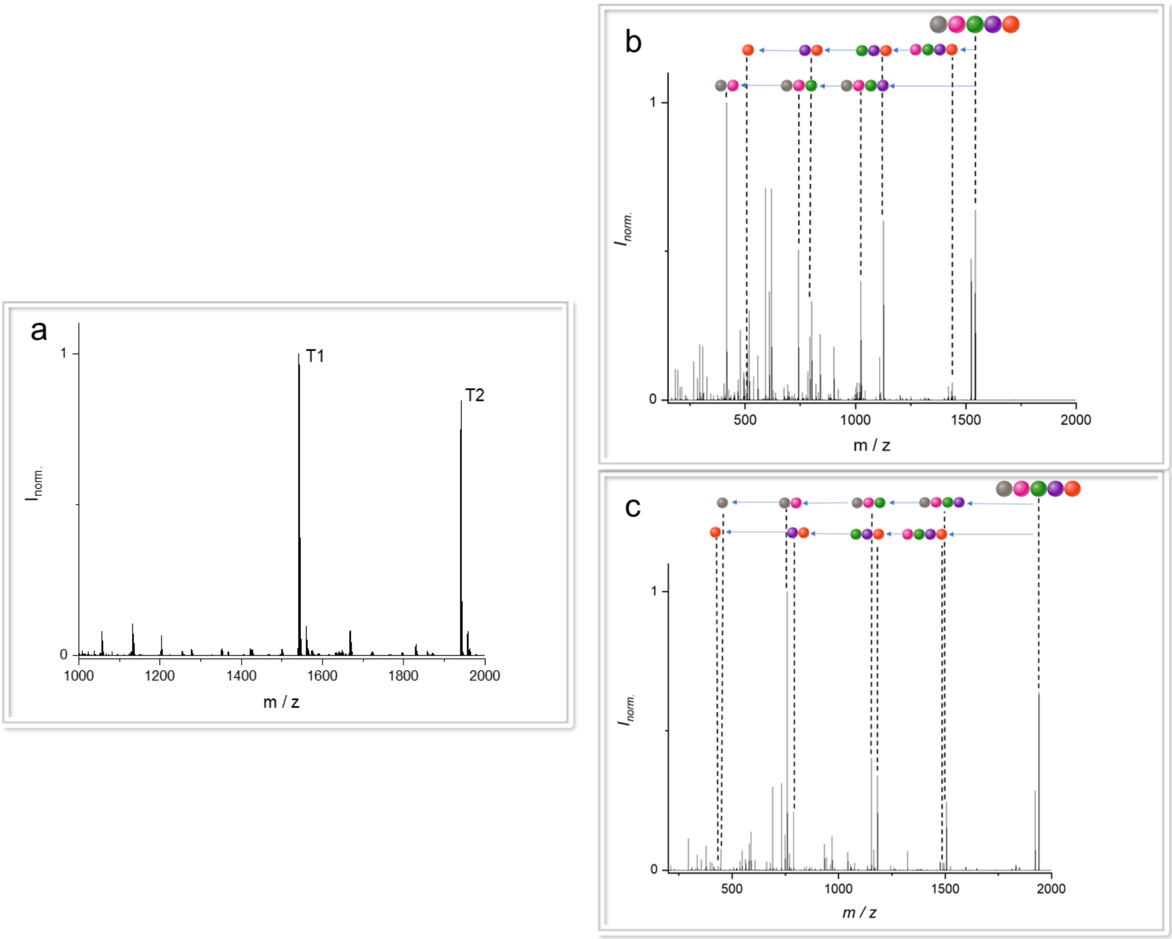

Supplementary Figure 147: Read-out of a mixture of two tetramers. a. ESI-MS spectrum of a mixture of two different tetramers **T1** and **T5** that was used for subsequent tandem ESI-MS/MS fragmentation. For the fragmentation, one of the respective molecule peaks was cihosen at a time. b. fragmentation and read-out of tetramer **T1**. c. fragmentation and read-out of tetramer **T5**.

1.3.8 MS and MS/MS Data’s

Tetramer T1:

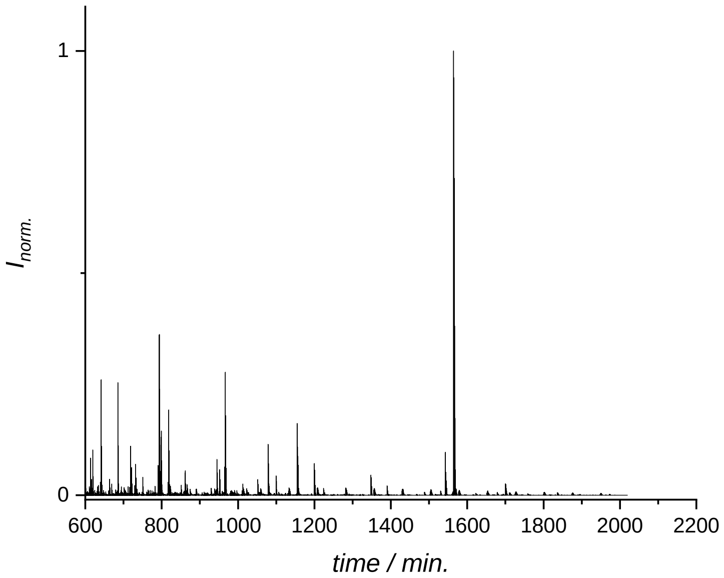

Supplementary Figure 148: ESI-MS spectra of T1, $[M+H]^+$ and $[M+Na]^+$ are visible.

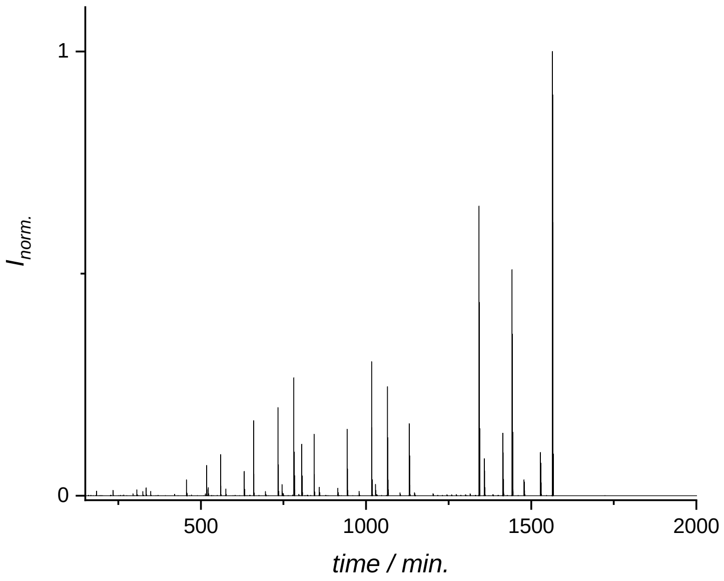

Supplementary Figure 149: ESI-MS/MS spectra of T1 with NCE 35.

Tetramer T2:

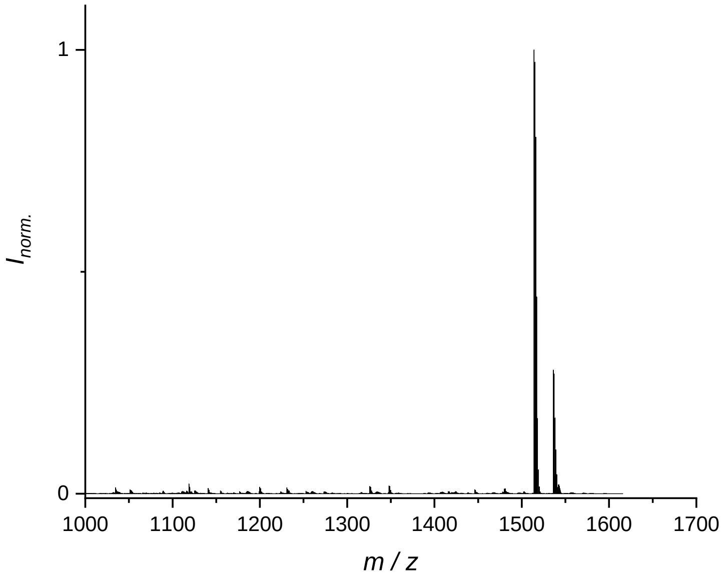

Supplementary Figure 150: ESI-MS spectra of T2, [M+H]$^+$ and [M+Na]$^+$ are visible.

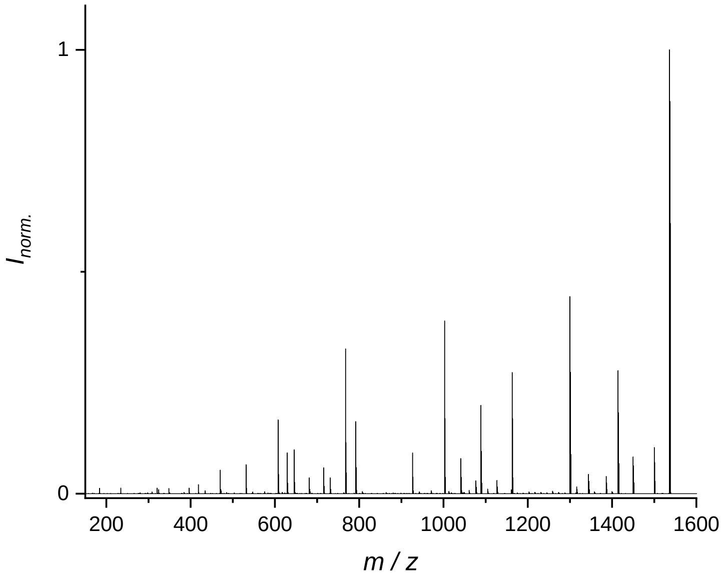

Supplementary Figure 151: ESI-MS/MS spectra of T2 with NCE 35.

Tetramer T3:

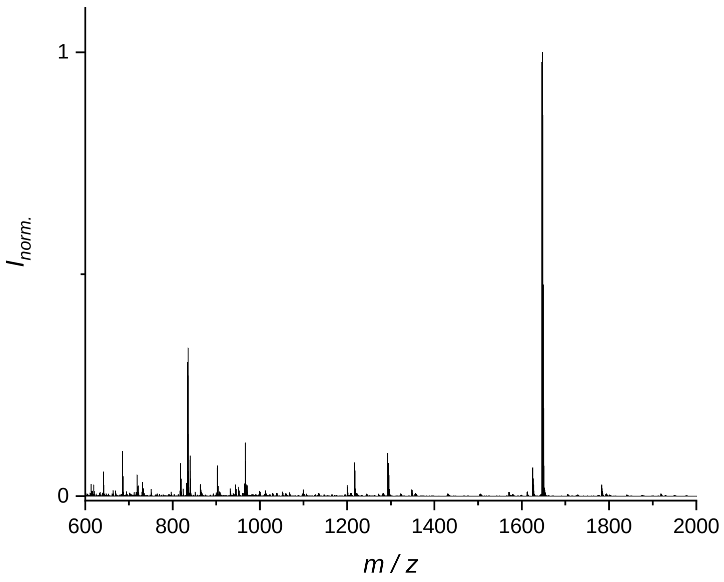

Supplementary Figure 152: ESI-MS spectra of T3, $[M+H]^+$ and $[M+Na]^+$ and $[M+Na]^{2+}$ are visible.

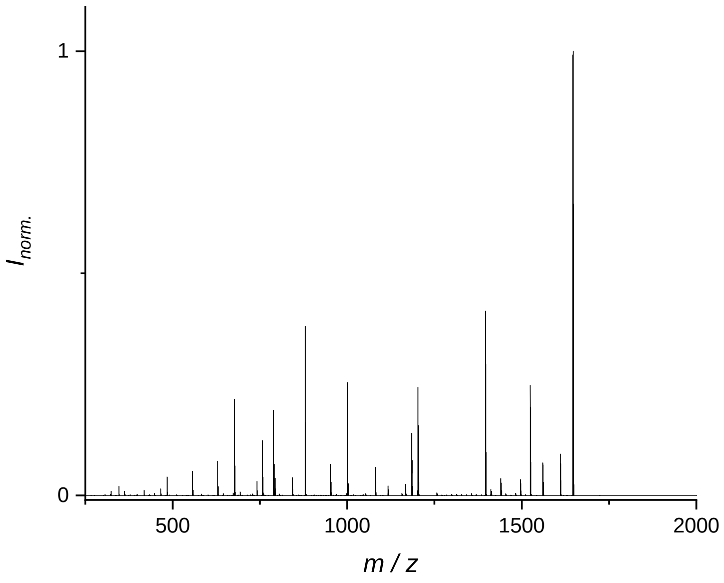

Supplementary Figure 153: ESI-MS/MS spectra of T3 with NCE 35.

Tetramer T4

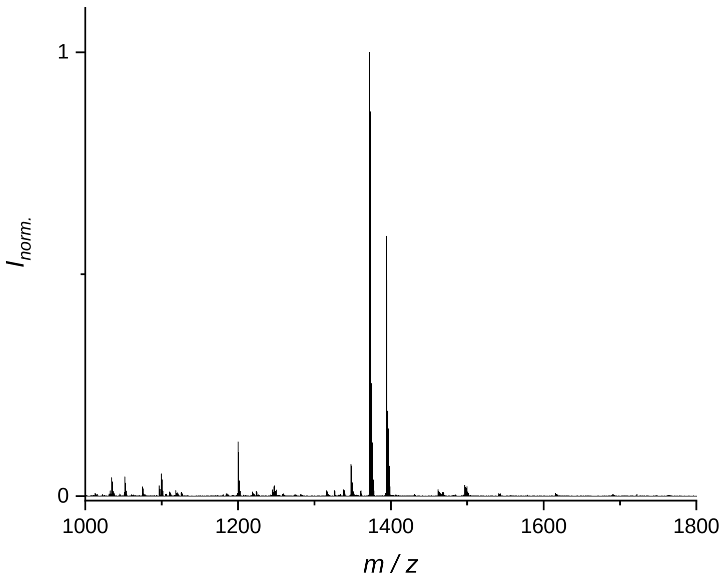

Supplementary Figure 154: ESI-MS spectra of T4, [M+H]$^+$ and [M+Na]$^+$ are visible.

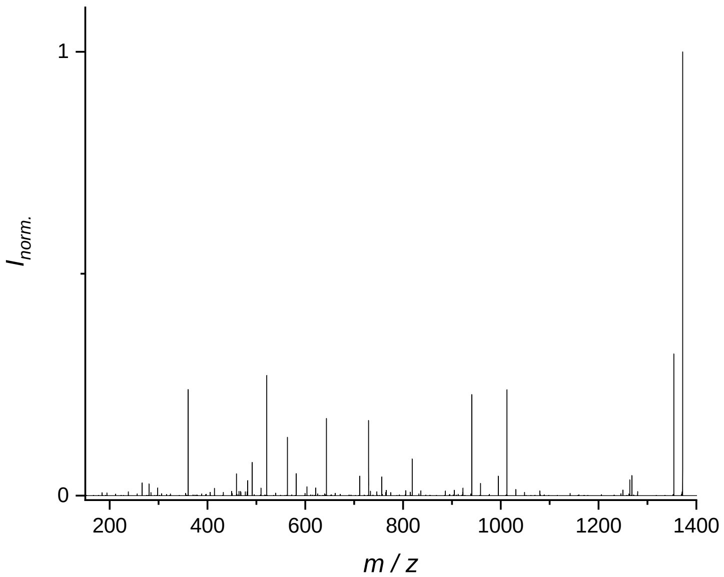

Supplementary Figure 155: ESI-MS/MS spectra of T4 with NCE 18.

Tetramer T5

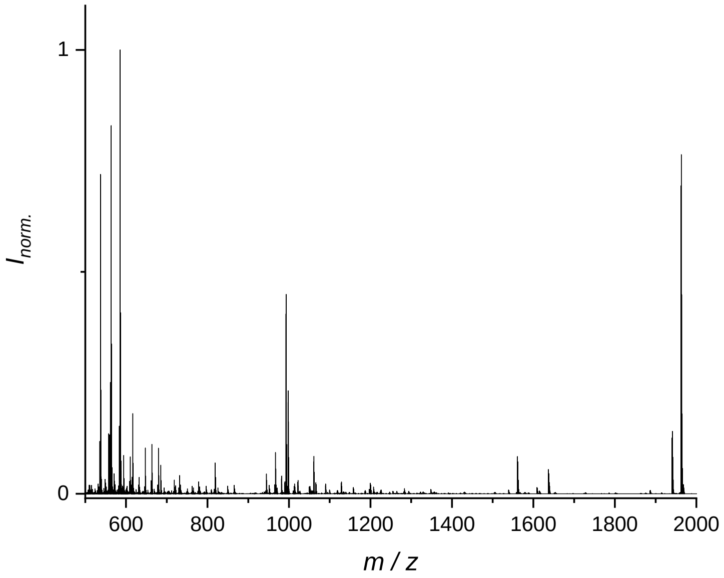

Supplementary Figure 156: ESI-MS spectra of T5, $[M+H]^+$ and $[M+Na]^+$ and $[M+Na]^{2+}$ are visible.

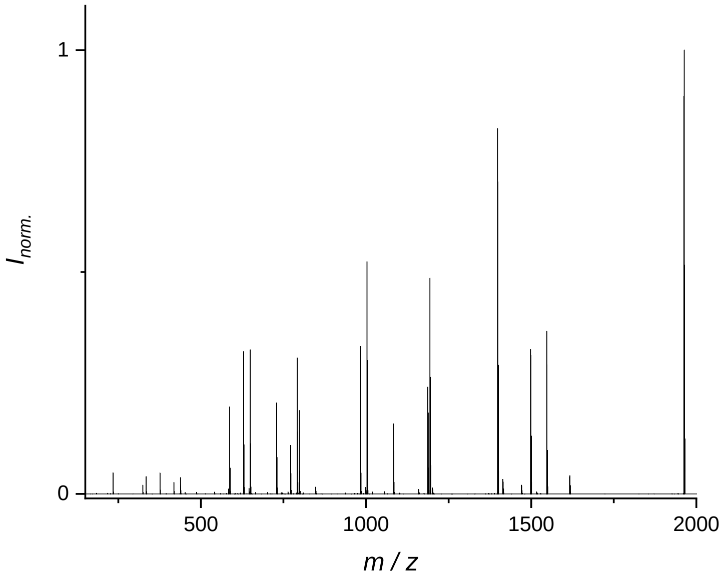

Supplementary Figure 157: ESI-MS/MS spectra of T5 with NCE 35.

Tetramer T6

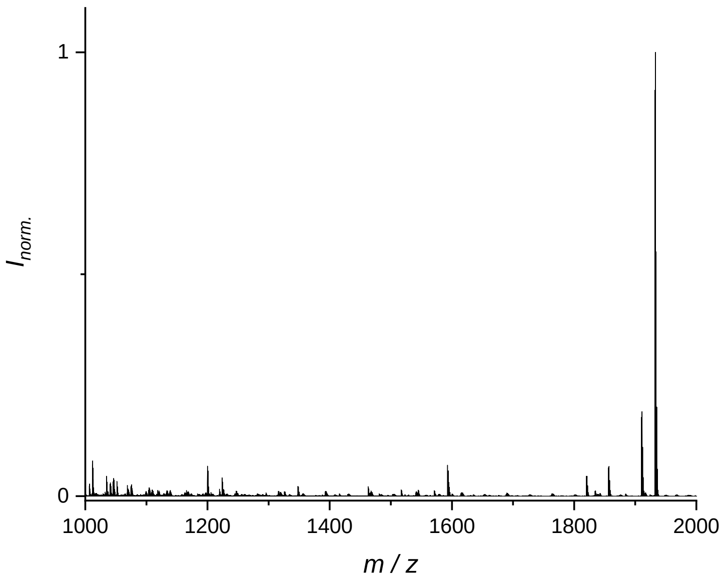

Supplementary Figure 158: ESI-MS spectra of T5, [M+H]$^+$ and [M+Na]$^+$ are visible.

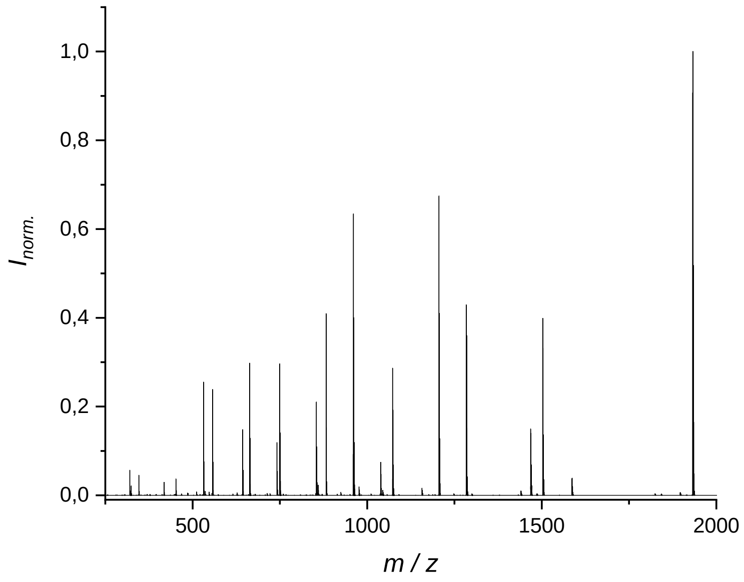

Supplementary Figure 159: ESI-MS/MS spectra of T6 with NCE 35.

Tetramer T7

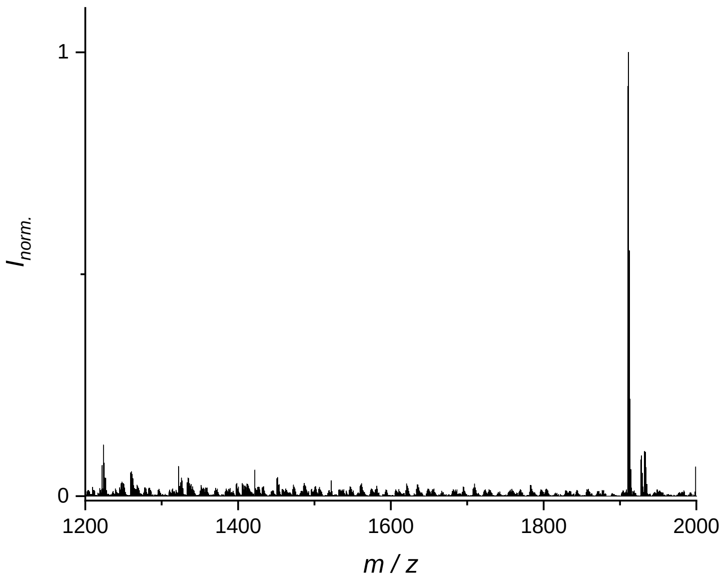

Supplementary Figure 160: ESI-MS spectra of T7, [M+H]$^+$ and [M+Na]$^+$ are visible.

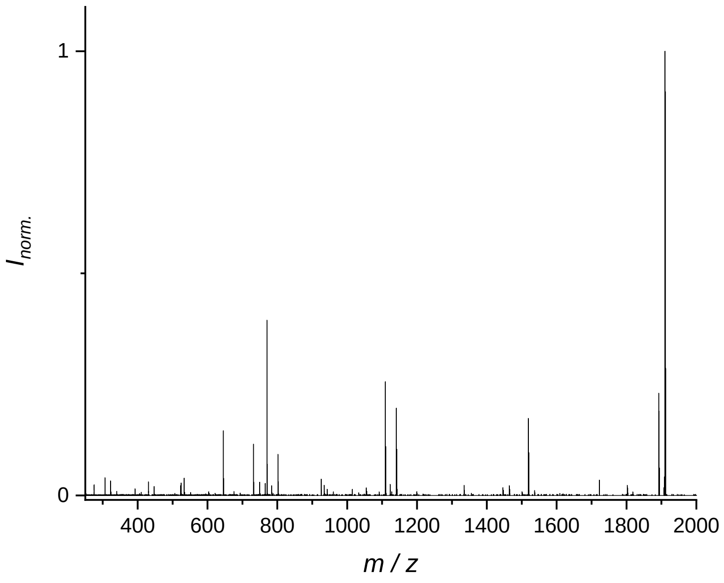

Supplementary Figure 161: ESI-MS/MS spectra of T7 with NCE 17.

Tetramer T8

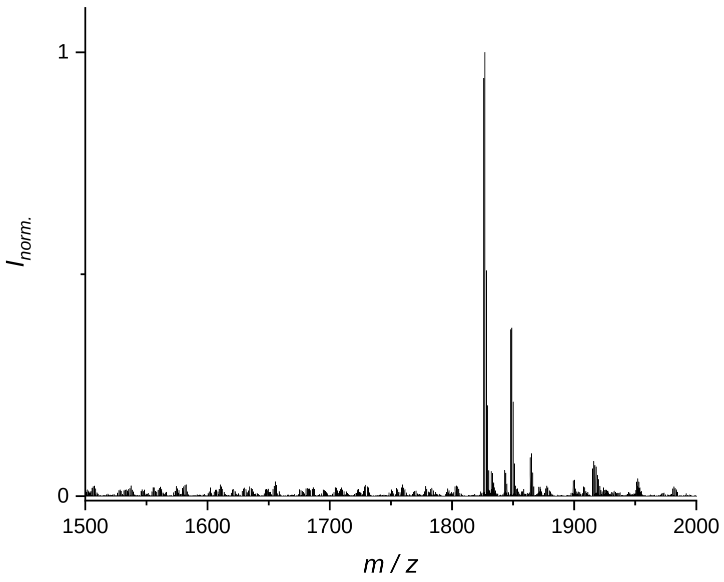

Supplementary Figure 162: ESI-MS spectra of T8, [M+H]$^+$ and [M+Na]$^+$ are visible.

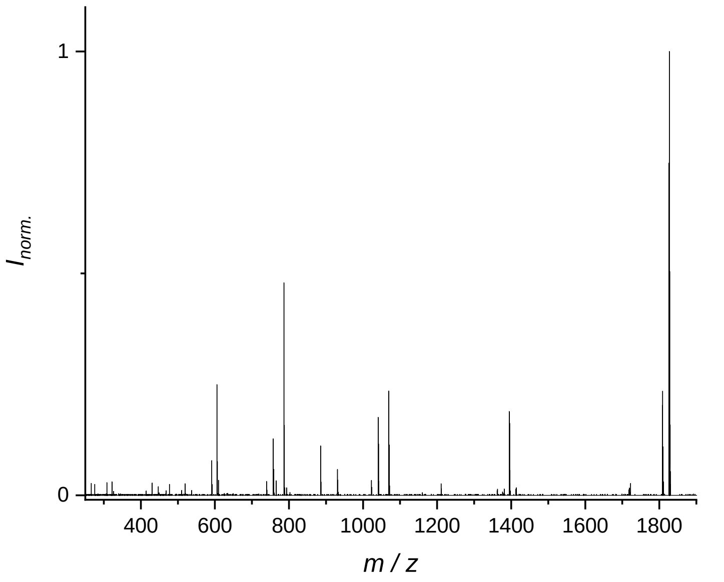

Supplementary Figure 163: ESI-MS/MS spectra of T8 with NCE 17.

Tetramer 9

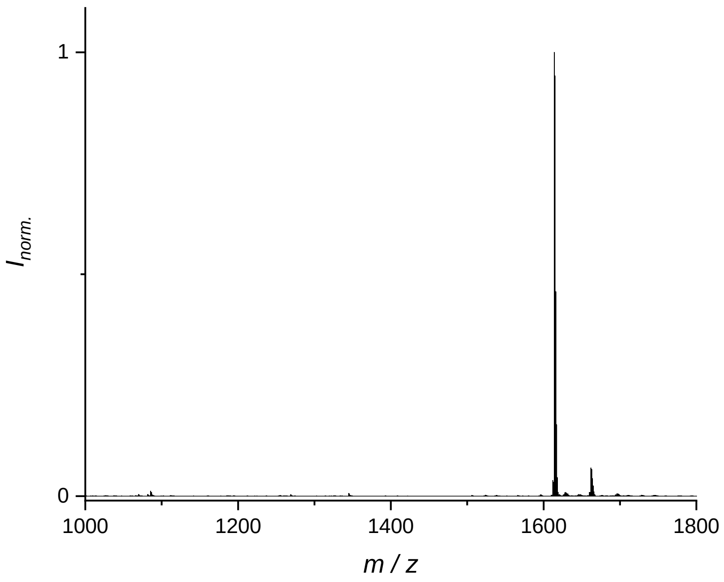

Supplementary Figure 164: ESI-MS spectra of T9, $[M+H]^+$ and $[M+Na]^+$ are visible.

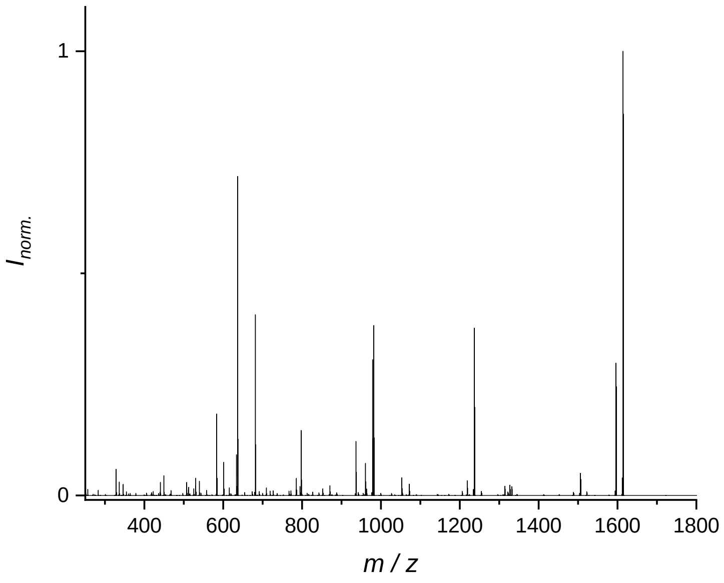

Supplementary Figure 165: ESI-MS/MS spectra of T9 with NCE 17.

Tetramer T10

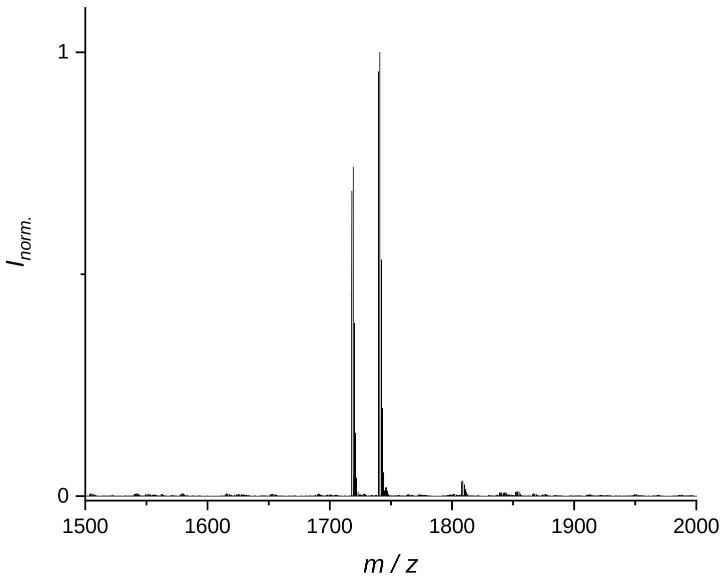

Supplementary Figure 166: ESI-MS spectra of T10, $[M+H]^+$ and $[M+Na]^+$ are visible.

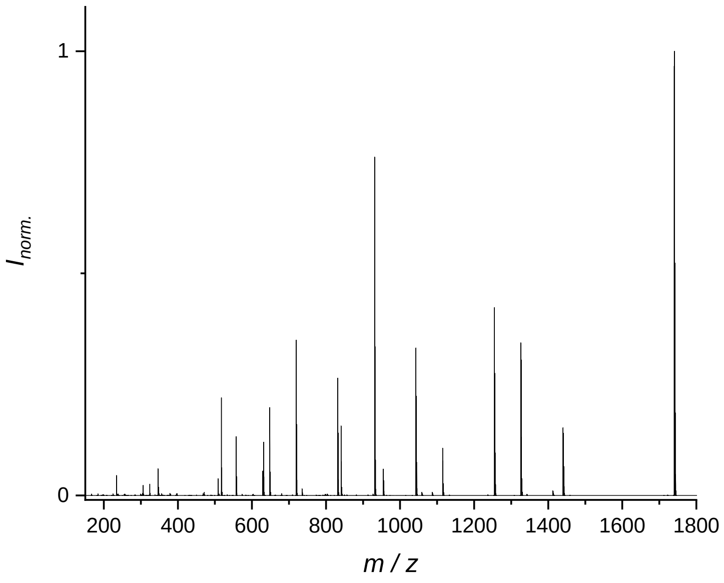

Supplementary Figure 167: ESI-MS/MS spectra of T10 with NCE 35.

Tetramer T11

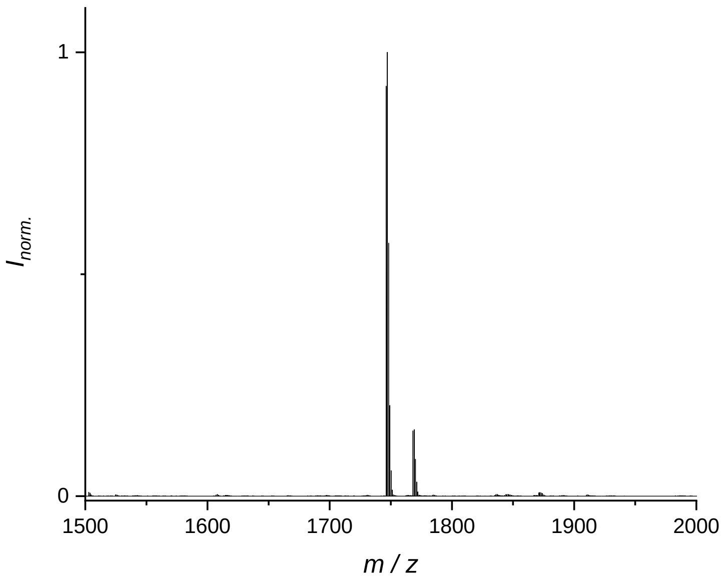

Supplementary Figure 168: ESI-MS spectra of T11, $[M+H]^+$ and $[M+Na]^+$ are visible.

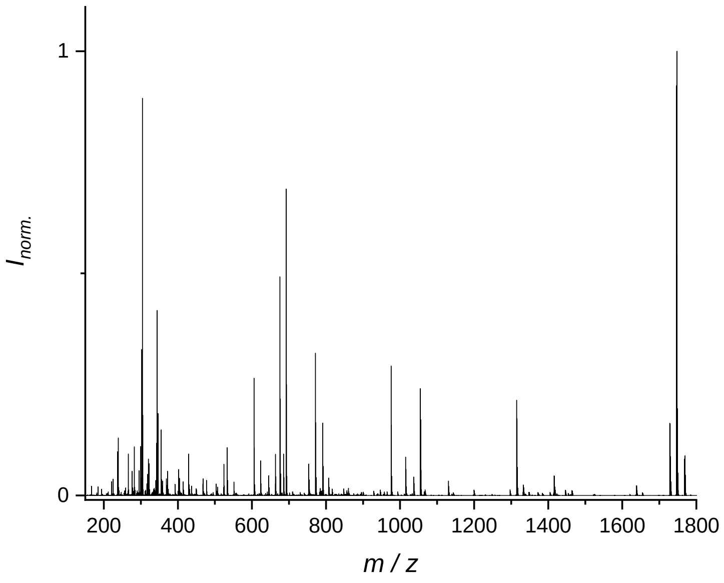

Supplementary Figure 169: ESI-MS/MS spectra of T11 with NCE 17.

Tetramer T12

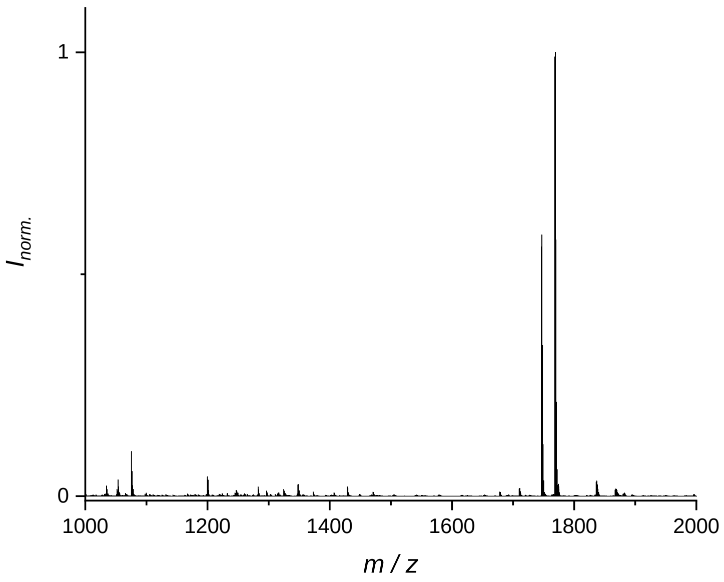

Supplementary Figure 170: ESI-MS spectra of T12, $[M+H]^+$ and $[M+Na]^+$ are visible.

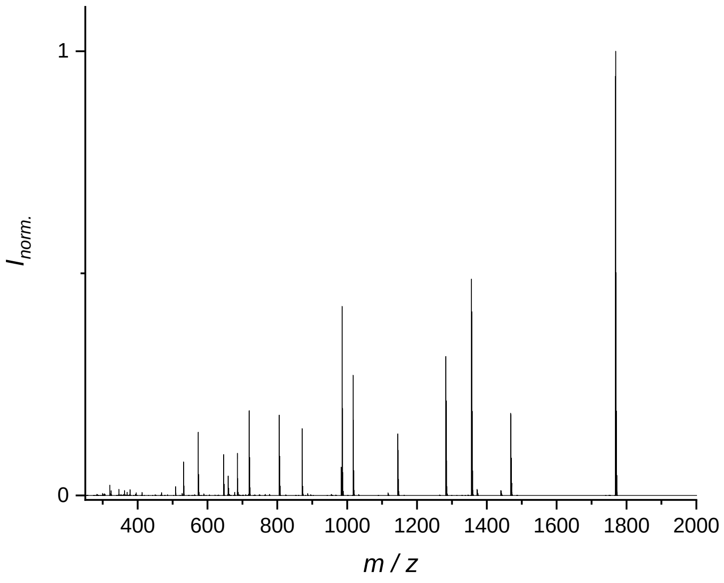

Supplementary Figure 171: ESI-MS/MS spectra of T12 with NCE 35.

1.4 Supplementary References

1. Solleder, S. C., Zengel, D., Wetzel, K. S. & Meier, M. A. R. A scalable and high-yield strategy for the synthesis of sequence-defined macromolecules. *Angew. Chemie - Int. Ed.* **55**, 1204–1207 (2016).
