# Supplementary material for: Reading mixtures of uniform sequence-defined macromolecules to increase data storage capacity
Source: Commun Chem. 2020 Dec 9;3:184. doi: 10.1038/s42004-020-00431-9 (PMC9814948; doi:10.1038/s42004-020-00431-9)
Supplement: Supplementary file 2 — Description of Additional Supplementary Files [file 42004_2020_431_MOESM2_ESM.pdf]

Description of Additional Supplementary Files

File Name: Supplementary Data 1
Description: a Python script, which was used for automated data analysis.
